# Fluorenone imidazolium salts as novel de Vries materials†

**DOI:** 10.1039/d0ra04650g

**Published:** 2020-06-23

**Authors:** Korinna Bader, Carsten Müller, Yann Molard, Angelika Baro, Philipp Ehni, Jakob Knelles, Sabine Laschat

**Affiliations:** Institut für Organische Chemie, Universität Stuttgart Pfaffenwaldring 55 70569 Stuttgart Germany sabine.laschat@oc.uni-stuttgart.de; Institut für Physikalische Chemie, Universität Stuttgart Pfaffenwaldring 55 70569 Stuttgart Germany; CNRS, ISCR-UMR 6226, ScanMAT-UMS 2001, University Rennes 35000 Rennes France yann.molard@univ-rennes1.fr

## Abstract

In ionic liquid crystals (ILCs) tilted mesophases such as SmC required for electro-optic devices are quite rare. We report a design concept that induced the SmC phase and enabled de Vries-like behaviour in ILCs. For this purpose, we synthesized and characterized a library of ILC derivatives ImR(On,Ym)X which consist of a rigid central fluorenone core containing an alkoxy or thioether side chain and connected *via* a flexible spacer to an imidazolium head group. The mesomorphic properties were studied by differential scanning calorimetry (DSC), polarizing optical microscopy (POM) and X-ray diffraction (XRD). Temperature-dependent measurements of smectic layer spacing *d* by small-angle X-ray scattering (SAXS) and of optical tilt angles by POM demonstrate that ILCs ImR(On,Ym)X undergo SmA–SmC phase transitions with maximum layer contraction values between 0.4% and 2.1%. The lowest reduction factor *R* of 0.2 at the reduced temperature *T* − *T*_AC_ = −10 K was calculated for Im(O12,S14)Br. Electron density calculations indicated a bilayer structure. Furthermore, temperature dependent emission studies show that self-assembling has a strong influence on the emission intensity of these ILCs.

## Abstract 2

ILCs consisting of cationic head group–spacer–fluorenone central core–side chain show de Vries-like behaviour.

## Graphical abstract

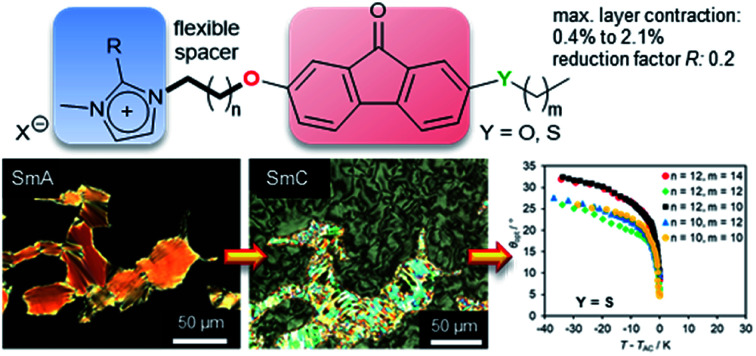

## Introduction

**Scheme 1:**
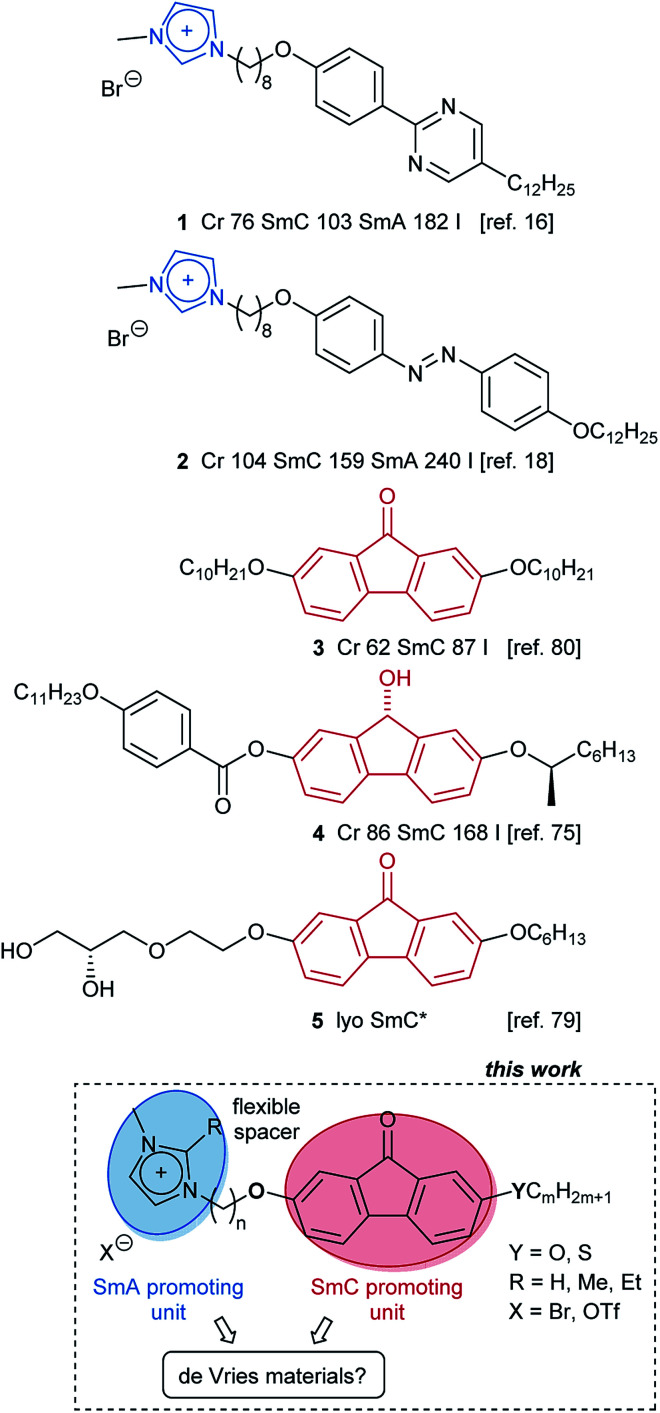


Ionic liquid crystals (ILCs) combine the physical properties of ionic liquids with those of thermotropic liquid crystals^1–8^ and thus provide highly desirable materials for anisotropically ordered 1D ion conductors in organic solar cells^9,10^ and as solid electrolytes in lithium ion batteries.^11^ While ILCs share many similarities with neutral thermotropic liquid crystals, they differ in the occurrence of certain mesophases.^1–8^ The strong tendency of ILCs to nanosegregate ionic and non-charged segments during liquid crystalline self-assembly leads to a strong preference of the non-tilted SmA phase as their archetypal mesophase, while for example the tilted SmC phase is much less common among ILCs.^12–16^ We have previously identified ILCs 1, 2 showing rare SmC phases (Scheme 1), which consist of a calamitic core unit carrying one side chain and a flexible spacer connected to a cationic head group.^15–18^ Upon examination of the order parameter of the SmA phase of these compounds it was found that the long range orientational order (*S*_2_) in the SmA phase was much smaller as compared to the values obtained for SmA phases from non-ionic liquid crystals.^19–22^ On the other hand, the 1D translational order (smectic order parameter *ε*) of the SmA phase of ILCs is much larger^17,18^ as compared to non-ionic SmA phases.^23–26^ Thus ILCs possess a high lamellar order but only a low long range orientational order as compared to neutral liquid crystals and in that respect behave similar to the so called de Vries materials.^26–29^ De Vries materials have created much interest recently, because ferroelectric displays based on de Vries materials might overcome limitations of current nematic LC displays such as limited resolution and low optical efficiency.^30–47^ One of the characteristic features of de Vries materials is their tilting transition without defect generation. These materials show no or only very low layer contraction upon the SmA to SmC phase transition, enabling ferroelectric displays without zig–zag defects caused by the chevron-like arrangement of the molecules in the meso- phase. With regard to the design of calamitic liquid crystals displaying SmA and SmC phases and de Vries behaviour most previous work has focused on 5-phenylpyrimidines,^39,41,43,48–53^ 2-phenylpyrimidines,^54–58^ biphenyls,^40,44,59–64^ thiadiazoles,^54,65^ aroylhydrazones^66^ and bent-core mesogens.^37,67–74^ Lemieux and Ivanov have reported independently for non-charged liquid crystals 3–5 that a rigid fluorenone core promotes formation of the SmC phase.^75–80^ As mentioned above, in contrast to neutral calamitic liquid crystals ILCs are rather reluctant to form SmC phases. Despite the few known examples,^12–18^ it is still an ongoing challenge to develop design principles for ILCs displaying both SmA and SmC phases. Moreover, ILCs with de Vries properties should provide a general insight into the driving forces for layer contraction and layer tilting of ionic mesogens and thus are relevant for electro-optic devices.^81^ Thus, we were curious, whether combination of a SmA-promoting unit, such as an imidazolium head group with a SmC-promoting calamitic core, *i.e.* fluorenone, tethered together *via* a flexible alkyl spacer would eventually lead to ILCs displaying both SmA and SmC phases and a minimal layer contraction, *i.e.* de Vries-like behaviour. Our results reveal that such merging of concepts is indeed successful and provides ILCs with a very broad mesophase stability, unique de Vries behaviour and solid state luminescence. The results are discussed below.

## Results and discussion

### Synthesis of fluorenone imidazolium ILCs

**Scheme 2:**
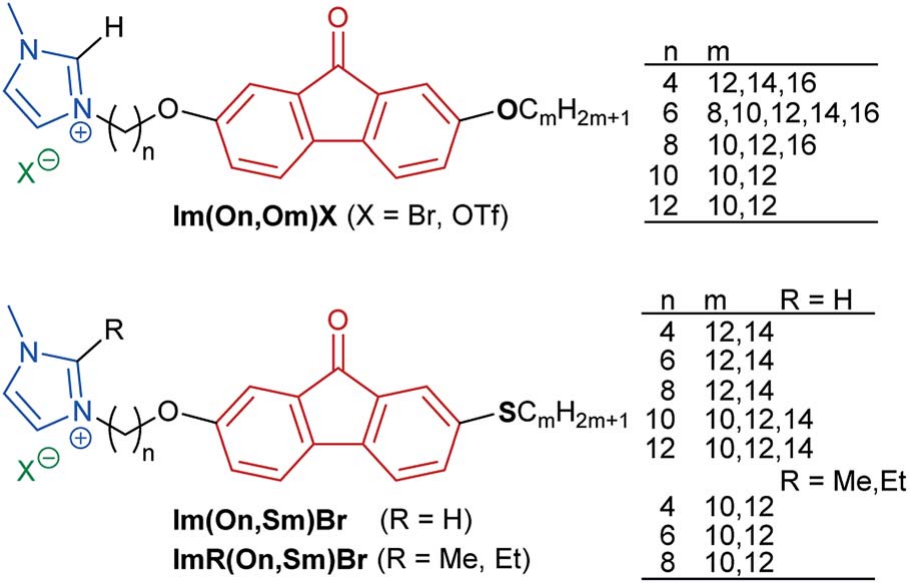
Library of fluorenone ionic liquid crystals (ILCs).

In order to obtain structure–property relationships, a library of fluorenone ILCs Im(On,Om)X and ImR(On,Sm)Br was prepared (Scheme 2), where the following structural parameters were varied: alkoxy *vs.* thioether side chain with different chain lengths *m*, spacer lengths *n*, C-1 substituent R at the imidazolium head group and counterion. These target structures required a synthetic approach providing access to unsymmetrical fluorenones.

**Scheme 3:**
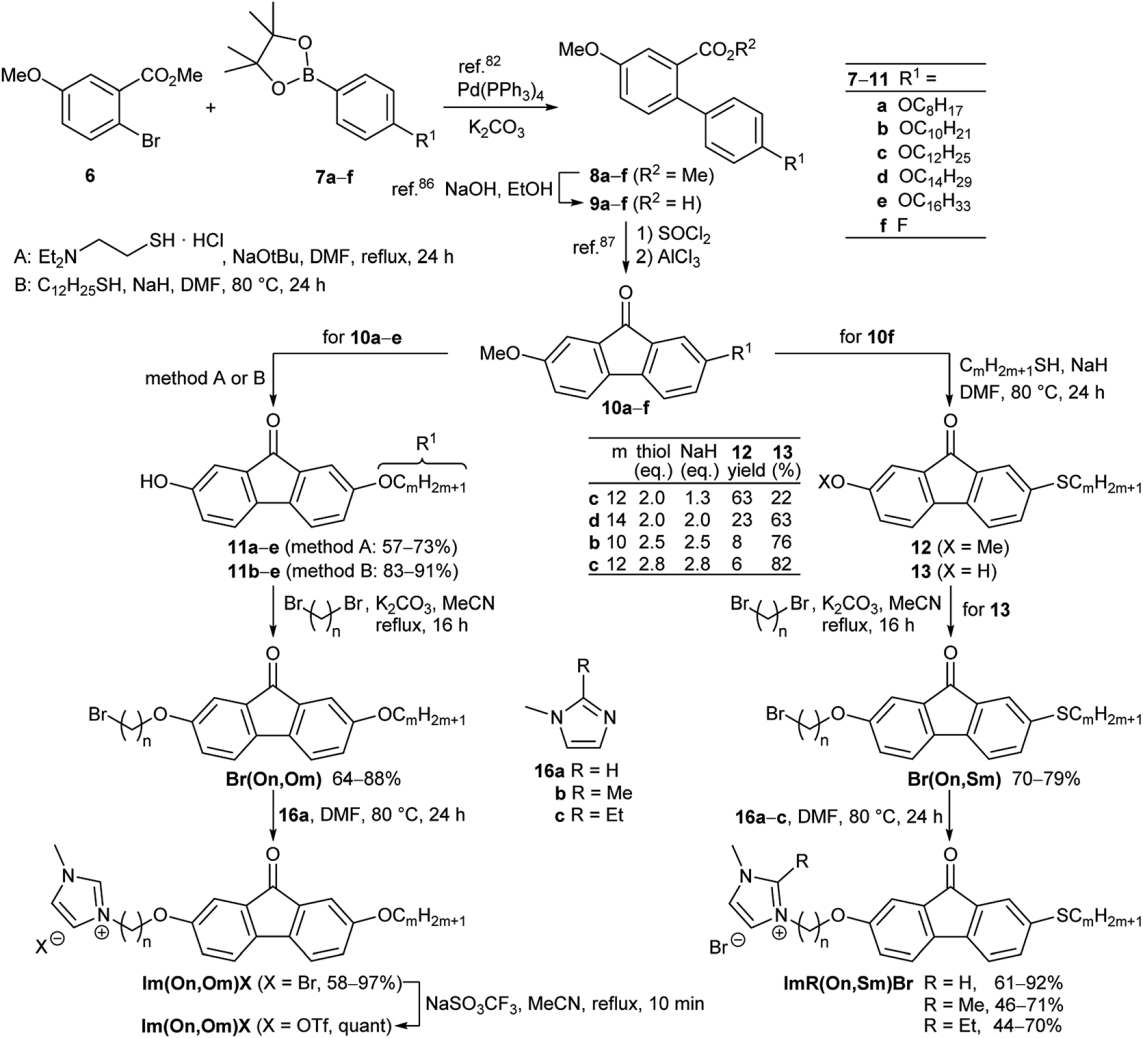
Synthesis of the imidazolium salt ILCs Im(On,Om)X and ImR(On,Sm)Br.

As shown in Scheme 3, the synthesis commenced with a sequential Suzuki cross coupling^82^ of methyl 6-bromo-3-methoxybenzoate 6 with known borolanes 7a–f^83–85^ to give the biphenyl esters 8a–f.

Subsequent saponification^86^ provided biphenyl carboxylic acids 9a–f, which were submitted to Friedel–Crafts acylation^87^*via* activation with thionyl chloride and reaction with AlCl_3_ to give the fluorenones 10a–f. For the selective deprotection of the methyl ether 10a–e two protocols employing thiols were examined.

According to a method by Magano^88^*N*,*N*-diethyl-aminoethanethiol was deprotonated with NaO*t*Bu in DMF at room temperature and then treated with the respective fluorenone 10a–e to give the hydroxyfluorenones 11a–e in moderate yields (method A). Alternatively, dodecanethiol was used together with NaH in DMF at 80 °C (method B) following a procedure by Chae^89^ resulting in higher yields of the desired hydroxyfluorenones 11b–e with alkoxy side chains.

For the corresponding hydroxy fluorenones 13 with thioethers, a nucleophilic displacement of aryl fluoride by thionucleophiles as reported by Kaszyński^90^ was planned as a key step. Upon treatment of 10f with 2.0 equiv. of dodecanethiol and 1.3 equiv. of NaH in DMF at 80 °C,^90^ the desired nucleophilic displacement competed with demethylation resulting in a mixture of 63% 12c and 22% of 13c (Scheme 3). This one-pot nucleophilic substitution/demethylation sequence could be optimized by employing equimolar amounts of thiol and NaH, *e.g.* giving 6% of 12c and 82% of the target alcohol 13c.

The hydroxyfluorenones were submitted to Williamson etherification with 1,ω-dibromoalkanes^91^ to provide the corresponding ω-bromoalkoxyfluorenones Br(On,Om) and Br(On,Sm) in good yields, followed by reaction with the methylimidazoles 16a–c (Scheme 3).^16^ While the 2-*H*-substituted imidazolium salts Im(On,Om)Br, Im(On,Sm)Br could be isolated in pure form after chromatography through HBr-treated silica, the corresponding 2-methyl- and 2-ethyl-substituted imidazolium bromides ImR(On,Sm)Br (R = Me, Et) required an additional recrystallization step, and thus lower overall yields were obtained. For a selected series Im(On,Om)Br the bromide counterion was replaced by triflate *via* salt metathesis^92^ (Scheme 3).

### Mesomorphic properties of the ω-bromoalkoxyfluorenones

First, the mesomorphic properties of the ILC precursors, *i.e.* the ω-bromoalkoxyfluorenones Br(On,Ym) were examined by differential scanning calorimetry (DSC), polarizing optical microscopy (POM) and X-ray diffraction (WAXS, SAXS). The DSC data are summarized in Fig. S1–S3, Tables S1 and S2 (see ESI†). Both linking atoms Y, side chain lengths *m* and spacer length *n* had a pronounced influence on the phase behaviour of Br(On,Ym).

**Fig. 1:**
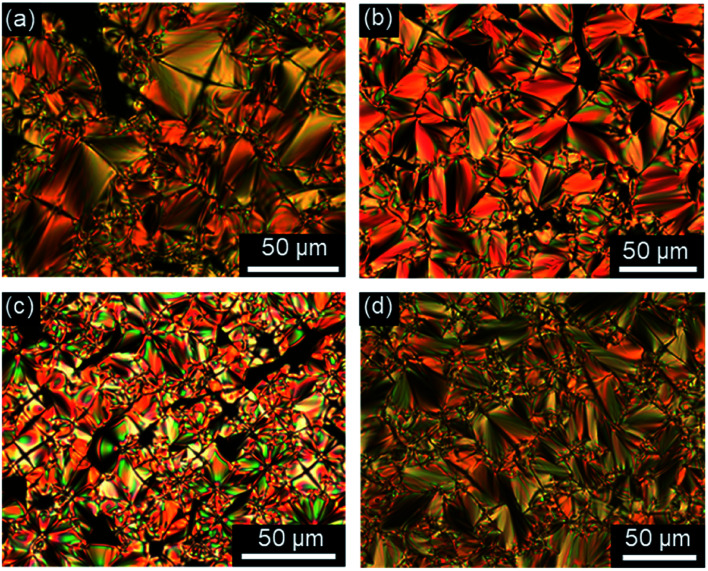
Focal-conic fan textures as seen between crossed polarizers upon cooling from the isotropic liquid (cooling rate 5 K min^−1^, magnification ×200). (a) Br(O4,O12) at 65 °C, (b) Br(O6,O12) at 71 °C, (c) Br(O8,S12) at 61 °C, and (d) Br(O10,O12) at 70 °C.

All ω-bromoalkoxyfluorenones Br(On,Om) showed enantiotropic mesomorphism. The DSC curves reveal supercooling for the mesophase-to-crystalline transition upon cooling from the isotropic liquid while clearing temperatures are maintained in heating/cooling scans. It should be noted that supercooling is quite common among ILCs^93–97^ and was recently rationalized by cationic clustering.^98^ Under the POM Br(On,Om) displayed focal-conic fan textures, which are characteristic of SmA phases (Fig. 1a, b and d). The broadest SmA phase in the series Br(O6,Om) with various chain lengths *m* was found for Br(O6,O10). Upon variation of the spacer lengths *n* in the series Br(On,O12) the broadest mesophase widths of 21–22 K were detected for *n* = 8, 10. Thus both very short and very long spacers seem to disfavour lamellar mesophases. In contrast, only ω-bromoalkoxyfluorenones Br(O6,S10), Br(O8,S10), Br(O8,S12), and Br(O10,S14) with a thioether side chain showed monotropic mesomorphism upon cooling. The clearing transitions ranged between 55 and 63 °C and melting temperature between 30–58 °C. The decreased clearing temperatures and resulting destabilization of the SmA phase by thioethers agree well with previous reports on calamitic phenylpyrimidines.^99,100^ However, the increase of melting temperatures of Br(On,Sm) in comparison with Br(On,Om) is in contrast to these reports where thioethers led to lowering of melting points.

**Fig. 2:**
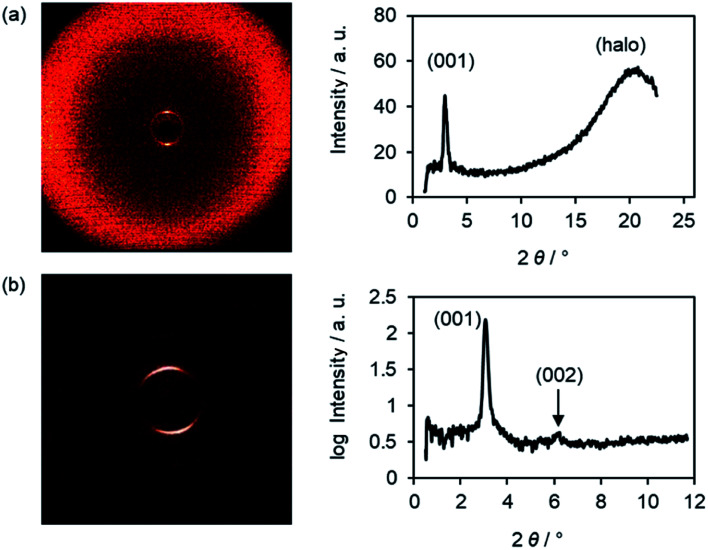
(a) Wide-angle scattering (WAXS) and (b) small-angle scattering (SAXS) profile and the corresponding diffraction pattern of Br(O6,O8) at 45 °C.

XRD experiments gave further insight into the phase geometry. The WAXS profile of an oriented sample of Br(O6,O8) showed a strong (001) layer reflection and a diffuse halo, which was tilted by 90° with respect to the (001) reflex, indicating an orthogonal SmA phase (Fig. 2a). In the SAXS profile a weak second order (002) reflection was visible besides the intense (001) reflection (Fig. 2b). From the (001) reflex the layer distance *d* was calculated according to equation *nλ* = 2*d* sin *θ* and plotted against the temperature (Fig. S23, ESI†). Typical for SmA phases, layer distances *d* decreased with increasing temperature due to decreased orientational order with increasing temperature. Furthermore, with increasing chain lengths the *d* values increased by 2.5 Å, which is equivalent to two CH_2_ units. The exclusive formation of SmA phases of the ω-bromoalkoxyfluorenones Br(On,Ym) can be rationalized by the presence of SmA-promoting halogens as suggested by Giesselmann and Lemieux,^101^*i.e.* reduced electrostatic repulsion between alkyl chains and improved van der Waals interaction between the aryl units rather than polar interactions at the interfaces between the smectic layers as was proposed by Goodby.^102^

### Mesomorphic properties of the fluorenone ILCs

**Fig. 3:**
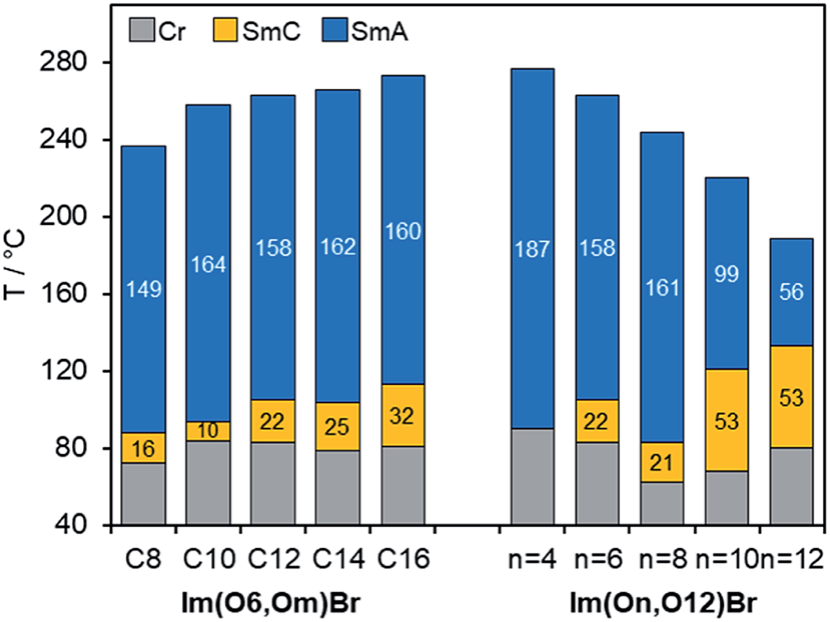
Mesophase stabilities of Im(O6,Om)Br and Im(On,O12)Br with varying alkoxy side chain *m* and varying spacer lengths *n*, respectively. The values in the bar diagram correspond to the mesophase temperature ranges. Phase transitions were determined by DSC (1^st^ heating) and POM (SmA–SmC transition). Im(O12,O12)Br (3^rd^ heating).

Phase transition temperatures and enthalpies of fluorenone ILCs Im(On,Om)X were determined by DSC from the first heating scans (Table S3 and Fig. S4, ESI†). Due to thermal decomposition of the samples caused by high isotropization temperatures in subsequent heating/cooling cycles no further transitions were detectable. In most cases SmC–SmA phase transitions were undetectable by DSC but were visible under the POM. Only in a few cases weak first order transitions with very small enthalpy changes were detected by DSC, which is in good agreement with previous work (Fig. S13–S17, ESI†).^53,103^ As exemplified for Im(O6,Om)Br in Fig. 3, the melting points remained relatively constant at 72–84 °C, while the clearing points were much stronger affected by the alkyl chain lengths *m* and increased from 237 °C for Im(O6,O8)Br up to 273 °C for Im(O6,O16)Br resulting in the broadest mesophase (192 K) for Im(O6,O16)Br. All ILCs within this series showed small SmC phases (10–32 K) and relatively broad SmA phases (149–164 K).

In contrast, Im(O12,Om)Br (*m* = 10,12) with a long C12 spacer showed no evidence for decomposition and thus DSC curves of three heating/cooling cycles were fully reproducible (Fig. S4, ESI†). The small transition enthalpies of 0.5–0.6 kJ mol^−1^ are typical for SmC–SmA transitions.^18^

As shown for Im(On,O12)Br with increasing spacer lengths *n* clearing temperatures decreased considerably (Fig. 3), which can be rationalized by the increased flexibility of the ILC molecules with larger spacers resulting in a decreased lamellar order. This trend is in agreement with previous observations on azobenzene ILCs.^17^ It should be noted that the behaviour of ILCs consisting of cationic group–spacer–mesogenic core–side chain is more complex as compared to ILCs containing side chain–cationic group–side chain.

Melting temperatures changed only little (68–90 °C), however, the stability of the SmC phase was strongly affected by variation of the spacer lengths *n*. While Im(O4,O12)Br showed only a monotropic SmA phase, derivatives with *n* > 4 displayed also enantiotropic SmC phases under the POM. Furthermore, the stability increased with increasing spacer lengths *n*, resulting in the broadest SmC phase (53 K) for *n* = 10, 12, while SmC phase widths of 21–22 K were found for derivatives with *n* = 6, 8 (Fig. 3).

Various experimental studies from the literature^20,104^ including very recent theoretical work by Saielli^105^ described a strong influence of the counterion on transition temperatures and mesophase type. Therefore, we surmised that an exchange of the counterion might be helpful to overcome the thermal decomposition. However, the triflate counterion decreased the clearing temperature significantly to 114–130 °C only for Im(O6,O8)OTf and Im(O8,O10)OTf. The triflate was stable and the complete phase sequence was reproducible over three heating/cooling cycles (Fig. S5, ESI†). ILCs Im(On,O14)OTf (*n* = 4, 6) showed only a slight decrease of the clearing temperature and thus isotropization was still accompanied by thermal decomposition. Most importantly, the triflate counterion affected the mesophase type, resulting in loss of the SmC phase. Similar results were reported by Trbojevic for guanidinium ILCs connected *via* a flexible alkyl spacer to a rod-like biphenyl unit,^15^ where replacement of chloride by triflate decreased the clearing transition but suppressed the SmC phase in favor of the SmA phase.

**Fig. 4:**
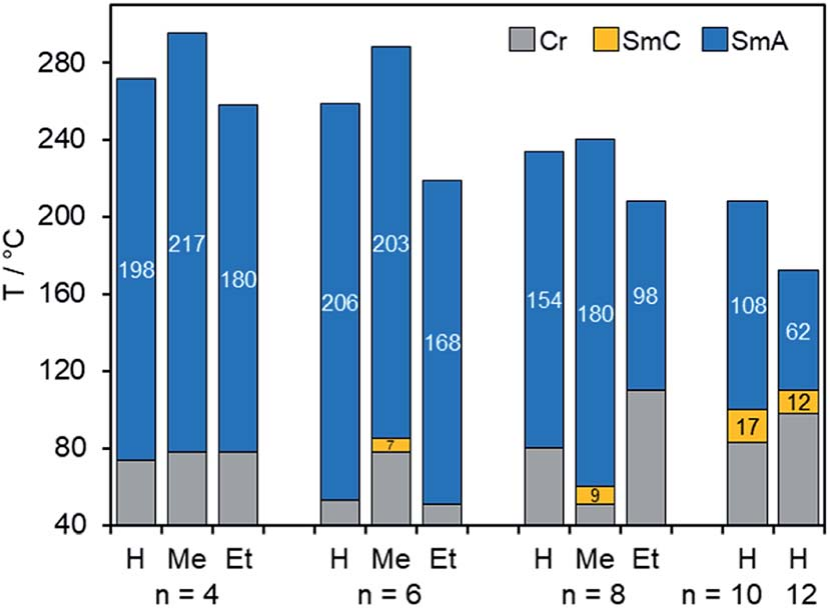
Comparison of mesophase stabilities of ILCs ImR(On,S12)Br with variable spacer lengths *n* upon heating. The values in the bar diagram correspond to the mesophase temperature ranges. Phase transitions were determined by DSC (1^st^ heating) and POM (SmA–SmC transition), for Im(On,S12)Br (*n* = 10, 12) 3^rd^ heating (see Tables S4 and S5, ESI†).

The phase behaviour of fluorenone imidazolium bromides Im(On,Sm)Br carrying thioether side chains was similar to that of the corresponding Im(On,Om)Br. As shown for Im(On,S12)Br with dodecylthio side chain, clearing temperatures decreased with increasing spacer lengths *n* (R = H, Fig. 4). Thioethers Im(On,S12)Br with short spacers *n* = 4, 6, 8 formed only SmA phases with broad phase widths of 154–206 K, whereas the homologous ILCs with longer spacers (*n* = 10, 12) displayed additional SmC phases. However, the phase stability was not increased by longer spacers. The replacement of oxygen with sulfur resulted in a significant destabilization of the SmC phase, and the phase ranges decreased to 12–17 K as compared to the corresponding ILCs Im(On,O12)Br (53 K).

The influence of substituent R at C-1 of the imidazolium head group on the mesophase stability of ILCs ImR(On,S12)Br is also shown in Fig. 4. Bromides ImR(On,S12)Br displayed broad SmA phases with phase widths ranging from 62–217 K. Within this series only the ILCs with H- or methyl-substituted imidazolium head group ImH(On,S12)Br and ImMe(On,S12)Br formed enantiotropic SmC phases with minimum spacer lengths of *n* = 10 (for R = H) and *n* = 6 (for R = Me). The corresponding ILCs ImEt(On,S12)Br showed only monotropic SmC phases under the POM upon cooling (Fig. 7 and S18–S20, ESI†). The clearing points were affected by substituent R. For example, for ImR(O4,S12)Br with a short C4 spacer the clearing point increased from 272 °C (R = H) to 295 °C (R = Me) and decreased to 258 °C (R = Et). It should be noted that ethyl-substituted imidazolium ILCs were still prone to thermal decomposition. These results are in good agreement with previous work by Swager,^106^ Butschies^92^ and Kapernaum.^17^

**Fig. 5:**
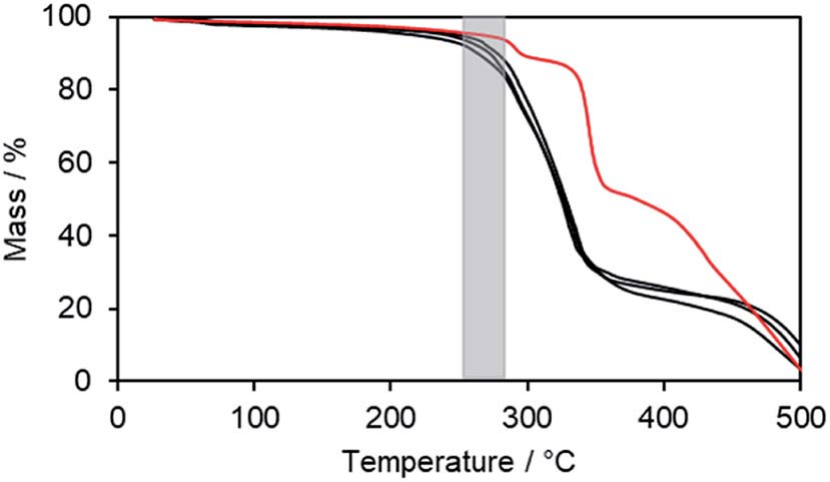
TGA thermograms of imidazolium bromides Im(On,O14)Br (*n* = 4,6), Im(O8,O16)Br (black lines) and triflate Im(O4,O14)OTf (red line). The grey zone illustrates the clearing temperature range.

To obtain further information on the thermal stability, ILCs Im(On,O14)Br (*n* = 4, 6) and Im(O8,O16)Br with clearing transitions >270 °C were submitted to thermogravimetric analysis (TGA) (Fig. 5). The ILCs were stable up to 200 °C (black curves). Upon further heating thermal decomposition was accompanied by loss of mass. The clearing points (grey zone) are located above the decomposition temperatures. As compared to the bromides, imidazolium triflate Im(O4,O14)OTf showed a slightly improved thermal stability (red curve), however, the clearing point (243 °C) is still within the decomposition range. Thus, TGA experiments confirmed the DSC results. Presumably the triflate anion is less nucleophilic than bromide and does not cause thermally induced dealkylations of the imidazolium unit. When comparing the DSC results of Im(On,Om)Br with Im(On,Om)OTf (Table S3, ESI†), it is remarkable that for imidazolium salts with large length differences between spacer *n* and side chain *m*, the clearing temperature decreased by ∼50 K, whereas the clearing temperature of imidazolium salts with similar lengths *n*, *m* decreased by >100 K.

**Fig. 6:**
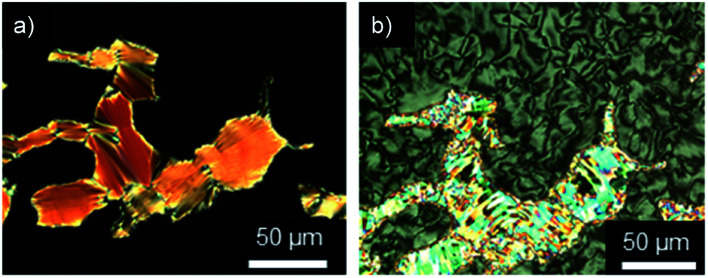
Textures of Im(O10,O12)Br as seen between crossed polarizers at (a) 132 °C and (b) 121 °C upon cooling from the isotropic liquid (cooling rate 5 K min^−1^, magnification ×200).

Investigations of fluorenone imidazolium bromides Im(On,Om)Br with alkoxy side chains under the POM showed large areas of homeotropic alignment. Birefringent areas could only be observed at phase boundaries and air bubbles (Fig. S13–S17, ESI†). ILCs Im(O4,Om)Br only showed fan textures (Fig. S13, ESI†), while imidazolium bromides forming SmA and SmC phases displayed both fan textures characteristic of SmA and at lower temperatures broken fan and Schlieren textures typical for SmC phases,^107^ as shown in Fig. 6 for Im(O10,O12)Br.

The POM studies provided initial hints for de Vries-like behaviour. The color change of the fan (SmA)/broken fan (SmC) texture of Im(O10,O12)Br during SmA–SmC phase transition from orange brown to pale blue indicated an increase in birefringence in the SmC phase and thus an increased orientational order, which is reported to be characteristic for de Vries-like materials.^30,54,55,65,103,108^

**Fig. 7:**
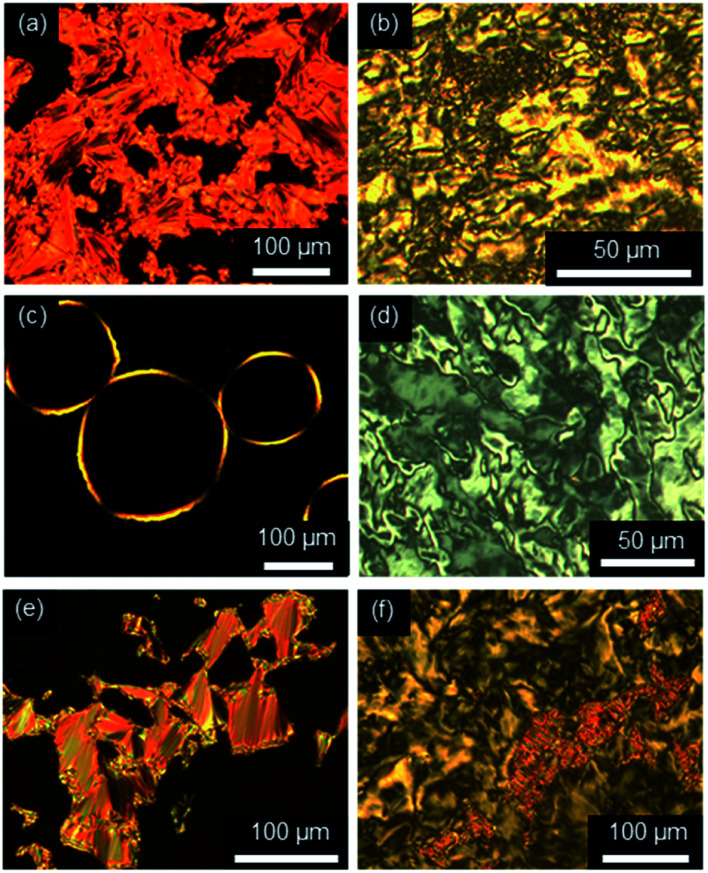
Textures as seen between crossed polarizers upon cooling from the isotropic liquid. (a and b) ImMe(O6,S12)Br at 180 °C and 52 °C, (c and d) ImEt(O8,S12)Br at 158 °C and 69 °C, and (e and f) ImH(O10,S12)Br at 128 °C and 94 °C, (cooling rate 5 K min^−1^, magnification ×100 (a, c and e), magnification ×200 (b and d)).

Fluorenone imidazolium bromides ImR(On,Sm)Br with thio-ether side chains formed fan and Bâtonnet textures or large homeotropic areas in the SmA phase and at lower temperatures Schlieren textures typical for SmC phases under the POM (Fig. 7). While for ILCs ImR(On,Sm)Br with R = H Schlieren textures occurred at a minimum spacer length *n* = 8 and side chain length *m* = 14, for the corresponding ILCs ImMe(On,Sm)Br and ImEt(On,Sm)Br Schlieren textures appeared already for spacer lengths *n* = 6.

**Fig. 8:**
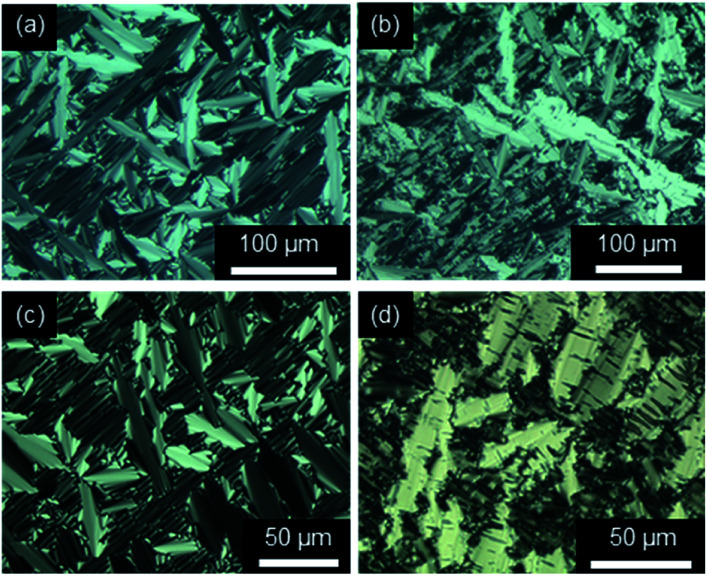
Textures of (a and b) Im(O10,O10)Br at 175 °C and 120 °C (magnification ×100) and (c and d) Im(O10,S10)Br at 180 °C and 86 °C (magnification ×200) under POM in a nylon-coated glass cell of 1.6 μm thickness (cooling rate 10 K min^−1^).

The strong tendency of the ILCs for homeotropic alignment in the SmA phase could be suppressed by using nylon-coated glass cells (1.6 μm). For example, upon cooling from the isotropic liquid ILCs Im(O10,Y10)Br displayed well-developed fan textures (Fig. 8a and c), which transformed into broken fan textures upon further cooling (Fig. 8b and d).

As expected from temperature-dependent SAXS experiments, layer distances *d* in the SmA phase of Im(On,Om)X and ImR(On,Sm)Br decreased with increasing temperature (Fig. S23 and S24, ESI†). The experimentally determined layer distances *d* were almost twice as large as compared to the calculated molecular lengths *L*_calc_ (molecular mechanics, force field: MMFF94s; software Avogadro^109^) of an all-trans fully extended conformation. The ratios *d*/*L*_calc_ were approximately 1.7–1.8 suggesting the presence of partially interdigitated smectic bilayers, that are also very prominent in de Vries-like materials (Tables S7 and S8, ESI†).

### Investigation of de Vries-like properties of the fluorenone ILCs

**Fig. 9:**
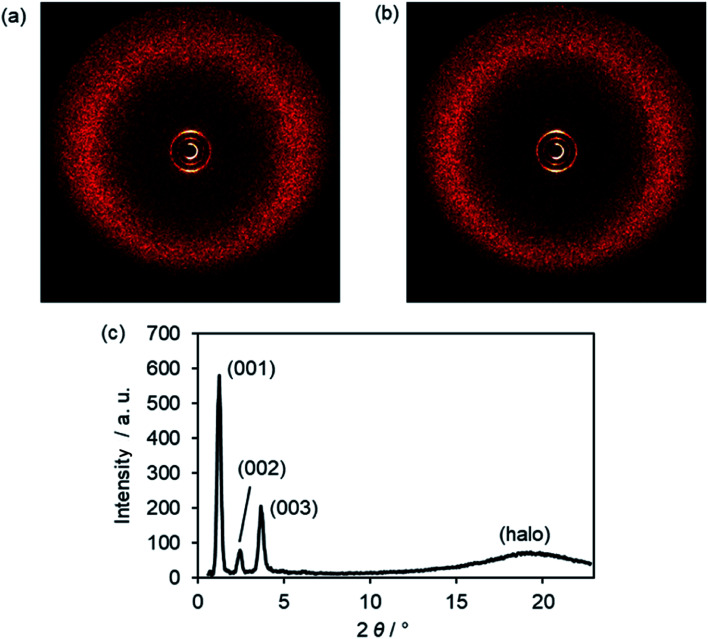
(a) WAXS diffraction image of Im(O12,O10)Br at 169 °C in the SmA phase, (b) WAXS diffraction image and (c) the corresponding scattering profile of Im(O12,O10)Br at 83 °C in the SmC phase.

In the WAXS pattern of the SmA phase of Im(O12,O10)Br at 169 °C three sharp reflexes in the small angle region and a diffuse maximum (halo) in the wide angle section are visible (Fig. 9a). The XRD pattern remained similar during SmA–SmC transition (Fig. 9b), because ILCs ImR(On,Ym)X did not give oriented samples.^17,18^

High order layer reflections up to 4^th^ order (004) could be observed for ILCs ImR(On,Ym)X (Table S8, ESI†), suggesting that the smectic phases of the ILCs possess a high degree of translational order presumably due to the strong nano-segregating effect of the ionic head group.^21,30,110^

One parameter for de Vries-like behaviour is the maximum layer contraction at the SmA–SmC phase transition. Therefore, the smectic layer spacing *d* of ILCs ImR(On,Ym)Br as a function of temperature was measured by small-angle X-ray scattering (SAXS). The layer distances *d* were calculated from the (001) reflex at different temperatures, where *d*_AC_ denotes the layer distance at the SmA–SmC transition temperature *T*_AC_. At the temperature where the largest layer shrinkage occurred, the maximum layer contraction *S*_max_ was determined.

**Fig. 10:**
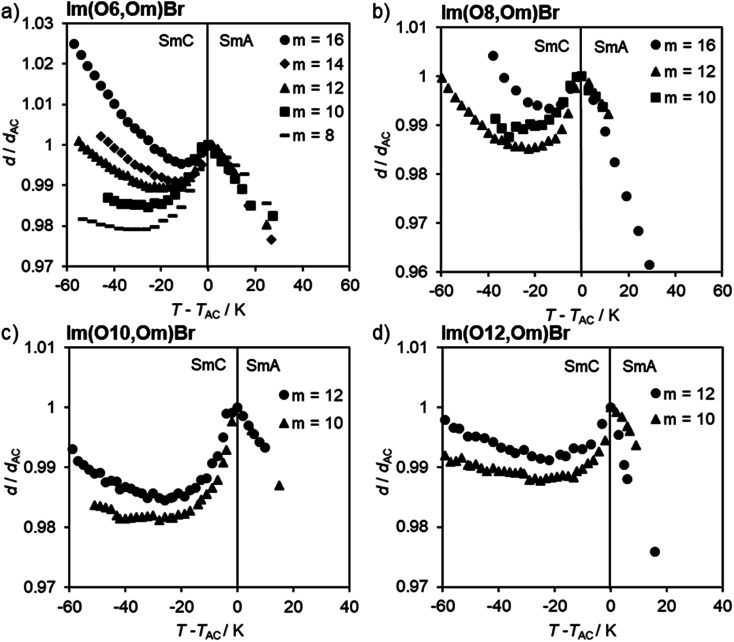
Layer spacing *d*/*d*_AC_*vs.* reduced temperature *T* − *T*_AC_ profiles of Im(On,Om)Br.

The profiles of normalized layer spacings *d*/*d*_AC_*vs.* reduced temperature *T* − *T*_AC_ for ILCs Im(On,Om)Br with an alkoxy side chain are summarized in Fig. 10 and Table 1. Estimated standard deviations were <5%. The temperature-dependent *d*/*d*_AC_ curves are similar in this series. Upon cooling an expansion in the SmA phase until the transition temperature was observed. At transition into the SmC phase, the layer spacing initially decreased and then increased upon further cooling. This behaviour is typical for de Vries-like materials, while in conventional liquid crystals the layer thickness decreases continuously in the SmC phase resulting in larger layer contractions.^50^ As seen in Fig. 10a, the maximum layer contraction *S*_max_ decreases with the length *m* of the alkoxy side chain, from 2.1% for Im(O6,O8)Br to 0.5% for Im(O6,O16)Br (Table 1).

**Table 1:** Maximum layer contraction *S*_max_, calculated from the layer spacing *d*_AC_ at the SmA–SmC transition and the layer spacing *d*_C_ in the SmC phase of ILCs ImR(On,Ym)Br at the reduced temperature *T* − *T*_AC_

Compound	*T* − *T*_AC_/K	*d*_AC_/Å	*d*_C_/Å	*S*_max_/%
Im(O6,O8)Br	−30	57.7	56.5	2.1
Im(O6,O10)Br	−26	63.8	62.8	1.5
Im(O6,O12)Br	−19	63.7	63.1	1.1
Im(O6,O14)Br	−14	69.9	69.3	0.9
Im(O6,O16)Br	−11	70.3	70.0	0.5
Im(O8,O10)Br	−31	66.0	65.2	1.4
Im(O8,O12)Br	−23	66.3	65.3	1.5
Im(O8,O16)Br	−14	74.1	73.6	0.7
Im(O10,O10)Br	−28	64.7	63.4	1.9
Im(O10,O12)Br	−26	68.0	67.0	1.5
Im(O12,O10)Br	−25	66.7	65.9	1.2
Im(O12,O12)Br	−22	72.0	71.4	0.9
Im(O8,S14)Br	−10	72.9	72.6	0.4
Im(O10,S10)Br	−13	68.9	68.1	1.1
Im(O10,S12)Br	−14	72.1	71.4	0.9
Im(O10,S14)Br	−6	72.8	72.4	0.5
Im(O12,S10)Br	−25	71.1	70.4	1.0
Im(O12,S12)Br	−6	74.5	73.8	0.9
Im(O12,S14)Br	−10	76.8	76.5	0.4
ImMe(O6,S12)Br	−17	69.4	68.8	0.9
ImMe(O6,S14)Br	−11	71.1	70.6	0.7
ImMe(O8,S12)Br	−20	70.7	70.0	1.0
ImMe(O8,S14)Br	−19	71.9	71.2	1.1
ImEt(O6,S12)Br	−14	66.5	65.7	1.1
ImEt(O8,S10)Br	−11	67.3	66.5	1.2
ImEt(O8,S12)Br	−17	70.7	69.8	1.4

A similar trend was observed for Im(O10,Om)Br and Im(O12,Om)Br (Fig. 10c and d). However, in the series of Im(O8,Om)Br the trend was less clearcut. ILC Im(O8,O16)Br exhibited a maximum layer contraction *S*_max_ = 0.7%, but Im(O8,Om)Br with *m* = 10, 12 gave similar *S*_max_ values of 1.5% and 1.4% (Fig. 10b and Table 1).

**Fig. 11:**
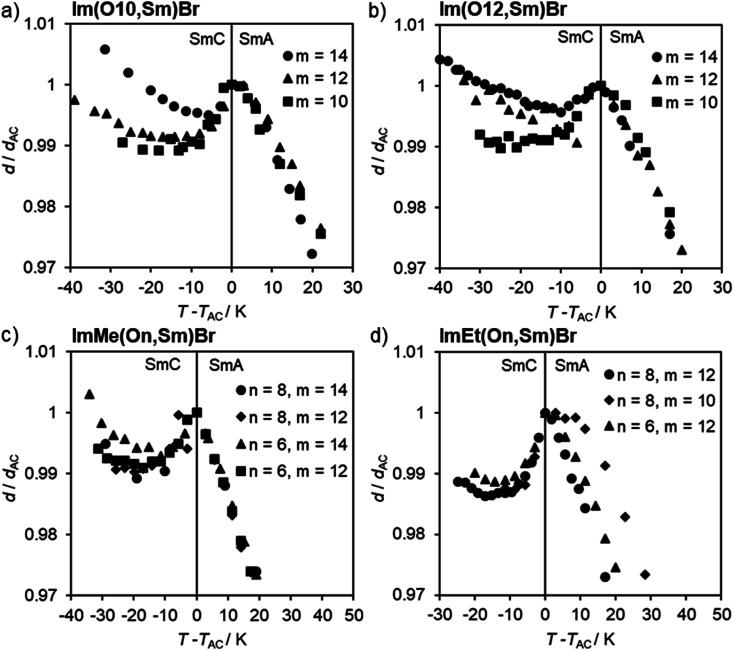
Layer spacing *d*/*d*_AC_*vs.* reduced temperature *T* − *T*_AC_ profiles of ImR(On,Sm)Br with R = H (a and b), Me (c), Et (d).

According to the *d*/*d*_AC_*vs. T* − *T*_AC_ profiles, ILCs Im(On,Sm)Br with thioether side behaved similarly, *i.e.* the maximum layer contractions decrease with increasing side chain length *m* (Fig. 11a, b and Table 1). In contrast, the maximum layer contraction of the ILCs ImMe(On,Sm)Br and ImEt(On,Sm)Br with methyl or ethyl substituents at the imidazolium unit (R = Me, Et) did not show any clear trend (Fig. 11c, d and Table 1).

The alkyl chain lengths effect on the layer contraction might be rationalized by the interdigitation with longer chains showing a higher degree of interdigitation as compared to shorter chains in agreement with recent studies by Wöhrle on SmA phases of MIDA boronates.^111^ The determined *d*/*L*_calc_ ratios, however, did not show a clear trend (Tables S7 and S8, ESI†). For example, the Im(O6,Om)Br series revealed *d*_AC_/*L*_calc_ ratios at the SmA–SmC phase transition of 1.79 (*m* = 8), 1.84 (*m* = 10), 1.71 (*m* = 12), 1.76 (*m* = 14), and 1.67 (*m* = 16). The pronounced color change of the textures observed for ImR(On,Ym)Br by POM during SmA–SmC transition indicated an increase in birefringence, which correlates with an increase in orientational order parameter *S*_2_.^45–47,53^ Therefore, we assume that the orientational order increased in the SmC phase and compensation for a large portion of the maximum layer contraction. However, due to unavailability of oriented samples of fluorenone ILCs ImR(On,Ym)Br for XRD studies we failed to clarify which effect compensates layer shrinkage and is responsible for the de Vries-like properties.

**Fig. 12:**
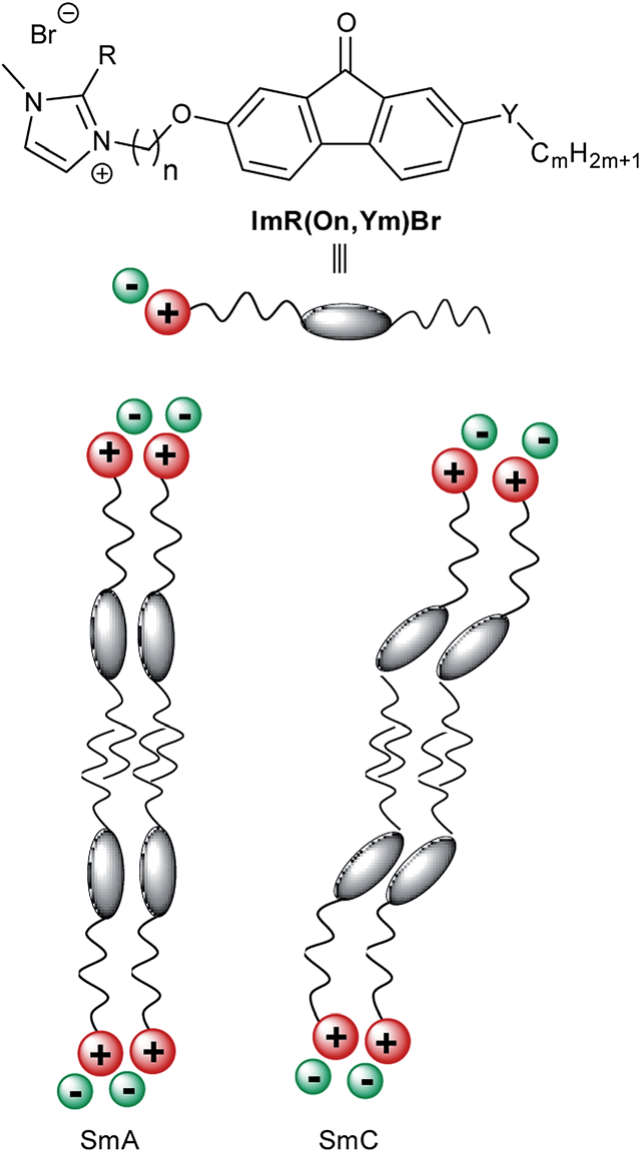
Schematic illustration of the SmA and SmC phase of ILCs ImR(On,Ym)Br.

An alternative method to rationalize the beneficial influence of the alkyl chain lengths on the layer contraction is the so-called zig–zag model, originally proposed by Bartolino, Doucet and Durand,^112^ and later confirmed by Böffel^113^ and Clark.^114^ In the zig–zag model the rigid cores are tilted to larger extent than the aliphatic chains in the SmC phase, *i.e.* the tilt of the layers is mainly caused by the tilted orientation of the rigid aromatic cores rather than the alkyl chains (Fig. 12).

The zig–zag model has also been implemented in the Boulder model by Walba and Clark to explain the polarization of ferroelectric liquid crystals.^115–117^ According to this model an increase of the non-tilted alkyl chains should lead to a smaller layer contraction at the SmA–SmC phase transition.

**Fig. 13:**
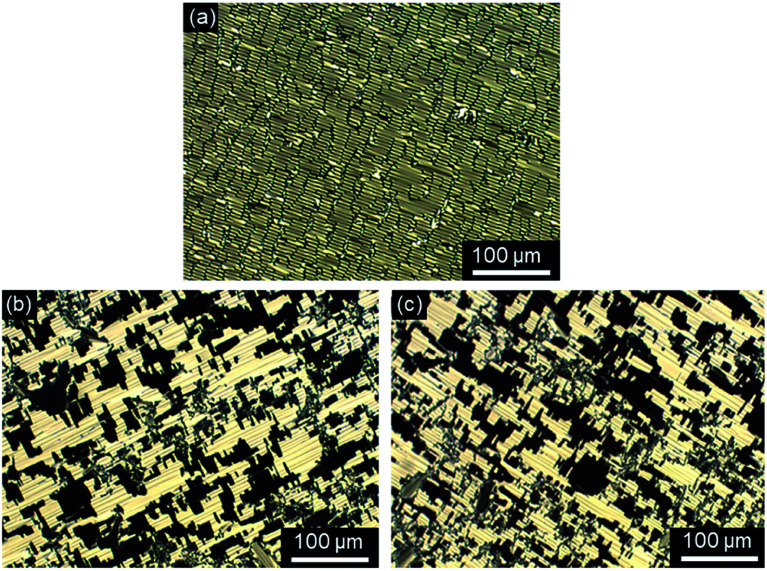
Textures of Im(O12,O10)Br observed under the POM in a glass cell with rubbed nylon alignment layer of 1.6 μm thickness upon slow cooling from the isotropic liquid to achieve planar alignment. (a) At 160 °C in the SmA phase, and (b and c) at 135 °C in the SmC phase (cooling rate 0.2 K min^−1^, magnification ×200).

The de Vries-like behaviour can be quantified by the reduction factor *R*, which considers both maximum layer contraction and optical tilt angle *θ*_opt_ in the SmC phase measured as a function of temperature by POM.^18,53,55,65,103^ As the samples were filled in ITO glass cells with rubbed nylon alignment layer (1.6 μm thickness) by isotropic heating, only those ILCs could be investigated, which were thermally stable above the isotropization temperature based on their reproducible DSC curves. As an example, planar textures of Im(O12,O10)Br are shown in Fig. 13. In the SmA phase uniform domains are visible due to a parallel orientation of the director *n* with respect to the layer normal *k* (Fig. 13a), while in the SmC phase two different domain structures with different brightness are visible (Fig. 13b and c).

**Fig. 14:**
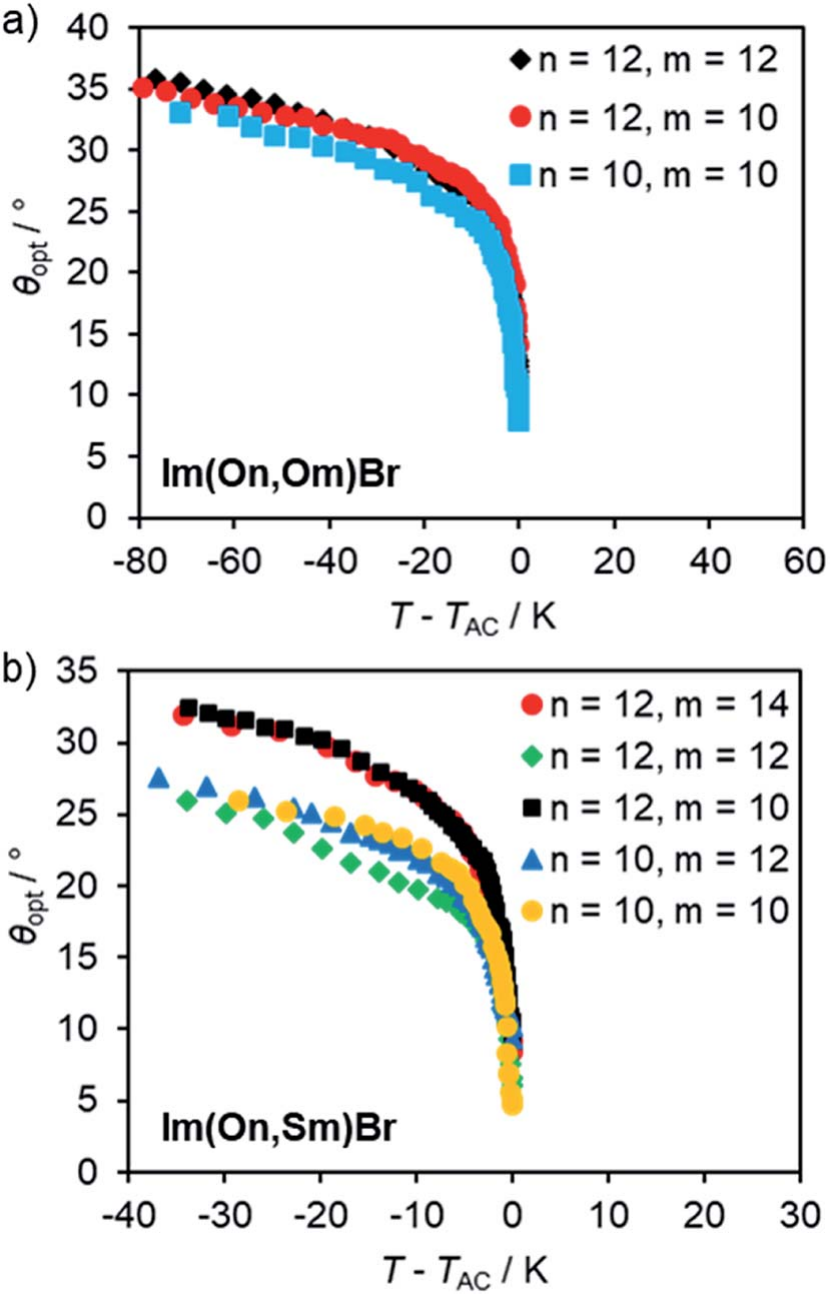
Optical tilt angle *θ*_opt_*vs.* reduced temperature *T* − *T*_AC_ for (a) Im(On,Om)Br and (b) Im(On,Sm)Br. For *θ*_opt_(*T*) profiles fitted to the power law according to ref. 103 see Fig. S25, ESI.†

Optical tilt angles *θ*_opt_ were measured by POM as function of reduced temperature *T* − *T*_AC_ in the absence of an electrical field upon cooling. In the SmA phase the director *n* is parallel to the layer normal *k* and thus *θ*_opt_ = 0 (Fig. 14).

At *T* − *T*_AC_ = 0, the optical tilt angle *θ*_opt_ of the SmC phase increased significantly and then steadily increased upon further cooling. The abrupt increase of *θ*_opt_ indicates a 1^st^ order SmA–SmC transition. This is in accordance with the DSC measurements, where for several compounds small peaks at the transitions from SmA to SmC were observed. In the series of ILCs with alkoxy side chain Im(On,Om)Br*θ*_opt_ increased up to 36° (Fig. 14a). The optical tilt angle profile of ILCs with thioether side chain Im(On,Sm)Br reveals slightly lower optical tilt angles (28–32°) (Fig. 14b). No general trend regarding spacer lengths *n* or alkyl chain lengths *m* could be detected.

From the maximum layer contraction and the optical tilt angle *θ*_opt_ the reduction factor *R* was calculated according to equation *R* = *δ*(*T*)/*θ*_opt_(*T*) = cos^−1^[*d*_C_(*T*)/*d*_AC_]/*θ*_opt_(*T*) for a given temperature.^53,118^ The *R* values, maximum layer contraction *S*_max_ and optical tilt angle *θ*_opt_ at the reduced temperature *T* − *T*_AC_ are summarized in Table 2.

**Table 2:** Maximum layer contraction *S*_max_, reduction factor *R* and optical tilt angle *θ*_opt_ of ILCs Im(On,Ym)Br at the temperature of the maximum layer contraction *T* − *T*_AC_

Compound	*T* −;*T*_AC_/K	*d*_AC_/Å	*d*_C_/Å	*S*_max_/%	*θ*_opt_/°	*R*
Im(O10,O10)Br	−28	64.7	63.4	1.9	28	0.40
Im(O12,O10)Br	−25	66.7	65.9	1.2	30	0.30
Im(O12,O12)Br	−22	72.0	71.4	0.9	29	0.26
Im(O10,S10)Br	−13	68.9	68.1	1.1	24	0.35
Im(O10,S12)Br	−14	72.1	71.4	0.9	23	0.33
Im(O12,S10)Br	−25	71.1	70.4	1.0	31	0.26
Im(O12,S12)Br	−6	74.5	73.8	0.9	19	0.41
Im(O12,S14)Br	−10	76.8	76.5	0.4	27	0.20

The ILCs have *R* values ranging from 0.20 to 0.41 indicating a high de Vries-like behaviour. Thus, our design concept not only induced the rare SmC phase but also enabled de Vries behaviour with layer contractions *S*_max_ and *R* values, which are comparable to those of known neutral liquid crystalline de Vries materials^50,53,65^ and further extends the scaffolds of ILCs previously developed by Kapernaum.^17,18^ Moreover, it confirms that fluorenones are beneficial for de Vries ILCs. In agreement with the procedure reported by Lemieux^103^ the *θ*_opt_(*T*) profiles were fitted to the power law,^103^ which provided the order parameter *β* related to the SmA–SmC transition (see also ref. 24 in Lemieux^103^). The ILCs possessed *β* values of 0.19–0.24, *i.e.* indicating weak 1^st^ order transitions or tricritical point transitions (in case of *β* = 0.25) in agreement with Lemieux' observations (for details see Fig. S25, ESI†).

### Calculation of the electron density

Based on layer reflections from SAXS experiments the electron density distribution in the smectic phase was obtained. The electron density profile *ρ*(*z*) along the director *n* was calculated by using eqn (1).^119^1
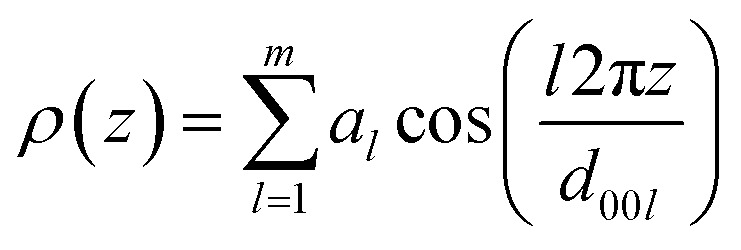


From the intensity of the Bragg reflections (00*l*) and subsequent correction *via* the Lorentz factor^120^ the square values |*a*_*l*_|^2^ were determined, while the signs of the *a*_*l*_ coefficients remained indefinite.^121^ Therefore, all possible combinations of signs of the different *a*_*l*_ coefficients must be generated and submitted to a plausibility check. The imidazolium bromides Im(On,Ym)Br showed three layer reflections and therefore three coefficients *a*_1_, *a*_2_ and *a*_3_ could be determined. The results are summarized in Table 3.

**Table 3:** Layer distance *d* of imidazolium bromides Im(On,Ym)Br and coefficients *a*_1_, *a*_2_, *a*_3_

	*T*/°C	*d*_001_/Å	*d*_002_/Å	*d*_003_/Å	*a*_1_	*a*_2_	*a*_3_
Im(O12,O10)Br	167	66.7	33.3	22.1	1	0.31	0.52
Im(O12,S10)Br	160	67.7	33.8	22.4	1	0.32	0.60
Im(O12,O12)Br	176	71.1	35.6	23.5	1	0.29	0.41
Im(O12,S12)Br	164	69.9	34.8	23.1	1	0.38	0.55

**Fig. 15:**
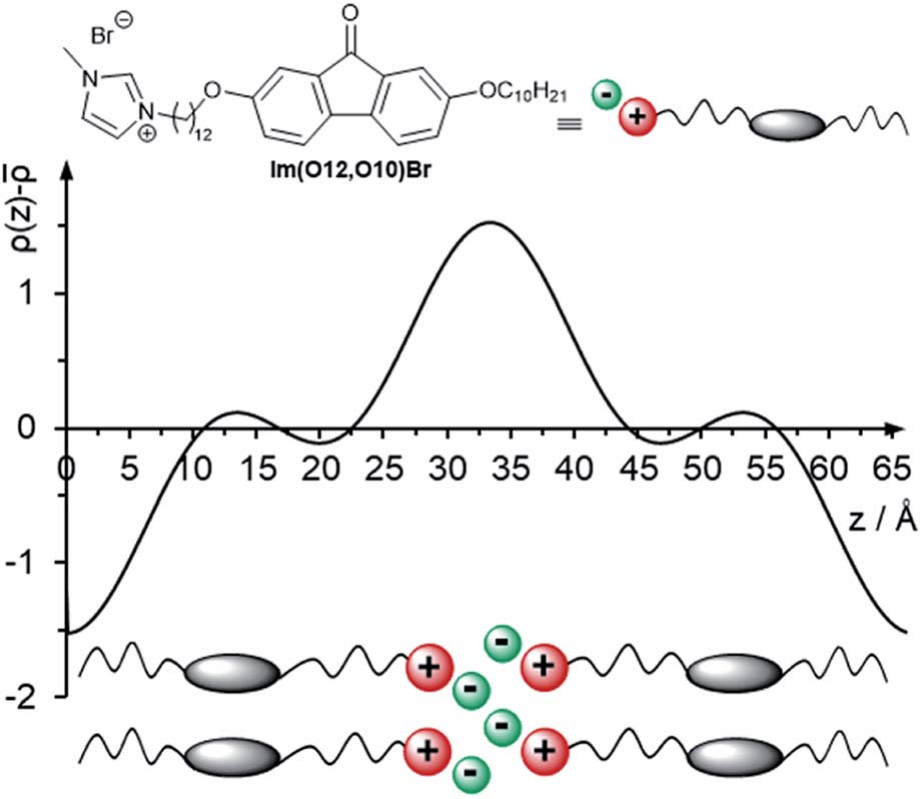
Electron density profile of Im(O12,O10)Br in the SmA phase at 167 °C indicating a bilayer structure and its suggested packing model.

The electron density profile and the packing model of Im(O12,O10)Br are shown in Fig. 15. A large maximum of the electron density *ρ*(*z*) is visible in the region of the ionic head groups, forming a charged sublayer. In the region of the aliphatic spacer a local minimum of the electron density *ρ*(*z*) is visible and a local maximum due to the aromatic fluorenone core. Along the terminal alkoxy side chains, the electron density *ρ*(*z*) shows a global minimum. For the other fluorenones in Table 3 analogous density profiles were obtained (Fig. S26, ESI†).

### Absorption and emission properties of fluorenone precursors and ILCs

**Fig. 16:**
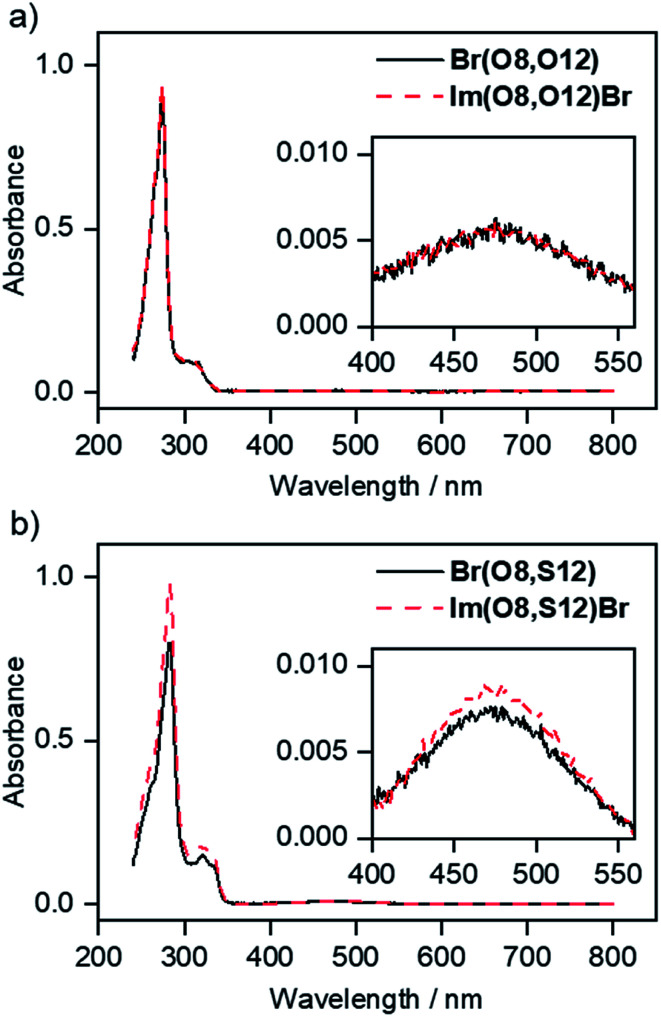
Absorbance spectra (in CHCl_3_) of (a) precursor fluorenone Br(O8,O12) and ILC Im(O8,O12)Br with alkoxy side chains and (b) precursor fluorenone Br(O8,S12) and ILC Im(O8,S12)Br with thioether side chains. For details of the fluorescence spectra in solution see Fig. S28 and S29 and the corresponding text in the ESI.†

UV/vis data of imidazolium salts Im(On,Ym)X and their precursors are shown in Fig. 16. All fluorenone derivatives with an alkoxy side chain displayed absorption maxima at *λ*_max_ = 272–274 nm with a shoulder at lower wavelengths and two smaller bands at 302 nm and 314 nm, respectively (Fig. 16a).^122^ The intense bands at 272–274 nm were assigned to the π–π* transition of the fluorenone core.^123,124^ The UV/vis spectra were neither affected by different donor groups at the fluorenone core, *i.e.* methoxy, alkoxy or hydroxy nor different anions (bromide *vs.* triflate). However, the replacement of alkoxy by thioether side chains resulted in a bathochromic shift of the absorption maxima to *λ*_max_ = 282–283 nm (Fig. 16b).

This effect has been used for organic solar cells and field effect transistors to decrease the HOMO–LUMO gap and to improve the charge carrier mobility.^125^ In contrast, the strongly electron-withdrawing fluoro substituent in 10f shifted the absorption maximum hypsochromically to *λ*_max_ = 267 nm (Fig. S27†). All studied fluorenones were orange-red amorphous solids due to a low absorption in the visible region. A weak band around 400 nm was assigned to symmetry-forbidden n–π* transition of the carbonyl group (Fig. 16, inset).^123,124^

**Fig. 17:**
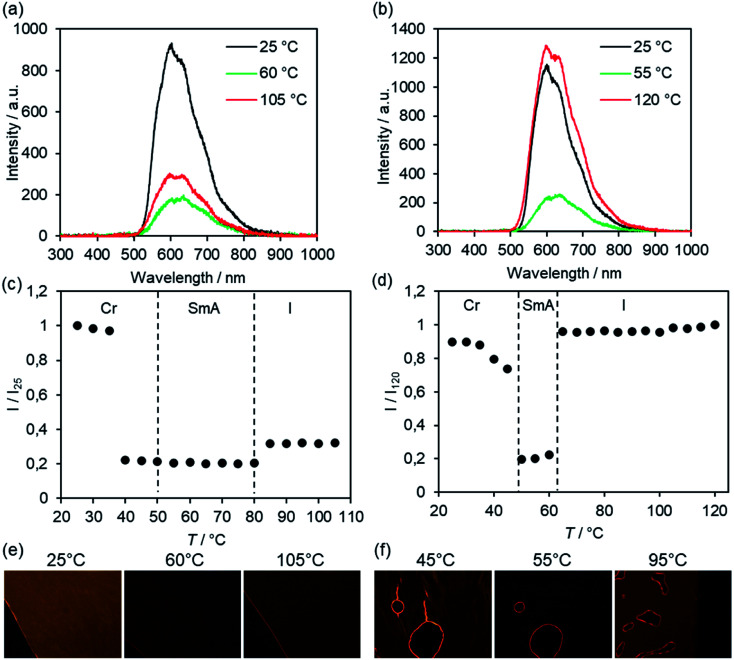
Emission spectra of (a) Br(O6,O16) and (b) Br(O8,S12) at different temperatures (*λ*_exc_ = 350–380 nm). Temperature-dependent emission intensity of (b) Br(O6,O16) and (d) Br(O8,S12) upon cooling from the isotropic liquid (cooling rate 5 K min^−1^). Transition temperatures (dashed lines) were determined from the 3^rd^ cooling scan by DSC (Tables S1 and S2, ESI†). POM pictures of (e) Br(O6,O16) and (f) Br(O8,S12) under irradiation with UV light at the given temperatures, irradiation time 8 s (e) and 4 s (f). POM pictures were taken without analyzer in order to detect sufficient emission intensity.

For potential applications bulk emission properties are particularly interesting. Therefore, selected samples were studied by solid state fluorescence spectroscopy utilizing a polarizing optical microscope equipped with UV light source (*λ*_exc_ = 350–380 nm) and photodetector. The method is limited to thermally stable compounds, because the samples required isotropization and thermal annealing to obtain uniform thin films for solid state emission spectra. The solid state emission spectra of Br(O6,O16), exemplified in Fig. 17a, displayed at 25 °C a strong band at *λ*_em_ = 604 nm with two shoulders at longer wavelengths caused by the excimer emission due to fluorenone–fluorenone interactions.^122^ At higher temperatures the intensity of the shoulder at longer wavelengths (635 nm) resulting from vibronic coupling increased.

The emission intensity was measured as a function of temperature upon cooling from the isotropic liquid (Fig. 17c). In the isotropic liquid the emission intensity was only 30% of the intensity at 25 °C (*I*_25_). The *I*_80_/*I*_25_ value decreased to 0.2 (20%) in the SmA phase and remained constant throughout the whole temperature range of the phase. Upon transition into the crystalline phase at 35 °C a strong increase of the emission was visible. It should be noted, that the SmA–Cr transition was detected at 51 °C by DSC (dashed line in Fig. 17c). The different emission intensities could also be observed *via* the POM pictures under irradiation with UV light (Fig. 17e).

A different behaviour of the bulk emission was observed for fluorenone Br(O8,S12) with thioether side chain (Fig. 17b, d and f). A strong emission at *λ*_em_ = 600 nm was observed in the isotropic liquid. Upon cooling and at the I–SmA transition the intensity decreased only slightly, but at 60 °C in the SmA phase, the intensity decreased steeply to 20% of the intensity of the isotropic phase. At the SmA–Cr transition again a steep increase of the emission was detected, with the intensity being slightly below the emission intensity of the isotropic phase. In other words, the strongest fluorescence was observed at 120 °C. This result is remarkable, because the strongest emission would be expected at low temperature in the highly ordered crystalline phase rather than in the disordered isotropic phase. Presumably, the observed band at 600 nm corresponds to the excimer emission and thus, comparison of ether and thioether derivatives suggests that the interaction between fluorenones in the thioether derivative is more pronounced in the isotropic and crystalline phase as compared to the SmA phase.^126^

The bulk emission behaviour of fluorenone imidazolium triflate Im(O6,O8)OTf resembled that of Br(O6,O16). Upon heating of the crystalline phase the emission intensity decreased slightly, but decreased steeply at 80 °C at the Cr–SmA transition to a 10 times lower intensity than that in the Cr phase and remained constant upon further heating and did not increase upon isotropic clearing (Fig. S30, ESI†).

## Conclusion

We developed a synthetic strategy to a series of ILCs ImR(On,Ym)X consisting of a SmC-promoting central fluorenone core with one alkoxy or thioether side chain and a flexible spacer connected to a SmA-promoting imidazolium head group. Precursor fluorenone bromides Br(On,Ym) formed only SmA mesophases, while the fluorenone ILCs Im(On,Om)Br carrying an alkoxy side chain indeed displayed both SmA and SmC mesophases. Thereby spacer lengths *n* > 10 increased the stability of the SmC phase up to phase widths of 53 K. Attempts to overcome the high clearing points of ILCs Im(On,Om)Br, being close to the thermal decomposition temperature *via* exchange of the bromides against triflate counterions resulted indeed in decreased clearing temperatures. However, the desired effect was accompanied by the loss of the SmC phase and thus triflate was not further considered in these ILCs. ILCs ImR(On,Sm)Br with thioether side chain behaved similarly as compared to the corresponding ether derivatives Im(On,Om)Br. In this series enantiotropic SmC mesophases were observed for the H- and Me-substituted imidazolium group with minimum spacer lengths *n* = 10 (R = H) and *n* = 6 (R = Me), while the ethyl substituent (R = Et) caused monotropic SmC phases upon cooling. Fluorenone ILCs Im(On,Sm)Br carrying thioethers displayed broader SmC phases as compared to the corresponding ether derivatives Im(On,Om)Br, while the opposite trend was observed for the temperature range of the SmA phase, suggesting that S–S interactions are beneficial for SmC phases in ILCs. Alternatively, the larger sulfur in the thioether side chains might be better accommodated in a tilted than a non-tilted orientation.

POM observations of a color change of the textures during the SmA–SmC phase transition indicated an increase in the birefringence of the SmC phase which is correlated with an increased orientational order as expected for de Vries-like materials.^30,108^ In XRD measurements higher order reflections were visible suggesting a high degree of translational order in the smectic phase which may originate from strong nano-segregation of the ionic imidazolium head group.^30^ From *d*/*d*_AC_*vs. T* − *T*_AC_ profiles maximum layer contraction values *S*_max_ ranging from 0.4 to 2.1% were obtained. The optical tilt angles *θ*_opt_ measured by POM, and finally the calculation of the reduction factor *R* based on layer spacings *d*/*d*_AC_ and *θ*_opt_ values gave further evidence for de Vries-like behaviour. The *R* values of ILCs ImR(On,Ym)Br ranged from 0.2 to 0.41 and are comparable to those of known neutral liquid crystalline compounds.^50,53,65^ The most promising de Vries ILC was thioether derivative Im(O12,S14)Br (*S*_max_ = 0.4%, *R* = 0.20).

Emission spectra of selected derivatives revealed “off–on” effects because the emission intensity strongly depended on the phase type (crystalline, liquid crystalline, isotropic). For bromides Br(On,Om) with an alkoxy side chain, the highest intensity observed in the crystalline state steeply decreased at the Cr–SmA transition and increased in the isotropic liquid. Bromide Br(O8,S12) with thioether side chain showed a reverse behaviour, *i.e.* a steep decrease of highest intensity in the isotropic liquid upon SmA transition followed by an increase at the SmA–Cr phase transition. The emission intensity profile of ILC Im(O6,O8)OTf resembled that of Br(On,Om) with the strongest intensity in the crystalline state. After abrupt reduction at the Cr–SmA transition, however, the intensity remained nearly constant also in the isotropic liquid.

In conclusion, we have demonstrated that the combination of charged imidazolium head groups with calamitic fluorenones carrying a lateral polar group and a flexible tether generates ILCs with remarkably low layer shrinkage during SmA–SmC transition. Moreover, fluorenone ILCs and their precursor bromides possess a strong solid state luminescence, which is switched off in the SmA phase and in case of Br(O8,S12) is recovered in the isotropic phase. The emission observed in the solid state is the excimer emission corresponding to the interaction between fluorenone moieties, indicating aggregation-induced emission (AIE) behaviour,^127,128^ which is much stronger in the isotropic and crystalline phase than in the SmA phase for Br(O8,S12). The phenomenon is less pronounced for the fully oxygenated compound. Thus, our approach has provided access to novel de Vries as well as thermoluminescent materials.

## Conflicts of interest

There are no conflicts to declare.

## Supplementary Material

RA-010-D0RA04650G-s001

## References

[cit1] Goossens K., Lava K., Bielawski C. W., Binnemans K. (2016). Chem. Rev..

[cit2] Mansueto M. and Laschat S., in Handbook of Liquid Crystals, ed. J. W. Goodby, P. J. Collings, T. Kato, C. Tschierske, H. Gleeson and P. Raynes, Wiley-VCH, Weinheim, 2nd edn, 2014, vol. 6, pp. 231–280

[cit3] Chen S., Eichhorn S. H. (2012). Isr. J. Chem..

[cit4] Axenov K. V., Laschat S. (2011). Materials.

[cit5] Douce L., Suisse J.-M., Guillon D., Taubert A. (2011). Liq. Cryst..

[cit6] Binnemans K. (2005). Chem. Rev..

[cit7] Causin V. and Saielli G., in Green Solvents II: Properties and Applications of Ionic Liquids, ed. A. Mohammad and D. Imamuddin, Springer, Dordrecht, 2012, pp. 79–118

[cit8] Pal S. K. and Kumar S., in Biosensors Nanotechnology, ed. A. Tiwani and A. P. F. Turner, Scrivener Publishing, Berkeley, MA 2014, pp. 267–314

[cit9] Högberg D., Soberats B., Yatagai R., Uchida S., Yoshio M., Kloo L., Segawa H., Kato T. (2016). Chem. Mater..

[cit10] Atasiei R., Raicopol M., Andronescu C., Hanganu A., Alexe-Ionescu A. L., Barbero G. (2018). J. Mol. Liq..

[cit11] Yuan F., Chi S., Dong S., Zou X., Lv S., Bao L., Wang J. (2019). Electrochim. Acta.

[cit12] Stappert K., Mudring A.-V. (2015). RSC Adv..

[cit13] Kohmoto S., Hara Y., Kishikawa K. (2010). Tetrahedron Lett..

[cit14] Goossens K., Lava K., Nockemann P., Van Hecke T., Van Meervelt L., Pattison P., Binnemans K., Cardinaels T. (2009). Langmuir.

[cit15] Trbojevic N., Haenle J. C., Wöhrle T., Kirres J., Laschat S. (2016). Liq. Cryst..

[cit16] Starkulla G. F., Klenk S., Butschies M., Tussetschläger S., Laschat S. (2012). J. Mater. Chem..

[cit17] Kapernaum N., Wuckert E., Frey W., Marino S., Wahl M., Giesselmann F., Laschat S. (2018). J. Phys. Org. Chem..

[cit18] Kapernaum N., Müller C., Moors S., Schlick M. C., Wuckert E., Laschat S., Giesselmann F. (2016). ChemPhysChem.

[cit19] Sanchez-Castillo A., Osipov M. A., Jagiella S., Nguyen Z. H., Kašpar M., Hamplova V., Maclennan J., Giesselmann F. (2012). Phys. Rev. E: Stat., Nonlinear, Soft Matter Phys..

[cit20] Haase W., Fan Z. X., Müller H. J. (1988). J. Chem. Phys..

[cit21] Leadbetter A. J., Norris E. K. (1979). Mol. Phys..

[cit22] McMillan W. L. (1972). Phys. Rev. A: At., Mol., Opt. Phys..

[cit23] Kapernaum N., Giesselmann F. (2008). Phys. Rev. E: Stat., Nonlinear, Soft Matter Phys..

[cit24] Watanabe J., Hayashi M. (1989). Macromolecules.

[cit25] Gramsbergen E. F., De Jeu W. H. (1989). Liq. Cryst..

[cit26] Takanishi Y., Ikeda A., Takezoe H., Fukuda A. (1995). Phys. Rev. E: Stat., Nonlinear, Soft Matter Phys..

[cit27] Nonnenmacher D., Jagiella S., Song Q. X., Lemieux R. P., Giesselmann F. (2013). ChemPhysChem.

[cit28] Gorkunov M. V., Osipov M. A., Kapernaum N., Nonnenmacher D., Giesselmann F. (2011). Phys. Rev. E: Stat., Nonlinear, Soft Matter Phys..

[cit29] Lagerwall S. T., Rudquist P., Giesselmann F. (2009). Mol. Cryst. Liq. Cryst..

[cit30] Lagerwall J. P. F., Giesselmann F. (2006). ChemPhysChem.

[cit31] de Vries A. (1979). J. Chem. Phys..

[cit32] de Vries A. (1979). Mol. Cryst. Liq. Cryst..

[cit33] de Vries A., Ekachai A., Spielberg N. (1979). Mol. Cryst. Liq. Cryst..

[cit34] Swaminathan V., Panov V. P., Panov A., Rodriguez-Lojo D., Stevenson P. J., Gorecka E., Vij J. K. (2020). J. Mater. Chem. C.

[cit35] Yadav N., Swaminathan V., Panov V. P., Dhar R., Vij J. K. (2019). Phys. Rev. E: Stat., Nonlinear, Soft Matter Phys..

[cit36] Yamada Y., Sano W., Fukuda A. (2019). Mol. Cryst. Liq. Cryst..

[cit37] Green A. A. S., Tuchband M. R., Shao R., Shen Y., Visvanathan R., Duncan A. E., Lehmann A., Tschierske C., Carlson E. D., Guzman E., Kolber M., Walba D. M., Park C. S., Glaser M. A., Maclennan J. E., Clark N. A. (2019). Phys. Rev. Lett..

[cit38] Goswami D., Mandal P. K., Gutowski O., Sarma A. (2019). Liq. Cryst..

[cit39] Sreenilayam S. P., Rodriguez-Lojo D., Agra-Kooijman D. M., Vij J. K., Panov V. P., Panov A., Fisch M. R., Kumar S., Stevenson P. J. (2018). Phys. Rev. Mater..

[cit40] Zoghaib W. M., Carboni C., Kashoub F. A., Al-Rushidi J. S., Al-Jabri B. Y., Al-Alawi A. S., Al-Mendhry H. F. (2018). Mol. Cryst. Liq. Cryst..

[cit41] Swaminathan V., Panov V. P., Kocot A., Vij J. K. (2019). J. Chem. Phys..

[cit42] Swaminathan V., Panov V. P., Sreenilayam S. P., Panarin Y. P., Vij J. K. (2019). Liq. Cryst..

[cit43] Sreenilayam S. P., Rodriguez-Lojo D., Panov V. P., Swaminathan V., Vij J. K., Panarin Y. P., Gorecka E., Panov A., Stevenson P. J. (2017). Phys. Rev. E: Stat., Nonlinear, Soft Matter Phys..

[cit44] Yadav N., Panov V. P., Swaminathan V., Sreenilayam S. P., Vij J. K., Perova T. S., Dhar R., Panov A., Rodriguez-Lojo D., Stevenson P. J. (2017). Phys. Rev. E: Stat., Nonlinear, Soft Matter Phys..

[cit45] de Jeu W. H. , Physical Properties of Liquid Crystalline Materials, Gordon and Breach, New York, 1980

[cit46] Chandrasekhar S. , Liquid Crystals, Cambridge University Press, Cambridge, 2003

[cit47] de Gennes P. G. , The Physics of Liquid Crystals, Clarendon Press, Oxford, 1975

[cit48] Ahmed Z., Müller C., Johnston J. J., Nguyen K., Schubert C. P. J., Abitaev K., Marino S., Giesselmann F., Lemieux R. P. (2019). Liq. Cryst..

[cit49] Müller C., Schubert C. P. J., Lemieux R. P., Giesselmann F. (2018). ChemPhysChem.

[cit50] Schubert C. P. J., Müller C., Bogner A., Giesselmann F., Lemieux R. P. (2017). Soft Matter.

[cit51] Schubert C. P. J., Müller C., Giesselmann F., Lemieux R. P. (2016). J. Mater. Chem. C.

[cit52] Schubert C. P. J., Müller C., Wand M. D., Giesselmann F., Lemieux R. P. (2015). Chem. Commun..

[cit53] Schubert C. P. J., Bogner A., Porada J. H., Ayub K., Andrea T., Giesselmann F., Lemieux R. P. (2014). J. Mater. Chem. C.

[cit54] Song Q., Nonnenmacher D., Giesselmann F., Lemieux R. P. (2013). J. Mater. Chem. C.

[cit55] Roberts J. C., Kapernaum N., Giesselmann F., Lemieux R. P. (2008). J. Am. Chem. Soc..

[cit56] Li L., Jones C. D., Magolan J., Lemieux R. P. (2007). J. Mater. Chem..

[cit57] Thompson M., Carkner C., Bailey A., Mosey N. J., Kapernaum N., Lemieux R. P. (2014). Liq. Cryst..

[cit58] Thompson M., Carkner C., Mosey N. J., Kapernaum N., Lemieux R. P. (2015). Soft Matter.

[cit59] Sreenilayam S. P., Agra-Kooijman D. M., Panov V. P., Swaminathan V., Vij J. K., Panarin Y. P., Kocot A., Panov A., Rodriguez-Lojo D., Stevenson P. J., Fisch M. R., Kumar S. (2017). Phys. Rev. E: Stat., Nonlinear, Soft Matter Phys..

[cit60] Zoghaib W. M., George A. K., Carboni C., Surekha M., Ashok Kumar A. V. N., Chalapathy P. V., Potukuchi D. M. (2018). Soft Mater..

[cit61] Zoghaib W. M., Carboni C., Al-Rawahi J., Al-Rubaiei F., Al-Bulushi H., Al-Aufi M., Al-Harrasi M., Al-Kalbani S., Al-Kiyumi A. (2016). Mol. Cryst. Liq. Cryst..

[cit62] Kohout M., Bubnov A., Šturala J., Novotná V., Svoboda J. (2016). Liq. Cryst..

[cit63] Zoghaib W. M., Carboni C., Al-Hinai H., Al-Abri S., Al-Kasbi S., Al-Nasseri E., Al-Masroori M., Al-Yahyaee M., Al-Busaidi S. (2015). Mol. Cryst. Liq. Cryst..

[cit64] Yeap G.-Y., Chan T.-N., Yam W.-S., Madrak K., Pociecha D., Gorecka E. (2012). Liq. Cryst..

[cit65] Mulligan K. M., Bogner A., Song Q., Schubert C. P. J., Giesselmann F., Lemieux R. P. (2014). J. Mater. Chem. C.

[cit66] Singh H. K., Singh S. K., Nandi R., Shankar Rao D. S., Prasad S. K., Singh R. K., Singh B. (2016). RSC Adv..

[cit67] Zhang Z., Kaur S., Kundu B., Sadashiva B. K., Gleeson H. F. (2017). J. Mater. Chem. C.

[cit68] Alaasar M., Prehm M., Tamba M.-G., Sebastián N., Eremin A., Tschierske C. (2016). ChemPhysChem.

[cit69] Ryu S. H., Shin T. J., Gong T., Shen Y., Korblova E., Shao R., Walba D. M., Clark N. A., Yoon D. K. (2014). Phys. Rev. E: Stat., Nonlinear, Soft Matter Phys..

[cit70] Shen Y., Wang L., Shao R., Gong T., Zhu C., Yang H., Maclennan J. E., Walba D. M., Clark N. A. (2013). Phys. Rev. E: Stat., Nonlinear, Soft Matter Phys..

[cit71] Walba D. M., Yang H., Keller P., Zhu C., Shao R., Coleman D. A., Jones C. D., Clark N. A. (2009). Macromol. Rapid Commun..

[cit72] Kapernaum N., Walba D. M., Korblova E., Zhu C., Jones C., Shen Y., Clark N. A., Giesselmann F. (2009). ChemPhysChem.

[cit73] Walba D. M., Korblova E., Eshdat L., Biewer M. C., Yang H., Jones C., Nakata M., Talarico M., Shao R., Clark N. A. (2007). J. Soc. Inf. Disp..

[cit74] Lagerwall J. P. F., Coleman D., Körblova E., Jones C., Shao R., Otón J. M., Walba D. M., Clark N., Giesselmann F. (2006). Liq. Cryst..

[cit75] McCubbin J. A., Tong X., Zhao Y., Snieckus V., Lemieux R. P. (2005). Chem. Mater..

[cit76] McCubbin J. A., Snieckus V., Lemieux R. P. (2005). Liq. Cryst..

[cit77] McCubbin J. A., Tong X., Wang R., Zhao Y., Snieckus V., Lemieux R. P. (2004). J. Am. Chem. Soc..

[cit78] Takatoh K., Sunohara K., Sakamoto M. (1988). Mol. Cryst. Liq. Cryst..

[cit79] Harjung M. D., Schubert C. P. J., Knecht F., Porada J. H., Lemieux R. P., Giesselmann F. (2017). J. Mater. Chem. C.

[cit80] Ivanov A. V., Lyakhov S. A., Yarkova M. Y., Galatina A. I., Mazepa A. V. (2002). Russ. J. Gen. Chem..

[cit81] Lemieux R. B. (2001). Acc. Chem. Res..

[cit82] Schultz A., Laschat S., Saipa A., Giesselmann F., Nimtz M., Schulte J. L., Baro A., Miehlich B. (2004). Adv. Funct. Mater..

[cit83] Wang Y., Liu Y., Luo J., Qi H., Li X., Nin M., Liu M., Shi D., Zhu W., Cao Y. (2011). Dalton Trans..

[cit84] Subrahmanyam S. V., Chelapathi P. V., Mahabaleshwara S., Srinivasulu M., George A. K., Potukuchi D. M. (2014). Phys. B.

[cit85] Yeh M.-C., Su Y.-L., Tzeng M.-C., Ong C. W., Kajitani T., Enozawa H., Takata M., Koizumi Y., Saeki A., Seki S., Fukushima T. (2013). Angew. Chem..

[cit86] Epperson J. R., Bruce M. A., Catt J. D., Deskus J. A., Hodges D. B., Karageorge G. N., Keavy D. J., Mahle C. D., Mattson R. J., Ortiz A. A., Parker M. F., Takaki K. S., Watson B. T., Yevich J. P. (2004). Bioorg. Med. Chem..

[cit87] George S. R. D., Elton T. E., Harper J. B. (2015). Org. Biomol. Chem..

[cit88] Magano J., Chen M. H., Clark J. D., Nussbaumer T. (2006). J. Org. Chem..

[cit89] Chae J. (2008). Arch. Pharm. Res..

[cit90] Jankowiak A., Debska Z., Romański J., Kaszyński P. (2012). J. Sulfur Chem..

[cit91] Choi S., Larson M. A., Hinrichs S. H., Narayanasamy P. (2016). Bioorg. Med. Chem. Lett..

[cit92] Butschies M., Sauer S., Kessler E., Siehl H.-U., Claasen B., Fischer P., Frey W., Laschat S. (2010). ChemPhysChem.

[cit93] Mars J., Hou B., Weiss H., Li H., Konovalov O., Festersen S., Murphy B. M., Rütt U., Bier M., Mezger M. (2017). Phys. Chem. Chem. Phys..

[cit94] Nozaki Y., Yamaguchi K., Tomida K., Taniguchi N., Hara H., Takikawa Y., Sadakane K., Nakamura K., Konishi T., Fukao K. (2016). J. Phys. Chem. B.

[cit95] Nemoto F., Kofu M., Yamamuro O. (2015). J. Phys. Chem. B.

[cit96] Nishikawa K. , in Ionic Liquids Further UnCOILed, ed. N. V. Plechkova and K. R. Seddon, Wiley, Hoboken, 2014, pp. 59–85

[cit97] Abate A., Petrozza A., Cavallo G., Lanzani G., Matteucci F., Bruce D. W., Houbenov N., Metrangolo P., Resnati G. (2013). J. Mater. Chem. A.

[cit98] Niemann T., Zaitsau D., Strate A., Villinger A., Ludwig R. (2018). Sci. Rep..

[cit99] Liu H., Nohira H. (1998). Liq. Cryst..

[cit100] Petrov V. F., Vinokurov V. A., Belyaev V. V. (2010). Mol. Cryst. Liq. Cryst..

[cit101] Rupar I., Mulligan K. M., Roberts J. C., Nonnenmacher D., Giesselmann F., Lemieux R. P. (2013). J. Mater. Chem. C.

[cit102] Goodby J. W., Saez I. M., Cowling S. J., Görtz V., Draper M., Hall A. W., Sia S., Cosquer G., Lee S.-E., Raynes E. P. (2008). Angew. Chem..

[cit103] Song Q., Nonnenmacher D., Giesselmann F., Lemieux R. P. (2011). Chem. Commun..

[cit104] Neidhardt M. M., Wolfrum M., Beardsworth S., Wöhrle T., Frey W., Baro A., Stubenrauch C., Giesselmann F., Laschat S. (2016). Chem.–Eur. J..

[cit105] Cao W., Senthilkumar B., Causin V., Swamy V. P., Wang Y., Saielli G. (2020). Soft Matter.

[cit106] Kouwer P. H. J., Swager T. M. (2007). J. Am. Chem. Soc..

[cit107] Dierking I. , Textures of Liquid Crystals, Wiley-VCH, Weinheim, 2003

[cit108] Horn R. G. (1978). J. Phys..

[cit109] Hanwell M. D., Curtis D. E., Lonie D. C., Vandermeersch T., Zurek E., Hutchison G. R. (2012). J. Cheminf..

[cit110] Leadbetter A. J., Wrighton P. G. (1979). J. Phys., Colloq..

[cit111] Wöhrle T., Gündemir R., Frey W., Knecht F., Köhn A., Laschat S. (2017). Chem.–Eur. J..

[cit112] Bartolino R., Doucet J., Durand G. (1978). Ann. Phys..

[cit113] Keller E. N., Nachaliel E., Davidov D., Böffel C. (1986). Phys. Rev. A: At., Mol., Opt. Phys..

[cit114] Jang W. G., Glaser M. A., Park C. S., Kim K. H., Lansac Y., Clark N. A. (2001). Phys. Rev. E: Stat., Nonlinear, Soft Matter Phys..

[cit115] Walba D. M., Clark N. A. (1987). Proc. SPIE.

[cit116] Walba D. M., Clark N. A. (1988). Ferroelectrics.

[cit117] Walba D. M., Razavi H. A., Horiuchi A., Eidman K. F., Otterholm B., Haltiwanger R. C., Clark N. A., Shao R., Parmar D. S., Wand M. D., Vohra R. T. (1991). Ferroelectrics.

[cit118] Radcliffe M. D., Brostrom M. L., Epstein K. A., Rappaport A. G., Thomas B. N., Shao R., Clark N. A. (1999). Liq. Cryst..

[cit119] Davidson P., Strzelecki L. (1988). Liq. Cryst..

[cit120] Aeffner S., Reusch T., Weinhausen B., Salditt T. (2009). Eur. Phys. J. E.

[cit121] Davidson P., Levelut A. M. (1992). Liq. Cryst..

[cit122] Xu F., Wang H., Du X., Wang W., Wang D.-E., Chen S., Han X., Li N., Yuan M.-S., Wang J. (2016). Dyes Pigm..

[cit123] Huang T.-H., Li X.-C., Wang Y.-H., Kang Z.-H., Lu R., Miao E.-L., Wang F., Wang G.-W., Zhang H.-Z. (2013). Opt. Mater..

[cit124] Poe A., Della Pelle A., Byrnes S., Thayumanavan S. (2015). Chem.–Eur. J..

[cit125] Sergeyev S., Pisula W., Geerts Y. H. (2007). Chem. Soc. Rev..

[cit126] Diring S., Camerel F., Donnio B., Dintzer T., Toffanin S., Capelli R., Muccini M., Ziessel R. (2009). J. Am. Chem. Soc..

[cit127] Luo J., Xie Z., Lam J. W. Y., Cheng L., Chen H., Qiu C., Kwok H. S., Zhan X., Liu Y., Zhu D., Tang B. Z. (2001). Chem. Commun..

[cit128] Mei J., Leung N. L. C., Kwok R. T. K., Lam J. W. Y., Tang B. Z. (2015). Chem. Rev..

