# Supplementary material for: Fluorenone imidazolium salts as novel de Vries materials
Source: RSC Adv. 2020 Jun 23;10(40):23999–4016. doi: 10.1039/d0ra04650g (PMC9055108; doi:10.1039/d0ra04650g)
Supplement: RA-010-D0RA04650G-s001 [file RA-010-D0RA04650G-s001.pdf]

Electronic Supplementary Material (ESI) for RSC Advances.
This journal is © The Royal Society of Chemistry 2020

Supporting Information

Fluorenone Imidazolium Salts as Novel De Vries Materials

Korinna Bader, Carsten Müller, Yann Molard, Angelika Baro, Philipp Ehni, Jakob Knelles and Sabine Laschat

Table of Contents

Materials and Methods S2

Synthesis S2

Differential scanning calorimetry (DSC) S52

Polarizing optical microscopy (POM) S63

XRD data of bromides and imidazolium salts S73

Temperature-dependent layer distance S78

Optical tilt angles S79

Electron density profiles S80

Absorbance and emission measurements in solution S80

Solid state fluorescence spectroscopy S83

References S83

NMR spectra

Materials and Methods

NMR spectra were recorded on Bruker Avance 300, 500, Ascend 400 and 700 spectrometers; chemical shifts δ are given in ppm relative to tetramethylsilane (TMS) as internal standard. Mass spectra were recorded on a Finnigan MAT 711 instrument in electron ionization (EI) mode and high resolution mass spectra on a Bruker Daltonics mikro-TOF-Q spectrometer in electron spray ionization (ESI) mode. IR spectra were recorded on a Bruker Vektor 22 FTIRspectrometer with MKII Golden Gate Single Reflection Diamant ATR system. Differential scanning calorimetry (DSC) was performed on a Mettler-Toledo DSC822e instrument with a scanning rate of 5 K min^{-1}, unless otherwise noted. Texture analyses were performed using an Olympus BX 50 polarized microscope fitted with a Linkam LTS 350 hot stage and temperature controller. Optical tilt angles were measured by polarized microscopy in the absence of an electric field by measuring the angle of rotation between dark states in domains of opposite tilt orientation with the sample aligned in glass cells with a rubbed polyimide alignment layer (4 µm spacing) by slow cooling from the isotropic layer to the SmC phase.[1] Temperature-dependent emissions were measured using a Nikon 80i polarized microscope fitted with a Linkam LTS 420 hot stage, a Nikon Intensilight C-HGFI (UV 1 filter, 350 nm<λ_{exc}<380 nm) light source, a Nikon DS-F12 camera and an Ocean Optics QE650000 photometer. X-ray diffraction measurements were performed using a Bruker AXS Nanostar C with $Cu_{K\alpha}$ radiation (λ = 1.5405 Å). The samples were filled into Mark capillary tubes of 7 mm diameter; calibration with silver behenate at 298 K. Thermogravimetry (TGA) was performed using a Netzsch STA 449 Jupiter under Ar atmosphere. UV/Vis spectra were recorded on a Shimadzu UV 160 spectrometer. Emission spectra were recorded using a Perkin Elmer LS 55 spectrometer.

Synthesis

4,4,5,5-Tetramethyl-2-(4-(tetradecyloxy)phenyl)-1,3,2-dioxaborolan (7d). Following a literature procedure,[2] to a solution of 1-bromo-4-(tetradecyloxy)benzene (3.00 g, 5.57 mmol) in abs. THF (40 mL) at –78 °C under N_2 atmosphere a 1.6 M solution of *n*-BuLi in hexane (5.23 mL, 8.36 mmol) was added and the reaction mixture stirred for 1 h at –78 °C. Then 2-isopropoxy-4,4,5,5-tetramethyl-1,3,2-dioxaborolan (2.27 mL, 2.07 g, 11.14 mmol) was added and the reaction mixture was stirred at room temperature overnight. A satd. solution of NH_4Cl in H_2O (25 mL) was added, the mixture stirred for 20 min and the aqueous suspension extracted with Et_2O (30 mL). The organic layer was washed with H_2O (3 x 50 mL), dried ($MgSO_4$) and the solvent was removed under reduced pressure. The residue was purified by chromatography on SiO_2 with hexanes/Et_2O (60:1). After drying under high vacuum, **7d** was obtained as a colorless solid (2.67 g, 6.41 mmol, 79 %). **^{1}H-NMR** (300 MHz, $CDCl_3$): δ = 0.79–0.96 (m, 3H, CH_3), 1.15–1.53 (m, 34H, CH_2, $OC(CH_3)_2$), 1.68–1.88 (m, 2H, OCH_2CH_2), 3.97 (t, *J* = 6.6 Hz, 2H, OCH_2), 6.82–6.95 (m, 2H, 3-H), 7.61–7.85 (m, 2H, 2-H) ppm; **^{13}C-NMR** (75 MHz, $CDCl_3$): δ = 14.1 (CH_3), 22.7 (CH_3CH_2), 24.9, 26.0, 26.9, 29.2, 29.37, 29.40, 29.58, 29.60, 29.66, 29.68, 29.70 (CH_2), 31.9 ($CH_3CH_2CH_2$), 67.8 (OCH_2), 83.5 ($OC(CH_3)_2$, 113.9 (C-3), 136.5 (C-2), 161.8 (C-4) ppm; **FT-IR**

(ATR): $\tilde{\nu}$ = 2977 (w), 2923 (s), 2854 (m), 1605 (s), 1569 (w), 1516 (w), 1467 (w), 1410 (w), 1397 (m), 1361 (vs), 1318 (m), 1275 (m), 1246 (s), 1175 (m), 1144 (s), 1091 (m), 1012 (w), 963 (w), 861 (w), 832 (w), 736 (w), 671 (w), 655 (w) cm^{-1}; **MS** (ESI): *m/z* = 439 $[M+Na]^+$, 417, 221; **HRMS** (ESI): calcd. for $[C_{26}H_{45}BO_3Na]^+$ 439.3354, found: 439.3349 $[M+Na]^+$; m.p. 45 °C.

General Procedure for Suzuki Couplings (GP1)[3]

To a solution of methyl 2-bromo-5-methoxybenzoate **6** (1.85 mmol) and the appropriate **7a-f** (2.41 mmol) in degassed DME (20 mL) and degassed H_2O (20 mL) under N_2 atmosphere K_2CO_3 (5.11 g, 37.0 mmol) and $Pd(PPh_3)_4$ (22 mg, 19 µmol) were added, and the reaction mixture was stirred for 18 h at reflux. After cooling to room temperature, the layers were separated and the aqueous layer was extracted with CH_2Cl_2 (3 x 30 mL). The combined organic layers were washed with H_2O (100 mL), dried ($MgSO_4$), and the solvent was removed under vacuum. The residue was purified by chromatography on SiO_2 with hexanes/Et_2O (10:1) to give products **8**.

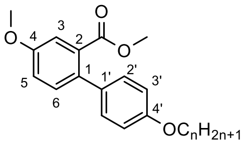

Methyl 4-methoxy-4'-(octyloxy)-1,1'-biphenyl-2-carboxylate (8a). According to GP1, from **6** (419 mg, 1.71 mmol), **7a** (738 mg, 2.22 mmol), K_2CO_3 (4.73 g, 34.2 mmol), $Pd(PPh_3)_4$ (20 mg, 17 µmol), DME (20 mL), H_2O (20 mL); yield: 633 mg, 1.71 mmol, quant., colorless oil. **^{1}H-NMR** (400 MHz, $CDCl_3$): δ = 0.84–0.94 (m, 3H, CH_3), 1.22–1.51 (m, 10H, CH_2), 1.74–1.85 (m, 2H, OCH_2CH_2), 3.65 (s, 3H, $COOCH_3$), 3.86 (s, 3H, $COCH_3$), 3.98 (t, J = 6.6 Hz, 2H, OCH_2), 6.87–6.93 (m, 2H, 3'-H), 7.04 (dd, $J_{5,6}$ = 8.5 Hz, $J_{3,5}$ = 2.8 Hz, 1H, 5-H), 7.16–7.21 (m, 2H, 2'-H), 7.25–7.28 (m, 1H, 6-H), 7.30 (d, $J_{3,5}$ = 2.8 Hz, 1H, 3-H) ppm; **^{13}C-NMR** (101 MHz, $CDCl_3$): δ = 14.1 (CH_3), 22.7 (CH_3CH_2), 26.1, 26.9, 29.3, 29.35, 29.40 (CH_2), 31.8 ($CH_3CH_2CH_2$), 52.0 ($COOCH_3$), 55.5 ($COCH_3$), 68.0 (OCH_2), 114.1 (C-3'), 114.3 (C-3), 117.5 (C-5), 129.4 (C-2'), 131.7 (C-6), 131.9 (C-2), 133.1 (C-1'), 134.6 (C-1), 158.3 (C-4, C-4'), 169.3 (*C*=O) ppm; **FT-IR** (ATR): $\tilde{\nu}$ = 2926 (w), 2855 (w), 1719 (m), 1607 (m), 1579 (w), 1520 (w), 1489 (s), 1468 (m), 1434 (m), 1405 (w), 1319 (m), 1285 (s), 1240 (s), 1220 (vs), 1176 (s), 1135 (w), 1081 (m), 1046 (s), 998 (w), 985 (w), 909 (w), 875 (w), 822 (s), 782 (m), 731 (m), 576 (w), 548 (m) cm^{-1}; **MS** (ESI): *m/z* = 393 $[M+Na]^+$, 339, 275, 227, 212, 199, 184, 171, 156, 128; **HRMS** (ESI): calcd. for $[C_{23}H_{30}O_4Na]^+$ 393.2036, found: 393.2039 $[M+Na]^+$.

Methyl 4-methoxy-4'-(decyloxy)-1,1'-biphenyl-2-carboxylate (8b). According to GP1, from **6** (453 mg, 1.85 mmol), **7b** (868 mg, 2.41 mmol), K_2CO_3 (5.11 g, 37.0 mmol), $Pd(PPh_3)_4$ (22 mg, 19 µmol), DME (20 mL), H_2O (20 mL); yield: 737 mg, 1.85 mmol, quant., colorless oil. **^{1}H-NMR** (700 MHz, $CDCl_3$): δ = 0.89 (t, J = 7.0 Hz, 3H, CH_3), 1.22–1.41 (m, 12H, CH_2), 1.41–1.52 (m, 2H, $OCH_2CH_2CH_2$), 1.75–1.85 (m, 2H, OCH_2CH_2), 3.65 (s, 3H, $COOCH_3$), 3.86 (s, 3H, $COCH_3$), 3.98 (t, J = 6.6 Hz, 2H, OCH_2), 6.88–6.92 (m, 2H, 3'-H), 7.04 (dd, $J_{5,6}$ = 8.5 Hz, $J_{3,5}$ = 2.8 Hz, 1H, 5-H), 7.17–7.21 (m, 2H, 2'-H), 7.25–7.28 (m, 1H, 6-H), 7.30 (d, $J_{3,5}$ = 2.8 Hz, 1H, 3-H) ppm; **^{13}C-NMR** (176 MHz, $CDCl_3$): δ = 14.1 (CH_3), 22.7 (CH_3CH_2), 26.1, 29.3, 29.34,

29.35, 29.58, 29.60 (*C*H_2), 31.9 (CH_3CH_2*C*H_2), 52.0 (COO*C*H_3), 55.5 (CO*C*H_3), 68.0 (O*C*H_2), 114.1 (C-3'), 114.3 (C-3), 117.5 (C-5), 129.4 (C-2'), 131.7 (C-6), 131.9 (C-2), 133.1 (C-1'), 134.6 (C-1), 158.3 (C-4, C-4'), 169.3 (*C*=O) ppm; **FT-IR** (ATR): $\tilde{\nu}$ = 2925 (s), 2854 (m), 1721 (m), 1608 (m), 1579 (w), 1520 (w), 1490 (s), 1469 (m), 1434 (m), 1405 (w), 1320 (w), 1288 (s), 1245 (vs), 1225 (s), 1177 (m), 1135 (w), 1082 (w), 1048 (m), 999 (w), 876 (w), 824 (w), 784 (w), 551 (w) cm^{-1}; **MS** (EI): *m/z* = 398.2 (100) $[M]^{+\cdot}$, 258.1 (75) $[M–C_{10}H_{21}]^+$, 227.1 (20) $[M–OC_{14}H_{29}–CH_3]^+$; **HRMS** (EI): calcd. for $[C_{25}H_{34}O_4]^+$ 398.2457, found: 398.2452 $[M]^+$.

Methyl 4-methoxy-4'-(dodecyloxy)-1,1'-biphenyl-2-carboxylate (8c). According to GP1, from **6** (400 mg, 1.63 mmol), **7c** (823 mg, 2.12 mmol), K_2CO_3 (4.51 g, 32.6 mmol), $Pd(PPh_3)_4$ (19 mg, 16 µmol), DME (20 mL), H_2O (20 mL); yield: 695 mg, 1.63 mmol, quant., colorless solid. **^{1}H-NMR** (300 MHz, $CDCl_3$): δ = 0.81–0.94 (m, 3H, C*H_3*), 1.19–1.53 (m, 18H, C*H_2*), 1.72–1.86 (m, 2H, OCH_2C*H_2*), 3.65 (s, 3H, COOC*H_3*), 3.86 (s, 3H, COC*H_3*), 3.98 (t, *J* = 6.6 Hz, 2H, OC*H_2*), 6.87–6.94 (m, 2H, 3'-H), 7.04 (dd, $J_{5,6}$ = 8.5 Hz, $J_{3,5}$ = 2.8 Hz, 1H), 7.15–7.22 (m, 2H, 2'-H), 7.27 (d, $J_{5,6}$ = 8.5 Hz, 1H, 6-H), 7.30 (d, *J* = 2.8 Hz, 1H, 3-H) ppm; **^{13}C-NMR** (75 MHz, $CDCl_3$): δ = 14.1 (*C*H_3), 22.7 (CH_3*C*H_2), 24.8, 26.1, 26.9, 29.36, 29.44, 29.60, 29.62, 29.65, 29.68 (*C*H_2), 31.9 (CH_3CH_2*C*H_2), 52.0 (COO*C*H_3), 55.5 (CO*C*H_3), 68.0 (O*C*H_2), 114.1 (C-3'), 114.3 (C-3), 117.5 (C-5), 129.4 (C-2'), 131.6 (C-6), 131.9 (C-2), 133.1 (C-1'), 134.6 (C-1), 158.3 (C-4, C-4'), 169.3 (*C*=O) ppm; **FT-IR** (ATR): $\tilde{\nu}$ = 2922 (s), 2853 (m), 1720 (m), 1607 (m), 1579 (w), 1520 (w), 1489 (s), 1468 (m), 1434 (m), 1320 (w), 1286 (s), 1242 (vs), 1225 (vs), 1177 (m), 1136 (w), 1082 (m), 1048 (m), 999 (w), 876 (w), 824 (m), 783 (w), 577 (w), 551 (w) cm^{-1}; **MS** (EI): *m/z* = 426.3 (100) $[M]^{+\cdot}$, 258.1 (75) $[M–C_{12}H_{25}]^+$, 227.1 (15) $[M–OC_{12}H_{25}–CH_3]^+$; **HRMS** (EI): calcd. for $[C_{27}H_{38}O_4]^+$ 426.2770, found: 426.2767 $[M]^+$; m.p. 54 °C.

Methyl 4-methoxy-4'-(tetradecyloxy)-1,1'-biphenyl-2-carboxylate (8d). According to GP1, from **6** (667 mg, 2.72 mmol), **7d** (1.47 g, 3.54 mmol), K_2CO_3 (7.52 g, 54.4 mmol), $Pd(PPh_3)_4$ (35 mg, 30 µmol), DME (20 mL), H_2O (20 mL); yield: 1.24 g, 2.72 mmol, quant., colorless solid. **^{1}H-NMR** (300 MHz, $CDCl_3$): δ = 0.83–0.95 (m, 3H, C*H_3*), 1.21–1.39 (m, 20H, C*H_2*), 1.39–1.53 (m, 2H, OCH_2CH_2C*H_2*), 1.73–1.86 (m, 2H, OCH_2C*H_2*), 3.66 (s, 3H, COOC*H_3*), 3.86 (s, 3H, COC*H_3*), 3.98 (t, *J* = 6.6 Hz, 2H, OC*H_2*), 6.86–6.94 (m, 2H, 3'-H), 7.04 (dd, $J_{5,6}$ = 8.5 Hz, $J_{3,5}$ = 2.8 Hz, 1H, 5-H), 7.15–7.22 (m, 2H, 2'-H), 7.27 (d, $J_{5,6}$ = 8.5 Hz, 1H, 6-H), 7.30 (d, $J_{3,5}$ = 2.8 Hz, 1H, 3-H) ppm; **^{13}C-NMR** (75 MHz, $CDCl_3$): δ = 14.1 (*C*H_3), 22.7 (CH_3*C*H_2), 26.1, 29.3, 29.35, 29.37, 29.60, 29.62, 29.67, 29.69 (*C*H_2), 31.9 (CH_3CH_2*C*H_2), 52.0 (COO*C*H_3), 55.5 (CO*C*H_3), 68.0 (O*C*H_2), 114.1 (C-3'), 114.3 (C-3), 117.5 (C-5), 129.4 (C-2'), 131.6 (C-6), 131.9 (C-2), 133.1 (C-1'), 134.6 (C-1), 158.3 (C-4, C-4'), 169.3 (*C*=O) ppm; **FT-IR** (ATR): $\tilde{\nu}$ = 2922 (s), 2852 (m), 1720 (m), 1607 (m), 1579 (w), 1520 (w), 1489 (s), 1468 (m), 1434 (m), 1320 (w), 1286 (s), 1242 (vs), 1225 (vs), 1177 (m), 1135 (w), 1081 (m), 1048 (m), 999 (w), 876 (w), 823 (m), 783 (w), 722 (w), 577 (w), 549 (w) cm^{-1}; **MS** (EI): *m/z* = 454.3 (100) $[M]^{+\cdot}$, 258.1 (55) $[M–C_{14}H_{29}]^+$, 227.1 (15) $[M–OC_{14}H_{29}–CH_3]^+$; **HRMS** (EI): calcd. for $[C_{29}H_{42}O_4]^+$ 454.3083, found: 454.3079 $[M]^+$; m.p. 57 °C.

Methyl 4-methoxy-4'-(hexadecyloxy)-1,1'-biphenyl-2-carboxylate (8e). According to GP1, from **6** (887 mg, 3.62 mmol), **7e** (2.09 g, 4.70 mmol), K_2CO_3 (10.00 g, 72.4 mmol), $Pd(PPh_3)_4$ (42 mg, 40 µmol), DME (25 mL), H_2O (25 mL); yield: 1.75 g, 3.62 mmol, quant., colorless solid. **^{1}H-NMR** (500 MHz, $CDCl_3$): δ = 0.88 (t, J = 6.9 Hz, 3H, CH_3), 1.20–1.40 (m, 24H, CH_2), 1.42–1.51 (m, 2H, $OCH_2CH_2CH_2$), 1.76–1.85 (m, 2H, OCH_2CH_2), 3.66 (s, 3H, COOCH_3), 3.86 (s, 3H, COCH_3), 3.98 (t, J = 6.6 Hz, 2H, OCH_2), 6.88–6.92 (m, 2H, 3'-H), 7.05 (dd, $J_{5,6}$ = 8.5 Hz, $J_{3,5}$ = 2.8 Hz, 1H, 5-H), 7.16–7.21 (m, 2H, 2'-H), 7.25–7.29 (m, 1H, 6-H), 7.30 (d, $J_{3,5}$ = 2.8 Hz, 1H, 3-H) ppm; **^{13}C-NMR** (126 MHz, $CDCl_3$): δ = 14.1 (CH_3), 22.7 (CH_3CH_2), 26.1, 29.3, 29.37, 29.44, 29.6, 29.6, 29.67, 29.68, 29.71 (CH_2), 31.9 ($CH_3CH_2CH_2$), 52.0 ($COOCH_3$), 55.5 ($COCH_3$), 68.0 (OCH_2), 114.0 (C-3'), 114.2 (C-3), 117.5 (C-5), 129.4 (C-2'), 131.6 (C-6), 131.9 (C-2), 133.1 (C-1'), 134.6 (C-1), 158.3 (C-4, C-4'), 169.3 (*C*=O) ppm; **FT-IR** (ATR): $\tilde{\nu}$ = 2922 (s), 2852 (m), 1720 (m), 1607 (m), 1579 (w), 1519 (w), 1490 (s), 1467 (m), 1434 (m), 1320 (w), 1286 (s), 1242 (vs), 1225 (vs), 1177 (m), 1135 (w), 1081 (m), 1048 (s), 999 (w), 876 (w), 823 (m), 783 (w), 722 (w), 577 (w), 549 (w) cm^{-1}; **MS** (EI): m/z = 482.3 (100) $[M]^{+\cdot}$, 258.1 (45) $[M–C_{16}H_{33}]^+$, 227.1 (15) $[M–OC_{16}H_{33}–CH_3]^+$; **HRMS** (EI): calcd. for $[C_{31}H_{46}O_4]^+$ 482.3396, found: 482.3397 $[M]^+$; m.p. 64 °C.

Methyl 4'-fluoro-4-methoxy-1,1'-biphenyl-2-carboxylate (8f). According to GP1, from **6** (2.12 g, 8.66 mmol), **7f** (2.50 g, 11.26 mmol), K_2CO_3 (11.97 g, 86.6 mmol), $Pd(PPh_3)_4$ (101 mg, 80 µmol), DME (30 mL), H_2O (30 mL); yield: 2.24 g, 8.61 mmol, 99 %, colorless oil. **^{1}H-NMR** (500 MHz, $CDCl_3$): δ = 3.65 (s, 3H, COOCH_3), 3.87 (s, 3H, COCH_3), 7.03–7.09 (m, 3H, 3'-H, 5-H), 7.20–7.27 (m, 3H, 2'-H, 6-H), 7.35 (d, $J_{3,5}$ = 2.8 Hz, 1H, 3-H) ppm; **^{13}C-NMR** (126 MHz, $CDCl_3$): δ = 52.0 ($COOCH_3$), 55.6 ($COCH_3$), 114.9 (d, J = 21.5 Hz, C-3'), 115.0 (C-3), 117.6 (C-5), 130.0 (d, J = 8.0 Hz, C-2'), 131.6 (C-6), 131.9 (C-2), 134.0 (C-1), 137.1 (d, J = 3.4 Hz, C-1'), 158.7 (C-4), 162.1 (d, J = 245.6 Hz, C-4'), 168.7 (*C*=O) ppm; **^{19}F-NMR** (376 MHz, $CDCl_3$): δ = -116.1 ppm; **FT-IR** (ATR): $\tilde{\nu}$ = 2951 (w), 2839 (w), 1718 (s), 1603 (m), 1568 (w), 1488 (s), 1434 (m), 1318 (m), 1285 (s), 1215 (vs), 1183 (m), 1158 (m), 1137 (w), 1081 (s), 1043 (s), 1005 (w), 982 (w), 876 (w), 817 (s), 780 (m), 677 (w), 559 (w), 537 (m), 470 (w) cm^{-1}; **MS** (ESI): m/z = 283 $[M]^+$, 229, 193, 151, 137, 123; **HRMS** (ESI): calcd. for $[C_{15}H_{13}FO_3Na]^+$ 283.0741, found: 283.0733 $[M+Na]^+$.

General Procedure for the Saponification of Esters 8 (GP2)[4]

A solution of the appropriate **8a**–**f** (1.81 mmol) in EtOH (20 mL) and NaOH (20 mL, 10%ic in H_2O) was heated for 3 h at reflux. The reaction mixture was then adjusted with conc. HCl to pH = 2 and extracted with Et_2O (3 x 30 mL). The combined organic layers were washed with H_2O (100 mL), dried ($MgSO_4$), the solvent was removed under reduced pressure and the residue dried under high vacuum to give products **9a-f**.

4-Methoxy-4'-(octyloxy)-1,1'-biphenyl-2-carboxylic acid (9a). According to GP2, from **8a** (650 mg, 1.75 mmol), NaOH (20 mL, 10%ic in H_2O), EtOH (20 mL); yield: 580 mg, 1.63 mmol, 93%, colorless solid. **^{1}H-NMR** (300 MHz, $CDCl_3$): δ = 0.83–0.97 (m, 3H, CH_3), 1.24–1.40 (m, 8H,

CH_2), 1.40–1.55 (m, 2H, $OCH_2CH_2CH_2$), 1.72–1.87 (m, 2H, OCH_2CH_2), 3.86 (s, 3H, OCH_3), 3.97 (t, J = 6.6 Hz, 2H, OCH_2), 6.85–6.93 (m, 2H, 3'-H), 7.08 (dd, $J_{5,6}$ = 8.5 Hz, $J_{3,5}$ = 2.8 Hz, 1H, 5-H), 7.19–7.24 (m, 2H, 2'-H), 7.24–7.28 (m, 1H, 6-H), 7.44 (d, $J_{3,5}$ = 2.8 Hz, 1H, 3-H) ppm; **^{13}C-NMR** (75 MHz, $CDCl_3$): δ = 14.1 (CH_3), 22.7 (CH_3CH_2), 26.1, 29.3, 29.36, 29.41 (CH_2), 31.8 ($CH_3CH_2CH_2$), 55.6 (OCH_3), 68.0 (OCH_2), 114.1 (C-3'), 115.0 (C-3), 118.5 (C-5), 129.7 (C-2'), 130.0 (C-2), 132.4 (C-6), 132.8 (C-1'), 135.6 (C-1), 158.3 (C-4), 158.5 (C-4'), 173.1 (*C*=O) ppm; **FT-IR** (ATR): $\tilde{\nu}$ = 3033 (w), 2924 (m), 2854 (m), 2662 (w), 2556 (w), 1688 (s), 1607 (m), 1578 (w), 1561 (w), 1489 (m), 1451 (m), 1434 (m), 1391 (w), 1293 (s), 1228 (vs), 1175 (m), 1146 (w), 1109 (w), 1089 (w), 1047 (s), 999 (w), 947 (w), 902 (w), 821 (m), 764 (w), 607 (w), 583 (w), 543 (w) cm^{-1}; **MS** (EI): *m/z* = 356.2 (65) $[M]^{+\cdot}$, 244.1 (100) $[M - C_8H_{17}]^+$, 229.0 (10) $[M–C_8H_{17}–CH_3]^+$; **HRMS** (EI): ber. für $[C_{22}H_{28}O_4]^+$ 356.1988, gef. 356.1985 $[M]^+$; m.p. 95 °C.

4-Methoxy-4'-(decyloxy)-1,1'-biphenyl-2-carboxylic acid (9b). According to GP2, from **8b** (720 mg, 1.81 mmol), NaOH (20 mL, 10%ic in H_2O), EtOH (20 mL); yield: 696 mg, 1.81 mmol, quant., colorless solid. **^{1}H-NMR** (700 MHz, $CDCl_3$): δ = 0.88 (t, J = 7.1 Hz, 3H, CH_3), 1.20–1.40 (m, 12H, CH_2), 1.42–1.51 (m, 2H, $OCH_2CH_2CH_2$), 1.75–1.85 (m, 2H, OCH_2CH_2), 3.86 (s, 3H, OCH_3), 3.97 (t, J = 6.6 Hz, 2H, OCH_2), 6.87–6.91 (m, 2H, 3'-H), 7.08 (dd, $J_{5,6}$ = 8.5 Hz, $J_{3,5}$ = 2.8 Hz, 1H, 5-H), 7.20–7.23 (m, 2H, 2'-H), 7.24–7.28 (m, 1H, 6-H), 7.43 (d, $J_{3,5}$ = 2.8 Hz, 1H, 3-H) ppm; **^{13}C-NMR** (176 MHz, $CDCl_3$): δ = 14.1 (CH_3), 22.7 (CH_3CH_2), 26.1, 29.3, 29.34, 29.36, 29.58, 29.61 (CH_2), 31.9 ($CH_3CH_2CH_2$), 55.6 (OCH_3), 68.0 (OCH_2), 114.1 (C-3'), 115.0 (C-3), 118.5 (C-5), 129.7 (C-2'), 130.0 (C-2), 132.4 (C-6), 132.8 (C-1'), 135.5 (C-1), 158.3 (C-4), 158.5 (C-4'), 172.8 (*C*=O) ppm; **FT-IR** (ATR): $\tilde{\nu}$ = 3036 (w), 2921 (s), 2851 (m), 2667 (w), 2576 (w), 1687 (s), 1607 (m), 1578 (w), 1562 (w), 1492 (m), 1477 (m), 1449 (w), 1434 (w), 1423 (w), 1398 (w), 1308 (m), 1294 (s), 1284 (s), 1260 (m), 1240 (m), 1225 (vs), 1177 (m), 1145 (w), 1107 (w), 1091 (w), 1048 (m), 999 (w), 944 (w), 905 (w), 827 (m), 765 (w), 575 (w), 538 (w) cm^{-1}; **MS** (EI): *m/z* = 384.2 (100) $[M]^{+\cdot}$, 244.1 (95) $[M–C_{10}H_{21}]^+$, 229.1 (10) $[M–C_{11}H_{24}]^+$; **HRMS** (EI): calcd. for $[C_{24}H_{32}O_4]^+$ 384.2301, found: 384.2300 $[M]^+$; m.p. 102 °C.

4-Methoxy-4'-(dodecyloxy)-1,1'-biphenyl-2-carboxylic acid (9c). According to GP2, from **8c** (830 mg, 1.95 mmol), NaOH (20 mL, 10%ic in H_2O), EtOH (20 mL); yield: 707 mg, 1.71 mmol, 88%, colorless solid. **^{1}H-NMR** (400 MHz, $CDCl_3$): δ = 0.88 (t, J = 6.7 Hz, 3H, CH_3), 1.19–1.40 (m, 16H, CH_2), 1.40–1.55 (m, 2H, $OCH_2CH_2CH_2$), 1.72–1.87 (m, 2H, OCH_2CH_2), 3.87 (s, 3H, OCH_3), 3.97 (t, J = 6.6 Hz, 2H, OCH_2), 6.87–6.92 (m, 2H, 3'-H), 7.08 (dd, $J_{5,6}$ = 8.5 Hz, $J_{3,5}$ = 2.8 Hz, 1H, 5-H), 7.19–7.24 (m, 2H, 2'-H), 7.24–7.28 (m, 1H, 6-H), 7.44 (d, $J_{3,5}$ = 2.8 Hz, 1H, 3-H) ppm; **^{13}C-NMR** (101 MHz, $CDCl_3$): δ = 14.1 (CH_3), 22.7 (CH_3CH_2), 26.1, 29.4, 29.5, 29.61, 29.63, 29.65, 29.69 (CH_2), 31.9 ($CH_3CH_2CH_2$), 55.6 (OCH_3), 68.0 (OCH_2), 114.1 (C-3'), 115.0 (C-3), 118.5 (C-5), 129.7 (C-2'), 129.9 (C-2), 132.4 (C-6), 132.8 (C-1'), 135.5 (C-1), 158.3 (C-4), 158.5 (C-4'), 172.8 (*C*=O) ppm; **FT-IR** (ATR): $\tilde{\nu}$ = 3037 (w), 2919 (vs), 2850 (m), 2668 (w), 2568 (w), 1687 (s), 1608 (m), 1577 (w), 1562 (w), 1492 (m), 1477 (m), 1450 (m), 1422 (w), 1396 (w), 1294 (s), 1284 (s), 1261 (m), 1240 (m), 1226 (vs), 1177 (m), 1145 (w), 1107 (w), 1091 (w), 1047 (s), 997 (w), 944 (w), 907 (w), 827 (m), 765 (w), 720 (w), 575 (w), 539 (w) cm^{-1}; **MS**

(ESI): *m/z* = 435 $[M+Na]^+$, 395, 227; **HRMS** (ESI): calcd. for $[C_{26}H_{36}O_4Na]^+$ 435.2506, found: 435.2533 $[M+Na]^+$; m.p. 97 °C.

4-Methoxy-4'-(tetradecyloxy)-1,1'-biphenyl-2-carboxylic acid (9d). According to GP2, from **8d** (1.30 g, 2.86 mmol), NaOH (25 mL, 10%ic in H_2O), EtOH (25 mL); yield: 1.26 mg, 2.86 mmol, quant., colorless solid. **^{1}H-NMR** (300 MHz, $CDCl_3$): δ = 0.81–0.98 (m, 3H, C*H*$_3$), 1.18–1.40 (m, 20H, C*H*$_2$), 1.40–1.54 (m, 2H, OCH_2CH_2C*H*$_2$), 1.79 (m_c, 2H, OCH_2C*H*$_2$), 3.87 (s, 3H, OC*H*$_3$), 3.97 (t, J = 6.6 Hz, 2H, OC*H*$_2$), 6.87–6.94 (m, 2H, 3'-H), 7.08 (dd, $J_{5,6}$ = 8.5 Hz, $J_{3,5}$ = 2.8 Hz, 1H, 5-H), 7.19–7.24 (m, 2H, 2'-H), 7.24–7.29 (m, 1H, 6-H), 7.44 (d, $J_{3,5}$ = 2.8 Hz, 1H, 3-H) ppm; **^{13}C-NMR** (101 MHz, $CDCl_3$): δ = 14.1 (*C*H$_3$), 22.7 (CH_3*C*H$_2$), 26.1, 29.4, 29.5, 29.61, 29.63, 29.67, 29.69 (*C*H$_2$), 31.9 (CH_3CH_2*C*H$_2$), 55.6 (O*C*H$_3$), 68.0 (O*C*H$_2$), 114.2 (C-3'), 115.0 (C-3), 118.4 (C-5), 129.7 (C-2'), 129.9 (C-2), 132.4 (C-6), 132.7 (C-1'), 135.4 (C-1), 158.3 (C-4), 158.6 (C-4'), 171.2 (*C*=O) ppm; **FT-IR** (ATR): $\tilde{\nu}$ = 3039 (w), 2919 (vs), 2850 (s), 2670 (w), 2560 (w), 1688 (s), 1607 (m), 1578 (w), 1562 (w), 1492 (m), 1477 (m), 1450 (m), 1422 (w), 1396 (w), 1293 (s), 1284 (s), 1262 (m), 1240 (m), 1226 (vs), 1177 (m), 1144 (w), 1106 (w), 1092 (w), 1047 (s), 998 (w), 943 (w), 908 (w), 827 (m), 766 (w), 718 (w), 575 (w), 538 (w) cm^{-1}; **MS** (ESI): *m/z* = 439 $[M+Na]^+$, 293; **HRMS** (ESI): calcd. for $[C_{28}H_{40}O_4]^+$ 439.2854, found: 439.2840 $[M]^+$; m.p. 90 °C.

4-Methoxy-4'-(hexadecyloxy)-1,1'-biphenyl-2-carboxylic acid (9e). According to GP2, from **8e** (1.82 g, 3.77 mmol), NaOH (25 mL, 10%ic in H_2O), EtOH (25 mL); yield: 1.77 mg, 3.77 mmol, quant., colorless solid. **^{1}H-NMR** (300 MHz, $CDCl_3$): δ = 0.83–0.94 (m, 3H, C*H*$_3$), 1.17–1.41 (m, 24H, C*H*$_2$), 1.40–1.54 (m, 2H, OCH_2CH_2C*H*$_2$), 1.79 (m_c, 2H, OCH_2C*H*$_2$), 3.87 (s, 3H, OC*H*$_3$), 3.97 (t, J = 6.5 Hz, 2H, OC*H*$_2$), 6.86–6.93 (m, 2H, 3'-H), 7.08 (dd, $J_{5,6}$ = 8.5 Hz, $J_{3,5}$ = 2.8 Hz, 1H, 5-H), 7.19–7.24 (m, 2H, 2'-H), 7.24–7.29 (m, 1H, 6-H), 7.44 (d, $J_{3,5}$ = 2.8 Hz, 1H, 3-H) ppm; **^{13}C-NMR** (101 MHz, $CDCl_3$): δ = 14.1 (*C*H$_3$), 22.7 (CH_3*C*H$_2$), 26.1, 29.4, 29.5, 29.61, 29.64, 29.67, 29.69, 29.71 (*C*H$_2$), 31.9 (CH_3CH_2*C*H$_2$), 55.6 (O*C*H$_3$), 68.0 (O*C*H$_2$), 114.2 (C-3'), 115.0 (C-3), 118.4 (C-5), 129.7 (C-2'), 129.9 (C-2), 132.4 (C-6), 132.7 (C-1'), 135.5 (C-1), 158.3 (C-4), 158.5 (C-4'), 171.6 (*C*=O) ppm; **FT-IR** (ATR): $\tilde{\nu}$ = 2920 (vs), 2850 (s), 2663 (w), 1691 (s), 1608 (m), 1562 (m), 1518 (w), 1491 (m), 1471 (m), 1452 (m), 1422 (w), 1389 (w), 1296 (s), 1229 (s), 1173 (m), 1105 (w), 1047 (s), 999 (w), 945 (w), 903 (w), 823 (w), 766 (w), 720 (w), 583 (w), 544 (w) cm^{-1}; **MS** (ESI): *m/z* = 467 $[M]^+$, 293; **HRMS** (ESI): calcd. for $[C_{30}H_{44}O_4]^+$ 467.3167, found: 467.3161 $[M]^+$; m.p. 100 °C.

4'-Fluoro-4-methoxy-1,1'-biphenyl-2-carboxylic acid (9f). According to GP2, from **8f** (845 mg, 3.25 mmol), NaOH (15 mL, 10%ic in H_2O), EtOH (15 mL); yield: 782 mg, 3.18 mmol, 98%, colorless solid. **^{1}H-NMR** (400 MHz, $CDCl_3$): δ = 3.87 (s, 3H, OC*H*$_3$), 7.00–7.13 (m, 3H, 3'-H, 5-H), 7.21–7.28 (m, 3H, 2'-H, 6-H), 7.47 (d, $J_{3,5}$ = 2.8 Hz, 1H, 3-H), 10.82 (s, 1H, COO*H*) ppm; **^{13}C-NMR** (101 MHz, $CDCl_3$): δ = 55.6 (O*C*H$_3$), 114.9 (d, J = 21.5 Hz, C-3'), 115.3 (C-3), 118.6 (C-5), 129.9 (C-2), 130.2 (d, J = 8.1 Hz, C-2'), 132.4 (C-6), 135.0 (C-1), 136.8 (d, J = 3.3 Hz, C-1'), 158.7 (C-4), 162.2 (d, J = 245.9 Hz, C-4'), 172.8 (*C*=O) ppm; **^{19}F-NMR** (376 MHz, $CDCl_3$):

δ = –115.9 ppm; **FT-IR** (ATR): $\tilde{\nu}$ = 3078 (w), 3003 (w), 2941 (w), 2909 (w), 1695 (s), 1604 (m), 1567 (w), 1491 (vs), 1465 (w), 1430 (w), 1316 (m), 1285 (m), 1225 (vs), 1184 (w), 1159 (w), 1093 (w), 1046 (w), 1005 (w), 899 (w), 826 (m), 792 (w), 757 (w), 668 (w), 536 (w), 434 (w) cm^{-1}; **MS** (ESI): *m/z* = 491 $[2 \text{ x } (M–H)]^-$, 245 $[M–H]^-$; **HRMS** (ESI): calcd. for $[C_{14}H_{11}FO_3]^-$ 245.0619, found: 245.0602 $[M–H]^-$; m.p. 134 °C.

General Procedure for the Friedel-Crafts Acylation (GP3)[5]

A mixture of the appropriate **9a-f** (1.46 mmol) and $SOCl_2$ (5.29 mL, 8.68 g, 73.0 mmol) was heated for 2 h under reflux. Then $SOCl_2$ was removed under reduced pressure and the residue taken up in CH_2Cl_2 (10 mL). With ice cooling $AlCl_3$ (233 mg, 1.75 mmol) was added and the resulting black reaction mixture stirred for 16 h at room temperature. After added of ice-water (10 mL), the layers were separated, and the aqueous layer was extracted with CH_2Cl_2 (3 x 15 mL). The combined organic layers were washed with H_2O (60 mL), dried ($MgSO_4$), and the solvent was removed under reduced pressure. The residue was purified by chromatography on SiO_2 with hexanes/EtOAc (10:1) and dried under high vacuum to give fluorenones **10a**–**f** as orange solids.

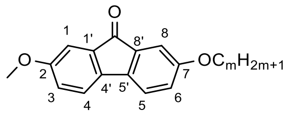

2-Methoxy-7-(octyloxy)-9*H*-fluoren-9-one (10a). According to GP3, from **9a** (520 mg, 1.46 mmol), $SOCl_2$ (5.29 mL, 8.68 g, 73.0 mmol), $AlCl_3$ (233 mg, 1.75 mmol), CH_2Cl_2 (5 mL); yield: 406 mg, 1.20 mmol, 82%. **^{1}H-NMR** (400 MHz, $CDCl_3$): δ = 0.82–0.96 (m, 3H, CH_3), 1.21–1.39 (m, 8H, CH_2), 1.39–1.52 (m, 2H, $OCH_2CH_2CH_2$), 1.70–1.86 (m, 2H, OCH_2CH_2), 3.83 (s, 3H, OCH_3), 3.91–4.03 (m, 2H, OCH_2), 6.89–6.96 (m, 2H, 3-H, 6-H), 7.11–7.18 (m, 2H, 1-H, 8-H), 7.23–7.30 (m, 2H, 4-H, 5-H) ppm; **^{13}C-NMR** (101 MHz, $CDCl_3$): δ = 14.1 (CH_3), 22.7 (CH_3CH_2), 26.0, 29.17, 29.23, 29.3 (CH_2), 31.8 ($CH_3CH_2CH_2$), 55.7 (OCH_3), 68.6 (OCH_2), 109.6 (C-8), 110.2 (C-1), 120.2 (C-5), 120.4 (C-4), 120.5 (C-6), 120.9 (C-3), 135.9 (C-8'), 136.0 (C-1'), 137.3 (C-5'), 137.6 (C-4'), 159.6 (C-2), 159.9 (C-7), 193.8 (*C*=O) ppm; **FT-IR** (ATR): $\tilde{\nu}$ = 2933 (m), 2866 (w), 1713 (m), 1614 (w), 1591 (w), 1479 (m), 1464 (vs), 1439 (m), 1388 (w), 1289 (s), 1278 (s), 1221 (m), 1212 (m), 1168 (w), 1042 (w), 1020 (w), 995 (w), 969 (w), 913 (w), 886 (w), 815 (w), 787 (m), 729 (w), 689 (w), 646 (w), 579 (w), 556 (w), 518 (w), 487 (w), 457 (w) cm^{-1}; **MS** (ESI): *m/z* = 361 $[M + Na]^+$, 339 $[M]^+$, 227; **HRMS** (ESI): calcd. for $[C_{22}H_{26}O_3Na]^+$ 361.1774, found: 361.1777 $[M+Na]^+$; m.p. 65 °C.

2-Methoxy-7-(decyloxy)-9*H*-fluoren-9-one (10b). According to GP3, from **9b** (630 mg, 1.64 mmol), $SOCl_2$ (5.95 mL, 9.76 g, 82.0 mmol), $AlCl_3$ (263 mg, 1.97 mmol), CH_2Cl_2 (10 mL); yield: 584 mg, 1.59 mmol, 97%. **^{1}H-NMR** (300 MHz, $CDCl_3$): δ = 0.81–0.96 (m, 3H, CH_3), 1.21–1.38 (m, 12H, CH_2), 1.38–1.52 (m, 2H, $OCH_2CH_2CH_2$), 1.69–1.85 (m, 2H, OCH_2CH_2), 3.83 (s, 3H, OCH_3), 3.97 (t, *J* = 6.6 Hz, 2H, OCH_2), 6.89–6.97 (m, 2H, 3-H, 6-H), 7.12–7.17 (m, 2H, 1-H, 8-H), 7.24–7.27 (m, 1H, 4-H), 7.27–7.30 (m, 1H, 5-H) ppm; **^{13}C-NMR** (75 MHz, $CDCl_3$): δ = 14.1 (CH_3), 22.7 (CH_3CH_2), 26.0, 29.2, 29.3, 29.4, 29.55, 29.57 (CH_2), 31.9 ($CH_3CH_2CH_2$), 55.7 (OCH_3), 68.6 (OCH_2), 109.6 (C-8), 110.2 (C-1), 120.2 (C-5), 120.4 (C-4), 120.5 (C-6), 120.9 (C-

3), 135.9 (C-8'), 136.0 (C-1'), 137.3 (C-5'), 137.6 (C-4'), 159.6 (C-2), 159.9 (C-7), 193.8 (*C*=O) ppm; **FT-IR** (ATR): $\tilde{\nu}$ = 3052 (w), 2918 (s), 2851 (m), 1710 (s), 1614 (w), 1591 (w), 1465 (vs), 1438 (m), 1388 (w), 1289 (s), 1278 (s), 1249 (m), 1221 (s), 1170 (w), 1135 (w), 1041 (m), 1023 (w), 999 (w), 970 (m), 931 (w), 908 (w), 888 (w), 810 (w), 786 (s), 724 (w), 689 (w), 646 (w), 577 (w), 556 (w), 515 (w) cm^{-1}; **MS** (ESI): *m/z* = 389 $[M+Na]^+$; **HRMS** (ESI): calcd. for $[C_{24}H_{30}O_3Na]^+$ 389.2087, found: 389.2092 $[M+Na]^+$; m.p. 60 °C.

2-Methoxy-7-(dodecyloxy)-9*H*-fluoren-9-one (10c). According to GP3, from **9c** (680 mg, 1.65 mmol), $SOCl_2$ (6.00 mL, 9.82 g, 82.5 mmol), $AlCl_3$ (264 mg, 1.98 mmol), CH_2Cl_2 (8 mL); yield: 636 mg, 1.61 mmol, 98%. **^{1}H-NMR** (500 MHz, $CDCl_3$): δ = 0.88 (t, *J* = 6.9 Hz, 3H, CH_3), 1.21–1.39 (m, 16H, CH_2), 1.41–1.49 (m, 2H, $OCH_2CH_2CH_2$), 1.74–1.82 (m, 2H, OCH_2CH_2), 3.84 (s, 3H, OCH_3), 3.98 (t, *J* = 6.6 Hz, 2H, OCH_2), 6.91–6.95 (m, 2H, 3-H, 6-H), 7.14–7.17 (m, 2H, 1-H, 8-H), 7.25–7.27 (m, 1H, 4-H), 7.27–7.29 (m, 1H, 5-H) ppm; **^{13}C-NMR** (126 MHz, $CDCl_3$): δ = 14.1 (CH_3), 22.7 (CH_3CH_2), 26.0, 29.2, 29.4, 29.57, 29.60, 29.64, 29.7 (CH_2), 31.9 ($CH_3CH_2CH_2$), 55.7 (OCH_3), 68.6 (OCH_2), 109.6 (C-8), 110.2 (C-1), 120.2 (C-5), 120.47 (C-4), 120.52 (C-6), 120.9 (C-3), 135.9 (C-8'), 136.0 8C-1'), 137.3 (C-5'), 137.6 (C-4'), 159.5 (C-2), 159.9 (C-7), 193.9 (*C*=O) ppm; **FT-IR** (ATR): $\tilde{\nu}$ = 3953 (w), 2915 (s), 2850 (m), 1705 (s), 1610 (m), 1597 (w), 1469 (vs), 1436 (m), 1394 (w), 1286 (s), 1270 (m), 1248 (m), 1225 (m), 1159 (w), 1135 (w), 1043 (m), 1001 (w), 974 (w), 892 (w), 847 (w), 834 (w), 816 (m), 785 (m), 718 (w), 646 (w), 576 (w), 513 (w) cm^{-1}; **MS** (EI): *m/z* = 394.3 (100) $[M]^{+\cdot}$, 226.1 (80) $[M–C_{12}H_{25}]^+$, 211.0 (20) $[M–C_{12}H_{25}–CH_3]^+$; **HRMS** (EI): calcd. for $[C_{26}H_{34}O_3]^+$ 394.2508, found: 394.2509 $[M]^+$; m.p. 75 °C.

2-Methoxy-7-(tetradecyloxy)-9*H*-fluoren-9-one (10d). According to GP3, from **9d** (1.24 g, 2.81 mmol), $SOCl_2$ (8.20 mL, 13.37 g, 112.4 mmol), $AlCl_3$ (450 mg, 3.37 mmol), CH_2Cl_2 (40 mL); yield: 1.19 g, 2.81 mmol, quant. **^{1}H-NMR** (300 MHz, $CDCl_3$): δ = 0.81–0.96 (m, 3H, CH_3), 1.19–1.38 (m, 20H, CH_2), 1.38–1.52 (m, 2H, $OCH_2CH_2CH_2$), 1.71–1.85 (m, 2H, OCH_2CH_2), 3.83 (s, 3H, OCH_3), 3.97 (t, *J* = 6.6 Hz, 2H, OCH_2), 6.90–6.93 (m, 1H, 3-H), 6.93–6.95 (m, 1H, 6-H), 7.13–7.17 (m, 2H, 1-H, 8-H), 7.24–7.27 (m, 1H 4-H), 7.27–7.30 (m, 1H, 5-H) ppm; **^{13}C-NMR** (75 MHz, $CDCl_3$): δ = 14.1 (CH_3), 22.7 (CH_3CH_2), 26.0, 29.2, 29.4, 29.57, 29.59, 29.66, 29.68, 29.70 (CH_2), 31.9 ($CH_3CH_2CH_2$), 55.7 (OCH_3), 68.6 (OCH_2), 109.6 (C-8), 110.2 (C-1), 120.2 (C-5), 120.4 (C-4), 120.5 (C-6), 120.9 (C-3), 135.9 (C-8'), 136.0 (C-1'), 137.3 (C-5'), 137.6 (C-4'), 159.6 (C-2), 159.9 (C-7), 193.8 (*C*=O) ppm; **FT-IR** (ATR): $\tilde{\nu}$ = 2953 (w), 2914 (vs), 2850 (s), 1704 (s), 1609 (m), 1597 (w), 1469 (vs), 1437 (m), 1394 (w), 1286 (s), 1271 (m), 1249 (m), 1247 (s), 1224 (s), 1159 (w), 1135 (w), 1043 (m), 1007 (w), 974 (w), 908 (w), 892 (w), 858 (w), 848 (w), 833 (w), 816 (m), 785 (s), 719 (w), 646 (w), 575 (w), 513 (w) cm^{-1}; **MS** (ESI): *m/z* = 445 $[M + Na]^+$, 423 $[M]^+$; **HRMS** (ESI): calcd. for $[C_{28}H_{38}O_3Na]^+$ 445.2713, found: 445.2709 $[M+Na]^+$; m.p. 74 °C.

2-Methoxy-7-(hexadecyloxy)-9*H*-fluoren-9-one (10e). According to GP3, from **9e** (1.73 g, 3.69 mmol), $SOCl_2$ (10.70 mL, 17.56 g, 147.6 mmol), $AlCl_3$ (591 mg, 4.43 mmol), CH_2Cl_2

(40 mL); yield: 1.62 g, 3.60 mmol, 98%. **^{1}H-NMR** (400 MHz, $CDCl_3$): δ = 0.88 (t, *J* = 6.7 Hz, 3H, C*H*$_3$), 1.20–1.39 (m, 24H, C*H*$_2$), 1.39–1.50 (m, 2H, $OCH_2CH_2CH_2$), 1.78 (m_c, 2H, OCH_2CH_2), 3.83 (s, 3H, OC*H*$_3$), 3.98 (t, *J* = 6.6 Hz, 2H, OC*H*$_2$), 6.90–6.93 (m, 1H, 3-H), 6.93–6.96 (m, 1H, 6-H), 7.13–7.18 (m, 2H, 1-H, 8-H), 7.25–7.27 (m, 1H 4-H), 7.27–7.31 (m, 1H, 5-H) ppm; **^{13}C-NMR** (101 MHz, $CDCl_3$): δ = 14.1 (*C*H$_3$), 22.7 (CH_3CH_2), 26.0, 29.2, 29.4, 29.57, 29.60, 29.67, 29.69, 29.71 (*C*H$_2$), 31.9 ($CH_3CH_2CH_2$), 55.7 (O*C*H$_3$), 68.6 (O*C*H$_2$), 109.6 (C-8), 110.2 (C-1), 120.3 (C-5), 120.45 (C-4), 120.50 (C-6), 120.9 (C-3), 135.96 (C-8'), 136.03 (C-1'), 137.3 (C-5'), 137.6 (C-4'), 159.6 (C-2), 160.0 (C-7), 193.8 (*C*=O) ppm; **FT-IR** (ATR): $\tilde{\nu}$ = 2913 (vs), 2849 (s), 1705 (s), 1609 (m), 1469 (vs), 1393 (w), 1286 (s), 1270 (m), 1248 (m), 1225 (s), 1159 (w), 1136 (w), 1043 (m), 1011 (w), 975 (w), 893 (w), 816 (m), 785 (s), 719 (w), 646 (w), 576 (w), 513 (w) cm^{-1}; **MS** (ESI): *m/z* = 473 $[M+Na]^+$, 451 $[M]^+$; **HRMS** (ESI): calcd. for $[C_{30}H_{42}O_3Na]^+$ 473.3026, found: 473.3016 $[M+Na]^+$; m.p. 78 °C.

2-Fluoro-7-methoxy-9*H*-fluoren-9-one (10f). According to GP3, from **9f** (2.53 g, 10.27 mmol), $SOCl_2$ (29.80 mL, 48.87 g, 410.8 mmol), $AlCl_3$ (1.64 g, 12.32 mmol), CH_2Cl_2 (40 mL); yield: 2.34 g, 10.27 mmol, quant. **^{1}H-NMR** (400 MHz, $CDCl_3$): δ = 3.85 (s, 3H, OC*H*$_3$), 6.97 (dd, $J_{3,4}$ = 8.2 Hz, $J_{1,3}$ = 2.5 Hz, 1H, 3-H), 7.06–7.13 (m, 1H, 6-H), 7.18 (d, $J_{1,3}$ = 2.5 Hz, 1H, 1-H), 7.25–7.29 (m, 1H, 8-H), 7.30–7.37 (m, 2H, 4-H, 5-H) ppm; **^{13}C-NMR** (101 MHz, $CDCl_3$): δ = 55.7 (O*C*H$_3$), 109.8 (C-1), 112.0 (d, *J* = 23.8 Hz, C-8), 120.5, 120.72, 120.73 (C-4, C-5), 120.8 (d, *J* = 32.3 Hz, C-6), 121.1 (C-3), 136.1 (d, *J* = 2.6 Hz, C-1'), 136.4 (d, *J* = 7.3 Hz, C-8'), 136.5 (C-4'), 140.6 (d, *J* = 3.1 Hz, C-5'), 160.6 (C-2), 162.9 (d, *J* = 248.6 Hz, C-7), 192.4 (d, *J* = 2.2 Hz, *C*=O) ppm; **^{19}F-NMR** (376 MHz, $CDCl_3$): δ = –113.7 ppm; **FT-IR** (ATR): $\tilde{\nu}$ = 1711 (s), 1606 (m), 1471 (vs), 1440 (m), 1290 (s), 1264 (s), 1221 (m), 1193 (w), 1127 (w), 1072 (w), 1038 (m), 1008 (w), 978 (w), 893 (w), 816 (m), 784 (s), 676 (w), 644 (w), 571 (w), 539 (w), 506 (w) cm^{-1}; **MS** (EI): *m/z* =228.1 (100) $[M]^+$, 213.0 (45), 185.0 (20), 157.1 (25); **HRMS** (EI): calcd. for $[C_{14}H_9 FO_2]^+$ 228.0587, found: 228.0589 $[M]^+$. The spectroscopic data were in accordance with those in the literature.[6]

General Procedure for Deprotection Using Thiols (GP4a and GP4b)[7,8]

Method A (GP4a): To a solution of 2-(diethylamino)ethanethiol hydrochloride (43 mg, 250 µmol) in abs. DMF (8 mL) under N_2 atmosphere bei 0 °C was added NaO*t*Bu (51 mg, 530 µmol), and the reaction mixture was stirred for 5 min at 0 °C and for a further 15 min at room temperature. Then the appropriate **10a**–**e** (210 µmol) was added and the reaction mixture heated for 24 h at reflux. The mixture was adjusted with 1 M HCl to pH = 1 prior to addition of EtOAc (15 mL). The layers were separated and the aqueous layer was extracted with EtOAc (3 x 50 mL). The combined organic layers were successively washed with H_2O (120 mL) and brine (120 mL), dried ($MgSO_4$) and concentrated. The residue was purified by chromatography on SiO_2 with hexanes/ EtOAc (30:1) and dried under high vacuum to give the alcohols **11a**–**e** as red solids.

Method B (GP4b): To a solution of NaH (60%ic, 432 mg, 10.78 mmol) in abs. DMF (10 mL) under N_2 atmosphere at 0 °C was added dropwise 1-dodecanethiol (10.78 mmol), and the reaction

mixture was stirred for 15 min at room temperature. Then a solution of the appropriate **10b**–**e** (4.31 mmol) in abs. DMF (10 mL) was added dropwise and the reaction mixture stirred for a further 18 h at 80 °C. The reaction mixture was then poured onto H_2O (30 mL) and EtOAc (30 mL) was added. The layers were separated and the aqueous layer was extracted with EtOAc (3 x 50 mL). The combined organic layers were washed with H_2O (120 mL) and dried ($MgSO_4$). The solvent was removed under reduced pressure and the residue purified by chromatography on SiO_2 with hexanes/EtOAc (30:1) and dried under high vacuum.

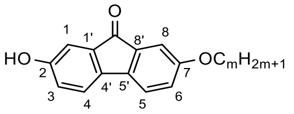

2-Hydroxy-7-(octyloxy)-9*H*-fluoren-9-one (11a). According to GP4a, from **10a** (70 mg, 0.21 mmol), 2-(diethylamino)ethanethiol·HCl (43 mg, 0.25 mmol), NaO*t*Bu (51 mg, 0.53 mmol), abs. DMF (5 mL); yield: 50 mg, 0.15 mmol, 73%. **^{1}H-NMR** (700 MHz, $CDCl_3$): δ = 0.89 (t, *J* = 7.0 Hz, 3H, CH_3), 1.24–1.38 (m, 8H, CH_2), 1.41–1.48 (m, 2H, $OCH_2CH_2CH_2$), 1.74–1.81 (m, 2H, OCH_2CH_2), 3.97 (t, *J* = 6.6 Hz, 2H, OCH_2), 5.60–5.82 (m, 1H, O*H*), 6.89 (dd, $J_{3,4}$ = 8.0 Hz, $J_{1,3}$ = 2.5 Hz, 1H, 3-H), 6.92 (dd, $J_{5,6}$ = 8.1 Hz, $J_{6,8}$ = 2.5 Hz, 1H, 6-H), 7.11 (d, $J_{1,3}$ = 2.5 Hz, 1H, 1-H), 7.13 (d, $J_{6,8}$ = 2.5 Hz, 1H, 8-H), 7.23 (d, $J_{3,4}$ = 8.0 Hz, 1H, 4-H), 7.25 (d, $J_{5,6}$ = 8.1 Hz, 1H, 5-H) ppm; **^{13}C-NMR** (176 MHz, $CDCl_3$): δ = 14.1 (CH_3), 22.7 (CH_3CH_2), 26.0, 29.17, 29.23, 29.3 (CH_2), 31.8 ($CH_3CH_2CH_2$), 68.6 (OCH_2), 110.3 (C-8), 112.0 (C-1), 120.5 (C-5), 120.7 (C-4), 121.0 (C-6), 121.1 (C-3), 135.8 (C-8'), 136.1 (C-1'), 137.4 (C-5'), 137.6 (C-4'), 156.1 (C-2), 159.6 (C-7), 194.1 (*C*=O) ppm; **FT-IR** (ATR): $\tilde{\nu}$ = 3407 (w), 2954 (m), 2918 (s), 2853 (m), 1715 (m), 1699 (vs), 1683 (m), 1610 (m), 1467 (s), 1413 (m), 1393 (w), 1291 (s), 1265 (w), 1238 (m), 1227 (m), 1167 (w), 1136 (w), 1080 (w), 1043 (w), 999 (w), 979 (w), 896 (w), 823 (m), 798 (m), 785 (m), 718 (w), 700 (w), 647 (w), 568 (w), 511 (w), 429 (w) cm^{-1}; **MS** (EI): *m/z* = 324.2 (35) $[M]^+$, 212.0 (100) $[M–C_8H_{17}]^+$; **HRMS** (EI): calcd. for $[C_{21}H_{24}O_3]^+$ 324.1725, found: 324.1724 $[M]^+$; m.p. 101 °C.

2-Hydroxy-7-(decyloxy)-9*H*-fluoren-9-one (11b). According to GP4b, from **10b** (1.60 g, 4.36 mmol), 1-dodecanethiol (2.62 mL, 2.21 mg, 10.90 mmol), NaH (60%ic, 436 mg, 10.90 mmol), abs. DMF (15 mL); yield: 1.28 g, 3.63 mmol, 83%. **^{1}H-NMR** (300 MHz, $CDCl_3$): δ = 0.82–0.96 (m, 3H, CH_3), 1.21–1.38 (m, 12H, CH_2), 1.38–1.52 (m, 2H, $OCH_2CH_2CH_2$), 1.77 (m_c, 2H, OCH_2CH_2), 3.97 (t, *J* = 6.6 Hz, 2H, OCH_2), 5.43 (s, 1H, O*H*), 6.86–6.90 (m, 1H, 3-H), 6.92 (dd, $J_{5,6}$ = 8.2 Hz, $J_{6,8}$ = 2.5 Hz, 1H, 6-H), 7.09–7.12 (m, 1H, 1-H), 7.14 (d, $J_{6,8}$ = 2.5 Hz, 1H, 8-H), 7.21–7.28 (m, 2H, 4-H, 5-H) ppm; **^{13}C-NMR** (75 MHz, $CDCl_3$): δ = 14.1 (CH_3), 22.7 (CH_3CH_2), 26.0, 29.2, 29.3, 29.4, 29.6 (CH_2), 31.9 ($CH_3CH_2CH_2$), 68.6 (OCH_2), 110.3 (C-8), 112.0 (C-1), 120.5 (C-5), 120.7 (C-4), 120.9 (C-6), 121.1 (C-3), 135.8 (C-8'), 136.2 (C-1'), 137.3 (C-5'), 137.6 (C-4'), 156.0 (C-2), 159.6 (C-7), 194.0 (*C*=O) ppm; **FT-IR** (ATR): $\tilde{\nu}$ = 3402 (m), 2922 (vs), 2853 (m), 1703 (s), 1612 (m), 1466 (vs), 1378 (w), 1318 (w), 1287 (m), 1238 (s), 1133 (w), 1077 (w), 1037 (w), 1010 (w), 980 (w), 885 (w), 816 (m), 798 (m), 785 (m), 722 (w), 696 (w), 646 (w), 568

(w), 512 (w), 431 (w) cm^{-1}; **MS** (ESI): *m/z* = 351 $[M]^+$; **HRMS** (ESI): calcd. for $[C_{23}H_{28}O_3]^+$ 351.1966, found: 351.1963 $[M]^+$; m.p. 102 °C.

2-Hydroxy-7-(dodecyloxy)-9*H*-fluoren-9-one (11c). According to GP4b, from **10c** (1.70 g, 4.31 mmol), 1-dodecanethiol (2.58 mL, 2.18 g, 10.78 mmol), NaH (60%ic, 432 mg, 10.78 mmol), abs. DMF (10 mL); yield: 1.38 g, 3.63 mmol, 84%. **^{1}H-NMR** (300 MHz, $CDCl_3$): δ = 0.78–0.99 (m, 3H, CH_3), 1.16–1.53 (m, 18H, CH_2), 1.69–1.87 (m, 2H, OCH_2CH_2), 3.97 (t, *J* = 6.6 Hz, 2H, OCH_2), 5.31 (s, 1H, O*H*), 6.89 (dd, $J_{3,4}$ = 8.0 Hz, $J_{1,3}$ = 2.5 Hz, 1H, 3-H), 6.93 (dd, $J_{5,6}$ = 8.2 Hz, $J_{6,8}$ = 2.5 Hz, 1H, 6-H), 7.10 (d, $J_{1,3}$ = 2.5 Hz, 1H, 1-H), 7.14 (d, $J_{6,8}$ = 2.5 Hz, 1H, 8-H), 7.22–7.25 (m, 1H, 4-H), 7.25–7.29 (m, 1H, 5-H) ppm; **^{13}C-NMR** (75 MHz, $CDCl_3$): δ = 14.1 (CH_3), 22.7 (CH_3CH_2), 26.0, 29.2, 29.4, 29.57, 29.59, 29.64, 29.7 (CH_2), 31.9 ($CH_3CH_2CH_2$), 68.6 (OCH_2), 110.2 (C-8), 112.0 (C-1), 120.5 (C-5), 120.6 (C-4), 120.9 (C-6), 121.0 (C-3), 135.8 (C-8'), 136.2 (C-1'), 137.3 (C-5'), 137.7 (C-4'), 155.9 (C-2), 159.6 (C-7), 193.9 (*C*=O) ppm; **FT-IR** (ATR): $\tilde{\nu}$ = 3346 (w), 2954 (w), 2916 (vs), 2851 (s), 1715 (m), 1694 (s), 1612 (m), 1460 (vs), 1394 (w), 1371 (w), 1289 (s), 1240 (s), 1227 (s), 1136 (w), 1077 (w), 1037 (m), 1002 (w), 981 (w), 891 (w), 824 (m), 798 (w), 785 (m), 718 (w), 647 (w), 568 (w), 513 (w), 479 (w), 420 (w) cm^{-1}; **MS** (ESI): *m/z* = 379 $[M]^+$; **HRMS** (ESI): calcd. for $[C_{25}H_{32}O_3]^+$ 379.2279, found: 379.2281 $[M]^+$; m.p. 104 °C.

2-Hydroxy-7-(tetradecyloxy)-9*H*-fluoren-9-one (11d). According to GP4b, from **10d** (425 mg, 1.01 mmol), 1-dodecanethiol (0.61 mL, 512 mg, 2.53 mmol), NaH (60%ic, 101 mg, 2.53 mmol), abs. DMF (9 mL); yield: 361 mg, 0.88 mmol, 87%. **^{1}H-NMR** (300 MHz, $CDCl_3$): δ = 0.81–0.96 (m, 3H, CH_3), 1.19–1.38 (m, 20H, CH_2), 1.38–1.53 (m, 2H, $OCH_2CH_2CH_2$), 1.71–1.87 (m, 2H, OCH_2CH_2), 3.97 (t, *J* = 6.6 Hz, 2H, OCH_2), 5.30 (s, 1H, O*H*), 6.89 (dd, $J_{3,4}$ = 8.0 Hz, $J_{1,3}$ = 2.5 Hz, 1H, 3-H), 6.93 (dd, $J_{5,6}$ = 8.2 Hz, $J_{6,8}$ = 2.5 Hz, 1H, 6-H), 7.10 (d, $J_{1,3}$ = 2.5 Hz, 1H, 1-H), 7.14 (d, $J_{6,8}$ = 2.5 Hz, 1H, 8-H), 7.22–7.30 (m, 2H, 4-H, 5-H) ppm, **^{13}C-NMR** (75 MHz, $CDCl_3$): δ = 14.1 (CH_3), 22.7 (CH_3CH_2), 26.0, 29.2, 29.4, 29.56, 29.59, 29.66, 29.69 (CH_2), 31.9 ($CH_3CH_2CH_2$), 68.6 (OCH_2), 110.2 (C-8), 112.0 (C-1), 120.5 (C-5), 120.6 (C-4), 120.9 (C-6), 121.1 (C-3), 135.8 (C-8'), 136.2 (C-1'), 137.3 (C-5'), 137.7 (C-4'), 155.9 (C-2), 159.6 (C-7), 193.9 (*C*=O) ppm; **FT-IR** (ATR): $\tilde{\nu}$ = 3358 (w), 2917 (vs), 2851 (s), 1715 (m), 1694 (m), 1613 (m), 1468 (s), 1395 (w), 1290 (m), 1241 (s), 1228 (m), 1136 (w), 1079 (w), 1041 (w), 1010 (w), 978 (w), 891 (w), 826 (w), 799 (m), 785 (m), 719 (w), 514 (w) cm^{-1}; **MS** (ESI): *m/z* = 431 $[M+Na]^+$, 409 $[M]^+$; **HRMS** (ESI): calcd. for $[C_{27}H_{36}O_3Na]^+$ 431.2557, found: 431.2553 $[M+Na]^+$; m.p. 106 °C.

2-Hydroxy-7-(hexadecyloxy)-9*H*-fluoren-9-one (11e). According to GP4b, from **10e** (2.15 g, 4.77 mmol), 1-Dodecanthiol (2.85 mL, 2.41 g, 11.93 mmol), NaH (60%ic, 477 mg, 11.93 mmol), abs. DMF (10 mL); yield: 1.89 g, 4.33 mmol, 91%. **^{1}H-NMR** (400 MHz, $CDCl_3$): δ = 0.81–0.96 (m, 3H, CH_3), 1.19–1.39 (m, 24H, CH_2), 1.39–1.53 (m, 2H, $OCH_2CH_2CH_2$), 1.71–1.86 (m, 2H, OCH_2CH_2), 3.97 (t, *J* = 6.6 Hz, 2H, OCH_2), 5.17 (s, 1H, O*H*), 6.88 (dd, $J_{3,4}$ = 8.0 Hz, $J_{1,3}$ = 2.5 Hz, 1H, 3-H), 6.93 (dd, $J_{5,6}$ = 8.1 Hz, $J_{6,8}$ = 2.5 Hz, 1H, 6-H), 7.09 (d, $J_{1,3}$ = 2.5 Hz, 1H, 1-H), 7.14 (d, $J_{6,8}$ = 2.5 Hz, 1H, 8-H), 7.22–7.26 (m, 1H, 4-H), 7.26–7.28 (m, 1H, 5-H) ppm; **^{13}C-**

NMR (101 MHz, $CDCl_3$): δ = 14.1 (CH_3), 22.7 (CH_3CH_2), 26.0, 29.2, 29.4, 29.57, 29.60, 29.67, 29.69, 29.71 (CH_2), 31.9 ($CH_3CH_2CH_2$), 68.6 (OCH_2), 110.3 (C-8), 112.0 (C-1), 120.5 (C-5), 120.6 (C-4), 120.9 (C-6), 121.0 (C-3), 135.8 (C-8'), 136.2 (C-1'), 137.3 (C-5'), 137.7 (C-4'), 155.9 (C-2), 159.6 (C-7), 193.8 (*C*=O) ppm; **FT-IR** (ATR): $\tilde{\nu}$ = 3345 (w), 2916 (vs), 2850 (s), 1697 (s), 1610 (m), 1470 (v), 1413 (w), 1290 (m), 1241 (m), 1227 (m), 1137 (w), 1036 (w), 896 (w), 823 (m), 798 (m), 785 (m), 718 (w), 595 (w), 511 (w), 467 (w), 433 (w) cm^{-1}; **MS** (ESI): *m/z* = 459 $[M+Na]^+$, 437 $[M]^+$; **HRMS** (ESI): calcd. for $[C_{29}H_{40}O_3Na]^+$ 459.2870, found: 459.2882 $[M+Na]^+$; m.p. 103 °C.

General Procedure for the Preparation of Thioethers 12, 13 (GP5)[9]

To a solution of NaH (60%ic, 127 mg, 3.18 mmol) in abs. DMF (5 mL) under N_2 atmosphere at 0 °C was added dropwise the appropriate 1-alkanethiol (3.18 mmol). After stirring for 15 min at room temperature, a solution of **10f** (290 mg, 1.27 mmol) in abs. DMF (10 mL) was added dropwise, and the reaction mixture stirred for 18 h at 80 °C. The reaction mixture was then poured onto H_2O (20 mL) and EtOAc (20 mL) was added. The layers were separated and the aqueous layer was extracted with EtOAc (3 x 30 mL). The combined organic layers were washed with H_2O (100 mL) and dried ($MgSO_4$). The solvent was removed under reduced pressure and the residue purified by chromatography on SiO_2 with hexanes/EtOAc (30:1) and dried under high vacuum to give **12** and **13**.

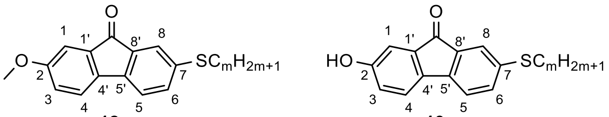

2-(Decylthio)-7-methoxy-9*H*-fluoren-9-one (12b) and 2-(decylthio)-7-hydroxy-9*H*-fluoren-9-one (13b). According to GP5, from **10f** (290 mg, 1.27 mmol), 1-decanethiol (0.65 mL, 554 mg, 3.18 mmol), NaH (60%ic, 127 mg, 3.18 mmol), abs. DMF (6 mL); yield: **12b** (39 mg, 102 µmol, 8%), orange solid, **13b** (356 mg, 970 µmol, 76%), red solid.

12b: **^{1}H-NMR** (300 MHz, $CDCl_3$): δ = 0.81–0.95 (m, 3H, CH_3), 1.16–1.50 (m, 14H, CH_2), 1.59–1.73 (m, 2H, SCH_2CH_2), 2.89–2.98 (m, 2H, SCH_2), 3.85 (s, 3H, OCH_3), 6.97 (dd, $J_{3,4}$ = 8.2 Hz, $J_{1,3}$ = 2.5 Hz, 1H, 3-H), 7.17–7.19 (m, 1H, 1-H), 7.27–7.31 (m, 1H, 5-H), 7.33–7.39 (m, 2H, 4-H, 6-H), 7.52–7.55 (m, 1H, 8-H) ppm; **^{13}C-NMR** (75 MHz, $CDCl_3$): δ = 14.1 (CH_3), 22.7 (CH_3CH_2), 28.8, 29.0, 29.1, 29.3, 29.49, 29.53 (CH_2), 31.9 ($CH_3CH_2CH_2$), 33.7 ($BrCH_2$), 55.7 (OCH_3), 109.5 (C-1), 119.8 (C-5), 120.4 (C-3), 121.2 (C-4), 124.3 (C-8), 134.8 (C-6), 135.0 (C-8'), 135.7 (C-1'), 136.9 (C-4'), 137.5 (C-7), 142.2 (C-5'), 160.7 (C-2), 193.5 (*C*=O) ppm; **FT-IR** (ATR): $\tilde{\nu}$ = 3060 (w), 2954 (m), 2919 (vs), 2871 (w), 2851 (m), 1720 (s), 1712 (s), 1598 (m), 1487 (m), 1469 (m), 1434 (m), 1349 (w), 1285 (m), 1260 (m), 1230 (m), 1145 (w), 1101 (w), 1033 (m), 1005 (w), 887 (w), 837 (w), 820 (m), 781 (m), 746 (w), 718 (w), 635 (w), 500 (m) cm^{-1}; **MS** (ESI): *m/z* = 787 $[2 M+Na]^+$, 405 $[M+Na]^+$; **HRMS** (ESI): calcd. for $[C_{24}H_{30}O_2SNa]^+$ 405.1859, found: 405.1852 $[M+Na]^+$; m.p. 72 °C.

13b: **^{1}H-NMR** (300 MHz, $CDCl_3$): δ = 0.83–0.91 (m, 3H, C*H*$_3$), 1.18–1.35 (m, 12H, C*H*$_2$), 1.35–1.48 (m, 2H, SCH_2CH_2C*H*$_2$), 1.58–1.71 (m, 2H, SCH_2C*H*$_2$), 2.88–2.98 (m, 2H, SC*H*$_2$), 5.92 (s, 1H, O*H*), 6.93 (dd, $J_{3,4}$ = 8.1 Hz, $J_{1,3}$ = 2.5 Hz, 1H, 3-H), 7.13–7.16 (m, 1H, 1-H), 7.23–7.37 (m, 3H, 4-H, 5-H, 6-H), 7.50–7.53 (m, 1H, 8-H) ppm; **^{13}C-NMR** (75 MHz, $CDCl_3$): δ = 14.1 (*C*H$_3$), 22.7 (CH_3*C*H$_2$), 28.8, 29.0, 29.2, 29.3, 29.50, 29.54 (*C*H$_2$), 31.9 (CH_3CH_2*C*H$_2$), 33.7 (S*C*H$_2$), 112.0 (C-1), 119.8 (C-5), 121.2 (C-3), 121.4 (C-4), 124.4 (C-8), 134.8 (C-6), 134.9 (C-8'), 135.8 (C-1'), 136.9 (C-4'), 137.6 (C-7), 142.2 (C-5'), 156.9 (C-2), 194.0 (*C*=O) ppm; **FT-IR** (ATR): $\tilde{\nu}$ = 3406 (m), 3339 (m), 2955 (m), 2917 (vs), 2874 (w), 2852 (m), 1733 (w), 1712 (s), 1703 (vs), 1598 (m), 1489 (m), 1470 (m), 1456 (m), 1368 (w), 1289 (m), 1252 (m), 1187 (w), 1136 (w), 1092 (w), 1059 (w), 887 (w), 824 (m), 783 (m), 753 (m), 716 (w), 496 (m) cm^{-1}; **MS** (ESI): *m/z* = 735 $[2\ M–2\ H]^-$, 367 $[M–H]^-$; **HRMS** (ESI): calcd. for $[C_{23}H_{28}O_2S]^-$ 367.1737, found: 367.1740 $[M–H]^-$; m.p. 109 °C.

2-(Dodecylthio)-7-methoxy-9*H*-fluoren-9-one (12c) and 2-(dodecylthio)-7-hydroxy-9*H*-fluoren-9-one (13c). According to GP5, from **10f** (900 mg, 3.94 mmol), 1-dodecanethiol (2.64 mL, 2.23 g, 11.03 mmol), NaH (60%ic, 442 mg, 11.03 mmol), abs. DMF (10 mL); yield: **12c** (98 mg, 239 µmol, 6%), orange solid, **13c** (1.28 g, 3.22 mmol, 82%), red solid.

12c: **^{1}H-NMR** (300 MHz, $CDCl_3$): δ = 0.83–0.92 (m, 3H, C*H*$_3$), 1.17–1.36 (m, 16H, C*H*$_2$), 1.36–1.49 (m, 2H, SCH_2CH_2C*H*$_2$), 1.59–1.72 (m, 2H, SCH_2C*H*$_2$), 2.90–2.98 (m, 2H, SC*H*$_2$), 3.85 (s, 3H, OC*H*$_3$), 6.97 (dd, $J_{3,4}$ = 8.2 Hz, $J_{1,3}$ = 2.5 Hz, 1H, 3-H), 7.17–7.20 (m, 1H, 1-H), 7.27–7.32 (m, 1H, 5-H), 7.33–7.40 (m, 2H, 4-H, 6-H), 7.52–7.56 (m, 1H, 8-H) ppm; **^{13}C-NMR** (75 MHz, $CDCl_3$): δ = 14.1 (*C*H$_3$), 22.7 (CH_3*C*H$_2$), 28.8, 29.0, 29.2, 29.3, 29.5, 29.58, 29.63 (*C*H$_2$), 31.9 (CH_3CH_2*C*H$_2$), 33.7 (Br*C*H$_2$), 55.7 (O*C*H$_3$), 109.5 (C-1), 119.8 (C-5), 120.5 (C-3), 121.2 (C-4), 124.3 (C-8), 134.8 (C-6), 135.0 (C-8'), 135.7 (C-1'), 136.9 (C-4'), 137.5 (C-7), 142.2 (C-5'), 160.7 (C-2), 193.5 (*C*=O) ppm; **FT-IR** (ATR): $\tilde{\nu}$ = 2954 (m), 2915 (vs), 2873 (m), 2851 (s), 1707 (s), 1604 (m), 1492 (m), 1470 (m), 1426 (m), 1348 (w), 1297 (m), 1280 (m), 1251 (m), 1228 (m), 1190 (w), 1126 (w), 1104 (w), 1060 (w), 1034 (m), 1008 (w), 972 (w), 888 (w), 815 (m), 781 (m), 742 (m), 716 (w), 498 (m) cm^{-1}; **MS** (ESI): *m/z* = 433 $[M+Na]^+$, 410 $[M]^+$, 393; **HRMS** (ESI): calcd. for $[C_{26}H_{34}O_2SNa]^+$ 433.2172, found: 433.2174 $[M+Na]^+$; m.p. 81 °C.

13c: **^{1}H-NMR** (400 MHz, Aceton-d$_6$): δ = 0.87 (t, *J* = 6.7 Hz, 3H, C*H*$_3$), 1.21–1.38 (m, 16H, C*H*$_2$), 1.41–1.52 (m, 2H, SCH_2CH_2C*H*$_2$), 1.66 (m_c, 2H, SCH_2C*H*$_2$), 3.02 (t, *J* = 7.3 Hz, 2H, SC*H*$_2$), 7.00 (dd, $J_{3,4}$ = 8.1 Hz, $J_{1,3}$ = 2.4 Hz, 1H, 3-H), 7.06 (d, $J_{1,3}$ = 2.4 Hz, 1H, 1-H), 7.44–7.48 (m, 2H, 5-H, 6-H), 7.49–7.54 (m, 2H, 4-H, 8-H), 8.98 (br s, 1H, O*H*) ppm; **^{13}C-NMR** (101 MHz, Aceton-d$_6$): δ = 15.2 (*C*H$_3$), 24.2, 33.5 (*C*H$_2$), 34.8 (S*C*H$_2$), 112.9 (C-1), 121.9 (C-5), 122.8 (C-3), 123.6 (C-4), 125.4 (C-8), 136.56 (C-6), 136.58 (C-8'), 137.3 (C-1'), 137.5 (C-4'), 138.9 (C-7), 144.3 (C-5'), 160.6 (C-2), 194.4 (*C*=O) ppm (further CH_2 signals are not visible due to solvent peaks); **FT-IR** (ATR): $\tilde{\nu}$ = 3370 (m), 2954 (m), 2918 (vs), 2851 (s), 1718 (s), 1698 (s), 1593 (m), 1484 (m), 1460 (m), 1397 (w), 1290 (m), 1254 (m), 1221 (w), 1185 (w), 1140 (w), 898 (w), 828 (m), 783 (m), 755 (w), 721 (w), 499 (m) cm^{-1}; **MS** (ESI): *m/z* = 791 $[2\ M–2\ H]^-$, 395 $[M–H]^-$; **HRMS** (ESI): calcd. for $[C_{25}H_{32}O_2S]^-$ 395.2050, found: 395.2041 $[M–H]^-$; m.p. 107 °C.

2-(Tetradecylthio)-7-methoxy-9*H*-fluoren-9-one (13d) and 2-(tetradecylthio)-7-hydroxy-9*H*-fluoren-9-one (13d). According to GP5, from **10f** (500 mg, 2.19 mmol), 1-tetradecanethiol (1.18 mL, 1.01 g, 4.38 mmol), NaH (60%ic, 175 mg, 4.38 mmol), abs. DMF (5 mL); yield: **12d** (222 mg, 506 µmol, 23%), orange solid, **13d** (589 mg, 1.39 mmol, 63%), red solid.

12d: **^{1}H-NMR** (400 MHz, $CDCl_3$): δ = 0.88 (t, *J* = 6.8 Hz, 3H, CH_3), 1.20–1.35 (m, 20H, CH_2), 1.37–1.47 (m, 2H, $SCH_2CH_2CH_2$), 1.65 (m_c, 2H, SCH_2CH_2), 2.94 (t, *J* = 7.4 Hz, 2H, SCH_2), 3.85 (s, 3H, OCH_3), 6.97 (dd, $J_{3,4}$ = 8.2 Hz, $J_{1,3}$ = 2.5 Hz, 1H, 3-H), 7.18 (d, $J_{1,3}$ = 2.5 Hz, 1H, 1-H), 7.29 (d, $J_{5,6}$ = 7.8 Hz, 1H, 5-H), 7.33–7.39 (m, 2H, 4-H, 6-H), 7.52–7.56 (m, 1H, 8-H) ppm; **^{13}C-NMR** (101 MHz, $CDCl_3$): δ = 14.1 (CH_3), 22.7 (CH_3CH_2), 28.8, 29.0, 29.2, 29.4, 29.5, 29.6, 29.65, 29.67, 29.69, 31.9 ($CH_3CH_2CH_2$), 33.7 (SCH_2), 55.8 (OCH_3), 109.5 (C-1), 119.8 (C-5), 120.5 (C-3), 121.2 (C-4), 124.4 (C-8), 134.8 (C-6), 135.0 (C-8'), 135.8 (C-1'), 136.9 (C-4'), 137.5 (C-7), 142.2 (C-5'), 160.7 (C-2), 193.5 (*C*=O) ppm; **FT-IR** (ATR): $\tilde{\nu}$ = 2954 (w), 2917 (vs), 2850 (s), 1710 (s), 1619 (w), 1597 (m), 1486 (m), 1469 (m), 1435 (m), 1348 (w), 1284 (m), 1258 (m), 1229 (m), 1193 (w), 1144 (w), 1101 (w), 1033 (m), 1005 (w), 972 (w), 887 (w), 820 (m), 781 (m), 745 (m), 717 (w), 500 (m) cm^{-1}; **MS** (ESI): *m/z* = 461 $[M+Na]^+$, 447, 439 $[M]^+$, 393; **HRMS** (ESI): calcd. for $[C_{28}H_{38}O_2SNa]^+$ 461.2485, found: 461.2450 $[M+Na]^+$; m.p. 86 °C.

13d: **^{1}H-NMR** (400 MHz, Aceton-d_6): δ = 0.87 (t, *J* = 6.6 Hz, 3H, CH_3), 1.21–1.38 (m, 20H, CH_2), 1.41–1.51 (m, 2H, $SCH_2CH_2CH_2$), 1.66 (m_c, 2H, SCH_2CH_2), 3.02 (t, *J* = 7.3 Hz, 2H, SCH_2), 7.00 (dd, $J_{3,4}$ = 8.1 Hz, $J_{1,3}$ = 2.4 Hz, 1H, 3-H), 7.06 (d, $J_{1,3}$ = 2.4 Hz, 1H, 1-H), 7.45–7.49 (m, 2H, 5-H, 6-H), 7.49–7.55 (m, 2H, 4-H, 8-H), 9.02 (br s, 1H, O*H*) ppm; **^{13}C-NMR** (101 MHz, Aceton-d_6): δ = 15.2 (CH_3), 24.2, 33.5 (CH_2), 34.8 (SCH_2), 112.9 (C-1), 121.9 (C-5), 122.8 (C-3), 123.6 (C-4), 125.5 (C-8), 136.56 (C-8), 136.61 (C-8'), 137.3 (C-1'), 137.5 (C-4'), 138.9 (C-7), 144.3 (C-5'), 160.6 (C-2), 194.4 (*C*=O) ppm (further CH_2 signals are not visible due to solvent peaks); **FT-IR** (ATR): $\tilde{\nu}$ = 3284 (w), 2917 (s), 2850 (m), 1715 (vs), 1599 (m), 1488 (w), 1471 (m), 1456 (m), 1375 (w), 1292 (w), 1252 (m), 885 (w), 831 (m), 781 (m), 755 (w), 716 (w), 500 (m) cm^{-1}; **MS** (ESI): *m/z* = 847 $[2\ M–2\ H]^-$, 459, 423 $[M–H]^-$; **HRMS** (ESI): calcd. for $[C_{27}H_{36}O_2S]^-$ 423.2363, found: 423.2360 $[M–H]^-$; m.p. 109 °C.

General Procedure for the Esterification with Dibromoalkanes (GP6)[10]

A solution of the appropriate fluorenone **11** or **13** (0.57 mmol), the respective dibromide (1.71 mmol) and K_2CO_3 (236 mg, 1.71 mmol) in MeCN (30 mL) were heated at reflux overnight. The reaction mixture was then poured onto H_2O (50 mL) and CH_2Cl_2 (50 mL) was added. The layers were separated and the aqueous layer was extracted with CH_2Cl_2 (2 x 50 mL). The combined organic layers were washed with H_2O (100 mL) and dried ($MgSO_4$). The solvent was removed under reduced pressure and the residue was purified by chromatography on SiO_2 with hexanes/EtOAc (30:1) and dried under high vacuum to give **Br(On,Ym)** as orange solids.

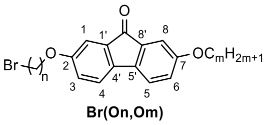

Br(On,Om)

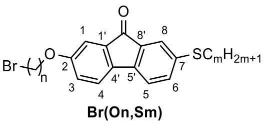

Br(On,Sm)

2-(4-Bromobutoxy)-7-(dodecyloxy)-9*H*-fluoren-9-one [Br(O4,O12)]. According to GP6, from **11c** (130 mg, 342 µmol), 1,4-dibromobutane (0.12 mL, 222 mg, 1.03 mmol), K_2CO_3 (142 mg, 1.03 mmol), MeCN (25 mL); yield: 131 mg, 254 µmol, 74%. **^{1}H-NMR** (500 MHz, $CDCl_3$): δ = 0.88 (t, *J* = 6.7 Hz, 3H, C*H*$_3$), 1.19–1.40 (m, 16H, C*H*$_2$), 1.40–1.52 (m, 2H, $OCH_2CH_2CH_2$), 1.78 (m_c, 2H, OCH_2CH_2), 1.90–2.01 (m, 2H, $BrCH_2CH_2CH_2$), 2.07 (m_c, 2H, $BrCH_2CH_2$), 3.49 (t, *J* = 6.8 Hz, 2H, $BrCH_2$), 3.97 (t, *J* = 6.8 Hz, 2H, OCH_2), 4.02 (t, *J* = 6.1 Hz, 2H, $BrCH_2CH_2CH_2CH_2O$), 6.89–6.95 (m, 2H, 3-H, 6-H), 7.12–7.16 (m, 2H, 1-H, 8-H), 7.24–7.30 (m, 2H, 4-H, 5-H) ppm; **^{13}C-NMR** (126 MHz, $CDCl_3$): δ = 14.1 (CH_3), 22.7 (CH_3CH_2), 26.0, 27.8, 29.2, 29.36, 29.38, 29.57, 29.59, 29.64, 29.7 (CH_2), 31.9 ($CH_3CH_2CH_2$), 33.3 ($BrCH_2$), 67.4 ($BrCH_2CH_2CH_2CH_2O$), 68.6 (OCH_2), 110.1, 110.2 (C-1, C-8), 120.48, 120.51 (C-4, C-5), 120.7, 120.9 (C-3, C-6), 135.9, 136.0 (C-1', C-8'), 137.2, 137.6 (C-4', C-5'), 159.1 (C-2), 159.6 (C-7), 193.8 (*C*=O) ppm; **FT-IR** (ATR): $\tilde{\nu}$ = 3051 (w), 2917 (s), 2850 (m), 1714 (s), 1607 (w), 1591 (w), 1462 (vs), 1403 (m), 1288 (vs), 1268 (m), 1249 (m), 1221 (s), 1141 (w), 1046 (m), 998 (w), 972 (w), 938 (w), 891 (w), 812 (m), 798 (m), 784 (m), 751 (w), 735 (w), 720 (w), 647 (w), 597 (w), 557 (w), 516 (w) cm^{-1}; **MS** (ESI): *m/z* = 539 $[M+Na]^+$, 515 $[M]^+$; **HRMS** (ESI): calcd. for $[C_{29}H_{39}BrO_3Na]^+$ 537.1975, gef. 537.1989 $[M+Na]^+$; **elemental analysis**: calcd. (%) for $C_{29}H_{39}BrO_3$: C 67.56, H 7.63, found: C 67.66, H 7.88; **DSC**: Cr 71 [26.2 kJ mol^{-1}] SmA 74 [1.6 kJ mol^{-1}] I (3. H); I 73 [–4.0 kJ mol^{-1}] SmA 43 [–26.6 kJ mol^{-1}] Cr (3. C).

2-(4-Bromobutoxy)-7-(tetradecyloxy)-9*H*-fluoren-9-one [Br(O4,O14)]. According to GP6, from **11d** (250 mg, 612 µmol), 1,4-dibromobutane (0.22 mL, 397 mg, 1.84 mmol), K_2CO_3 (254 mg, 1.84 mmol), MeCN (50 mL); yield: 248 mg, 456 µmol, 75%. **^{1}H-NMR** (400 MHz, $CDCl_3$): δ = 0.88 (t, *J* = 6.7 Hz, 3H, C*H*$_3$), 1.20–1.40 (m, 22H, C*H*$_2$), 1.40–1.50 (m, 2H, $OCH_2CH_2CH_2$), 1.72–1.83 (m, 2H, OCH_2CH_2), 1.90–2.01 (m, 2H, $BrCH_2CH_2CH_2$), 2.01–2.13 (m, 2H, $BrCH_2CH_2$), 3.49 (t, *J* = 6.6 Hz, 2H, $BrCH_2$), 3.98 (t, *J* = 6.6 Hz, 2H, OCH_2), 4.02 (t, *J* = 6.0 Hz, 2H, $BrCH_2CH_2CH_2CH_2O$), 6.89–6.95 (m, 2H, 3-H, 6-H), 7.12–7.17 (m, 2H, 1-H, 8-H), 7.24–7.30 (m, 2H, 4-H, 5-H) ppm; **^{13}C-NMR** (101 MHz, $CDCl_3$): δ = 14.1 (CH_3), 22.7 (CH_3CH_2), 26.0, 27.8, 29.2, 29.37, 29.41, 29.57, 29.60, 29.66, 29.68, 29.70 (CH_2), 31.9 ($CH_3CH_2CH_2$), 33.3 ($BrCH_2$), 67.4 ($BrCH_2CH_2CH_2CH_2O$), 68.6 (OCH_2), 110.19, 110.24 (C-1, C-8), 120.48, 120.52 (C-4, C-5), 120.8, 120.9 (C-3, C-6), 135.96, 136.02 (C-1', C-8'), 137.3, 137.7 (C-4', C-5'), 159.2 (C-2), 159.6 (C-7), 193.8 (*C*=O) ppm; **FT-IR** (ATR): $\tilde{\nu}$ = 2950 (w), 1721 (w), 1658 (s), 1622 (w), 1525 (s), 1467 (w), 1415 (m), 1397 (m), 1367 (m), 1348 (w), 1326 (m), 1203 (s), 1151 (vs), 1084 (m), 1052 (m), 1029 (w), 970 (w), 916 (w), 789 (m), 753 (m), 728 (m), 645 (w), 556 (w), 528 (w) cm^{-1}; **MS** (ESI): *m/z* = 565 $[M+Na]^+$, 543 $[M]^+$; **HRMS** (ESI): calcd. for $[C_{31}H_{43}BrO_3Na]^+$ 565.2288, found: 565.2299 $[M+Na]^+$; **elemental analysis**: calcd. (%) for $C_{31}H_{43}BrO_3$: C 68.50, H 7.97, found: C 68.63, H 7.99; **DSC**: Cr 74 [45.0 kJ mol^{-1}] I (3. H); I 73 [–2.6 kJ mol^{-1}] SmA 55 [–37.2 kJ mol^{-1}] Cr (3. C).

2-(4-Bromobutoxy)-7-(hexadecyloxy)-9*H*-fluoren-9-one [Br(O4,O16)]. According to GP6, from **11e** (124 mg, 283 µmol), 1,4-dibromobutane (0.10 mL, 183 mg, 0.849 mmol), K_2CO_3 (117 mg, 849 µmol), MeCN (40 mL); yield: 130 mg, 0.227 mmol, 80%. **^{1}H-NMR** (400 MHz,

$CDCl_3$): δ = 0.82–0.94 (m, 3H, CH_3), 1.19–1.39 (m, 24H, CH_2), 1.39–1.52 (m, 2H, $OCH_2CH_2CH_2$), 1.71–1.86 (m, 2H, OCH_2CH_2), 1.91–2.01 (m, 2H, $BrCH_2CH_2CH_2$), 2.01–2.13 (m, 2H, $BrCH_2CH_2$), 3.49 (t, *J* = 6.5 Hz, 2H, $BrCH_2$), 3.98 (t, *J* = 6.6 Hz, 2H, OCH_2), 4.02 (t, *J* = 6.0 Hz, 2H, Br $CH_2CH_2CH_2CH_2O$), 6.90–6.95 (m, 2H, 3-H, 6-H), 7.12–7.17 (m, 2H, 1-H, 8-H), 7.25–7.30 (m, 2H, 4-H, 5-H) ppm; **^{13}C-NMR** (101 MHz, $CDCl_3$): δ = 14.1 (*C*H_3), 22.7 (CH_3*C*H_2), 26.0, 27.8, 29.2, 29.37, 29.41, 29.57, 29.60, 29.67, 29.69, 29.71 (*C*H_2), 31.9 (CH_3CH_2*C*H_2), 33.3 (Br*C*H_2), 67.4 ($Br(CH_2)_3$*C*H_2O), 68.6 (O*C*H_2), 110.19, 110.24 (C-1, C-8), 120.48, 120.51 (C-4, C-5), 120.8, 120.9 (C-3, C-6), 135.96, 136.02 (C-1', C-8'), 137.3, 137.7 (C-4', C-5') 159.2 (C-2), 159.6 (C-7), 193.8 (*C*=O) ppm; **FT-IR** (ATR): $\tilde{\nu}$ = 2916 (vs), 2850 (s), 1714 (s), 1606 (w), 1591 (w), 1464 (s), 1404 (w), 1291 (s), 1267 (m), 1249 (m), 1223 (m), 1141 (w), 1046 (m), 997 (w), 972 (w), 938 (w), 891 (w), 812 (m), 799 (m), 784 (m), 719 (w), 647 (w), 597 (w), 557 (w), 515 (w) cm^{-1}; **MS** (ESI): *m/z* = 593 $[M+Na]^+$, 447, 393, 353; **HRMS** (ESI): calcd. for $[C_{33}H_{47}BrO_3Na]^+$ 593.2601, found: 593.2551 $[M+Na]^+$; **elemental analysis**: calcd. (%) for $C_{33}H_{47}BrO_3$: C 69.34, H 8.29, found: C 69.57, H 8.49; **DSC**: Cr 80 [50.8 kJ mol^{-1}] I (3. H); I 77 [–4.7 kJ mol^{-1}] SmA 66 [–46.5 kJ mol^{-1}] Cr (3. C).

2-(6-Bromohexyloxy)-7-(octyloxy)-9*H*-fluoren-9-one [Br(O6,O8)]. According to GP6, from **11a** (185 mg, 570 µmol), 1,6-dibromohexane (0.26 mL, 417 mg, 1.71 mmol), K_2CO_3 (236 mg, 1.71 mmol), MeCN (30 mL); yield: 216 mg, 443 µmol, 77%. **^{1}H-NMR** (500 MHz, $CDCl_3$): δ = 0.84–0.93 (m, 3H, CH_3), 1.22–1.39 (m, 8H, CH_2), 1.40–1.57 (m, 6H, $OCH_2CH_2CH_2$, $BrCH_2CH_2CH_2$), 1.73–1.85 (m, 4H, OCH_2CH_2), 1.85–1.96 (m, 2H, $BrCH_2CH_2$), 3.43 (t, *J* = 6.8 Hz, 2H, $BrCH_2$), 3.92–4.04 (m, 4H, OCH_2), 6.90–6.92 (m, 1H, 3-H), 6.92–6.94 (m, 1H, 6-H), 7.12–7.15 (m, 2H, 1-H, 8-H), 7.24–7.28 (m, 2H, 4-H, 5-H) ppm; **^{13}C-NMR** (126 MHz, $CDCl_3$): δ = 14.1 (*C*H_3), 22.7 (CH_3*C*H_2), 25.2, 26.0, 27.9, 29.0, 29.16, 29.23, 29.3 (*C*H_2), 31.8 (CH_3CH_2*C*H_2), 32.7 ($BrCH_2$*C*H_2), 33.8 (Br*C*H_2), 68.2, 68.6 (O*C*H_2), 110.1, 110.2 (C-1, C-8), 120.5 (C-4, C-5), 120.8, 120.9 (C-3, C-6), 135.91, 135.94 (C-1', C-8') 137.3, 137.5 (C-4', C-5'), 159.3, 159.5 (C-2, C-7), 193.9 (*C*=O) ppm; **FT-IR** (ATR): $\tilde{\nu}$ = 3053 (w), 2925 (m), 2855 (m), 1712 (s), 1607 (w), 1592 (w), 1459 (vs), 1392 (m), 1281 (s), 1244 (s), 1221 (s), 1169 (w), 1136 (w), 1040 (m), 996 (w), 969 (w), 894 (w), 817 (m), 788 (s), 727 (w), 646 (w), 608 (w), 562 (w), 517 (w) cm^{-1}; **MS** (EI): *m/z* = 488.2 (70) $[M]^+$, 212.1 (100) $[M–C_8H_{17}–C_6H_{12}Br]^+$; **HRMS** (EI): calcd. for $[C_{27}H_{35}BrO_3]^+$ 488.1753, found: 488.1754 $[M]^+$; **elemental analysis**: calcd. (%) for $C_{27}H_{35}BrO_3$: C 66.53, H 7.24, found: C 66.24, H 7.07; **DSC**: Cr 50 [20.4 kJ mol^{-1}] SmA 70 [4.3 kJ mol^{-1}] I (3. H); I 69 [–3.6 kJ mol^{-1}] SmA 25 [–16.9 kJ mol^{-1}] Cr (3. C).

2-(6-Bromohexyloxy)-7-(decyloxy)-9*H*-fluoren-9-one [Br(O6,O10)]. According to GP6, from **11b** (130 mg, 369 µmol), 1,6-dibromohexane (0.17 mL, 271 mg, 1.11 mmol), K_2CO_3 (153 mg, 1.11 mmol), MeCN (40 mL); yield: 135 mg, 262 µmol, 71%. **^{1}H-NMR** (400 MHz, $CDCl_3$): δ = 0.88 (t, *J* = 6.6 Hz, 3H, CH_3), 1.20–1.40 (m, 14H, CH_2), 1.40–1.55 (m, 4H, $OCH_2CH_2CH_2$), 1.73–1.85 (m, 4H, OCH_2CH_2), 1.90 (m_c, 2H, $BrCH_2CH_2$), 3.43 (t, *J* = 6.8 Hz, 2H, $BrCH_2$), 3.94–4.02 (m, 4H, OCH_2), 6.89–6.95 (m, 2H, 3-H, 6-H), 7.12–7.16 (m, 2H, 1-H, 8-H), 7.24–7.29 (m, 2H, 4-H, 5-H) ppm; **^{13}C-NMR** (101 MHz, $CDCl_3$): δ = 14.1 (*C*H_3), 22.7, 25.3, 26.0, 27.9, 29.0, 29.2,

29.3, 29.4, 29.55, 29.57 (CH_2), 31.9 ($CH_3CH_2CH_2$), 32.7 ($BrCH_2CH_2$), 33.7 ($BrCH_2$), 68.3, 68.6 (OCH_2), 110.18, 110.22 (C-1, C-8), 120.5 (C-4, C-5), 120.8, 120.9 (C-3, C-6), 135.96, 136.99 (C-1', C-8'), 137.3, 137.5 (C-4', C-5'), 159.4, 159.5 (C-2, C-7), 193.9 (*C*=O) ppm; **FT-IR** (ATR): $\tilde{\nu}$ = 3051 (w), 2923 (s), 2853 (m), 1712 (s), 1607 (m), 1592 (w), 1462 (vs), 1392 (m), 1287 (s), 1223 (s), 1136 (w), 1040 (m), 998 (w), 971 (w), 893 (w), 816 (m), 787 (m), 729 (w), 647 (w), 607 (w), 563 (w), 517 (w) cm^{-1}; **MS** (EI): *m/z* = 516.2 (100) $[M]^+$, 212.0 (90) $[M–C_{10}H_{21}–C_6H_{12}Br]^+$; **HRMS** (EI): calcd. for $[C_{29}H_{39}BrO_3]^+$ 516.2067, found: 516.2063 $[M]^+$; **elemental analysis**: calcd. (%) for $C_{29}H_{39}BrO_3$: C 67.56, H 7.63, found: C 67.69, H 7.52; **DSC**: Cr 30 [8.7 kJ mol^{-1}] SmA 74 [3.9 kJ mol^{-1}] I (3. H); I 72 [–3.9 kJ mol^{-1}] SmA 21 [–7.0 kJ mol^{-1}] Cr (3. C).

2-(6-Bromohexyloxy)-7-(dodecyloxy)-9*H*-fluoren-9-one [Br(O6,O12)]. According to GP6, from **11c** (120 mg, 315 µmol), 1,6-dibromohexane (0.15 mL, 231 mg, 0.945 mmol), K_2CO_3 (131 mg, 945 µmol), MeCN (30 mL); yield: 139 mg, 256 µmol, 81%. **^{1}H-NMR** (300 MHz, $CDCl_3$): δ = 0.84–0.92 (m, 3H, CH_3), 1.20–1.57 (m, 22H, CH_2), 1.71–1.96 (m, 6H, OCH_2CH_2, $BrCH_2CH_2$), 3.43 (t, *J* = 6.8 Hz, 2H, $BrCH_2$), 3.92–4.04 (m, 4H, OCH_2), 6.89–6.92 (m, 1H, 3-H), 6.92–6.95 (m, 1H, 6-H), 7.12–7.16 (m, 2H, 1-H, 8-H), 7.24–7.29 (m, 2H, 4-H, 5-H) ppm; **^{13}C-NMR** (75 MHz, $CDCl_3$): δ = 14.1 (CH_3), 22.7 (CH_3CH_2), 25.3, 26.0, 27.9, 29.0, 29.2, 29.4, 29.57, 29.60, 29.64 (CH_2), 31.9 ($CH_3CH_2CH_2$), 32.7 ($BrCH_2CH_2$), 33.8 ($BrCH_2$), 68.3, 68.6 (OCH_2), 110.15, 110.19 (C-1, C-8), 120.5 (C-4, C-5), 120.8, 120.9 (C-3, C-6), 135.94, 135.96 (C-1', C-8'), 137.3, 137.5 (C-4', C-5'), 159.4, 159.5 (C-2, C-7), 193.9 (*C*=O) ppm; **FT-IR** (ATR): $\tilde{\nu}$ = 3053 (w), 2922 (s), 2852 (m), 1712 (s), 1607 (w), 1592 (w), 1460 (vs), 1392 (m), 1283 (s), 1246 (s), 1222 (s), 1168 (w), 1136 (w), 1039 (m), 999 (w), 970 (w), 894 (w), 816 (m), 798 (m), 788 (m), 726 (w), 647 (w), 608 (w), 563 (w), 517 (w) cm^{-1}; **MS** (ESI): *m/z* = 565 $[M+Na]^+$, 485, 413; **HRMS** (ESI): calcd. for $[C_{31}H_{43}BrO_3Na]^+$ 565.2288, found: 565.2272 $[M+Na]^+$; **elemental analysis**: calcd. (%) for $C_{31}H_{43}BrO_3$: C 68.50, H 7.97, found: C 68.46, H 7.74; **DSC**: Cr_1 10 [–3.4 kJ mol^{-1}] Cr_2 52 [32.3 kJ mol^{-1}] SmA 74 [6.9 kJ mol^{-1}] I (3. H); I 73 [–6.4 kJ mol^{-1}] SmA 14 [–7.4 kJ mol^{-1}] Cr (3. C).

2-(6-Bromohexyloxy)-7-(tetradecyloxy)-9*H*-fluoren-9-one [Br(O6,O14)]. According to GP6, from **11d** (298 mg, 729 µmol), 1,6-dibromohexane (0.34 mL, 534 mg, 2.19 mmol), K_2CO_3 (303 mg, 2.19 mmol), MeCN (30 mL); yield: 325 mg, 569 µmol, 78%. **^{1}H-NMR** (500 MHz, $CDCl_3$): δ = 0.88 (t, *J* = 6.9 Hz, 3H, CH_3), 1.21–1.39 (m, 20H, CH_2), 1.39–1.55 (m, 6H, $OCH_2CH_2CH_2$, $BrCH_2CH_2CH_2$), 1.72–1.85 (m, 4H, OCH_2CH_2), 1.85–1.95 (m, 2H, $BrCH_2CH_2$), 3.43 (t, *J* = 6.8 Hz, 2H, $BrCH_2$), 3.94–4.02 (m, 4H, OCH_2), 6.89–6.94 (m, 2H, 3-H, 6-H), 7.12–7.15 (m, 2H, 1-H, 8-H), 7.24–7.28 (m, 2H, 4-H, 5-H) ppm; **^{13}C-NMR** (126 MHz, $CDCl_3$): δ = 14.1 (CH_3), 22.7 (CH_3CH_2), 25.3, 26.0, 27.9, 29.0, 29.2, 29.4, 29.57, 29.60, 29.66, 29.68, 29.70, 29.72 (CH_2), 31.9 ($CH_3CH_2CH_2$), 32.7 ($BrCH_2CH_2$), 33.7 ($BrCH_2$), 68.3, 68.6 (OCH_2), 110.15, 110.19 (C-1, C-8), 120.5 (C-4, C-5), 120.8, 120.9 C-3, C-6), 135.9, 136.0 (C-1', C-8'), 137.3, 137.5 (C-4', C-5'), 159.4, 159.5 (C-2, C-7), 193.9 (*C*=O) ppm; **FT-IR** (ATR): $\tilde{\nu}$ = 3306 (w), 2981 (w), 2935 (w), 2875 (w), 1706 (s), 1617 (w), 1508 (m), 1468 (m), 1386 (m), 1375 (m), 1323 (w), 1225 (s), 1179 (m), 1145 (m), 1105 (vs), 1039 (s), 1016 (s), 929 (w), 859 (w), 798 (m), 772 (m),

600 (w), 413 (w) cm^{-1}; **MS** (ESI): *m/z* = 593 $[M+Na]^+$, 571 $[M]^+$; **HRMS** (ESI): calcd. for $[C_{33}H_{47}BrO_3Na]^+$ 593.2601, found: 593.2621 $[M+Na]^+$; **elemental analysis**: calcd. (%) for $C_{33}H_{47}BrO_3$: C 69.34, H 8.29, found: C 69.62, H 8.18; **DSC**: Cr_1 19 [–19.4 kJ mol^{-1}] Cr_2 61 [26.8 kJ mol^{-1}] SmA 79 [5.5 kJ mol^{-1}] I (3. H); I 75 [–5.6 kJ mol^{-1}] SmA 14 [–3.7 kJ mol^{-1}] Cr (3. C).

2-(6-Bromohexyloxy)-7-(hexadecyloxy)-9*H*-fluoren-9-one [Br(O6,O16)]. According to GP6, from **11e** (220 mg, 504 µmol), 1,6-dibromohexane (0.23 mL, 369 mg, 1.51 mmol), K_2CO_3 (209 mg, 1.51 mmol), MeCN (50 mL); yield: 193 mg, 322 µmol, 64%. **^{1}H-NMR** (400 MHz, $CDCl_3$): δ = 0.88 (t, *J* = 6.8 Hz, 3H, CH_3), 1.19–1.39 (m, 24H, CH_2), 1.39–1.60 (m, 6H, $OCH_2CH_2CH_2$, $BrCH_2CH_2CH_2$), 1.72–1.85 (m, 4H, OCH_2CH_2), 1.85–1.98 (m, 2H, $BrCH_2CH_2$), 3.43 (t, *J* = 6.8 Hz, 2H, $BrCH_2$), 3.91–4.05 (m, 4H, OCH_2), 6.90–6.92 (m, 1H, 3-H), 6.92–6.95 (m, 1H, 6-H), 7.12–7.16 (m, 2H, 1-H, 8-H), 7.24–7.29 (m, 2H, 4-H, 5-H) ppm; **^{13}C-NMR** (101 MHz, $CDCl_3$): δ = 14.1 (CH_3), 22.7 (CH_3CH_2), 25.3, 26.0, 27.9, 29.0, 29.2, 29.4, 29.57, 29.60, 29.67, 29.68, 29.70 (CH_2), 31.9 ($CH_3CH_2CH_2$), 32.7 ($BrCH_2CH_2$), 33.7 ($BrCH_2$), 68.3, 68.6 (OCH_2), 110.18, 110.22 (C-1, C-8), 120.5 (C-4, C-5), 120.85, 120.90 (C-3, C-6), 135.96, 135.99 (C-1', C-8'), 137.3, 137.5 (C-4', C-5'), 159.4, 159.6 (C-2, C-7), 193.9 (*C*=O) ppm; **FT-IR** (ATR): $\tilde{\nu}$ = 2916 (vs), 2850 (s), 1715 (s), 1612 (w), 1591 (w), 1464 (s), 1391 (w), 1290 (s), 1250 (m), 1223 (s), 1142 (w), 1041 (m), 995 (w), 970 (w), 907 (w), 891 (w), 811 (m), 799 (m), 784 (m), 720 (m), 647 (w), 515 (w) cm^{-1}; **MS** (ESI): *m/z* = 621 $[M+Na]^+$, 599 $[M]^+$; **HRMS** (ESI): calcd. for $[C_{35}H_{51}BrO_3Na]^+$ 621.2914, found: 621.2909 $[M+Na]^+$; **elemental analysis**: calcd. (%) for $C_{35}H_{451}BrO_3$: C 70.10, H 8.57, found: C 70.19, H 8.56; **DSC**: Cr 70 [42.6 kJ mol^{-1}] SmA 81 [7.7 kJ mol^{-1}] I (3. H); I 80 [–7.0 kJ mol^{-1}] SmA 51 [–38.3 kJ mol^{-1}] Cr (3. C).

2-(8-Bromooctyloxy)-7-(decyloxy)-9*H*-fluoren-9-one [Br(O8,O10)]. According to GP6, from **11b** (130 mg, 369 µmol), 1,8-dibromooctane (0.21 mL, 302 mg, 1.11 mmol), K_2CO_3 (153 mg, 1.11 mmol), MeCN (40 mL); yield: 142 mg, 261 µmol, 71%. **^{1}H-NMR** (700 MHz, $CDCl_3$): δ = 0.88 (t, *J* = 7.1 Hz, 3H, CH_3), 1.22–1.41 (m, 16H, CH_2), 1.41–1.51 (m, 6H, $OCH_2CH_2CH_2$, $BrCH_2CH_2CH_2$), 1.72–1.82 (m, 4H, OCH_2CH_2), 1.82–1.92 (m, 2H, $BrCH_2CH_2$), 3.41 (t, *J* = 6.8 Hz, 2H, $BrCH_2$), 3.92–4.02 (m, 4H, OCH_2), 6.90–6.94 (m, 2H, 3-H, 6-H), 7.13–7.15 (m, 2H, 1-H, 8-H), 7.25–7.27 (m, 2H, 4-H, 5-H) ppm; **^{13}C-NMR** (176 MHz, $CDCl_3$): δ = 14.1 (CH_3), 22.7 (CH_3CH_2), 25.9, 26.0, 28.1, 28.7, 29.1, 29.15, 29.17, 29.3, 29.4, 29.55, 29.57 (CH_2), 31.9 ($CH_3CH_2CH_2$), 32.8 ($BrCH_2CH_2$), 34.0 ($BrCH_2$), 68.5, 68.6 (OCH_2), 110.16, 110.19 (C-1, C-8), 120.5 (C-4, C-5), 120.86, 120.87 (C-3, C-6), 135.9, 136.0 (C-1', C-8'), 137.36, 137.42 (C-4', C-5'), 159.4, 159.5 (C-2, C-7), 193.9 (*C*=O) ppm; **FT-IR** (ATR): $\tilde{\nu}$ = 2922 (s), 2852 (m), 1713 (s), 1607 (w), 1592 (w), 1461 (vs), 1444 (s), 1391 (w), 1285 (s), 1247 (s), 1223 (s), 1170 (w), 1137 (w), 1038 (m), 1004 (w), 970 (w), 908 (w), 816 (m), 788 (m), 732 (m), 647 (w), 607 (w), 563 (w), 518 (w) cm^{-1}; **MS** (ESI): *m/z* = 565 $[M+Na]^+$, 543 $[M]^+$; **HRMS** (ESI): calcd. for $[C_{31}H_{43}BrO_3]^+$ 543.2468, found: 543.2463 $[M]^+$; **elemental analysis**: calcd. (%) for $C_{31}H_{43}BrO_3$: C 68.50, H 7.97, found: C 68.26, H 7.81; **DSC**: Cr 38 [17.2 kJ mol^{-1}] SmA 75 [5.8 kJ mol^{-1}] I (3. H); I 72 [–4.9 kJ mol^{-1}] SmA 28 [–14.8 kJ mol^{-1}] Cr (3. C).

2-(8-Bromooctyloxy)-7-(dodecyloxy)-9*H*-fluoren-9-one [Br(O8,O12)]. According to GP6, from **11c** (250 mg, 657 µmol), 1,8-dibromooctane (0.37 mL, 539 mg, 1.98 mmol), K_2CO_3 (274 mg, 1.98 mmol), MeCN (20 mL); yield: 330 mg, 577 µmol, 88%. **^{1}H-NMR** (500 MHz, $CDCl_3$): δ = 0.88 (t, *J* = 6.8 Hz, 3H, C*H*$_3$), 1.20–1.51 (m, 26H, C*H*$_2$), 1.74–1.82 (m, 4H, OCH$_2$C*H*$_2$), 1.86 (m$_c$, 2H, BrCH$_2$C*H*$_2$), 3.41 (t, *J* = 6.9 Hz, 2H, BrC*H*$_2$), 3.97 (t, *J* = 6.5 Hz, 4H, OC*H*$_2$), 6.92 (dd, *J* = 8.2 Hz, 2.4 Hz, 2H, 3-H, 6-H), 7.12–7.16 (m, 2H, 1-H, 8-H), 7.24–7.28 (m, 2H, 4-H, 5-H) ppm; **^{13}C-NMR** (126 MHz, $CDCl_3$): δ = 14.2 (*C*H$_3$), 22.7 (CH$_3$*C*H$_2$), 25.9, 26.0, 28.1, 28.7, 29.1, 29.2, 29.4, 29.59, 29.62, 29.66, 29.68 (*C*H$_2$), 31.9 (CH$_3$CH$_2$*C*H$_2$), 32.8 (BrCH$_2$*C*H$_2$), 34.0 (Br*C*H$_2$), 68.5, 68.6 (O*C*H$_2$), 110.15, 110.18 (C-1, C-8), 120.5 (C-4, C-5), 120.87, 120.88 (C-3, C-6), 135.95, 135.96 (C-1', C-8'), 137.36, 137.43 (C-4', C-5'), 159.4, 159.5 (C-2, C-7), 193.9 (*C*=O) ppm; **FT-IR** (ATR): $\tilde{\nu}$ = 2923 (s), 2852 (m), 1712 (m), 1608 (w), 1592 (w), 1460 (vs), 1444 (s), 1390 (w), 1284 (s), 1222 (s), 1169 (w), 1135 (w), 1037 (m), 1001 (m), 968 (w), 907 (s), 816 (m), 787 (m), 732 (vs), 647 (w), 607 (w), 562 (w), 517 (w) cm^{-1}; **MS** (ESI): *m/z* = 595 $[M+Na]^+$, 573 $[M+H]^+$; **HRMS** (ESI): calcd. for $[C_{33}H_{47}BrO_3H]^+$ 573.2767, found: 573.2795 $[M+H]^+$; **elemental analysis**: calcd. (%) for $C_{33}H_{47}BrO_3$: C 69.34, H 8.29, found: C 68.94, H 7.91; **DSC**: Cr$_1$ 31 [11.9 kJ mol^{-1}] Cr$_2$ 54 [2.9 kJ mol^{-1}] SmA 75 [8.5 kJ mol^{-1}] I (3. H); I 75 [–7.7 kJ mol^{-1}] SmA 19 [–9.7 kJ mol^{-1}] Cr (3. C).

2-(8-Bromooctyloxy)-7-(hexadecyloxy)-9*H*-fluoren-9-one [Br(O8,O16)]. According to GP6, from **11e** (200 mg, 458 µmol), 1,8-dibromooctane (0.26 mL, 374 mg, 1.37 mmol), K_2CO_3 (190 mg, 1.37 mmol), MeCN (50 mL); yield: 185 mg, 295 µmol, 64%. **^{1}H-NMR** (400 MHz, $CDCl_3$): δ = 0.83–0.93 (m, 3H, C*H*$_3$), 1.19–1.51 (m, 34H, C*H*$_2$), 1.73–1.82 (m, 4H, OCH$_2$C*H*$_2$), 1.87 (m$_c$, 2H, BrCH$_2$C*H*$_2$), 3.41 (t, *J* = 6.8 Hz, 2H, BrC*H*$_2$), 3.92–4.03 (m, 4H, OC*H*$_2$), 6.90–6.92 (m, 1H, 3-H), 6.92–6.95 (m, 1H, 6-H), 7.12–7.16 (m, 2H, 1-H, 8-H), 7.23–7.30 (m, 2H, 4-H, 5-H) ppm; **^{13}C-NMR** (101 MHz, $CDCl_3$): δ = 14.1 (*C*H$_3$), 22.7 (CH$_3$*C*H$_2$), 25.9, 26.0, 28.1, 28.7, 29.1, 29.16, 29.18, 29.4, 29.57, 29.60, 29.67, 29.69, 29.71 (*C*H$_2$), 31.9 (CH$_3$CH$_2$*C*H$_2$), 32.8 (BrCH$_2$*C*H$_2$), 33.9 (Br*C*H$_2$), 68.5, 68.6 (O*C*H$_2$), 110.19, 110.22 (C-1, C-8), 120.4 (C-4, C-5), 120.87, 120.88 (C-3, C-6), 135.97, 135.98 (C-1', C-8'), 137.37, 137.44 (C-4', C-5'), 159.46, 159.54 (C-2, C-7), 193.9 (*C*=O) ppm; **FT-IR** (ATR): $\tilde{\nu}$ = 2920 (vs), 2851 (s), 1714 (s), 1608 (w), 1466 (s), 1392 (w), 1291 (m), 1249 (m), 1224 (m), 1137 (w), 1041 (w), 970 (w), 892 (w), 815 (m), 788 (m), 722 (w), 647 (w), 516 (w) cm^{-1}; **MS** (ESI): *m/z* = 649 $[M+Na]^+$; **HRMS** (ESI): calcd. for $[C_{37}H_{55}BrO_3Na]^+$ 649.3227, found: 649.2949 $[M+Na]^+$; **elemental analysis**: calcd. (%) for $C_{37}H_{55}BrO_3$: C 70.79, H 8.83, found: C 70.88, H 8.87; **DSC**: Cr$_1$ 22 [–3.8 kJ mol^{-1}] Cr$_2$ 64 [43.3 kJ mol^{-1}] SmA 80 [9.5 kJ mol^{-1}] I (3. H); I 78 [–9.0 kJ mol^{-1}] SmA 38 [–9.7 kJ mol^{-1}] Cr$_3$ 30 [–4.0 kJ mol^{-1}] Cr$_2$ 19 [–3.5 kJ mol^{-1}] Cr$_1$ (3. C).

2-(10-Bromodecyloxy)-7-(decyloxy)-9*H*-fluoren-9-one [Br(O10,O10)]. According to GP6, from **11b** (400 mg, 1.13 mmol), 1,10-dibromodecane (1.02 g, 3.39 mmol), K_2CO_3 (468 mg, 3.39 mmol), MeCN (40 mL); yield: 500 mg, 875 µmol, 77%. **^{1}H-NMR** (400 MHz, $CDCl_3$): δ = 0.88 (t, *J* = 6.6 Hz, 3H, C*H*$_3$), 1.21–1.52 (m, 26H, C*H*$_2$), 1.73–1.81 (m, 4H, OCH$_2$C*H*$_2$), 1.85 (m$_c$, 2H, BrCH$_2$C*H*$_2$), 3.40 (t, *J* = 6.8 Hz, 2H, BrC*H*$_2$), 3.97 (t, *J* = 6.5 Hz, 4H, OC*H*$_2$), 6.92 (dd,

J = 8.2 Hz, J = 2.4 Hz, 2H, 3-H, 6-H), 7.14 (d, J = 2.4 Hz, 2H, 1-H, 8-H), 7.24–7.28 (m, 2H, 4-H, 5-H) ppm; **^{13}C-NMR** (101 MHz, $CDCl_3$): δ = 14.1 (CH_3), 22.7 (CH_3CH_2), 25.96, 25.99, 28.2, 28.7, 29.15, 29.18, 29.29, 29.32, 29.35, 29.36, 29.42, 29.55, 29.57 (CH_2), 31.9 ($CH_3CH_2CH_2$), 32.8 ($BrCH_2CH_2$), 34.0 ($BrCH_2$), 68.5, 68.6 (OCH_2), 110.20, 110.21 (C-1, C-8), 120.4 (C-4, C-5), 120.9 (C-3, C-6), 136.0 (C-1', C-8'), 137.37, 137.41 (C-4', C-5'), 159.49, 159.52 (C-2, C-7), 193.9 (*C*=O) ppm; **FT-IR** (ATR): $\tilde{\nu}$ = 2921 (s), 2852 (s), 1712 (s), 1608 (w), 1592 (w), 1479 (m), 1461 (vs), 1445 (s), 1391 (m), 1284 (s), 1247 (m), 1221 (s), 1170 (w), 1136 (w), 1110 (w), 1039 (m), 1003 (m), 970 (w), 894 (w), 815 (m), 798 (m), 788 (m), 734 (w), 685 (w), 646 (w), 607 (w), 563 (w), 517 (w) cm^{-1}; **MS** (ESI): *m/z* = 571 $[M+H]^+$, 413, 326; **HRMS** (ESI): calcd. for $[C_{33}H_{47}BrO_3H]^+$ 571.2781, found: 571.2764 $[M+H]^+$; **elemental analysis**: calcd. (%) for $C_{33}H_{47}BrO_3$: C 69.34, H 8.29, found: C 69.64, H 8.38; **DSC**: Cr 49 [23.9 kJ mol^{-1}] SmA 74 [9.9 kJ mol^{-1}] I (3. H); I 74 [–9.7 kJ mol^{-1}] SmA 36 [–17.8 kJ mol^{-1}] Cr (3. C).

2-(10-Bromodecyloxy)-7-(dodecyloxy)-9*H*-fluoren-9-one [Br(O10,O12)]. According to GP6, from **11c** (150 mg, 394 µmol), 1,10-dibromodecane (361 mg, 1.17 mmol), K_2CO_3 (162 mg, 1.17 mmol), MeCN (40 mL); 190 mg, 317 µmol, 81%. **^{1}H-NMR** (500 MHz, $CDCl_3$): δ = 0.88 (t, J = 6.9 Hz, 3H, CH_3), 1.22–1.51 (m, 30H, CH_2), 1.73–1.82 (m, 4H, OCH_2CH_2), 1.82–1.90 (m, 2H, $BrCH_2CH_2$), 3.41 (t, J = 6.9 Hz, 2H, $BrCH_2$), 3.97 (t, J = 6.6 Hz, 4H, OCH_2), 6.92 (dd, J = 8.1 Hz, J = 2.5 Hz, 2H, 3-H, 6-H), 7.14 (d, J = 2.5 Hz, 2H, 1-H, 8-H), 7.24–7.28 (m, 2H, 4-H, 5-H) ppm; **^{13}C-NMR** (126 MHz, $CDCl_3$): δ = 14.2 (CH_3), 22.7 (CH_3CH_2), 25.97, 26.00, 28.2, 28.8, 29.16, 29.19, 29.3, 29.4, 29.5, 29.59, 29.62, 29.66, 29.69 (CH_2), 31.9 ($CH_3CH_2CH_2$), 32.8 ($BrCH_2CH_2$), 34.1 ($BrCH_2$), 68.5, 68.6 (OCH_2), 110.16, 110.17 (C-1, C-8), 120.5 (C-4, C-5), 120.9 (C-3, C-6), 135.9 (C-1', C-8'), 137.37, 137.40 (C-4', C-5'), 159.47, 159.50 (C-2, C-7), 193.9 (*C*=O) ppm; **FT-IR** (ATR): $\tilde{\nu}$ = 2922 (vs), 2852 (s), 1713 (s), 1608 (w), 1592 (w), 1464 (vs), 1392 (w), 1288 (s), 1248 (m), 1223 (m), 1170 (w), 1136 (w), 1039 (m), 1001 (w), 969 (w), 894 (w), 816 (m), 788 (m), 724 (w), 647 (w), 607 (w), 563 (w), 517 (w) cm^{-1}; **MS** (ESI): *m/z* = 601 $[M+H]^+$, 623 $[M+Na]^+$, 326; **HRMS** (ESI): calcd. for $[C_{35}H_{51}BrO_3H]^+$ 601.3080, found: 601.3081 $[M+H]^+$; **elemental analysis**: calcd. (%) for $C_{35}H_{51}BrO_3$: C 70.10, H 8.57, found: C 70.35, H 8.65; **DSC**: Cr 54 [32.4 kJ mol^{-1}] SmA 76 [11.6 kJ mol^{-1}] I (3. H); I 76 [–11.5 kJ mol^{-1}] SmA 43 [–27.2 kJ mol^{-1}] Cr (3. C).

2-(12-Bromododecyloxy)-7-(decyloxy)-9*H*-fluoren-9-one [Br(O12,O10)]. According to GP6, from **11b** (400 mg, 1.13 mmol), 1,12-dibromododecane (1.11 g, 3.39 mmol), K_2CO_3 (468 mg, 3.39 mmol), MeCN (40 mL); yield: 532 mg, 887 µmol, 79%. **^{1}H-NMR** (400 MHz, $CDCl_3$): δ = 0.88 (t, J = 6.6 Hz, 3H, CH_3), 1.20–1.51 (m, 30H, CH_2), 1.71–1.82 (m, 4H, OCH_2CH_2), 1.85 (m_c, 2H, $BrCH_2CH_2$), 3.40 (t, J = 7.0 Hz, 2H, $BrCH_2$), 3.97 (t, J = 6.6 Hz, 4H, OCH_2), 6.92 (dd, J = 8.2 Hz, J = 2.4 Hz, 2H, 3-H, 6-H), 7.14 (d, J = 2.4 Hz, 2H, 1-H, 8-H), 7.23–7.29 (m, 2H, 4-H, 5-H) ppm; **^{13}C-NMR** (101 MHz, $CDCl_3$): δ = 14.1 (CH_3), 22.7 (CH_3CH_2), 25.97, 25.99, 28.2, 28.8, 29.17, 29.18, 29.32, 29.33, 29.37, 29.43, 29.51, 29.52, 29.55, 29.57 (CH_2), 31.9 ($CH_3CH_2CH_2$), 32.9 ($BrCH_2CH_2$), 34.0 ($BrCH_2$), 68.56, 68.59 (OCH_2), 110.2 (C-1, C-8), 120.4 (C-4, C-5), 120.9 (C-3, C-6), 136.0 (C-1', C-8'), 137.37, 137.39 (C-4', C-5'), 159.50, 159.52 (C-2,

C-7), 193.9 (C=O) ppm; **FT-IR** (ATR): $\tilde{\nu}$ = 2921 (vs), 2852 (s), 1713 (s), 1608 (w), 1592 (w), 1479 (m), 1459 (vs), 1444 (s), 1391 (m), 1280 (s), 1247 (s), 1220 (s), 1170 (w), 1136 (w), 1039 (m), 1002 (m), 970 (m), 908 (m), 816 (m), 798 (m), 788 (m), 734 (m), 685 (w), 647 (w), 607 (w), 563 (w), 517 (w) cm^{-1}; **MS** (ESI): *m/z* = 623 $[M+Na]^+$, 601 $[M+H]^+$, 413, 326; **HRMS** (ESI): calcd. for $[C_{35}H_{51}BrO_3H]^+$ 601.3080, found: 601.3060 $[M+H]^+$; **elemental analysis**: calcd. (%) for $C_{35}H_{51}BrO_3$: C 70.10, H 8.57, found: C 70.22, H 8.65; **DSC**: Cr_1 28 [13.9 kJ mol^{-1}] Cr_2 35 [9.0 kJ mol^{-1}] SmA 69 [9.9 kJ mol^{-1}] I (3. H); I 69 [–9.5 kJ mol^{-1}] SmA 15 [–10.3 kJ mol^{-1}] Cr (3. C).

2-(12-Bromododecyloxy)-7-(dodecyloxy)-9*H*-fluoren-9-one [Br(O12,O12)]. According to GP6, from **11c** (150 mg, 394 µmol), 1,12-dibromododecane (384 mg, 1.17 mmol), K_2CO_3 (162 mg, 1.17 mmol), MeCN (40 mL); yield: 195 mg, 311 µmol, 79%. **^{1}H-NMR** (500 MHz, $CDCl_3$): δ = 0.88 (t, J = 6.9 Hz, 3H, CH_3), 1.22–1.50 (m, 34H, CH_2), 1.74–1.82 (m, 4H, OCH_2CH_2), 1.82–1.89 (m, 2H, $BrCH_2CH_2$), 3.41 (t, J = 6.9 Hz, 2H, $BrCH_2$), 3.97 (t, J = 6.6 Hz, 4H, OCH_2), 6.92 (dd, J = 8.2 Hz, J = 2.4 Hz, 2H, 3-H, 6-H), 7.14 (d, J = 2.4 Hz, 2H, 1-H, 8-H), 7.24–7.28 (m, 2H, 4-H, 5-H) ppm; **^{13}C-NMR** (126 MHz, $CDCl_3$): δ = 14.2 (CH_3), 22.7 (CH_3CH_2), 26.0, 28.2, 28.8, 29.2, 29.35, 29.38, 29.45, 29.54, 29.59, 29.62, 29.66, 29.68 (CH_2), 31.9 ($CH_3CH_2CH_2$), 32.9 ($BrCH_2CH_2$), 34.1 ($BrCH_2$), 68.55, 68.58 (OCH_2), 110.2 (C-1, C-8), 120.5 (C-4, C-5), 120.9 (C-3, C-6), 136.0 (C-1', C-8'), 137.38, 137.40 (C-4', C-5'), 159.49, 159.50 (C-2, C-7), 194.0 (C=O) ppm; **FT-IR** (ATR): $\tilde{\nu}$ = 2919 (vs), 2851 (s), 1716 (s), 1612 (w), 1591 (w), 1466 (s), 1390 (w), 1290 (s), 1251 (m), 1223 (m), 1141 (w), 1137 (m), 998 (w), 969 (w), 908 (w), 891 (w), 810 (m), 799 (m), 785 (m), 733 (m), 648 (w), 606 (w), 566 (w), 515 (w) cm^{-1}; **MS** (ESI): *m/z* = 629 $[M+H]^+$, 371; **HRMS** (ESI): calcd. for $[C_{37}H_{55}BrO_3H]^+$ 629.3394, found: 629.3383 $[M+H]^+$; **elemental analysis**: calcd. (%) for $C_{37}H_{55}BrO_3$: C 70.79, H 8.83, found: C 70.92, H 8.90; **DSC**: Cr 63 [35.6 kJ mol^{-1}] SmA 75 [13.4 kJ mol^{-1}] I (3. H); I 75 [–11.1 kJ mol^{-1}] SmA 52 [–33.7 kJ mol^{-1}] Cr (3. C).

2-(4-Bromobutoxy)-7-(decylthio)-9*H*-fluoren-9-one [Br(O4,S10)]. According to GP6, from **13b** (127 mg, 345 µmol), 1,4-dibromobutane (0.12 mL, 223 mg, 1.04 mmol), K_2CO_3 (143 mg, 1.04 mmol), MeCN (15 mL); yield: 125 mg, 248 µmol, 72%. **^{1}H-NMR** (500 MHz, $CDCl_3$): δ = 0.87 (t, J = 6.9 Hz, 3H, CH_3), 1.19–1.34 (m, 12H, CH_2), 1.37–1.46 (m, 2H, $SCH_2CH_2CH_2$), 1.65 (m_c, 2H, SCH_2CH_2), 1.92–2.02 (m, 2H, OCH_2CH_2), 2.03–2.12 (m, 2H, $BrCH_2CH_2$), 2.93 (t, J = 7.4 Hz, 2H, SCH_2), 3.49 (t, J = 6.6 Hz, 2H, $BrCH_2$), 4.04 (t, J = 6.1 Hz, 2H, OCH_2), 6.96 (dd, $J_{3,4}$ = 8.2 Hz, $J_{1,3}$ = 2.5 Hz, 1H, 3-H), 7.16 (d, $J_{1,3}$ = 2.5 Hz, 1H, 1-H), 7.27–7.31 (m, 1H, 5-H), 7.32–7.38 (m, 2H, 4-H, 6-H), 7.52–7.54 (m, 1H, 8-H) ppm; **^{13}C-NMR** (126 MHz, $CDCl_3$): δ = 14.1 (CH_3), 22.7 (CH_3CH_2), 27.8, 28.8, 29.0, 29.1, 29.3, 29.4, 29.49, 29.53 (CH_2), 31.9 ($CH_3CH_2CH_2$), 33.3 ($BrCH_2$), 33.7 (SCH_2), 67.4 (OCH_2), 110.0 (C-1), 119.9 (C-5), 120.9 (C-3), 121.2 (C-4), 124.3 (C-8), 134.8 (C-6), 135.0 (C-8'), 135.7 (C-1'), 137.0 (C-4'), 137.5 (C-7), 142.2 (C-5'), 159.9 (C-2), 193.5 (C=O) ppm; **FT-IR** (ATR): $\tilde{\nu}$ = 2921 (vs), 2852 (s), 1718 (vs), 1602 (m), 1488 (m), 1458 (s), 1436 (m), 1416 (m), 1342 (w), 1297 (m), 1251 (s), 1225 (w), 1207 (m), 1182 (w), 1142 (m), 1093 (w), 1057 (m), 1045 (w), 934 (w), 900 (w), 829 (m), 784 (s), 735 (w), 644 (w), 501 (m) cm^{-1}; **MS** (EI): *m/z* =504.1 (100) $[M]^+$, 368.2 (15), 228.0 (23), 135.0 (27);

HRMS (EI): calcd. for $[C_{27}H_{35}\,BrO_2S]^+$ 504.1524, found: 504.1519 $[M]^+$; **elemental analysis**: calcd. (%) for $C_{27}H_{35}BrO_2S$: C 64.40, H 7.01, found: C 64.60, H 6.95; **DSC**: Cr 80 [37.5 kJ mol^{-1}] I (3. H); I 50 [–34.3 kJ mol^{-1}] Cr (3. C).

2-(4-Brombutoxy)-7-(dodecylthio)-9*H*-fluoren-9-one [Br(O4,S12)]. According to GP6, from **13c** (180 mg, 454 µmol), 1,4-dibromobutane (0.16 mL, 291 mg, 1.35 mmol), K_2CO_3 (187 mg, 1.35 mmol), MeCN (30 mL); yield: 179 mg, 337 µmol, 74%. **^{1}H-NMR** (700 MHz, $CDCl_3$): δ = 0.88 (t, J = 7.1 Hz, 3H, CH_3), 1.20–1.32 (m, 16H, CH_2), 1.39–1.45 (m, 2H, $SCH_2CH_2CH_2$), 1.65 (m_c, 2H, SCH_2CH_2), 1.93–1.99 (m, 2H, OCH_2CH_2), 2.04–2.10 (m, 2H, $BrCH_2CH_2$), 2.94 (t, J = 7.4 Hz, 2H, SCH_2), 3.50 (t, J = 6.6 Hz, 2H, $BrCH_2$), 4.04 (t, J = 6.1 Hz, 2H, OCH_2), 6.96 (dd, $J_{3,4}$ = 8.2 Hz, $J_{1,3}$ = 2.5 Hz, 1H, 3-H), 7.16 (d, $J_{1,3}$ = 2.5 Hz, 1H, 1-H), 7.29 (d, $J_{5,6}$ = 7.7 Hz, 1H, 5-H), 7.34–7.38 (m, 2H, 4-H, 6-H), 7.53–7.54 (m, 1H, 8-H) ppm; **^{13}C-NMR** (176 MHz, $CDCl_3$): δ = 14.1 (CH_3), 22.7 (CH_3CH_2), 27.8, 28.8, 29.0, 29.1, 29.3, 29.4, 29.5, 29.57, 29.63, 29.64 (CH_2), 31.9 ($CH_3CH_2CH_2$), 33.3 ($BrCH_2$), 33.7 (SCH_2), 67.4 (OCH_2), 110.0 (C-1), 119.9 (C-5), 120.9 (C-3), 121.2 (C-4), 124.3 (C-8), 134.8 (C-6), 135.0 (C-8'), 135.7 (C-1'), 137.0 (C-4'), 137.5 (C-7), 142.2 (C-5'), 159.9 (C-2), 193.5 (*C*=O) ppm; **FT-IR** (ATR): $\tilde{\nu}$ = 2917 (s), 2849 (s), 1716 (s), 1601 (m), 1488 (m), 1456 (m), 1434 (m), 1415 (m), 1341 (w), 1296 (m), 1249 (vs), 1206 (m), 1178 (w), 1141 (m), 1123 (w), 1092 (w), 1057 (m), 1046 (w), 933 (w), 900 (w), 828 (m), 783 (s), 732 (m), 643 (w), 557 (w), 500 (m) cm^{-1}; **MS** (EI): m/z = 532.2 (100) $[M]^+$, 450.3 (10), 396.2 (10), 228.0 (15), 135.0 (18); **HRMS** (EI): calcd. for $[C_{29}H_{39}\,BrO_2S]^+$ 532.1837, found: 532.1835 $[M]^+$; **elemental analysis**: calcd. (%) for $C_{29}H_{39}BrO_2S$: C 65.52, H 7.40, S 6.03, found: C 65.65, H 7.39, S 5.95; **DSC**: Cr 83 [35.7 kJ mol^{-1}] I (3. H); I 68 [–37.2 kJ mol^{-1}] Cr (3. C).

2-(4-Bromobutoxy)-7-(tetradecylthio)-9*H*-fluoren-9-one [Br(O4,S14)]. According to GP6, from **13d** (200 mg, 471 µmol), 1,4-dibromobutane (0.17 mL, 305 mg, 1.41 mmol), K_2CO_3 (195 mg, 1.41 mmol), MeCN (40 mL); yield: 194 mg, 347 µmol, 74%. **^{1}H-NMR** (400 MHz, $CDCl_3$): δ = 0.88 (t, J = 6.8 Hz, 3H, CH_3), 1.20–1.35 (m, 20H, CH_2), 1.37–1.47 (m, 2H, $SCH_2CH_2CH_2$), 1.65 (m_c, 2H, SCH_2CH_2), 1.91–2.02 (m, 2H, OCH_2CH_2), 2.02–2.13 (m, 2H, $BrCH_2CH_2$), 2.91–2.96 (m, 2H, SCH_2), 3.49 (t, J = 6.5 Hz, 2H, $BrCH_2$), 4.04 (t, J = 6.0 Hz, 2H, OCH_2), 6.96 (dd, $J_{3,4}$ = 8.2 Hz, $J_{1,3}$ = 2.5 Hz, 1H, 3-H), 7.16 (d, $J_{1,3}$ = 2.5 Hz, 1H, 1-H), 7.29 (d, $J_{5,6}$ = 7.8 Hz, 1H, 5-H), 7.33–7.39 (m, 2H, 4-H, 6-H), 7.53–7.55 (m, 1H, 8-H) ppm; **^{13}C-NMR** (101 MHz, $CDCl_3$): δ = 14.1 (CH_3), 22.7 (CH_3CH_2), 27.8, 28.8, 29.0, 29.2, 29.36, 29.39, 29.5, 29.6, 29.65, 29.67, 29.69 (CH_2), 31.9 ($CH_3CH_2CH_2$), 33.3 ($BrCH_2$), 33.7 (SCH_2), 67.5 (OCH_2), 110.0 (C-1), 119.9 (C-5), 120.9 (C-3), 121.2 (C-4), 124.3 (C-8), 134.8 (C-6), 135.0 (C-8'), 135.7 (C-1'), 137.0 (C-4'), 137.6 (C-7), 142.2 (C-5'), 160.0 (C-2), 193.5 (*C*=O) ppm; **FT-IR** (ATR): $\tilde{\nu}$ = 2917 (vs), 2850 (s), 1717 (s), 1601 (m), 1488 (m), 1458 (m), 1435 (m), 1417 (w), 1342 (w), 1296 (m), 1283 (m), 1251 (s), 1207 (w), 1181 (w), 1141 (m), 1092 (w), 1058 (w), 1045 (w), 934 (w), 900 (w), 830 (m), 784 (m), 735 (w), 721 (w), 644 (w), 557 (w), 500 (m) cm^{-1}; **MS** (ESI): m/z = 591, 560 $[M]^+$, 447, 424; **HRMS** (ESI): calcd. for $[C_{31}H_{43}BrO_2S]^+$ 560.2145, found: 560.2093 $[M]^+$; **elemental analysis**: calcd. (%) for $C_{31}H_{43}BrO_2S$: C 66.53, H 7.74, found: C 66.71, H 7.58; **DSC**: Cr 89 [44.6 kJ mol^{-1}] I (3. H); I 74 [–44.5 kJ mol^{-1}] Cr (3. C).

2-(6-Bromohexyloxy)-7-(decylthio)-9*H*-fluoren-9-one [Br(O6,S10)]. According to GP6, from **13b** (110 mg, 298 µmol), 1,6-dibromohexane (0.14 mL, 218 mg, 894 µmol), K_2CO_3 (124 mg, 894 µmol), MeCN (20 mL); yield: 122 mg, 230 µmol, 77%. **^{1}H-NMR** (300 MHz, $CDCl_3$): δ = 0.83–0.92 (m, 3H, CH_3), 1.19–1.36 (m, 12H, CH_2), 1.36–1.58 (m, 6H, $SCH_2CH_2CH_2$, $OCH_2CH_2CH_2$, $BrCH_2CH_2CH_2$), 1.65 (m_c, 2H, SCH_2CH_2), 1.75–1.97 (m, 4H, OCH_2CH_2, $BrCH_2CH_2$), 2.88–2.99 (m, 2H, SCH_2), 3.43 (t, J = 6.8 Hz, 2H, $BrCH_2$), 4.00 (t, J = 6.4 Hz, 2H, OCH_2), 6.96 (dd, $J_{3,4}$ = 8.2 Hz, $J_{1,3}$ = 2.5 Hz, 1H, 3-H), 7.16 (d, $J_{1,3}$ = 2.4 Hz, 1H, 1-H), 7.27–7.31 (m, 1H, 5-H), 7.31–7.39 (m, 2H, 4-H, 6-H), 7.51–7.55 (m, 1H, 8-H) ppm; **^{13}C-NMR** (75 MHz, $CDCl_3$): δ = 14.1 (CH_3), 22.7 (CH_3CH_2), 25.2, 27.9, 28.8, 28.98, 29.02, 29.2, 29.3, 29.50, 29.53 (CH_2), 31.9 ($CH_3CH_2CH_2$), 32.7 ($BrCH_2CH_2$), 33.7 ($BrCH_2$, SCH_2), 68.3 (OCH_2), 110.0 (C-1), 119.8 (C-5), 121.0 (C-3), 121.2 (C-4), 124.3 (C-8), 134.8 (C-6), 135.0 (C-8'), 135.7 (C-1'), 136.8 (C-4'), 137.4 (C-7), 142.3 (C-5'), 160.2 (C-2), 193.6 (*C*=O) ppm; **FT-IR** (ATR): $\tilde{\nu}$ = 2922 (vs), 2852 (s), 1715 (vs), 1601 (m), 1488 (m), 1457 (s), 1435 (m), 1417 (m), 1344 (w), 1298 (m), 1252 (s), 1226 (m), 1208 (w), 1181 (w), 1142 (m), 1089 (w), 1056 (w), 1029 (w), 995 (w), 902 (w), 841 (w), 830 (m), 784 (m), 758 (w), 724 (w), 646 (w), 554 (w), 501 (m) cm^{-1}; **MS** (ESI): *m/z* = 563, 532 $[M]^+$, 447, 423, 393, 368; **HRMS** (ESI): calcd. for $[C_{29}H_{39}BrO_2S]^+$ 532.1832, found: 532.1827 $[M]^+$; **elemental analysis**: calcd. (%) for $C_{29}H_{39}BrO_2S$: C 65.52, H 7.40, S 6.03, found: C 65.67, H 7.40, S 5.78; **DSC**: Cr 75 [43.8 kJ mol^{-1}] I (3. H); I 55 [–7.3 kJ mol^{-1}] SmA 30 [–17.4 kJ mol^{-1}] Cr (3. C).

2-(6-Bromohexyloxy)-7-(dodecylthio)-9*H*-fluoren-9-one [Br(O6,S12)]. According to GP6, from **13c** (130 mg, 328 µmol), 1,6-dibromohexane (0.15 mL, 240 mg, 984 µmol), K_2CO_3 (137 mg, 984 µmol), MeCN (30 mL); yield: 144 mg, 257 µmol, 78%. **^{1}H-NMR** (400 MHz, $CDCl_3$): δ = 0.88 (t, J = 6.7 Hz, 3H, CH_3), 1.20–1.36 (m, 16H, CH_2), 1.36–1.46 (m, 2H, $SCH_2CH_2CH_2$), 1.46–1.58 (m, 4H, $OCH_2CH_2CH_2$, $BrCH_2CH_2CH_2$), 1.65 (m_c, 2H, SCH_2CH_2), 1.81 (m_c, 2H, OCH_2CH_2), 1.90 (m_c, 2H, $BrCH_2CH_2$), 2.93 (t, J = 7.4 Hz, 2H, SCH_2), 3.43 (t, J = 6.8 Hz, 2H, $BrCH_2$), 4.00 (t, J = 6.5 Hz, 2H, OCH_2), 6.96 (dd, $J_{3,4}$ = 8.2 Hz, $J_{1,3}$ = 2.4 Hz, 1H, 3-H), 7.16 (d, $J_{1,3}$ = 2.4 Hz, 1H, 1-H), 7.28 (d, $J_{5,6}$ = 7.8 Hz, 1H, 5-H), 7.32–7.39 (m, 2H, 4-H, 6-H), 7.52–7.55 (m, 1H, 8-H) ppm; **^{13}C-NMR** (101 MHz, $CDCl_3$): δ = 14.1 (CH_3), 22.7 (CH_3CH_2), 25.3, 27.9, 28.8, 28.98, 29.03, 29.2, 29.3, 29.5, 29.58, 29.63, 29.7 (CH_2), 31.9 ($CH_3CH_2CH_2$), 32.7 ($BrCH_2CH_2$), 33.70, 33.74 ($BrCH_2$, SCH_2), 68.3 (OCH_2), 110.0 (C-1), 119.8 (C-5), 121.0 (C-3), 121.2 (C-4), 124.4 (C-8), 134.8 (C-6), 135.0 (C-8'), 135.7 (C-1'), 136.8 (C-4'), 137.4 (C-7), 142.3 (C-5'), 160.2 (C-2), 193.5 (*C*=O) ppm; **FT-IR** (ATR): $\tilde{\nu}$ = 2922 (vs), 2852 (s), 1712 (s), 1601 (m), 1487 (m), 1457 (s), 1436 (m), 1394 (w), 1346 (w), 1287 (m), 1248 (m), 1226 (m), 1204 (w), 1183 (w), 1142 (w), 1101 (w), 1031 (w), 998 (w), 968 (w), 898 (w), 818 (m), 785 (m), 760 (w), 726 (w), 644 (w), 566 (w), 501 (m) cm^{-1}; **MS** (EI): *m/z* = 560.2 (100) $[M]^+$, 478.3 (10), 396.2 (12), 228.0 (18); **HRMS** (EI): calcd. for $[C_{31}H_{43}BrO_2S]^+$ 560.2151, found: 560.2145 $[M]^+$; **elemental analysis**: calcd. (%) for $C_{31}H_{43}BrO_2S$: C 66.53, H 7.74, S 5.73, found: C 66.57, H 7.80, S 5.71; **DSC**: Cr 79 [47.5 kJ mol^{-1}] I (3. H); I 63 [–39.6 kJ mol^{-1}] Cr (3. C).

2-(6-Bromohexyloxy)-7-(tetradecylthio)-9*H*-fluoren-9-one [Br(O6,S14)]. According to GP6, from **13d** (100 mg, 235 µmol), 1,6-dibromohexane (0.11 mL, 172 mg, 705 µmol), K_2CO_3 (97 mg, 705 µmol), MeCN (25 mL); yield: 115 mg, 196 µmol, 83%. **^{1}H-NMR** (400 MHz, $CDCl_3$): δ = 0.88 (t, *J* = 6.7 Hz, 3H, C*H*$_3$), 1.19–1.35 (m, 20H, C*H*$_2$), 1.37–1.47 (m, 2H, $SCH_2CH_2CH_2$), 1.47–1.57 (m, 4H, $OCH_2CH_2CH_2$, $BrCH_2CH_2CH_2$), 1.65 (m$_c$, 2H, SCH_2CH_2), 1.77–1.86 (m, 2H, OCH_2CH_2), 1.86–1.95 (m, 2H, $BrCH_2CH_2$), 2.93 (t, *J* = 7.4 Hz, 2H, SCH_2), 3.43 (t, *J* = 6.8 Hz, 2H, $BrCH_2$), 4.00 (t, *J* = 6.4 Hz, 2H, OCH_2), 6.96 (dd, $J_{3,4}$ = 8.2 Hz, $J_{1,3}$ = 2.4 Hz, 1H, 3-H), 7.16 (d, $J_{1,3}$ = 2.4 Hz, 1H, 1-H), 7.29 (d, $J_{5,6}$ = 7.7 Hz, 1H, 5-H), 7.32–7.39 (m, 2H, 4-H, 6-H), 7.52–7.55 (m, 1H, 8-H) ppm; **^{13}C-NMR** (101 MHz, $CDCl_3$): δ = 14.1 (*C*H$_3$), 22.7 (CH_3CH_2), 25.3, 27.9, 28.8, 28.98, 29.03, 29.2, 29.4, 29.5, 29.6, 29.65, 29.67, 29.69 (*C*H$_2$), 31.9 ($CH_3CH_2CH_2$), 32.7 ($BrCH_2CH_2$), 33.70, 33.74 ($BrCH_2$, SCH_2), 68.3 (OCH_2), 110.0 (C-1), 119.8 (C-5), 121.0 (C-3), 121.2 (C-4), 124.4 (C-8), 134.8 (C-6), 135.0 (C-8'), 135.7 (C-1'), 136.8 (C-4'), 137.4 (C-7), 142.3 (C-5'), 160.2 (C-2), 193.5 (*C*=O) ppm; **FT-IR** (ATR): $\tilde{\nu}$ = 2916 (vs), 2850 (s), 1715 (s), 1601 (m), 1488 (m), 1457 (m), 1434 (m), 1342 (w), 1296 (m), 1250 (s), 1206 (w), 1178 (w), 1139 (w), 1089 (w), 1057 (w), 1030 (w), 996 (w), 972 (w), 902 (w), 820 (m), 781 (m), 757 (w), 719 (w), 644 (w), 498 (m) cm^{-1}; **MS** (EI): *m/z* = 588.2 (100) [M]$^+$, 508.3 (10), 424.2 (10), 228.0 (15); **HRMS** (EI): calcd. for $[C_{33}H_{47}BrO_2S]^+$ 588.2464, found: 588.2466 [M]$^+$; **elemental analysis**: calcd. (%) for $C_{33}H_{47}BrO_2S$: C 67.44, H 8.06, S 5.46, found: C 67.65, H 8.05, S 5.45; **DSC**: Cr 80 [49.6 kJ mol^{-1}] I (3. H); I 65 [–49.4 kJ mol^{-1}] Cr (3. C).

2-(8-Bromooctyloxy)-7-(decylthio)-9*H*-fluoren-9-one [Br(O8,S10)]. According to GP6, from **13b** (100 mg, 271 µmol), 1,8-dibromooctane (0.15 mL, 221 mg, 813 µmol), K_2CO_3 (112 mg, 813 µmol), MeCN (20 mL); yield: 119 mg, 213 µmol, 79%. **^{1}H-NMR** (500 MHz, $CDCl_3$): δ = 0.87 (t, *J* = 6.9 Hz, 3H, C*H*$_3$), 1.21–1.51 (m, 22H, C*H*$_2$), 1.65 (m$_c$, 2H, SCH_2CH_2), 1.79 (m$_c$, 2H, OCH_2CH_2), 1.83–1.91 (m, 2H, $BrCH_2CH_2$), 2.93 (t, *J* = 7.4 Hz, 2H, SCH_2), 3.41 (t, *J* = 6.8 Hz, 2H, $BrCH_2$), 3.99 (t, *J* = 6.5 Hz, 2H, OCH_2), 6.96 (dd, $J_{3,4}$ = 8.2 Hz, $J_{1,3}$ = 2.5 Hz, 1H, 3-H), 7.16 (d, $J_{1,3}$ = 2.4 Hz, 1H, 1-H), 7.28 (d, $J_{5,6}$ = 7.8 Hz, 1H, 5-H), 7.34 (d, $J_{3,4}$ = 8.2 Hz, 1H, 4-H), 7.36 (dd, $J_{5,6}$ = 7.8 Hz, $J_{6,8}$ = 1.8 Hz, 1H, 6-H), 7.53 (d, $J_{6,8}$ = 1.8 Hz, 1H, 8-H) ppm; **^{13}C-NMR** (126 MHz, $CDCl_3$): δ = 14.1 (*C*H$_3$), 22.7 (CH_3CH_2), 25.9, 28.1, 28.7, 28.8, 29.0, 29.1, 29.2, 29.3, 29.49, 29.53 (*C*H$_2$), 31.9 ($CH_3CH_2CH_2$), 32.8 ($BrCH_2CH_2$), 33.7 (SCH_2), 34.0 ($BrCH_2$), 68.5 (OCH_2), 110.0 (C-1), 119.8 (C-5), 121.0 (C-3), 121.2 (C-4), 124.3 (C-8), 134.8 (C-6), 135.0 (C-8'), 135.7 (C-1'), 136.7 (C-4'), 137.4 (C-7), 142.3 (C-5'), 160.2 (C-2), 193.6 (*C*=O) ppm; **FT-IR** (ATR): $\tilde{\nu}$ = 2921 (vs), 2852 (s), 1716 (s), 1601 (m), 1488 (m), 1467 (m), 1458 (m), 1435 (m), 1341 (w), 1300 (m), 1253 (m), 1226 (w), 1208 (w), 1180 (w), 1141 (w), 1090 (w), 1056 (w), 1026 (w), 972 (w), 901 (w), 827 (w), 783 (m), 757 (w), 721 (w), 647 (w), 500 (m) cm^{-1}; **MS** (EI): *m/z* =560.2 (100) [M]$^+$, 368.2 (25), 228.0 (20); **HRMS** (EI): calcd. for $[C_{31}H_{43}BrO_2S]^+$ 560.2151, found: 560.2148 [M]$^+$; **elemental analysis**: calcd. (%) for $C_{31}H_{43}BrO_2S$: C 66.53, H 7.74, found: C 66.29, H 7.61; **DSC**: Cr 69 [40.9 kJ mol^{-1}] I (3. H); I 59 [–7.6 kJ mol^{-1}] SmA 54 [–29.6 kJ mol^{-1}] Cr (3. C).

2-(8-Bromooctyloxy)-7-(dodecylthio)-9*H*-fluoren-9-one [Br(O8,S12)]. According to GP6, from **13c** (350 mg, 883 µmol), 1,8-dibromooctane (0.49 mL, 721 mg, 2.65 mmol), K_2CO_3 (366 mg, 2.65 mmol), MeCN (50 mL); yield: 408 mg, 694 µmol, 79%. **^{1}H-NMR** (400 MHz, $CDCl_3$): δ = 0.88 (t, *J* = 6.8 Hz, 3H, CH_3), 1.20–1.32 (m, 26H, CH_2), 1.65 (m_c, 2H, SCH_2CH_2), 1.75–1.82 (m, 2H, OCH_2CH_2), 1.87 (m_c, 2H, $BrCH_2CH_2$), 2.93 (t, *J* = 7.4 Hz, 2H, SCH_2), 3.41 (t, *J* = 6.8 Hz, 2H, $BrCH_2$), 3.99 (t, *J* = 6.5 Hz, 2H, OCH_2), 6.96 (dd, $J_{3,4}$ = 8.2 Hz, $J_{1,3}$ = 2.5 Hz, 1H, 3-H), 7.16 (d, $J_{1,3}$ = 2.4 Hz, 1H, 1-H), 7.28 (d, $J_{5,6}$ = 7.8 Hz, 1H, 5-H), 7.32–7.38 (m, 2H, 4-H, 6-H), 7.52–7.55 (m, 1H, 8-H) ppm; **^{13}C-NMR** (101 MHz, $CDCl_3$): δ = 14.1 (CH_3), 22.7 (CH_3CH_2), 25.9, 28.1, 28.7, 28.8, 29.0, 29.1, 29.2, 29.3, 29.5, 29.58, 29.63, 29.7 (CH_2), 31.9 ($CH_3CH_2CH_2$), 32.8 ($BrCH_2CH_2$), 33.8 (SCH_2), 33.9 ($BrCH_2$), 68.5 (OCH_2), 110.0 (C-1), 119.8 (C-5), 121.0 (C-3), 121.2 (C-4), 124.4 (C-8), 134.9 (C-6), 135.0 (C-8'), 135.7 (C-1'), 136.7 (C-4'), 137.4 (C-7), 142.3 (C-5'), 160.2 (C-2), 193.6 (*C*=O) ppm; **FT-IR** (ATR): $\tilde{\nu}$ = 2919 (vs), 2850 (s), 1714 (s), 1600 (m), 1488 (m), 1466 (m), 1457 (m), 1434 (m), 1417 (m), 1397 (w), 1341 (w), 1298 (m), 1251 (s), 1226 (m), 1209 (w), 1178 (w), 1140 (w), 1124 (w), 1090 (w), 1055 (w), 1026 (w), 1008 (w), 972 (w), 900 (w), 842 (w), 827 (m), 783 (m), 757 (m), 722 (w), 646 (w), 500 (m) cm^{-1}; **MS** (ESI): *m/z* = 611 $[M+Na]^+$, 588 $[M]^+$, 447, 393; **HRMS** (ESI): calcd. for $[C_{33}H_{47}BrO_2SNa]^+$ 611.2357, found: 611.2344 $[M+Na]^+$; **elemental analysis**: calcd. (%) for $C_{33}H_{47}BrO_2S$: C 67.44, H 8.06, S 5.46, found: C 67.67, H 8.11, S 5.44; **DSC**: Cr_1 65 [–1.5 kJ mol^{-1}] Cr_2 77 [56.1 kJ mol^{-1}] I (3. H); I 63 [–10.8 kJ mol^{-1}] SmA 49 [–34.6 kJ mol^{-1}] Cr (3. C).

2-(8-Bromooctyloxy)-7-(tetradecylthio)-9*H*-fluoren-9-one [Br(O8,S14)]. According to GP6, from **13d** (230 mg, 542 µmol), 1,8-Dibromoctan (0.30 mL, 442 mg, 1.63 mmol), K_2CO_3 (225 mg, 1.63 mmol), MeCN (40 mL); yield: 238 mg, 387 µmol, 71%. **^{1}H-NMR** (400 MHz, $CDCl_3$): δ = 0.88 (t, *J* = 6.7 Hz, 3H, CH_3), 1.19–1.34 (m, 30H, CH_2), 1.65 (m_c, 2H, SCH_2CH_2), 1.74–1.83 (m, 2H, OCH_2CH_2), 1.87 (m_c, 2H, $BrCH_2CH_2$), 2.93 (t, *J* = 7.4 Hz, 2H, SCH_2), 3.41 (t, *J* = 6.8 Hz, 2H, $BrCH_2$), 3.99 (t, *J* = 6.5 Hz, 2H, OCH_2), 6.96 (dd, $J_{3,4}$ = 8.2 Hz, $J_{1,3}$ = 2.5 Hz, 1H, 3-H), 7.17 (d, $J_{1,3}$ = 2.4 Hz, 1H, 1-H), 7.29 (d, $J_{5,6}$ = 7.8 Hz, 1H, 5-H), 7.32–7.39 (m, 2H, 4-H, 6-H), 7.52–7.55 (m, 1H, 8-H) ppm; **^{13}C-NMR** (101 MHz, $CDCl_3$): δ = 14.1 (CH_3), 22.7 (CH_3CH_2), 25.9, 28.1, 28.7, 28.8, 29.0, 29.1, 29.2, 29.4, 29.5, 29.6, 29.65, 29.67, 29.69 (CH_2), 31.9 ($CH_3CH_2CH_2$), 32.8 ($BrCH_2CH_2$), 33.8 (SCH_2), 33.9 ($BrCH_2$), 68.5 (OCH_2), 110.0 (C-1), 119.8 (C-5), 121.0 (C-3), 121.2 (C-4), 124.4 (C-8), 134.9 (C-6), 135.0 (C-8'), 135.7 (C-1'), 136.7 (C-4'), 137.4 (C-7), 142.3 (C-5'), 160.2 (C-2), 193.6 (*C*=O) ppm; **FT-IR** (ATR): $\tilde{\nu}$ = 2919 (vs), 2851 (s), 1715 (s), 1602 (m), 1488 (m), 1468 (m), 1435 (m), 1417 (w), 1299 (m), 1250 (m), 1226 (m), 1206 (w), 1177 (w), 1138 (w), 1090 (w), 1055 (w), 1026 (m), 1008 (w), 901 (w), 820 (m), 781 (m), 757 (w), 720 (w), 647 (w), 498 (m) cm^{-1}; **MS** (ESI): *m/z* = 647, 616 $[M]^+$, 451, 424; **HRMS** (ESI): calcd. for $[C_{35}H_{51}BrO_2S]^+$ 616.2772, found: 616.2717 $[M]^+$; **elemental analysis**: calcd. (%) for $C_{35}H_{51}BrO_2S$: C 68.27, H 8.35, found: C 68.64, H 8.11; **DSC**: Cr 79 [59.9 kJ mol^{-1}] I (3. H); I 61 [–52.1 kJ mol^{-1}] Cr (3. C).

2-(10-Bromodecyloxy)-7-(decylthio)-9*H*-fluoren-9-one [Br(O10,S10)]. According to GP6, from **13b** (400 mg, 1.08 mmol), 1,10-dibromodecane (972 mg, 3.24 mmol), K_2CO_3 (448 mg, 3.24

mmol), MeCN (40 mL); yield: 466 mg, 793 µmol, 73%. **^{1}H-NMR** (500 MHz, $CDCl_3$): δ = 0.87 (t, *J* = 6.9 Hz, 3H, CH_3), 1.19–1.50 (m, 26H, CH_2), 1.65 (m_c, 2H, SCH_2CH_2), 1.75–1.82 (m, 2H, OCH_2CH_2), 1.85 (m_c, 2H, $BrCH_2CH_2$), 2.93 (t, *J* = 7.4 Hz, 2H, SCH_2), 3.41 (t, *J* = 6.9 Hz, 2H, $BrCH_2$), 3.99 (t, *J* = 6.5 Hz, 2H, OCH_2), 6.95 (dd, $J_{3,4}$ = 8.2 Hz, $J_{1,3}$ = 2.5 Hz, 1H, 3-H), 7.16 (d, $J_{1,3}$ = 2.4 Hz, 1H, 1-H), 7.28 (d, $J_{5,6}$ = 7.8 Hz, 1H, 5-H), 7.33 (d, $J_{3,4}$ = 8.2 Hz, 1H, 4-H), 7.35 (dd, $J_{5,6}$ = 7.8 Hz, $J_{6,8}$ = 1.8 Hz, 1H, 6-H), 7.53 (d, $J_{6,8}$ = 1.8 Hz, 1H, 8-H) ppm; **^{13}C-NMR** (126 MHz, $CDCl_3$): δ = 14.1 (CH_3), 22.7 (CH_3CH_2), 26.0, 28.2, 28.76, 28.82, 29.0, 29.1, 29.2, 29.31, 29.32, 29.37, 29.44, 29.5, 29.6 (CH_2), 31.9 ($CH_3CH_2CH_2$), 32.8 ($BrCH_2CH_2$), 33.7 (SCH_2), 34.1 ($BrCH_2$), 68.6 (OCH_2), 110.0 (C-1), 119.8 (C-5), 121.0 (C-3), 121.2 (C-4), 124.3 (C-8), 134.8 (C-6), 135.0 (C-8'), 135.7 (C-1'), 136.7 (C-4'), 137.4 (C-7), 142.3 (C-5'), 160.2 (C-2), 193.6 (*C*=O) ppm; **FT-IR** (ATR): $\tilde{\nu}$ = 2916 (vs), 2850 (s), 1716 (s), 1601 (m), 1489 (m), 1468 (m), 1458 (m), 1435 (m), 1417 (w), 1301 (m), 1253 (m), 1209 (w), 1178 (w), 1140 (w), 1091 (w), 1056 (w), 1029 (w), 1002 (w), 902 (w), 843 (w), 820 (w), 783 (m), 757 (w), 720 (w), 647 (w), 499 (w) cm^{-1}; **MS** (ESI): *m/z* = 657, 589 $[M+H]^+$, 539, 507; **HRMS** (ESI): calcd. for $[C_{33}H_{47}BrO_2SH]^+$ 589.2537, found: 589.2524 $[M+H]^+$; **elemental analysis**: calcd. (%) for $C_{33}H_{47}BrO_2S$: C 67.44, H 8.06, S 5.46, found: C 67.34, H 8.05, S 5.37; **DSC**: Cr 74 [62.8 kJ mol^{-1}] I (3. H); I 57 [–59.9 kJ mol^{-1}] Cr (3. C).

2-(10-Bromodecyloxy)-7-(dodecylthio)-9*H*-fluoren-9-one [Br(O10,S12)]. According to GP6, from **13c** (200 mg, 504 µmol), 1,10-dibromodecane (454 mg, 1.51 mmol), K_2CO_3 (209 mg, 1.51 mmol), MeCN (30 mL); yield: 228 mg, 370 µmol, 73%. **^{1}H-NMR** (400 MHz, $CDCl_3$): δ = 0.87 (t, *J* = 6.7 Hz, 3H, CH_3), 1.19–1.50 (m, 30H, CH_2), 1.65 (m_c, 2H, SCH_2CH_2), 1.73–1.91 (m, 4H, OCH_2CH_2, $BrCH_2CH_2$), 2.93 (t, *J* = 7.4 Hz, 2H, SCH_2), 3.41 (t, *J* = 6.9 Hz, 2H, $BrCH_2$), 3.99 (t, *J* = 6.5 Hz, 2H, OCH_2), 6.96 (dd, $J_{3,4}$ = 8.2 Hz, $J_{1,3}$ = 2.4 Hz, 1H, 3-H), 7.17 (d, $J_{1,3}$ = 2.4 Hz, 1H, 1-H), 7.29 (d, $J_{5,6}$ = 7.8 Hz, 1H, 5-H), 7.32–7.39 (m, 2H, 4-H, 6-H), 7.52–7.56 (m, 1H, 8-H) ppm; **^{13}C-NMR** (101 MHz, $CDCl_3$): δ = 14.1 (CH_3), 22.7 (CH_3CH_2), 26.0, 28.2, 28.7, 28.8, 29.0, 29.1, 29.2, 29.28, 29.34, 29.4, 29.5, 29.57, 29.62, 29.64 (CH_2), 31.9 ($CH_3CH_2CH_2$), 32.8 ($BrCH_2CH_2$), 33.8 (SCH_2), 34.0 ($BrCH_2$), 68.6 (OCH_2), 110.0 (C-1), 119.8 (C-5), 121.0 (C-3), 121.2 (C-4), 124.4 (C-8), 134.9 (C-6), 135.0 (C-8'), 135.7 (C-1'), 136.7 (C-4'), 137.4 (C-7), 142.3 (C-5'), 160.3 (C-2), 193.6 (*C*=O) ppm; **FT-IR** (ATR): $\tilde{\nu}$ = 2919 (vs), 2852 (s), 1716 (s), 1602 (m), 1488 (m), 1470 (m), 1458 (m), 1436 (w), 1418 (w), 1396 (m), 1297 (m), 1248 (m), 1177 (w), 1138 (w), 1090 (w), 1056 (w), 1034 (w), 1003 (w), 971 (w), 891 (w), 820 (w), 780 (m), 759 (w), 719 (w), 645 (w), 497 (w) cm^{-1}; **MS** (ESI): *m/z* = 634, 617 $[M+H]^+$; **HRMS** (ESI): calcd. for $[C_{35}H_{51}BrO_2SH]^+$ 617.2851, found: 617.2818 $[M+H]^+$; **elemental analysis**: calcd. (%) for $C_{35}H_{51}BrO_2S$: C 68.27, H 8.35, S 5.21, found: C 68.38, H 8.35, S 5.06; **DSC**: Cr 78 [65.2 kJ mol^{-1}] I (3. H); I 64 [–65.5 kJ mol^{-1}] Cr (3. C).

2-(10-Bromodecyloxy)-7-(tetradecylthio)-9*H*-fluoren-9-one [Br(O10,S14)]. According to GP6, from **13d** (250 mg, 589 µmol), 1,10-dibromodecane (530 mg, 1.77 mmol), K_2CO_3 (244 mg, 1.77 mmol), MeCN (25 mL); yield: 279 mg, 433 µmol, 74%. **^{1}H-NMR** (400 MHz, $CDCl_3$): δ = 0.88 (t, *J* = 6.7 Hz, 3H, CH_3), 1.20–1.51 (m, 34H, CH_2), 1.65 (m_c, 2H, SCH_2CH_2), 1.73–1.91 (m,

4H, OCH_2CH_2, $BrCH_2CH_2$), 2.93 (t, J = 7.4 Hz, 2H, SCH_2), 3.41 (t, J = 6.9 Hz, 2H, $BrCH_2$), 3.99 (t, J = 6.5 Hz, 2H, OCH_2), 6.96 (dd, $J_{3,4}$ = 8.2 Hz, $J_{1,3}$ = 2.4 Hz, 1H, 3-H), 7.17 (d, $J_{1,3}$ = 2.4 Hz, 1H, 1-H), 7.28 (d, $J_{5,6}$ = 7.8 Hz, 1H, 5-H), 7.32–7.39 (m, 2H, 4-H, 6-H), 7.53 (d, $J_{6,8}$ = 1.7 Hz, 1H, 8-H) ppm; **^{13}C-NMR** (101 MHz, $CDCl_3$): δ = 14.1 (CH_3), 22.7 (CH_3CH_2), 26.0, 28.2, 28.7, 28.8, 29.0, 29.1, 29.2, 29.28, 29.34, 29.36, 29.42, 29.5, 29.57, 29.64, 29.67, 29.69 (CH_2), 31.9 ($CH_3CH_2CH_2$), 32.8 ($BrCH_2CH_2$), 33.8 (SCH_2), 34.0 ($BrCH_2$), 68.6 (OCH_2), 110.0 (C-1), 119.8 (C-5), 121.0 (C-3), 121.2 (C-4), 124.4 (C-8), 134.9 (C-6), 135.0 (C-8'), 135.7 (C-1'), 136.7 (C-4'), 137.4 (C-7), 142.3 (C-5'), 160.3 (C-2), 193.6 (*C*=O) ppm; **FT-IR** (ATR): $\tilde{\nu}$ = 2919 (vs), 2851 (s), 1715 (s), 1602 (m), 1488 (m), 1470 (m), 1435 (w), 1299 (m), 1247 (m), 1138 (w), 1090 (w), 1035 (w), 1004 (w), 820 (w), 780 (m), 758 (w), 719 (w), 649 (w), 497 (w) cm^{-1}; **MS** (ESI): *m/z* = 643 $[M+H]^+$; **HRMS** (ESI): calcd. for $[C_{37}H_{55}BrO_2SH]^+$ 643.3179, found: 643.3105 $[M+H]^+$; **elemental analysis**: calcd. (%) for $C_{37}H_{55}BrO_2S$: C 69.03, H 8.61, S 4.98, found: C 69.13, H 8.62, S 4.90; **DSC**: Cr 79 [119.7 kJ mol^{-1}] I (3. H); I 63 [–12.7 kJ mol^{-1}] SmA 58 [–55.4 kJ mol^{-1}] Cr (3. C).

2-(12-Bromododecyloxy)-7-(decylthio)-9*H*-fluoren-9-one [Br(O12,S10)]. According to GP6, from **13b** (400 mg, 1.08 mmol), 1,12-dibromododecane (1.06 g, 3.24 mmol), K_2CO_3 (448 mg, 3.24 mmol), MeCN (50 mL); yield: 501 mg, 814 µmol, 75%. **^{1}H-NMR** (500 MHz, $CDCl_3$): δ = 0.87 (t, J = 6.9 Hz, 3H, CH_3), 1.20–1.50 (m, 30H, CH_2), 1.65 (m_c, 2H, SCH_2CH_2), 1.75–1.82 (m, 2H, OCH_2CH_2), 1.85 (m_c, 2H, $BrCH_2CH_2$), 2.93 (t, J = 7.4 Hz, 2H, SCH_2), 3.41 (t, J = 6.9 Hz, 2H, $BrCH_2$), 3.99 (t, J = 6.6 Hz, 2H, OCH_2), 6.96 (dd, $J_{3,4}$ = 8.2 Hz, $J_{1,3}$ = 2.5 Hz, 1H, 3-H), 7.16 (d, $J_{1,3}$ = 2.4 Hz, 1H, 1-H), 7.28 (d, $J_{5,6}$ = 7.8 Hz, 1H, 5-H), 7.34 (d, $J_{3,4}$ = 8.2 Hz, 1H, 4-H), 7.36 (dd, $J_{5,6}$ = 7.8 Hz, $J_{6,8}$ = 1.8 Hz, 1H, 6-H), 7.53 (d, $J_{6,8}$ = 1.8 Hz, 1H, 8-H) ppm; **^{13}C-NMR** (126 MHz, $CDCl_3$): δ = 14.1 (CH_3), 22.7 (CH_3CH_2), 26.0, 28.2, 28.79, 28.81, 29.0, 29.1, 29.2, 29.3, 29.4, 29.45, 29.52, 29.6 (CH_2), 31.9 ($CH_3CH_2CH_2$), 32.9 ($BrCH_2CH_2$), 33.7 (SCH_2), 34.1 ($BrCH_2$), 68.6 (OCH_2), 110.0 (C-1), 119.8 (C-5), 121.0 (C-3), 121.2 (C-4), 124.3 (C-8), 134.8 (C-6), 135.0 (C-8'), 135.7 (C-1'), 136.7 (C-4'), 137.4 (C-7), 142.3 (C-5'), 160.3 (C-2), 193.6 (*C*=O) ppm; **FT-IR** (ATR): $\tilde{\nu}$ = 2918 (vs), 2851 (s), 1714 (vs), 1601 (m), 1487 (m), 1469 (m), 1457 (m), 1434 (w), 1417 (w), 1393 (w), 1300 (m), 1290 (m), 1246 (s), 1206 (w), 1174 (w), 1138 (m), 1090 (w), 1055 (w), 1034 (w), 1004 (w), 972 (w), 890 (w), 862 (w), 820 (m), 780 (m), 758 (m), 719 (w), 643 (w), 497 (m) cm^{-1}; **MS** (ESI): *m/z* = 617 $[M+H]^+$, 567, 535; **HRMS** (ESI): calcd. for $[C_{35}H_{51}BrO_2SH]^+$ 617.2851, found: 617.2832 $[M+H]^+$; **elemental analysis**: calcd. (%) for $C_{35}H_{51}BrO_2S$: C 68.27, H 8.35, S 5.21, found: C 68.10, H 8.29, S 5.07; **DSC**: Cr 79 [57.9 kJ mol^{-1}] I (3. H); I 59 [–58.1 kJ mol^{-1}] Cr (3. C).

2-(12-Bromododecyloxy)-7-(dodecylthio)-9*H*-fluoren-9-one [Br(O12,S12)]. According to GP6, from **13c** (450 mg, 1.13 mmol), 1,12-dibromododecane (1.11 g, 3.39 mmol), K_2CO_3 (468 mg, 3.39 mmol), MeCN (50 mL); yield: 509 mg, 791 µmol, 70%. **^{1}H-NMR** (500 MHz, $CDCl_3$): δ = 0.88 (t, J = 6.9 Hz, 3H, CH_3), 1.19–1.50 (m, 34H, CH_2), 1.65 (m_c, 2H, SCH_2CH_2), 1.78 (m_c, 2H, OCH_2CH_2), 1.85 (m_c, 2H, $BrCH_2CH_2$), 2.93 (t, J = 7.4 Hz, 2H, SCH_2), 3.40 (t, J = 6.9 Hz, 2H, $BrCH_2$), 3.99 (t, J = 6.5 Hz, 2H, OCH_2), 6.96 (dd, $J_{3,4}$ = 8.2 Hz, $J_{1,3}$ = 2.5 Hz, 1H, 3-H), 7.16 (d,

$J_{1,3}$ = 2.5 Hz, 1H, 1-H), 7.28 (d, $J_{5,6}$ = 7.8 Hz, 1H, 5-H), 7.34 (d, $J_{3,4}$ = 8.2 Hz, 1H, 4-H), 7.36 (dd, $J_{5,6}$ = 7.8 Hz, $J_{6,8}$ = 1.8 Hz, 1H, 6-H), 7.53 (d, $J_{6,8}$ = 1.8 Hz, 1H, 8-H) ppm; **^{13}C-NMR** (126 MHz, $CDCl_3$): δ = 14.1 (CH_3), 22.7 (CH_3CH_2), 26.0, 28.2, 28.77, 28.79, 29.0, 29.1, 29.2, 29.3, 29.35, 29.42, 29.50, 29.52, 29.58, 29.63, 29.7 (CH_2), 31.9 ($CH_3CH_2CH_2$), 32.8 ($BrCH_2CH_2$), 33.7 (SCH_2), 34.1 ($BrCH_2$), 68.6 (OCH_2), 110.0 (C-1), 119.8 (C-5), 121.0 (C-3), 121.2 (C-4), 124.3 (C-8), 134.8 (C-6), 135.0 (C-8'), 135.7 (C-1'), 136.7 (C-4'), 137.4 (C-7), 142.3 (C-5'), 160.3 (C-2), 193.6 (*C*=O) ppm; **FT-IR** (ATR): $\tilde{\nu}$ = 2918 (vs), 2851 (s), 1715 (s), 1601 (m), 1488 (m), 1470 (m), 1458 (m), 1435 (w), 1418 (w), 1298 (m), 1277 (w), 1250 (m), 1209 (w), 1175 (w), 1138 (w), 1091 (w), 1055 (w), 1029 (w), 1003 (w), 972 (w), 891 (w), 861 (w), 820 (w), 780 (m), 759 (m), 718 (w), 649 (w), 497 (w) cm^{-1}; **MS** (ESI): *m/z* = 645 $[M+H]^+$; **HRMS** (ESI): calcd. for $[C_{37}H_{55}BrO_2SH]^+$ 645.3164, found: 645.3153 $[M+H]^+$; **elemental analysis**: calcd. (%) for $C_{37}H_{55}BrO_2S$: C 69.03, H 8.61, S 4.98, found: C 69.27, H 8.57, S 4.83; **DSC**: Cr 78 [67.1 kJ mol^{-1}] I (3. H); I 65 [–73.7 kJ mol^{-1}] Cr (3. K).

2-(12-Bromododecyloxy)-7-(tetradecylthio)-9*H*-fluoren-9-one [Br(O12,S14)]. According to GP6, from **13d** (250 mg, 589 µmol), 1,12-dibromododecane (580 mg, 1.77 mmol), K_2CO_3 (244 mg, 1.77 mmol), MeCN (30 mL); yield: 305 mg, 454 µmol, 77%. **^{1}H-NMR** (400 MHz, $CDCl_3$): δ = 0.88 (t, *J* = 6.7 Hz, 3H, CH_3), 1.19–1.50 (m, 38H, CH_2), 1.65 (m_c, 2H, SCH_2CH_2), 1.73–1.82 (m, 2H, OCH_2CH_2), 1.85 (m_c, 2H, $BrCH_2CH_2$), 2.93 (t, *J* = 7.4 Hz, 2H, SCH_2), 3.40 (t, *J* = 6.9 Hz, 2H, $BrCH_2$), 3.99 (t, *J* = 6.5 Hz, 2H, OCH_2), 6.96 (dd, $J_{3,4}$ = 8.2 Hz, $J_{1,3}$ = 2.4 Hz, 1H, 3-H), 7.16 (d, $J_{1,3}$ = 2.4 Hz, 1H, 1-H), 7.28 (d, $J_{5,6}$ = 7.8 Hz, 1H, 5-H), 7.31–7.39 (m, 2H, 4-H, 6-H), 7.53 (d, $J_{6,8}$ = 1.7 Hz, 1H, 8-H) ppm; **^{13}C-NMR** (101 MHz, $CDCl_3$): δ = 14.1 (CH_3), 22.7 (CH_3CH_2), 26.0, 28.2, 28.77, 28.79, 29.0, 29.1, 29.3, 29.36, 29.42, 29.5, 29.6, 29.65, 29.67, 29.69 (CH_2), 31.9 ($CH_3CH_2CH_2$), 32.9 ($BrCH_2CH_2$), 33.7 (SCH_2), 34.0 ($BrCH_2$), 68.6 (OCH_2), 110.0 (C-1), 119.8 (C-5), 121.0 (C-3), 121.1 (C-4), 124.4 (C-8), 134.8 (C-6), 135.0 (C-8'), 135.7 (C-1'), 136.7 (C-4'), 137.4 (C-7), 142.3 (C-5'), 160.3 (C-2), 193.6 (*C*=O) ppm; **FT-IR** (ATR): $\tilde{\nu}$ = 2917 (vs), 2851 (s), 1715 (s), 1602 (m), 1488 (m), 1471 (m), 1458 (m), 1435 (w), 1394 (w), 1299 (m), 1250 (m), 1208 (w), 1176 (w), 1138 (w), 1091 (w), 1055 (w), 1029 (w), 1002 (w), 972 (w), 890 (w), 861 (w), 820 (w), 780 (m), 758 (w), 718 (w), 648 (w), 497 (w) cm^{-1}; **MS** (ESI): *m/z* = 673 $[M+H]^+$, 439, 371; **HRMS** (ESI): calcd. for $[C_{39}H_{59}BrO_2SH]^+$ 673.3478, found: 673.3433 $[M+H]^+$; **elemental analysis**: calcd. (%) for $C_{39}H_{59}BrO_2S$: C 69.72, H 8.85, S 4.77, found: C 69.59, H 8.86, S 4.64; **DSC**: Cr 82 [81.9 kJ mol^{-1}] I (3. H); I 76 [–75.9 kJ mol^{-1}] Cr (3. C).

General Procedure for the Preparation of Imidazolium bromides (GP7)[11]

To a solution of the appropriate **Br(On,Ym)** (184 µmol) in abs. DMF (10 mL) under N_2 atmosphere was added the respective **16a**–**c** (276 µmol), and the reaction mixture was stirred for 16 h at 80 °C. The solvent was then removed under reduced pressure and the residue purified by chromatography on HBr-SiO_2 with CH_2Cl_2/MeOH (20:1). **ImMe(On,Sm)Br** and **ImEt(On,Sm)Br** were additionally recrystallized from acetone (20 mL). Drying under high vacuum gave products **ImR(On,Ym)Br** as orange solids.

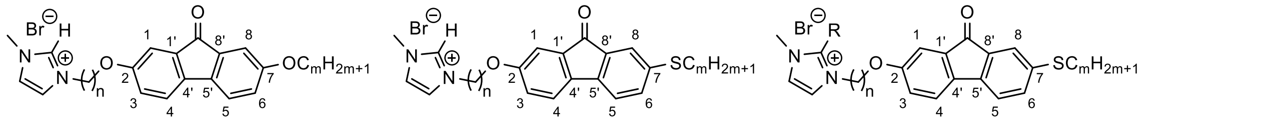

Im(On,Om)Br **Im(On,Sm)Br** **ImMe(On,Om)Br** (R=Me), **ImEt(On,Om)Br** (R=Et)

3-(4-{[7-(Dodecyloxy)-9-oxo-9*H*-fluoren-2-yl]oxy}butyl)-1-methyl-1*H*-imidazol-3-ium bromide [Im(O4,O12)Br]. According to GP7, from **Br(O4,O12)** (92 mg, 178 µmol), **16a** (21 µL, 22 mg, 267 µmol), abs. DMF (8 mL); yield: 96 mg, 153 µmol, 96%. **^{1}H-NMR** (300 MHz, $CDCl_3$): δ = 0.81–0.93 (m, 3H, C*H*$_3$), 1.18–1.53 (m, 18H, C*H*$_2$), 1.77 (m$_c$, 2H, OCH$_2$C*H*$_2$), 1.83–1.96 (m, 2H, NCH$_2$CH$_2$C*H*$_2$), 2.10–2.24 (m, 2H, NCH$_2$C*H*$_2$), 3.96 (t, J = 6.6 Hz, 2H, OC*H*$_2$), 4.04 (t, J = 5.8 Hz, 2H, NCH$_2$CH$_2$CH$_2$C*H*$_2$O), 4.11 (s, 3H, NC*H*$_3$), 4.49 (t, J = 7.3 Hz, 2H, NC*H*$_2$), 6.88–6.95 (m, 2H, 3-H, 6-H), 7.05 (d, $J_{1,3}$ = 2.4 Hz, 1H, 1-H), 7.11 (d, $J_{6,8}$ = 2.4 Hz, 1H, 8-H), 7.22–7.29 (m, 2H, 4-H, 5-H), 7.33–7.37 (m, 1H, Im), 7.40–7.45 (m, 1H, Im), 10.60 (s, 1H, NC*H*N) ppm; **^{13}C-NMR** (75 MHz, $CDCl_3$): δ = 14.1 (*C*H$_3$), 22.7 (CH$_3$*C*H$_2$), 25.8, 26.0, 27.4, 29.2, 29.36, 29.38, 29.57, 29.60, 29.64, 29.7 (*C*H$_2$), 31.9 (CH$_3$CH$_2$*C*H$_2$), 36.8 (N*C*H$_3$), 49.9 (N*C*H$_2$), 67.5, 68.6 (O*C*H$_2$), 110.2 (C-8), 110.5 (C-1), 120.3, 120.6, 120.9 (C-3, C-4, C-5, C-6), 121.9 (Im), 123.1 (Im), 135.85, 135.93 (C-1', C-8'), 137.2 (C-5'), 137.7 (C-4'), 138.1 (N*C*HN), 158.8 (C-2), 159.6 (C-7), 193.8 (*C*=O) ppm; **FT-IR** (ATR): $\tilde{\nu}$ = 3471 (w), 3409 (w), 3093 (w), 2917 (s), 2850 (m), 1717 (s), 1613 (w), 1588 (w), 1573 (w), 1461 (vs), 1398 (w), 1287 (vs), 1252 (w), 1221 (s), 1167 (m), 1048 (m), 997 (w), 971 (w), 940 (w), 888 (w), 820 (w), 806 (m), 784 (m), 754 (w), 623 (w), 513 (m) cm^{-1}; **MS** (ESI): *m/z* = 517 [M]$^+$; **HRMS** (ESI): calcd. for $[C_{33}H_{45}N_2O_3]^+$ 517.3425, found: 517.3392 [M]$^+$; **elemental analysis**: calcd. (%) for $C_{33}H_{45}BrN_2O_3$: C 66.32, H 7.59, N 4.69, found: C 64.13, H 7.70, N 4.86; **DSC**: Cr 90 [26.0 kJ mol^{-1}] SmA 277 [2.4 kJ mol^{-1}] I (1. H).

3-(4-{[7-(Tetradecyloxy)-9-oxo-9*H*-fluoren-2-yl]oxy}butyl)-1-methyl-1*H*-imidazol-3-ium bromide [Im(O4,O14)Br]. According to GP7, from **Br(O4,O14)** (100 mg, 184 µmol), **16a** (22 µL, 23 mg, 276 µmol), abs. DMF (10 mL); yield: 96 mg, 153 µmol, 83%. **^{1}H-NMR** (500 MHz, $CDCl_3$): δ = 0.88 (t, J = 6.9 Hz, 3H, C*H*$_3$), 1.19–1.39 (m, 20H, C*H*$_2$), 1.39–1.50 (m, 2H, O(CH$_2$)$_2$C*H*$_2$), 1.77 (m$_c$, 2H, OCH$_2$C*H*$_2$), 1.84–1.94 (m, 2H, NCH$_2$CH$_2$C*H*$_2$), 2.18 (m$_c$, 2H, NCH$_2$C*H*$_2$), 3.96 (t, J = 6.7 Hz, 2H, OC*H*$_2$), 4.04 (t, J = 5.8 Hz, 2H, NCH$_2$CH$_2$CH$_2$C*H*$_2$O), 4.11 (s, 3H, NC*H*$_3$), 4.49 (t, J = 7.4 Hz, 2H, NC*H*$_2$), 6.89–6.94 (m, 2H, 3-H, 6-H), 7.05 (d, $J_{1,3}$ = 2.4 Hz, 1H, 1-H), 7.11 (d, $J_{6,8}$ = 2.5 Hz, 1H, 8-H), 7.23–7.29 (m, 2H, 4-H, 5-H), 7.36–7.39 (m, 1H, Im), 7.43–7.46 (m, 1H, Im), 10.65 (s, 1H, NC*H*N) ppm; **^{13}C-NMR** (126 MHz, $CDCl_3$): δ = 14.1 (*C*H$_3$), 22.7 (CH$_3$*C*H$_2$), 25.9, 26.0, 27.4, 29.2, 29.36, 29.38, 29.58, 29.61, 29.66, 29.68, 29.70 (*C*H$_2$), 31.9 (CH$_3$CH$_2$*C*H$_2$), 36.9 (N*C*H$_3$), 49.9 (N*C*H$_2$), 67.5 (NCH$_2$CH$_2$CH$_2$*C*H$_2$O), 68.6 (O*C*H$_2$), 110.2 (C-8), 110.5 (C-1), 120.4, 120.6, 120.9 (C-3, C-4, C-5, C-6) 121.9 (Im), 123.2 (Im), 135.8, 135.9 (C-1', C-8'), 137.1 (C-5'), 137.7 (C-4'), 138.1 (N*C*HN), 158.8 (C-2), 159.6 (C-7), 193.7 (*C*=O) ppm; **FT-IR** (ATR): $\tilde{\nu}$ = 3414 (w), 3061 (w), 2953 (w), 2917 (vs), 2851 (m), 1715 (s), 1610 (w), 1590 (w), 1573 (w), 1465 (s), 1403 (w), 1290 (s), 1247 (w), 1222 (m), 1168 (w), 1039 (m), 1004 (w), 971 (w), 889 (w), 811 (m), 799 (w), 784 (m), 720 (w), 646 (w), 514 (w) cm^{-1}; **MS** (ESI): *m/z* = 545 [M]$^+$; **HRMS** (ESI): calcd. for $[C_{35}H_{49}N_2O_3]^+$ 545.3738, found: 545.3729 [M]$^+$; **elemental**

analysis: calcd. (%) for $C_{35}H_{49}BrN_2O_3$: C 67.19, H 7.89, N 4.48, found: C 65.67, H 7.67, N 4.17; **DSC**: Cr 97 [33.0 kJ mol^{-1}] SmA 294 [18.1 kJ mol^{-1}] I (1. H).

3-(4-{[7-(Hexadecyloxy)-9-oxo-9*H*-fluoren-2-yl]oxy}butyl)-1-methyl-1*H*-imidazol-3-ium bromide [Im(O4,O16)Br]. According to GP7, from **Br(O4,O16)** (100 mg, 175 µmol), **16a** (21 µL, 22 mg, 263 µmol), abs. DMF (5 mL); yield: 105 mg, 161 µmol, 92%. **^{1}H-NMR** (400 MHz, $CDCl_3$): δ = 0.88 (t, *J* = 6.7 Hz, 3H, CH_3), 1.18–1.39 (m, 24H, CH_2), 1.39–1.50 (m, 2H, $O(CH_2)_2CH_2$), 1.77 (m_c, 2H, OCH_2CH_2), 1.85–1.93 (m, 2H, $NCH_2CH_2CH_2$), 2.18 (m_c, 2H, NCH_2CH_2), 3.96 (t, *J* = 6.7 Hz, 2H, OCH_2), 4.04 (t, *J* = 5.8 Hz, 2H, $NCH_2CH_2CH_2CH_2O$), 4.11 (s, 3H, NCH_3), 4.49 (t, *J* = 7.3 Hz, 2H, NCH_2), 6.90–6.95 (m, 2H, 3-H, 6-H), 7.05 (d, $J_{1,3}$ = 2.4 Hz, 1H, 1-H), 7.11 (d, $J_{6,8}$ = 2.4 Hz, 1H, 8-H), 7.23–7.29 (m, 2H, 4-H, 5-H), 7.32–7.36 (m, 1H, Im), 7.39–7.42 (m, 1H, Im), 10.70 (s, 1H, NC*H*N) ppm; **^{13}C-NMR** (101 MHz, $CDCl_3$): δ = 14.1 (CH_3), 22.7 (CH_3CH_2), 25.9, 26.0, 27.4, 29.2, 29.37, 29.38, 29.58, 29.61, 29.67, 29.69, 29.70 (CH_2), 31.9 ($CH_3CH_2CH_2$), 36.8 (NCH_3), 49.9 (NCH_2), 67.5 ($NCH_2CH_2CH_2CH_2O$), 68.6 (OCH_2), 110.3 (C-8), 110.6 (C-1), 120.4, 120.6, 120.9 (C-3, C-4, C-5, C-6), 121.8 (Im), 123.1 (Im), 135.9, 136.0 (C-1', C-8'), 137.2 (C-5'), 137.8 (C-4'), 138.2 (N*C*HN), 158.9 (C-2), 159.6 (C-7), 193.7 (*C*=O) ppm; **FT-IR** (ATR): $\tilde{\nu}$ =3414 (w), 3074 (w), 2953 (m), 2916 (vs), 2850 (s), 1715 (s), 1612 (w), 1590 (w), 1465 (s), 1403 (w), 1291 (s), 1249 (w), 1223 (m), 1170 (m), 1042 (m), 1000 (w), 971 (w), 889 (w), 812 (m), 800 (m), 784 (m), 719 (w), 620 (w), 515 (w) cm^{-1}; **MS** (ESI): *m/z* = 573 $[M]^+$, 491, 447, 393; **HRMS** (ESI): calcd. for $[C_{37}H_{53}N_2O_3]^+$ 573.4051, found: 573.4005 $[M]^+$; **elemental analysis**: calcd. (%) for $C_{37}H_{53}BrN_2O_3$: C 67.98, H 8.17, N 4.29, found: C 65.29, H 7.85 N 4.21; **DSC**: Cr 94 [43.7 kJ mol^{-1}] SmA 281 [0.7 kJ mol^{-1}] I (1. H).

3-(6-{[7-(Octyloxy)-9-oxo-9*H*-fluoren-2-yl]oxy}hexyl)-1-methyl-1*H*-imidazol-3-ium bromide [Im(O6,O8)Br]. According to GP7, from **Br(O6,O8)** (140 mg, 287 µmol), **16a** (34 µL, 35 mg, 431 µmol), abs. DMF (5 mL); yield: 117 mg, 205 µmol, 71%. **^{1}H-NMR** (400 MHz, $CDCl_3$): δ = 0.85–0.93 (m, 3H, CH_3), 1.21–1.39 (m, 8H, CH_2), 1.39–1.59 (m, 6H, $OCH_2CH_2CH_2$, $NCH_2CH_2CH_2$), 1.72–1.83 (m, 4H, OCH_2CH_2), 1.98 (m_c, 2H, NCH_2CH_2), 3.91–4.02 (m, 4H, OCH_2), 4.12 (s, 3H, NCH_3), 4.37 (t, *J* = 7.4 Hz, 2H, NCH_2), 6.88–6.95 (m, 2H, 3-H, 6-H), 7.06 (d, $J_{1,3}$ = 2.4 Hz, 1H, 1-H), 7.12 (d, $J_{6,8}$ = 2.4 Hz, 1H, 8-H), 7.23–7.29 (m, 2H, 4-H, 5-H), 7.36–7.40 (m, 1H, Im), 7.40–7.44 (m, 1H, Im), 10.59 (s, 1H, NC*H*N) ppm; **^{13}C-NMR** (101 MHz, $CDCl_3$): δ = 14.1 (CH_3), 22.7 (CH_3CH_2), 25.5, 25.9, 26.0, 28.8, 29.18, 29.23, 29.3, 30.2 (CH_2), 31.8 ($CH_3CH_2CH_2$), 36.8 (NCH_3), 50.1 (NCH_2), 68.1, 68.6 (OCH_2), 110.3, 110.4 (C-1, C-8), 120.56, 120.58, 120.8 (C-3, C-4, C-5, C-6), 121.8 (Im), 123.3 (Im), 135.88, 135.91 (C-1', C-8'), 137.3, 137.5 (C-4', C-5'), 138.0 (N*C*HN), 159.3 (C-2), 159.6 (C-7), 193.9 (*C*=O) ppm; **FT-IR** (ATR): $\tilde{\nu}$ = 3412 (w), 3066 (w), 2924 (s), 2855 (m), 1711 (s), 1607 (m), 1592 (w), 1572 (w), 1460 (vs), 1445 (s), 1392 (w), 1285 (s), 1247 (m), 1222 (s), 1167 (m), 1137 (w), 1039 (m), 996 (w), 970 (w), 892 (w), 818 (m), 788 (m), 647 (w), 621 (w), 517 (w) cm^{-1}; **MS** (ESI): *m/z* = 489 $[M]^+$; **HRMS** (ESI): calcd. for $[C_{31}H_{41}N_2O_3]^+$ 489.3112, found: 489.3108 $[M]^+$; **elemental analysis**: calcd. (%) for $C_{31}H_{41}BrN_2O_3$: C 65.37, H 7.26, N 4.92, found: C 64.42, H 6.98 N 4.93; **DSC**: Cr 72 [33.9 kJ mol^{-1}] SmC 88 [via POM] SmA 237 [1.7 kJ mol^{-1}] I (1. H).

3-(6-{[7-(Decyloxy)-9-oxo-9*H*-fluoren-2-yl]oxy}hexyl)-1-methyl-1*H*-imidazol-3-ium bromide [Im(O6,O10)Br]. According to GP7, from **Br(O6,O10)** (90 mg, 175 µmol), **16a** (21 µL, 22 mg, 263 µmol), abs. DMF (3 mL); yield: 61 mg, 102 µmol, 58%. **^{1}H-NMR** (500 MHz, $CDCl_3$): δ = 0.88 (t, *J* = 6.9 Hz, 3H, CH_3), 1.19–1.39 (m, 12H, CH_2), 1.39–1.50 (m, 4H, $OCH_2CH_2CH_2$,), 1.50–1.60 (m, 2H, $NCH_2CH_2CH_2$), 1.73–1.84 (m, 4H, OCH_2CH_2), 1.98 (m_c, 2H, NCH_2CH_2), 3.92–4.02 (m, 4H, OCH_2), 4.12 (s, 3H, NCH_3), 4.37 (t, *J* = 7.4 Hz, 2H, NCH_2), 6.90–6.94 (m, 2H, 3-H, 6-H), 7.07 (d, $J_{1,3}$ = 2.4 Hz, 1H, 1-H), 7.12 (d, $J_{6,8}$ = 2.4 Hz, 1H, 8-H), 7.25–7.29 (m, 2H, 4-H, 5-H), 7.31–7.34 (m, 1H, Im), 7.34–7.36 (m, 1H, Im), 10.63 (s, 1H, NC*H*N) ppm; **^{13}C-NMR** (126 MHz, $CDCl_3$): δ = 14.1 (CH_3), 22.7 (CH_3CH_2), 25.5, 25.9, 26.0, 28.8, 29.2, 29.3, 29.4, 29.55, 29.57, 30.2 (CH_2), 31.9 ($CH_3CH_2CH_2$), 36.8 (NCH_3), 50.1 (NCH_2), 68.1, 68.6 (OCH_2), 110.2 (C-8), 110.4 (C-1), 120.56, 120.58, 120.9 (C-3, C-4, C-5, C-6), 121.6 (Im), 123.1 (Im), 135.88, 135.91 (C-1', C-8'), 137.3, 137.5 (C-4', C-5'), 138.1 (N*C*HN), 159.2 (C-2), 159.5 (C-7), 193.9 (*C*=O) ppm; **FT-IR** (ATR): $\tilde{\nu}$ = 3408 (w), 3065 (w), 2923 (s), 2853 (m), 1712 (s), 1607 (m), 1592 (m), 1572 (w), 1462 (vs), 1445 (s), 1392 (m), 1286 (s), 1247 (m), 1223 (s), 1168 (m), 1137 (w), 1038 (m), 1003 (w), 972 (w), 892 (w), 818 (m), 788 (m), 736 (w), 647 (w), 621 (w), 518 (w) cm^{-1}; **MS** (ESI): *m/z* = 417 $[M]^+$; **HRMS** (ESI): calcd. for $[C_{33}H_{45}N_2O_3]^+$ 517.3452, found: 517.3420 $[M]^+$; **elemental analysis**: calcd. (%) for $C_{33}H_{45}BrN_2O_3$: C 66.32, H 7.59, N 4.69, found: C 63.83, H 7.47 N 4.47; **DSC**: Cr_1 71 [4.1 kJ mol^{-1}] Cr_2 84 [28.9 kJ mol^{-1}] SmC 94 [via POM] SmA 258 [1.3 kJ mol^{-1}] I (1. H).

3-(6-{[7-(Dodecyloxy)-9-oxo-9*H*-fluoren-2-yl]oxy}hexyl)-1-methyl-1*H*-imidazol-3-ium bromide [Im(O6,O12)Br]. According to GP7, from **Br(O6,O12)** (100 mg, 184 µmol), **16a** (22 µL, 23 mg, 276 µmol), abs. DMF (5 mL); yield: 80 mg, 128 µmol, 70%. **^{1}H-NMR** (400 MHz, $CDCl_3$): δ = 0.88 (t, *J* = 6.7 Hz, 3H, CH_3), 1.19–1.39 (m, 16H, CH_2), 1.39–1.49 (m, 4H, $OCH_2CH_2CH_2$), 1.49–1.59 (m, 2H, $NCH_2CH_2CH_2$), 1.72–1.84 (m, 4H, OCH_2CH_2), 1.98 (m_c, 2H, NCH_2CH_2), 3.91–4.03 (m, 4H, OCH_2), 4.12 (s, 3H, NCH_3), 4.37 (t, *J* = 7.4 Hz, 2H, NCH_2), 6.88–6.95 (m, 2H, 3-H, 6-H), 7.07 (d, $J_{1,3}$ = 2.4 Hz, 1H, 1-H), 7.12 (d, $J_{6,8}$ = 2.4 Hz, 1H, 8-H), 7.24–7.29 (m, 2H, 4-H, 5-H), 7.32–7.35 (m, 1H, Im), 7.35–7.39 (m, 1H, Im), 10.64 (s, 1H, NC*H*N) ppm; **^{13}C-NMR** (101 MHz, $CDCl_3$): δ = 14.1 (CH_3), 22.7 (CH_3CH_2), 25.5, 25.9, 26.0, 28.8, 29.2, 29.35, 29.37, 29.57, 29.60, 29.64, 29.7, 30.2 (CH_2), 31.9 ($CH_3CH_2CH_2$), 36.8 (NCH_3), 50.1 (NCH_2), 68.1, 68.6 (OCH_2), 110.3 (C-8), 110.4 (C-1), 120.57, 120.59, 120.9 (C-3, C-4, C-5, C-6), 121.7 (Im), 123.2 (Im), 135.90, 135.93 (C-1', C-8'), 137.3, 137.5 (C-4', C-5'), 138.2 (N*C*HN), 159.3 (C-2), 159.6 (C-7), 193.9 (*C*=O) ppm; **FT-IR** (ATR): $\tilde{\nu}$ = 3409 (w), 3068 (w), 2921 (s), 2852 (m), 1711 (s), 1607 (w), 1592 (w), 1572 (w), 1461 (vs), 1392 (w), 1284 (s), 1247 (m), 1221 (s), 1167 (m), 1137 (w), 1038 (m), 998 (w), 970 (w), 893 (w), 817 (m), 798 (m), 788 (m), 735 (w), 647 (w), 621 (w), 518 (w) cm^{-1}; **MS** (ESI): *m/z* = 545 $[M]^+$, 428, 123; **HRMS** (ESI): calcd. for $[C_{35}H_{49}N_2O_3]^+$ 545.3738, found: 545.3730 $[M]^+$; **elemental analysis**: calcd. (%) for $C_{35}H_{49}BrN_2O_3$: C 67.19, H 7.89, N 4.48, gef.: C 65.70, H 7.69 N 4.21; **DSC**: Cr 83 [47.0 kJ mol^{-1}] SmC 105 [via POM] SmA 263 [1.3 kJ mol^{-1}] I (1. H).

3-(6-{[7-(Tetradecyloxy)-9-oxo-9*H*-fluoren-2-yl]oxy}hexyl)-1-methyl-1*H*-imidazol-3-ium bromide [Im(O6,O14)Br]. According to GP7, from **Br(O6,O14)** (130 mg, 227 µmol), **16a** (27 µL, 28 mg, 341 µmol), abs. DMF (10 mL); yield: 130 mg, 199 µmol, 88%. **^{1}H-NMR** (400 MHz, $CDCl_3$): δ = 0.83–0.93 (m, 3H, CH_3), 1.20–1.39 (m, 18H, CH_2), 1.39–1.60 (m, 6H, $O(CH_2)_2CH_2$, $NCH_2CH_2CH_2$), 1.72–1.84 (m, 4H, OCH_2CH_2), 1.98 (m_c, J = 7.4 Hz, 2H, NCH_2CH_2), 3.94–4.00 (m, 4H, OCH_2), 4.12 (s, 3H, NCH_3), 4.37 (t, J = 7.4 Hz, 2H, NCH_2), 6.89–6.95 (m, 2H, 3-H, 6-H), 7.07 (d, $J_{1,3}$ = 2.4 Hz, 1H, 1-H), 7.13 (d, $J_{6,8}$ = 2.4 Hz, 1H, 8-H), 7.24–7.29 (m, 3H, 4-H, 5-H, Im), 7.29–7.32 (m, 1H, Im), 10.76 (s, 1H, NC*H*N) ppm; **^{13}C-NMR** (101 MHz, $CDCl_3$): δ = 14.1 (CH_3), 22.7 (CH3CH_2), 25.5, 25.9, 26.0, 28.8, 29.2, 29.4, 29.57, 29.60, 29.66, 29.68, 29.70, 30.2 (CH_2), 31.9 ($CH_3CH_2CH_2$), 36.8 (NCH_3), 50.2 (NCH_2), 68.1, 68.6 (OCH_2), 110.3 (C-8), 110.4 (C-1), 120.57, 120.60, 120.9 (C-3, C-4, C-5, C-6), 121.6 (Im), 123.0 (Im), 136.0 (C-1', C-8'), 137.5 (C-4', C-5'), 138.4 (N*C*HN), 159.3 (C-2), 159.6 (C-7), 193.9 (*C*=O) ppm; **FT-IR** (ATR): $\tilde{\nu}$ = 3412 (w), 3076 (w), 2918 (vs), 2851 (s), 1715 (s), 1611 (w), 1591 (w), 1574 (w), 1465 (s), 1401 (w), 1289 (s), 1248 (m), 1223 (m), 1169 (m), 1142 (w), 1039 (m), 1001 (w), 971 (w), 889 (w), 810 (m), 800 (m), 783 (m), 719 (w), 647 (w), 620 (w), 514 (w) cm^{-1}; **MS** (ESI): *m/z* = 573 $[M]^+$; **HRMS** (ESI): calcd. for $[C_{37}H_{53}N_2O_3]^+$ 573.4051, found: 573.4050 $[M]^+$; **elemental analysis**: calcd. (%) for $C_{37}H_{53}BrN_2O_3$: C 67.98, H 8.17, N 4.29, found: C 65.72, H 8.06 N 4.23; **DSC**: Cr_1 38 [6.2 kJ mol^{-1}] Cr_2 79 [45.0 kJ mol^{-1}] SmC 104 [via POM] SmA 266 [17.4 kJ mol^{-1}] I (1. H).

3-(6-{[7-(Hexadecyloxy)-9-oxo-9*H*-fluoren-2-yl]oxy}hexyl)-1-methyl-1*H*-imidazol-3-ium bromide [Im(O6,O16)Br]. According to GP7, from **Br(O6,O16)** (100 mg, 167 µmol), **16a** (20 µL, 21 mg, 251 µmol), abs. DMF (4 mL); yield: 85 mg, 125 µmol, 75%. **^{1}H-NMR** (700 MHz, $CDCl_3$): δ = 0.88 (t, J = 7.0 Hz, 3H, CH_3), 1.18–1.39 (m, 24H, CH_2), 1.40–1.49 (m, 4H, $OCH_2CH_2CH_2$), 1.50–1.58 (m, 2H, $NCH_2CH_2CH_2$), 1.73–1.82 (m, 4H, OCH_2CH_2), 1.98 (m_c, J = 7.4 Hz, 2H, NCH_2CH_2), 3.94–4.00 (m, 4H, OCH_2), 4.12 (s, 3H, NCH_3), 4.37 (t, J = 7.4 Hz, 2H, NCH_2), 6.89–6.94 (m, 2H, 3-H, 6-H), 7.05–7.09 (m, 1H, 1-H), 7.10–7.14 (m, 1H, 8-H), 7.24–7.30 (m, 2H, 4-H, 5-H), 7.33–7.42 (m, 2H, Im), 10.55–10.64 (m, 1H, NC*H*N) ppm; **^{13}C-NMR** (176 MHz, $CDCl_3$): δ = 14.1 (CH_3), 22.7 (CH_3CH_2), 25.5, 25.9, 26.0, 28.8, 29.2, 29.37, 29.38, 29.57, 29.60, 29.66, 29.68, 29.70, 30.2 (CH_2), 31.9 ($CH_3CH_2CH_2$), 36.8 (NCH_3), 50.1 (NCH_2), 68.1, 68.6 (OCH_2), 110.2 (C-8), 110.4 (C-1), 120.6, 120.6, 120.8 (C-3, C-4, C-5, C.6), 121.7 (Im), 123.2 (Im), 135.9, 135.9 (C-1', C-8'), 137.3, 137.5 (C-4', C-5'), 138.0 (N*C*HN), 159.2 (C-2), 159.5 (C-7), 193.9 (*C*=O) ppm; **FT-IR** (ATR): $\tilde{\nu}$ = 3409 (w), 3060 (w), 2916 (s), 2849 (s), 1713 (s), 1611 (w), 1590 (w), 1572 (w), 1463 (vs), 1402 (w), 1388 (w), 1353 (w), 1288 (vs), 1249 (w), 1221 (s), 1170 (m), 1141 (w), 1106 (w), 1041 (w), 1016 (w), 996 (w), 970 (w), 923 (w), 890 (w), 811 (m), 799 (m), 785 (m), 719 (s), 642 (w), 621 (w), 515 (w) cm^{-1}; **MS** (ESI): *m/z* = 601 $[M]^+$; **HRMS** (ESI): calcd. for $[C_{39}H_{57}N_2O_3]^+$ 601.4364, found: 601.4380 $[M]^+$; **elemental analysis**: calcd. (%) for $C_{39}H_{57}BrN_2O_3$: C 68.70, H 8.43, N 4.11, found: C 67.19, H 8.05 N 4.12; **DSC**: Cr 81 [39.0 kJ mol^{-1}] SmC 113 [via POM] SmA 273 [0.7 kJ mol^{-1}] I (1. H).

3-(8-{[7-(Decyloxy)-9-oxo-9*H*-fluoren-2-yl]oxy}octyl)-1-methyl-1*H*-imidazol-3-ium bromide [Im(O8,O10)Br]. According to GP7, from **Br(O8,O10)** (45 mg, 83 µmol), **16a** (9 µL, 10 mg,

125 µmol), abs. DMF (5 mL); yield: 42 mg, 67 µmol, 81%. **^{1}H-NMR** (500 MHz, $CDCl_3$): δ = 0.88 (t, *J* = 6.9 Hz, 3H, C*H*$_3$), 1.19–1.40 (m, 18H, C*H*$_2$), 1.40–1.51 (m, 4H, OCH_2CH_2C*H*$_2$), 1.72–1.83 (m, 4H, OCH_2C*H*$_2$), 1.89–2.00 (m, 2H, NCH_2C*H*$_2$), 3.93–4.02 (m, 4H, OC*H*$_2$), 4.13 (s, 3H, NC*H*$_3$), 4.34 (t, *J* = 7.5 Hz, 2H, NC*H*$_2$), 6.90–6.94 (m, 2H, 3-H, 6-H), 7.10 (d, $J_{1,3}$ = 2.4 Hz, 1H, 1-H), 7.13 (d, $J_{6,8}$ = 2.4 Hz, 1H, 8-H), 7.25–7.28 (m, 2H, 4-H, 5-H), 7.28–7.30 (m, 1H, Im), 7.34–7.37 (m, 1H, Im), 10.62 (s, 1H, NC*H*N) ppm; **^{13}C-NMR** (126 MHz, $CDCl_3$): δ = 14.1 (*C*H$_3$), 22.7 (CH_3*C*H$_2$), 25.8, 26.0, 26.1, 28.8, 28.97, 29.02, 29.2, 29.3, 29.4, 29.55, 29.57, 30.2 (*C*H$_2$), 31.9 (CH_3CH_2*C*H$_2$), 36.8 (N*C*H$_3$), 50.3 (N*C*H$_2$), 68.4, 68.6 (O*C*H$_2$), 110.2 (C-8), 110.3 (C-1), 120.5, 120.7, 120.8 (C-3, C-4, C-5, C-6), 121.6 (Im), 123.1 (Im), 135.90, 135.92 (C-1', C-8'), 137.3, 137.4 (C-4', C-5'), 138.2 (N*C*HN) 159.4 (C-2), 159.5 (C-7), 194.0 (*C*=O) ppm; **FT-IR** (ATR): $\tilde{\nu}$ = 3401 (w), 3068 (w), 2922 (s), 2853 (m), 1712 (s), 1607 (m), 1592 (m), 1572 (w), 1462 (vs), 1445 (m), 1392 (m), 1286 (s), 1248 (m), 1223 (s), 1169 (m), 1137 (w), 1039 (m), 1008 (w), 971 (w), 892 (w), 817 (m), 799 (m), 788 (m), 726 (w), 647 (w), 621 (w), 517 (w) cm^{-1}; **MS** (ESI): *m/z* = 545 $[M]^+$, 481, 431, 363; **HRMS** (ESI): calcd. for $[C_{35}H_{49}N_2O_3]^+$ 545.3738, found: 545.3742 $[M]^+$; **elemental analysis**: calcd. (%) for $C_{35}H_{49}BrN_2O_3$: C 67.19, H 7.89, N 4.48, found: C 64.43, H 7.78 N 4.44; **DSC**: Cr 51 [24.5 kJ mol^{-1}] SmC 82 [via POM] SmA 233 [1.3 kJ mol^{-1}] I (1. H).

3-(8-{[7-(Dodecyloxy)-9-oxo-9*H*-fluoren-2-yl]oxy}octyl)-1-methyl-1*H*-imidazol-3-ium bromide [Im(O8,O12)Br]. According to GP7, from **Br(O8,O12)** (200 mg, 350 µmol), **16a** (43 µL, 44 mg, 530 µmol), abs. DMF (7 mL); yield: 179 mg, 274 µmol, 78%. **^{1}H-NMR** (400 MHz, $CDCl_3$): δ = 0.88 (t, *J* = 6.6 Hz, 3H, C*H*$_3$), 1.18–1.52 (m, 26H, C*H*$_2$), 1.71–1.83 (m, 4H, OCH_2C*H*$_2$), 1.89–2.01 (m, 2H, NCH_2C*H*$_2$), 3.92–4.01 (m, 4H, OC*H*$_2$), 4.13 (s, 3H, NC*H*$_3$), 4.33 (t, *J* = 7.5 Hz, 2H, NC*H*$_2$), 6.88–6.96 (m, 2H, 3-H, 6-H), 7.10 (d, $J_{1,3}$ = 2.4 Hz, 1H, 1-H), 7.12 (d, $J_{6,8}$ = 2.3 Hz, 1H, 8-H), 7.23–7.29 (m, 2H, 4-H, 5-H), 7.32–7.37 (m, 1H, Im), 7.41–7.45 (m, 1H, Im), 10.52 (s, 1H, NC*H*N) ppm; **^{13}C-NMR** (126 MHz, $CDCl_3$): δ = 14.1 (*C*H$_3$), 22.7 (CH_3*C*H$_2$), 25.8, 26.0, 26.1, 28.8, 28.99, 29.00, 29.2, 29.3, 29.4, 29.57, 29.59, 29.63, 29.7, 30.3 (*C*H$_2$), 31.9 (CH_3CH_2*C*H$_2$), 36.8 (N*C*H$_3$), 50.2 (N*C*H$_2$), 68.4, 68.6 (O*C*H$_2$), 110.2 (C-8), 110.3 (C-1), 120.5, 120.7, 120.8 (C-3, C-4, C-5, C-6), 121.7 (Im), 123.3 (Im), 135.90, 135.91 (C-1', C-8'), 137.3, 137.4 (C-4', C-5'), 137.9 (N*C*HN), 159.4 (C-2), 159.5 (C-7), 194.0 (*C*=O) ppm; **FT-IR** (ATR): $\tilde{\nu}$ = 3408 (w), 3065 (w), 2918 (s), 2851 (m), 1713 (s), 1609 (w), 1591 (w), 1574 (w), 1462 (vs), 1391 (w), 1286 (vs), 1249 (m), 1221 (s), 1169 (m), 1140 (w), 1038 (m), 998 (w), 970 (w), 890 (w), 810 (m), 799 (m), 785 (m), 723 (w), 646 (w), 620 (w), 607 (w), 516 (w) cm^{-1}; **MS** (ESI): *m/z* = 573 $[M]^+$; **HRMS** (ESI): calcd. for $[C_{37}H_{53}N_2O_3]^+$ 573.4051, found: 573.4068 $[M]^+$; **elemental analysis**: calcd. (%) for $C_{37}H_{53}BrN_2O_3$: C 67.98, H 8.17, N 4.29, found: C 67.24, H 8.05, N 4.06; **DSC**: Cr 62 [29.5 kJ mol^{-1}] SmC 83 [via POM] SmA 244 [7.9 kJ mol^{-1}] I (1. H).

3-(8-{[7-(hexadecyloxy)-9-oxo-9*H*-fluoren-2-yl]oxy}octyl)-1-methyl-1*H*-imidazol-3-ium bromide [Im(O8,O16)Br]. According to GP7, from **Br(O8,O16)** (41 mg, 65 µmol), **16a** (8 µL, 8 mg, 98 µmol), abs. DMF (5 mL); yield: 45 mg, 63 µmol, 97%. **^{1}H-NMR** (500 MHz, $CDCl_3$): δ = 0.88 (t, *J* = 6.9 Hz, 3H, C*H*$_3$), 1.19–1.50 (m, 34H, C*H*$_2$), 1.71–1.83 (m, 4H, OCH_2C*H*$_2$), 1.88–1.98 (m, 2H, NCH_2C*H*$_2$), 3.92–4.01 (m, 4H, OC*H*$_2$), 4.12 (s, 3H, NC*H*$_3$), 4.33 (t, *J* = 7.5 Hz, 2H,

NCH_2), 6.90–6.94 (m, 2H, 3-H, 6-H), 7.11 (d, $J_{1,3}$ = 2.5 Hz, 1H, 1-H), 7.13 (d, $J_{6,8}$ = 2.5 Hz, 1H, 8-H), 7.24–7.29 (m, 3H, 4-H, 5-H, Im), 7.29–7.32 (m, 1H, Im), 10.68 (s, 1H, NC*H*N) ppm; **^{13}C-NMR** (126 MHz, $CDCl_3$): δ = 14.1 (CH_3), 22.7 (CH_3CH_2), 25.8, 26.0, 26.1, 28.8, 28.96, 28.99, 29.2, 29.4, 29.57, 29.60, 29.66, 29.68, 29.70, 30.2 (CH_2), 31.9 ($CH_3CH_2CH_2$), 36.8 (NCH_3), 50.3 (NCH_2), 68.4, 68.6 (OCH_2), 110.2 (C-8), 110.3 (C-1), 120.5, 120.7, 120.9 (C-3, C-4, C-5, C-6), 121.5 (Im), 123.0 (Im), 135.91, 135.92 (C-1', C-8'), 137.3, 137.4 (C-4', C-5'), 138.2 (N*C*HN), 159.4 (C-2), 159.5 (C-7), 194.0 (*C*=O) ppm; **FT-IR** (ATR): $\tilde{\nu}$ = 3417 (w), 3074 (w), 2917 (vs), 2851 (s), 1715 (s), 1614 (w), 1591 (w), 1465 (s), 1389 (w), 1291 (s), 1250 (m), 1223 (m), 1172 (m), 1042 (w), 971 (w), 890 (w), 810 (m), 800 (m), 784 (m), 719 (w), 621 (w), 515 (w) cm^{-1}; **MS** (ESI): *m/z* = 629 $[M]^+$; **HRMS** (ESI): calcd. for $[C_{41}H_{61}N_2O_3]^+$ 629.4677, found: 629.4682 $[M]^+$; **elemental analysis**: calcd. (%) for $C_{41}H_{61}BrN_2O_3$: C 69.37, H 8.66, N 3.95, found: C 66.23, H 8.34, N 3.63; **DSC**: Cr 61 [53.5 kJ mol^{-1}] SmC 90 [via POM] SmA 266 [6.5 kJ mol^{-1}] I (1. H).

3-(10-{[7-(Decyloxy)-9-oxo-9*H*-fluoren-2-yl]oxy}decyl)-1-methyl-1*H*-imidazol-3-ium bromide [Im(O10,O10)Br]. According to GP7, from **Br(O10,O10)** (170 mg, 297 µmol), **16a** (35 µL, 36 mg, 445 µmol), abs. DMF (8 mL); yield: 166 mg, 254 µmol, 86%. **^{1}H-NMR** (400 MHz, $CDCl_3$): δ = 0.88 (t, *J* = 6.6 Hz, 3H, CH_3), 1.20–1.39 (m, 22H, CH_2), 1.39–1.50 (m, 4H, $O(CH_2)_2CH_2$), 1.72–1.82 (m, 4H, OCH_2CH_2), 1.86–1.99 (m, 2H, NCH_2CH_2), 3.96 (t, *J* = 6.5 Hz, 4H, OCH_2), 4.14 (s, 3H, NCH_3), 4.32 (t, *J* = 7.4 Hz, 2H, NCH_2), 6.91 (dd, *J* = 8.1 Hz, 2.4 Hz, 2H, 3-H, 6-H), 7.08–7.15 (m, 2H, 1-H, 8-H), 7.23–7.30 (m, 2H, 4-H, 5-H), 7.36–7.42 (m, 1H, Im), 7.50–7.54 (m, 1H, Im), 10.51 (s, 1H, NC*H*N) ppm; **^{13}C-NMR** (101 MHz, $CDCl_3$): δ = 14.1 (CH_3), 22.7 (CH_3CH_2), 25.9, 26.0, 26.2, 28.9, 29.1, 29.17, 29.20, 29.3, 29.4, 29.5, 29.6, 30.3 (CH_2), 31.9 ($CH_3CH_2CH_2$), 36.8 (NCH_3), 50.2 (NCH_2), 68.5, 68.6 (OCH_2), 110.26 (C-8), 110.32 (C-1), 120.5, 120.7, 120.8 (C-3, C-4, C-5, C-6), 121.6 (Im), 123.3 (Im), 135.9 (C-1', C-8'), 137.3, 137.4 (C-4', C-5'), 137.9 (N*C*HN), 159.46 (C-2), 159.51 (C-7), 193.9 (*C*=O) ppm; **FT-IR** (ATR): $\tilde{\nu}$ = 3419 (w), 2922 (vs), 2853 (s), 1713 (m), 1608 (w), 1592 (w), 1573 (w), 1463 (s), 1391 (w), 1287 (s), 1248 (m), 1223 (m), 1169 (m), 1040 (w), 1006 (w), 971 (w), 921 (w), 891 (w), 811 (w), 799 (m), 730 (s), 645 (w), 622 (w), 516 (w) cm^{-1}; **MS** (ESI): *m/z* = 573 $[M]^+$; **HRMS** (ESI): calcd. for $[C_{37}H_{53}N_2O_3]^+$ 573.4051, found: 573.4059 $[M]^+$; **elemental analysis**: calcd. (%) for $C_{37}H_{53}BrN_2O_3$: C 67.98, H 8.17, N 4.29, found: C 67.24, H 8.34, N 4.05; **DSC**: Cr 73 [33.6 kJ mol^{-1}] SmC 124 [0.4 kJ mol^{-1}] SmA 201 [2.3 kJ mol^{-1}] I (1. H); I 199 [–2.1 kJ mol^{-1}] SmA 124 [–0.3 kJ mol^{-1}] SmC 52 [–31.8 kJ mol^{-1}] Cr (1. K).

3-(10-{[7-(Dodecyloxy)-9-oxo-9*H*-fluoren-2-yl]oxy}decyl)-1-methyl-1*H*-imidazol-3-ium bromide [Im(O10,O12)Br]. According to GP7, from **Br(O10,O12)** (110 mg, 183 µmol), **16a** (21 µL, 22 mg, 275 µmol), abs. DMF (6 mL); yield: 118 mg, 173 µmol, 95%. **^{1}H-NMR** (400 MHz, $CDCl_3$): δ = 0.88 (t, *J* = 6.6 Hz, 3H, CH_3), 1.19–1.39 (m, 26H, CH_2), 1.39–1.49 (m, 4H, $OCH_2CH_2CH_2$), 1.72–1.83 (m, 4H, OCH_2CH_2), 1.92 (m_c, 2H, NCH_2CH_2), 3.97 (t, *J* = 6.5 Hz, 4H, OCH_2), 4.13 (s, 3H, NCH_3), 4.32 (t, *J* = 7.4 Hz, 2H, NCH_2), 6.92 (dd, *J* = 8.1 Hz, 2.4 Hz, 2H, 3-H, 6-H), 7.12 (d, $J_{1,3}$ = 2.4 Hz, 1H, 1-H), 7.13 (d, $J_{6,8}$ = 2.4 Hz, 1H, 8-H) 7.24–7.28 (m, 2H, 4-H, 5-H), 7.29–7.31 (m, 1H, Im), 7.37–7.40 (m, 1H, Im), 10.56 (s, 1H, NC*H*N) ppm; **^{13}C-NMR** (101

MHz, $CDCl_3$): δ = 14.1 (CH_3), 22.7 (CH_3CH_2), 25.9, 26.0, 26.2, 28.9, 29.1, 29.17, 29.20, 29.30, 29.34, 29.4, 29.56, 29.58, 29.62, 29.7, 30.3 (CH_2), 31.9 ($CH_3CH_2CH_2$), 36.8 (NCH_3), 50.2 (NCH_2), 68.5, 68.6 (OCH_2), 110.2 (C-8), 110.3 (C-1), 120.5, 120.7, 120.8 (C-3, C-4, C-5, C-6), 121.6 (Im), 123.3 (Im), 135.9 (C-1', C-8'), 137.3, 137.4 (C-4', C-5'), 138.0 (NCHN), 159.46 (C-2), 159.51 (C-7), 194.0 (*C*=O) ppm; **FT-IR** (ATR): $\tilde{\nu}$ = 3405 (w), 2919 (s), 2851 (m), 1714 (m), 1609 (w), 1591 (w), 1573 (w), 1461 (vs), 1446 (m), 1390 (w), 1286 (s), 1248 (m), 1221 (s), 1169 (m), 1039 (w), 1000 (w), 970 (w), 909 (m), 810 (m), 799 (m), 785 (m), 731 (vs), 646 (w), 621 (w), 515 (m) cm^{-1}; **MS** (ESI): *m/z* = 601 $[M]^+$; **HRMS** (ESI): calcd. for $[C_{39}H_{57}N_2O_3]^+$ 601.4364, found: 601.4339 $[M]^+$; **elemental analysis**: calcd. (%) for $C_{39}H_{57}BrN_2O_3$: C 68.70, H 8.43, N 4.11, found: C 67.07, H 8.44, N 4.30; **DSC**: Cr 68 [22.4 kJ mol^{-1}] SmC 121 [via POM] SmA 220 [8.5 kJ mol^{-1}] I (1. H); I 214 [–1.3 kJ mol^{-1}] SmA 121 [via POM] SmC 50 [via POM] Cr (1. K).

3-(12-{[7-(Decyloxy)-9-oxo-9*H*-fluoren-2-yl]oxy}dodecyl)-1-methyl-1*H*-imidazol-3-ium bromide [Im(O12,O10)Br]. According to GP7, from **Br(O12,O10)** (170 mg, 280 µmol), **16a** (33 µL, 34 mg, 420 µmol), abs. DMF (8 mL); yield: 136 mg, 200 µmol, 71%. **^{1}H-NMR** (400 MHz, $CDCl_3$): δ = 0.88 (t, *J* = 6.6 Hz, 3H, CH_3), 1.20–1.39 (m, 26H, CH_2), 1.39–1.51 (m, 4H, $O(CH_2)_2CH_2$), 1.73–1.82 (m, 4H, OCH_2CH_2), 1.87–1.99 (m, 2H, NCH_2CH_2), 3.97 (t, *J* = 6.5 Hz, 4H, OCH_2), 4.14 (s, 3H, NCH_3), 4.32 (t, *J* = 7.5 Hz, 2H, NCH_2), 6.91 (dd, *J* = 8.2 Hz, 2.4 Hz, 2H, 3-H, 6-H), 7.10–7.14 (m, 2H, 1-H, 8-H), 7.24–7.30 (m, 2H, 4-H, 5-H), 7.34–7.38 (m, 1H, Im), 7.48–7.50 (m, 1H, Im), 10.54 (s, 1H, NC*H*N) ppm; **^{13}C-NMR** (101 MHz, $CDCl_3$): δ = 14.1 (CH_3), 22.7 (CH_3CH_2), 25.9, 26.0, 26.3, 28.9, 29.1, 29.2, 29.27, 29.31, 29.36, 29.40, 29.42, 29.44, 29.5, 29.6, 30.3 (CH_2), 31.9 ($CH_3CH_2CH_2$), 36.8 (NCH_3), 50.2 (NCH_2), 68.56, 68.59 (OCH_2), 110.25 (C-8), 110.29 (C-1), 120.5, 120.7, 120.8 (C-3, C-4, C-5, C-6), 121.6 (Im), 123.2 (Im), 135.9 (C-1', C-8'), 137.4 (C-4', C-5'), 138.0 (NCHN), 159.48 (C-2), 159.50 (C-7), 193.9 (*C*=O) ppm; **FT-IR** (ATR): $\tilde{\nu}$ = 3403 (w), 2920 (s), 2851 (m), 1713 (s), 1607 (w), 1593 (w), 1574 (w), 1463 (vs), 1392 (w), 1286 (s), 1266 (m), 1250 (m), 1223 (s), 1171 (m), 1138 (w), 1037 (m), 1005 (w), 970 (w), 909 (m), 813 (w), 799 (m), 786 (m), 731 (vs), 645 (w), 623 (w), 606 (w), 516 (w) cm^{-1}; **MS** (ESI): *m/z* = 601 $[M]^+$; **HRMS** (ESI): calcd. for $[C_{39}H_{57}N_2O_3]^+$ 601.4364, found: 601.4356 $[M]^+$; **elemental analysis**: calcd. (%) for $C_{39}H_{57}BrN_2O_3$: C 68.70, H 8.43, N 4.11, found: C 68.31, H 8.59, N 4.12; **DSC**: Cr 83 [39.2 kJ mol^{-1}] SmC 136 [0.5 kJ mol^{-1}] SmA 176 [2.8 kJ mol^{-1}] I (3. H); I 176 [–2.9 kJ mol^{-1}] SmA 136 [–0.5 kJ mol^{-1}] SmC 73 [–40.2 kJ mol^{-1}] Cr (3. K).

3-(12-{[7-(Dodecyloxy)-9-oxo-9*H*-fluoren-2-yl]oxy}dodecyl)-1-methyl-1*H*-imidazol-3-ium bromide [Im(O12,O12)Br]. According to GP7, from **Br(O12,O12)** (110 mg, 175 µmol), **16a** (21 µL, 22 mg, 263 µmol), abs. DMF (8 mL); yield: 117 mg, 165 µmol, 94%. **^{1}H-NMR** (400 MHz, $CDCl_3$): δ = 0.88 (t, *J* = 6.6 Hz, 3H, CH_3), 1.19–1.39 (m, 30H, CH_2), 1.39–1.52 (m, 4H, $O(CH_2)_2CH_2$), 1.77 (m_c, 4H, OCH_2CH_2), 1.92 (m_c, 2H, NCH_2CH_2), 3.97 (t, *J* = 6.6 Hz, 4H, OCH_2), 4.13 (s, 3H, NCH_3), 4.31 (t, *J* = 7.4 Hz, 2H, NCH_2), 6.92 (dd, *J* = 8.2 Hz, 2.4 Hz, 2H, 3-H, 6-H), 7.11–7.15 (m, 2H, 1-H, 8-H), 7.24–7.29 (m, 2H, 4-H, 5-H), 7.29–7.33 (m, 1H, Im), 7.39–7.44 (m, 1H, Im), 10.61 (s, 1H, NC*H*N) ppm; **^{13}C-NMR** (101 MHz, $CDCl_3$): δ = 14.1 (CH_3), 22.7 (CH_3CH_2), 25.9, 26.0, 26.3, 28.9, 29.1, 29.2, 29.27, 29.31, 29.34, 29.36, 29.40, 29.42, 29.44,

29.56, 29.58, 29.62, 29.7, 30.3 (CH_2), 31.9 ($CH_3CH_2CH_2$), 36.8 (NCH_3), 50.3 (NCH_2), 68.56, 68.59 (OCH_2), 110.2 (C-8), 110.3 (C-1), 120.5, 120.7, 120.8 (C-3, C-4, C-5, C-6), 121.6 (Im), 123.2 (Im), 135.9 (C-1', C-8'), 137.4 (C-4', C-5'), 138.0 (N*C*HN), 159.48 (C-2), 159.51 (C-7), 193.9 (*C*=O) ppm; **FT-IR** (ATR): $\tilde{\nu}$ = 3415 (w), 2917 (vs), 2850 (s), 1715 (s), 1615 (w), 1590 (w), 1575 (w), 1465 (vs), 1403 (w), 1389 (w), 1289 (vs), 1251 (w), 1223 (m), 1171 (m), 1142 (w), 1038 (m), 1005 (w), 998 (w), 970 (w), 891 (w), 810 (m), 800 (m), 784 (m), 720 (w), 647 (w), 619 (w), 602 (w), 515 (w) cm^{-1}; **MS** (ESI): *m/z* = 629 $[M]^+$; **HRMS** (ESI): calcd. for $[C_{41}H_{61}N_2O_3]^+$ 629.4677, found: 629.4638 $[M]^+$; **elemental analysis**: calcd. (%) for $C_{41}H_{61}BrN_2O_3$: C 69.37, H 8.66, N 3.95, found: C 68.04, H 8.39, N 3.76; **DSC**: Cr 80 [37.9 kJ mol^{-1}] SmC 133 [0.6 kJ mol^{-1}] SmA 189 [1.9 kJ mol^{-1}] I (3. H); I 189 [–2.1 kJ mol^{-1}] SmA 133 [–0.5 kJ mol^{-1}] SmC 69 [–36.4 kJ mol^{-1}] Cr (3. K).

General Procedure for the Anion Exchange[12]

A solution of **Im(On,Om)Br** (80 µmol) in MeCN (20 mL) and NaOTf (17 mg, 96 µmol) was heated at reflux for 10 min. The solvent was removed under reduced pressure, and the residue was taken up in CH_2Cl_2 (4 mL) and filtered through a syringe filter (0.2 µm, PVDF). The filtrate was concentrated and dried under high vacuum to give **Im(On,Om)OTf** quantitatively as orange solids.

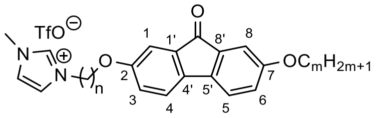

3-(4-{[7-(Tetradecyloxy)-9-oxo-9*H*-fluoren-2-yl]oxy}butyl)-1-methyl-1*H*-imidazol-3-ium trifluoromethansulfonate [Im(O4,O14)OTf]. **^{1}H-NMR** (500 MHz, $CDCl_3$): δ = 0.88 (t, *J* = 6.9 Hz, 3H, CH_3), 1.16–1.50 (m, 22H, CH_2), 1.77 (m_c, 2H, OCH_2CH_2), 1.80–1.88 (m, 2H, $N(CH_2)_2CH_2$), 2.11 (m_c, 2H, NCH_2CH_2), 3.91–4.05 (m, 7H, OCH_2, NCH_3), 4.33 (t, *J* = 7.4 Hz, 2H, NCH_2), 6.87–6.93 (m, 2H, 3-H, 6-H), 7.03 (d, $J_{1,3}$ = 2.4 Hz, 1H, 1-H), 7.10 (d, $J_{6,8}$ = 2.4 Hz, 1H, 8-H), 7.22–7.27 (m, 2H, 4-H, 5-H), 7.28–7.31 (m, 1H, Im), 7.34–7.37 (m, 1H, Im), 9.26 (s, 1H, NC*H*N) ppm; **^{13}C-NMR** (126 MHz, $CDCl_3$): δ = 14.1 (CH_3), 22.7 (CH_3CH_2), 25.7, 26.0, 27.1, 29.2, 29.37, 29.39, 29.58, 29.61, 29.66, 29.68, 29.70 (CH_2), 31.9 ($CH_3CH_2CH_2$), 36.5 (NCH_3), 49.9 (NCH_2), 67.4, 68.6 (OCH_2), 110.2 (C-8), 110.4 (C-1), 120.3, 120.6, 120.8 (C-3, C-4, C-5, C-6), 122.0 (Im), 123.4 (Im), 135.8, 135.9 (C-1', C-8'), 137.1, 137.3, 137.7 (C-4', C-5', N*C*HN), 158.8 (C-2), 159.6 (C-7), 193.7 (*C*=O) ppm; **^{19}F-NMR** (376 MHz, $CDCl_3$): δ = -78.5 (s, CF_3) ppm; **FT-IR** (ATR): $\tilde{\nu}$ = 3155 (w), 3116 (w), 2918 (m), 2851 (m), 1715 (m), 1608 (w), 1577 (w), 1463 (s), 1394 (w), 1261 (vs), 1224 (s), 1162 (m), 1031 (s), 970 (w), 892 (w), 813 (w), 786 (m), 756 (w), 638 (m), 573 (w), 517 (m) cm^{-1}; **MS** (ESI, positive): *m/z* = 545 $[M]^+$, 463, 431, 363; **MS** (ESI, negative): *m/z* = 149 $[CF_3O_3S]^-$; **HRMS** (ESI): calcd. for $[C_{35}H_{49}N_2O_3]^+$ 545.3738, found: 545.3717 $[M]^+$; **elemental analysis**: calcd. (%) for $C_{36}H_{49}F_3N_2O_6S$: C 62.23, H 7.11, N 4.03, found: C 61.77, H 6.84, N 3.78; **DSC**: Cr 65 [41.8 kJ mol^{-1}] SmA 243 [1.0 kJ mol^{-1}] I (1. H).

3-(6-{[7-(Octyloxy)-9-oxo-9*H*-fluoren-2-yl]oxy}hexyl)-1-methyl-1*H*-imidazol-3-ium trifluoromethansulfonate [Im(O6,O8)OTf]. **^{1}H-NMR** (400 MHz, $CDCl_3$): δ = 0.85–0.92 (m, 3H, CH_3),

1.22–1.57 (m, 14H, CH_2), 1.71–1.82 (m, 4H, OCH_2CH_2), 1.92 (m_c, 2H, NCH_2CH_2), 3.89–4.04 (m, 7H. OCH_2, NCH_3), 4.22 (t, J = 7.4 Hz, 2H, NCH_2), 6.87–6.94 (m, 2H, 3-H, 6-H), 7.05 (d, $J_{1,3}$ = 2.4 Hz, 1H, 1-H), 7.11 (d, $J_{6,8}$ = 2.4 Hz, 1H, 8-H), 7.22–7.28 (m, 2H, 4-H, 5-H), 7.30–7.35 (m, 2H, Im), 9.19 (s, 1H, NC*H*N) ppm; **^{13}C-NMR** (101 MHz, $CDCl_3$): δ = 14.1 (CH_3), 22.7 (CH_3CH_2), 25.4, 25.8, 26.0, 28.8, 29.2, 29.2, 29.3, 30.0 (CH_2), 31.8 ($CH_3CH_2CH_2$), 36.5 (NCH_3), 50.1 (NCH_2), 68.1, 68.6 (OCH_2), 110.3 (C-8), 110.4 (C-1), 120.6, 120.8 (C-3, C-4, C-5, C-6), 122.0 (Im), 123.5 (Im), 135.89, 135.91 (C-1', C-8'), 137.1, 137.3, 137.4 (C-4', C-5', N*C*HN), 159.3 (C-2), 159.6 (C-7), 193.9 (*C*=O) ppm; **^{19}F-NMR** (376 MHz, $CDCl_3$): δ = –78.5 (s, CF_3) ppm; **FT-IR** (ATR): $\tilde{\nu}$ = 3115 (w), 2927 (m), 2857 (w), 1711 (m), 1608 (w), 1593 (w), 1573 (w), 1461 (s), 1445 (m), 1392 (w), 1256 (vs), 1223 (s), 1159 (s), 1074 (w), 1029 (s), 970 (w), 908 (m), 818 (w), 787 (m), 727 (vs), 636 (vs), 573 (m), 516 (m) cm^{-1}; **MS** (ESI, positive): *m/z* = 489 $[M]^+$,376, 325, 123; **MS** (ESI, negative): *m/z* = 149 $[CF_3O_3S]^-$; **HRMS** (ESI): calcd. for $[C_{31}H_{41}N_2O_3]^+$ 489.3112, found: 489.3127 $[M]^+$; **elemental analysis**: calcd. (%) for $C_{32}H_{41}F_3N_2O_6S$: C 60.17, H 6.47, N 4.39, found: C 59.83, H 6.41, N 4.32; **DSC**: Cr_1 35 [–2.8 kJ mol^{-1}] Cr_2 73 [38.8 kJ mol^{-1}] Cr_3 81 [8.0 kJ mol^{-1}] SmA 114 [1.4 kJ mol^{-1}] I (3. H); I 113 [–1.5 kJ mol^{-1}] SmA 22 [–32.8 kJ mol^{-1}] Cr (3. C).

3-(6-{[7-(Tetradecyloxy)-9-oxo-9*H*-fluoren-2-yl]oxy}hexyl)-1-methyl-1*H*-imidazol-3-ium trifluoromethansulfonate [Im(O6,O14)OTf]. **^{1}H-NMR** (400 MHz, $CDCl_3$): δ = 0.88 (t, J = 6.7 Hz, 3H, CH_3), 1.20–1.38 (m, 22H, CH_2), 1.39–1.59 (m, 4H, $OCH_2CH_2CH_2$), 1.72–1.85 (m, 4H, OCH_2CH_2), 1.94 (m_c, 2H, NCH_2CH_2), 3.93–4.03 (m, 7H, OCH_2, NCH_3), 4.24 (t, J = 7.4 Hz, 2H, NCH_2), 6.89–6.95 (m, 2H, 3-H, 6-H), 7.07 (d, $J_{1,3}$ = 2.4 Hz, 1H, 1-H), 7.13 (d, $J_{6,8}$ = 2.4 Hz, 1H, 8-H), 7.23–7.30 (m, 4H, 4-H, 5-H, Im), 9.33 (s, 1H, NC*H*N) ppm; **^{13}C-NMR** (101 MHz, $CDCl_3$): δ = 14.1 (CH_3), 22.7 (CH_3CH_2), 25.4, 25.8, 26.0, 28.7, 29.2, 29.4, 29.58, 29.60, 29.67, 29.70, 30.0 (CH_2), 31.9 ($CH_3CH_2CH_2$), 36.6 (NCH_3), 50.2 (NCH_2), 68.1, 68.6 (OCH_2), 110.3 (C-8), 110.4 (C-1), 120.6, 120.9 (C-3, C-4, C-5 C-6), 121.8 (Im), 123.3 (Im), 135.9, 136.0 (C-1', C-8'), 137.3, 137.5, 137.6 (C-4', C-5', N*C*HN), 159.3 (C-2), 159.6 (C-7), 193.9 (*C*=O) ppm; **^{19}F-NMR** (376 MHz, $CDCl_3$): δ = –78.5 (s, CF_3) ppm; **FT-IR** (ATR): $\tilde{\nu}$ = 3156 (w), 3118 (w), 2920 (m), 2852 (m), 1713 (m), 1608 (w), 1592 (w), 1576 (w), 1463 (s), 1393 (w), 1258 (vs), 1224 (s), 1161 (s), 1031 (s), 972 (w), 892 (w), 817 (m), 788 (m), 756 (w), 729 (w), 639 (s), 574 (w), 518 (m) cm^{-1}; **MS** (ESI, positive): *m/z* = 573 $[M]^+$; **MS** (ESI, negative): *m/z* = 149 $[CF_3O_3S]^-$; **HRMS** (ESI): calcd. for $[C_{37}H_{53}N_2O_3]^+$ 573.4051, found: 573.4076 $[M]^+$; **elemental analysis**: calcd. (%) for $C_{38}H_{53}F_3N_2O_6S$: C 63.14, H 7.39, N 3.88, found: C 58.65, H 6.66, N 3.47; **DSC**: Cr 71 [40.8 kJ mol^{-1}] SmA 220 [1.0 kJ mol^{-1}] I (1. H).

3-(8-{[7-(Decyloxy)-9-oxo-9*H*-fluoren-2-yl]oxy}octyl)-1-methyl-1*H*-imidazol-3-ium trifluoromethansulfonate [Im(O8,O10)OTf]. **^{1}H-NMR** (500 MHz, $CDCl_3$): δ = 0.88 (t, J = 6.9 Hz, 3H, CH_3), 1.20–1.40 (m, 18H, CH_2), 1.40–1.50 (m, 4H, $OCH_2CH_2CH_2$), 1.71–1.82 (m, 4H, OCH_2CH_2), 1.89 (m_c, 2H, NCH_2CH_2), 3.92–4.03 (m, 7H, OCH_2, NCH_3), 4.21 (t, J = 7.5 Hz, 2H, NCH_2), 6.89–6.94 (m, 2H, 3-H, 6-H), 7.10 (d, $J_{1,3}$ = 2.4 Hz, 1H, 1-H), 7.12 (d, $J_{6,8}$ = 2.5 Hz, 1H, 8-H), 7.24–7.28 (m, 3H, 4-H, 5-H, Im), 7.30–7.32 (m, 1H, Im), 9.30 (s, 1H, NC*H*N) ppm; **^{13}C-**

NMR (126 MHz, $CDCl_3$): δ = 14.1 (*C*H_3), 22.7 (CH_3*C*H_2), 25.8, 26.0, 26.1, 28.8, 28.9, 29.0, 29.2, 29.3, 29.4, 29.55, 29.57, 30.0 (*C*H_2), 31.9 (CH_3CH_2*C*H_2), 36.5 (N*C*H_3), 50.2 (N*C*H_2), 68.4, 68.6 (O*C*H_2), 110.2 (C-8), 110.3 (C-1), 120.5, 120.7, 120.8 (C-3, C-4, C-5, C-6), 121.8 (Im), 123.4 (Im), 135.90, 135.91 (C-1', C-8'), 137.29, 137.34, 137.4 (C-4', C-5', N*C*HN), 159.4 (C-2), 159.5 (C-7), 194.0 (*C*=O) ppm; **^{19}F-NMR** (376 MHz, $CDCl_3$): δ = –78.5 (s, C*F*$_3$) ppm; **FT-IR** (ATR): $\tilde{\nu}$ = 3115 (w), 2922 (m), 2854 (m), 1714 (m), 1608 (w), 1591 (w), 1575 (w), 1481 (m), 1464 (m), 1391 (w), 1251 (vs), 1224 (s), 1160 (s), 1030 (s), 969 (w), 915 (w), 836 (w), 813 (m), 798 (m), 790 (m), 757 (w), 730 (m), 638 (s), 623 (m), 608 (w), 574 (w), 518 (m) cm^{-1}; **MS** (ESI, positive): *m/z* = 545 $[M]^+$, 404, 353, 193, 165, 151, 137, 109; **MS** (ESI, negative): *m/z* = 149 $[CF_3O_3S]^-$; **HRMS** (ESI): calcd. for $[C_{35}H_{49}N_2O_3]^+$ 545.3738, found: 545.3734 $[M]^+$; **elemental analysis**: calcd. (%) for $C_{36}H_{49}F_3N_2O_6S$: C 62.23, H 7.11, N 4.03, found: C 61.44, H 7.02, N 4.22; **DSC**: Cr_1 13 [–3.1 kJ mol^{-1}] Cr_2 47 [–1.0 kJ mol^{-1}] Cr_3 74 [21.1 kJ mol^{-1}] SmA 130 [1.3 kJ mol^{-1}] I (3. H); I 130 [–1.6 kJ mol^{-1}] SmA 47 [–1.4 kJ mol^{-1}] Cr (3. K).

3-(4-{[7-(Dodecylthio)-9-oxo-9*H*-fluoren-2-yl]oxy}butyl)-1-methyl-1*H*-imidazol-3-ium bromide [Im(O4,S12)Br]. According to GP7, from **Br(O4,S12)** (130 mg, 245 µmol), **16a** (29 µL, 30 mg, 368 µmol), abs. DMF (5 mL); yield: 124 mg, 202 µmol, 82%. **^{1}H-NMR** (400 MHz, $CDCl_3$): δ = 0.87 (t, *J* = 6.7 Hz, 3H, C*H*$_3$), 1.18–1.36 (m, 16H, C*H*$_2$), 1.36–1.48 (m, 2H, SCH_2CH_2C*H*$_2$), 1.64 (m_c, 2H, SCH_2C*H*$_2$), 1.84–1.95 (m, 2H, OCH_2C*H*$_2$), 2.18 (m_c, 2H, NCH_2C*H*$_2$), 2.92 (t, *J* = 7.4 Hz, 2H, SC*H*$_2$), 4.05 (t, *J* = 5.9 Hz, 2H, OC*H*$_2$), 4.11 (s, 3H, NC*H*$_3$), 4.50 (t, *J* = 7.4 Hz, 2H, NC*H*$_2$), 6.95 (dd, $J_{3,4}$ = 8.2 Hz, $J_{1,3}$ = 2.4 Hz, 1H, 3-H), 7.06 (d, $J_{1,3}$ = 2.4 Hz, 1H, 1-H), 7.24–7.29 (m, 1H, 5-H), 7.29–7.36 (m, 2H, 4-H, 6-H), 7.41–7.44 (m, 1H, Im), 7.47–7.51 (m, 2H, 8-H, Im), 10.52 (s, 1H, NC*H*N) ppm; **^{13}C-NMR** (101 MHz, $CDCl_3$): δ = 14.1 (*C*H_3), 22.7 (CH_3*C*H_2), 25.9, 27.3, 28.8, 29.0, 29.2, 29.3, 29.5, 29.58, 29.62, 29.64 (*C*H_2), 31.9 (CH_3CH_2*C*H_2), 33.7 (S*C*H_2), 36.9 (N*C*H_3), 49.8 (N*C*H_2), 67.5 (O*C*H_2), 110.4 (C-1), 120.0 (C-5), 120.5 (C-3), 121.4 (C-4), 122.0 (Im), 123.3 (Im), 124.2 (C-8), 134.7 (C-6), 134.9 (C-8'), 135.6 (C-1'), 137.0 (C-4'), 137.7 (C-7), 137.9 (N*C*HN), 142.1 (C-5'), 159.6 (C-2), 193.4 (*C*=O) ppm; **FT-IR** (ATR): $\tilde{\nu}$ = 3391 (m), 3151 (w), 3102 (w), 2954 (w), 2919 (vs), 2851 (s), 1712 (s), 1601 (m), 1574 (w), 1488 (m), 1456 (s), 1437 (m), 1420 (w), 1344 (w), 1289 (m), 1248 (s), 1226 (m), 1204 (w), 1169 (m), 1142 (w), 1101 (w), 1064 (w), 1034 (w), 1004 (w), 965 (w), 908 (w), 861 (w), 817 (m), 781 (m), 759 (m), 735 (m), 644 (w), 620 (w), 498 (m) cm^{-1}; **MS** (ESI): *m/z* = 533 $[M]^+$; **HRMS** (ESI): calcd. for $[C_{33}H_{45}N_2O_2S]^+$ 533.3196, found: 533.3203 $[M]^+$; **elemental analysis**: calcd. (%) for $C_{33}H_{45}BrN_2O_2S$: C 64.59, H 7.39, N 4.56, found: C 62.07, H 7.25, N 4.52; **DSC**: Cr 74 [25.4 kJ mol^{-1}] SmA 272 [1.2 kJ mol^{-1}] I (1. H).

3-(4-{[7-(Tetradecylthio)-9-oxo-9*H*-fluoren-2-yl]oxy}butyl)-1-methyl-1*H*-imidazol-3-ium bromide [Im(O4,S14)Br]. According to GP7, from **Br(O4,S14)** (130 mg, 232 µmol), **16a** (28 µL, 29 mg, 348 µmol), abs. DMF (4 mL); yield: 116 mg, 181 µmol, 78%. **^{1}H-NMR** (400 MHz, $CDCl_3$): δ = 0.87 (t, *J* = 6.7 Hz, 3H, C*H*$_3$), 1.19–1.36 (m, 20H, C*H*$_2$), 1.37–1.48 (m, 2H, SCH_2CH_2C*H*$_2$), 1.65 (m_c, 2H, SCH_2C*H*$_2$), 1.85–1.96 (m, 2H, OCH_2C*H*$_2$), 2.19 (m_c, 2H, NCH_2C*H*$_2$), 2.93 (t, *J* = 7.4 Hz, 2H, SC*H*$_2$), 4.06 (t, *J* = 5.9 Hz, 2H, OC*H*$_2$), 4.11 (s, 3H, NC*H*$_3$),

4.50 (t, $J = 7.4$ Hz, 2H, NCH_2), 6.96 (dd, $J_{3,4} = 8.2$ Hz, $J_{1,3} = 2.4$ Hz, 1H, 3-H), 7.07 (d, $J_{1,3} = 2.4$ Hz, 1H, 1-H), 7.25–7.30 (m, 1H, 5-H), 7.31–7.39 (m, 3H, 4-H, 6-H, Im), 7.43–7.46 (m, 1H, Im), 7.48–7.51 (m, 1H, 8-H), 10.69 (s, 1H, NC*H*N) ppm; **^{13}C-NMR** (101 MHz, $CDCl_3$): δ = 14.1 (CH_3), 22.7 (CH_3CH_2), 25.8, 27.3, 28.8, 29.0, 29.2, 29.4, 29.5, 29.58, 29.65, 29.67, 29.69 (CH_2), 31.9 ($CH_3CH_2CH_2$), 33.7 (SCH_2), 36.8 (NCH_3), 49.8 (NCH_2), 67.5 (OCH_2), 110.4 (C-1), 120.0 (C-5), 120.5 (C-3), 121.4 (C-4), 121.9 (Im), 123.1 (Im), 124.2 (C-8), 134.7 (C-6), 134.9 (C-8'), 135.7 (C-1'), 137.0 (C-4'), 137.7 (C-7), 138.2 (N*C*HN), 142.1 (C-5'), 159.6 (C-2), 193.4 (*C*=O) ppm; **FT-IR** (ATR): $\tilde{\nu}$ = 3416 (w), 3148 (w), 3082 (w), 2954 (w), 2917 (vs), 2850 (s), 1714 (s), 1602 (m), 1574 (w), 1488 (m), 1470 (m), 1457 (m), 1435 (w), 1420 (w), 1383 (w), 1341 (w), 1295 (m), 1248 (m), 1204 (w), 1171 (m), 1138 (w), 1090 (w), 1065 (w), 1036 (w), 963 (w), 889 (w), 861 (w), 819 (w), 781 (m), 765 (w), 741 (w), 644 (w), 620 (w), 497 (w) cm^{-1}; **MS** (ESI): *m/z* = 561 $[M]^+$; **HRMS** (ESI): calcd. for $[C_{35}H_{49}N_2O_2S]^+$ 561.3509, found: 561.3497 $[M]^+$; **elemental analysis**: calcd. (%) for $C_{35}H_{49}BrN_2O_2S$: C 65.51, H 7.70, N 4.37, S 5.00, found: C 64.83, H 7.76, N 4.24, S 4.93; **DSC**: Cr 86 [36.1 kJ mol^{-1}] SmA 273 [1.0 kJ mol^{-1}] I (1. H).

3-(6-{[7-(Dodecylthio)-9-oxo-9*H*-fluoren-2-yl]oxy}hexyl)-1-methyl-1*H*-imidazol-3-ium bromide [Im(O6,S12)Br]. According to GP7, from **Br(O6,S12)** (100 mg, 179 µmol), **16a** (20 µL, 22 mg, 269 µmol), abs. DMF (6 mL); yield: 85 mg, 132 µmol, 74%. **^{1}H-NMR** (400 MHz, $CDCl_3$): δ = 0.87 (t, $J = 6.7$ Hz, 3H, CH_3), 1.15–1.34 (m, 16H, CH_2), 1.34–1.59 (m, 6H, $SCH_2CH_2CH_2$, $OCH_2CH_2CH_2$, $NCH_2CH_2CH_2$), 1.65 (m_c, 2H, SCH_2CH_2), 1.79 (m_c, 2H, OCH_2CH_2), 1.98 (m_c, 2H, NCH_2CH_2), 2.93 (t, $J = 7.4$ Hz, 2H, SCH_2), 3.98 (t, $J = 6.4$ Hz, 2H, OCH_2), 4.12 (s, 3H, NCH_3), 4.37 (t, $J = 7.3$ Hz, 2H, NCH_2), 6.95 (dd, $J_{3,4} = 8.2$ Hz, $J_{1,3} = 2.4$ Hz, 1H, 3-H), 7.09 (d, $J_{1,3} = 2.4$ Hz, 1H, 1-H), 7.25–7.31 (m, 1H, 5-H), 7.31–7.40 (m, 4H, 4-H, 6-H, Im), 7.49–7.53 (m, 1H, 8-H), 10.55 (s, 1H, NC*H*N) ppm; **^{13}C-NMR** (101 MHz, $CDCl_3$): δ = 14.1 (CH_3), 22.7 (CH_3CH_2), 25.5, 25.9, 28.79, 28.80 29.0, 29.2, 29.3, 29.5, 29.58, 29.62, 29.64, 30.2 (CH_2), 31.9 ($CH_3CH_2CH_2$), 33.7 (SCH_2), 36.8 (NCH_3), 50.1 (NCH_2), 68.2 (NCH_3), 110.3 (C-1), 119.9 (C-5), 120.8 (C-3), 121.3 (C-4), 121.7 (Im), 123.2 (Im), 124.3 (C-8), 134.8 (C-6), 134.9 (C-8'), 135.7 (C-1'), 136.8 (C-4'), 137.5 (C-7), 138.1 (N*C*HN), 142.2 (C-5'), 160.1 (C-2), 193.6 (*C*=O) ppm; **FT-IR** (ATR): $\tilde{\nu}$ = 3405 (m), 3150 (w), 3085 (w), 2922 (vs), 2852 (s), 1711 (s), 1601 (m), 1573 (w), 1487 (m), 1457 (s), 1437 (m), 1393 (w), 1345 (w), 1289 (m), 1249 (m), 1227 (w), 1205 (w), 1168 (m), 1143 (w), 1100 (w), 1059 (w), 1030 (w), 1001 (w), 969 (w), 819 (w), 785 (m), 759 (w), 644 (w), 622 (w), 501 (w) cm^{-1}; **MS** (ESI): *m/z* = 561 $[M]^+$; **HRMS** (ESI): calcd. for $[C_{35}H_{49}N_2O_2S]^+$ 561.3509, found: 561.3496 $[M]^+$; **elemental analysis**: calcd. (%) for $C_{35}H_{49}BrN_2O_2S$: C 65.51, H 7.70, N 4.37, S 5.00, found: C 64.58, H 7.75, N 4.42, S 4.70; **DSC**: Cr 53 [37.9 kJ mol^{-1}] SmA 259 [1.4 kJ mol^{-1}] I (1. H).

3-(6-{[7-(Tetradecylthio)-9-oxo-9*H*-fluoren-2-yl]oxy}hexyl)-1-methyl-1*H*-imidazol-3-ium bromide [Im(O6,S14)Br]. According to GP7, from **Br(O6,S14)** (110 mg, 187 µmol), **16a** (22 µL, 23 mg, 281 µmol), abs. DMF (5 mL); yield: 101 mg, 151 µmol, 81%. **^{1}H-NMR** (500 MHz, $CDCl_3$): δ = 0.87 (t, $J = 6.9$ Hz, 3H, CH_3), 1.25 (s, 20H, CH_2), 1.37–1.50 (m, 4H, $SCH_2CH_2CH_2$, $OCH_2CH_2CH_2$), 1.49–1.58 (m, 2H, $NCH_2CH_2CH_2$), 1.64 (m_c, 2H, SCH_2CH_2), 1.74–1.83 (m, 2H,

OCH_2CH_2), 1.98 (m_c, 2H, NCH_2CH_2), 2.93 (t, J = 7.4 Hz, 2H, SCH_2), 3.98 (t, J = 6.3 Hz, 2H, OCH_2), 4.12 (s, 3H, NCH_3), 4.38 (t, J = 7.5 Hz, 2H, NCH_2), 6.95 (dd, $J_{3,4}$ = 8.2 Hz, $J_{1,3}$ = 2.5 Hz, 1H, 3-H), 7.09 (d, $J_{1,3}$ = 2.4 Hz, 1H, 1-H), 7.26–7.30 (m, 1H, 5-H), 7.31–7.37 (m, 2H, 4-H, 6-H), 7.38–7.41 (m, 1H, Im), 7.42–7.45 (m, 1H, Im), 7.49–7.51 (m, 1H, 8-H), 10.53 (s, 1H, NC*H*N) ppm; **^{13}C-NMR** (126 MHz, $CDCl_3$): δ = 14.1 (CH_3), 22.7 (CH_3CH_2), 25.5, 25.9, 28.8, 29.0, 29.2, 29.4, 29.5, 29.6, 29.65, 29.67, 29.68, 30.2 (CH_2), 31.9 ($CH_3CH_2CH_2$), 33.7 (SCH_2), 36.8 (NCH_3), 50.1 (NCH_2), 68.2 (NCH_3), 110.2 (C-1), 119.9 (C-5), 120.7 (C-3), 121.3 (C-4), 121.8 (Im), 123.3 (Im), 124.3 (C-8), 134.8 (C-6), 134.9 (C-8'), 135.6 (C-1'), 136.8 (C-4'), 137.5 (C-7), 137.9 (N*C*HN), 142.2 (C-5'), 160.0 (C-2), 193.6 (*C*=O) ppm; **FT-IR** (ATR): $\tilde{\nu}$ = 3411 (w), 3066 (w), 2916 (vs), 2850 (s), 1714 (s), 1599 (m), 1573 (w), 1489 (w), 1469 (m), 1457 (m), 1438 (m), 1390 (w), 1339 (w), 1294 (m), 1246 (m), 1226 (w), 1211 (w), 1169 (m), 1148 (w), 1091 (w), 1063 (w), 1030 (w), 1004 (w), 961 (w), 927 (w), 882 (w), 830 (m), 780 (m), 757 (w), 717 (w), 646 (w), 620 (w), 499 (m) cm^{-1}; **MS** (ESI): m/z = 589 $[M]^+$; **HRMS** (ESI): calcd. for $[C_{37}H_{53}N_2O_2S]^+$ 589.3822, found: 589.3809 $[M]^+$; **elemental analysis**: calcd. (%) for $C_{37}H_{53}BrN_2O_2S$: C 66.35, H 7.98, N 4.18, S 4.79, found: C 66.16, H 8.07, N 3.88, S 4.75; **DSC**: Cr 75 [50.6 kJ mol^{-1}] SmA 259 [1.0 kJ mol^{-1}] I (1. H).

3-(8-{[7-(Dodecylthio)-9-oxo-9*H*-fluoren-2-yl]oxy}octyl)-1-methyl-1*H*-imidazol-3-ium bromide [Im(O8,S12)Br]. According to GP7, from **Br(O8,S12)** (90 mg, 153 µmol), **16a** (18 µL, 19 mg, 230 µmol), abs. DMF (9 mL); yield: 84 mg, 125 µmol, 82%. **^{1}H-NMR** (400 MHz, $CDCl_3$): δ = 0.87 (t, J = 6.8 Hz, 3H, CH_3), 1.16–1.51 (m, 26H, CH_2), 1.65 (m_c, 2H, SCH_2CH_2), 1.72–1.82 (m, 2H, OCH_2CH_2), 1.88–2.00 (m, 2H, NCH_2CH_2), 2.93 (t, J = 7.4 Hz, 2H, SCH_2), 3.98 (t, J = 6.5 Hz, 2H, OCH_2), 4.13 (s, 3H, NCH_3), 4.34 (t, J = 7.4 Hz, 2H, NCH_2), 6.95 (dd, $J_{3,4}$ = 8.2 Hz, $J_{1,3}$ = 2.5 Hz, 1H, 3-H), 7.12 (d, $J_{1,3}$ = 2.4 Hz, 1H, 1-H), 7.26–7.31 (m, 1H, 5-H), 7.32–7.37 (m, 3H, 4-H, 6-H, Im), 7.42–7.44 (m, 1H, Im), 7.51 (d, $J_{6,8}$ = 1.7 Hz, 1H, 8-H), 10.59 (s, 1H, NC*H*N) ppm; **^{13}C-NMR** (101 MHz, $CDCl_3$): δ = 14.1 (CH_3), 22.7 (CH_3CH_2), 25.8, 26.2, 28.8, 28.9, 29.0, 29.2, 29.3, 29.5, 29.57, 29.62, 29.64, 30.3 (CH_2), 31.9 ($CH_3CH_2CH_2$), 33.7 (SCH_2), 36.8 (NCH_3), 50.2 (NCH_2), 68.5 (OCH_2), 110.2 (C-1), 119.9 (C-5), 120.9 (C-3), 121.3 (C-4), 121.7 (Im), 123.3 (Im), 124.3 (C-8), 134.8 (C-6), 135.0 (C-8'), 135.7 (C-1'), 136.7 (C-4'), 137.4 (C-7), 138.0 (N*C*HN), 142.3 (C-5'), 160.2 (C-2), 193.6 (*C*=O) ppm; **FT-IR** (ATR): $\tilde{\nu}$ = 3443 (w), 3052 (w), 2918 (s), 2848 (s), 1709 (s), 1600 (w), 1575 (w), 1488 (m), 1455 (s), 1431 (m), 1417 (m), 1391 (w), 1348 (w), 1294 (m), 1274 (m), 1247 (vs), 1226 (w), 1205 (w), 1173 (s), 1136 (w), 1091 (w), 1056 (w), 1034 (w), 1000 (w), 972 (w), 908 (m), 860 (w), 822 (m), 781 (m), 757 (m), 730 (vs), 643 (w), 622 (w), 553 (w), 498 (m) cm^{-1}; **MS** (ESI): m/z = 589 $[M]^+$; **HRMS** (ESI): calcd. for $[C_{37}H_{53}N_2O_2S]^+$ 589.3822, found: 589.3811 $[M]^+$; **elemental analysis**: calcd. (%) for $C_{37}H_{53}BrN_2O_2S$: C 66.35, H 7.98, N 4.18, S 4.79, found: C 65.41, H 7.98, N 4.25, S 4.59; **DSC**: Cr 80 [55.1 kJ mol^{-1}] SmA 234 [1.5 kJ mol^{-1}] I (1. H).

3-(8-{[7-(Tetradecylthio)-9-oxo-9*H*-fluoren-2-yl]oxy}octyl)-1-methyl-1*H*-imidazol-3-ium bromide [Im(O8,S14)Br]. According to GP7, from **Br(O8,S14)** (150 mg, 244 µmol), **16a** (29 µL, 30 mg, 366 µmol), abs. DMF (8 mL); yield: 138 mg, 198 µmol, 81%. **^{1}H-NMR** (500 MHz, $CDCl_3$):

δ = 0.88 (t, J = 6.9 Hz, 3H, CH_3), 1.19–1.32 (m, 20H, CH_2), 1.33–1.51 (m, 10H, CH_2), 1.64 (m_c, 2H, SCH_2CH_2), 1.73–1.81 (m, 2H, OCH_2CH_2), 1.90–2.00 (m, 2H, NCH_2CH_2), 2.93 (t, J = 7.4 Hz, 2H, SCH_2), 3.97 (t, J = 6.4 Hz, 2H, OCH_2), 4.13 (s, 3H, NCH_3), 4.34 (t, J = 7.4 Hz, 2H, NCH_2), 6.95 (dd, $J_{3,4}$ = 8.2 Hz, $J_{1,3}$ = 2.4 Hz, 1H, 3-H), 7.12 (d, $J_{1,3}$ = 2.4 Hz, 1H, 1-H), 7.27–7.31 (m, 1H, 5-H), 7.32–7.37 (m, 3H, 4-H, 6-H, Im), 7.43–7.46 (m, 1H, Im), 7.49–7.53 (m, 1H, 8-H), 10.57 (s, 1H, NC*H*N) ppm; **^{13}C-NMR** (126 MHz, $CDCl_3$): δ = 14.1 (CH_3), 22.7 (CH_3CH_2), 25.8, 26.2, 28.8, 28.9, 29.0, 29.2, 29.4, 29.5, 29.57, 29.64, 29.66, 29.68, 30.3 (CH_2), 31.9 ($CH_3CH_2CH_2$), 33.7 (SCH_2), 36.8 (NCH_3), 50.2 (NCH_2), 68.4 (OCH_2), 110.2 (C-1), 119.9 (C-5), 120.8 (C-3), 121.3 (C-4), 121.7 (Im), 123.3 (Im), 124.3 (C-8), 134.8 (C-6), 134.9 (C-8'), 135.6 (C-1'), 136.7 (C-4'), 137.4 (C-7), 138.0 (N*C*HN), 142.3 (C-5'), 160.2 (C-2), 193.6 (*C*=O) ppm; **FT-IR** (ATR): $\tilde{\nu}$ = 3418 (w), 3066 (w), 2916 (vs), 2850 (s), 1714 (s), 1601 (m), 1574 (w), 1489 (m), 1470 (m), 1438 (m), 1392 (w), 1338 (w), 1295 (m), 1247 (m), 1173 (m), 1091 (w), 1062 (w), 1035 (w), 998 (w), 961 (w), 881 (w), 860 (w), 831 (m), 780 (m), 757 (m), 717 (m), 645 (w), 620 (w), 557 (w), 498 (m) cm^{-1}; **MS** (ESI): m/z = 617 $[M]^+$; **HRMS** (ESI): calcd. for $[C_{39}H_{57}N_2O_2S]^+$ 617.4135, found: 617.4134 $[M]^+$; **elemental analysis**: calcd. (%) for $C_{39}H_{57}BrN_2O_2S$: C 67.12, H 8.23, N 4.01, S 4.59, found: C 66.46, H 8.23, N 4.03, S 4.08; **DSC**: Cr 83 [58.6 kJ mol^{-1}] SmC 98 [via POM] SmA 240 [1.0 kJ mol^{-1}] I (1. H).

3-(10-{[7-(Decylthio)-9-oxo-9*H*-fluoren-2-yl]oxy}decyl)-1-methyl-1*H*-imidazol-3-ium bromide [Im(O10,S10)Br]. According to GP7, from **Br(O10,S10)** (150 mg, 244 µmol), **16a** (29 µL, 30 mg, 366 µmol), abs. DMF (8 mL); yield: 138 mg, 198 µmol, 81%. **^{1}H-NMR** (500 MHz, $CDCl_3$): δ = 0.87 (t, J = 6.8 Hz, 3H, CH_3), 1.19–1.51 (m, 26H, CH_2), 1.65 (m_c, 2H, SCH_2CH_2), 1.77 (m_c, 2H, OCH_2CH_2), 1.93 (m_c, 2H, NCH_2CH_2), 2.93 (t, J = 7.4 Hz, 2H, SCH_2), 3.98 (t, J = 6.6 Hz, 2H, OCH_2), 4.13 (s, 3H, NCH_3), 4.32 (t, J = 7.4 Hz, 2H, NCH_2), 6.96 (dd, $J_{3,4}$ = 8.2 Hz, $J_{1,3}$ = 2.3 Hz, 1H, 3-H), 7.14 (d, $J_{1,3}$ = 2.3 Hz, 1H, 1-H), 7.26–7.30 (m, 1H, 5-H), 7.30–7.38 (m, 3H, 4-H, 6-H, Im), 7.39–7.42 (m, 1H, Im), 7.50–7.54 (m, 1H, 8-H), 10.61 (s, 1H, NC*H*N) ppm; **^{13}C-NMR** (126 MHz, $CDCl_3$): δ = 14.1 (CH_3), 22.7 (CH_3CH_2), 25.9, 26.3, 28.8, 28.9, 29.0, 29.1, 29.15, 29.19, 29.23, 29.30, 29.32, 29.50, 29.52, 30.3 (CH_2), 31.9 ($CH_3CH_2CH_2$), 33.7 (SCH_2), 36.8 (NCH_3), 50.2 (NCH_2), 68.6 (OCH_2), 110.1 (C-1), 119.9 (C-5), 120.9 (C-3), 121.2 (C-4), 121.6 (Im), 123.2 (Im), 124.3 (C-8), 134.8 (C-6), 135.0 (C-8'), 135.6 (C-1'), 136.7 (C-4'), 137.4 (C-7), 138.0 (N*C*HN), 142.3 (C-5'), 160.2 (C-2), 193.6 (*C*=O) ppm; **FT-IR** (ATR): $\tilde{\nu}$ = 3444 (w), 3055 (w), 2919 (s), 2850 (m), 1710 (s), 1601 (m), 1575 (w), 1488 (m), 1468 (m), 1456 (m), 1434 (m), 1419 (w), 1391 (w), 1342 (w), 1290 (m), 1248 (s), 1173 (m), 1137 (w), 1092 (w), 1057 (w), 1032 (w), 972 (w), 909 (m), 860 (w), 822 (m), 782 (m), 758 (m), 732 (vs), 644 (w), 622 (w), 554 (w), 499 (w) cm^{-1}; **MS** (ESI): m/z = 589 $[M]^+$; **HRMS** (ESI): calcd. for $[C_{37}H_{53}N_2O_2S]^+$ 589.3822, found: 589.3843 $[M]^+$; **elemental analysis**: calcd. (%) for $C_{37}H_{53}BrN_2O_2S$: C 66.35, H 7.98, N 4.18, S 4.79, found: C 65.76, H 8.05, N 4.14, S 4.68; **DSC**: Cr 78 [45.9 kJ mol^{-1}] SmC 95 [via POM] SmA 186 [2.1 kJ mol^{-1}] I (3. H); I 186 [–2.1 kJ mol^{-1}] SmA 93 [via POM] SmC 54 [–44.5 kJ mol^{-1}] Cr (3. C).

3-(10-({[7-(Dodecylthio)-9-oxo-9*H*-fluoren-2-yl]oxy}decyl)-1-methyl-1*H*-imidazol-3-ium bromide [Im(O10,S12)Br]. According to GP7, from **Br(O10,S12)** (100 mg, 162 µmol), **16a** (20 µL, 20 mg, 243 µmol), abs. DMF (8 mL); yield: 104 mg, 149 µmol, 81%. **^{1}H-NMR** (400 MHz, $CDCl_3$): δ = 0.87 (t, *J* = 6.7 Hz, 3H, CH_3), 1.18–1.52 (m, 30H, CH_2), 1.65 (m_c, 2H, SCH_2CH_2), 1.77 (m_c, 2H, OCH_2CH_2), 1.93 (m_c, 2H, NCH_2CH_2), 2.93 (t, *J* = 7.4 Hz, 2H, SCH_2), 3.98 (t, *J* = 6.6 Hz, 2H, OCH_2), 4.13 (s, 3H, NCH_3), 4.32 (t, *J* = 7.3 Hz, 2H, NCH_2), 6.96 (dd, $J_{3,4}$ = 8.2 Hz, $J_{1,3}$ = 2.4 Hz, 1H, 3-H), 7.14 (d, $J_{1,3}$ = 2.4 Hz, 1H, 1-H), 7.26–7.32 (m, 2H, 4-H, 5-H), 7.32–7.38 (m, 2H, 6-H, Im), 7.38–7.41 (m, 1H, Im), 7.50–7.54 (m, 1H, 8-H), 10.62 (s, 1H, NC*H*N) ppm; **^{13}C-NMR** (101 MHz, $CDCl_3$): δ = 14.1 (CH_3), 22.7 (CH_3CH_2), 25.9, 26.2, 28.8, 28.9, 29.0, 29.07, 29.14, 29.18, 29.21, 29.31, 29.33, 29.5, 29.56, 29.61, 29.63, 30.3 (CH_2), 31.9 ($CH_3CH_2CH_2$), 33.7 (SCH_2), 36.8 (NCH_3), 50.2 (NCH_2), 68.6 (OCH_2), 110.2 (C-1), 119.8 (C-5), 120.9 (C-3), 121.2 (C-4), 121.6 (Im), 123.2 (C-8), 124.3 (C-8), 134.8 (C-6), 135.0 (C-8'), 135.7 (C-1'), 136.7 (C-4'), 137.4 (C-7), 138.1 (N*C*HN), 142.3 (C-5'), 160.2 (C-2), 193.6 (*C*=O) ppm; **FT-IR** (ATR): $\tilde{\nu}$ = 3450 (w), 3052 (w), 2919 (vs), 2850 (s), 1711 (s), 1601 (m), 1576 (w), 1488 (m), 1470 (m), 1457 (m), 1433 (w), 1331 (w), 1295 (m), 1250 (m), 1205 (w), 1175 (m), 1137 (w), 1092 (w), 1032 (w), 972 (w), 892 (w), 860 (w), 822 (m), 782 (m), 759 (m), 720 (w), 643 (w), 622 (w), 554 (w), 499 (w) cm^{-1}; **MS** (ESI): *m/z* = 617 $[M]^+$; **HRMS** (ESI): calcd. for $[C_{39}H_{57}N_2O_2S]^+$ 617.4135, found: 617.4140 $[M]^+$; **elemental analysis**: calcd. (%) for $C_{39}H_{57}BrN_2O_2S$: C 67.12, H 8.23, N 4.01, S 4.59, found: C 65.46, H 8.33, N 4.09, S 4.32; **DSC**: Cr 83 [58.3 kJ mol^{-1}] SmC 100 [via POM] SmA 208 [1.5 kJ mol^{-1}] I (1. H); I 207 [–1.8 kJ mol^{-1}] SmA 99 [via POM] SmC 77 [–53.5 kJ mol^{-1}] Cr (1. C).

3-(10-{[7-(Tetradecylthio)-9-oxo-9*H*-fluoren-2-yl]oxy}decyl)-1-methyl-1*H*-imidazol-3-ium bromide [Im(O10,S14)Br]. According to GP7, from **Br(O10,S14)** (130 mg, 202 µmol), **16a** (24 µL, 25 mg, 303 µmol), abs. DMF (9 mL); yield: 89 mg, 123 µmol, 61%. **^{1}H-NMR** (400 MHz, $CDCl_3$): δ = 0.87 (t, *J* = 6.7 Hz, 3H, CH_3), 1.20–1.39 (m, 30H, CH_2), 1.39–1.50 (m, 4H, $OCH_2CH_2CH_2$, $NCH_2CH_2CH_2$), 1.65 (m_c, 2H, SCH_2CH_2), 1.77 (m_c, 2H, OCH_2CH_2), 1.92 (m_c, 2H, NCH_2CH_2), 2.93 (t, *J* = 7.4 Hz, 2H, SCH_2), 3.98 (t, *J* = 6.6 Hz, 2H, OCH_2), 4.13 (s, 3H, NCH_3), 4.32 (t, *J* = 7.3 Hz, 2H, NCH_2), 6.96 (dd, $J_{3,4}$ = 8.2 Hz, $J_{1,3}$ = 2.4 Hz, 1H, 3-H), 7.14 (d, $J_{1,3}$ = 2.4 Hz, 1H, 1-H), 7.26–7.32 (m, 2H, 4-H, 5-H), 7.32–7.38 (m, 2H, 6-H, Im), 7.39–7.43 (m, 1H, Im), 7.50–7.53 (m, 1H, 8-H), 10.58 (s, 1H, NC*H*N) ppm; **^{13}C-NMR** (101 MHz, $CDCl_3$): δ = 14.1 (CH_3), 22.7 (CH_3CH_2), 25.9, 26.2, 28.8, 28.9, 29.0, 29.07, 29.14, 29.18, 29.21, 29.31, 29.34, 29.5, 29.56, 29.63, 29.65, 29.67, 30.3 (CH_2), 31.9 ($CH_3CH_2CH_2$), 33.7 (SCH_2), 36.8 (NCH_3), 50.2 (NCH_2), 68.6 (OCH_2), 110.2 (C-1), 119.8 (C-5), 120.9 (C-3), 121.2 (C-4), 121.6 (Im), 123.2 (C-8), 124.3 (C-8), 134.8 (C-6), 135.0 (C-8'), 135.7 (C-1'), 136.7 (C-4'), 137.4 (C-7), 138.0 (N*C*HN), 142.3 (C-5'), 160.2 (C-2), 193.6 (*C*=O) ppm; **FT-IR** (ATR): $\tilde{\nu}$ = 3448 (w), 3054 (w), 2916 (vs), 2849 (s), 1710 (s), 1601 (m), 1576 (w), 1488 (m), 1470 (m), 1456 (m), 1432 (w), 1331 (w), 1297 (m), 1248 (s), 1205 (w), 1174 (m), 1136 (w), 1091 (w), 1032 (w), 973 (w), 892 (w), 860 (w), 822 (m), 781 (m), 758 (m), 719 (w), 643 (w), 622 (w), 554 (w), 499 (w) cm^{-1}; **MS** (ESI): *m/z* = 645 $[M]^+$; **HRMS** (ESI): calcd. for $[C_{41}H_{61}N_2O_2S]^+$ 645.4448, found: 645.4426 $[M]^+$; **elemental**

analysis: calcd. (%) for $C_{41}H_{61}BrN_2O_2S$: C 67.84, H 8.47, N 3.86, S 4.42, found: C 66.78, H 8.48, N 3.81, S 4.13; **DSC**: Cr 93 [67.9 kJ mol^{-1}] SmC 105 [via POM] SmA 221 [1.5 kJ mol^{-1}] I (1. H); I 209 [–1.2 kJ mol^{-1}] SmA 100 [via POM] SmC 61 [–35.9 kJ mol^{-1}] Cr (1. C).

3-(12-{[7-(Decylthio)-9-oxo-9*H*-fluoren-2-yl]oxy}dodecyl)-1-methyl-1*H*-imidazol-3-ium bromide [Im(O12,S10)Br]. According to GP7, from **Br(O12,S10)** (180 mg, 292 µmol), **16a** (35 µL, 36 mg, 438 µmol), abs. DMF (10 mL); yield: 178 mg, 255 µmol, 87%. **^{1}H-NMR** (500 MHz, $CDCl_3$): δ = 0.87 (t, *J* = 6.9 Hz, 3H, C*H*$_3$), 1.22–1.38 (m, 26H, C*H*$_2$), 1.38–1.49 (m, 4H, $OCH_2CH_2CH_2$, $NCH_2CH_2CH_2$), 1.65 (m$_c$, 2H, SCH_2CH_2), 1.73–1.83 (m, 2H, OCH_2CH_2), 1.92 (m$_c$, 2H, NCH_2CH_2), 2.93 (t, *J* = 7.4 Hz, 2H, SC*H*$_2$), 3.98 (t, *J* = 6.5 Hz, 2H, OC*H*$_2$), 4.14 (s, 3H, NC*H*$_3$), 4.32 (t, *J* = 7.4 Hz, 2H, NC*H*$_2$), 6.96 (dd, $J_{3,4}$ = 8.2 Hz, $J_{1,3}$ = 2.4 Hz, 1H, 3-H), 7.15 (d, $J_{1,3}$ = 2.4 Hz, 1H, 1-H), 7.27–7.31 (m, 1H, 5-H), 7.31–7.38 (m, 3H, 4-H, 6-H, Im), 7.42–7.45 (m, 1H, Im), 7.51 (d, $J_{6,8}$ = 1.8 Hz, 1H, 8-H), 10.57 (s, 1H, NC*H*N) ppm; **^{13}C-NMR** (126 MHz, $CDCl_3$): δ = 14.1 (*C*H$_3$), 22.7 (CH_3CH_2), 26.0, 26.3, 28.8, 28.98, 29.00, 29.1, 29.2, 29.30, 29.34, 29.4, 29.45, 29.47, 29.50, 29.53, 30.3 (*C*H$_2$), 31.9 ($CH_3CH_2CH_2$), 33.7 (S*C*H$_2$), 36.8 (N*C*H$_3$), 50.2 (N*C*H$_2$), 68.6 (O*C*H$_2$), 110.1 (C-1), 119.9 (C-5), 120.9 (C-3), 121.2 (C-4), 121.6 (Im), 123.3 (C-8), 124.3 (C-8), 134.8 (C-6), 135.0 (C-8'), 135.6 (C-1'), 136.6 (C-4'), 137.3 (C-7), 138.0 (N*C*HN), 142.3 (C-5'), 160.2 (C-2), 193.7 (*C*=O) ppm; **FT-IR** (ATR): $\tilde{\nu}$ = 3457 (w), 3054 (w), 2918 (vs), 2850 (s), 1711 (s), 1601 (m), 1576 (w), 1488 (m), 1470 (m), 1457 (m), 1434 (m), 1332 (w), 1291 (m), 1249 (s), 1205 (w), 1174 (m), 1137 (w), 1092 (w), 1034 (w), 972 (w), 908 (m), 861 (w), 822 (m), 781 (m), 758 (m), 732 (m), 643 (w), 622 (w), 553 (w), 499 (w) cm^{-1}; **MS** (ESI): *m/z* = 617 $[M]^+$; **HRMS** (ESI): calcd. for $[C_{39}H_{57}N_2O_2S]^+$ 617.4135, found: 617.4110 $[M]^+$; **elemental analysis**: calcd. (%) for $C_{39}H_{57}BrN_2O_2S$: C 67.12, H 8.23, N 4.01, S 4.59, found: C 66.52, H 8.19, N 4.03, S 4.49; **DSC**: Cr 89 [54.6 kJ mol^{-1}] SmC 102 [via POM] SmA 165 [3.5 kJ mol^{-1}] I (3. H); I 165 [–1.8 kJ mol^{-1}] SmA 101 [via POM] SmC 70 [–56.1 kJ mol^{-1}] Cr (3. C).

3-(12-{[7-(Dodecylthio)-9-oxo-9*H*-fluoren-2-yl]oxy}dodecyl)-1-methyl-1*H*-imidazol-3-ium bromide [Im(O12,S12)Br]. According to GP7, from **Br(O12,S12)** (150 mg, 233 µmol), **16a** (28 µL, 29 mg, 350 µmol), abs. DMF (15 mL); yield: 106 mg, 146 µmol, 63%. **^{1}H-NMR** (500 MHz, $CDCl_3$): δ = 0.87 (t, *J* = 6.9 Hz, 3H, C*H*$_3$), 1.19–1.49 (m, 34H, C*H*$_2$), 1.65 (m$_c$, 2H, SCH_2CH_2), 1.78 (m$_c$, 2H, OCH_2CH_2), 1.92 (m$_c$, 2H, NCH_2CH_2), 2.93 (t, *J* = 7.4 Hz, 2H, SC*H*$_2$), 3.99 (t, *J* = 6.6 Hz, 2H, OC*H*$_2$), 4.13 (s, 3H, NC*H*$_3$), 4.32 (t, *J* = 7.5 Hz, 2H, NC*H*$_2$), 6.96 (dd, $J_{3,4}$ = 8.2 Hz, $J_{1,3}$ = 2.5 Hz, 1H, 3-H), 7.15 (d, $J_{1,3}$ = 2.5 Hz, 1H, 1-H), 7.28–7.31 (m, 2H, 4-H, 5-H), 7.33–7.37 (m, 2H, 6-H, Im), 7.38–7.40 (m, 1H, Im), 7.52 (d, $J_{6,8}$ = 1.8 Hz, 1H, 8-H), 10.60 (s, 1H, NC*H*N) ppm; **^{13}C-NMR** (126 MHz, $CDCl_3$): δ = 14.1 (*C*H$_3$), 22.7 (CH_3CH_2), 25.9, 26.3, 28.8, 28.97, 29.01, 29.1, 29.2, 29.29, 29.34, 29.42, 29.44, 29.46, 29.50, 29.58, 29.63, 29.7, 30.3 (*C*H$_2$), 31.9 ($CH_3CH_2CH_2$), 33.7 (S*C*H$_2$), 36.8 (N*C*H$_3$), 50.3 (N*C*H$_2$), 68.6 (O*C*H$_2$), 110.1 (C-1), 119.9 (C-5), 120.9 (C-3), 121.2 (C-4), 121.6 (Im), 123.2 (C-8), 124.3 (C-8), 134.8 (C-6), 135.0 (C-8'), 135.7 (C-1'), 136.7 (C-4'), 137.4 (C-7), 138.0 (N*C*HN), 142.3 (C-5'), 160.3 (C-2), 193.6 (*C*=O) ppm; **FT-IR** (ATR): $\tilde{\nu}$ = 3448 (w), 3054 (w), 2918 (vs), 2851 (s), 1713 (s), 1601 (m), 1576 (w), 1488 (m), 1470 (m), 1458 (m), 1433 (w), 1392 (w), 1295 (m), 1249 (m), 1206 (w), 1174 (m),

1137 (w), 1092 (w), 1034 (w), 1008 (w), 972 (w), 892 (w), 861 (w), 822 (m), 780 (m), 759 (m), 719 (w), 643 (w), 622 (w), 555 (w), 499 (w) cm^{-1}; **MS** (ESI): *m/z* = 645 $[M]^+$; **HRMS** (ESI): calcd. for $[C_{41}H_{61}N_2O_2S]^+$ 645.4448, found: 645.4457 $[M]^+$; **elemental analysis**: calcd. (%) for $C_{41}H_{61}BrN_2O_2S$: C 67.84, H 8.47, N 3.86, S 4.42, found: C 66.42, H 8.56, N 3.97, S 4.16; **DSC**: Cr 98 [45.1 kJ mol^{-1}] SmC 110 [via POM] SmA 172 [3.3 kJ mol^{-1}] I (3. H); I 171 [–3.7 kJ mol^{-1}] SmA 109 [via POM] SmC 79 [–48.7 kJ mol^{-1}] Cr (3. C).

3-(12-{[7-(Tetradecylthio)-9-oxo-9*H*-fluoren-2-yl]oxy}dodecyl)-1-methyl-1*H*-imidazol-3-ium bromide [Im(O12,S14)Br]. According to GP7, from **Br(O12,S14)** (100 mg, 149 µmol), **16a** (17 µL, 18 mg, 224 µmol), abs. DMF (5 mL); yield: 90 mg, 119 µmol, 80%. **^{1}H-NMR** (400 MHz, $CDCl_3$): δ = 0.87 (t, *J* = 6.7 Hz, 3H, CH_3), 1.20–1.51 (m, 38H, CH_2), 1.65 (m_c, 2H, SCH_2CH_2), 1.78 (m_c, 2H, OCH_2CH_2), 1.92 (m_c, 2H, NCH_2CH_2), 2.93 (t, *J* = 7.4 Hz, 2H, SCH_2), 3.99 (t, *J* = 6.6 Hz, 2H, OCH_2), 4.13 (s, 3H, NCH_3), 4.32 (t, *J* = 7.4 Hz, 2H, NCH_2), 6.96 (dd, $J_{3,4}$ = 8.1 Hz, $J_{1,3}$ = 2.3 Hz, 1H, 3-H), 7.15 (d, $J_{1,3}$ = 2.3 Hz, 1H, 1-H), 7.26–7.31 (m, 2H, 4-H, 5-H), 7.32–7.40 (m, 3H, 6-H, Im), 7.52 (d, $J_{6,8}$ = 1.7 Hz, 1H, 8-H), 10.59 (s, 1H, NC*H*N) ppm; **^{13}C-NMR** (101 MHz, $CDCl_3$): δ = 14.1 (CH_3), 22.7 (CH_3CH_2), 25.9, 26.3, 28.8, 28.95, 29.01, 29.11, 29.14, 29.27, 29.32, 29.34, 29.41, 29.43, 29.45, 29.48, 29.56, 29.63, 29.65, 29.67, 30.3 (CH_2), 31.9 ($CH_3CH_2CH_2$), 33.7 (SCH_2), 36.8 (NCH_3), 50.3 (NCH_2), 68.6 (OCH_2), 110.1 (C-1), 119.8 (C-5), 120.9 (C-3), 121.2 (C-4), 121.5 (Im), 123.2 (C-8), 124.3 (C-8), 134.8 (C-6), 135.0 (C-8'), 135.7 (C-1'), 136.7 (C-4'), 137.4 (C-7), 138.1 (N*C*HN), 142.3 (C-5'), 160.3 (C-2), 193.6 (*C*=O) ppm; **FT-IR** (ATR): $\tilde{\nu}$ = 3446 (w), 3052 (w), 2916 (vs), 2849 (s), 1710 (s), 1601 (m), 1575 (w), 1488 (w), 1470 (m), 1457 (m), 1433 (w), 1392 (w), 1296 (m), 1248 (s), 1205 (w), 1173 (m), 1136 (w), 1092 (w), 1034 (w), 1008 (w), 972 (w), 892 (w), 860 (w), 822 (m), 780 (m), 758 (m), 718 (m), 643 (w), 622 (w), 554 (w), 499 (w) cm^{-1}; **MS** (ESI): *m/z* = 673 $[M]^+$; **HRMS** (ESI): calcd. for $[C_{43}H_{65}N_2O_2S]^+$ 673.4761, found: 673.4743 $[M]^+$; **elemental analysis**: calcd. (%) for $C_{43}H_{65}BrN_2O_2S$: C 68.50, H 8.69, N 3.72, S 4.25, found: C 66.68, H 8.83, N 3.68, S 3.92; **DSC**: Cr 98 [74.0 kJ mol^{-1}] SmC 111 [via POM] SmA 188 [1.8 kJ mol^{-1}] I (1. H); I 188 [–1.9 kJ mol^{-1}] SmA 108 [via POM] SmC 82 [–59.1 kJ mol^{-1}] Cr (1. C).

3-(4-{[7-(Dodecylthio)-9-oxo-9*H*-fluoren-2-yl]oxy}butyl)-1,2-dimethyl-1*H*-imidazol-3-ium bromide [ImMe(O4,S12)Br]. According to GP7, from **Br(O4,S12)** (120 mg, 226 µmol), **16b** (33 mg, 339 µmol), abs. DMF (8 mL); yield: 68 mg, 108 µmol, 48%. **^{1}H-NMR** (400 MHz, $CDCl_3$): δ = 0.87 (t, *J* = 6.7 Hz, 3H, CH_3), 1.18–1.36 (m, 16H, CH_2), 1.37–1.47 (m, 2H, $SCH_2CH_2CH_2$), 1.64 (m_c, 2H, SCH_2CH_2), 1.84–1.98 (m, 2H, OCH_2CH_2), 2.09 (m_c, 2H, NCH_2CH_2), 2.85 (s, 3H, $NCCH_3$), 2.93 (t, *J* = 7.4 Hz, 2H, SCH_2), 4.00 (s, 3H, NCH_3), 4.05 (t, *J* = 5.9 Hz, 2H, OCH_2), 4.38 (t, *J* = 7.5 Hz, 2H, NCH_2), 6.95 (dd, $J_{3,4}$ = 8.2 Hz, $J_{1,3}$ = 2.5 Hz, 1H, 3-H), 7.06 (d, $J_{1,3}$ = 2.5 Hz, 1H, 1-H), 7.25–7.29 (m, 1H, 5-H), 7.30–7.37 (m, 2H, 4-H, 6-H), 7.49 (d, $J_{6,8}$ = 1.7 Hz, 1H, 8-H), 7.65 (d, *J* = 2.1 Hz, 1H, Im), 7.68 (d, *J* = 2.1 Hz, 1H, Im) ppm; **^{13}C-NMR** (101 MHz, $CDCl_3$): δ = 11.1 ($NCCH_3$), 14.1 (CH_2CH_3), 22.7, 26.0, 26.9, 28.8, 29.0, 29.2, 29.3, 29.5, 29.58, 29.62, 29.64, 31.9 (CH_2), 33.7 (SCH_2), 36.2 (NCH_3), 48.7 (NCH_2), 67.7 (OCH_2), 110.4 (C-1), 120.0 (C-5), 120.5 (C-3), 121.37 (Im), 121.40 (C-4), 122.9 (Im), 124.2 (C-

8), 134.7 (C-6), 134.9 (C-8'), 135.7 (C-1'), 137.0 (C-4'), 137.7 (C-7), 142.0 (C-5'), 144.0 (N*C*CH_2), 159.6 (C-2), 193.4 (*C*=O) ppm; **FT-IR** (ATR): $\tilde{\nu}$ = 3466 (w), 3387 (w), 3076 (w), 2954 (m), 2917 (vs), 2851 (s), 1710 (s), 1601 (m), 1540 (w), 1487 (w), 1469 (m), 1457 (s), 1437 (m), 1420 (m), 1391 (w), 1294 (m), 1249 (s), 1206 (w), 1178 (w), 1139 (w), 1094 (w), 1031 (w), 964 (w), 909 (w), 889 (w), 861 (w), 814 (w), 781 (m), 757 (m), 735 (m), 659 (w), 644 (w), 581 (w), 496 (w) cm^{-1}; **MS** (ESI): *m/z* = 547 $[M]^+$; **HRMS** (ESI): calcd. for $[C_{34}H_{47}N_2O_2S]^+$ 547.3353, found: 547.3370 $[M]^+$; **elemental analysis**: calcd. (%) for $C_{34}H_{47}BrN_2O_2S$: C 65.06, H 7.55, N 4.46, S 5.11, found: C 63.26, H 7.65, N 4.38, S 4.87; **DSC**: Cr 78 [48.4 kJ mol^{-1}] SmA 295 [20.3 kJ mol^{-1}] I (1. H).

3-(4-{[7-(Tetradecylthio)-9-oxo-9*H*-fluoren-2-yl]oxy}butyl)-1,2-dimethyl-1*H*-imidazol-3-ium bromide [ImMe(O4,S14)Br]. According to GP7, from **Br(O4,S14)** (50 mg, 89 µmol), **16b** (13 mg, 134 µmol), abs. DMF (5 mL); yield: 38 mg, 58 µmol, 65%. **^{1}H-NMR** (700 MHz, $CDCl_3$): δ = 0.87 (t, *J* = 7.0 Hz, 3H, C*H_3*), 1.21–1.33 (m, 20H, C*H_2*), 1.42 (m_c, 2H, SCH_2CH_2C*H_2*), 1.64 (m_c, 2H, SCH_2C*H_2*), 1.89–1.96 (m, 2H, OCH_2C*H_2*), 2.09 (m_c, 2H, NCH_2C*H_2*), 2.86 (s, 3H, NCC*H_3*), 2.93 (t, *J* = 7.4 Hz, 2H, SC*H_2*), 4.01 (s, 3H, NC*H_3*), 4.05 (t, *J* = 5.9 Hz, 2H, OC*H_2*), 4.39 (t, *J* = 7.5 Hz, 2H, NC*H_2*), 6.95 (dd, $J_{3,4}$ = 8.2 Hz, $J_{1,3}$ = 2.4 Hz, 1H, 3-H), 7.06 (d, $J_{1,3}$ = 2.4 Hz, 1H, 1-H), 7.27 (d, $J_{5,6}$ = 8.2 Hz, 1H, 5-H), 7.31–7.36 (m, 2H, 4-H, 6-H), 7.48 (d, $J_{6,8}$ = 1.6 Hz, 1H, 8-H), 7.68 (d, *J* = 2.0 Hz, 1H, Im), 7.71 (d, *J* = 2.0 Hz, 1H, Im) ppm; **^{13}C-NMR** (126 MHz, $CDCl_3$): δ = 11.2 (NC*C*H_3), 14.1 (CH_2*C*H_3), 22.7, 26.0, 26.9, 28.8, 29.0, 29.2, 29.4, 29.5, 29.6, 29.65, 29.67, 29.69, 31.9 (*C*H_2), 33.6 (S*C*H_2), 36.2 (N*C*H_3), 48.7 (N*C*H_2), 67.6 (O*C*H_2), 110.3 (C-1), 120.0 (C-5), 120.5 (C-3), 121.37 (Im), 121.41 (C-4), 122.9 (Im), 124.2 (C-8), 134.7 (C-6), 134.9 (C-8'), 135.6 (C-1'), 137.0 (C-4'), 137.7 (C-7), 142.0 (C-5'), 144.0 (N*C*CH_2), 159.6 (C-2), 193.4 (*C*=O) ppm; **FT-IR** (ATR): $\tilde{\nu}$ = 2923 (m), 2852 (m), 1710 (m), 1600 (w), 1538 (w), 1487 (m), 1455 (m), 1437 (m), 1390 (w), 1287 (m), 1246 (m), 1226 (w), 1205 (w), 1181 (w), 1141 (w), 1100 (w), 1032 (w), 964 (w), 907 (s), 821 (w), 784 (m), 726 (vs), 642 (m), 549 (w), 498 (w) cm^{-1}; **MS** (ESI): *m/z* = 575 $[M]^+$; **HRMS** (ESI): calcd. for $[C_{36}H_{51}N_2O_2S]^+$ 575.3666, found: 575.3667 $[M]^+$; **elemental analysis**: calcd. (%) for $C_{36}H_{51}BrN_2O_2S$: C 65.94, H 7.84, N 4.27, S 4.89, found: C 64.54, H 7.93, N 4.54, S 4.62; **DSC**: Cr 81 [60.3 kJ mol^{-1}] SmA 287 [18.3 kJ mol^{-1}] I (1. H).

3-(6-{[7-(Dodecylthio)-9-oxo-9*H*-fluoren-2-yl]oxy}hexyl)-1,2-dimethyl-1*H*-imidazol-3-ium bromide [ImMe(O6,S12)Br]. According to GP7, from **Br(O6,S12)** (95 mg, 169 µmol), **16b** (24 mg, 254 µmol), abs. DMF (4 mL); yield: 75 mg, 114 µmol, 68%. **^{1}H-NMR** (700 MHz, $CDCl_3$): δ = 0.84–0.90 (m, 3H, C*H_3*), 1.21–1.33 (m, 16H, C*H_2*), 1.39–1.57 (m, 6H, SCH_2CH_2C*H_2*, OCH_2CH_2C*H_2*, NCH_2CH_2C*H_2*), 1.61–1.68 (m, 2H, SCH_2C*H_2*), 1.79 (m_c, 2H, OCH_2C*H_2*), 1.85–1.92 (m, 2H, NCH_2C*H_2*), 2.83 (s, 3H, NCC*H_3*), 2.91–2.96 (m, 2H, SC*H_2*), 3.96–4.00 (m, 2H, OC*H_2*), 4.02 (s, 3H, NC*H_3*), 4.26 (t, *J* = 7.5 Hz, 2H, NC*H_2*), 6.92–6.97 (m, 1H, 3-H), 7.08–7.11 (m, 1H, 1-H), 7.27–7.30 (m, 1H, 5-H), 7.32–7.37 (m, 2H, 4-H, 6-H), 7.49–7.51 (m, 1H, 8-H), 7.55–7.58 (m, 1H, Im), 7.69–7.72 (m, 1H, Im) ppm; **^{13}C-NMR** (176 MHz, $CDCl_3$): δ = 11.1 (NC*C*H_3), 14.1 (CH_2*C*H_3), 22.7, 25.6, 26.1, 28.80, 28.83, 29.0, 29.2, 29.3, 29.5, 29.57, 29.62, 29.64, 29.8, 31.9 (*C*H_2), 33.7 (S*C*H_2), 36.2 (N*C*H_3), 48.9 (N*C*H_2), 68.1 (O*C*H_2), 110.1 (C-1),

119.9 (C-5), 120.8 (C-3), 121.2 (Im), 121.3 (C-4), 123.0 (Im), 124.2 (C-8), 134.7 (C-6), 134.9 (C-8'), 135.6 (C-1'), 136.8 (C-4'), 137.5 (C-7), 142.2 (C-5'), 143.8 (N*C*CH_2), 160.0 (C-2), 193.6 (*C*=O) ppm; **FT-IR** (ATR): $\tilde{\nu}$ = 3410 (w), 3066 (w), 2921 (vs), 2851 (m), 1711 (s), 1600 (m), 1538 (w), 1487 (m), 1455 (vs), 1436 (s), 1419 (m), 1393 (w), 1288 (s), 1246 (s), 1226 (m), 1205 (w), 1182 (w), 1142 (w), 1029 (w), 1000 (w), 968 (w), 908 (w), 819 (w), 785 (m), 758 (m), 733 (s), 666 (m), 643 (w), 501 (w) cm^{-1}; **MS** (ESI): *m/z* = 575 $[M]^+$; **HRMS** (ESI): calcd. for $[C_{36}H_{51}N_2O_2S]^+$ 575.3666, found: 575.3636 $[M]^+$; **elemental analysis**: calcd. (%) for $C_{36}H_{51}BrN_2O_2S$: C 65.94, H 7.84, N 4.27, S 4.89, found: C 65.76, H 7.97, N 4.26, S 4.74; **DSC**: Cr 78 [23.3 kJ mol^{-1}] 85 [via POM] SmA 287 [18.3 kJ mol^{-1}] I (1. H).

3-(6-{[7-(Tetradecylthio)-9-oxo-9*H*-fluoren-2-yl]oxy}hexyl)-1,2-dimethyl-1*H*-imidazol-3-ium bromide [ImMe(O6,S14)Br]. According to GP7, from **Br(O6,S14)** (100 mg, 170 µmol), **16b** (25 mg, 255 µmol), abs. DMF (4 mL); yield: 54 mg, 79 µmol, 46%. ^{1}**H-NMR** (500 MHz, $CDCl_3$): δ = 0.88 (t, *J* = 6.9 Hz, 3H, CH_3), 1.20–1.34 (m, 20H, CH_2), 1.37–1.58 (m, 6H, $SCH_2CH_2CH_2$, $O(CH_2)_2CH_2$, $NCH_2CH_2CH_2$), 1.65 (m_c, 2H, SCH_2CH_2), 1.76–1.84 (m, 2H, OCH_2CH_2), 1.89 (m_c, 2H, NCH_2CH_2), 2.83 (s, 3H, $NCCH_3$), 2.93 (t, *J* = 7.4 Hz, 2H, SCH_2), 3.95–4.03 (m, 5H, OCH_2, NCH_3), 4.24 (t, *J* = 7.6 Hz, 2H, NCH_2), 6.95 (dd, $J_{3,4}$ = 8.2 Hz, $J_{1,3}$ = 2.4 Hz, 1H, 3-H), 7.11 (d, *J* = 2.4 Hz, 1H, 1-H), 7.26–7.31 (m, 1H, 5-H), 7.32–7.38 (m, 2H, 4-H, 6-H), 7.48–7.54 (m, 2H, 8-H, Im), 7.63 (d, *J* = 2.1 Hz, 1H, Im) ppm; ^{13}**C-NMR** (126 MHz, $CDCl_3$): δ = 11.1 (NC*C*H_3), 14.1 (CH_2CH_3), 22.7, 25.6, 26.1, 28.80, 28.82, 29.0, 29.2, 29.4, 29.5, 29.6, 29.65, 29.67, 29.69, 29.8, 31.9 (*C*H_2), 33.7 (S*C*H_2), 36.2 (N*C*H_3), 49.0 (N*C*H_2), 68.1 (O*C*H_2), 110.2 (C-1), 119.9 (C-5), 120.8 (C-3), 121.1 (Im), 121.3 (C-4), 123.0 (Im), 124.3 (C-8), 134.8 (C-6), 134.9 (C-8'), 135.6 (C-1'), 136.8 (C-4'), 137.5 (C-7), 142.2 (C-5'), 143.9 (N*C*CH_2), 160.0 (C-2), 193.6 (*C*=O) ppm; **FT-IR** (ATR): $\tilde{\nu}$ = 3420 (w), 3067 (w), 2918 (vs), 2851 (s), 1714 (s), 1602 (m), 1538 (w), 1487 (m), 1457 (m), 1436 (m), 1293 (m), 1247 (m), 1181 (w), 1138 (w), 1101 (w), 1028 (w), 891 (w), 816 (w), 781 (m), 759 (w), 667 (w), 498 (w) cm^{-1}; **MS** (ESI): *m/z* = 603 $[M]^+$; **HRMS** (ESI): calcd. for $[C_{38}H_{55}N_2O_2S]^+$ 603.3979, found: 603.3958 $[M]^+$; **elemental analysis**: calcd. (%) for $C_{38}H_{55}BrN_2O_2S$: C 66.74, H 8.11, N 4.10, S 4.69, found: C 66.46, H 8.18, N 4.19, S 4.61; **DSC**: Cr_1 53 [21.7 kJ mol^{-1}] Cr_2 59 [10.3 kJ mol^{-1}] Cr_3 65 [18.6 kJ mol^{-1}] SmC 75 [via POM] SmA 263 [1.4 kJ mol^{-1}] I (1. H).

3-(8-{[7-(Dodecylthio)-9-oxo-9*H*-fluoren-2-yl]oxy}octyl)-1,2-dimethyl-1*H*-imidazol-3-ium bromide [ImMe(O8,S12)Br]. According to GP7, from **Br(O8,S12)** (100 mg, 170 µmol), **16b** (25 mg, 255 µmol), abs. DMF (5 mL); yield: 82 mg, 120 µmol, 71%. ^{1}**H-NMR** (400 MHz, $CDCl_3$): δ = 0.87 (t, *J* = 6.7 Hz, 3H, CH_2CH_3), 1.19–1.51 (m, 26H, CH_2), 1.65 (m_c, 2H, SCH_2CH_2), 1.71–1.92 (m, 4H, OCH_2CH_2, NCH_2CH_2), 2.83 (s, 3H, $NCCH_3$), 2.93 (t, *J* = 7.4 Hz, 2H, SCH_2), 3.98 (t, *J* = 6.4 Hz, 2H, OCH_2), 4.03 (s, 3H, NCH_3), 4.21 (t, *J* = 7.5 Hz, 2H, NCH_2), 6.95 (dd, $J_{3,4}$ = 8.2 Hz, $J_{1,3}$ = 2.4 Hz, 1H, 3-H), 7.13 (d, $J_{1,3}$ = 2.4 Hz, 1H, 1-H), 7.29 (d, $J_{5,6}$ = 7.9 Hz, 1H, 5-H), 7.32–7.39 (m, 2H, 4-H, 6-H), 7.47 (d, *J* = 2.1 Hz, 1H, Im), 7.52 (d, $J_{6,8}$ = 1.7 Hz, 1H, 8-H), 7.69 (d, *J* = 2.1 Hz, 1H, Im) ppm; ^{13}**C-NMR** (101 MHz, $CDCl_3$): δ = 11.1 (NC*C*H_3), 14.1 (CH_2CH_3), 22.7, 25.9, 26.3, 28.8, 28.96, 29.01, 29.03, 29.2, 29.3, 29.5, 29.57, 29.62, 29.64, 29.8,

31.9 (CH_2), 33.7 (SCH_2), 36.3 (NCH_3), 49.1 (NCH_2), 68.4 (OCH_2), 110.2 (C-1), 119.9 (C-5), 120.9 (C-3), 121.0 (Im), 121.3 (C-4), 123.1 (Im), 124.3 (C-8), 134.8 (C-6), 135.0 (C-8'), 135.7 (C-1'), 136.7 (C-4'), 137.5 (C-7), 142.3 (C-5'), 143.9 ($NCCH_3$), 160.2 (C-2), 193.6 (*C*=O) ppm; **FT-IR** (ATR): $\tilde{\nu}$ = 3420 (w), 3064 (w), 2917 (vs), 2850 (s), 1714 (s), 1602 (m), 1538 (w), 1487 (m), 1469 (m), 1457 (m), 1434 (m), 1420 (m), 1293 (m), 1248 (s), 1224 (w), 1205 (w), 1180 (w), 1137 (w), 1102 (w), 1090 (w), 1032 (w), 891 (w), 820 (w), 780 (m), 758 (m), 719 (w), 498 (w) cm^{-1}; **MS** (ESI): *m/z* = 603 $[M]^+$; **HRMS** (ESI): calcd. for $[C_{38}H_{55}N_2O_2S]^+$ 603.3979, found: 603.3990 $[M]^+$; **elemental analysis**: calcd. (%) for $C_{38}H_{55}BrN_2O_2S$: C 66.74, H 8.11, N 4.10, S 4.69, found: C 67.00, H 8.32, N 3.94, S 4.53; **DSC**: Cr 51 [40.5 kJ mol^{-1}] SmC 60 [via POM] SmA 240 [1.0 kJ mol^{-1}] I (1. H).

3-(8-{[7-(Tetradecylthio)-9-oxo-9*H*-fluoren-2-yl]oxy}octyl)-1,2-dimethyl-1*H*-imidazol-3-ium bromide [ImMe(O8,S14)Br]. According to GP7, from **Br(O8,S14)** (70 mg, 113 µmol), **16b** (16 mg, 170 µmol), abs. DMF (5 mL); yield: 46 mg, 65 µmol, 57%. **^{1}H-NMR** (500 MHz, $CDCl_3$): δ = 0.88 (t, *J* = 6.9 Hz, 3H, CH_2CH_3), 1.19–1.50 (m, 30H, CH_2), 1.65 (m_c, 2H, SCH_2CH_2), 1.74–1.81 (m, 2H, OCH_2CH_2), 1.81–1.89 (m, 2H, NCH_2CH_2), 2.82 (s, 3H, $NCCH_3$), 2.91–2.96 (m, 2H, SCH_2), 3.98 (t, *J* = 6.5 Hz, 2H, OCH_2), 4.03 (s, 3H, NCH_3), 4.18–4.24 (m, 2H, NCH_2), 6.95 (dd, $J_{3,4}$ = 8.2 Hz, $J_{1,3}$ = 2.5 Hz, 1H, 3-H), 7.13 (d, $J_{1,3}$ = 2.4 Hz, 1H, 1-H), 7.27–7.30 (m, 1H, 5-H), 7.33–7.37 (m, 2H, 4-H, 6-H), 7.47 (d, *J* = 2.1 Hz, 1H, Im), 7.51 (d, $J_{6,8}$ = 1.7 Hz, 1H, 8-H), 7.70 (d, *J* = 2.1 Hz, 1H, Im) ppm; **^{13}C-NMR** (126 MHz, $CDCl_3$): δ = 11.1 ($NCCH_2CH_3$), 14.1 ($CH_2CH_2CH_3$), 22.7, 25.8, 26.3, 28.8, 28.96, 29.00, 29.04, 29.2, 29.4, 29.5, 29.57, 29.64, 29.66, 29.68, 29.8, 31.9 (CH_2), 33.7 (SCH_2), 36.2 (NCH_3), 49.1 (NCH_2), 68.4 (OCH_2), 110.1 (C-1), 119.9 (C-5), 120.9 (C-3), 121.0 (Im), 121.3 (C-4), 123.1 (Im), 124.3 (C-8), 134.8 (C-6), 134.9 (C-8'), 135.6 (C-1'), 136.7 (C-4'), 137.4 (C-7), 142.3 (C-5'), 143.8 ($NCCH_3$), 160.2 (C-2), 193.6 (*C*=O) ppm; **FT-IR** (ATR): $\tilde{\nu}$ = 3418 (w), 3066 (w), 2916 (vs), 2850 (s), 1714 (s), 1600 (m), 1538 (w), 1489 (m), 1469 (m), 1456 (m), 1437 (m), 1392 (w), 1294 (m), 1246 (m), 1211 (w), 1147 (w), 1092 (w), 1031 (w), 998 (w), 830 (m), 780 (m), 757 (m), 718 (w), 499 (m) cm^{-1}; **MS** (ESI): *m/z* = 631 $[M]^+$; **HRMS** (ESI): calcd. for $[C_{40}H_{59}N_2O_2S]^+$ 631.4292, found: 631.4287 $[M]^+$; **elemental analysis**: calcd. (%) for $C_{40}H_{59}BrN_2O_2S$: C 67.49, H 8.35, N 3.94, S 4.50, found: C 66.45, H 8.44, N 3.99, S 4.31; **DSC**: Cr 57 [48.7 kJ mol^{-1}] SmC 67 [via POM] SmA 257 [13.7 kJ mol^{-1}] I (1. H).

3-(4-{[7-(Decylthio)-9-oxo-9*H*-fluoren-2-yl]oxy}butyl)-2-ethyl-1-methyl-1*H*-imidazol-3-ium bromide [ImEt(O4,S10)Br]. According to GP7, from **Br(O4,S10)** (80 mg, 159 µmol), **16c** (26 mg, 239 µmol), abs. DMF (4 mL); yield: 65 mg, 106 µmol, 67%. **^{1}H-NMR** (400 MHz, $CDCl_3$): δ = 0.87 (t, *J* = 6.7 Hz, 3H, $CH_2CH_2CH_3$), 1.19–1.37 (m, 15H, CH_2, $NCCH_2CH$), 1.37–1.46 (m_c, 2H, $SCH_2CH_2CH_2$), 1.65 (m_c, 2H, SCH_2CH_2), 1.90–2.01 (m, 2H, OCH_2CH_2), 2.07–2.18 (m, 2H, NCH_2CH_2), 2.93 (t, *J* = 7.4 Hz, 2H, SCH_2), 3.22 (q, *J* = 7.7 Hz, 2H, $NCCH_2$), 4.04 (s, 3H, NCH_3), 4.07 (t, *J* = 5.9 Hz, 2H, OCH_2), 4.40 (t, *J* = 7.6 Hz, 2H, NCH_2), 6.95 (dd, $J_{3,4}$ = 8.2 Hz, $J_{1,3}$ = 2.4 Hz, 1H, 3-H), 7.07 (d, $J_{1,3}$ = 2.4 Hz, 1H, 1-H), 7.25–7.30 (m, 1H, 5-H), 7.31–7.37 (m, 2H, 4-H, 6-H), 7.49 (d, $J_{6,8}$ = 1.7 Hz, 1H, 8-H), 7.78 (d, *J* = 2.1 Hz, 1H, Im), 7.82 (d, *J* = 2.1 Hz, 1H, Im) ppm; **^{13}C-NMR** (101 MHz, $CDCl_3$): δ = 11.7 ($NCCH_2CH_3$), 14.1 ($CH_2CH_2CH_3$), 17.7

(NCCH_2), 22.7, 26.0, 27.5, 28.8, 29.0, 29.2, 29.3, 29.50, 29.53, 31.9 (CH_2), 33.7 (SCH_2), 35.9 (NCH_3), 48.4 (NCH_2), 67.7 (OCH_2), 110.4 (C-1), 120.0 (C-5), 120.4 (C-3), 121.4 (Im), 121.6 (C-4), 123.4 (Im), 124.2 (C-8), 134.7 (C-6), 134.9 (C-8'), 135.7 (C-1'), 137.0 (C-4'), 137.7 (C-7), 142.1 (C-5'), 147.6 (N$C$$CH_2$), 159.6 (C-2), 193.4 ($C$=O) ppm; **FT-IR** (ATR): $\tilde{\nu}$ = 3405 (w), 3063 (w), 2954 (m), 2922 (s), 2852 (m), 1710 (s), 1599 (m), 1532 (w), 1488 (m), 1455 (vs), 1437 (s), 1397 (w), 1345 (w), 1287 (s), 1246 (s), 1227 (s), 1206 (w), 1182 (w), 1143 (w), 1100 (m), 1063 (w), 1029 (w), 1002 (w), 967 (w), 908 (w), 821 (w), 785 (m), 731 (s), 643 (w), 501 (w) cm^{-1}; **MS** (ESI): *m/z* = 533 $[M]^+$; **HRMS** (ESI): calcd. for $[C_{33}H_{45}N_2O_2S]^+$ 533.3196, found: 533.3208 $[M]^+$; **elemental analysis**: calcd. (%) for $C_{33}H_{45}BrN_2O_2S$: C 64.59, H 7.39, N 4.56, S 5.22, found: C 64.04, H 7.45, N 4.47, S 5.17; **DSC**: Cr 73 [36.8 kJ mol^{-1}] SmA 245 [1.2 kJ mol^{-1}] I (1. H).

3-(4-{[7-(Dodecylthio)-9-oxo-9*H*-fluoren-2-yl]oxy}butyl)-2-ethyl-1-methyl-1*H*-imidazol-3-ium bromide [ImEt(O4,S12)Br]. According to GP7, from **Br(O4,S12)** (105 mg, 197 µmol), **16c** (33 mg, 296 µmol), abs. DMF (5 mL); yield: 84 mg, 131 µmol, 66%. **^{1}H-NMR** (400 MHz, $CDCl_3$): δ = 0.87 (t, *J* = 6.7 Hz, 3H, $CH_2CH_2CH_3$), 1.18–1.48 (m, 21H, CH_2, $NCCH_2CH_3$), 1.65 (m_c, 2H, SCH_2CH_2), 1.90–2.00 (m, 2H, OCH_2CH_2), 2.07–2.19 (m, 2H, NCH_2CH_2), 2.93 (t, *J* = 7.4 Hz, 2H, SCH_2), 3.22 (q, *J* = 7.7 Hz, 2H, $NCCH_2$), 4.03 (s, 3H, NCH_3), 4.07 (t, *J* = 5.8 Hz, 2H, OCH_2), 4.39 (t, *J* = 7.6 Hz, 2H, NCH_2), 6.95 (dd, $J_{3,4}$ = 8.2 Hz, $J_{1,3}$ = 2.4 Hz, 1H, 3-H), 7.08 (d, $J_{1,3}$ = 2.4 Hz, 1H, 1-H), 7.26–7.30 (m, 1H, 5-H), 7.32–7.38 (m, 2H, 4-H, 6-H), 7.50 (d, $J_{6,8}$ = 1.7 Hz, 1H, 8-H), 7.75 (d, *J* = 2.1 Hz, 1H, Im), 7.79 (d, *J* = 2.1 Hz, 1H, Im) ppm; **^{13}C-NMR** (101 MHz, $CDCl_3$): δ = 11.7 ($NCCH_2CH_3$), 14.1 ($CH_2CH_2CH_3$), 17.8 (NCCH_2), 22.7, 26.0, 27.5, 28.8, 29.0, 29.2, 29.3, 29.5, 29.58, 29.63, 29.7, 31.9 (CH_2), 33.7 (SCH_2), 35.9 (NCH_3), 48.5 (NCH_2), 67.6 (OCH_2), 110.4 (C-1), 120.0 (C-5), 120.4 (C-3), 121.4 (Im), 121.6 (C-4), 123.3 (Im), 124.3 (C-8), 134.8 (C-6), 134.9 (C-8'), 135.7 (C-1'), 137.1 (C-4'), 137.7 (C-7), 142.1 (C-5'), 147.7 (N$C$$CH_2$), 159.6 (C-2), 193.4 ($C$=O) ppm; **FT-IR** (ATR): $\tilde{\nu}$ = 3413 (m), 3061 (w), 2955 (m), 2921 (vs), 2852 (m), 1710 (s), 1600 (m), 1532 (w), 1487 (m), 1456 (s), 1437 (m), 1345 (w), 1289 (m), 1248 (m), 1228 (m), 1183 (w), 1142 (w), 1101 (w), 1002 (w), 968 (w), 820 (w), 785 (m), 758 (w), 726 (w), 644 (w), 501 (w) cm^{-1}; **MS** (ESI): *m/z* = 561 $[M]^+$; **HRMS** (ESI): calcd. for $[C_{35}H_{49}N_2O_2S]^+$ 561.3509, found: 561.3517 $[M]^+$; **elemental analysis**: calcd. (%) for $C_{35}H_{49}BrN_2O_2S$: C 65.51, H 7.70, N 4.37, S 5.00, found: C 64.56, H 7.73, N 4.27, S 4.84; **DSC**: Cr 78 [48.4 kJ mol^{-1}] SmA 258 [1.3 kJ mol^{-1}] I (1. H).

3-(6-{[7-(Decylthio)-9-oxo-9*H*-fluoren-2-yl]oxy}hexyl)-2-ethyl-1-methyl-1*H*-imidazol-3-ium bromide [ImEt(O6,S10)Br]. According to GP7, from **Br(O6,S10)** (70 mg, 131 µmol), **16c** (22 mg, 197 µmol), abs. DMF (5 mL); yield: 37 mg, 58 µmol, 44%. **^{1}H-NMR** (700 MHz, $CDCl_3$): δ = 0.87 (t, *J* = 7.0 Hz, 3H, $CH_2CH_2CH_3$), 1.20–1.36 (m, 15H, CH_2, $NCCH_2CH_3$), 1.42 (m_c, 2H, $SCH_2CH_2CH_2$), 1.46–1.52 (m, 2H, $OCH_2CH_2CH_2$), 1.55 (m_c, 2H, $NCH_2CH_2CH_2$), 1.65 (m_c, 2H, SCH_2CH_2), 1.80 (m_c, 2H, OCH_2CH_2), 1.92 (m_c, 2H, NCH_2CH_2), 2.93 (t, *J* = 7.4 Hz, 2H, SCH_2), 3.20 (q, *J* = 7.7 Hz, 2H, $NCCH_2$), 3.99 (t, *J* = 6.5 Hz, 2H, OCH_2), 4.06 (s, 3H, NCH_3), 4.26 (t, *J* = 7.6 Hz, 2H, NCH_2), 6.95 (dd, $J_{3,4}$ = 8.2 Hz, $J_{1,3}$ = 2.4 Hz, 1H, 3-H), 7.11 (d, $J_{1,3}$ = 2.4 Hz, 1H, 1-H), 7.26–7.30 (m, 1H, 5-H), 7.33–7.37 (m, 2H, 4-H, 6-H), 7.50–7.52 (m, 1H, 8-H), 7.67 (d,

J = 2.0 Hz, 1H, Im), 7.83 (d, J = 2.0 Hz, 1H, Im) ppm; **^{13}C-NMR** (176 MHz, $CDCl_3$): δ = 11.7 ($NCCH_2CH_3$), 14.1 ($CH_2CH_2CH_3$), 17.7 ($NCCH_2$), 22.7, 25.6, 26.2, 28.79, 28.83, 29.0, 29.1, 29.3, 29.49, 29.53, 29.6, 30.3, 31.9 (CH_2), 33.7 (SCH_2), 35.9 (NCH_3), 48.7 (NCH_2), 68.1 (OCH_2), 110.2 (C-1), 119.9 (C-5), 120.8 (C-3), 121.3 (C-4, Im), 123.5 (Im), 124.3 (C-8), 134.8 (C-6), 134.9 (C-8'), 135.6 (C-1'), 136.8 (C-4'), 137.5 (C-7), 142.2 (C-5'), 147.5 ($NCCH_2$), 160.0 (C-2), 193.6 (*C*=O) ppm; **FT-IR** (ATR): $\tilde{\nu}$ = 3408 (w), 3062 (w), 2922 (s), 2853 (m), 1711 (s), 1600 (m), 1532 (w), 1487 (m), 1456 (vs), 1437 (m), 1345 (w), 1288 (s), 1247 (s), 1228 (m), 1183 (w), 1142 (w), 1100 (w), 1029 (w), 1000 (w), 968 (w), 819 (w), 785 (m), 759 (w), 727 (w), 644 (w), 501 (w) cm^{-1}; **MS** (ESI): *m/z* = 561 $[M]^+$; **HRMS** (ESI): calcd. for $[C_{35}H_{49}N_2O_2S]^+$ 561.3509, found: 561.3466 $[M]^+$; **elemental analysis**: calcd. (%) for $C_{35}H_{49}BrN_2O_2S$: C 65.51, H 7.70, N 4.37, S 5.00, found: C 63.82, H 7.77, N 4.29, S 4.82; **DSC**: Cr 51 [47.0 kJ mol^{-1}] SmA 219 [1.5 kJ mol^{-1}] I (1. H).

3-(6-{[7-(Dodecylthio)-9-oxo-9*H*-fluoren-2-yl]oxy}hexyl)-2-ethyl-1-methyl-1*H*-imidazol-3-ium bromide [ImEt(O6,S12)Br]. According to GP7, from **Br(O6,S12)** (90 mg, 161 µmol), **16c** (27 mg, 242 µmol), abs. DMF (4 mL); yield: 76 mg, 113 µmol, 70%. **^{1}H-NMR** (500 MHz, $CDCl_3$): δ = 0.87 (t, J = 6.9 Hz, 3H, $CH_2CH_2CH_3$), 1.18–1.36 (m, 19H, CH_2, $NCCH_2CH_3$), 1.38–1.59 (m, 6H, $SCH_2CH_2CH_2$, $OCH_2CH_2CH_2$, $NCH_2CH_2CH_2$), 1.65 (m_c, 2H, SCH_2CH_2), 1.75–1.84 (m, 2H, OCH_2CH_2), 1.86–1.96 (m, 2H, NCH_2CH_2), 2.93 (t, J = 7.4 Hz, 2H, SCH_2), 3.20 (q, J = 7.7 Hz, 2H, $NCCH_2$), 3.98 (t, J = 6.2 Hz, 2H, OCH_2), 4.05 (s, 3H, NCH_3), 4.26 (t, J = 7.6 Hz, 2H, NCH_2), 6.95 (dd, $J_{3,4}$ = 8.2 Hz, $J_{1,3}$ = 2.4 Hz, 1H, 3-H), 7.10 (d, $J_{1,3}$ = 2.4 Hz, 1H, 1-H), 7.27–7.30 (m, 1H, 5-H), 7.32–7.37 (m, 2H, 4-H, 6-H), 7.51 (d, $J_{6,8}$ = 1.7 Hz, 1H, 8-H), 7.67 (d, J = 2.1 Hz, 1H, Im), 7.82 (d, J = 2.1 Hz, 1H, Im) ppm; **^{13}C-NMR** (126 MHz, $CDCl_3$): δ = 11.7 ($NCCH_2CH_3$), 14.1 ($CH_2CH_2CH_3$), 17.7 ($NCCH_2$), 22.7, 25.6, 26.2, 28.80, 28.84, 29.0, 29.2, 29.3, 29.5, 29.57, 29.62, 29.64, 30.3, 31.9 (CH_2), 33.7 (SCH_2), 35.9 (NCH_3), 48.7 (NCH_2), 68.2 (OCH_2), 110.2 (C-1), 119.9 (C-5), 120.8 (C-3), 121.30 (Im), 121.32 (C-4), 123.5 (Im), 124.3 (C-8), 134.8 (C-6), 134.9 (C-8'), 135.6 (C-1'), 136.8 (C-4'), 137.5 (C-7), 142.2 (C-5'), 147.5 ($NCCH_2$), 160.0 (C-2), 193.6 (*C*=O) ppm; **FT-IR** (ATR): $\tilde{\nu}$ = 3399 (m), 2922 (vs), 2851 (m), 1710 (s), 1600 (m), 1532 (w), 1487 (m), 1456 (vs), 1437 (m), 1345 (w), 1287 (s), 1247 (s), 1228 (m), 1183 (w), 1142 (w), 1100 (w), 1028 (w), 999 (w), 968 (w), 819 (w), 785 (m), 759 (m), 725 (m), 643 (w), 501 (w) cm^{-1}; **MS** (ESI): *m/z* = 589 $[M]^+$; **HRMS** (ESI): calcd. for $[C_{37}H_{53}N_2O_2S]^+$ 589.3822, found: 589.3797 $[M]^+$; **elemental analysis**: calcd. (%) for $C_{37}H_{53}BrN_2O_2S$: C 66.35, H 7.98, N 4.18, S 4.79, found: C 65.72, H 7.99, N 3.96, S 4.57; **DSC**: Cr 67 [33.5 kJ mol^{-1}] SmA 235 [1.2 kJ mol^{-1}] I (1. H).

3-(8-{[7-(decylthio)-9-oxo-9*H*-fluoren-2-yl]oxy}octyl)-2-ethyl-1-methyl-1*H*-imidazol-3-ium bromide [ImEt(O8,S10)Br]. According to GP7, from **Br(O8,S10)** (80 mg, 143 µmol), **16c** (24 mg, 215 µmol), abs. DMF (4 mL); yield: 67 mg, 100 µmol, 70%. **^{1}H-NMR** (500 MHz, $CDCl_3$): δ = 0.87 (t, J = 6.9 Hz, 3H, $CH_2CH_2CH_3$), 1.18–1.51 (m, 25H, CH_2, $NCCH_2CH_3$), 1.65 (m_c, 2H, SCH_2CH_2), 1.73–1.82 (m, 2H, OCH_2CH_2), 1.83–1.93 (m, 2H, NCH_2CH_2), 2.93 (t, J = 7.4 Hz, 2H, SCH_2), 3.19 (q, J = 7.7 Hz, 2H, $NCCH_2$), 3.98 (t, J = 6.4 Hz, 2H, OCH_2), 4.06 (s, 3H, NCH_3),

4.18–4.25 (m, 2H, NCH_2), 6.95 (dd, $J_{3,4}$ = 8.2 Hz, $J_{1,3}$ = 2.4 Hz, 1H, 3-H), 7.13 (d, $J_{1,3}$ = 2.4 Hz, 1H, 1-H), 7.27–7.31 (m, 1H, 5-H), 7.32–7.38 (m, 2H, 4-H, 6-H), 7.51 (d, $J_{6,8}$ = 1.8 Hz, 1H, 8-H), 7.58 (d, J = 2.1 Hz, 1H, Im), 7.84 (d, J = 2.1 Hz, 1H, Im) ppm; **^{13}C-NMR** (126 MHz, $CDCl_3$): δ = 11.7 (NCCH$_2$*C*H$_3$), 14.1 (CH$_2$CH$_2$*C*H$_3$), 17.7 (NC*C*H$_2$), 22.7, 25.8, 26.4, 28.8, 29.00, 29.04, 29.1, 29.3, 29.49, 29.52, 30.4, 31.9 (*C*H$_2$), 33.7 (S*C*H$_2$), 36.0 (N*C*H$_3$), 48.8 (N*C*H$_2$), 68.4 (O*C*H$_2$), 110.1 (C-1), 119.9 (C-5), 120.9 (C-3), 121.2 (Im), 121.3 (C-4), 123.6 (Im), 124.3 (C-8), 134.8 (C-6), 134.9 (C-8'), 135.6 (C-1'), 136.7 (C-4'), 137.4 (C-7), 142.3 (C-5'), 147.4 (N*C*CH$_2$), 160.2 (C-2), 193.6 (*C*=O) ppm; **FT-IR** (ATR): $\tilde{\nu}$ = 3403 (m), 2922 (s), 2852 (m), 1711 (s), 1601 (m), 1532 (w), 1487 (m), 1455 (vs), 1436 (m), 1391 (w), 1345 (w), 1285 (s), 1246 (s), 1226 (s), 1183 (w), 1141 (w), 1100 (w), 1030 (w), 1003 (w), 967 (w), 922 (w), 818 (w), 785 (m), 759 (m), 725 (s), 643 (w), 501 (m) cm^{-1}; **MS** (ESI): *m/z* = 589 [M]$^+$; **HRMS** (ESI): calcd. for $[C_{37}H_{53}N_2O_2S]^+$ 589.3822, found: 589.3833 [M]$^+$; **elemental analysis**: calcd. (%) for $C_{37}H_{53}BrN_2O_2S$: C 66.35, H 7.98, N 4.18, S 4.79, found: C 65.15, H 7.99, N 4.11, S 4.57; **DSC**: Cr 84 [2.6 kJ mol^{-1}] SmA 184 [1.5 kJ mol^{-1}] I (1. H).

3-(8-{[7-(Dodecylthio)-9-oxo-9*H*-fluoren-2-yl]oxy}octyl)-2-ethyl-1-methyl-1*H*-imidazol-3-ium bromide [ImEt(O8,S12)Br]. According to GP7, from **Br(O8,S12)** (100 mg, 170 µmol), **16c** (28 mg, 255 µmol), abs. DMF (5 mL); yield: 70 mg, 100 µmol, 59%. **^{1}H-NMR** (500 MHz, $CDCl_3$): δ = 0.87 (t, J = 6.9 Hz, 3H, CH$_2$CH$_2$C*H*$_3$), 1.20–1.50 (m, 29H, C*H*$_2$, NCCH$_2$C*H*$_3$), 1.65 (m$_c$, 2H, SCH$_2$C*H*$_2$), 1.73–1.81 (m, 2H, OCH$_2$C*H*$_2$), 1.87 (m$_c$, 2H, NCH$_2$C*H*$_2$), 2.93 (t, J = 7.4 Hz, 2H, SC*H*$_2$), 3.19 (q, J = 7.7 Hz, 2H, NCC*H*$_2$), 3.98 (t, J = 6.5 Hz, 2H, OC*H*$_2$), 4.06 (s, 3H, NC*H*$_3$), 4.19–4.24 (m, 2H, NC*H*$_2$), 6.95 (dd, $J_{3,4}$ = 8.2 Hz, $J_{1,3}$ = 2.5 Hz, 1H, 3-H), 7.13 (d, $J_{1,3}$ = 2.5 Hz, 1H, 1-H), 7.27–7.31 (m, 1H, 5-H), 7.32–7.38 (m, 2H, 4-H, 6-H), 7.51 (d, $J_{6,8}$ = 1.8 Hz, 1H, 8-H), 7.59 (d, J = 2.1 Hz, 1H, Im), 7.85 (d, J = 2.1 Hz, 1H, Im) ppm; **^{13}C-NMR** (126 MHz, $CDCl_3$): δ = 11.7 (NCCH$_2$*C*H$_3$), 14.1 (CH$_2$CH$_2$*C*H$_3$), 17.7 (NC*C*H$_2$), 22.7, 25.9, 26.4, 28.8, 29.00, 29.01, 29.04, 29.1, 29.3, 29.5, 29.57, 29.62, 29.64, 30.4, 31.9 (*C*H$_2$), 33.7 (S*C*H$_2$), 36.0 (N*C*H$_3$), 48.8 (N*C*H$_2$), 68.4 (O*C*H$_2$), 110.2 (C-1), 119.9 (C-5), 120.9 (C-3), 121.2 (Im), 121.3 (C-4), 123.6 (Im), 124.3 (C-8), 134.8 (C-6), 134.9 (C-8'), 135.6 (C-1'), 136.7 (C-4'), 137.4 (C-7), 142.3 (C-5'), 147.4 (N*C*CH$_2$), 160.2 (C-2), 193.6 (*C*=O) ppm; **FT-IR** (ATR): $\tilde{\nu}$ = 3400 (m), 2921 (vs), 2851 (s), 1710 (s), 1601 (m), 1532 (w), 1487 (m), 1456 (vs), 1436 (m), 1392 (w), 1345 (w), 1286 (m), 1247 (m), 1227 (m), 1183 (w), 1141 (w), 1100 (w), 1029 (w), 1002 (w), 967 (w), 818 (w), 785 (m), 759 (m), 724 (m), 643 (w), 501 (m) cm^{-1}; **MS** (ESI): *m/z* = 617 [M]$^+$; **HRMS** (ESI): calcd. for $[C_{39}H_{57}N_2O_2S]^+$ 617.4135, found: 617.4105 [M]$^+$; **elemental analysis**: calcd. (%) for $C_{39}H_{57}BrN_2O_2S$: C 67.12, H 8.23, N 4.01, found: C 67.08, H 8.06, N 3.85; **DSC**: Cr 110 [62.6 kJ mol^{-1}] SmA 208 [1.5 kJ mol^{-1}] I (1. H).

Differential scanning calorimetry (DSC)

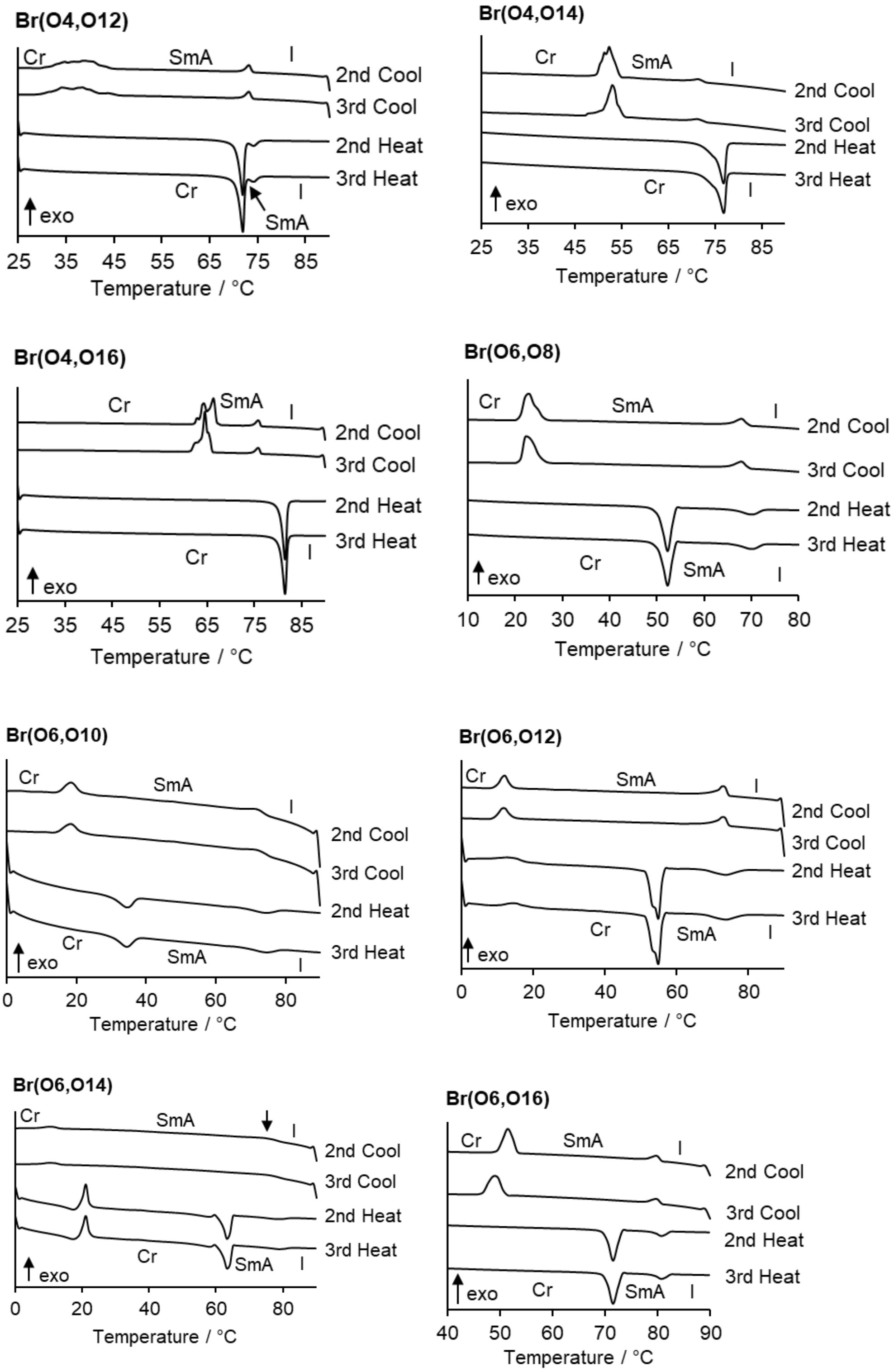

Fig. S1 DSC curves of the bromides **Br(On,Om)** with a short spacer n = 4, 6 (heating/ cooling rate 5 K/min).

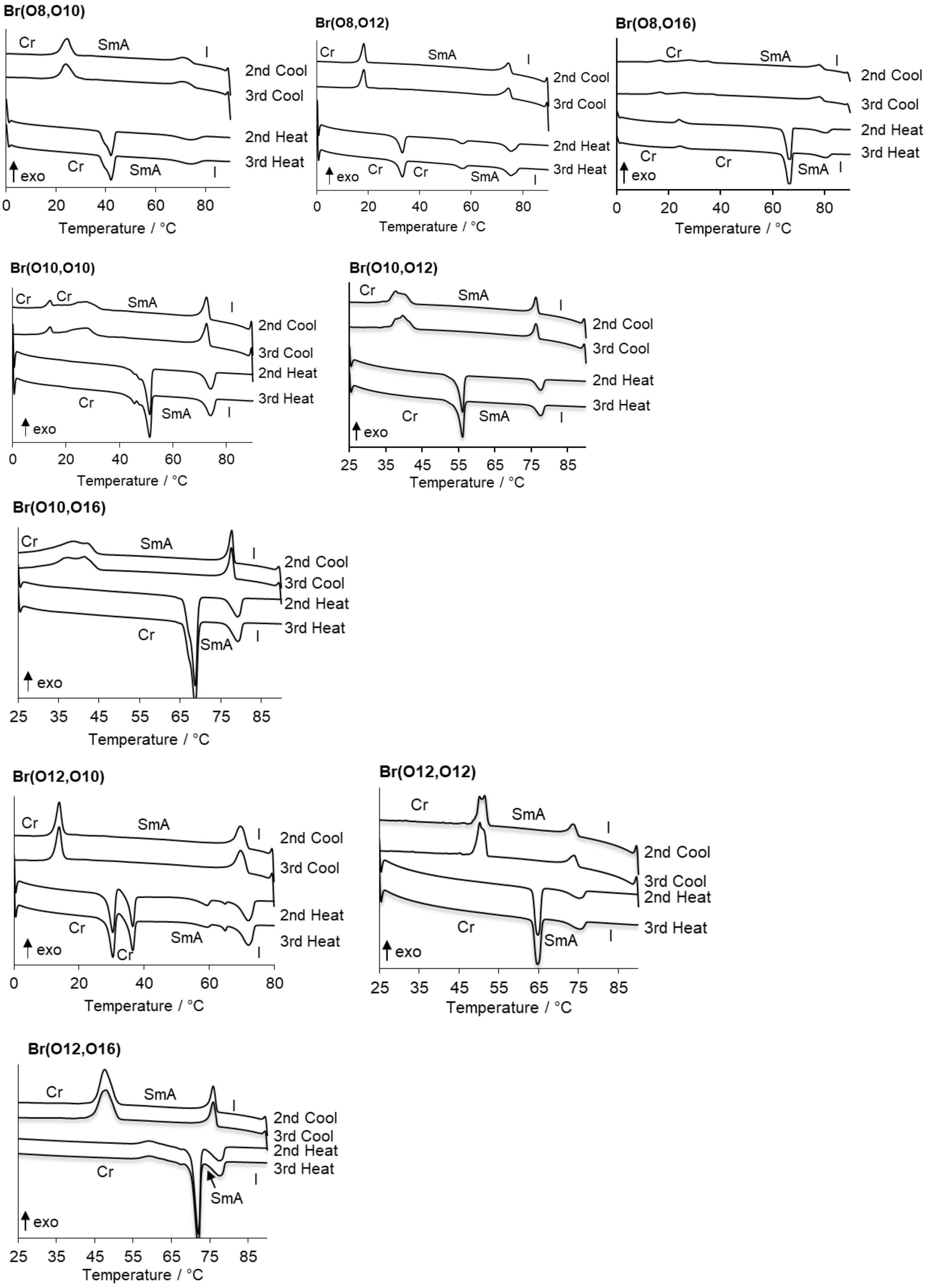

Fig. S2 DSC curves of the bromides **Br(O8,Om)**, **Br(O10,Om)** and **Br(O12,Om)** (heating/cooling rate 5 K/min).

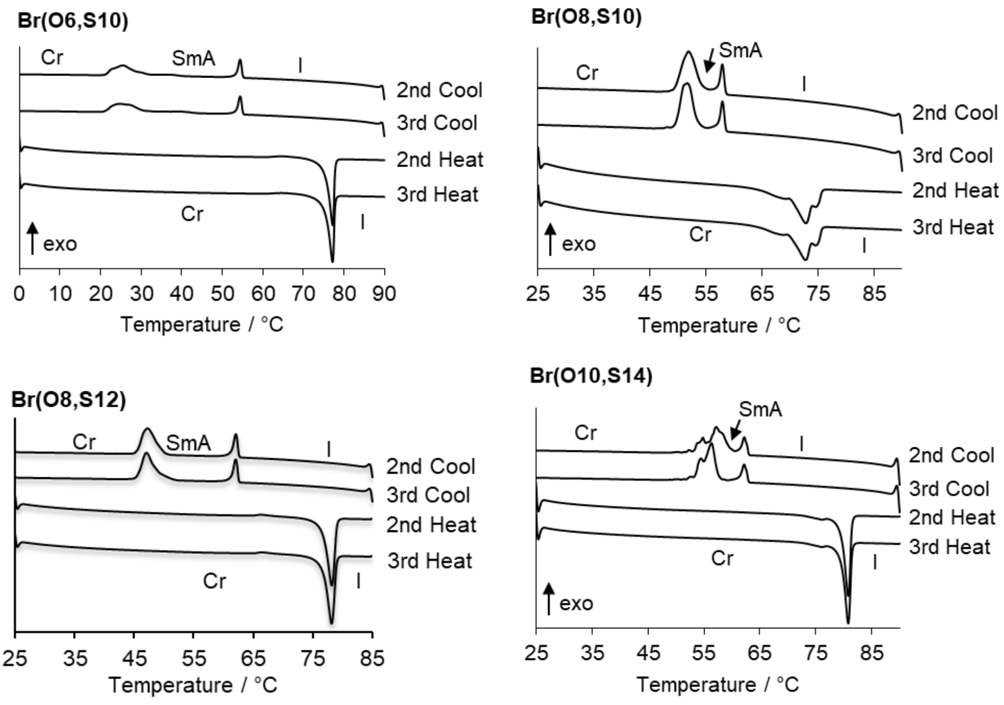

Fig. S3 DSC curves of the bromides **Br(On,Sm)** (heating/cooling rate 5 K/min).

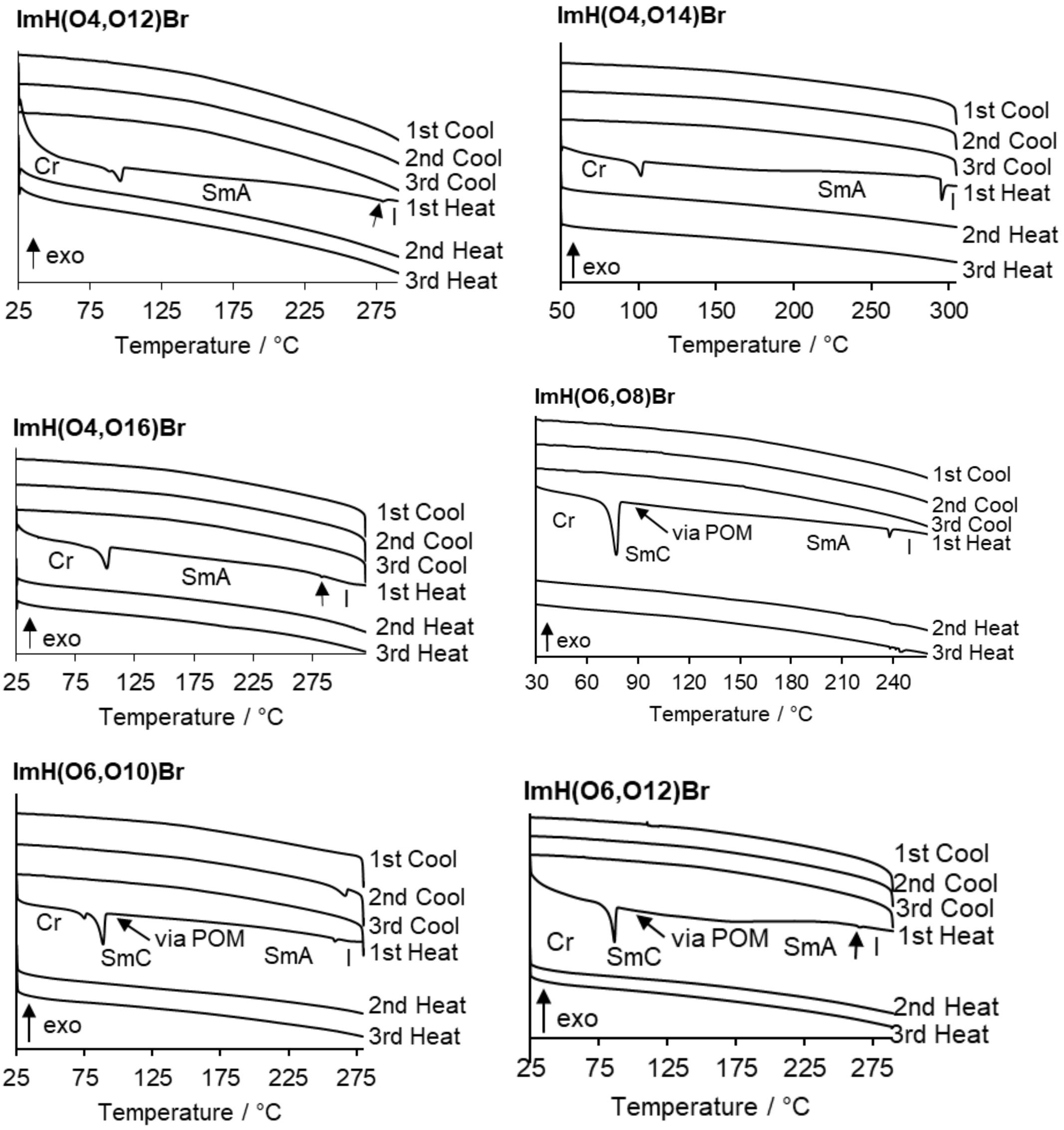

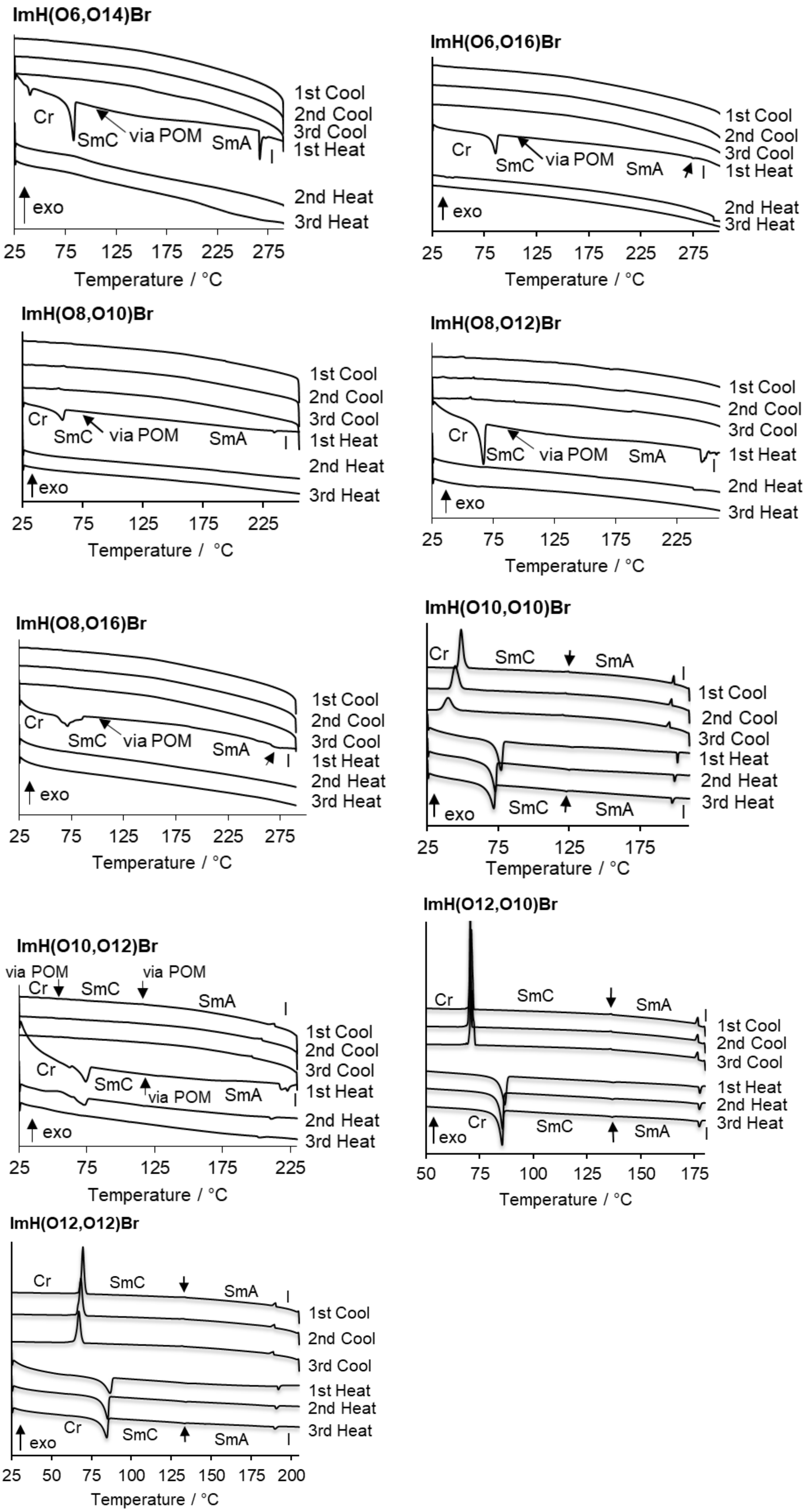

Fig. S4 DSC curves of **ImH(On,Om)Br** (heating/cooling rate 5 K/min).

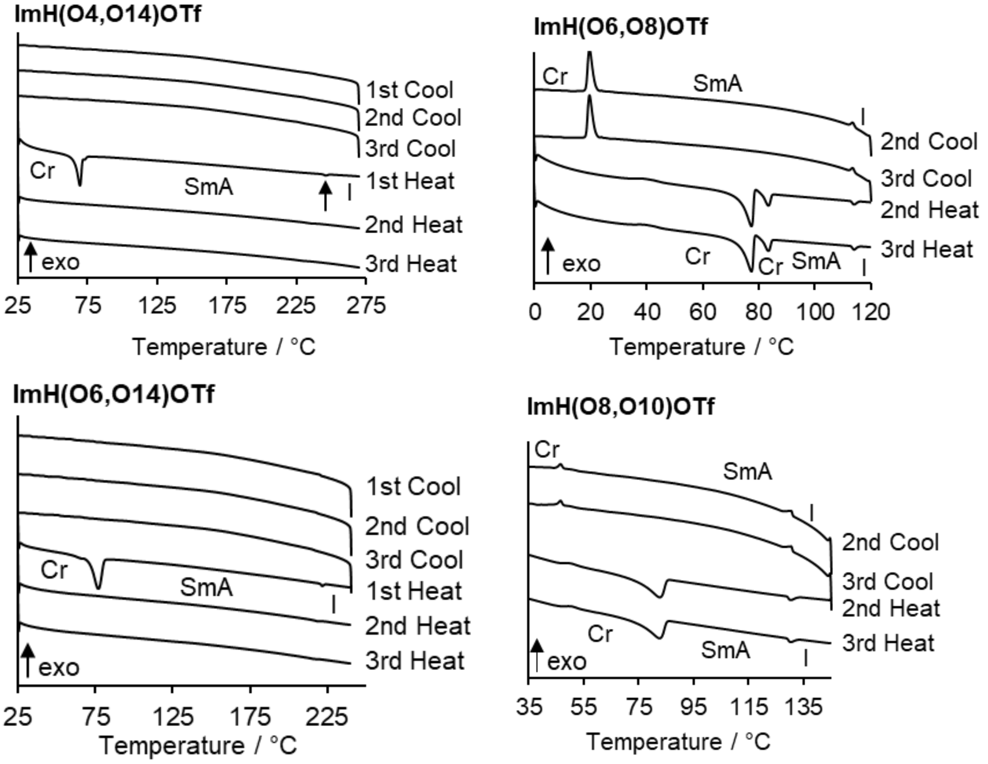

Fig. S5 DSC curves of triflates **ImH(On,Om)OTf** (heating/cooling rate 5 K/min).

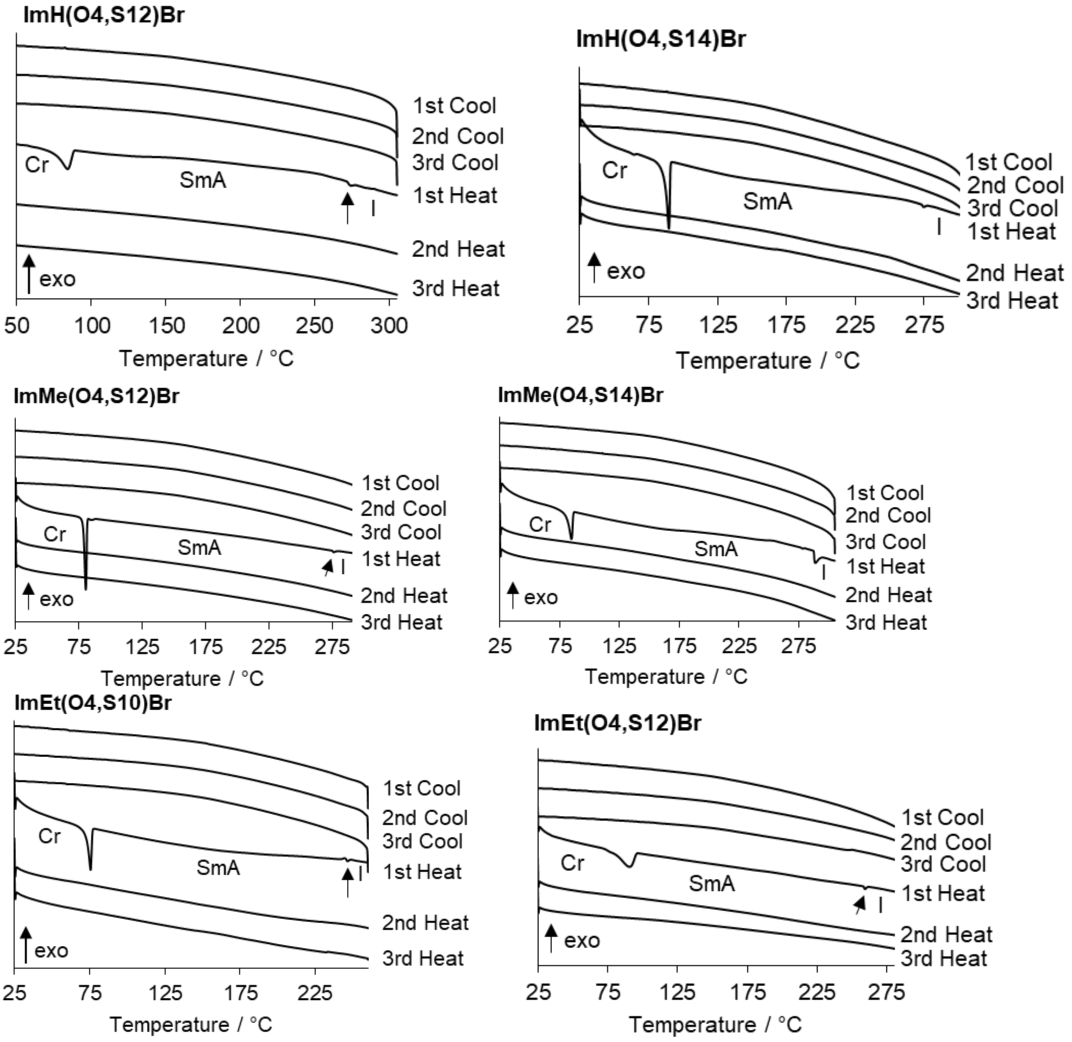

ImH(O6,S12)Br

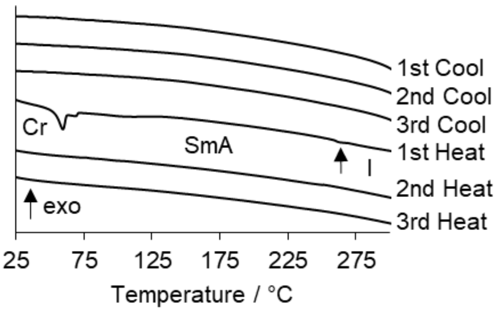

ImH(O6,S14)Br

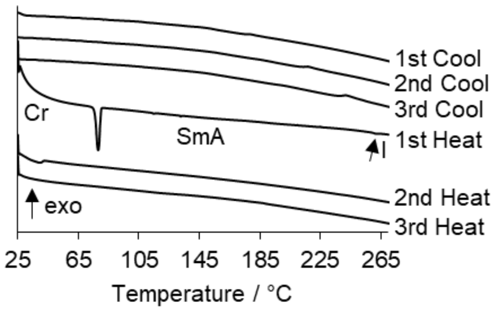

ImMe(O6,S12)Br

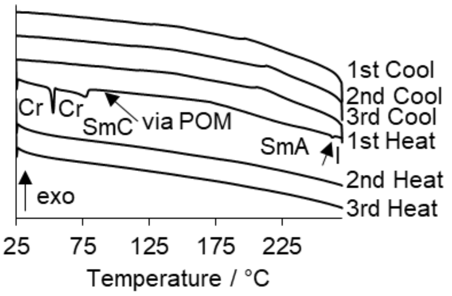

ImMe(O6,S14)Br

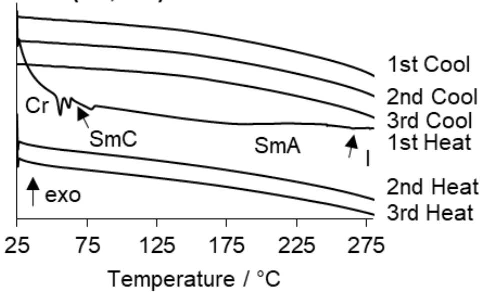

ImEt(O6,S10)Br

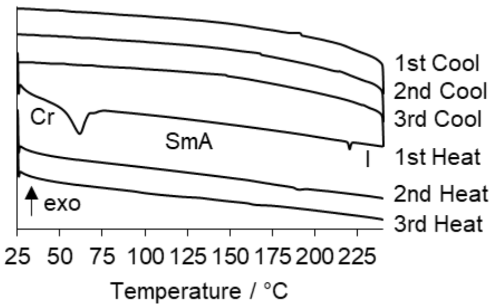

ImEt(O6,S12)Br

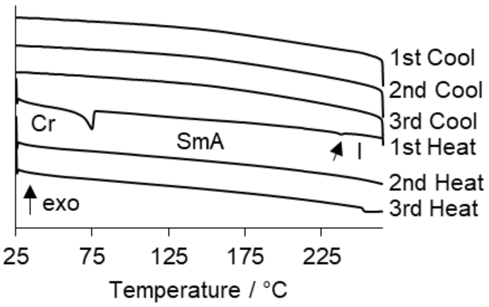

ImH(O8,S12)Br

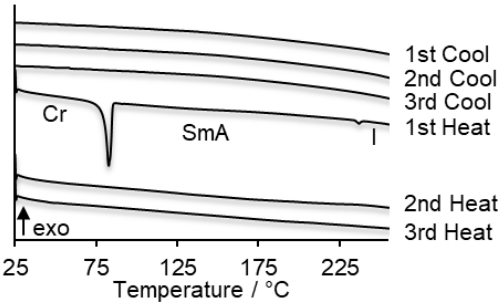

ImH(O8,S14)Br

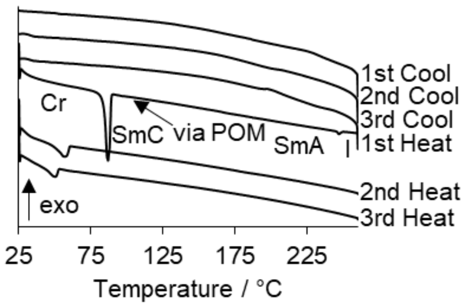

ImMe(O8,S12)Br

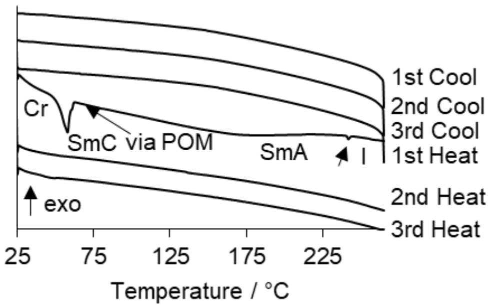

ImMe(O8,S14)Br

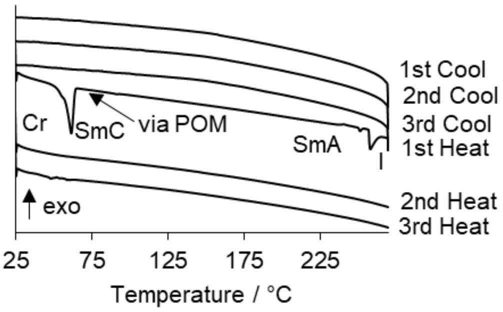

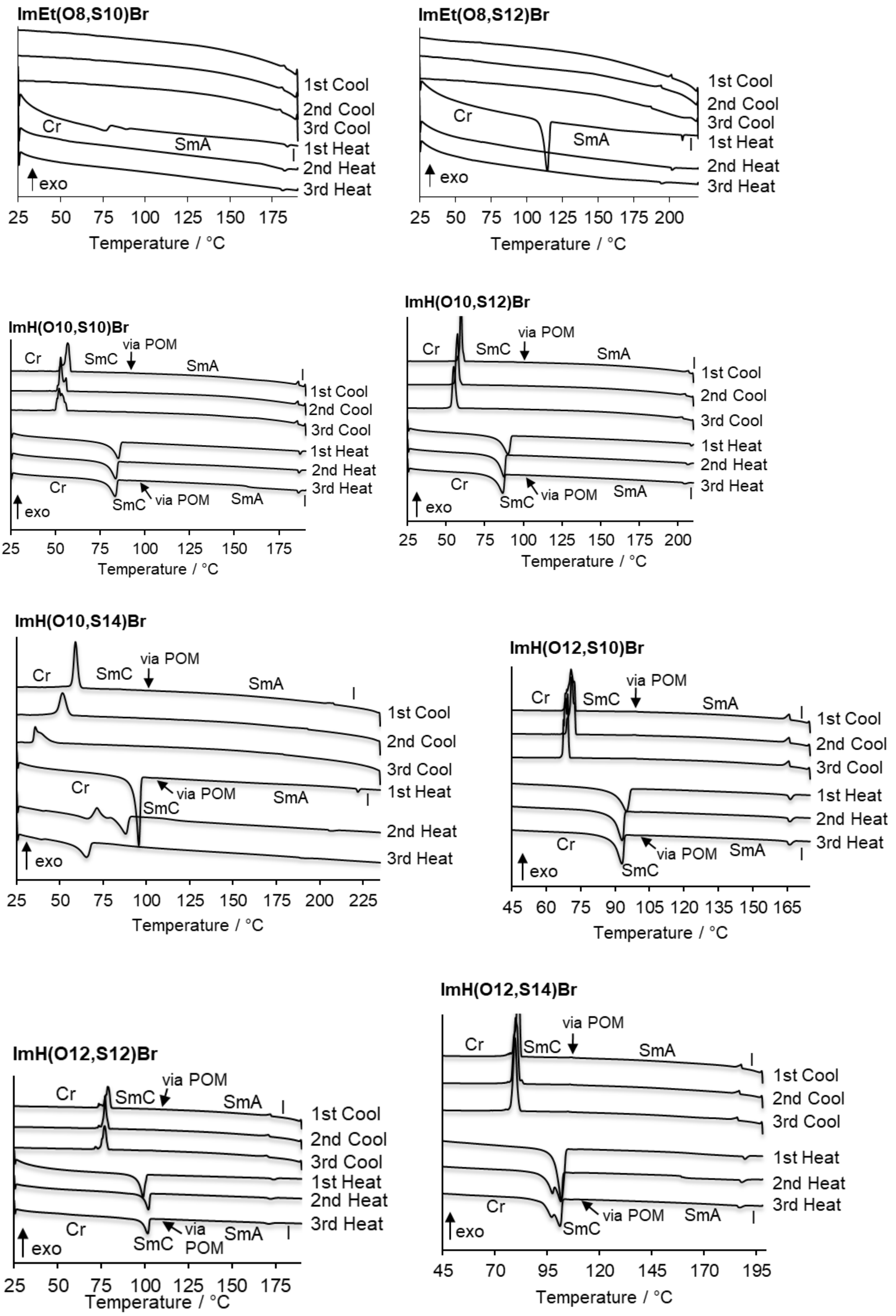

Fig. S6 DSC curves of the imidazolium bromides **ImR(On,Sm)Br** (heating/cooling rate 5 K/min).

Table S1 Phase transition temperatures *T* (°C) and enthalpies *ΔH* (kJ mol^{-1}) of **Br(On,Om)** determined by DSC from 3. heating (H) and cooling (C) (heating/cooling rate 5 K min^{-1}).

Compd	Cr	*T* (Δ*H*)	SmA	*T* (Δ*H*)	I	Cycle
**Br(O4,O12)**	●	71 (26.2)	●	74 (1.6)	●	3. H
	●	43 (-26.6)	●	73 (-4.0)	●	3. C
**Br(O4,O14)**	●		–	74 (45.0)	●	3. H
	●	55 (-37.2)	●	73 (-2.6)	●	3. C
**Br(O4,O16)**	●		–	80 (50.8)	●	3. H
	●	66 (-46.5)	●	77 (-4.7)	●	3. C
**Br(O6,O8)**	●	50 (20.4)	●	70 (4.3)	●	3. H
	●	25 (-16.9)	●	69 (-3.6)	●	3. C
**Br(O6,O10)**	●	30 (8.7)	●	74 (3.9)	●	3. H
	●	21 (-7.0)	●	72 (-3.9)	●	3. C
**Br(O6,O12)**	● [a]	52 (32.3)	●	74 (6.9)	●	3. H
	●	14 (-7.4)	●	73 (-6.4)	●	3. C
**Br(O6,O14)**	● [b]	61 (26.8)	●	79 (5.5)	●	3. H
	●	14 (-3.7)	●	75 (-5.6)	●	3. C
**Br(O6,O16)**	●	70 (42.6)	●	81 (7.7)	●	3. H
	●	51 (-38.3)	●	80 (-7.0)	●	3. C
**Br(O8,O10)**	●	38 (17.2)	●	75 (5.8)	●	3. H
	●	28 (-14.8)	●	72 (-4.9)	●	3. C
**Br(O8,O12)**	● [c]	54 (2.9)	●	75 (8.5)	●	3. H
	●	19 (-9.7)	●	75 (-7.7)	●	3. C
**Br(O8,O16)**	● [d]	64 (43.3)	●	80 (9.5)	●	3. H
	● [e,f]	38 (-1.4)	●	78 (-9.0)	●	3. C
**Br(O10,O10)**	●	49 (23.9)	●	74 (9.9)	●	3. H
	●	36 (-17.8)	●	74 (-9.7)	●	3. C
**Br(O10,O12)**	●	54 (32.4)	●	76 (11.6)	●	3. H
	●	43 (-27.2)	●	76 (-11.5)	●	3. C
**Br(O12,O10)**	● [g]	35 (9.0)	●	69 (9.9)	●	3. H
	●	15 (-10.3)	●	69 (-9.5)	●	3. C
**Br(O12,O12)**	●	63 (35.6)	●	75 (13.4)	●	3. H
	●	52 (-33.7)	●	75 (-11.1)	●	3. C

Crystalline (Cr), smectic A (SmA), isotropic (I), phase observed (●), not observed (–). [a] Cr–Cr transition at 10 °C (–3.4 kJ mol^{-1}). [b] Cr–Cr transition at 19 °C (–19.4 kJ mol^{-1}). [c] Cr–Cr transition at 31 °C (11.9 kJ mol^{-1}). [d] Cr–Cr transition at 22 °C (–3.8 kJ mol^{-1}). [e] Cr–Cr transition at 30 °C (–4.0 kJ mol^{-1}). [f] Cr–Cr transition at 19 °C (–3.5 kJ mol^{-1}). [g] Cr–Cr transition at 28 °C (13.9 kJ mol^{-1}).

Table S2 Phase transition temperatures *T* (°C) and enthalpies *ΔH* (kJ mol^{-1}) of **Br(On,Sm)** determined by DSC from 3. heating (H) and cooling (C) (heating/cooling rate 5 K min^{-1}).

Compd	Cr	*T* (Δ*H*)	SmA	*T* (Δ*H*)	I	Cycle
**Br(O4,S10)**	●		–	80 (37.5)	●	3. H
	●		–	50 (-34.3)	●	3. C
**Br(O4,S12)**	●		–	83 (35.7)	●	3. H
	●		–	68 (-37.2)	●	3. C
**Br(O4,S14)**	●		–	89 (44.6)	●	3. H
	●		–	74 (-44.5)	●	3. C
**Br(O6,S10)**	●		–	75 (43.8)	●	3. H
	●	30 (-17.4)	●	55 (-7.3)	●	3. C
**Br(O6,S12)**	●		–	79 (47.5)	●	3. H
	●		–	63 (-39.6)	●	3. C
**Br(O6,S14)**	●		–	80 (49.6)	●	3. H
	●		–	65 (-49.4)	●	3. C
**Br(O8,S10)**	●		–	69 (40.9)	●	3. H
	●	54 (-29.6)	●	59 (-7.6)	●	3. C
**Br(O8,S12)**	● [a]		–	77 (56.1)	●	3. H
	●	49 (-34.6)	●	63 (-10.8)	●	3. C
**Br(O8,S14)**	●		–	79 (59.9)	●	3. H
	●		–	61 (-52.1)	●	3. C
**Br(O10,S10)**	●		–	74 (62.8)	●	3. H
	●		–	57 (-59.9)	●	3. C
**Br(O10,S12)**	●		–	78 (65.2)	●	3. H
	●		–	64 (-65.5)	●	3. C
**Br(O10,S14)**	●		–	79 (119.7)	●	3. H
	●	58 (-55.4)	●	63 (-12.7)	●	3. C
**Br(O12,S10)**	●		–	79 (57.9)	●	3. H
	●		–	59 (-58.1)	●	3. C
**Br(O12,S12)**	●		–	78 (67.1)	●	3. H
	●		–	65 (-73.7)	●	3. C
**Br(O12,S14)**	●		–	82 (81.9)	●	3. H
	●		–	76 (-75.9)	●	3. C

Crystalline (Cr), smectic A (SmA), isotropic (I), phase observed (●), not observed (–).

[a] Additional Cr–Cr transition at 65°C (–1.5 kJ mol^{-1}).

Table S3 Phase transition temperatures T (°C) and enthalpies ΔH (kJ mol^{-1}) of **Im(On,Om)Br** and **Im(On,Om)OTf** determined by DSC from heating (H) and cooling (C) (heating/cooling rate 5 K min^{-1}).

Compd	Cr	T (ΔH)	SmC	T (ΔH)	SmA	T (ΔH)	I	Cycle
**Im(O4,O12)Br**	●	90 (26.0)	–		●	277 (2.4)	●	1. H
	–		–		–		●	1. C
**Im(O4,O14)Br**	●	97 (33.0)	–		●	294 (18.1)	●	1. H
	–		–		–		●	1. C
**Im(O4,O16)Br**	●	94 (43.7)	–		●	281 (0.7)	●	1. H
	–		–		–		●	1. C
**Im(O6,O8)Br**	●	72 (33.9)	●	88 [a]	●	237 (1.7)	●	1. H
	–		–		–		●	1. C
**Im(O6,O10)Br**	● [b]	84 (28.9)	●	94 [a]	●	258 (1.3)	●	1. H
	–		–		–		●	1. C
**Im(O6,O12)Br**	●	83 (47.0)	●	105 [a]	●	263 (1.3)	●	1. H
	–		–		–		●	1. C
**Im(O6,O14)Br**	● [c]	79 (45.0)	●	104 [a]	●	266 (17.4)	●	1. H
	–		–		–		●	1. C
**Im(O6,O16)Br**	●	81 (39.0)	●	113 [a]	●	273 (0.7)	●	1. H
	–		–		–		●	1. C
**Im(O8,O10)Br**	●	51 (24.5)	●	82 [a]	●	233 (1.3)	●	1. H
	–		–		–		●	1. C
**Im(O8,O12)Br**	●	62 (29.5)	●	83 [a]	●	244 (7.9)	●	1. H
	–		–		–		●	1. C
**Im(O8,O16)Br**	●	61 (53.5)	●	90 [a]	●	266 (6.5)	●	1. H
	–		–		–		●	1. C
**Im(O10,O10)Br**	●	73 (33.6)	●	124 (0.4)	●	201 (2.3)	●	1. H
	●	52 (-31.8)	●	124 (-0.3)	●	199 (-2.1)	●	1. C
**Im(O10,O12)Br**	●	68 (22.4)	●	121 [a]	●	220 (8.5)	●	1. H
	–	50 [a]	●	121 [a]	●	214 (-1.3)	●	1. C
**Im(O12,O10)Br**	●	83 (39.2)	●	136 (0.5)	●	176 (2.8)	●	3. H
	●	73 (-40.2)	●	136 (-0.5)	●	176 (-2.9)	●	3. C
**Im(O12,O12)Br**	●	80 (37.9)	●	133 (0.6)	●	189 (1.9)	●	3. H
	●	69 (-36.4)	●	133 (-0.5)	●	189 (-2.1)	●	3. C
**Im(O4,O14)OTf**	●	65 (41.8)	–		●	243 (1.0)	●	1. H
	–		–		–		●	1. C
**Im(O6,O8)OTf**	● [d]	81 (8.0)	–		●	114 (1.4)	●	3. H
	●	22 (-32.8)	–		●	113 (-1.5)	●	3. C
**Im(O6,O14)OTf**	●	71 (40.8)	–		●	220 (1.0)	●	1. H
	–		–		–		●	1. C
**Im(O8,O10)OTf**	● [e]	74 (21.1)	–		●	130 (1.3)	●	3. H
	●	47 (-1.4)	–		●	130 (-1.6)	●	3. C

Crystalline (Cr), smectic C (SmC), smectic A (SmA), isotropic (I), phase observed (●), not observed (–). [a] Phase transition determined by POM. [b] Cr–Cr transition at 71°C (4.1 kJ mol^{-1}). [c] Cr–Cr transition at 38°C (6.2 kJ mol^{-1}). [d] Cr–Cr transition at 35°C (–2.8 kJ mol^{-1}) and 73°C (38.8 kJ mol^{-1}). [e] Cr–Cr transition at 13°C (–3.1 kJ mol^{-1}) and 47°C (–1.0 kJ mol^{-1}).

Table S4 Phase transition temperatures T (°C) and enthalpies ΔH (kJ mol^{-1}) of **Im(On,Sm)Br** determined by DSC from heating (H) and cooling (C) (heating/cooling rate 5 K min^{-1}).

Compd	Cr	T (ΔH)	SmC	T (ΔH)	SmA	T (ΔH)	I	Cycle
**Im(O4,S12)Br**	●	74 (25.4)	–		●	272 (1.2)	●	1. H
	–		–		–		●	1. C
**Im(O4,S14)Br**	●	86 (36.1)	–		●	273 (1.0)	●	1. H
	–		–		–		●	1. C
**Im(O6,S12)Br**	●	53 (37.9)	–		●	259 (1.4)	●	1. H
	–		–		–		●	1. C
**Im(O6,S14)Br**	●	75 (50.6)	–		●	259 (1.0)	●	1. H
	–		–		–		●	1. C
**Im(O8,S12)Br**	●	80 (55.1)	–		●	234 (1.5)	●	1. H
	–		–		–		●	1. C
**Im(O8,S14)Br**	●	83 (58.6)	●	98 [a]	●	240 (1.0)	●	1. H
	–		–		–		●	1. C
**Im(O10,S10)Br**	●	78 (45.9)	●	95 [a]	●	186 (2.1)	●	3. H
	●	54 (-44.5)	●	93 [a]	●	186 (-2.1)	●	3. C
**Im(O10,S12)Br**	●	83 (58.3)	●	100 [a]	●	208 (1.5)	●	1. H
	●	77 (-53.5)	●	99 [a]	●	207 (-1.8)	●	1. C
**Im(O10,S14)Br**	●	93 (67.9)	●	105 [a]	●	221 (1.5)	●	1. H
	●	61 (-35.9)	●	100 [a]	●	209 (-1.2)	●	1. C
**Im(O12,S10)Br**	●	89 (54.6)	●	102 [a]	●	165 (3.5)	●	3. H
	●	70 (-56.1)	●	101 [a]	●	165 (-3.9)	●	3. C
**Im(O12,S12)Br**	●	98 (45.1)	●	110 [a]	●	172 (3.3)	●	3. H
	●	79 (-48.7)	●	109 [a]	●	171 (-3.7)	●	3. C
**Im(O12,S14)Br**	●	98 (74.0)	●	111 [a]	●	188 (1.8)	●	1. H
	●	82 (-59.1)	●	108 [a]	●	188 (-1.9)	●	1. C

Crystalline (Cr), smectic C (SmC), smectic A (SmA), isotropic (I), phase observed (●), not observed (–). [a] Phase transition determined by POM.

Table S5 Phase transition temperatures *T* (°C) and enthalpies *ΔH* (kJ mol^{-1}) of **ImR(On,Sm)Br** determined by DSC from heating (H) and cooling (C) (heating/cooling rate 5 K min^{-1}).

Compd	Cr	*T* (Δ*H*)	SmC	*T* (Δ*H*)	SmA	*T* (Δ*H*)	I	Cycle
**ImMe(O4,S12)Br**	●	78 (48.4)	–		●	295 (20.3)	●	1. H
	–		–		–		●	1. C
**ImMe(O4,S14)Br**	●	81 (60.3)	–		●	287 (18.3)	●	1. H
	–		–		–		●	1. C
**ImMe(O6,S12)Br**	● [b]	78 (23.3)	●	85 [a]	●	288 (9.8)	●	1. H
	–		–		–		●	1. C
**ImMe(O6,S14)Br**	● [c]	65 (18.6)	●	75 [a]	●	263 (1.4)	●	1. H
	–		–		–		●	1. C
**ImMe(O8,S12)Br**	●	51 (40.5)	●	60 [a]	●	240 (1.0)	●	1. H
	–		–		–		●	1. C
**ImMe(O8,S14)Br**	●	57 (48.7)	●	67 [a]	●	257 (13.7)	●	1. H
	–		–		–		●	1. C
**ImEt(O4,S10)Br**	●	73 (36.8)	–		●	245 (1.2)	●	1. H
	–		–		–		●	1. C
**ImEt(O4,S12)Br**	●	78 (48.4)	–		●	258 (1.3)	●	1. H
	–		–		–		●	1. C
**ImEt(O6,S10)Br**	●	51 (47.0)	–		●	219 (1.5)	●	1. H
	–		–		●	192 (-2.0)	●	1. C
**ImEt(O6,S12)Br**	●	67 (33.5)	–		●	235 (1.2)	●	1. H
	●	40 [a]	●	55 [a]	●	208 [a]	●	1. C
**ImEt(O8,S10)Br**	●	84 (2.6)	–		●	184 (1.5)	●	1. H
	●	36 [a]	●	52 [a]	●	183 (-1.1)	●	1. C
**ImEt(O8,S12)Br**	●	110 (62.6)	–		●	208 (1.5)	●	1. H
	●	55 [a]	●	71 [a]	●	202 (-1.4)	●	1. C

Crystalline (Cr), smectic C (SmC), smectic A (SmA), isotropic (I), phase observed (●), not observed (–). [a] Phase transitionn determined by POM. [b] Cr–Cr transition at 51°C (4.8 kJ mol^{-1}). [c] Cr–Cr transition at 53°C (21.7 kJ mol^{-1}) and 59°C (10.3 kJ mol^{-1}).

Polarizing optical microscopy (POM)

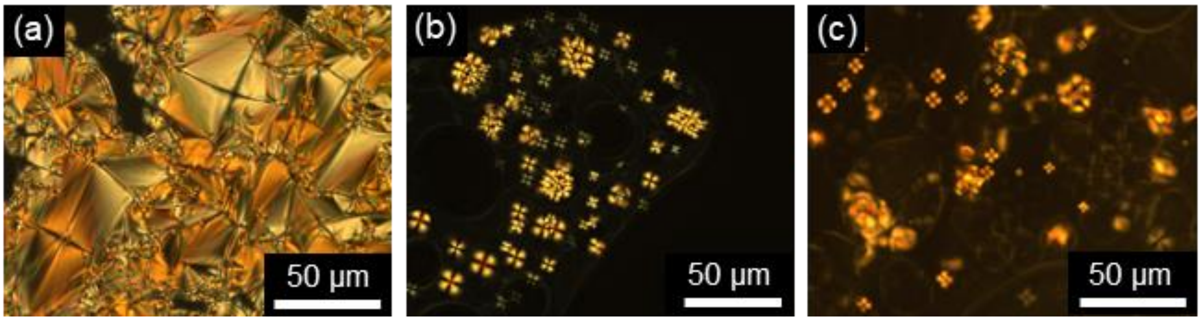

Fig. S7 Textures of bromides **Br(O4,Om)** as seen between crossed polarizers upon cooling from the isotropic liquid (cooling rate 5 K min^{-1}, magnification 200x). (a) **Br(O4,O12)** at 65°C, (b) **Br(O4,O14)** at 73°C, (c) **Br(O4,O16)** at 76°C.

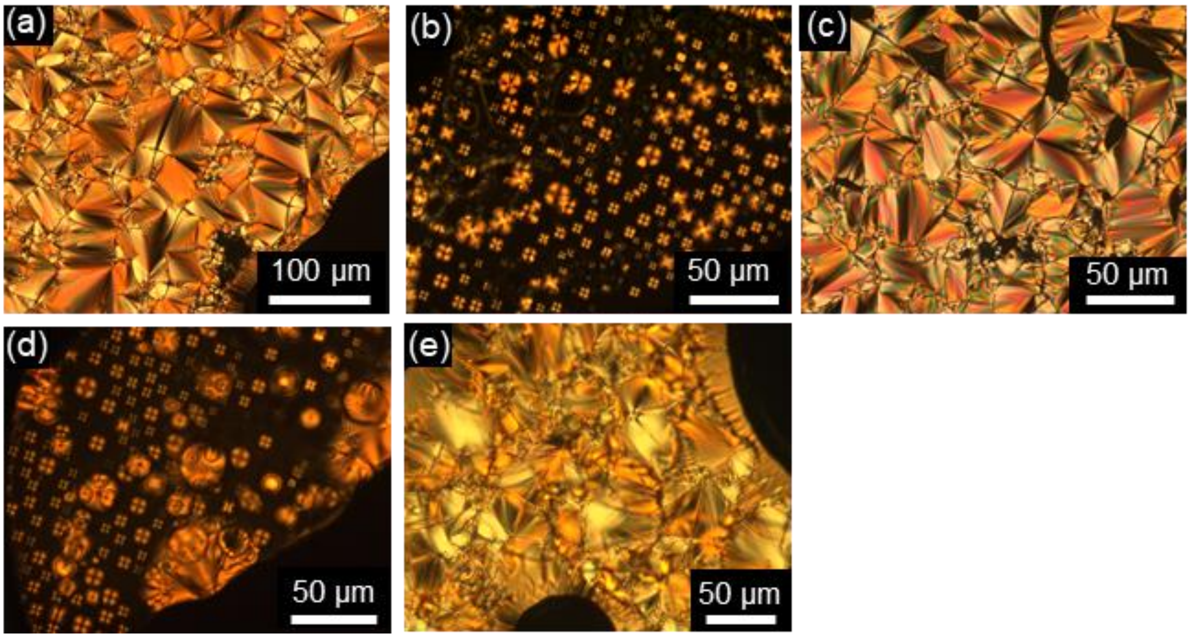

Fig. S8 Textures of bromides **Br(O6,Om)** as seen between crossed polarizers upon cooling from the isotropic liquid (cooling rate 5 K min^{-1}; (a), (e) magnification 100x; (b), (c), (d) magnification 200x). (a) **Br(O6,O8)** at 56°C, (b) **Br(O6,O10)** at 65°C, (c) **Br(O6,O12)** at 71°C, (d) **Br(O6,O14)** at 77°C, (e) **Br(O6,O16)** at 79°C.

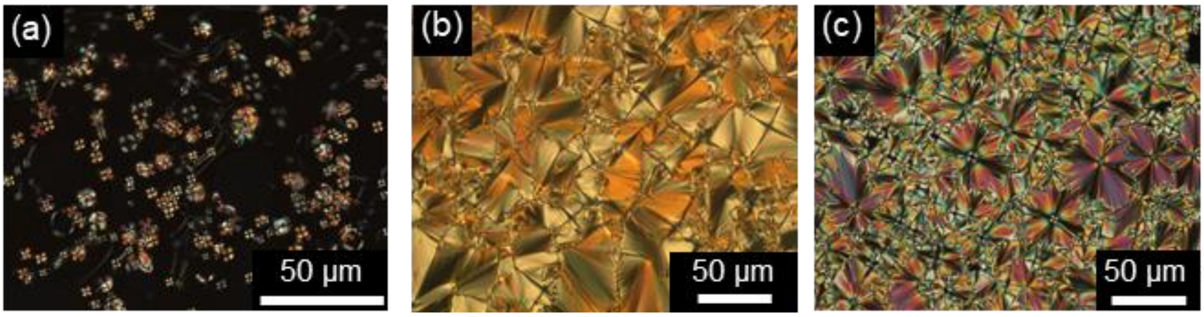

Fig. S9 Textures of bromides **Br(O8,Om)** as seen between crossed polarizers upon cooling from the isotropic liquid (cooling rate 5 K min^{-1}; magnification 200x). (a) **Br(O8,O10)** at 55°C, (b) **Br(O8,O12)** at 72°C, (c) **Br(O8,O16)** at 77°C.

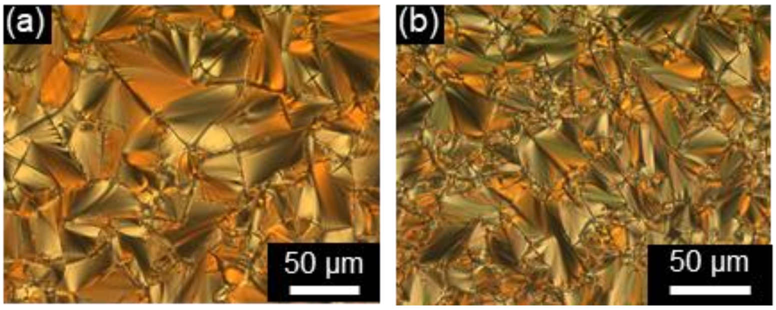

Fig. S10 Textures of bromides **Br(O10,Om)** as seen between crossed polarizers upon cooling from the isotropic liquid (cooling rate 5 K min^{-1}; magnification 200x). (a) **Br(O10,O10)** at 71°C, (b) **Br(O10,O12)** at 70°C.

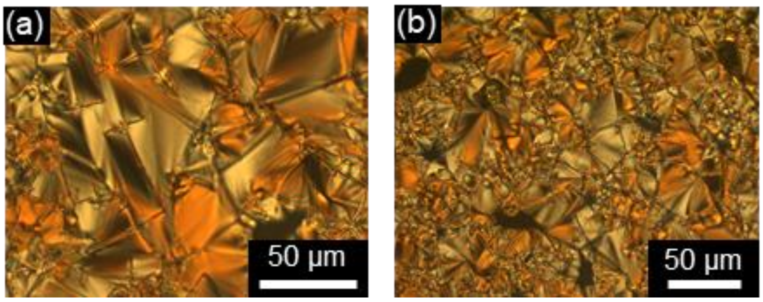

Fig. S11 Textures of bromides **Br(O12,Om)** as seen between crossed polarizers upon cooling from the isotropic liquid (cooling rate 5 K min^{-1}; magnification 200x). (a) **Br(O12,O10)** at 66°C, (b) **Br(O12,O12)** at 72°C.

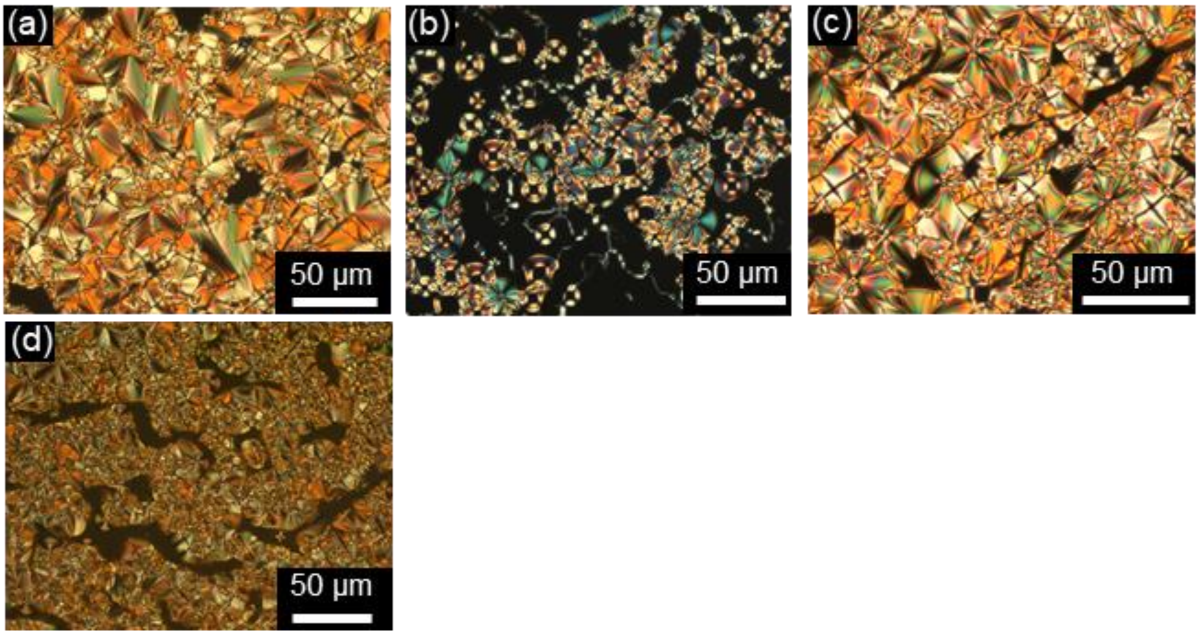

Fig. S12 Textures of bromides **Br(On,Sm)** as seen between crossed polarizers upon cooling from the isotropic liquid (cooling rate 5 K min^{-1}; magnification 200x). (a) **Br(O6,S10)** at 53°C, (b) **Br(O8,S10)** at 56°C, (c) **Br(O8,S12)** at 61°C, (d) **Br(O10,S14)** at 63°C.

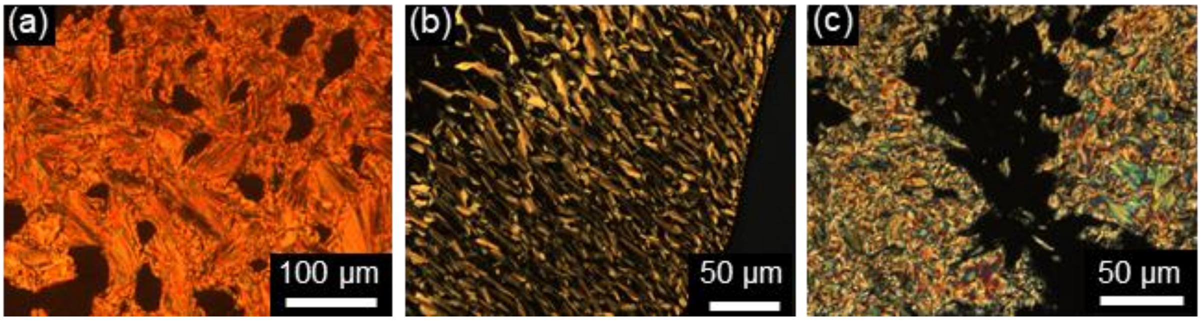

Fig. S13 Textures of imidazolium bromides **Im(O4,Om)Br** as seen between crossed polarizers upon cooling from the isotropic liquid (cooling rate 5 K min^{-1}; (a) magnification 100x, (b), (c) magnification 200x). (a) **Im(O4,O12)Br** at 149°C, (b) **Im(O4,O14)Br** at 119°C, (c) **Im(O4,O16)Br** at 140°C.

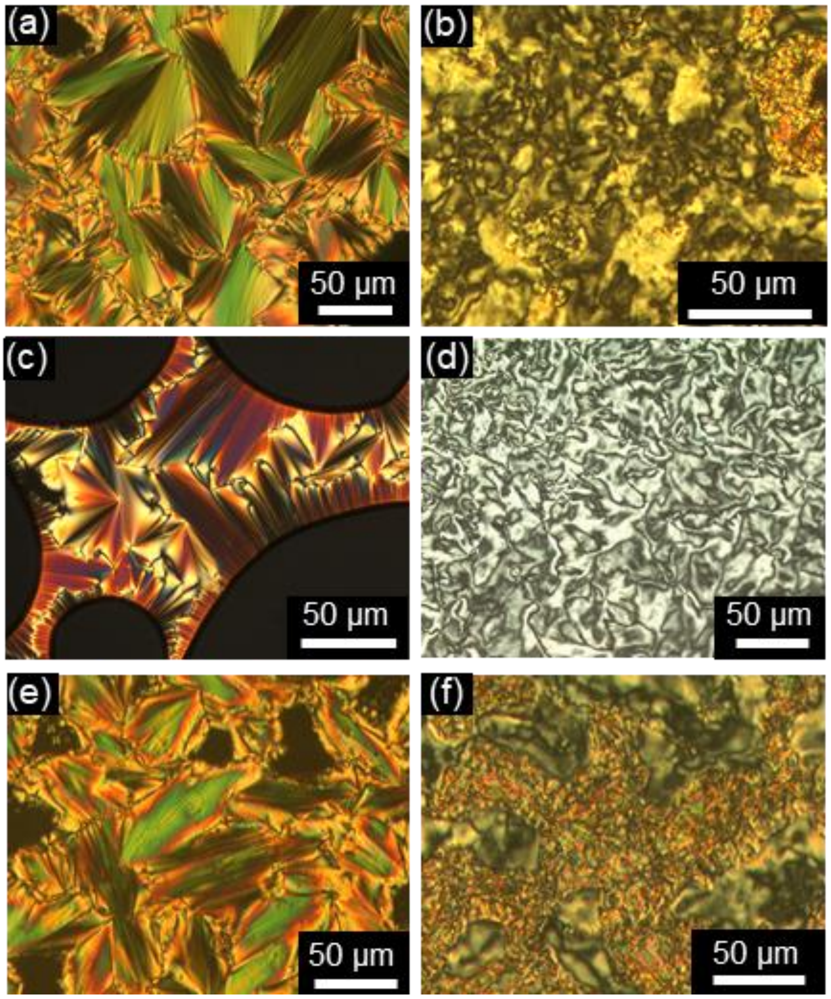

Fig. S14 Textures of imidazolium bromides **Im(O8,Om)Br** as seen between crossed polarizers upon cooling from the isotropic liquid (cooling rate 5 K min^{-1}; (a - d) magnification 200x, (e, f) magnification 100x). (a, b) **Im(O8,O10)Br** at 199°C and at 80°C, (c, d) **Im(O8,O12)Br** at 235°C and at 83°C, (e, f) **Im(O8,O16)Br** at 188°C and at 80°C.

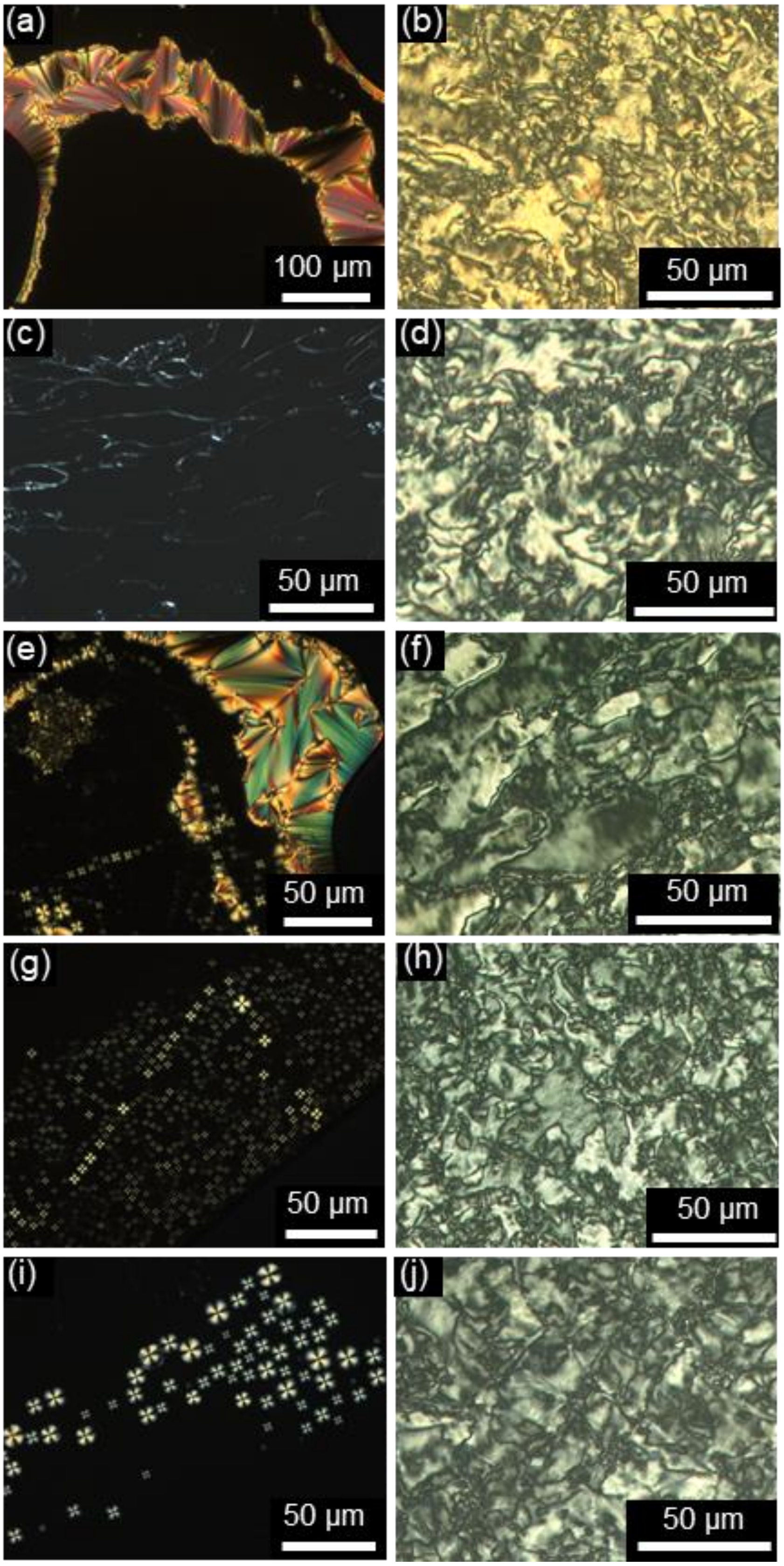

Fig. S15 Textures of imidazolium bromides **Im(O6,Om)Br** as seen between crossed polarizers upon cooling from the isotropic liquid (cooling rate 5 K min^{-1}; (a) magnification 100x, (b - j) magnification 200x). (a, b) **Im(O6,O8)Br** at 210°C and at 74°C, (c, d) **Im(O6,O10)Br** at 104°C and at 71°C, (e, f) **Im(O6,O12)Br** at 183°C and at 92°C, (g, h) **Im(O6,O14)Br** at 217°C and at 92°C, (i, j) **Im(O6,O16)Br** at 134°C and at 90°C.

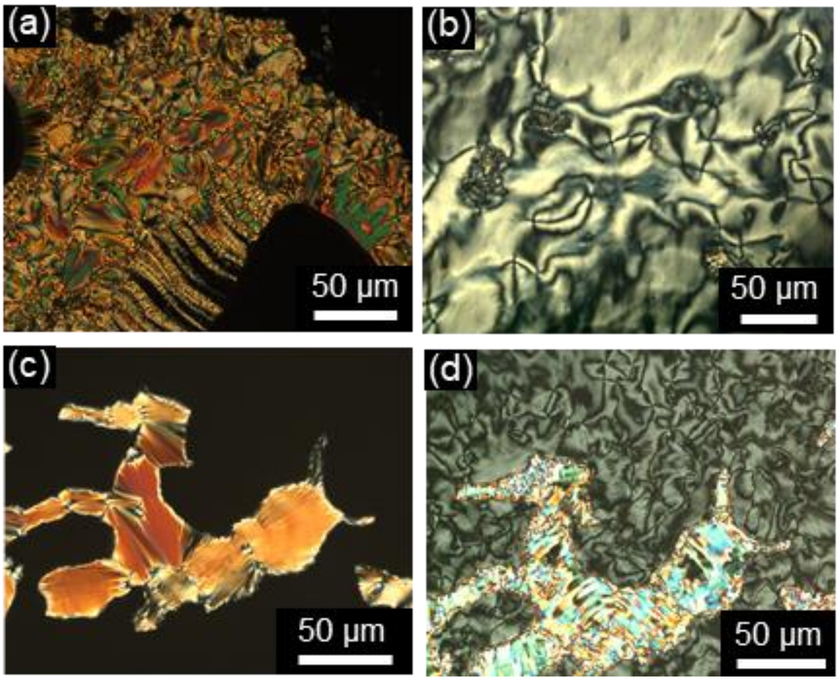

Fig. S16 Textures of imidazolium bromides **Im(O10,Om)Br** as seen between crossed polarizers upon cooling from the isotropic liquid (cooling rate 5 K min^{-1}; magnification 200x. (a, b) **Im(O10,O10)Br** at 179°C and at 120°C, (c, d) **Im(O10,O12)Br** at 130°C and at 121°C.

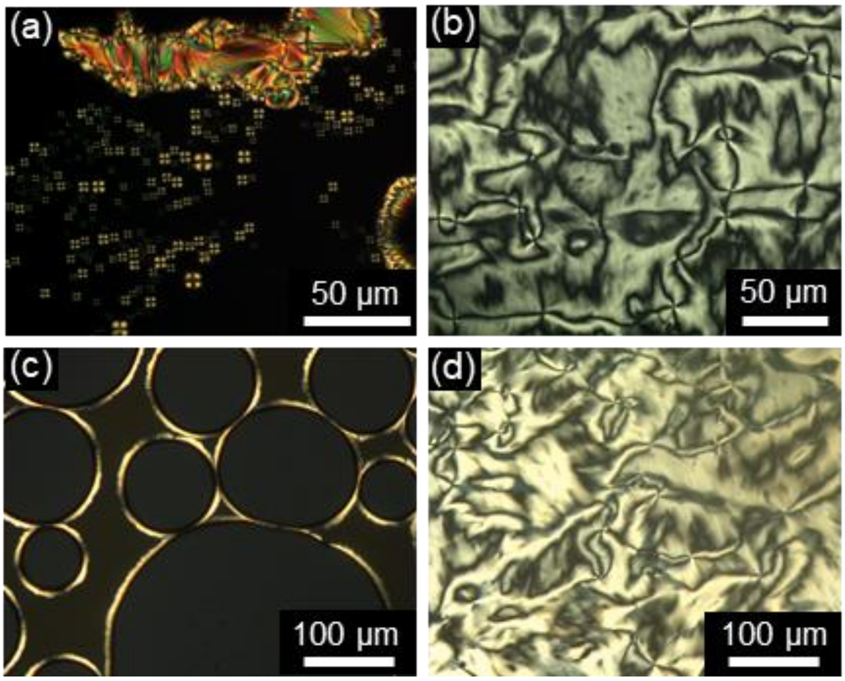

Fig. S17 Textures of imidazolium bromides **Im(O12,Om)Br** as seen between crossed polarizers upon cooling from the isotropic liquid (cooling rate 5 K min^{-1}; (a, b) magnification 200x, (c, d) magnification 100x). (a, b) **Im(O12,O10)Br** at 163°C and at 140°C, (c, d) **Im(O12,O12)Br** at 188°C and at 137°C.

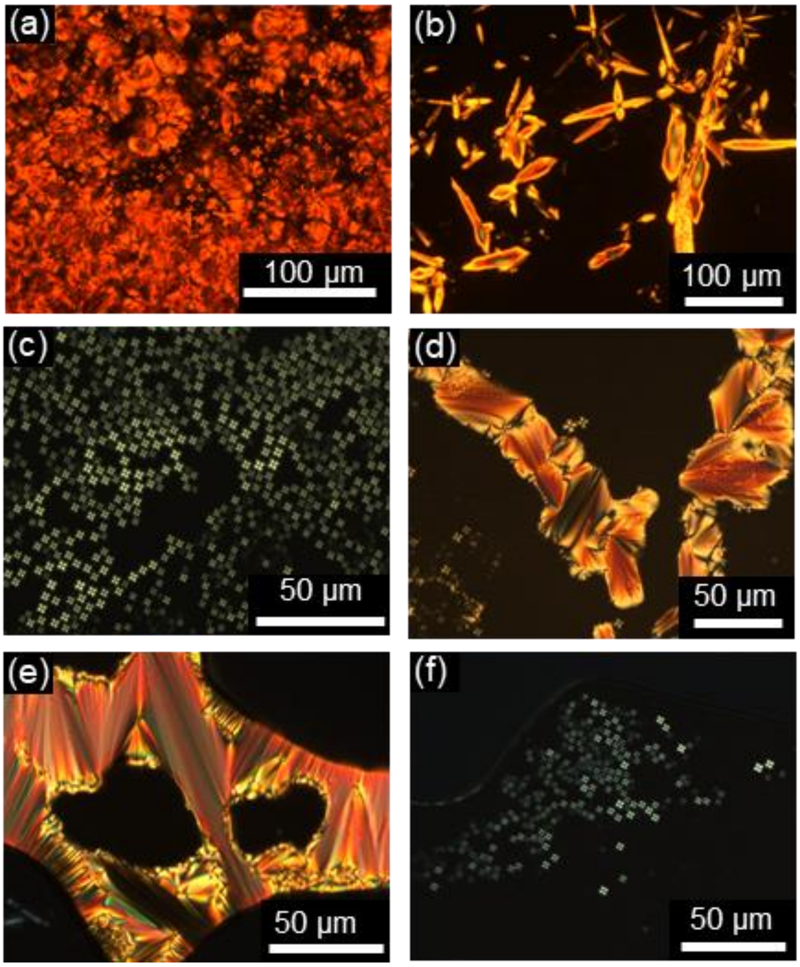

Fig. S18 Textures of imidazolium bromides **ImR(O4,Sm)Br** as seen between crossed polarizers upon cooling from the isotropic liquid (cooling rate 5 K min^{-1}; (a, c - f) magnification 200x, (b) magnification 100x. (a) **ImH(O4,S12)Br** at 141°C, (b) **ImH(O4,S14)Br** at 197°C, (c) **ImMe(O4,S12)Br** at 171°C, (d) **ImMe(O4,S14)Br** at 200°C, (e) **ImEt(O4,S10)Br** at 166°C, (f) **ImEt(O4,S12)Br** at 189°C.

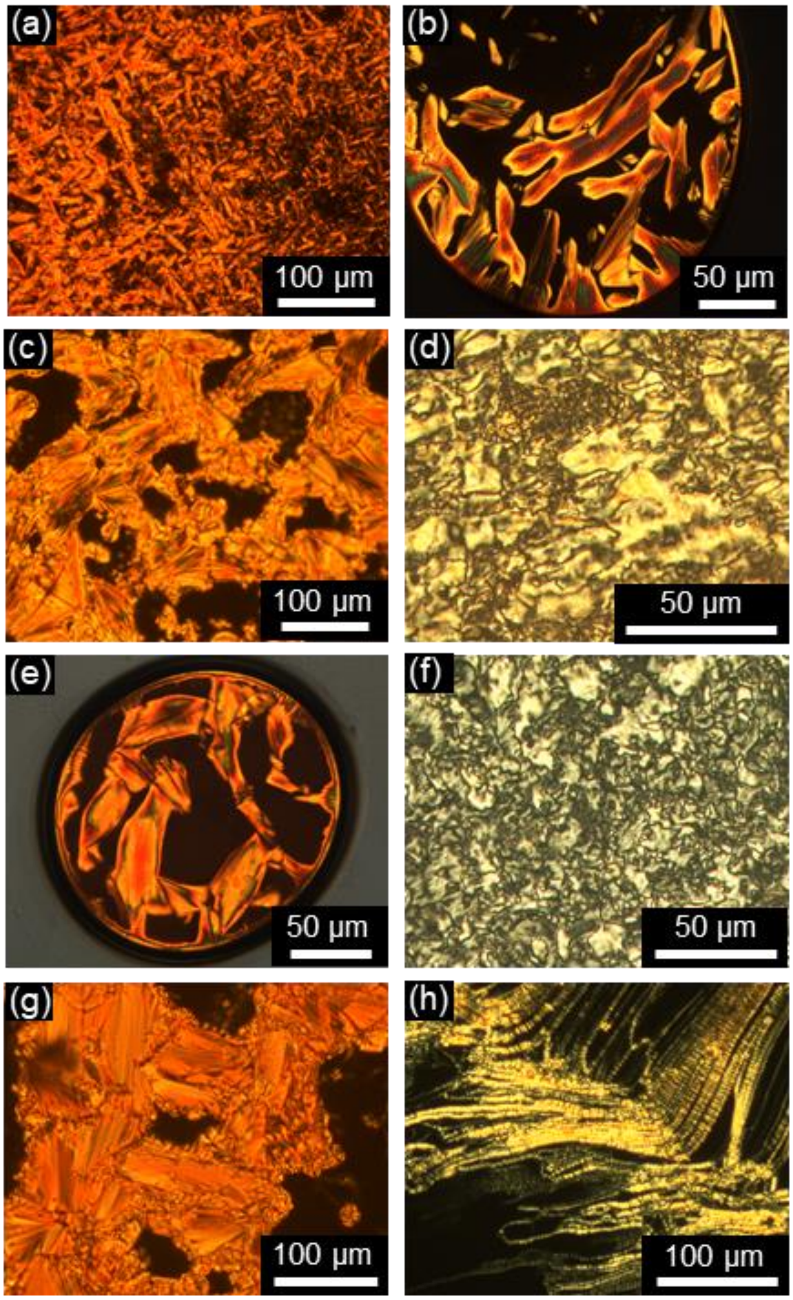

Fig. S19 Textures of imidazolium bromides **ImR(O6,Sm)Br** as seen between crossed polarizers upon cooling from the isotropic liquid (cooling rate 5 K min^{-1}; (a, c, g, h) magnification 100x, (b, d-f) magnification 200x. (a) **ImH(O6,S12)Br** at 180°C, (b) **ImH(O4,S14)Br** at 177°C, (c, d) **ImMe(O6,S12)Br** at 180°C and at 75°C, (e, f) **ImMe(O6,S14)Br** at 179°C and at 72°C, (g) **ImEt(O6,S10)Br** at 194°C, (h) **ImEt(O6,S12)Br** at 160°C.

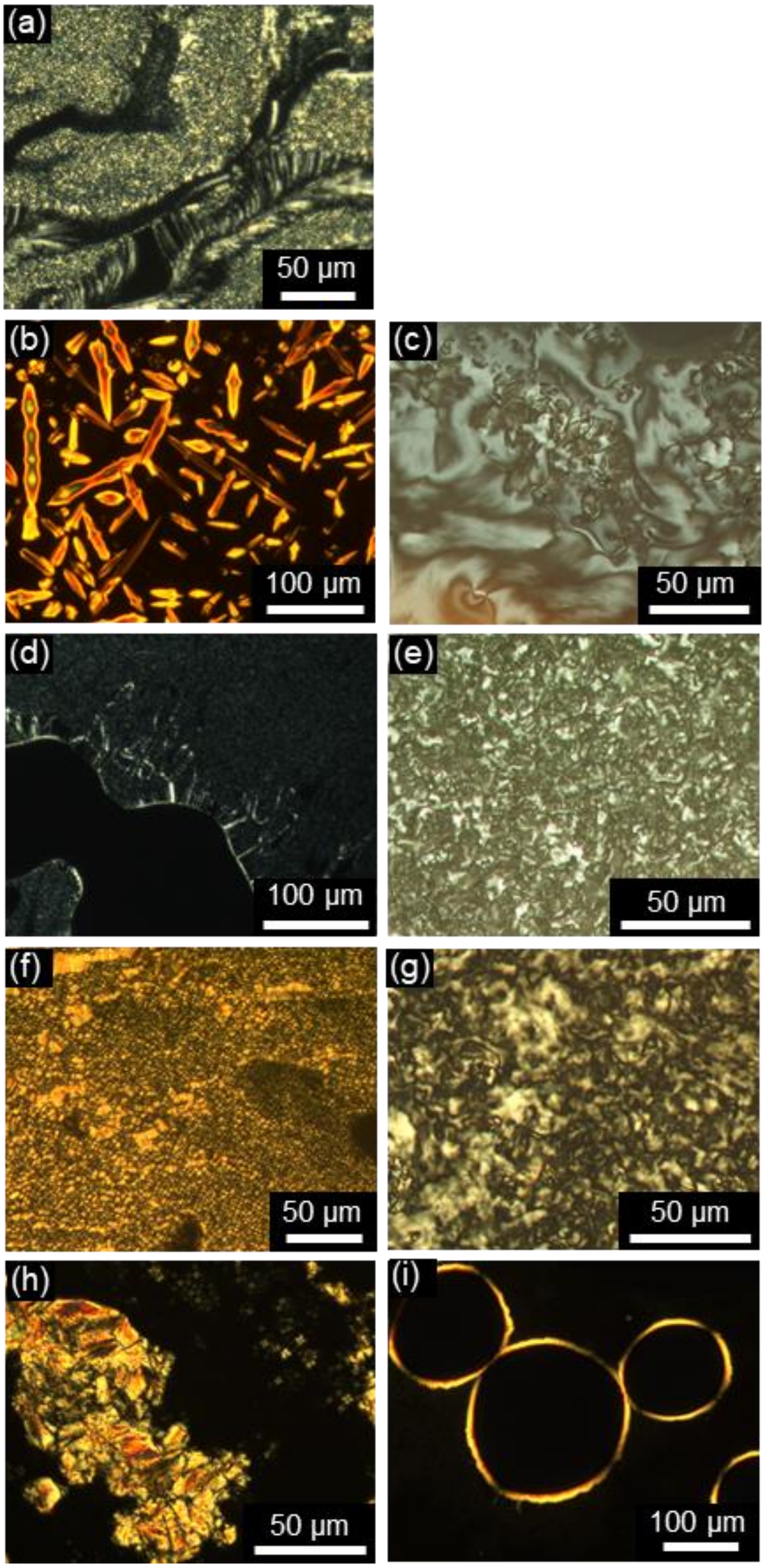

Fig. S20 Textures of imidazolium bromides **ImR(O8,Sm)Br** as seen between crossed polarizers upon cooling from the isotropic liquid (cooling rate 5 K min^{-1}; (a, b, d, f, i) magnification 100x, (c, e, g, h) magnification 200x. (a) **ImH(O8,S12)Br** at 188°C, (b, c) **ImH(O8,S14)Br** at 200°C and at 80°C, (d, e) **ImMe(O8,S12)Br** at 192°C and at 50°C, (f, g) **ImMe(O8,S14)Br** at 198°C and at 55°C, (h) **ImEt(O8,S10)Br** at 159°C, (i) **ImEt(O8,S10)Br** at 210°C.

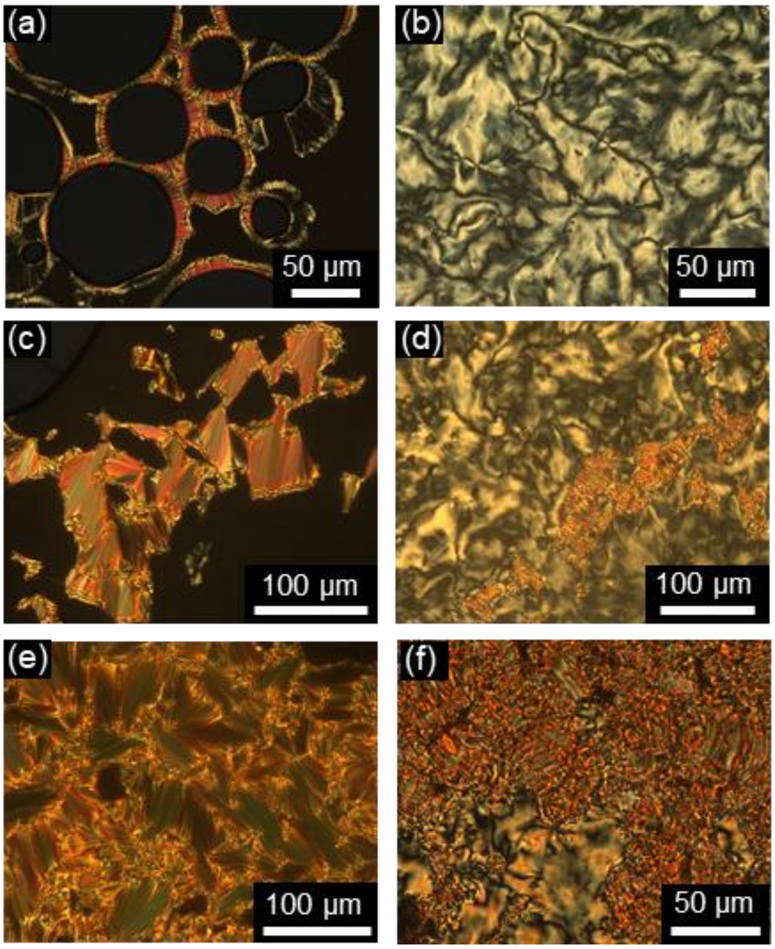

Fig. S21 Textures of imidazolium bromides **ImH(O10,Sm)Br** as seen between crossed polarizers upon cooling from the isotropic liquid (cooling rate 5 K min^{-1}; (c, d, e) magnification 100x, (a, b, f) magnification 200x. (a, b) **ImH(O10,S10)Br** at 127°C and at 80°C, (c, d) **ImH(O10,S12)Br** at 128°C and at 94°C, (e, f) **ImH(O10,S14)Br** at 200°C and at 85°C.

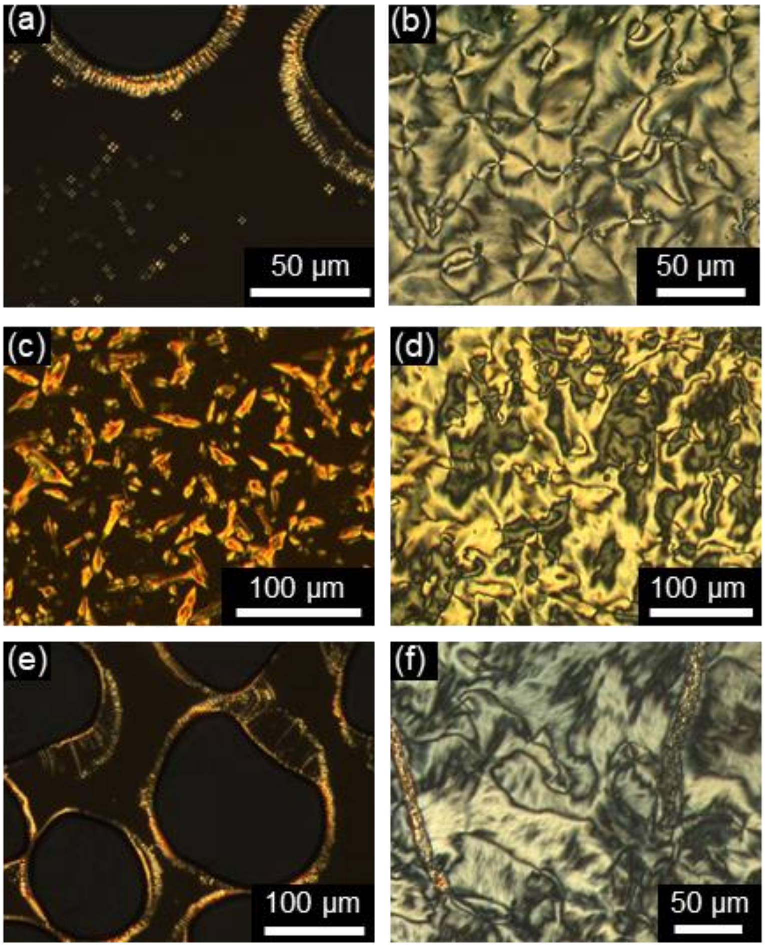

Fig. S22 Textures of imidazoliumbromides **ImH(O12,Sm)Br** as seen between crossed polarizers upon cooling from the isotropic liquid (cooling rate 5 K min^{-1}; (c-f) magnification 100x, (a, b) magnification 200x. (a, b) **ImH(O12,S10)Br** at 104°C and at 95°C, (c, d) **ImH(O12,S12)Br** at 172°C and at 105°C, (e, f) **ImH(O12,S14)Br** at 157°C and at 90°C.

XRD data of bromides and imidazolium salts

Table S6 XRD data, theoretically calculated molecular lengths L_{calc} and ratios d / L_{calc} of bromides **Br(On,Ym)**.

Compd	Mesophase	d / Å	Miller indices	L_{calc} / Å	Ratio d / L_{calc}
**Br(O4,O12)**	SmA	31.1	(001)	31.5	0.99
	at 56 °C	4.0	(halo)		
**Br(O4,O14)**	SmA	33.7	(001)	34.0	0.99
	at 61 °C	4.3	(halo)		
**Br(O4,O16)**	SmA	35.6	(001)	36.5	0.98
	at 68 °C	4.4	(halo)		
**Br(O6,O8)**	SmA	28.8	(001)	29.2	0.99
	at 45 °C	14.4	(002)		
		4.4	(halo)		
**Br(O6,O10)**	SmA	31.2	(001)	31.7	0.98
	at36 °C	4.3	(halo)		
**Br(O6,O12)**	SmA	33.1	(001)	34.2	0.97
	at 59 °C	4.3	(halo)		
**Br(O6,O14)**	SmA	36.3	(001)	36.7	0.99
	at 60 °C	4.3	(halo)		
**Br(O6,O16)**	SmA	38.9	(001)	39.2	0.99
	at 60 °C	4.3	(halo)		
**Br(O8,O10)**	SmA	34.0	(001)	34.2	0.99
	at 50 °C	4.3	(halo)		
**Br(O8,O16)**	SmA	41.0	(001)	41.3	0.99
	at 69 °C	4.2	(halo)		
**Br(O10,O12)**	SmA	39.0	(001)	39.2	0.99
	at 60 °C	4.5	(halo)		
**Br(O12,O12)**	SmA	41.0	(001)	41.6	0.99
	at 68 °C	4.6	(halo)		
**Br(O6,S10)**	SmA	31.4	(001)	31.7	0.99
	at 51 °C	4.3	(halo)		
**Br(O8,S12)**	SmA	35.9	(001)	36.5	0.98
	at 59 °C	18.0	(002)		
		4.3	(halo)		

Table S7 XRD data, theoretically calculated molecular lengths L_{calc} and ratios d / L_{calc} of imidazolium salts **Im(On,Om)Br** and **Im(On,Om)OTf**.

Compd	Mesophase	d / Å	Miller indices	L_{calc} / Å	Ratio d / L_{calc}
**Im(O4,O12)Br**	SmA	61.3	(001)	34.6	1.77
	at 95 °C	30.5	(002)		
		4.5	(halo)		
**Im(O4,O14)Br**	SmA	64.6	(001)	37.1	1.74
	at 96 °C	32.2	(002)		
		4.2	(halo)		
**Im(O4,O16)Br**	SmA	68.0	(001)	39.6	1.72
	at 112 °C	34.1	(002)		
		22.7	(003)		
		4.3	(halo)		
**Im(O4,O14)OTf**	SmA	62.7	(001)	37.1	1.69
	at 90 °C	31.3	(002)		
		4.6	(halo)		
**Im(O6,O8)Br**	SmC	56.7	(001)	32.2	1.76
	at 81 °C	28.2	(002)		
		18.8	(003)		
	SmA	57.7	(001)		1.79
	at 100 °C	28.7	(002)		
		19.0	(003)		
		4.2	(halo)		
**Im(O6,O10)Br**	SmC	63.1	(001)	34.6	1.82
	at 92 °C	31.5	(002)		
		20.8	(003)		
	SmA	63.2	(001)		1.83
	at 115 °C	31.5	(002)		
		20.9	(003)		
		4.2	(halo)		
**Im(O6,O12)Br**	SmC	63.1	(001)	37.2	1.70
	at 95 °C	31.5	(002)		
		21.0	(003)		
		4.3	(halo)		
	SmA	63.6	(001)		1.71
	at 125 °C	31.7	(002)		
		21.2	(003)		
		4.6	(halo)		
**Im(O6,O14)Br**	SmC	69.3	(001)	39.7	1.75
	at 102 °C	34.6	(002)		
	SmA	69.8	(001)		1.76
	at 116 °C	34.9	(002)		
		4.2	(halo)		
**Im(O6,O16)Br**	SmC	70.0	(001)	42.2	1.66
	at 114 °C	34.9	(002)		
		4.3	(halo)		
	SmA	70.2	(001)		1.66
	at 132 °C	35.0	(002)		
**Im(O6,O8)OTf**	SmA	54.1	(001)	32.2	1.68
	at 102 °C	27.0	(002)		
		18.0	(003)		

		4.4	(halo)		
**Im(O6,O14)OTf**	SmA	66.9	(001)	39.7	1.69
	at 100 °C	33.4	(002)		
		22.4	(003)		
		4.6	(halo)		
**Im(O8,O10)Br**	SmC	65.1	(001)	36.8	1.77
	at 70 °C	32.3	(002)		
		21.5	(003)		
	SmA	65.7	(001)		1.79
	at 98 °C	32.7	(002)		
		21.6	(003)		
		4.6	(halo)		
**Im(O8,O12)Br**	SmC	66.1	(001)	39.3	1.68
	at 81 °C	32.9	(002)		
		21.9	(003)		
		4.6	(halo)		
	SmA	66.3	(001)		1.69
	at 138 °C	32.9	(002)		
		21.9	(003)		
		4.7	(halo)		
**Im(O8,O16)Br**	SmC	73.7	(001)	44.3	1.66
	at 98 °C	36.9	(002)		
		24.6	(003)		
		18.3	(004)		
		4.3	(halo)		
	SmA	74.1	(001)		1.67
	at 121 °C	37.1	(002)		
		24.7	(003)		
		18.3	(004)		
		4.7	(halo)		
**Im(O8,O10)OTf**	SmA	61.4	(001)	36.8	1.67
	at 112 °C	30.7	(002)		
		20.4	(003)		
		4.6	(halo)		
**Im(O10,O10)Br**	SmC	63.4	(001)	39.3	1.61
	at 131 °C	31.7	(002)		
		21.0	(003)		
		4.7	(halo)		
	SmA	64.7	(001)		1.65
	at 159 °C	32.2	(002)		
		21.4	(003)		
		4.8	(halo)		
**Im(O10,O12)Br**	SmC	67.1	(001)	41.8	1.61
	at 117 °C	33.5	(002)		
		22.2	(003)		
		16.7	(004)		
		4.7	(halo)		
	SmA	67.9	(001)		1.62
	at 161 °C	33.9	(002)		
		22.5	(003)		
		4.7	(halo)		
**Im(O12,O10)Br**	SmC	66.1	(001)	41.7	1.59
	at 117 °C	33.0	(002)		

		21.9	(003)		
		4.6	(halo)		
	SmA	66.6	(001)		1.60
	at 171 °C	33.3	(002)		
		22.0	(003)		
		4.7	(halo)		
**Im(O12,O12)Br**	SmC	71.5	(001)	44.2	1.62
	at 131 °C	35.7	(002)		
		23.7	(003)		
		17.8	(004)		
		4.7	(halo)		
	SmA	71.7	(001)		1.62
	at 173 °C	35.8	(002)		
		23.7	(003)		
		4.7	(halo)		

Table S8 XRD data, theoretically calculated molecular lengths L_{cac} and ratios d / L_{calc} of imidazolium bromides **ImR(On,Sm)Br**.

Compd	Mesophase	d / Å	Miller indices	L_{calc} / Å	Ratio d / L_{calc}
**ImH(O4,S12)Br**	SmA	60.2	(001)	35.0	1.72
	at 107 °C	30.1	(002)		
		4.4	(halo)		
**ImH(O4,S14)Br**	SmA	64.3	(001)	37.5	1.72
	at 110 °C	32.2	(002)		
		4.4	(halo)		
**ImMe(O4,S12)Br**	SmA	59.6	(001)	35.0	1.70
	at 120 °C	29.8	(002)		
		4.6	(halo)		
**ImMe(O4,S14)Br**	SmA	64.9	(001)	37.5	1.73
	at 100 °C	32.5	(002)		
		4.6	(halo)		
**ImEt(O4,S10)Br**	SmA	55.2	(001)	32.5	1.70
	at 100 °C	27.6	(002)		
		18.3	(003)		
		4.7	(halo)		
**ImEt(O4,S12)Br**	SmA	60.2	(001)	35.0	1.72
	at 100 °C	30.1	(002)		
		4.6	(halo)		
**ImH(O6,S12)Br**	SmA	68.9	(001)	37.6	1.83
	at 74 °C	34.3	(002)		
		22.8	(003)		
		4.4	(halo)		
**ImH(O6,S14)Br**	SmA	70.1	(001)	40.1	1.75
	at 100 °C	35.0	(002)		
		4.5	(halo)		
**ImMe(O6,S12)Br**	SmC	68.9	(001)	37.6	1.83
	at 60 °C	34.3	(002)		
		22.7	(003)		
	SmA	68.9	(001)		1.83
	at 78 °C	34.3	(002)		
		22.8	(003)		

		4.4	(halo)		
**ImMe(O6,S14)Br**	SmC at 74 °C	70.6	(001)	40.1	1.76
		35.2	(002)		
	SmA at 85 °C	71.1	(001)		1.77
		35.5	(002)		
		4.7	(halo)		
**ImEt(O6,S10)Br**	SmA at 62 °C	62.0	(001)	35.0	1.77
		30.9	(002)		
		20.6	(003)		
		4.7	(halo)		
**ImEt(O6,S12)Br**	SmC at 51 °C	65.7	(001)	37.6	1.75
		32.7	(002)		
		21.8	(003)		
	SmA at 74 °C	65.7	(001)		1.75
		32.8	(002)		
		21.8	(003)		
		4.4	(halo)		
**ImH(O8,S12)Br**	SmA at 93 °C	73.9	(001)	39.7	1.86
		36.9	(002)		
		24.5	(003)		
		4.4	(halo)		
**ImH(O8,S14)Br**	SmC at 96 °C	72.8	(001)	42.2	1.73
		36.3	(002)		
		24.2	(003)		
		18.0	(004)		
		4.4	(halo)		
	SmA at 134 °C	70.7	(001)		1.68
		35.3	(002)		
		23.5	(003)		
		17.5	(004)		
		4.7	(halo)		
**ImMe(O8,S12)Br**	SmC at 74 °C	70.0	(001)	39.7	1.76
		35.0	(002)		
		23.2	(003)		
		17.4	(004)		
		4.4	(halo)		
	SmA at 99 °C	70.7	(001)		1.78
		35.3	(002)		
		23.4	(003)		
**ImMe(O8,S14)Br**	SmC at 81 °C	71.6	(001)	42.2	1.70
		35.7	(002)		
		23.7	(003)		
		4.4	(halo)		
	SmA at 110 °C	71.9	(001)		1.70
		35.9	(002)		
		23.9	(003)		
**ImEt(O8,S10)Br**	SmC at 47 °C	66.5	(001)	37.2	1.79
		33.4	(002)		
		22.2	(003)		
	SmA at 59 °C	67.3	(001)		1.81
		33.6	(002)		
		22.4	(003)		
		4.7	(halo)		

**ImEt(O8,S12)Br**	SmC	69.8	(001)	39.7	1.76
	at 68 °C	34.8	(002)		
		23.2	(003)		
	SmA	70.2	(001)		1.77
	at 85 °C	35.1	(002)		
		23.3	(003)		
		4.7	(halo)		

Temperature-dependent layer distance

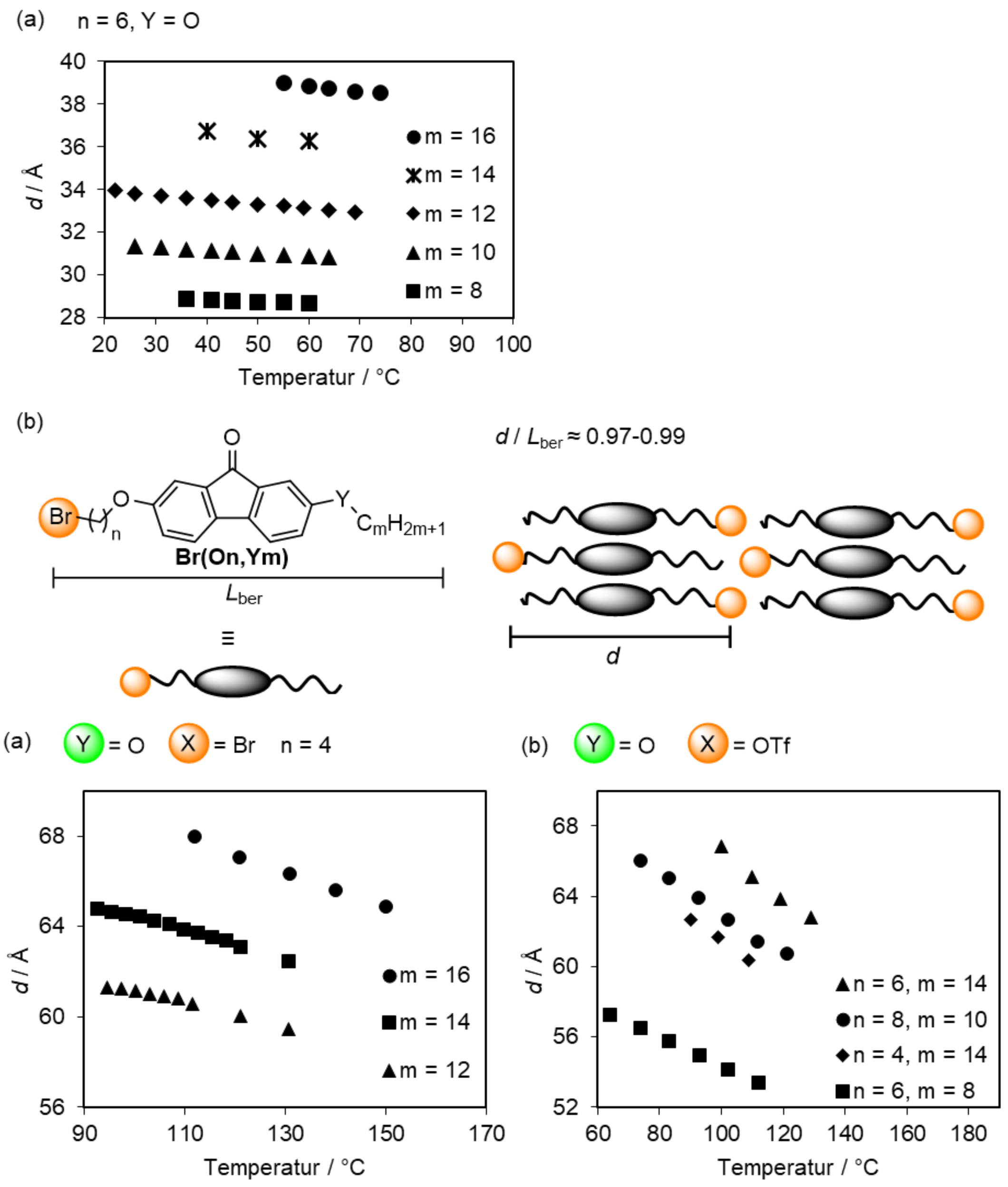

Fig. S23 Top: Temperature-dependent layer distance *d* of bromides **Br(O6,Om)** and packing model of the SmA phase. Below: Temperature-dependent layer distance *d* of (a) **Im(O4,Om)Br** and (b) **Im(On,Om)OTf**.

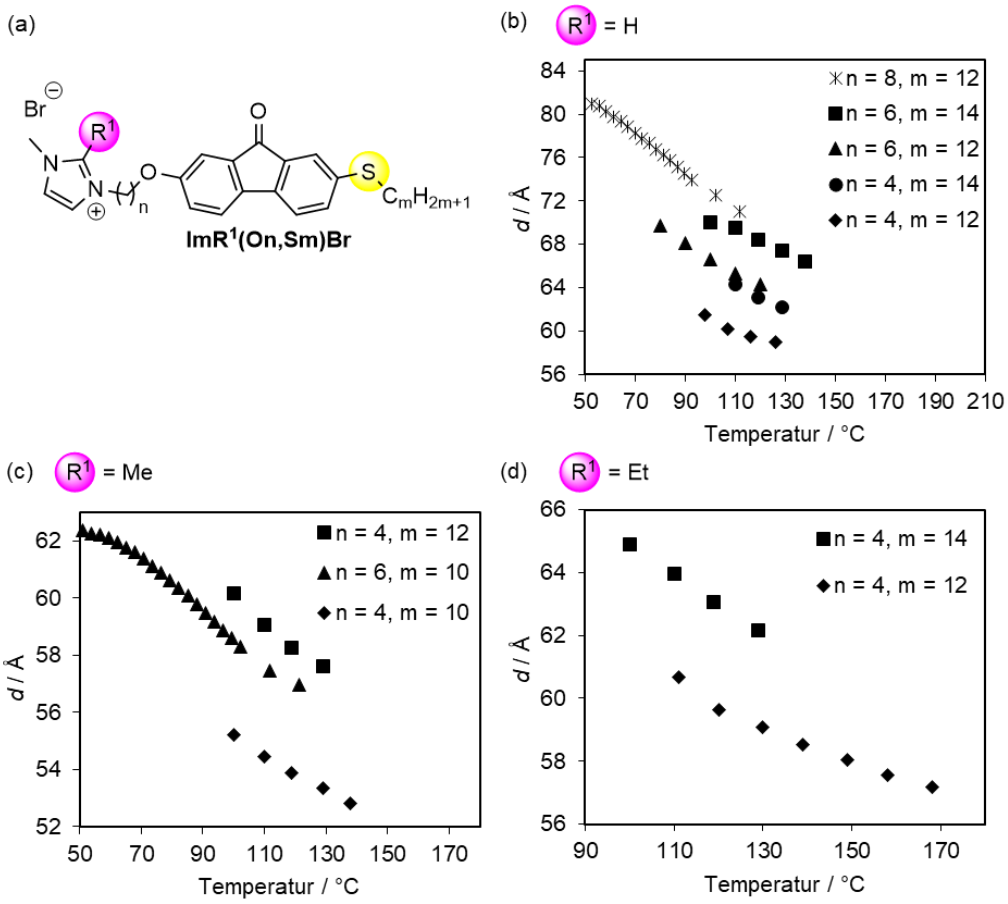

Fig. S24 (a) Thioether ILCs. Temperature-dependent layer distance *d* of (b) **ImH(On,Sm)Br**, (c) **ImMe(On,Sm)Br** and (d) **ImEt(On,Sm)Br**.

Optical tilt angles

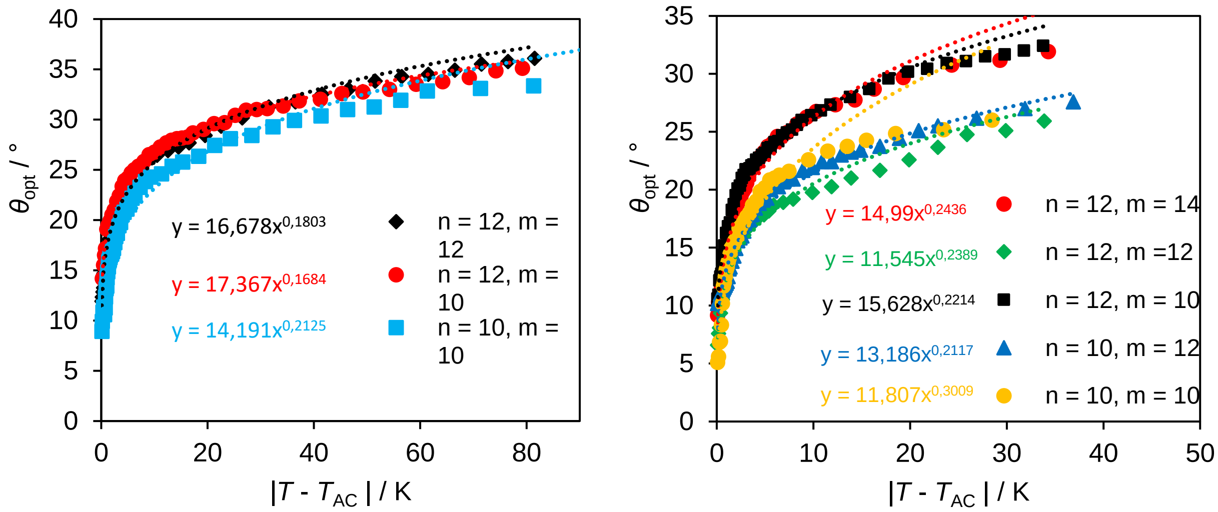

Fig. S25 θ_{opt}(T) profiles fitted to the power law ($\theta \propto |T - T_{AC}|^{\beta}$) [14] for (a) **Im(On,Om)Br** and (b) **Im(On,Sm)Br**.

Electron density profiles

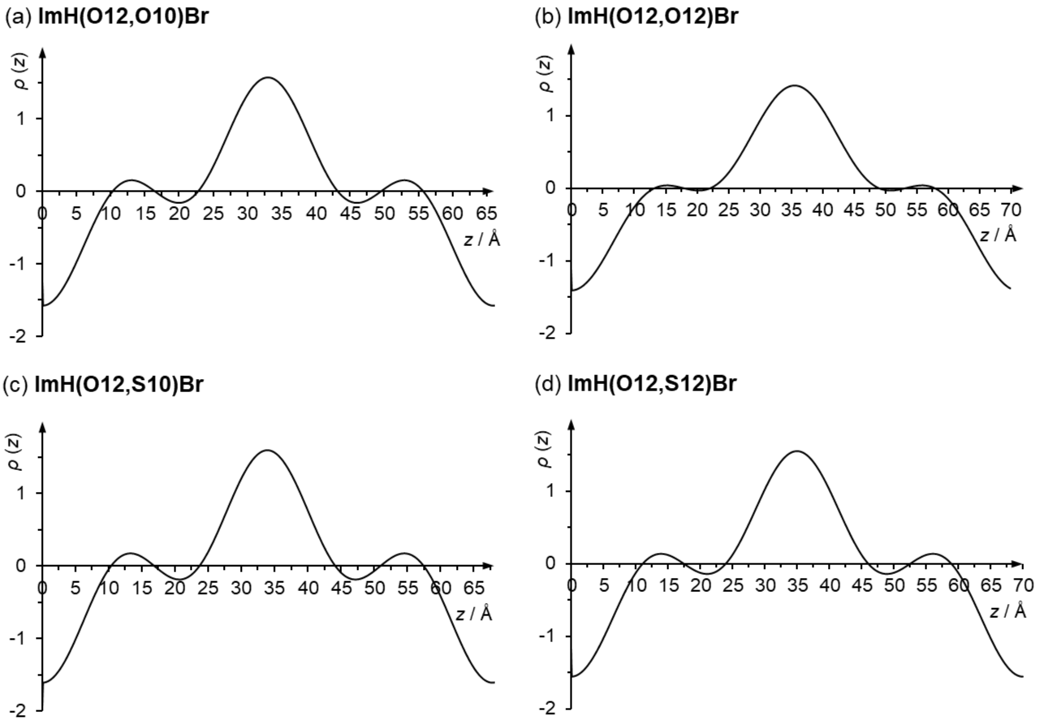

Fig. S26 Electron density profiles of imidazolium bromides **Im(On,Ym)Br** at (a) 167 °C, (b) 176 °C, (c) 160 °C, and (d) 164 °C.

Absorbance and emission measurements in solution

A comparison of fluorescence spectra in dilute solution of precursor fluorenones **Br(On,Ym)** with ILCs **Im(On,Ym)X** is depicted in Fig. S28. All compounds were excited at λ_{exc} = 283 nm. Emission maxima of **Br(On,O16)** were detected at λ_{em} = 336–358 nm (Fig. S28a) with an additional weak band at 448 nm and 444 nm for **Br(O6,O16)** and **Br(O8,O16)**, respectively. Presumably the weak bands are derived from an intramolecular exciplex formation via bromide-fluorenone interaction, which is supported by absence of the weak band for **Br(O4,O16)**, because the C_4 spacer is too short to accommodate the intramolecular exciplex. The emission maximum of **Br(O8,O12)** was bathochromically shifted to λ_{em} = 355 nm and two shoulders at shorter and longer wavelengths were observed. This bathochromic shift was more pronounced for the corresponding **Br(O8,S12)** with thioether side chain, resulting in an emission maximum at λ_{em} = 399 nm.

Both **ILCs Im(O8,O12)Br** and **Im(O8,S12)Br** emitted blue light with emission maxima at λ_{em} = 409 nm (Fig. S28b). The emission band of **Im(O6,O8)OTf** was hypsochromically shifted by 50 nm to λ_{em} = 359 nm as compared to the above mentioned bromides. The C_6 linker is too short for good interaction between imidazolium and fluorenone in an intramolecular exciplex and thus only monomer emission was visible. For the C_8 linker the interaction is more pronounced and thus both monomer and exciplex emission were observed.

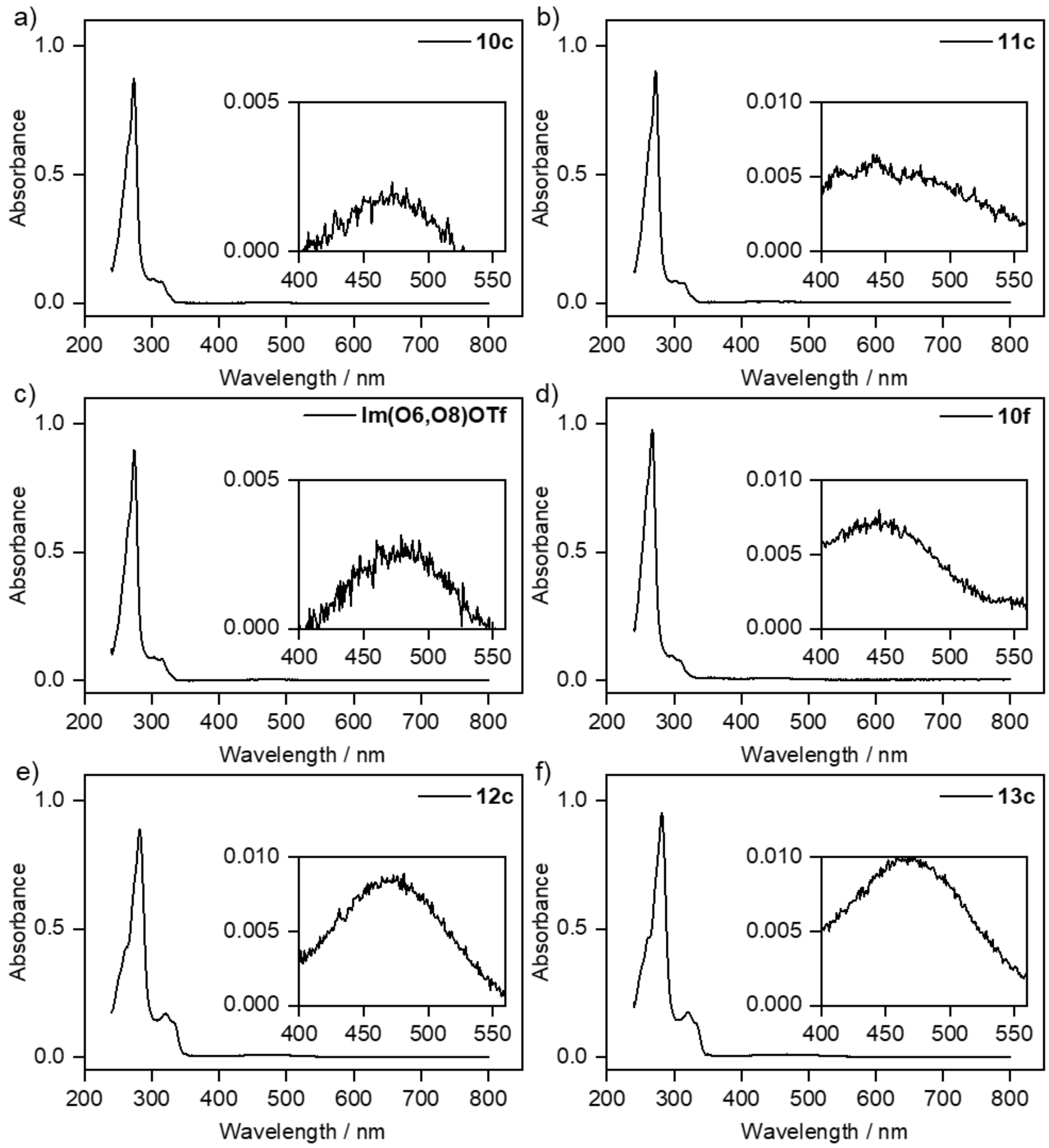

Fig. S27 Absorbance spectra in $CHCl_3$ ($c = 10^{-5}$ mol L^{-1}) of (a) **10c**, (b) **11c**, (c) **Im(O6,O8)OTf**, (d) **10f**, (e) **12c** and (f). The bands in the visible region are shown in the corresponding insets.

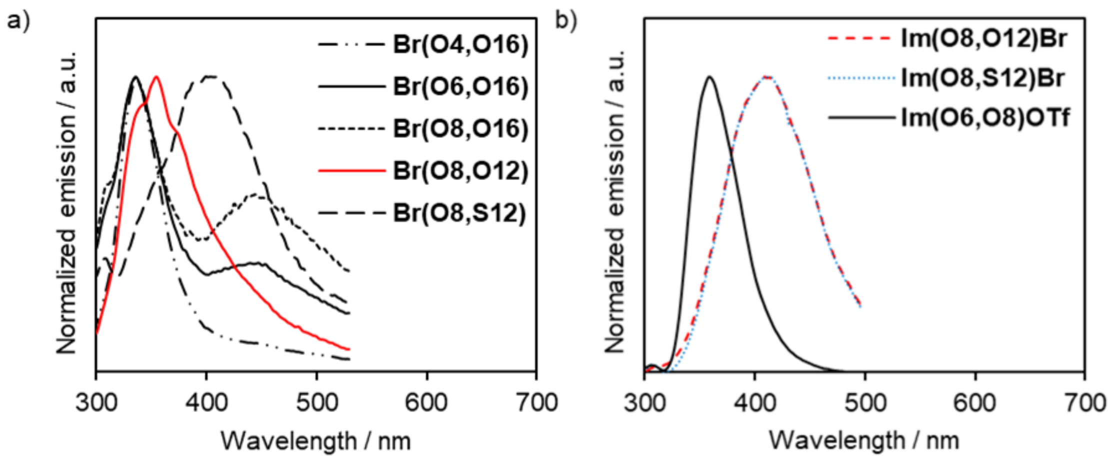

Fig. S28 Normalized emission spectra (in $CHCl_3$ $c = 10^{-6}$ mol L^{-1}, $\lambda_{exc} = 283$ nm) of (a) bromides **Br(On,Ym)** and (b) imidazolium salts **Im(On,Ym)X**.

To extinguish reabsorption phenomena, further emission experiments on **Im(O8,O12)Br**, **Im(O8,S12)Br** and **Im(O6,O8)OTf** were conducted. Therefore, two different excitation wavelengths were chosen. At first, those compounds were excited in the shoulder at λ_{exc} = 340 nm to avoid reabsorption in the UV-area. Furthermore, those compounds were excited at λ_{exc} = 500 nm to study the absorption band in the visible region (Fig. S29). When exciting at λ_{exc} = 340 nm **Im(O8,O12)Br** and **Im(O8,S12)Br** both display one sharp (λ_{em} = 376 nm) and one broad (λ_{em} = 431 nm) emission signal, whereas for compound **Im(O6,O8)OTf** only the sharper band at λ_{em} = 376 nm could be seen. Presumably, the band at 376 nm corresponds to the monomer emission, while the peak around 431 nm might come from i.e. fluorenone and imidazolium forming intramolecular π-cation interactions, as excimer emission resulting from fluorenone-fluorenone interactions should not occur at low concentrations below 10^{-5} M. Related phenomena were previously reported by Wang for 2,7-diphenylfluorenone-diboronic acids capable of hydrogen bonding.[13] When excited at 500 nm, **Im(O8,O12)Br** and **Im(O8,S12)Br** do not show any emission (Fig. S29b). Surprisingly, **Im(O6,O8)OTf** exhibited green emission at 532 nm. The different emission behaviour of **Im(O6,O8)OTf** as compared to bromide-containing ILCs salts might be due to (a) the bulkiness of the triflate as compared to bromide.

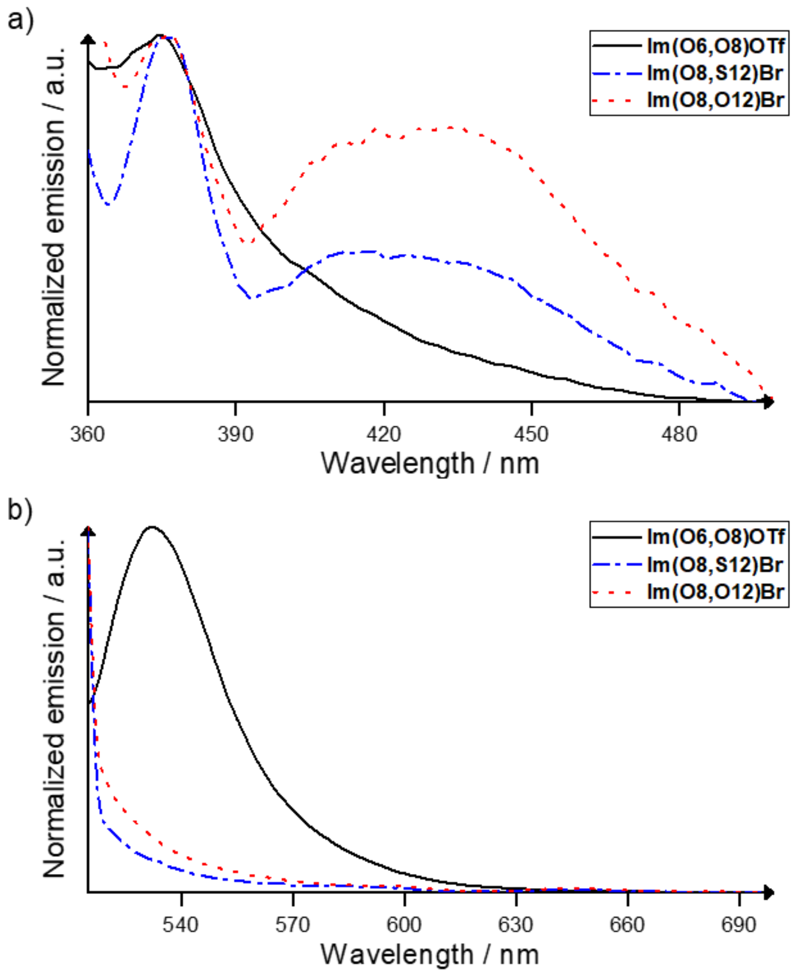

Fig. S29 Normalized emission spectra in $CHCl_3$ ($c = 10^{-6}$ mol L^{-1}) of **Im(O8,O12)Br** (red dots), **Im(O8,S12)Br** (blue dashes) and **Im(O6,O8)OTf** (black line) at excitation wavelengths of a) λ_{exc} = 340 nm and b) λ_{exc} = 500 nm.

Solid state fluorescence spectroscopy

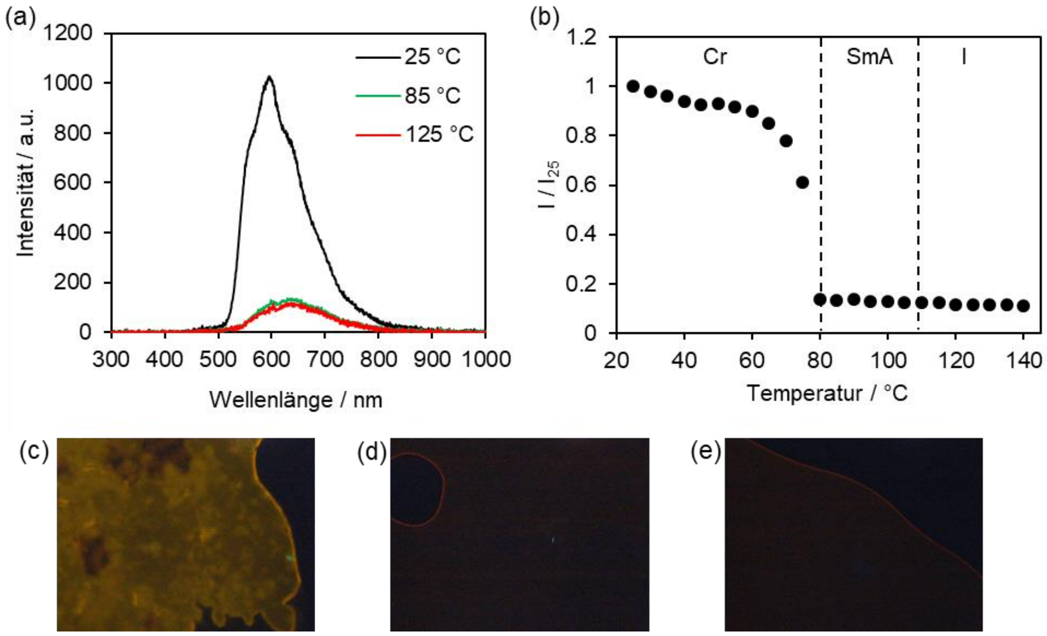

Fig. S30 (a) Emission spectra of **Im(O6,O8)OTf** at different temperatures (λ_{exc} = 350–380 nm), (b) temperature-dependent emission intensity upon 2. heating (heating rate 5 K min^{-1}). Transition temperatures (dashed lines) were determined by DSC from the 3. heating scan (Table S3). (c – e) POM pictures under UV irradiation at 25 °C, 85 °C, 125 °C, irradiation time 4 s.

References

1 Q. Song, D. Nonnenmacher, F. Giesselmann, R. P. Lemieux, *J. Mater. Chem. C* **2013**, *1*, 343–350.
2 M.-C. Yeh, Y.-L. Su, M.-C. Tzeng, C. W. Ong, T. Kajitani, H. Enozawa, M. Takata, Y. Koizumi, A. Saeki, S. Seki, T. Fukushima, *Angew. Chem. Int. Ed.* **2013**, *52*, 1031–1034.
3 A. Schultz, S. Laschat, A. Saipa, F. Gießelmann, M. Nimtz, J. L. Schulte, A. Baro, B. Miehlich, *Adv. Funct. Mater.* **2004**, *14*, 163–168.
4 J. R. Epperson, M. A. Bruce, J. D. Catt, J. A. Deskus, D. B. Hodges, G. N. Karageorge, D. J. Keavy, C. D. Mahle, R. J. Mattson, A. A. Ortiz, M. F. Parker, K. S. Takaki, B. T. Watson, J. P. Yevich, *Bioorg. Med. Chem.* **2004**, *12*, 4601–4611.
5 S. R. D. George, T. E. Elton, J. B. Harper, *Org. Biomol. Chem.* **2015**, *13*, 10745–10750.
6 X.-Y. Chen, S. Ozturk, E. J. Sorensen, *Org. Lett.* **2017**, *19*, 1140–1143.
7 J. Magano, M. H. Chen, J. D. Clark, T. Nussbaumer, *J. Org. Chem.* **2006**, *71*, 7103–7105.
8 J. Chae, *Arch. Pharm. Res.* **2008**, *31*, 305–309.
9 A. Jankowiak, Ż. Debska, J. Romański, P. Kaszyński, *J. Sulfur Chem.* **2012**, *33*, 1–7.
10 S. Choi, M. A. Larson, S. H. Hinrichs, P. Narayanasamy, *Bioorg. Med. Chem. Lett.* **2016**, *26*, 1997–1999.
11 G. F. Starkulla, S. Klenk, M. Butschies, S. Tussetschläger, S. Laschat, *J. Mater. Chem.* **2012**, *22*, 21987–21997.
12 M. Butschies, S. Sauer, E. Kessler, H.-U. Siehl, B. Claasen, P. Fischer, W. Frey, S. Laschat, *ChemPhysChem* **2010**, *11*, 3752–3765.
13 F. Xu, H. Wang, X. Du, W. Wang, D.-E. Wang, S. Chen, X. Han, N. Li, M.-S Yuan, J. Wang, *Dyes Pigm.* **2016**, *129*, 121–128.
14 Q. Song, D. Nonnenmacher, F. Giesselmann, R. P. Lemieux, *Chem. Commun.* **2011**, *47*, 4781–4783.

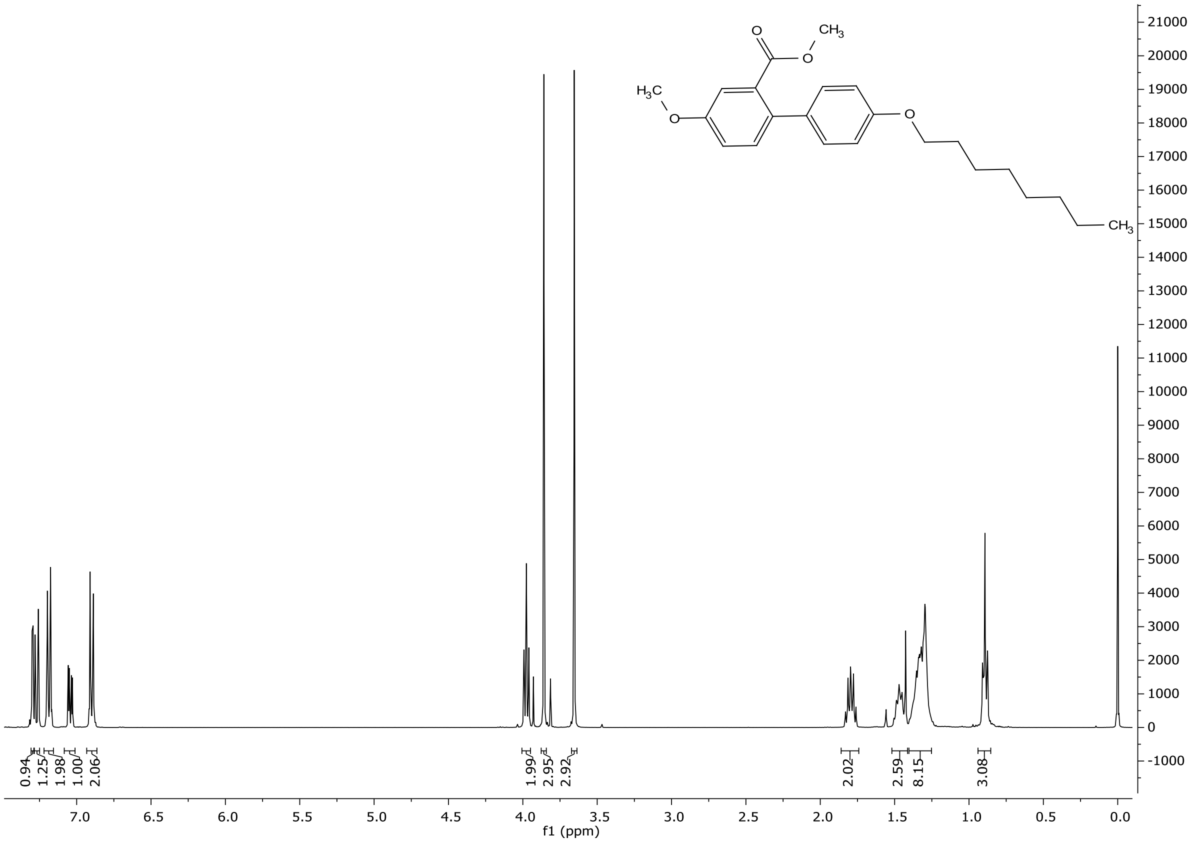

CH3
O
O
H3C
O
O
CH3
0.94
1.25
1.98
1.00
2.06
1.99
2.95
2.92
2.02
2.59
8.15
3.08
f1 (ppm)

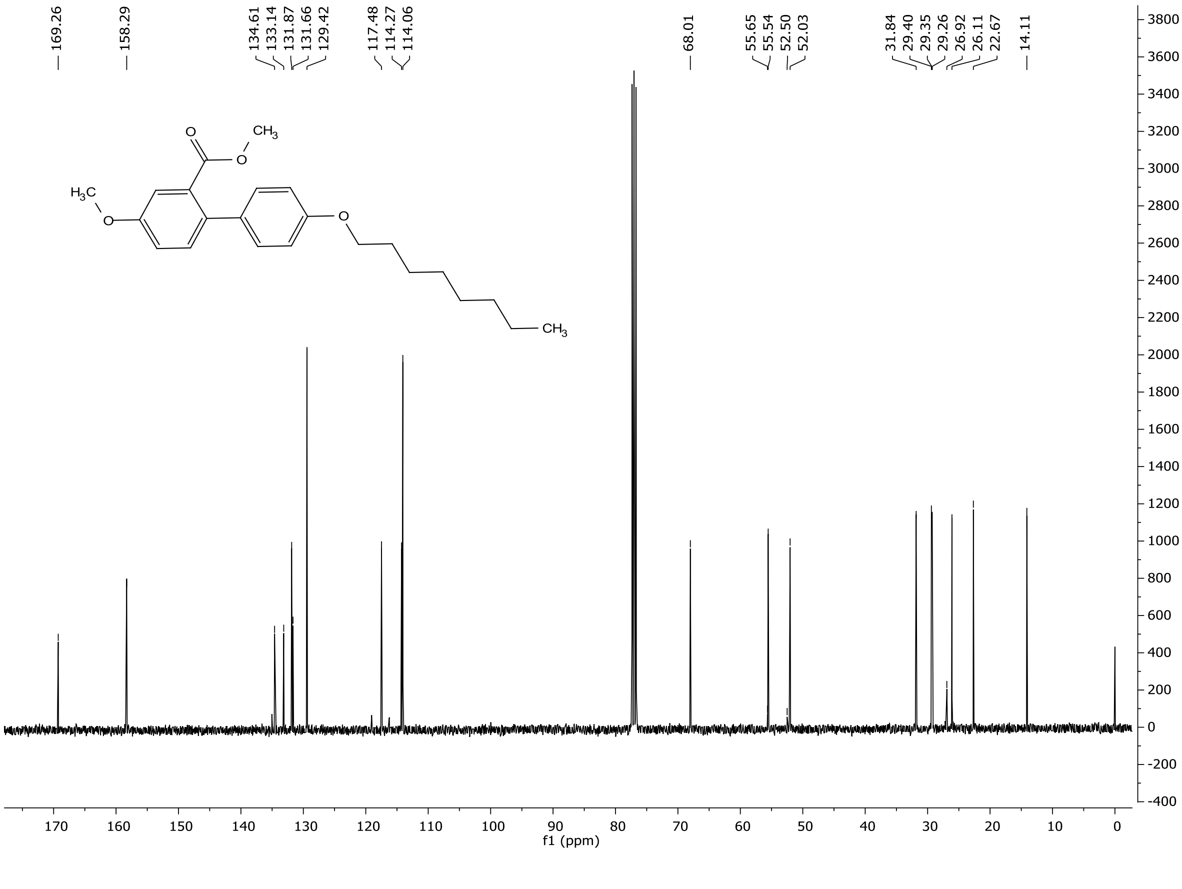

169.26
158.29
134.61
133.14
131.87
131.66
129.42
117.48
114.27
114.06
68.01
55.65
55.54
52.50
52.03
31.84
29.40
29.35
29.26
26.92
26.11
22.67
14.11
CH3
O
O
H3C
O
O
CH3
f1 (ppm)

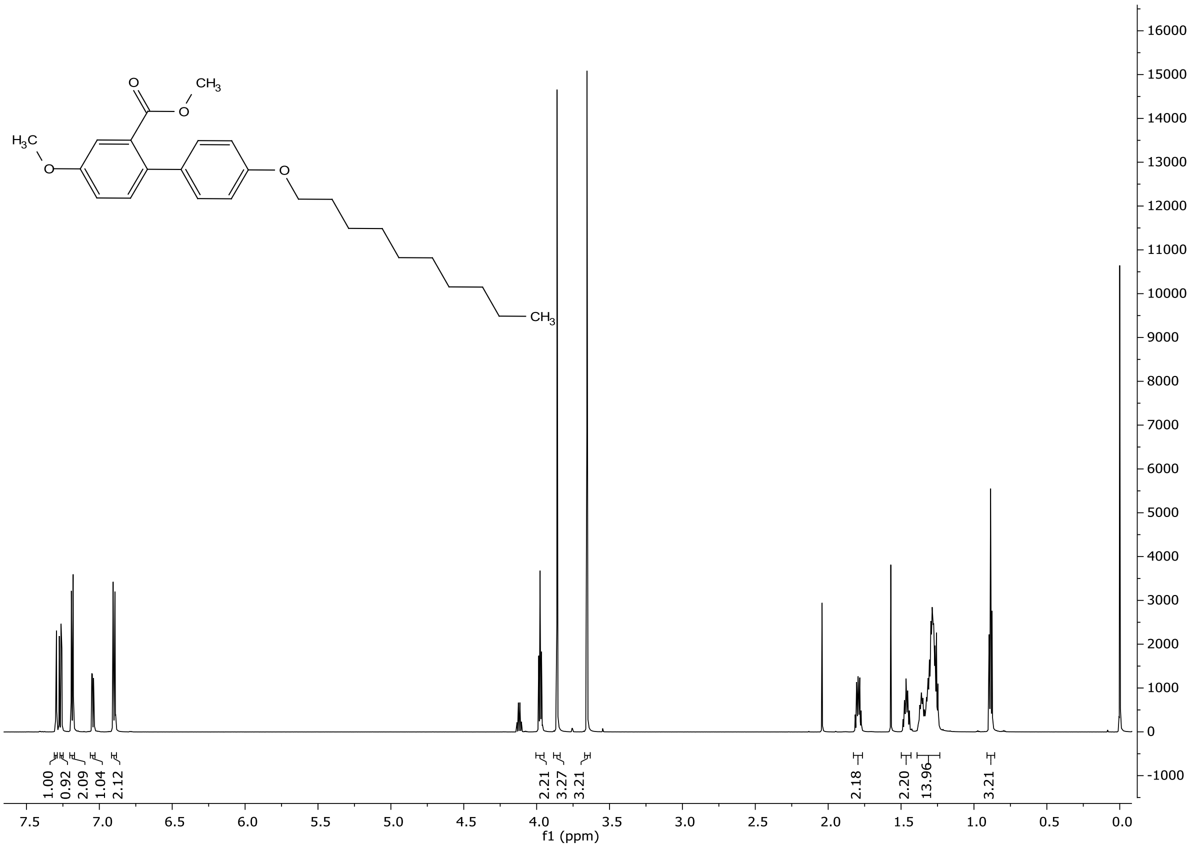

1.00
0.92
2.09
1.04
2.12
2.21
3.27
3.21
2.18
2.20
13.96
3.21
f1 (ppm)

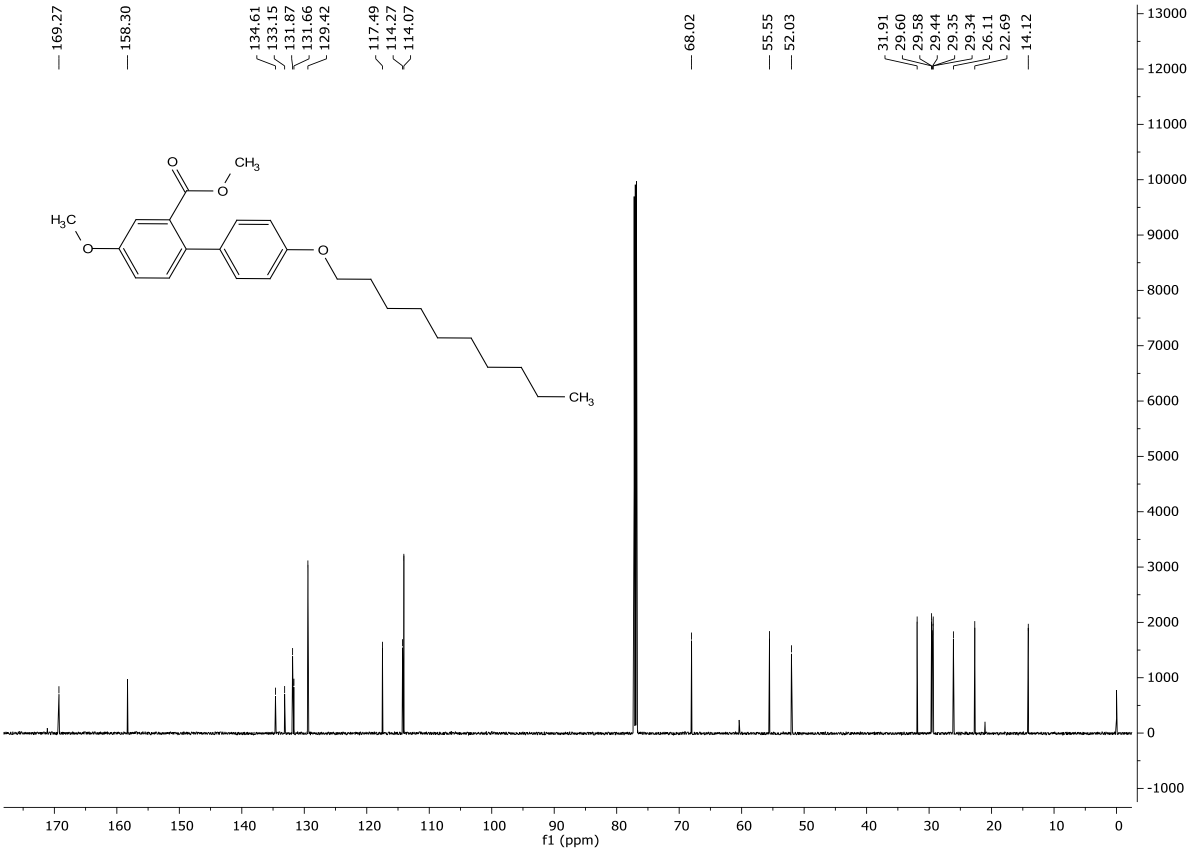

169.27
158.30
134.61
133.15
131.87
131.66
129.42
117.49
114.27
114.07
68.02
55.55
52.03
31.91
29.60
29.58
29.44
29.35
29.34
26.11
22.69
14.12
f1 (ppm)

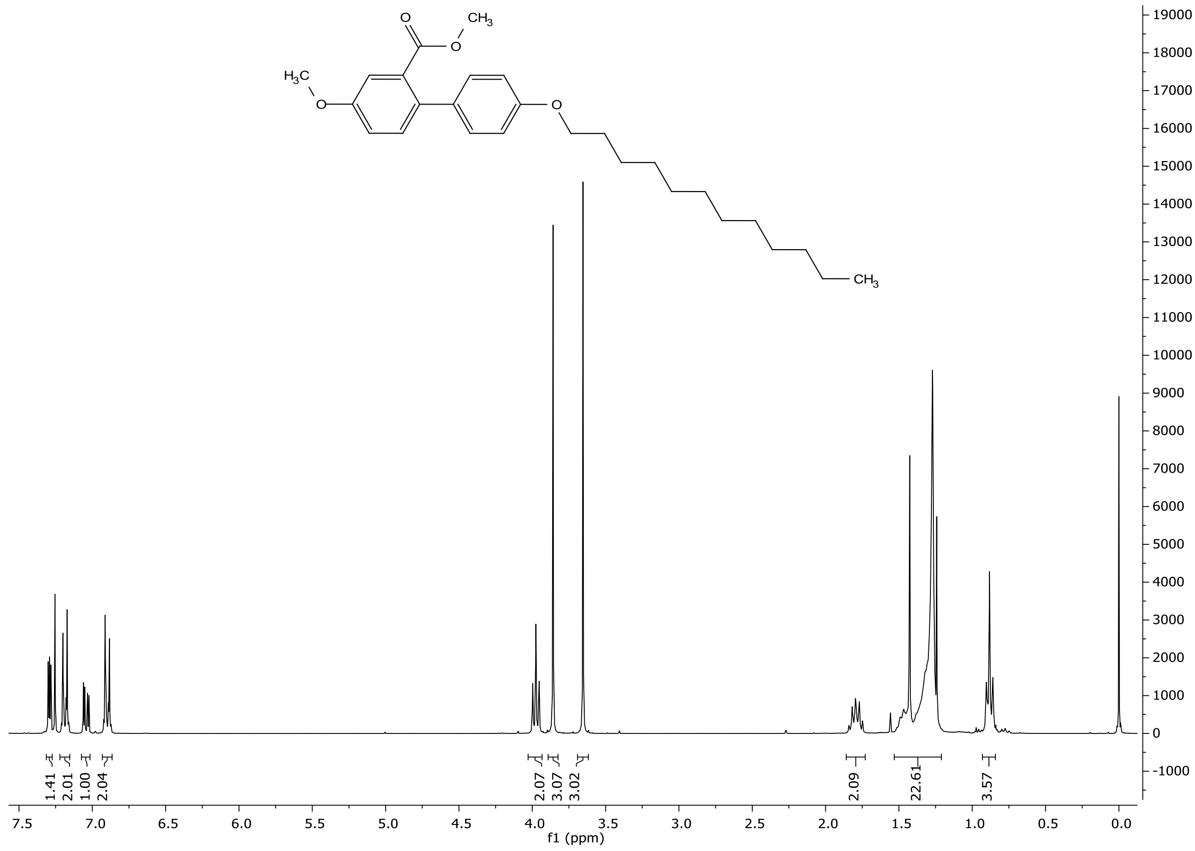

CH3
O
H3C
CH3
1.41
2.01
1.00
2.04
2.07
3.07
3.02
2.09
22.61
3.57
f1 (ppm)

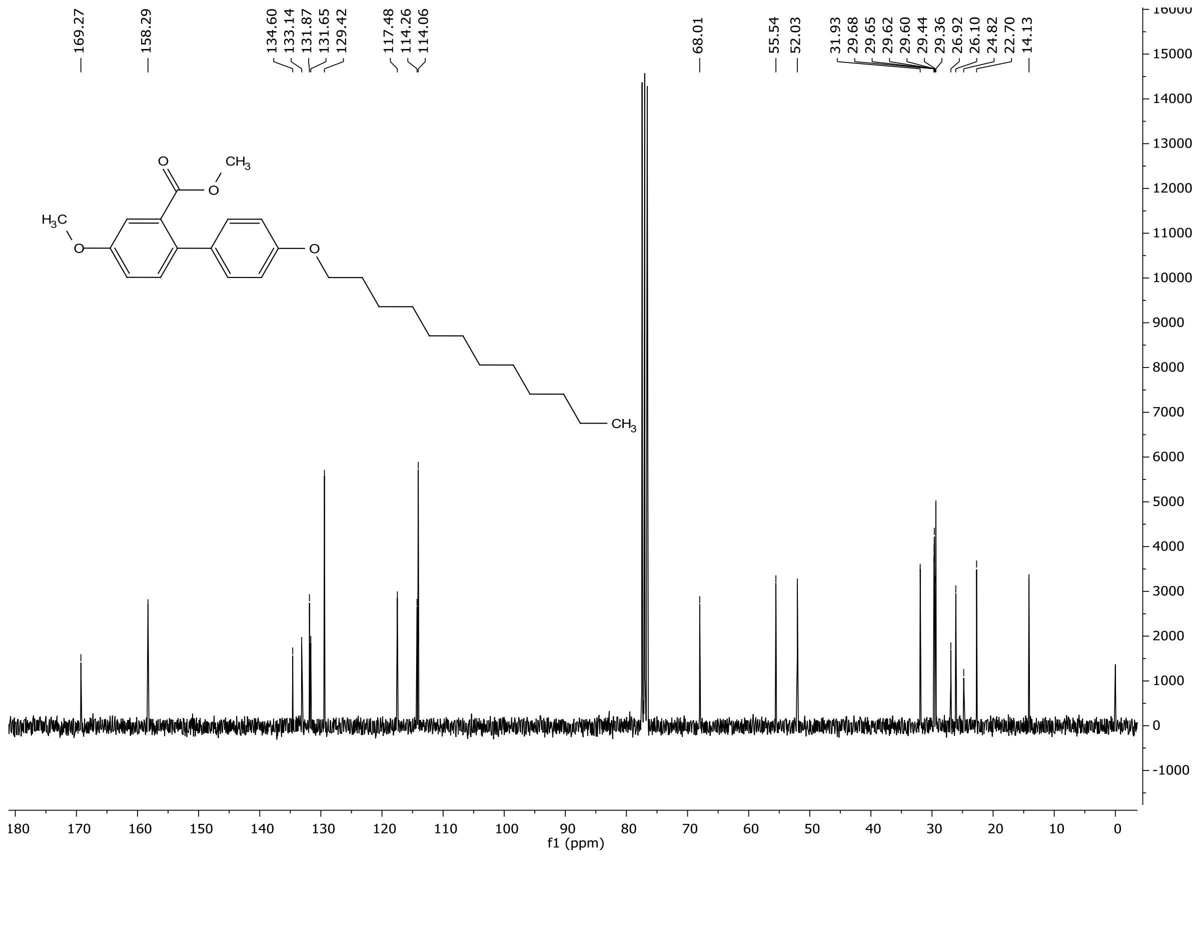

169.27
158.29
134.60
133.14
131.87
131.65
129.42
117.48
114.26
114.06
68.01
55.54
52.03
31.93
29.68
29.65
29.62
29.60
29.44
29.36
26.92
26.10
24.82
22.70
14.13
CH3
O
H3C
CH3
f1 (ppm)

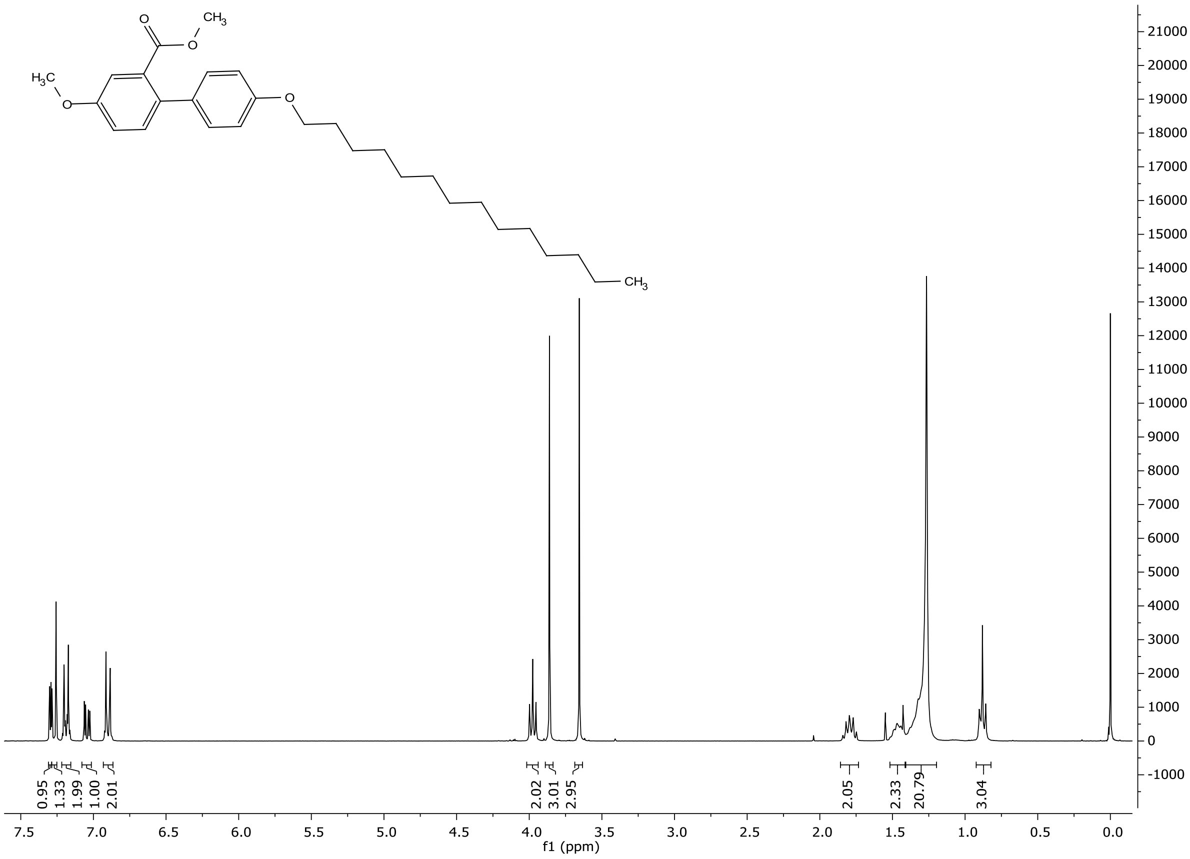

O
CH_3
H_3C
O
O
O
CH_3
0.95
1.33
1.99
1.00
2.01
2.02
3.01
2.95
2.05
2.33
20.79
3.04
f1 (ppm)

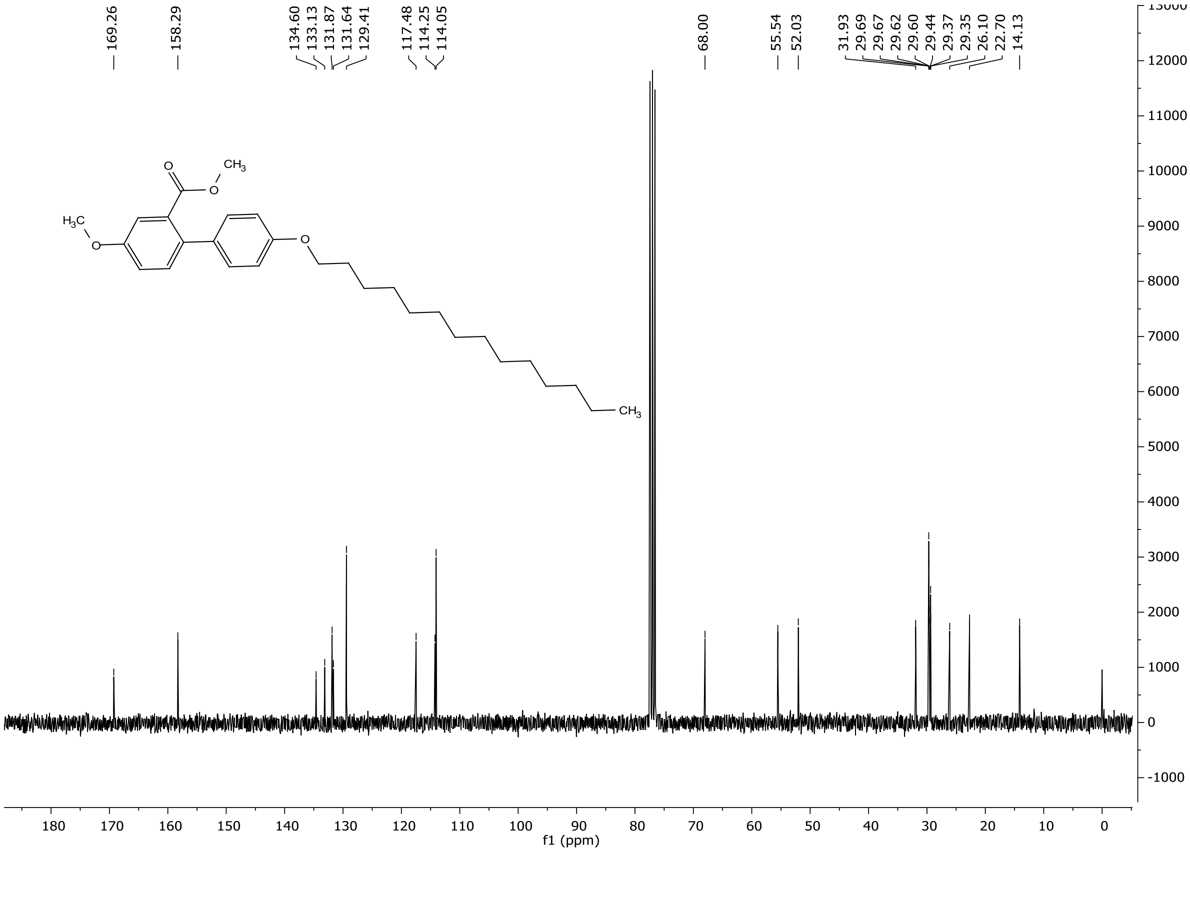

169.26
158.29
134.60
133.13
131.87
131.64
129.41
117.48
114.25
114.05
68.00
55.54
52.03
31.93
29.69
29.67
29.62
29.60
29.44
29.37
29.35
26.10
22.70
14.13
O
CH_3
H_3C
O
O
O
CH_3
f1 (ppm)

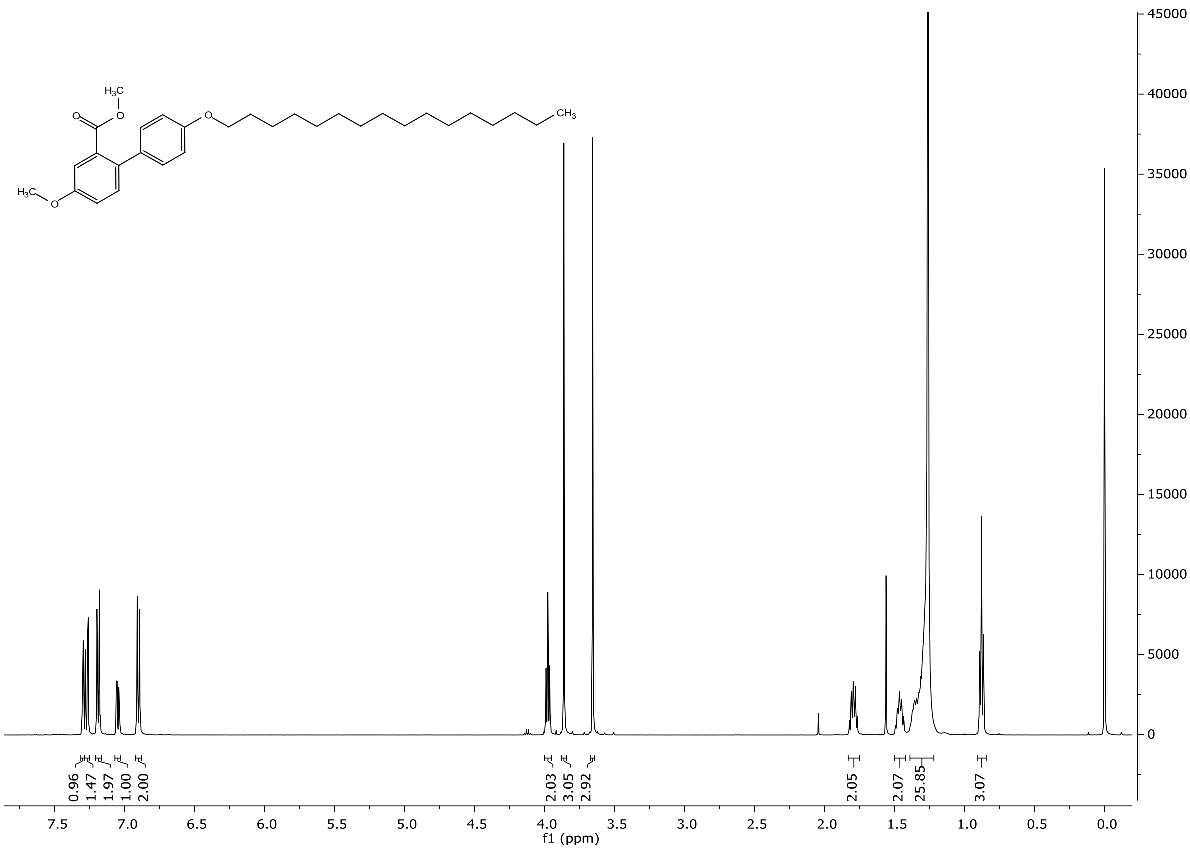
H3C
O
O
O
CH3
H3C
O
45000
40000
35000
30000
25000
20000
15000
10000
5000
0
0.96
1.47
1.97
1.00
2.00
2.03
3.05
2.92
2.05
2.07
25.85
3.07
7.5
7.0
6.5
6.0
5.5
5.0
4.5
4.0
3.5
3.0
2.5
2.0
1.5
1.0
0.5
0.0
f1 (ppm)

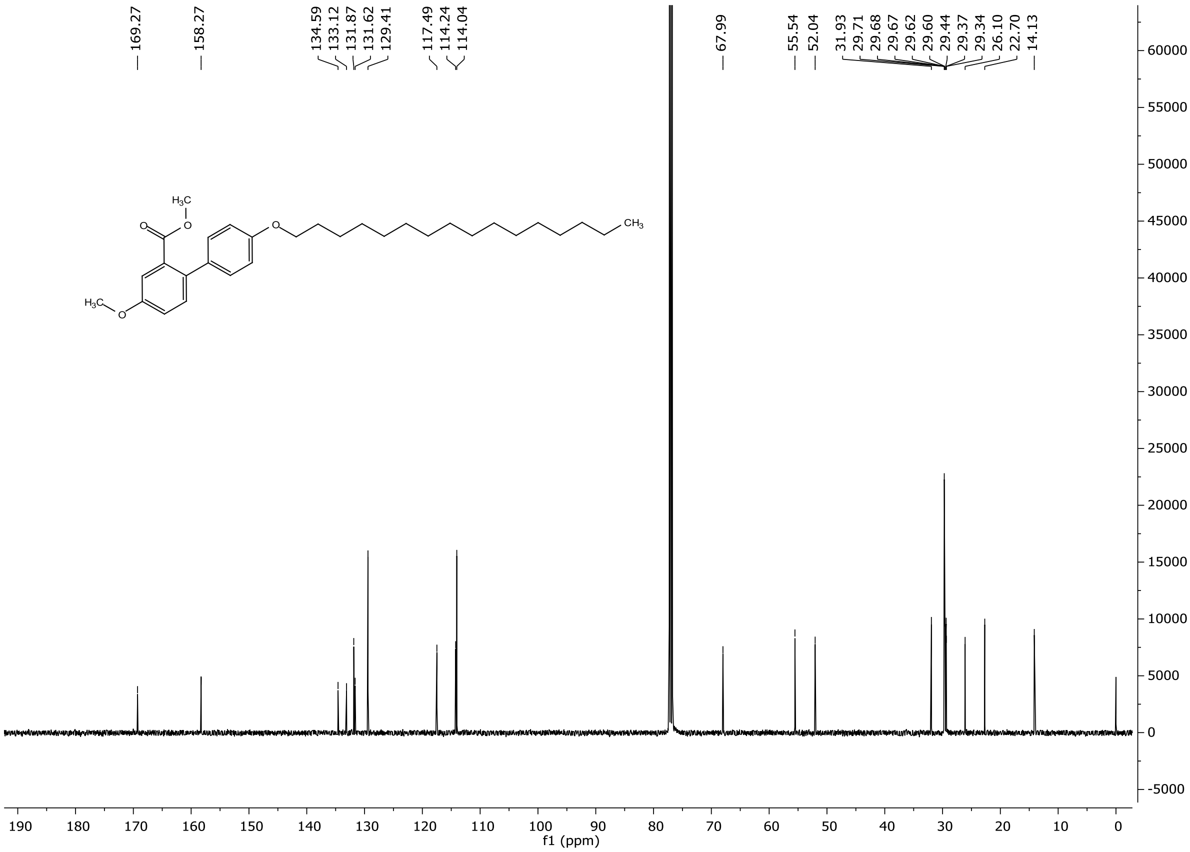
169.27
158.27
134.59
133.12
131.87
131.62
129.41
117.49
114.24
114.04
67.99
55.54
52.04
31.93
29.71
29.68
29.67
29.62
29.60
29.44
29.37
29.34
26.10
22.70
14.13
60000
55000
50000
45000
40000
35000
30000
25000
20000
15000
10000
5000
0
-5000
190
180
170
160
150
140
130
120
110
100
90
80
70
60
50
40
30
20
10
0
f1 (ppm)

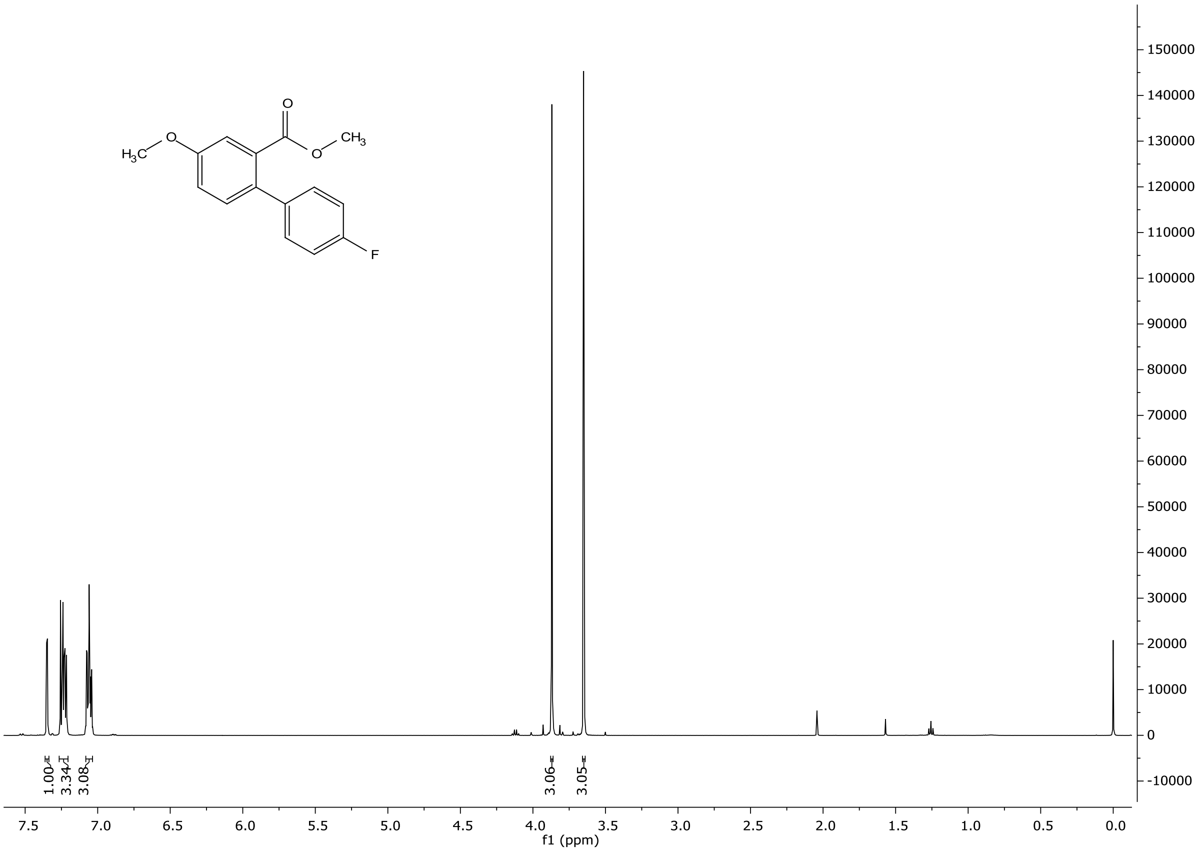

H3C O O CH3 O F
1.00
3.34
3.08
3.06
3.05
7.5 7.0 6.5 6.0 5.5 5.0 4.5 4.0 3.5 3.0 2.5 2.0 1.5 1.0 0.5 0.0
f1 (ppm)
150000 140000 130000 120000 110000 100000 90000 80000 70000 60000 50000 40000 30000 20000 10000 0 -10000

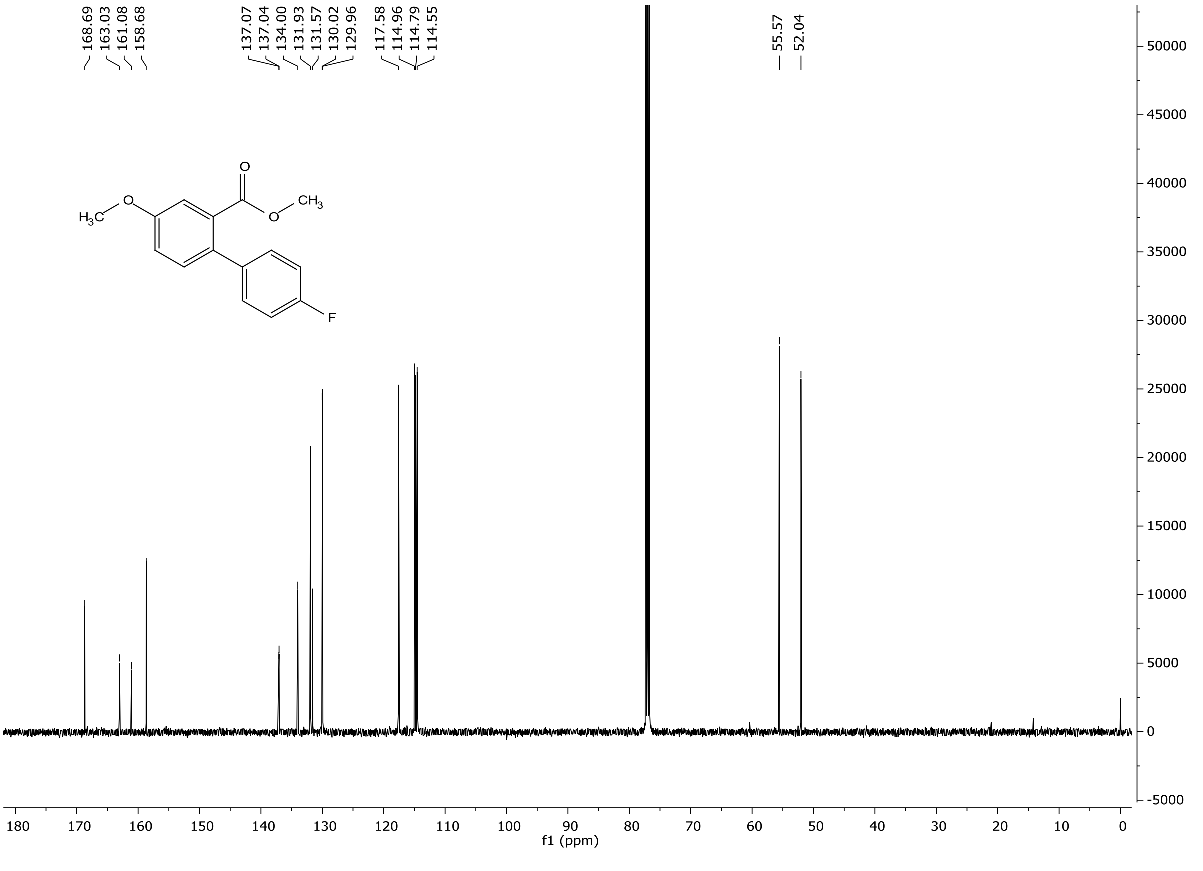

168.69
163.03
161.08
158.68
137.07
137.04
134.00
131.93
131.57
130.02
129.96
117.58
114.96
114.79
114.55
55.57
52.04
H3C O O CH3 O F
180 170 160 150 140 130 120 110 100 90 80 70 60 50 40 30 20 10 0
f1 (ppm)
50000 45000 40000 35000 30000 25000 20000 15000 10000 5000 0 -5000

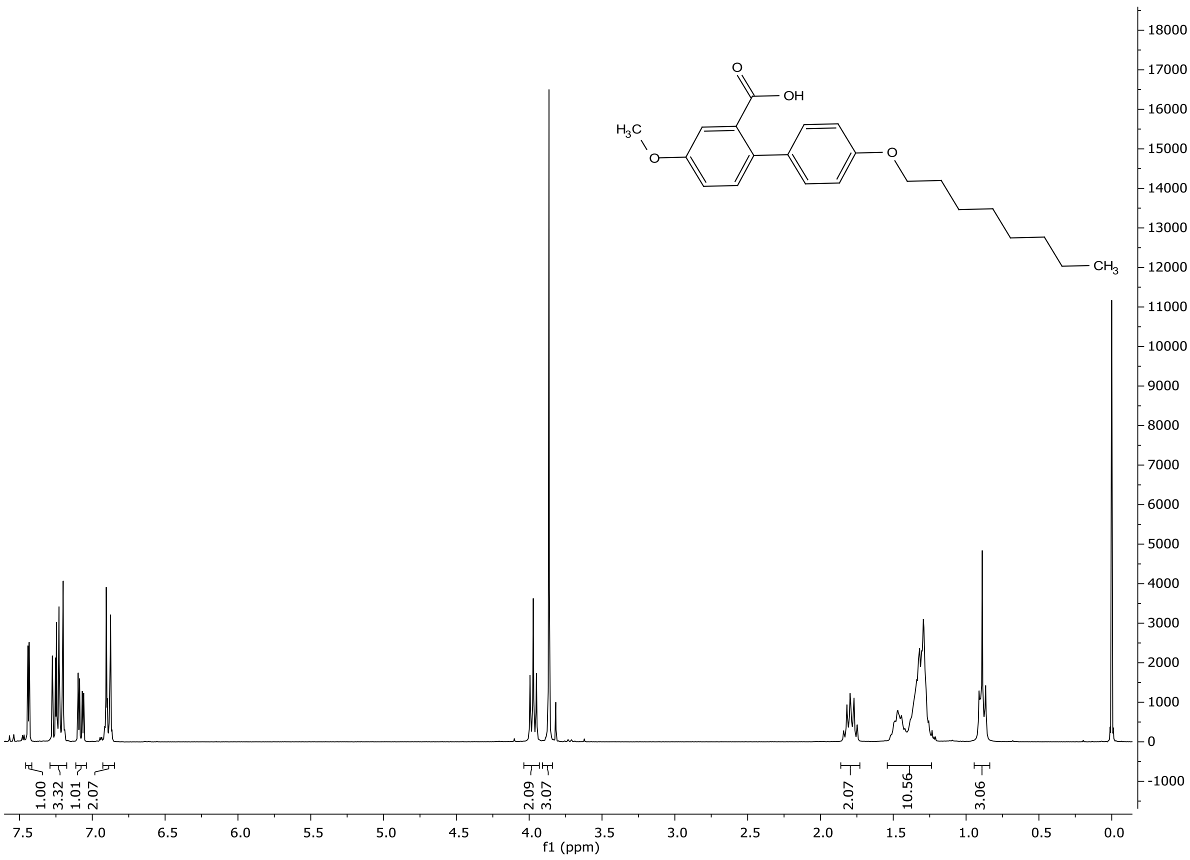

O
OH
H_3C
O
O
CH_3
1.00
3.32
1.01
2.07
2.09
3.07
2.07
10.56
3.06
f1 (ppm)

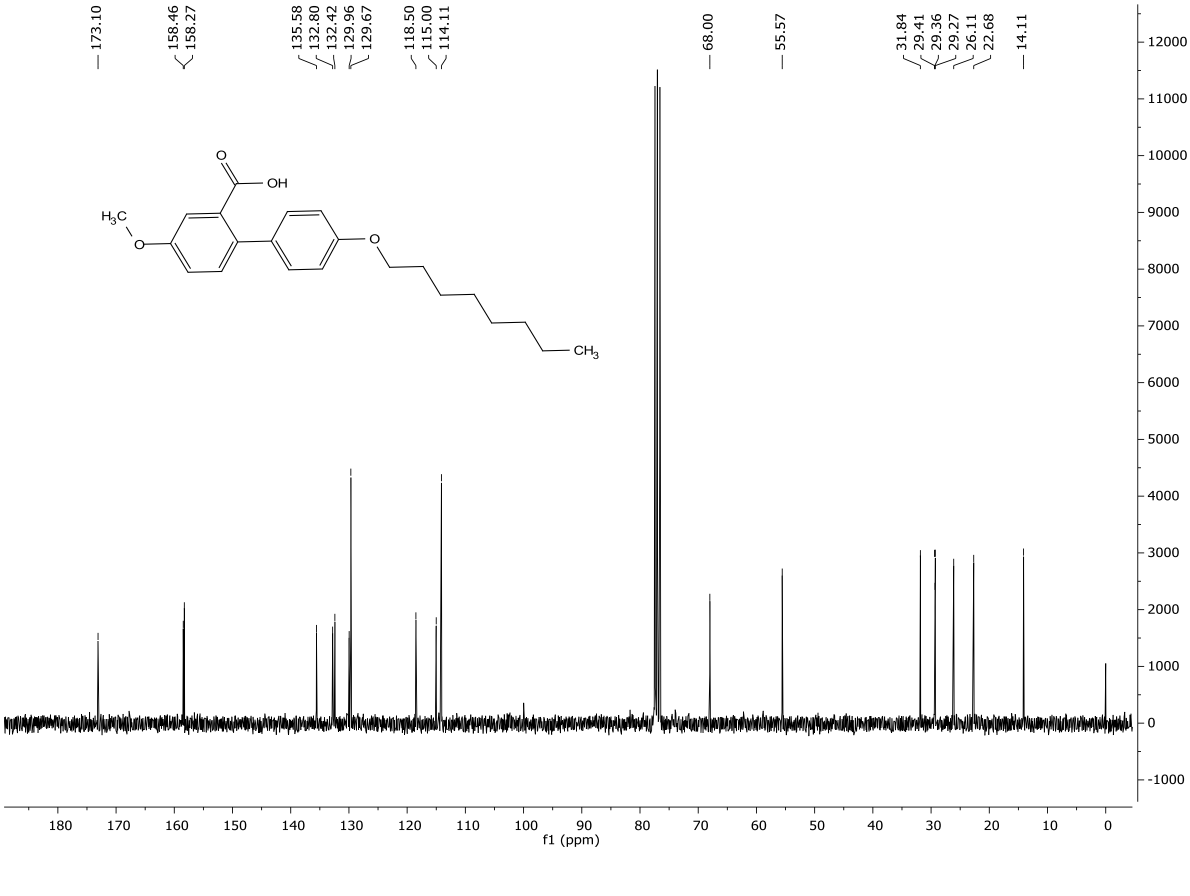

173.10
158.46
158.27
135.58
132.80
132.42
129.96
129.67
118.50
115.00
114.11
68.00
55.57
31.84
29.41
29.36
29.27
26.11
22.68
14.11
O
OH
H_3C
O
O
CH_3
f1 (ppm)

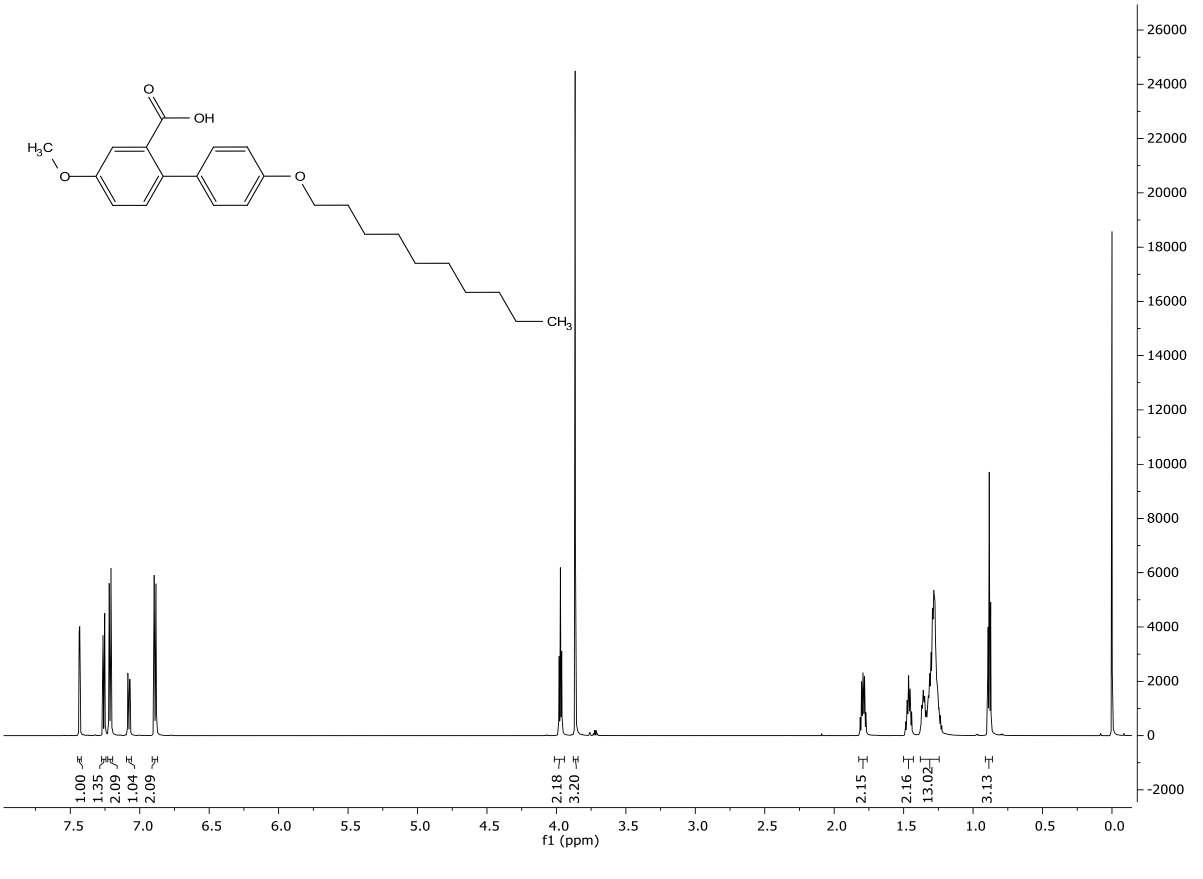

OH
O
H3C
O
CH3
1.00
1.35
2.09
1.04
2.09
2.18
3.20
2.15
2.16
13.02
3.13
f1 (ppm)

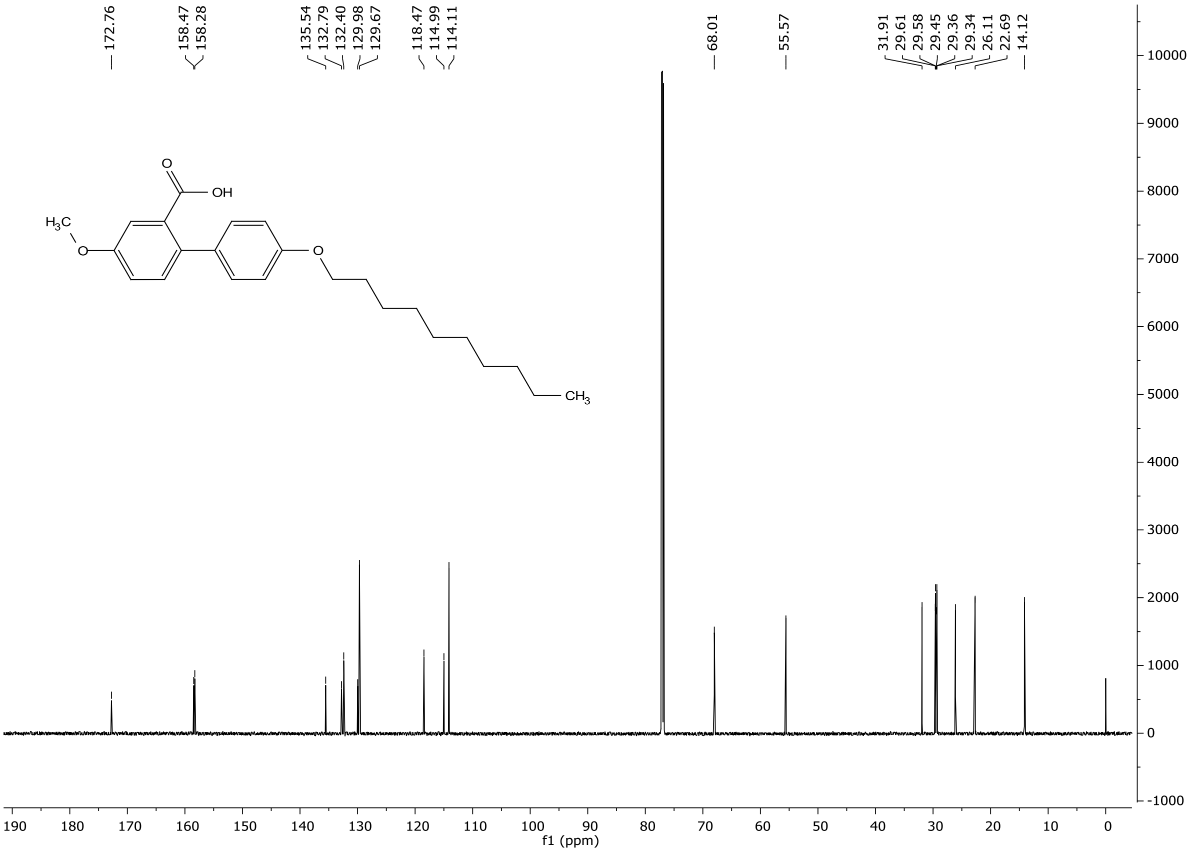

172.76
158.47
158.28
135.54
132.79
132.40
129.98
129.67
118.47
114.99
114.11
68.01
55.57
31.91
29.61
29.58
29.45
29.36
29.34
26.11
22.69
14.12
OH
O
H3C
O
CH3
f1 (ppm)

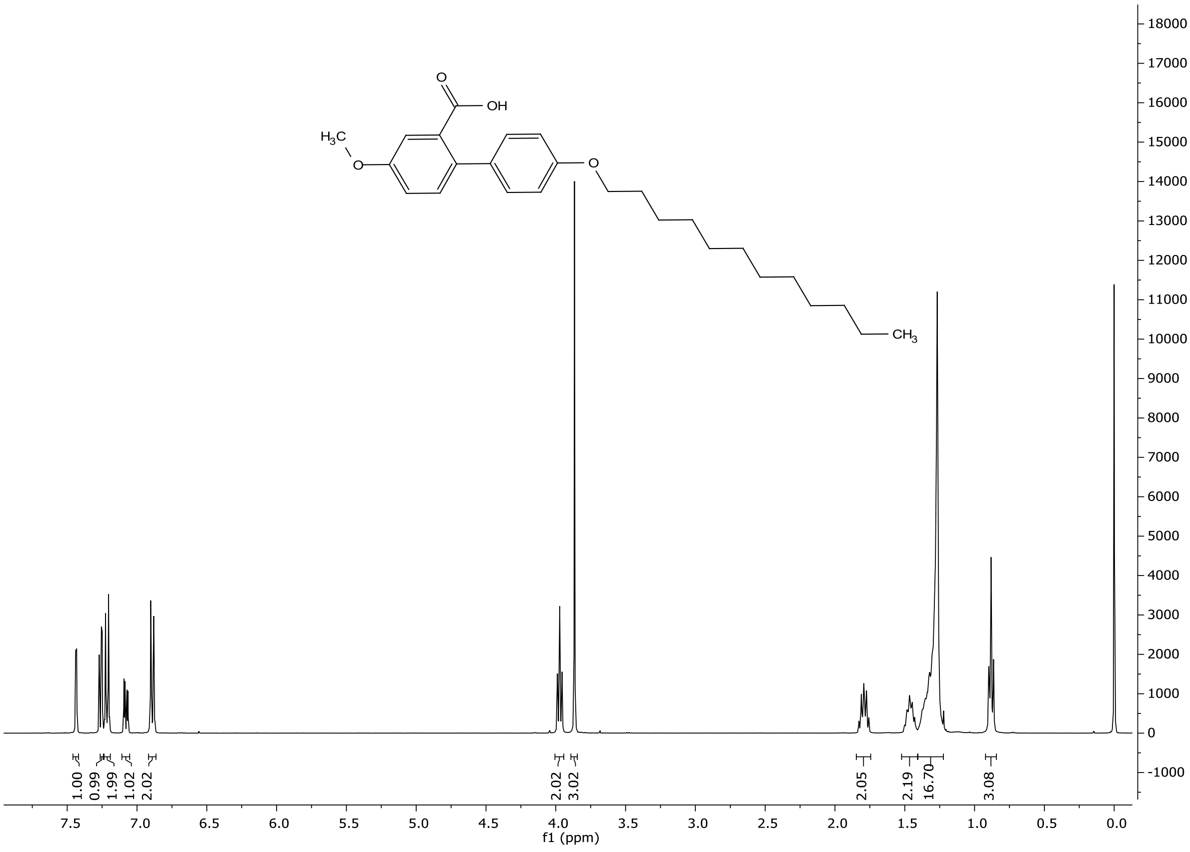

O
OH
H_3C
O
O
CH_3
1.00
0.99
1.99
1.02
2.02
2.02
3.02
2.05
2.19
16.70
3.08
f1 (ppm)

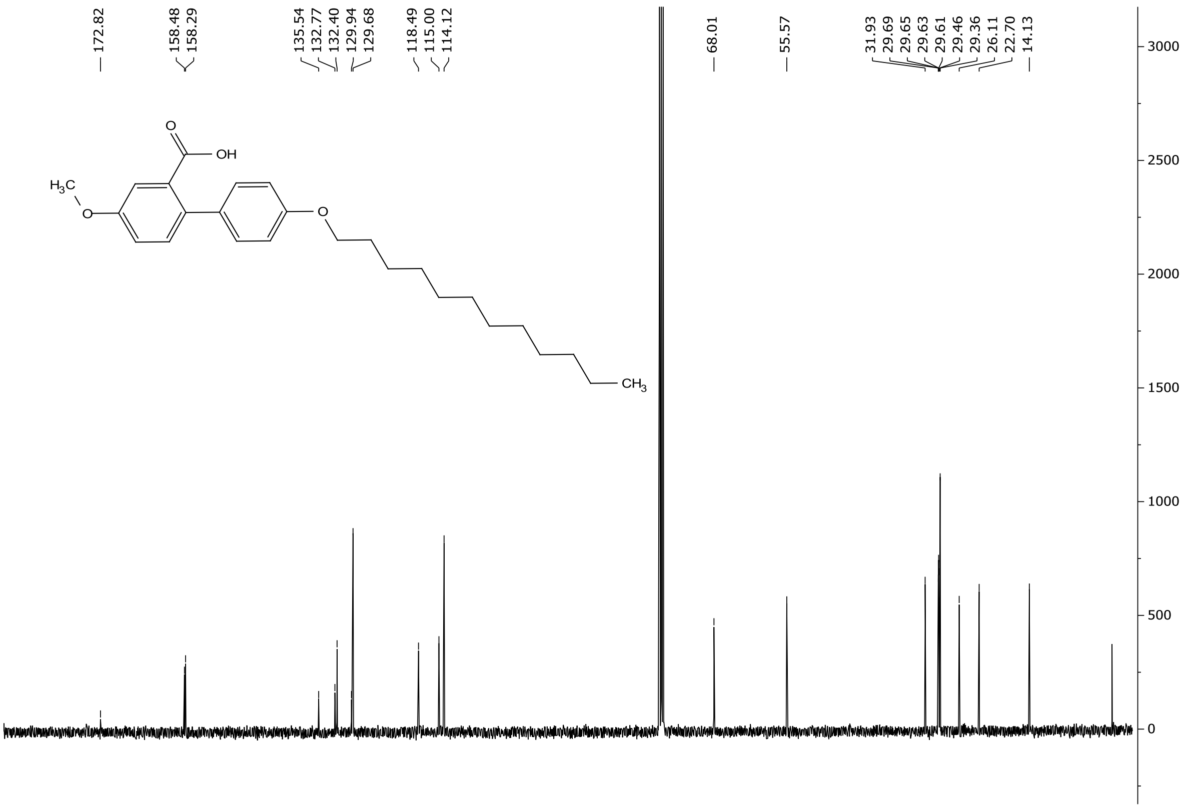

172.82
158.48
158.29
135.54
132.77
132.40
129.94
129.68
118.49
115.00
114.12
68.01
55.57
31.93
29.69
29.65
29.63
29.61
29.46
29.36
26.11
22.70
14.13
O
OH
H_3C
O
O
CH_3
f1 (ppm)

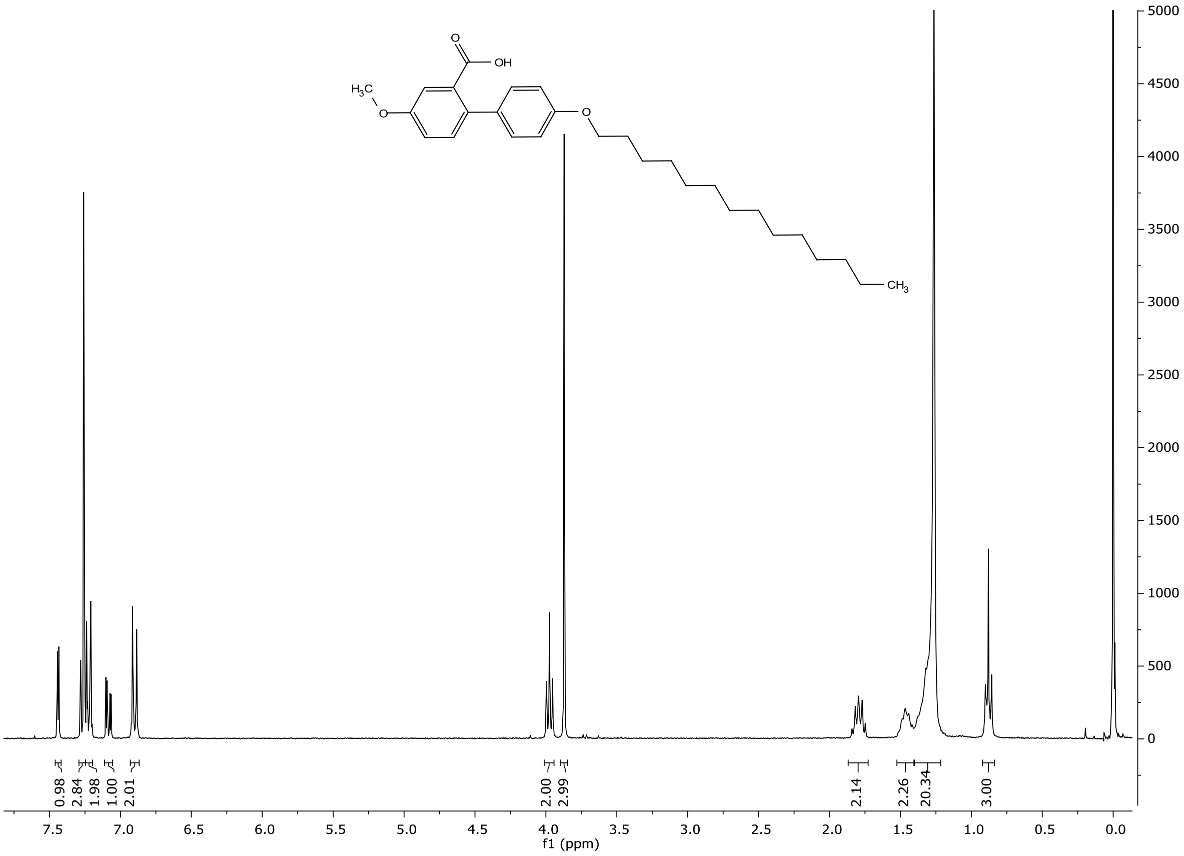
H3C
O
OH
O
CH3
0.98
2.84
1.98
1.00
2.01
2.00
2.99
2.14
2.26
20.34
3.00
f1 (ppm)

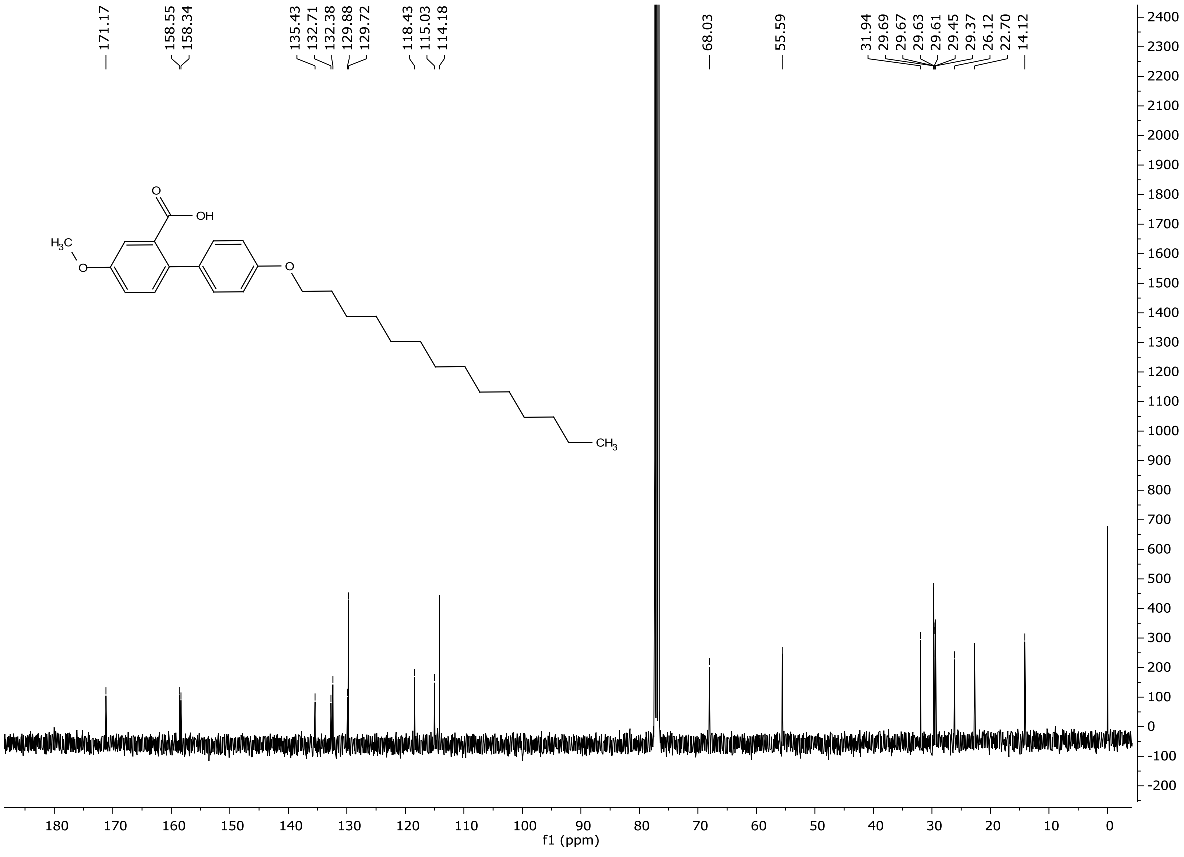
171.17
158.55
158.34
135.43
132.71
132.38
129.88
129.72
118.43
115.03
114.18
68.03
55.59
31.94
29.69
29.67
29.63
29.61
29.45
29.37
26.12
22.70
14.12
H3C
O
OH
O
CH3
f1 (ppm)

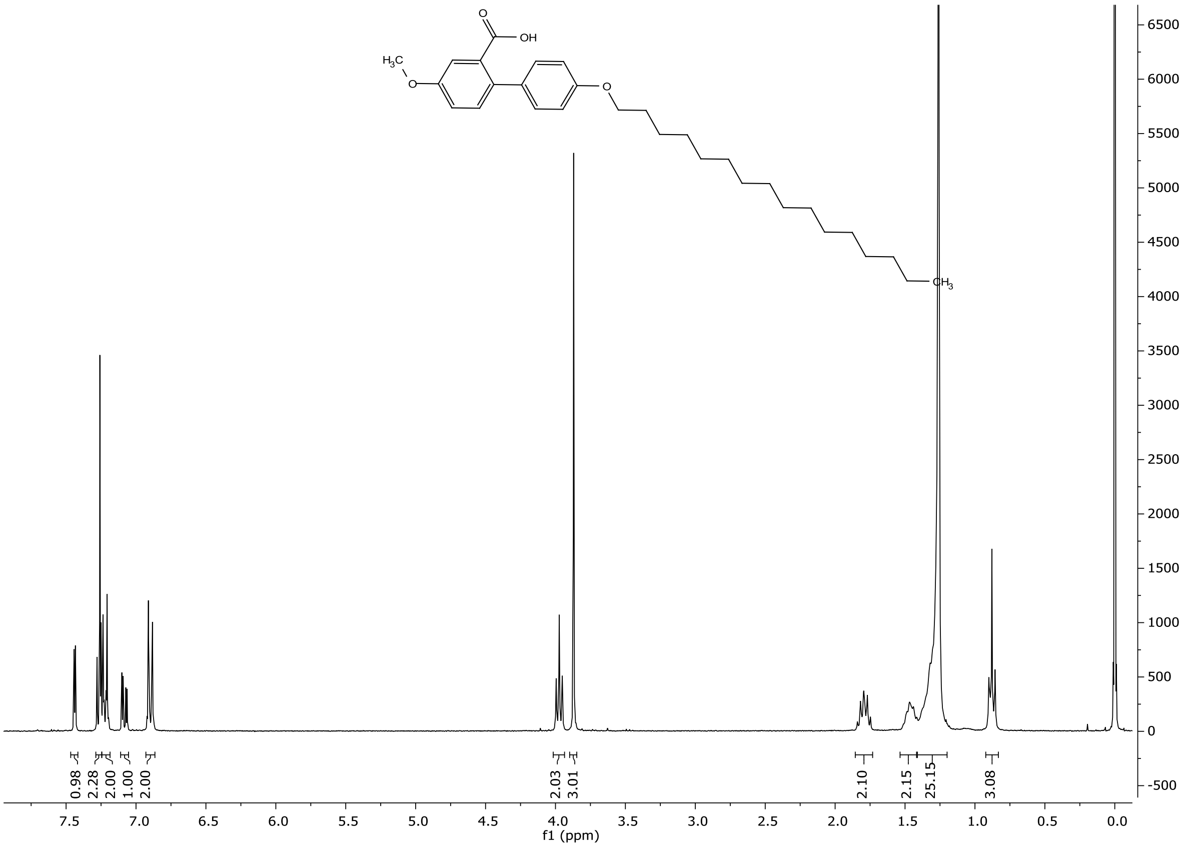

OH
O
H3C
O
O
CH3
0.98
2.28
2.00
1.00
2.00
2.03
3.01
2.10
2.15
25.15
3.08
f1 (ppm)

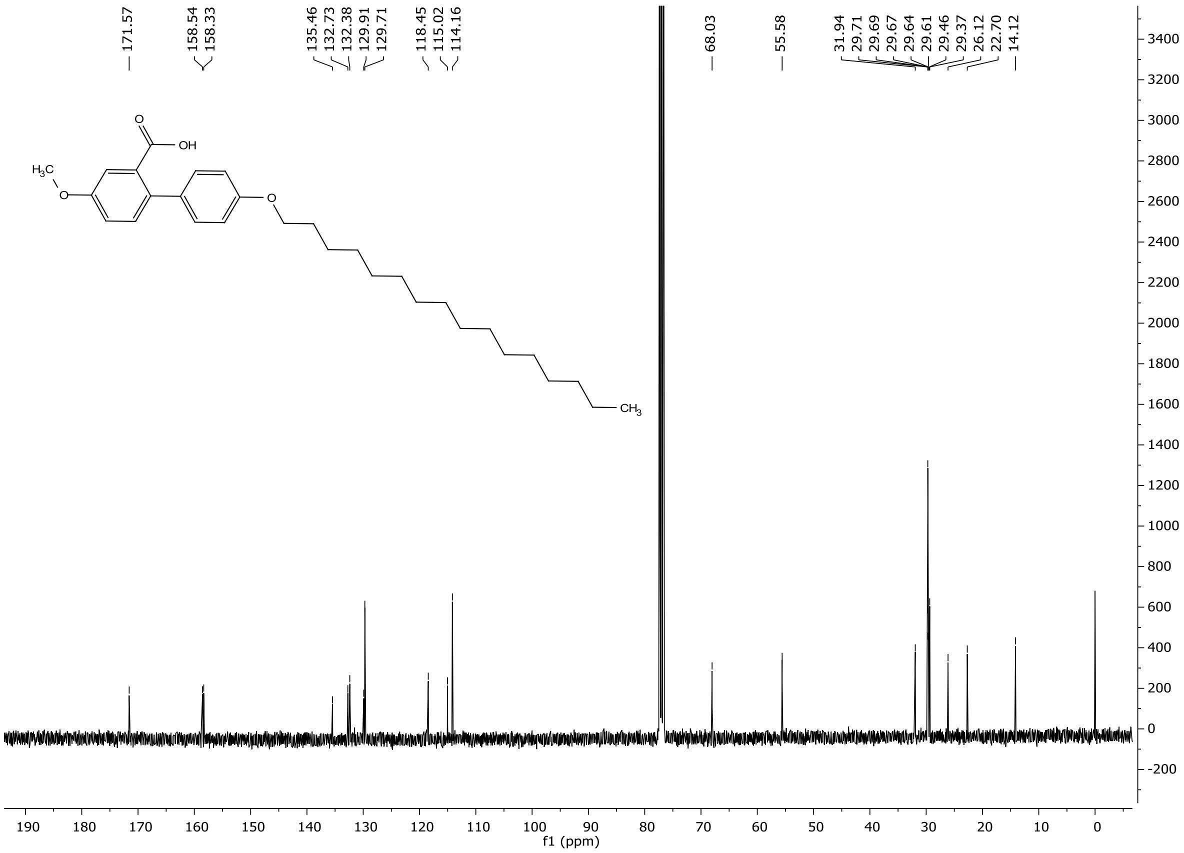

171.57
158.54
158.33
135.46
132.73
132.38
129.91
129.71
118.45
115.02
114.16
68.03
55.58
31.94
29.71
29.69
29.67
29.64
29.61
29.46
29.37
26.12
22.70
14.12
OH
O
H3C
O
O
CH3
f1 (ppm)

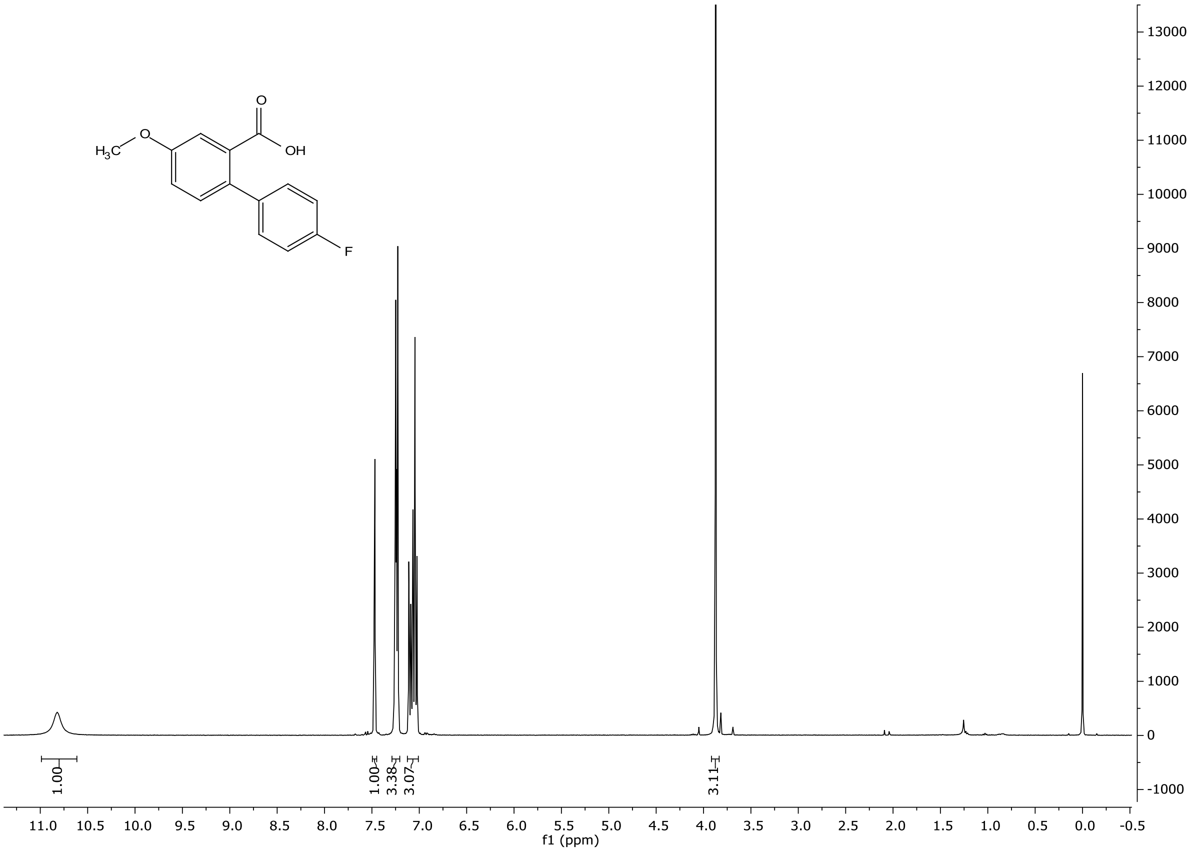
H3C O OH F
1.00
1.00
3.38
3.07
3.11
f1 (ppm)
11.0 10.5 10.0 9.5 9.0 8.5 8.0 7.5 7.0 6.5 6.0 5.5 5.0 4.5 4.0 3.5 3.0 2.5 2.0 1.5 1.0 0.5 0.0 -0.5
13000 12000 11000 10000 9000 8000 7000 6000 5000 4000 3000 2000 1000 0 -1000

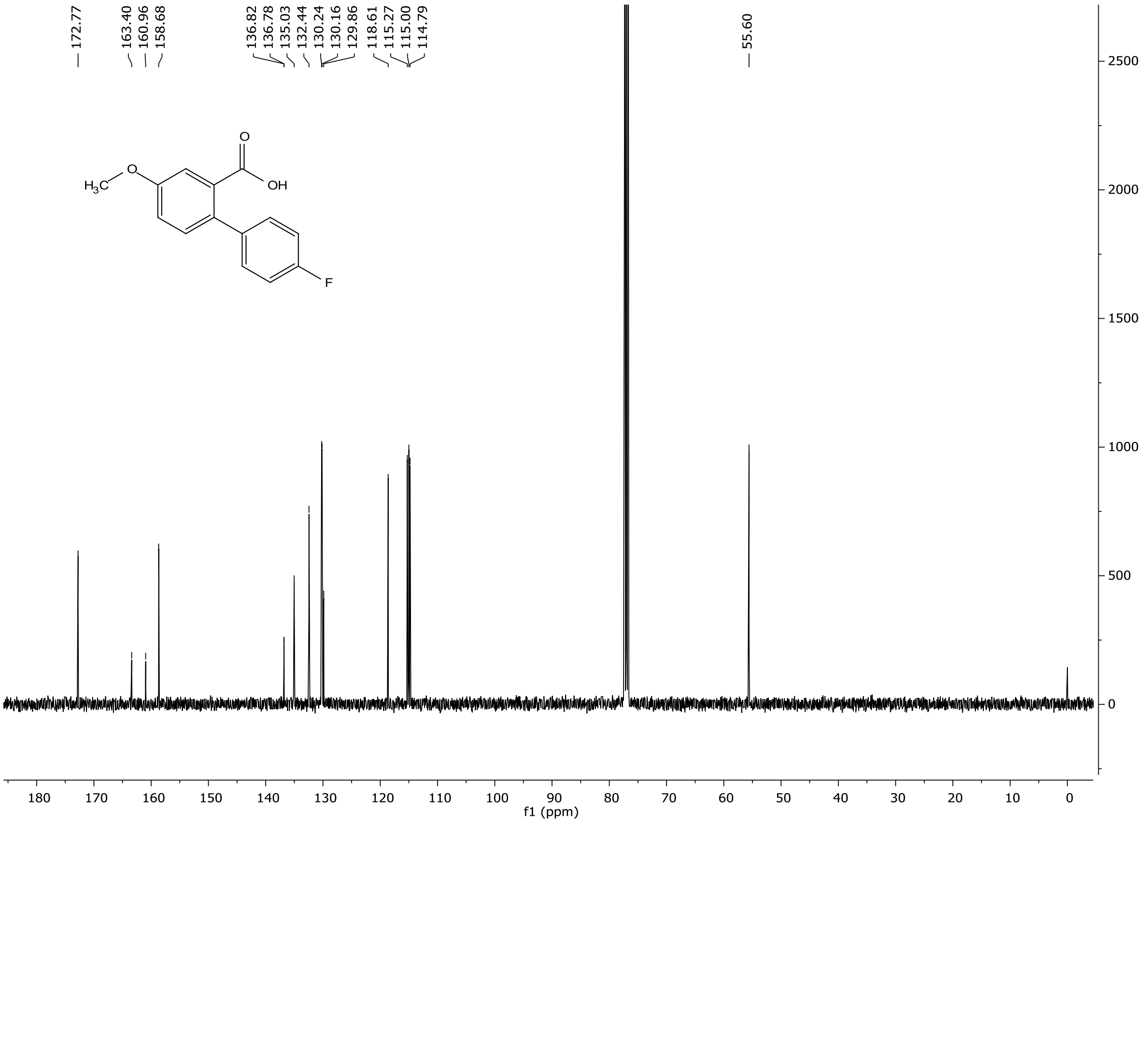
172.77
163.40
160.96
158.68
136.82
136.78
135.03
132.44
130.24
130.16
129.86
118.61
115.27
115.00
114.79
55.60
H3C O OH F
f1 (ppm)
180 170 160 150 140 130 120 110 100 90 80 70 60 50 40 30 20 10 0
2500 2000 1500 1000 500 0

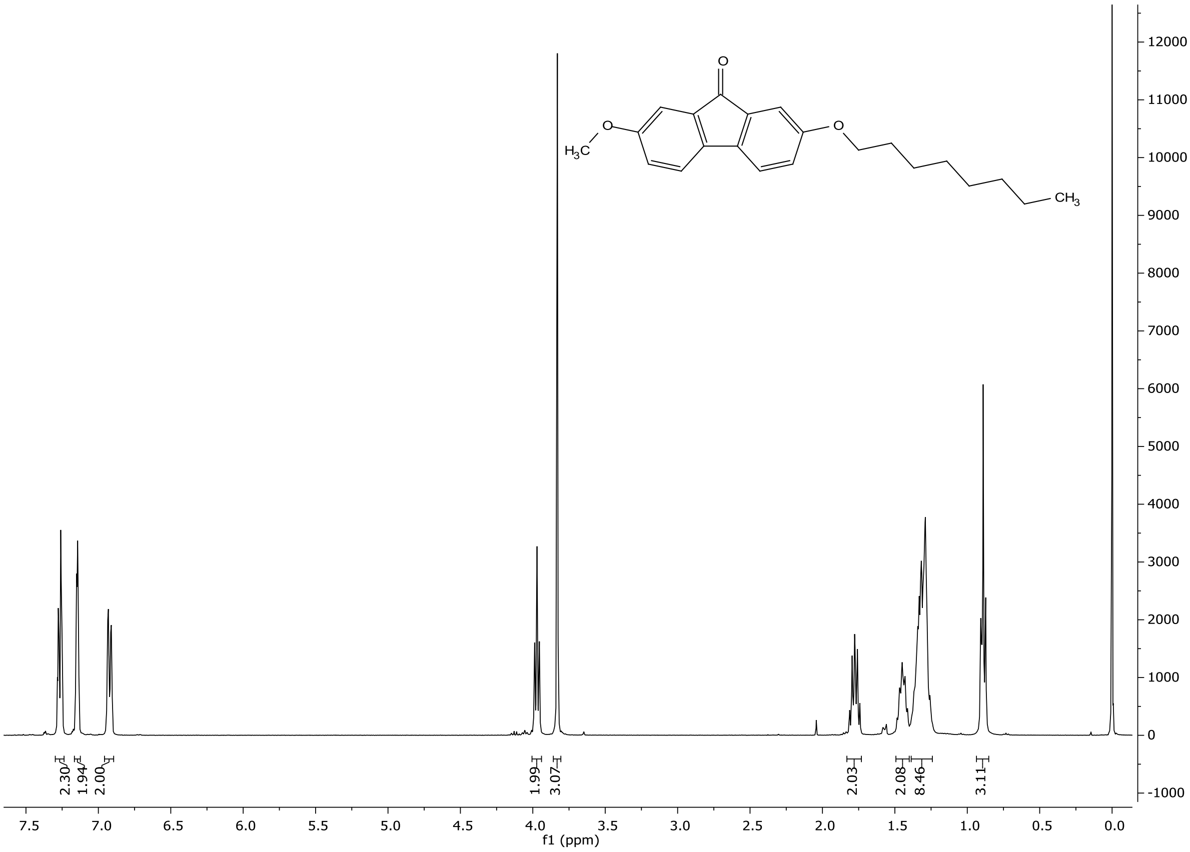
O
H3C
O
O
CH3
2.30
1.94
2.00
1.99
3.07
2.03
2.08
8.46
3.11
f1 (ppm)

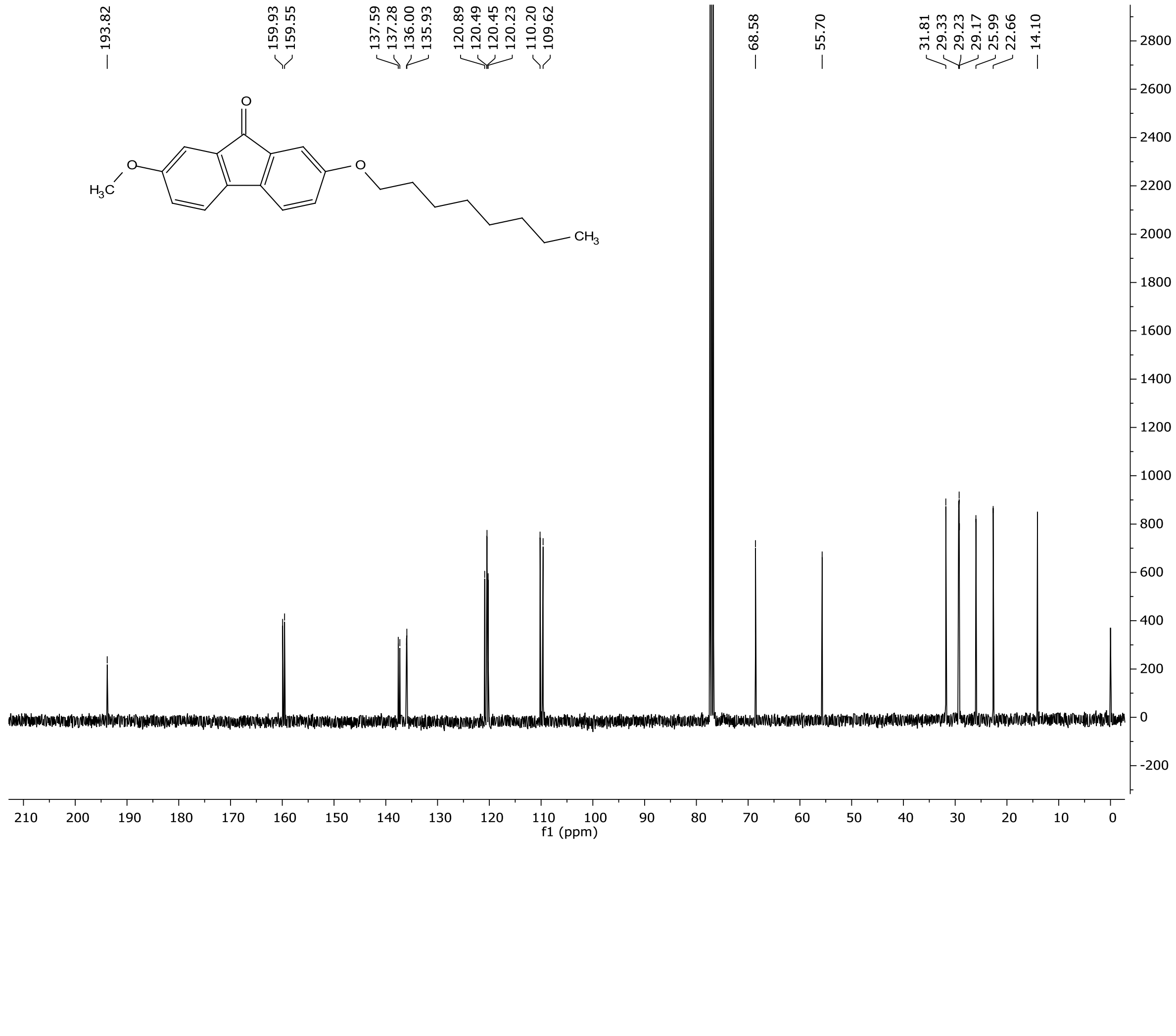
193.82
159.93
159.55
137.59
137.28
136.00
135.93
120.89
120.49
120.45
120.23
110.20
109.62
68.58
55.70
31.81
29.33
29.23
29.17
25.99
22.66
14.10
f1 (ppm)

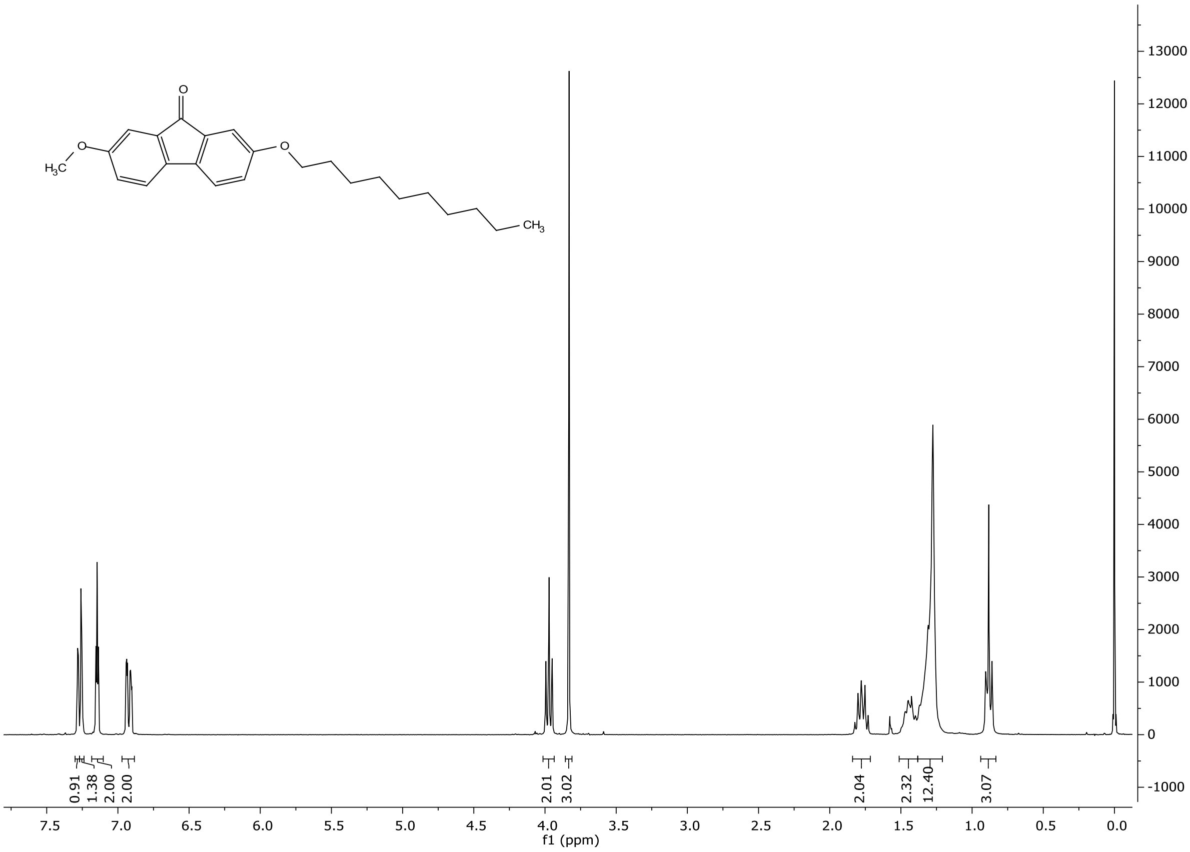

O
H_3C
O
O
CH_3
0.91
1.38
2.00
2.00
2.01
3.02
2.04
2.32
12.40
3.07
7.5 7.0 6.5 6.0 5.5 5.0 4.5 4.0 3.5 3.0 2.5 2.0 1.5 1.0 0.5 0.0
f1 (ppm)
13000 12000 11000 10000 9000 8000 7000 6000 5000 4000 3000 2000 1000 0 -1000

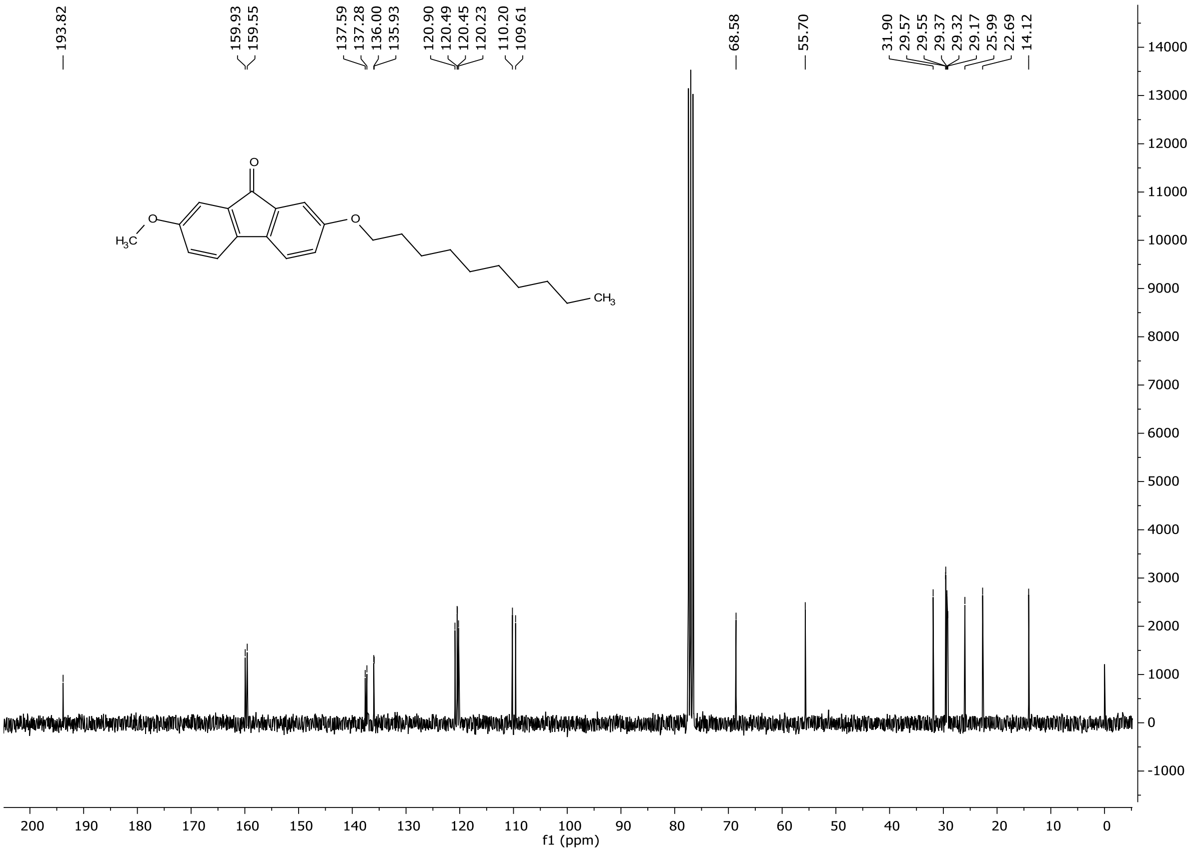

193.82
159.93
159.55
137.59
137.28
136.00
135.93
120.90
120.49
120.45
120.23
110.20
109.61
68.58
55.70
31.90
29.57
29.55
29.37
29.32
29.17
25.99
22.69
14.12
O
H_3C
O
O
CH_3
200 190 180 170 160 150 140 130 120 110 100 90 80 70 60 50 40 30 20 10 0
f1 (ppm)
14000 13000 12000 11000 10000 9000 8000 7000 6000 5000 4000 3000 2000 1000 0 -1000

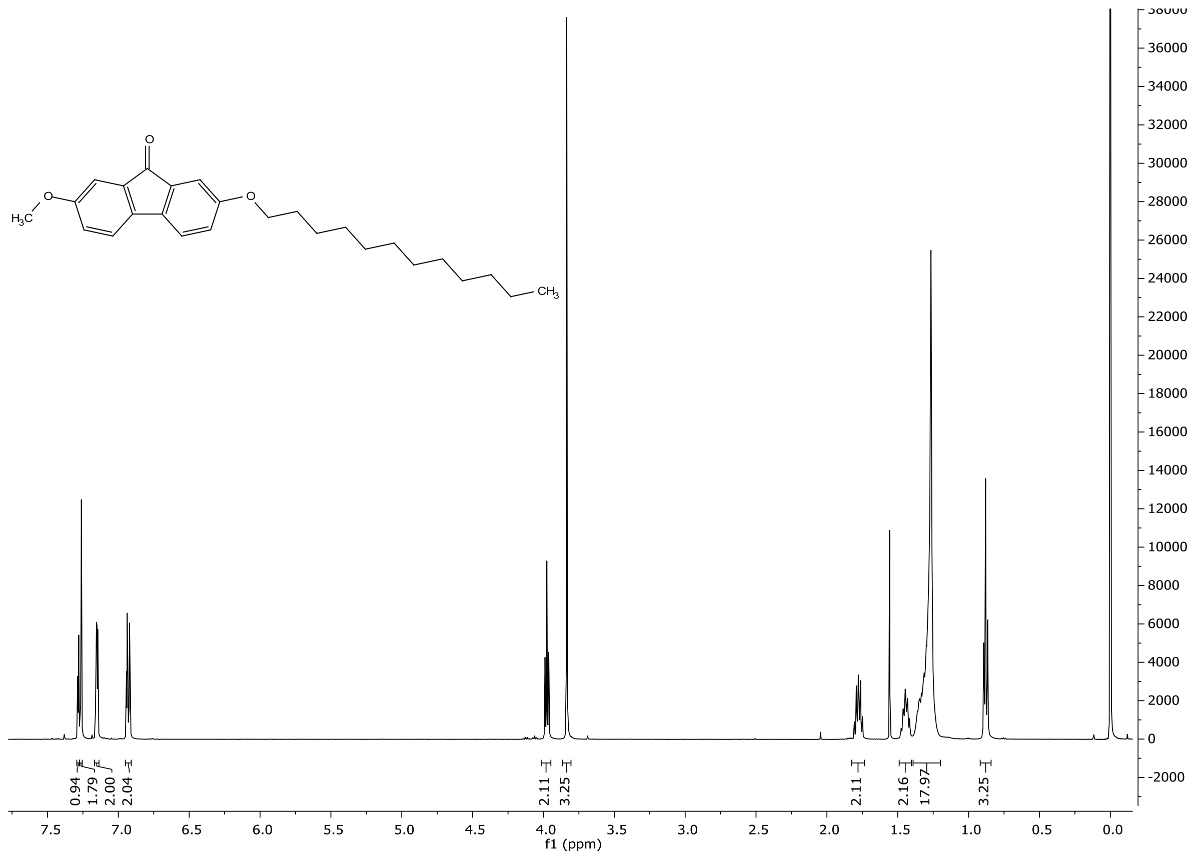

0.94
1.79
2.00
2.04
2.11
3.25
2.11
2.16
17.97
3.25
f1 (ppm)

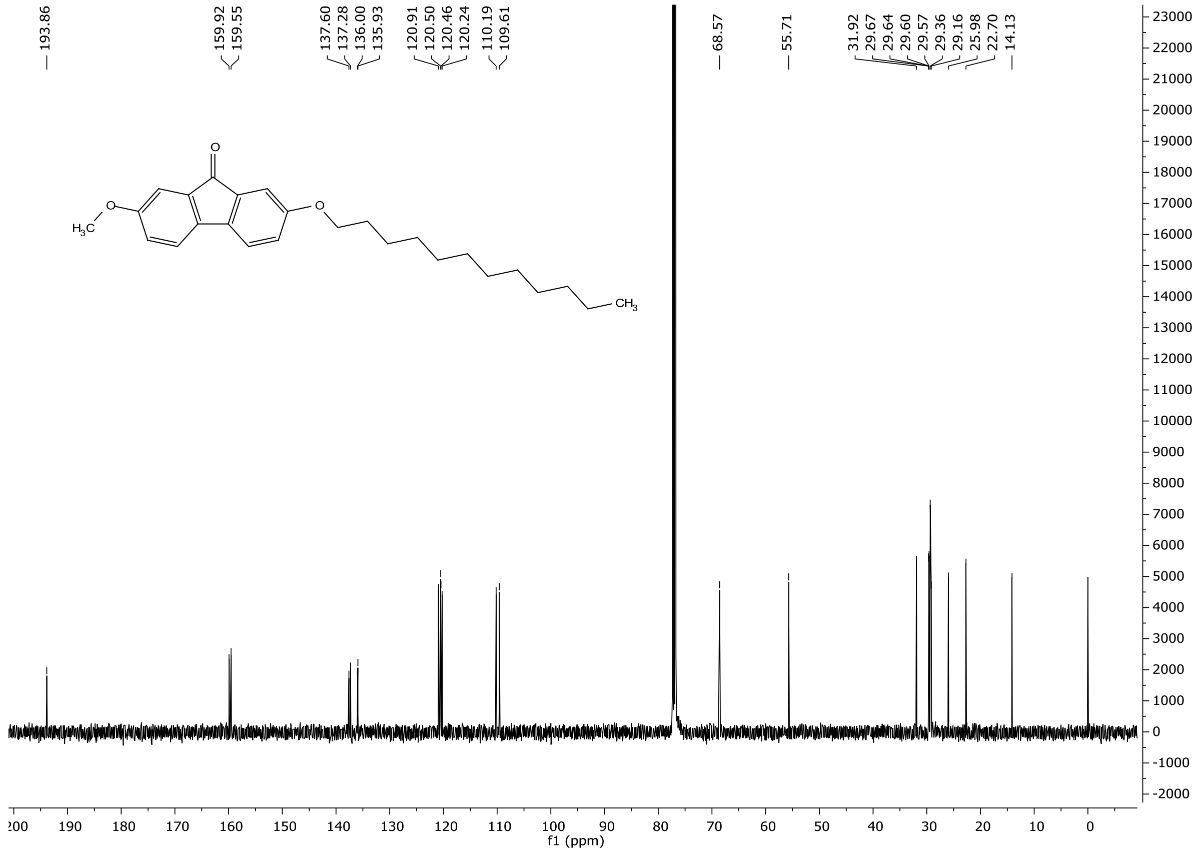

193.86
159.92
159.55
137.60
137.28
136.00
135.93
120.91
120.50
120.46
120.24
110.19
109.61
68.57
55.71
31.92
29.67
29.64
29.60
29.57
29.36
29.16
25.98
22.70
14.13
f1 (ppm)

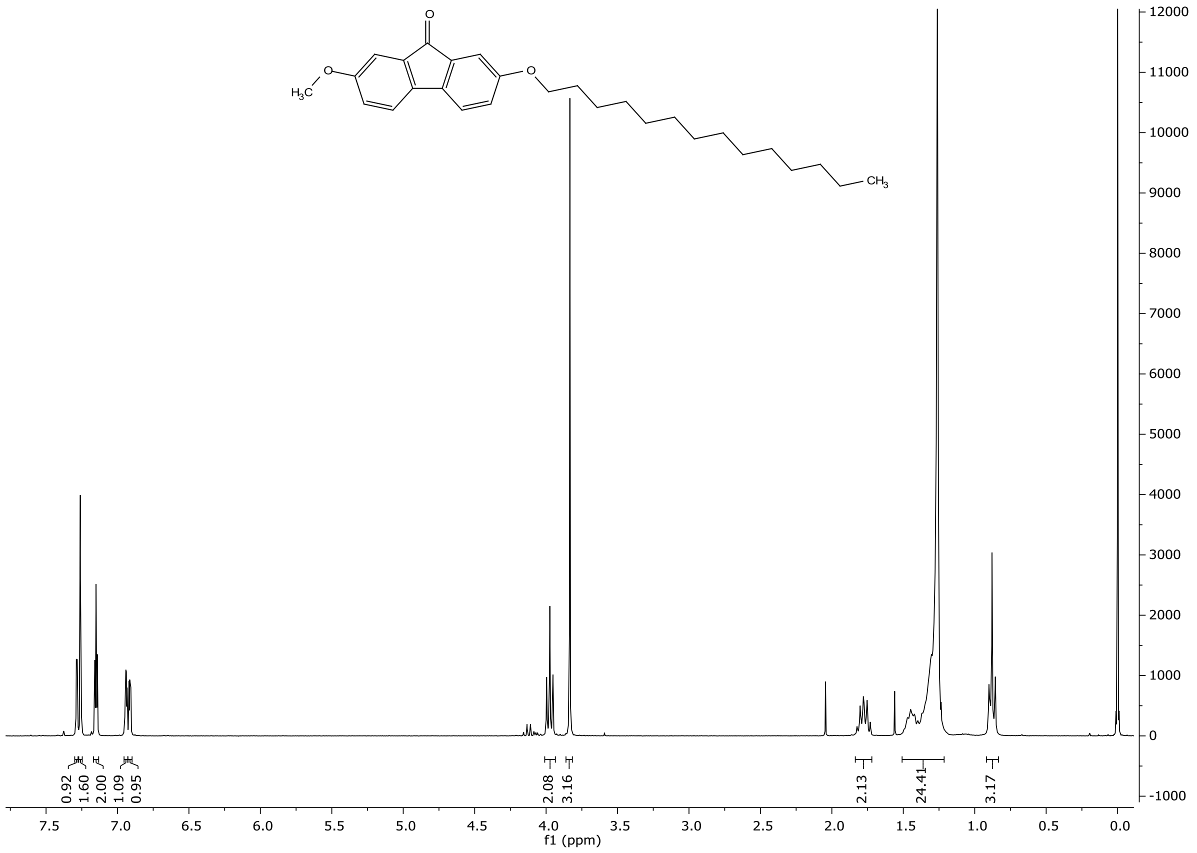

O
H_3C
O
O
CH_3
0.92
1.60
2.00
1.09
0.95
2.08
3.16
2.13
24.41
3.17
7.5
7.0
6.5
6.0
5.5
5.0
4.5
4.0
3.5
3.0
2.5
2.0
1.5
1.0
0.5
0.0
f1 (ppm)
12000
11000
10000
9000
8000
7000
6000
5000
4000
3000
2000
1000
0
-1000

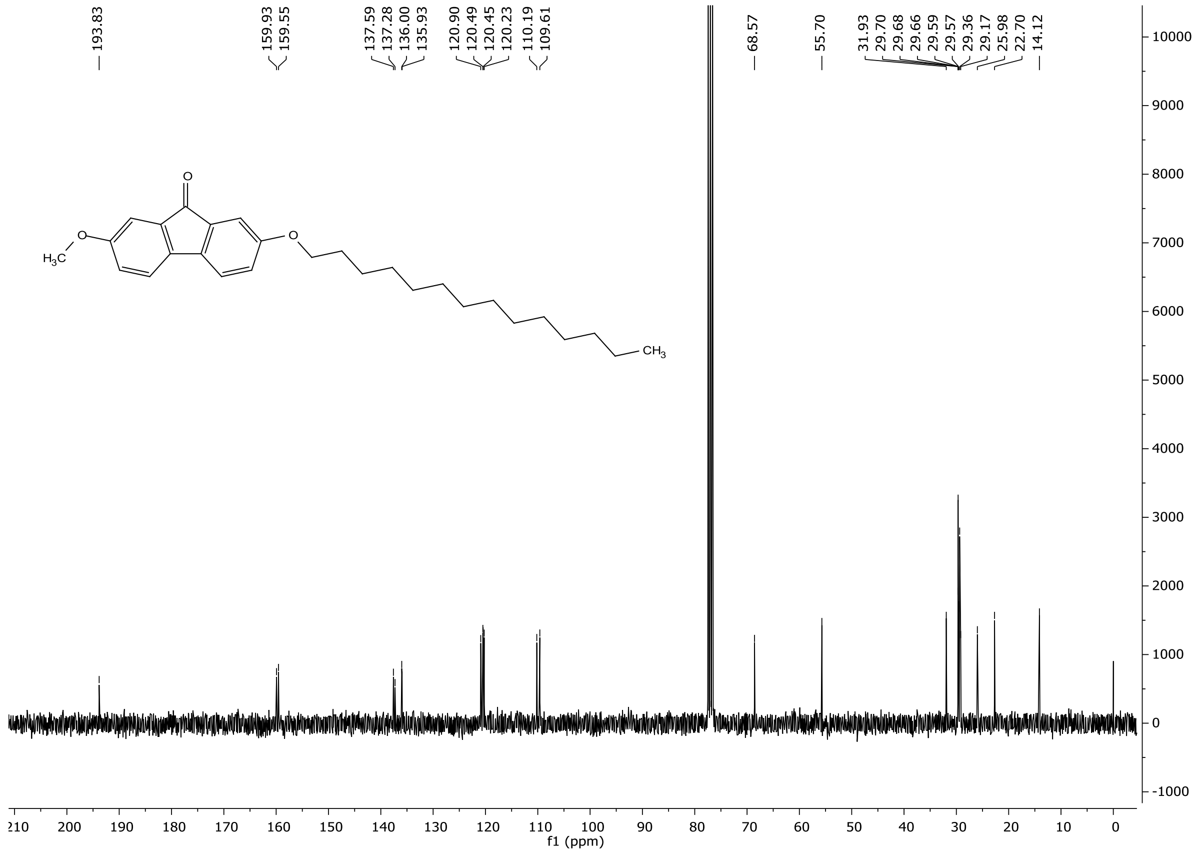

193.83
159.93
159.55
137.59
137.28
136.00
135.93
120.90
120.49
120.45
120.23
110.19
109.61
68.57
55.70
31.93
29.70
29.68
29.66
29.59
29.57
29.36
29.17
25.98
22.70
14.12
O
H_3C
O
O
CH_3
200
190
180
170
160
150
140
130
120
110
100
90
80
70
60
50
40
30
20
10
0
f1 (ppm)
10000
9000
8000
7000
6000
5000
4000
3000
2000
1000
0
-1000

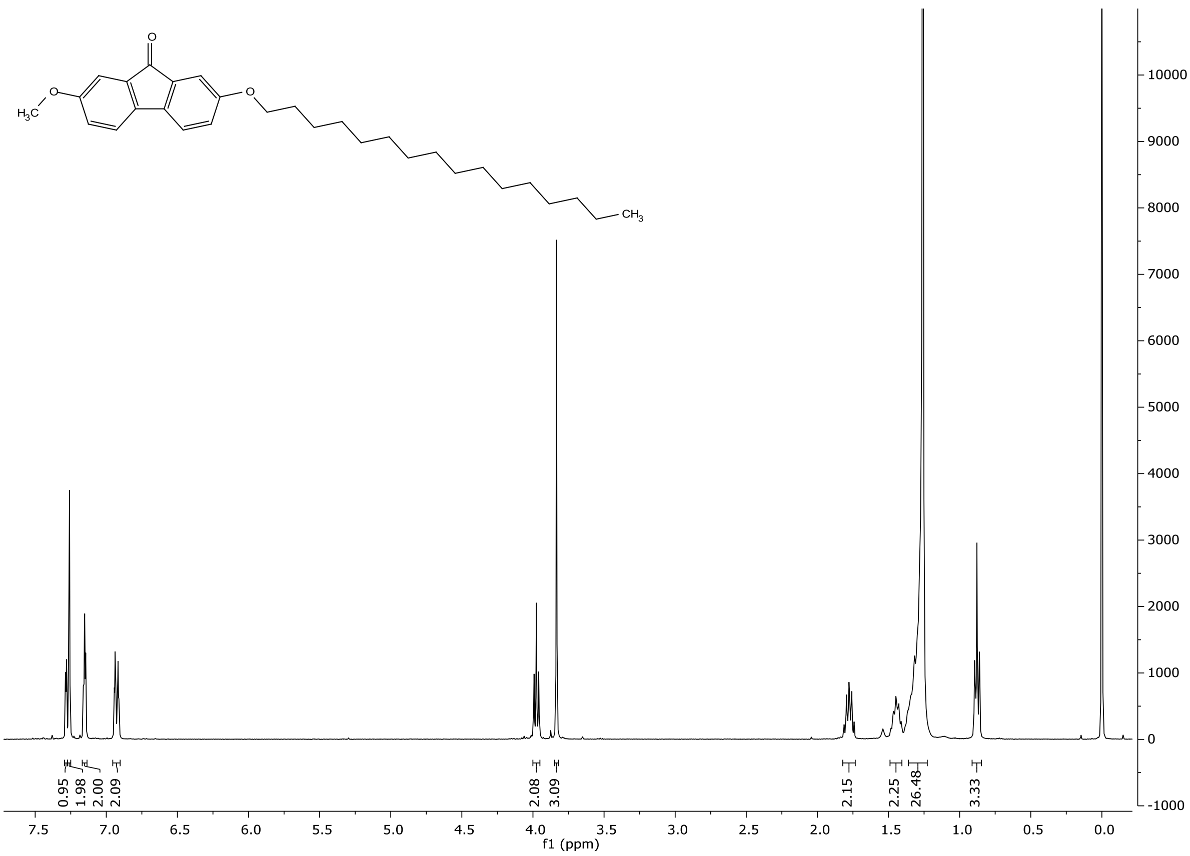

O
H_3C
O
O
CH_3
0.95
1.98
2.00
2.09
2.08
3.09
2.15
2.25
26.48
3.33
f1 (ppm)

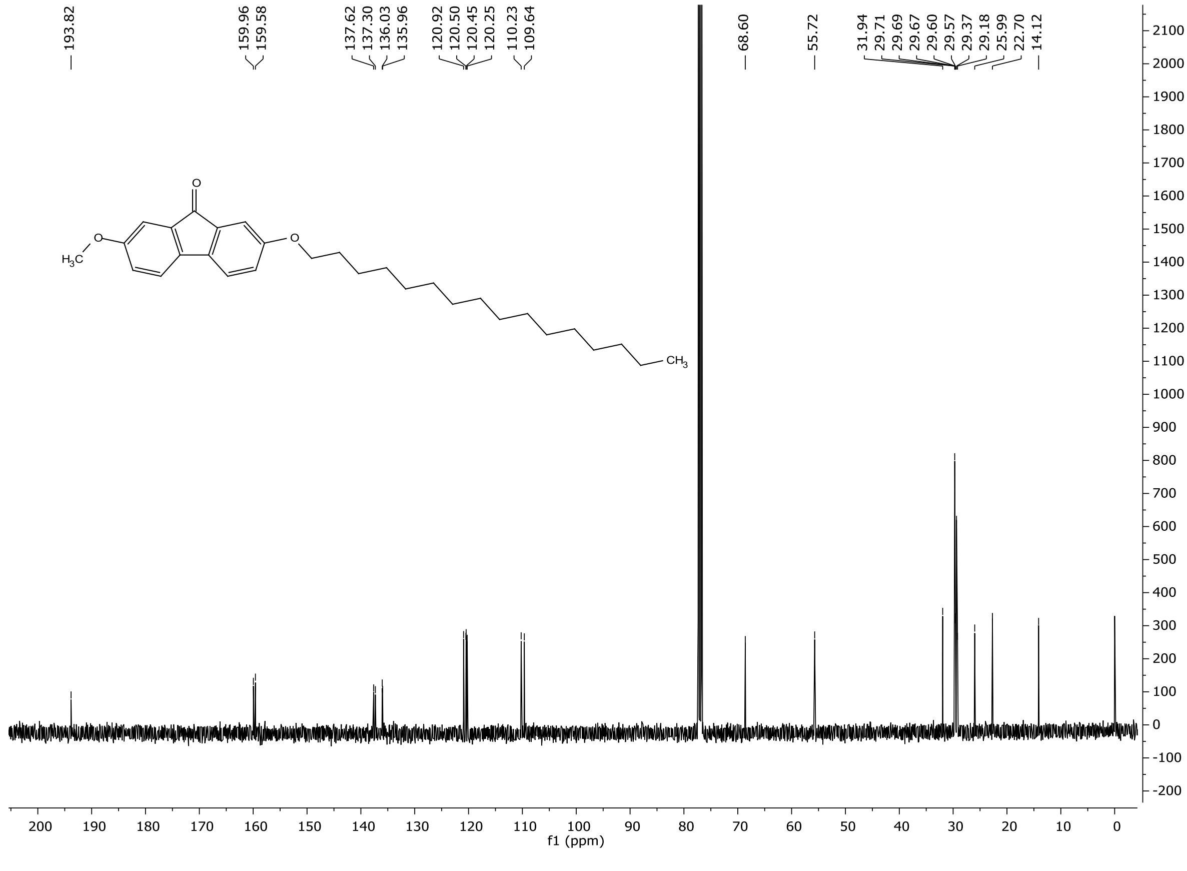

193.82
159.96
159.58
137.62
137.30
136.03
135.96
120.92
120.50
120.45
120.25
110.23
109.64
68.60
55.72
31.94
29.71
29.69
29.67
29.60
29.57
29.37
29.18
25.99
22.70
14.12
O
H_3C
O
O
CH_3
f1 (ppm)

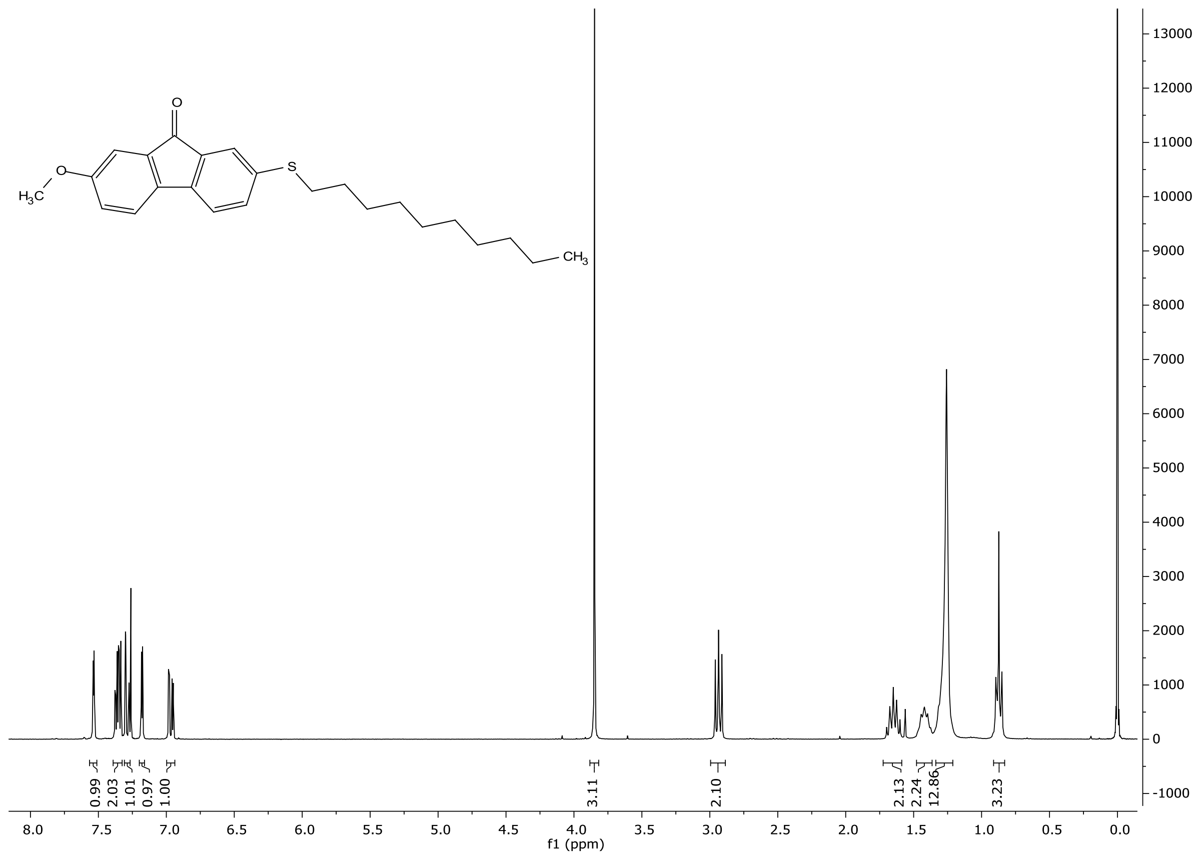

O
H3C
S
CH3
0.99
2.03
1.01
0.97
1.00
3.11
2.10
2.13
2.24
12.86
3.23
f1 (ppm)

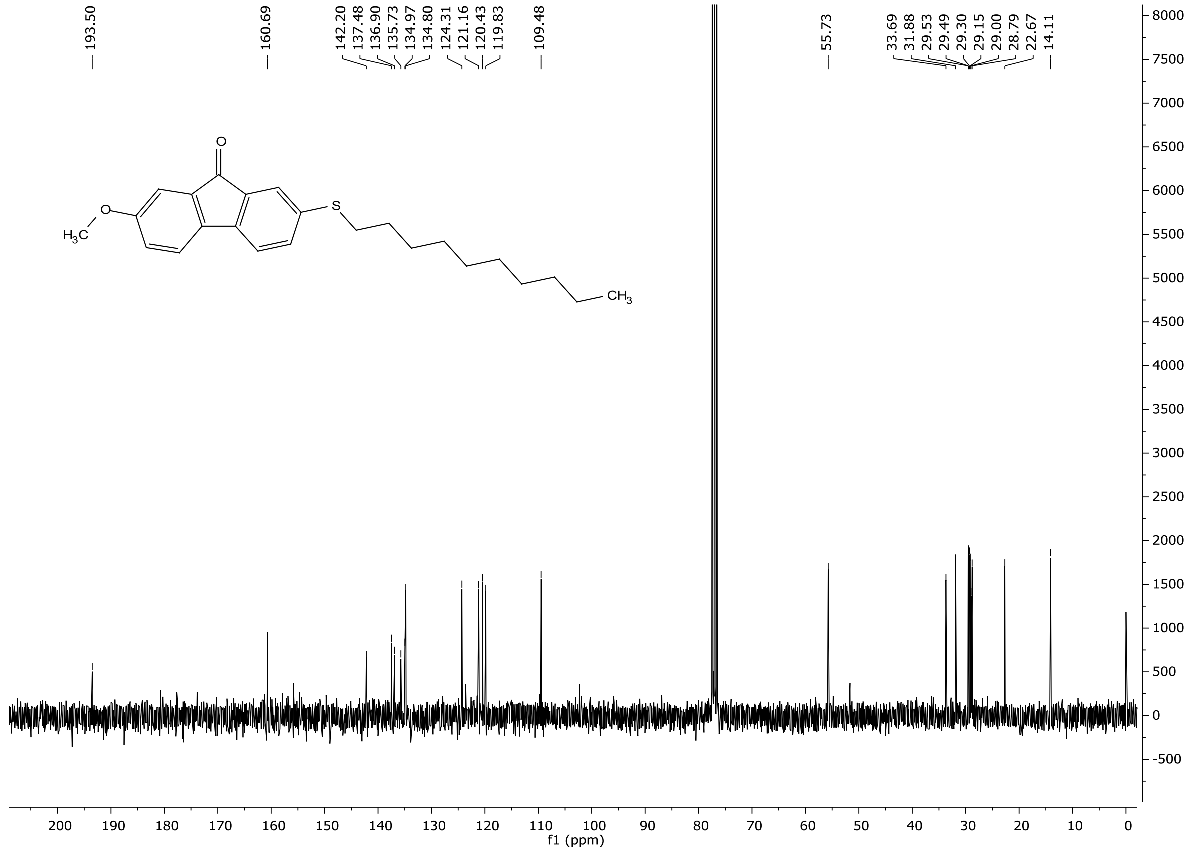

193.50
160.69
142.20
137.48
136.90
135.73
134.97
134.80
124.31
121.16
120.43
119.83
109.48
55.73
33.69
31.88
29.53
29.49
29.30
29.15
29.00
28.79
22.67
14.11
O
H3C
S
CH3
f1 (ppm)

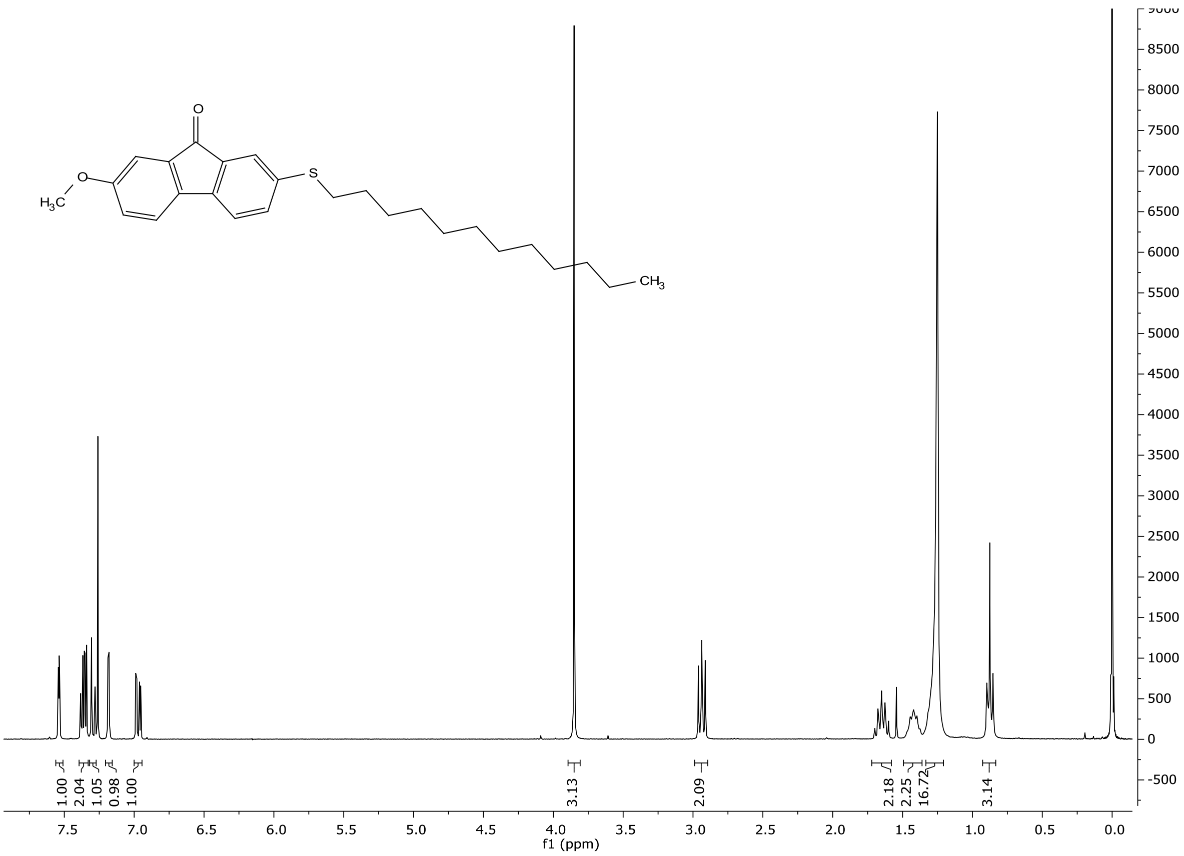

O
H_3C
S
CH_3
1.00
2.04
1.05
0.98
1.00
3.13
2.09
2.18
2.25
16.72
3.14
f1 (ppm)

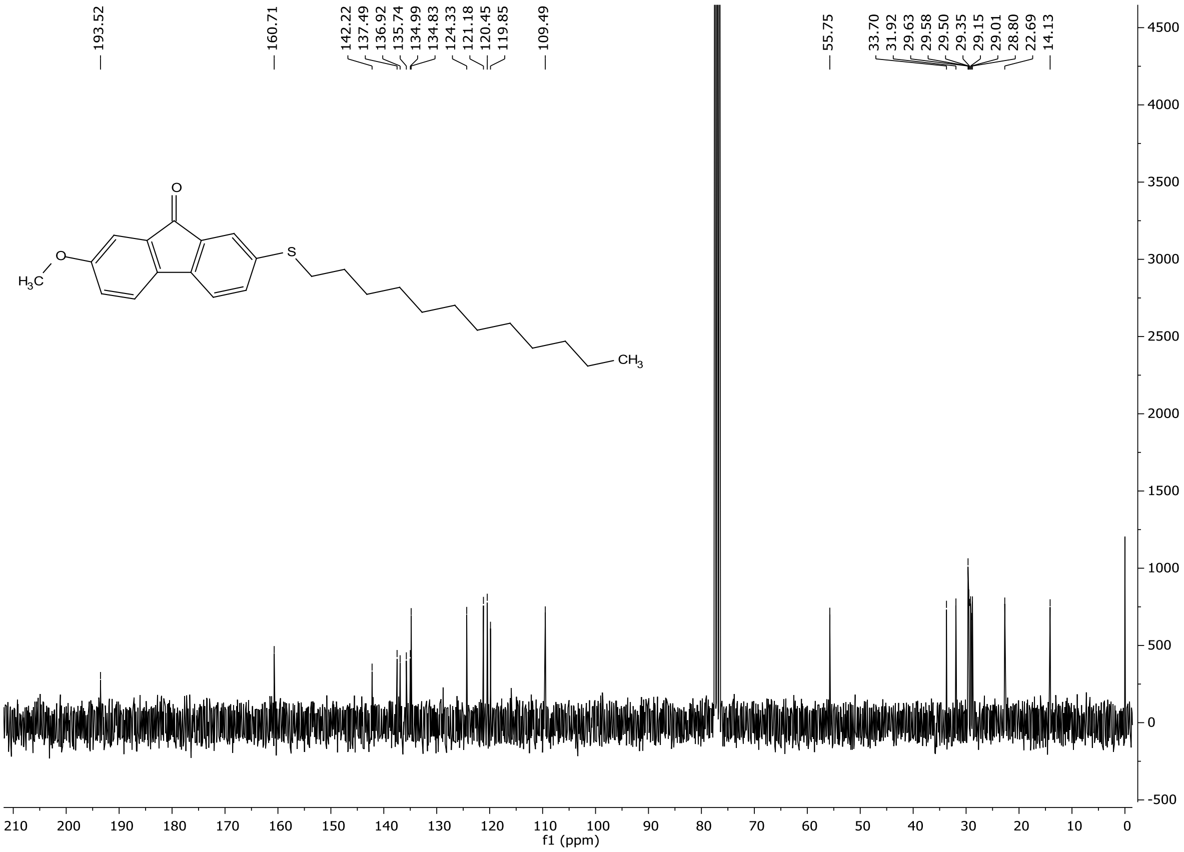

193.52
160.71
142.22
137.49
136.92
135.74
134.99
134.83
124.33
121.18
120.45
119.85
109.49
55.75
33.70
31.92
29.63
29.58
29.50
29.35
29.15
29.01
28.80
22.69
14.13
O
H_3C
S
CH_3
f1 (ppm)

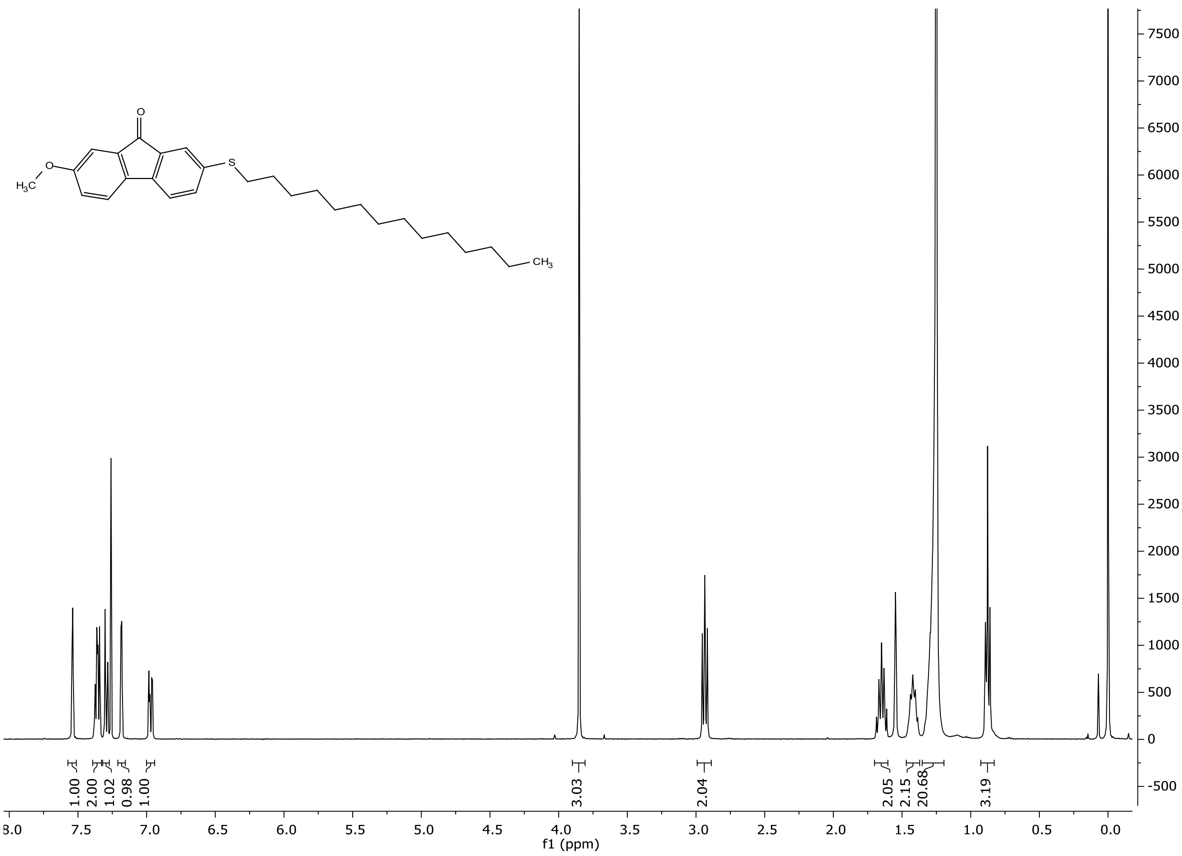
O
H3C
S
CH3
1.00
2.00
1.02
0.98
1.00
3.03
2.04
2.05
2.15
20.68
3.19
f1 (ppm)

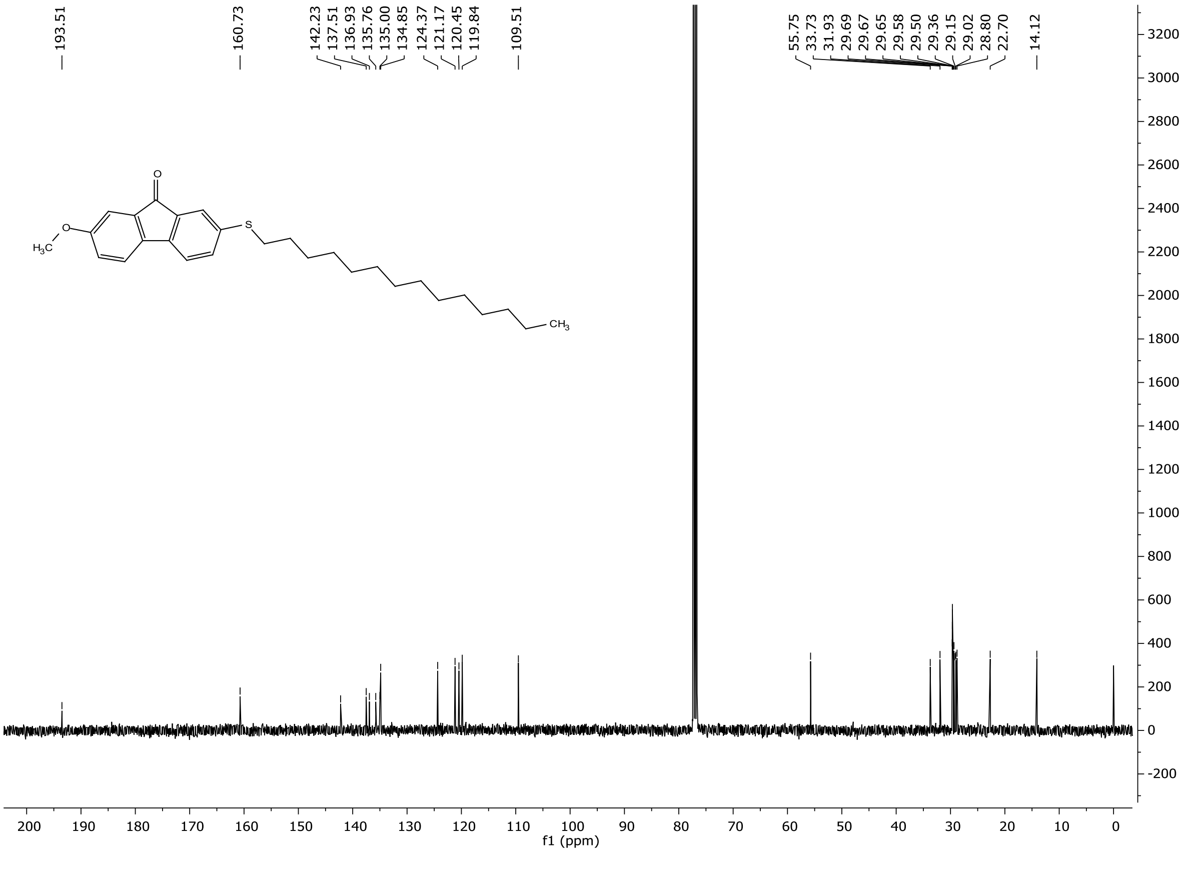
193.51
160.73
142.23
137.51
136.93
135.76
135.00
134.85
124.37
121.17
120.45
119.84
109.51
55.75
33.73
31.93
29.69
29.67
29.65
29.58
29.50
29.36
29.15
29.02
28.80
22.70
14.12
O
H3C
S
CH3
f1 (ppm)

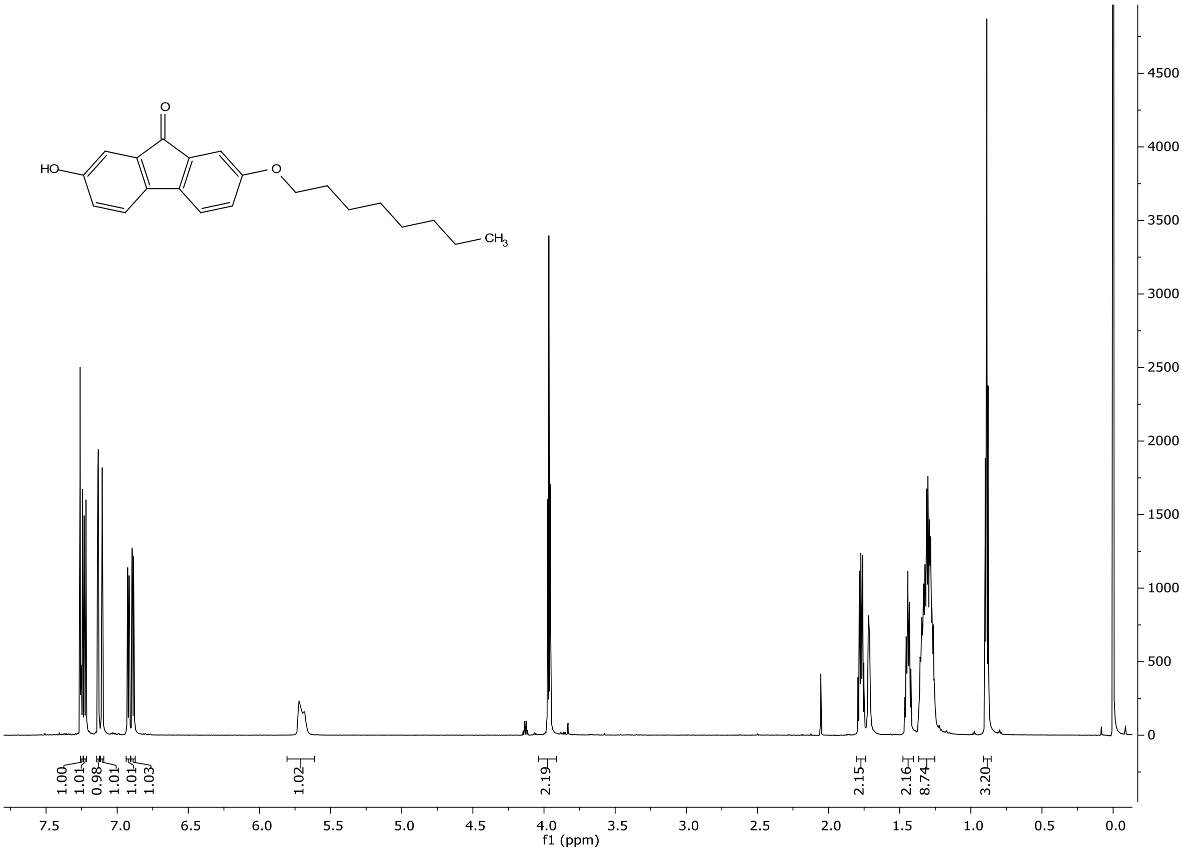

HO
O
O
CH3
1.00
1.01
0.98
1.01
1.01
1.03
1.02
2.19
2.15
2.16
8.74
3.20
f1 (ppm)

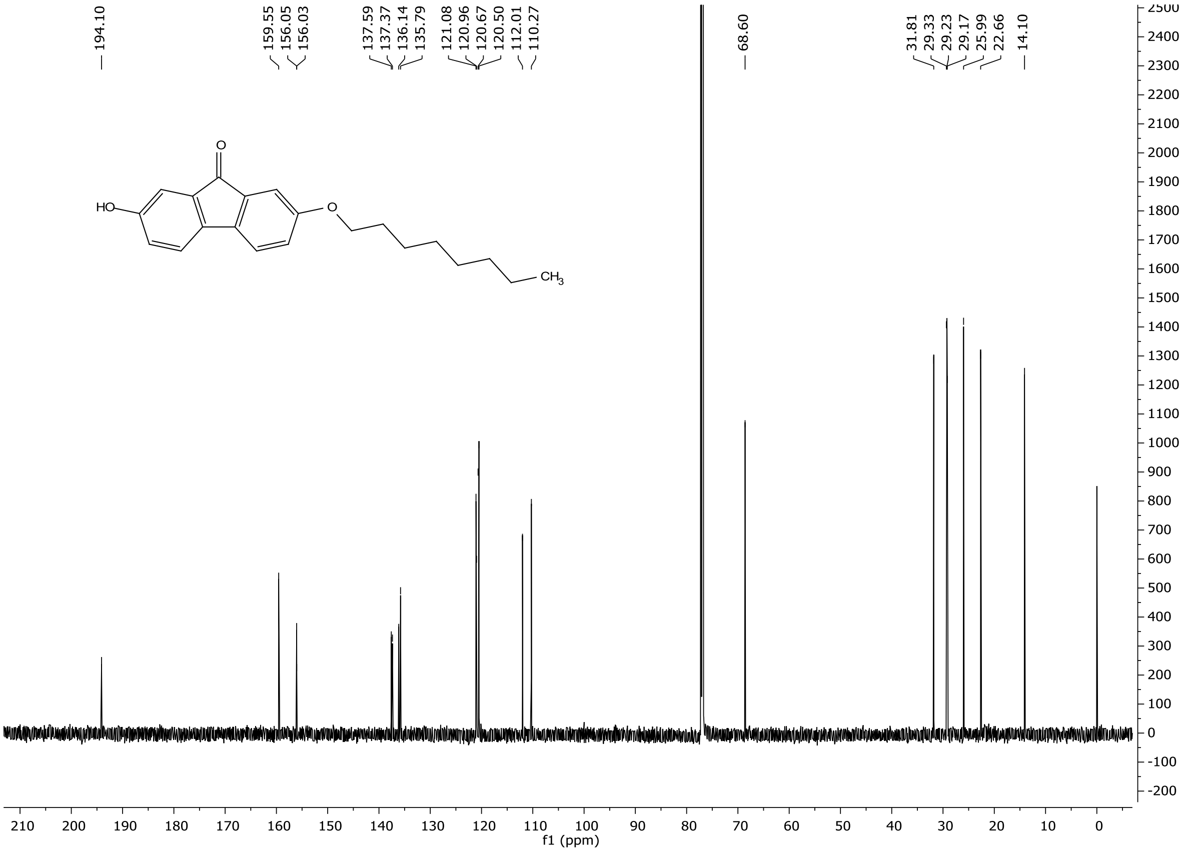

194.10
159.55
156.05
156.03
137.59
137.37
136.14
135.79
121.08
120.96
120.67
120.50
112.01
110.27
68.60
31.81
29.33
29.23
29.17
25.99
22.66
14.10
HO
O
O
CH3
f1 (ppm)

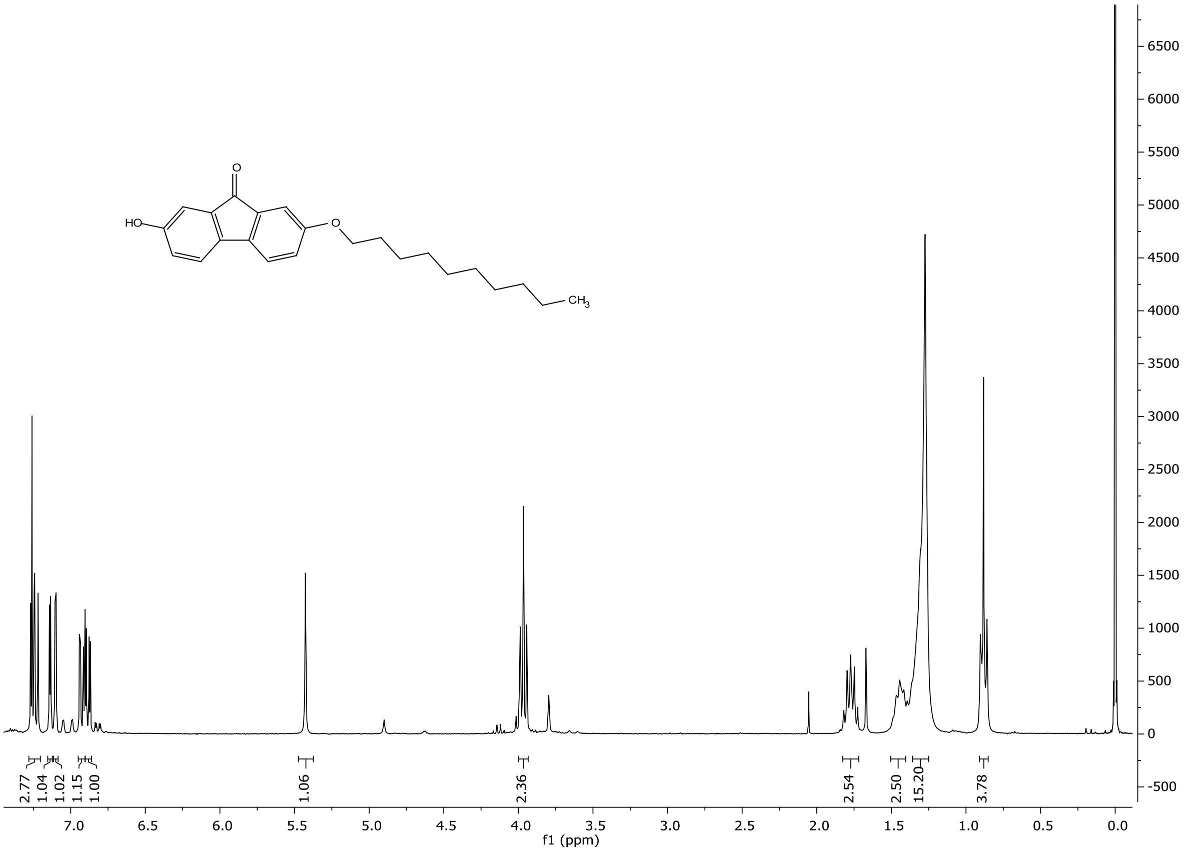
HO
O
O
CH3
2.77
1.04
1.02
1.15
1.00
1.06
2.36
2.54
2.50
15.20
3.78
f1 (ppm)

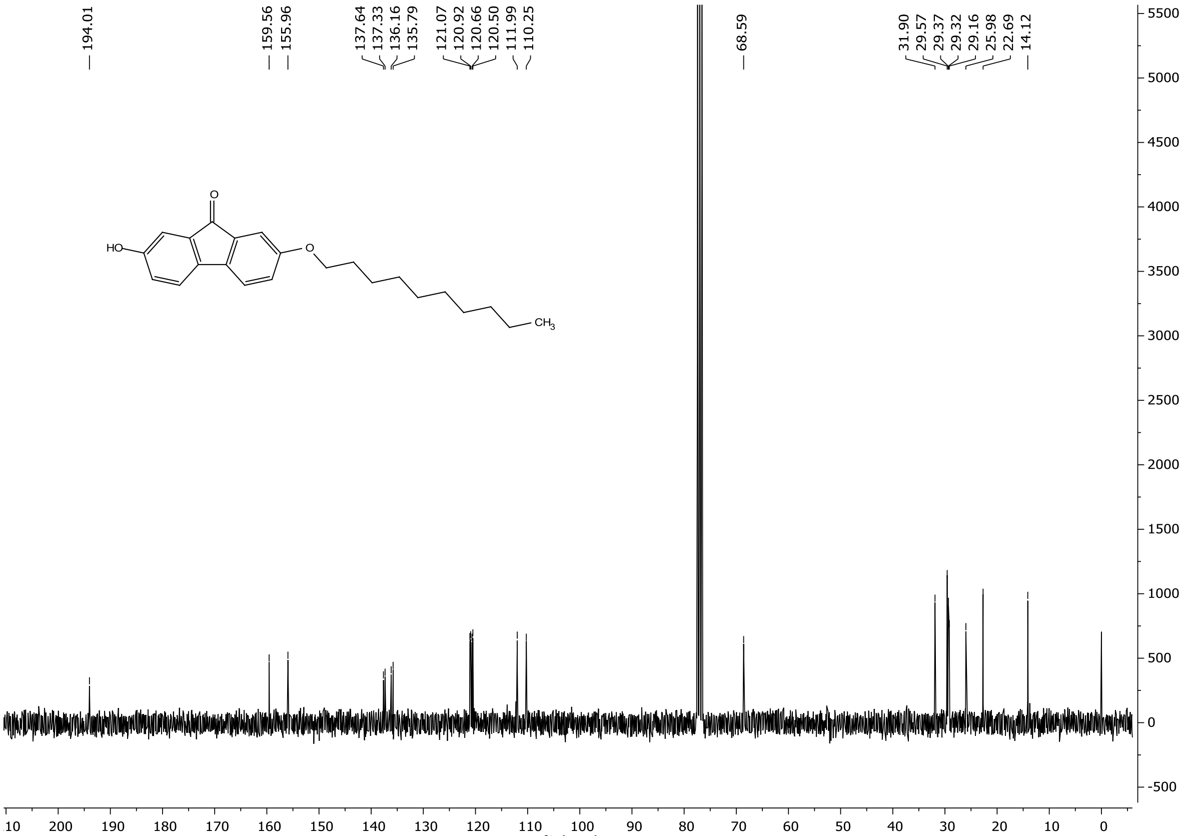
194.01
159.56
155.96
137.64
137.33
136.16
135.79
121.07
120.92
120.66
120.50
111.99
110.25
68.59
31.90
29.57
29.37
29.32
29.16
25.98
22.69
14.12
HO
O
O
CH3
f1 (ppm)

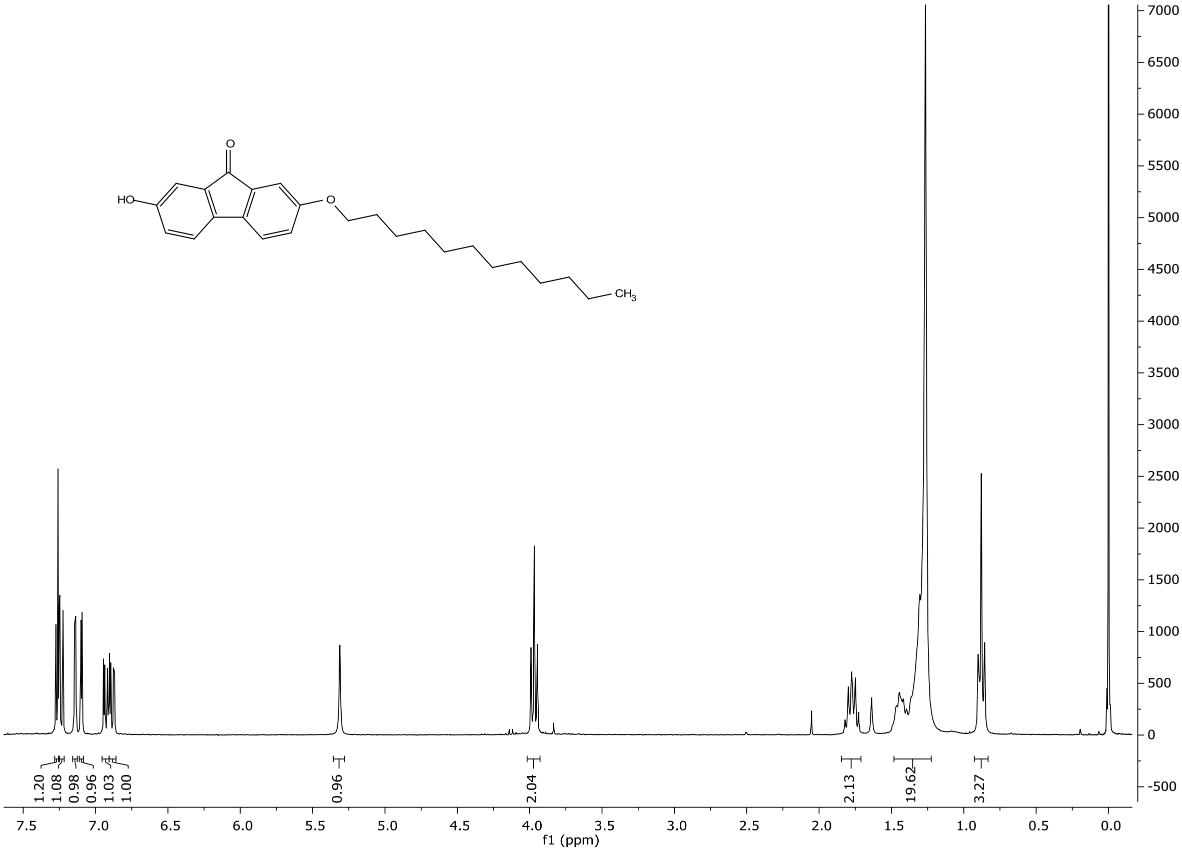
HO
O
CH3
1.20
1.08
0.98
0.96
1.03
1.00
0.96
2.04
2.13
19.62
3.27
f1 (ppm)

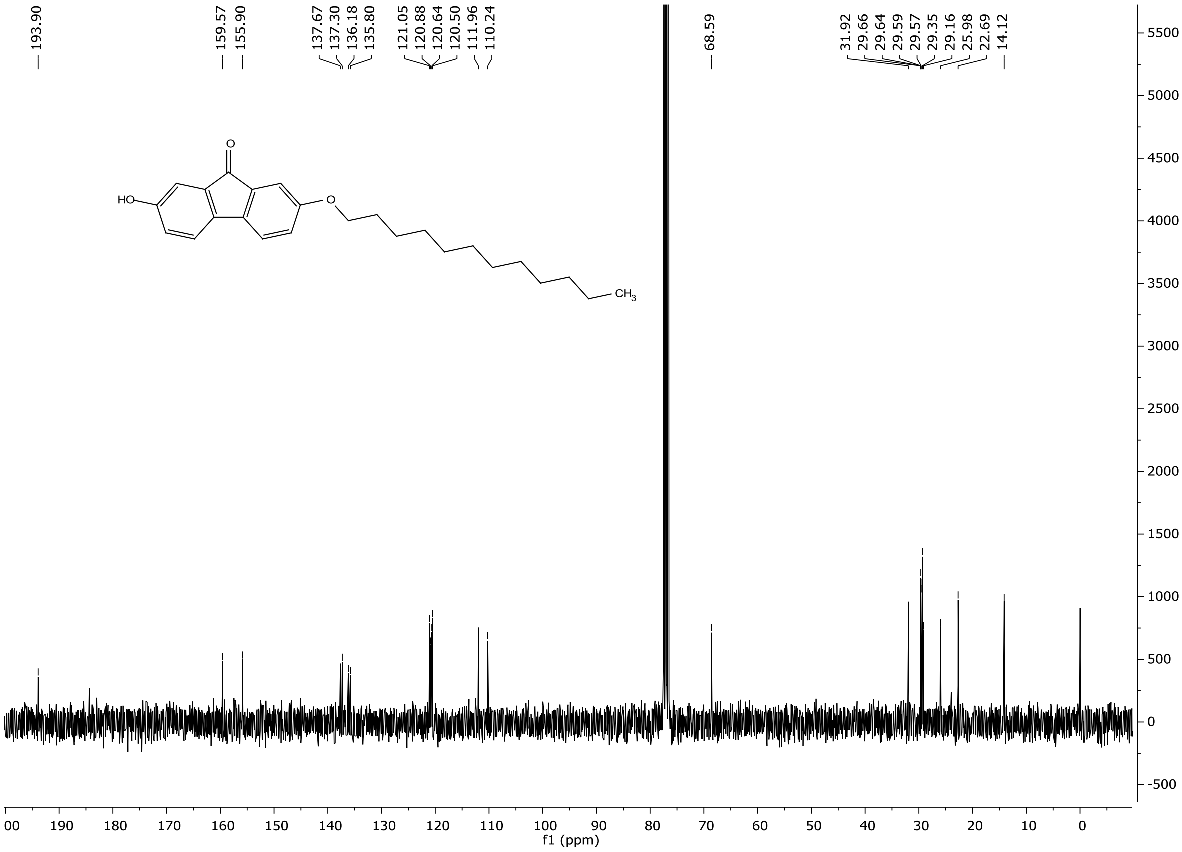
193.90
159.57
155.90
137.67
137.30
136.18
135.80
121.05
120.88
120.64
120.50
111.96
110.24
68.59
31.92
29.66
29.64
29.59
29.57
29.35
29.16
25.98
22.69
14.12
HO
O
CH3
f1 (ppm)

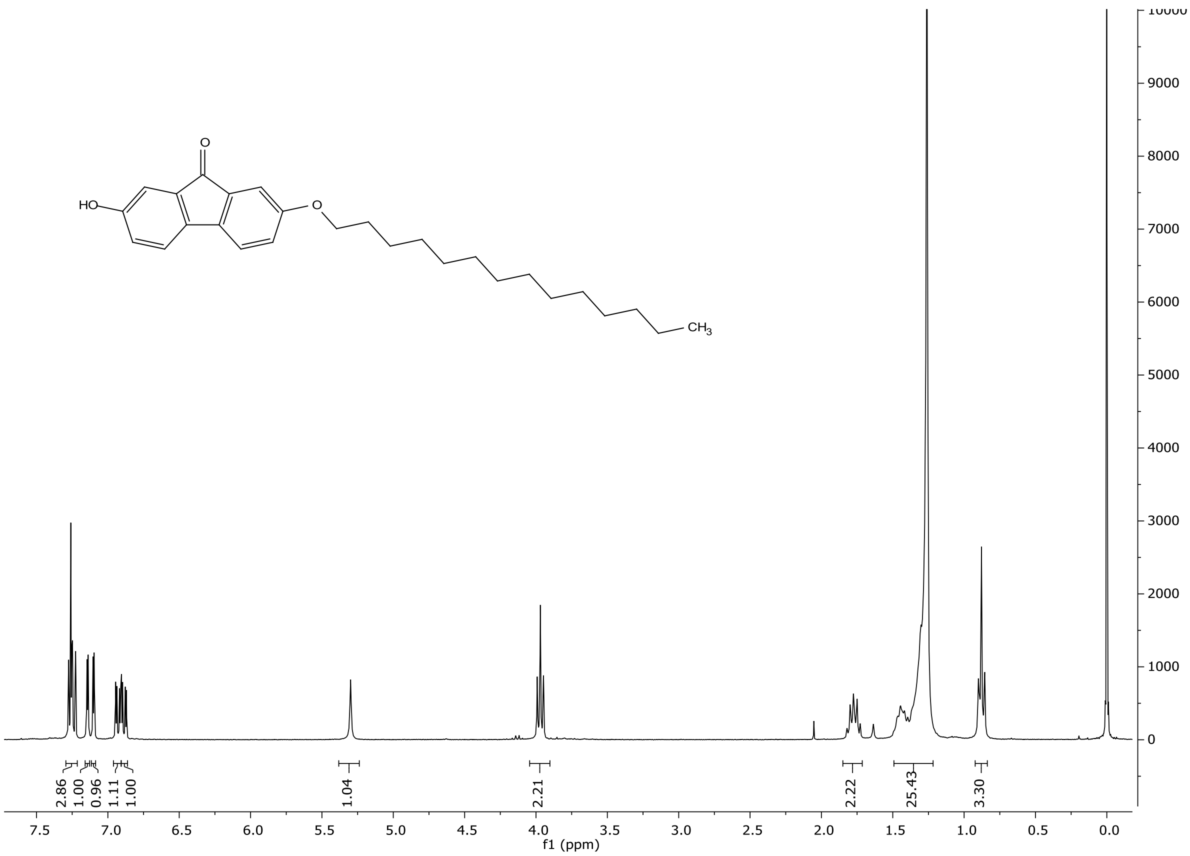

O
HO
O
CH3
2.86
1.00
0.96
1.11
1.00
1.04
2.21
2.22
25.43
3.30
f1 (ppm)

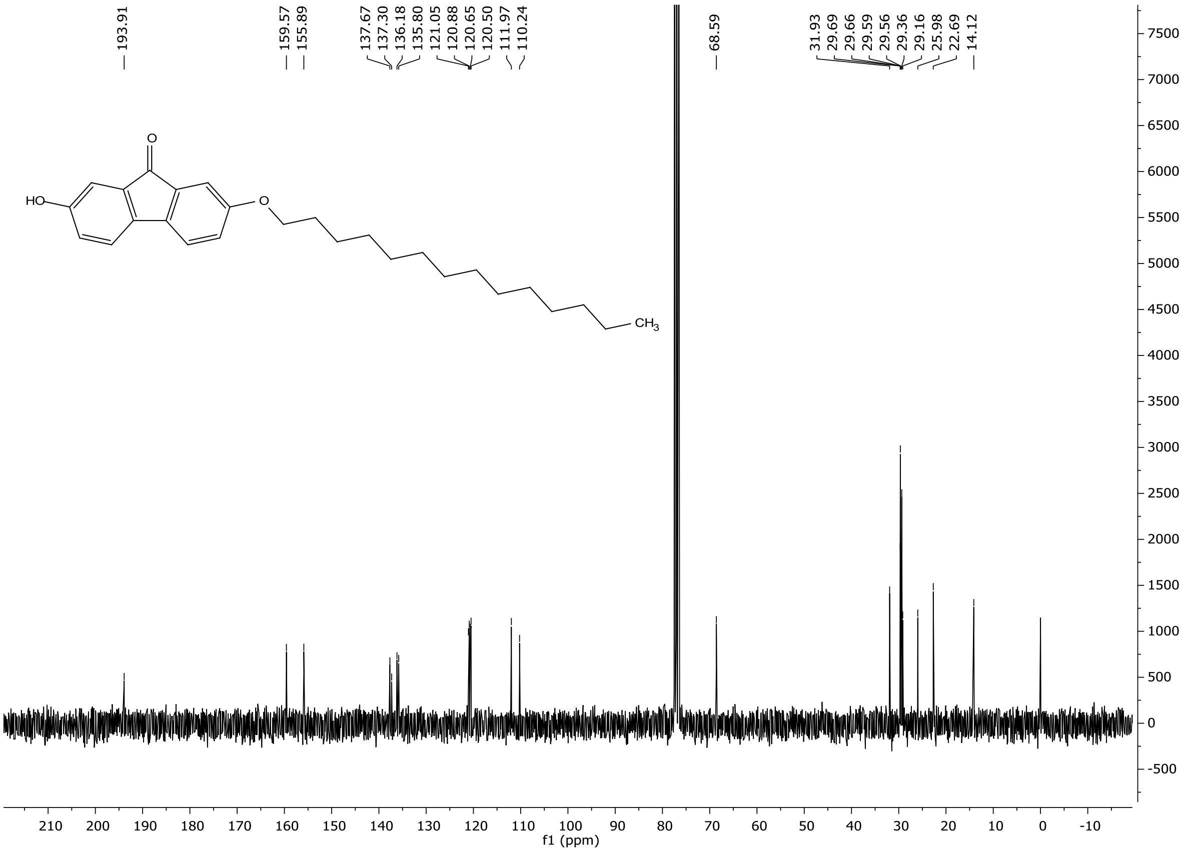

193.91
159.57
155.89
137.67
137.30
136.18
135.80
121.05
120.88
120.65
120.50
111.97
110.24
68.59
31.93
29.69
29.66
29.59
29.56
29.36
29.16
25.98
22.69
14.12
O
HO
O
CH3
f1 (ppm)

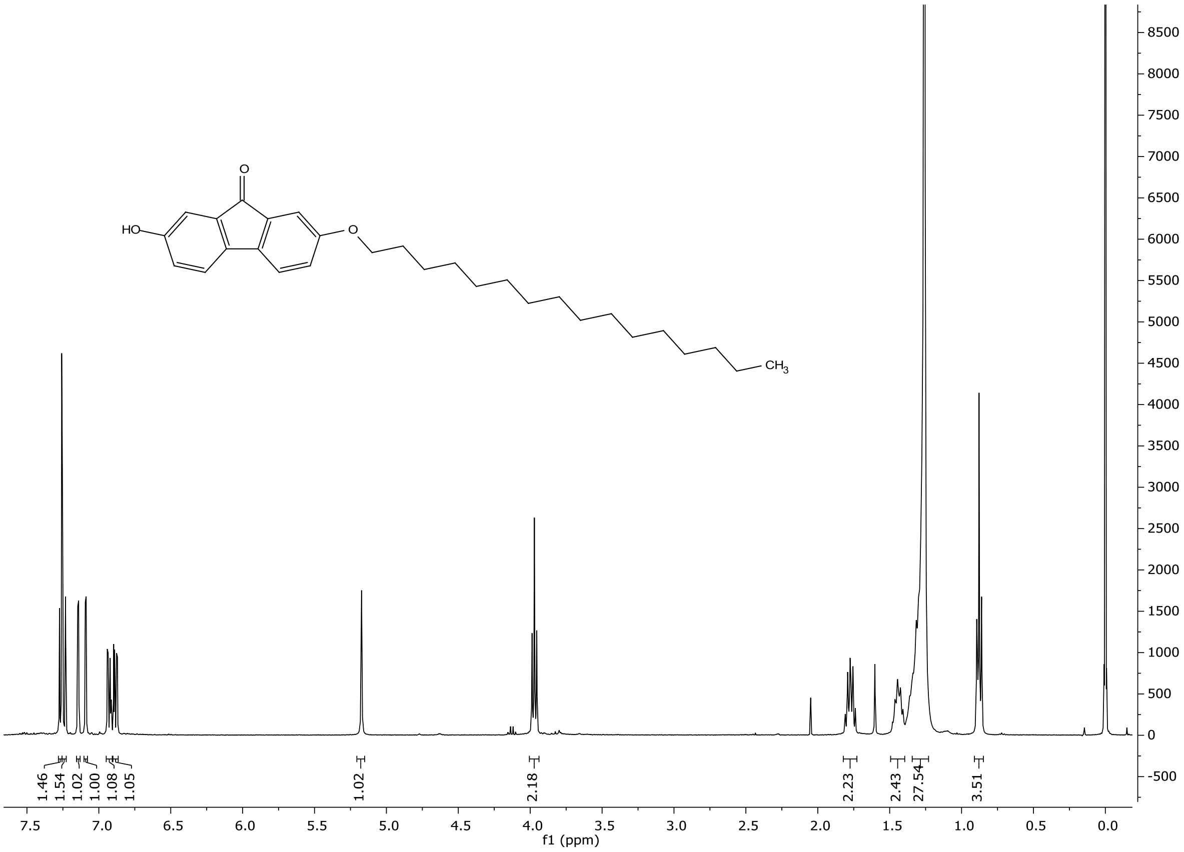
HO
O
CH3
1.46
1.54
1.02
1.00
1.08
1.05
1.02
2.18
2.23
2.43
27.54
3.51
f1 (ppm)

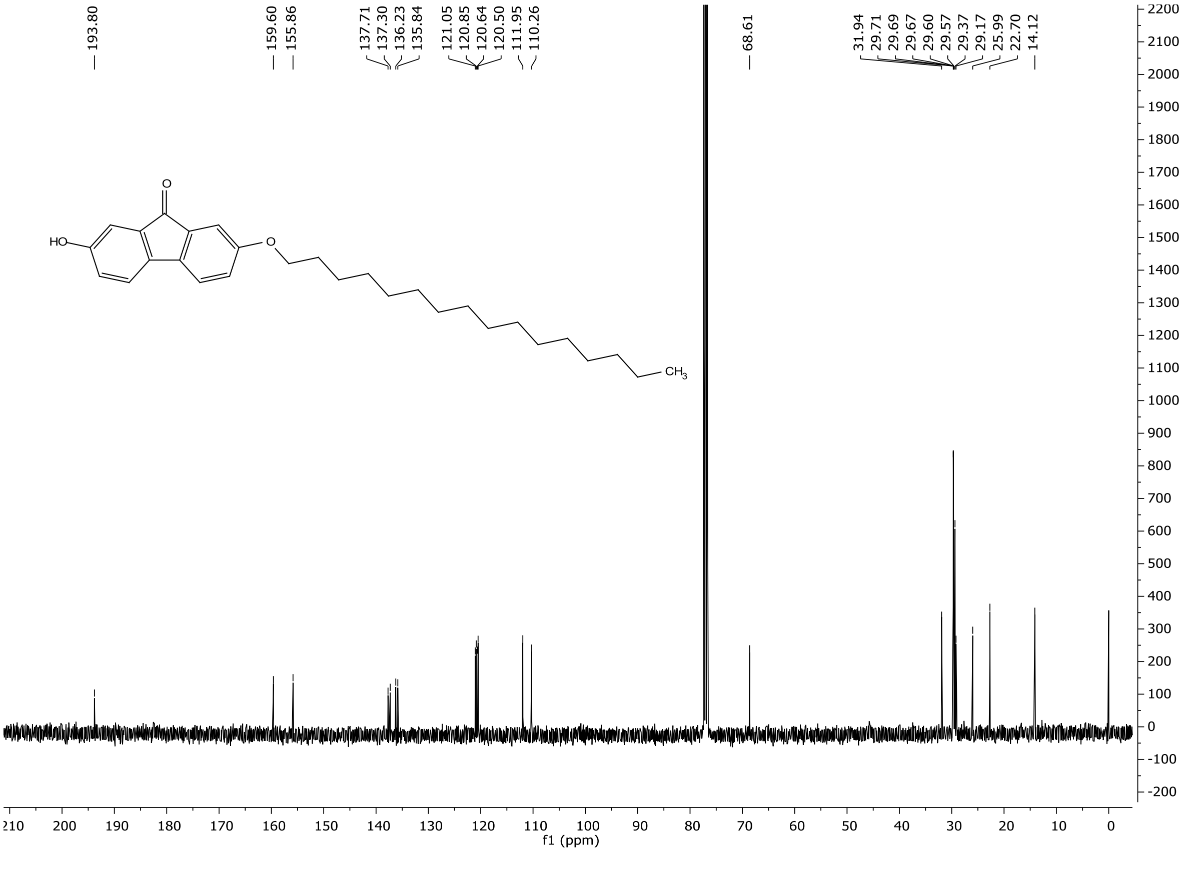
193.80
159.60
155.86
137.71
137.30
136.23
135.84
121.05
120.85
120.64
120.50
111.95
110.26
68.61
31.94
29.71
29.69
29.67
29.60
29.57
29.37
29.17
25.99
22.70
14.12
HO
O
CH3
f1 (ppm)

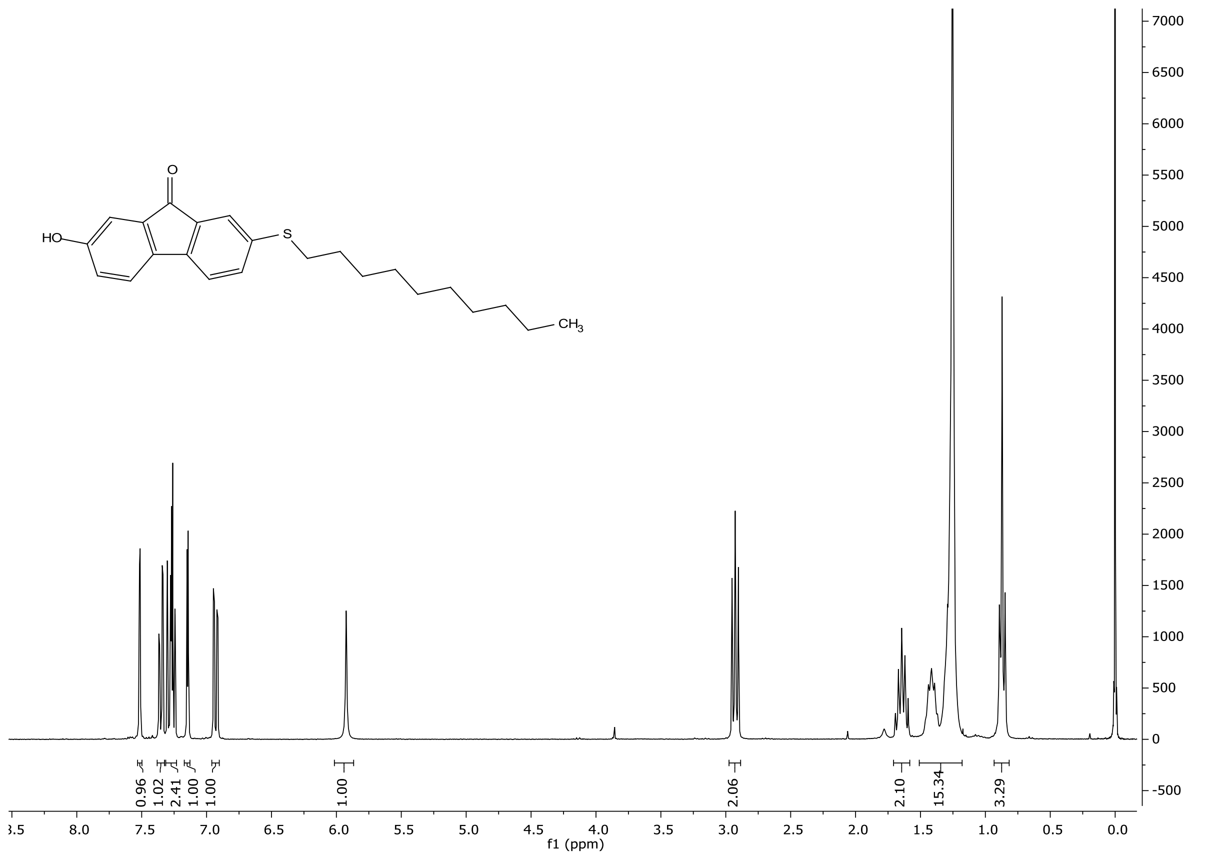
HO
S
CH3
O
0.96
1.02
2.41
1.00
1.00
1.00
2.06
2.10
15.34
3.29
f1 (ppm)

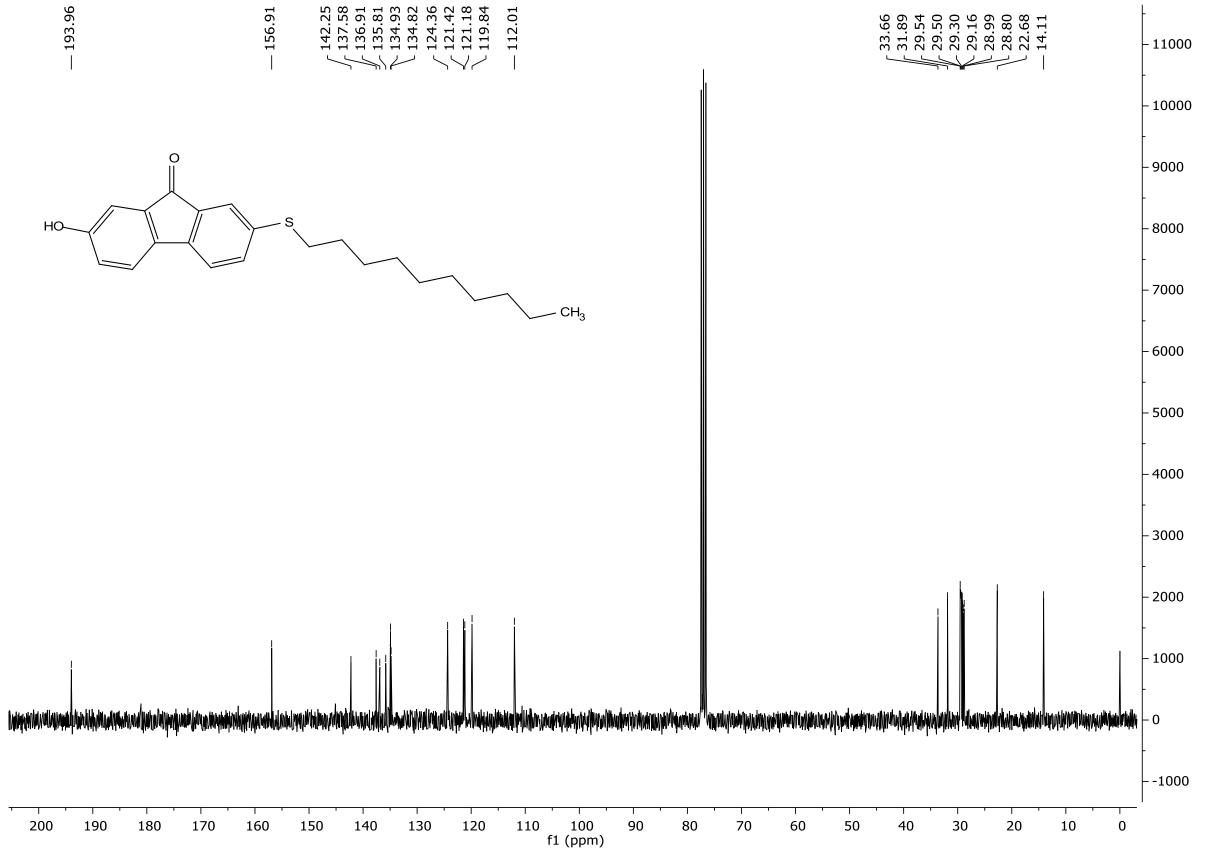
193.96
156.91
142.25
137.58
136.91
135.81
134.93
134.82
124.36
121.42
121.18
119.84
112.01
33.66
31.89
29.54
29.50
29.30
29.16
28.99
28.80
22.68
14.11
HO
S
CH3
O
f1 (ppm)

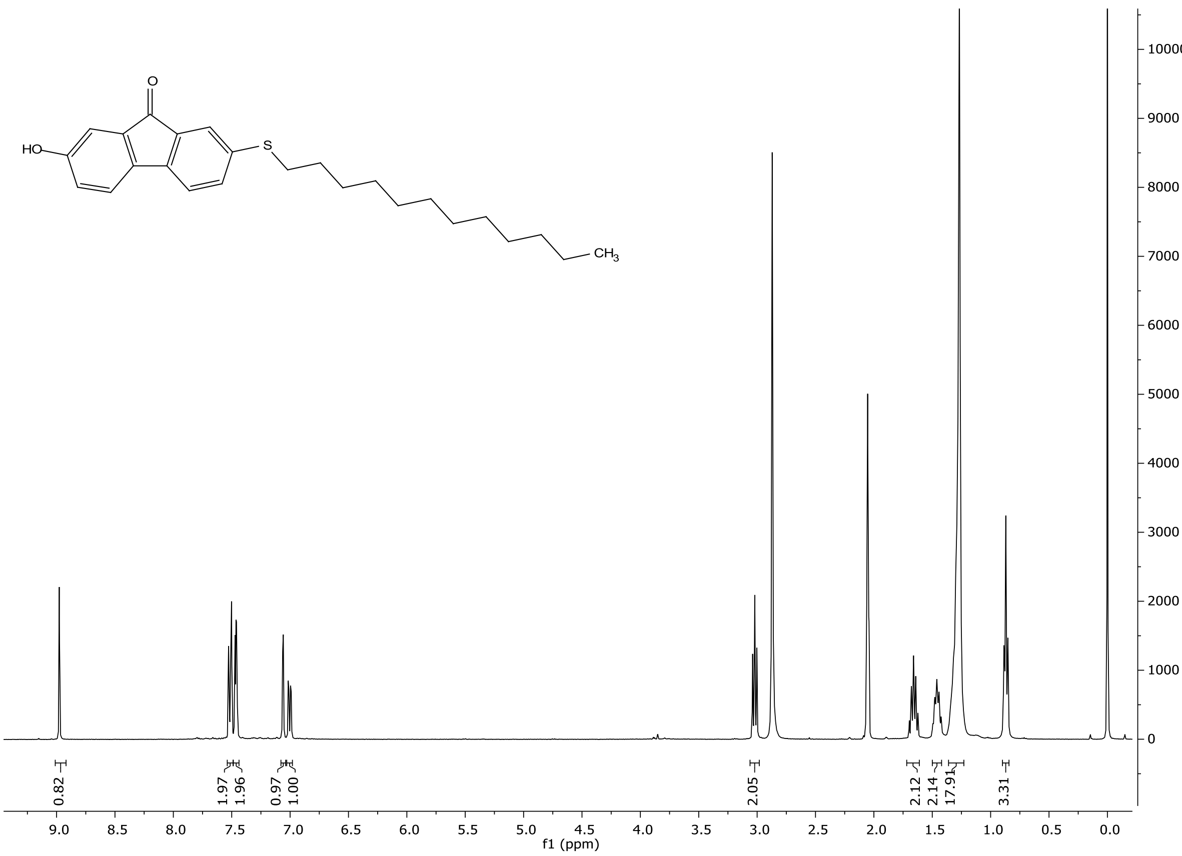

HO
S
CH3
O
0.82
1.97
1.96
0.97
1.00
2.05
2.12
2.14
17.91
3.31
f1 (ppm)

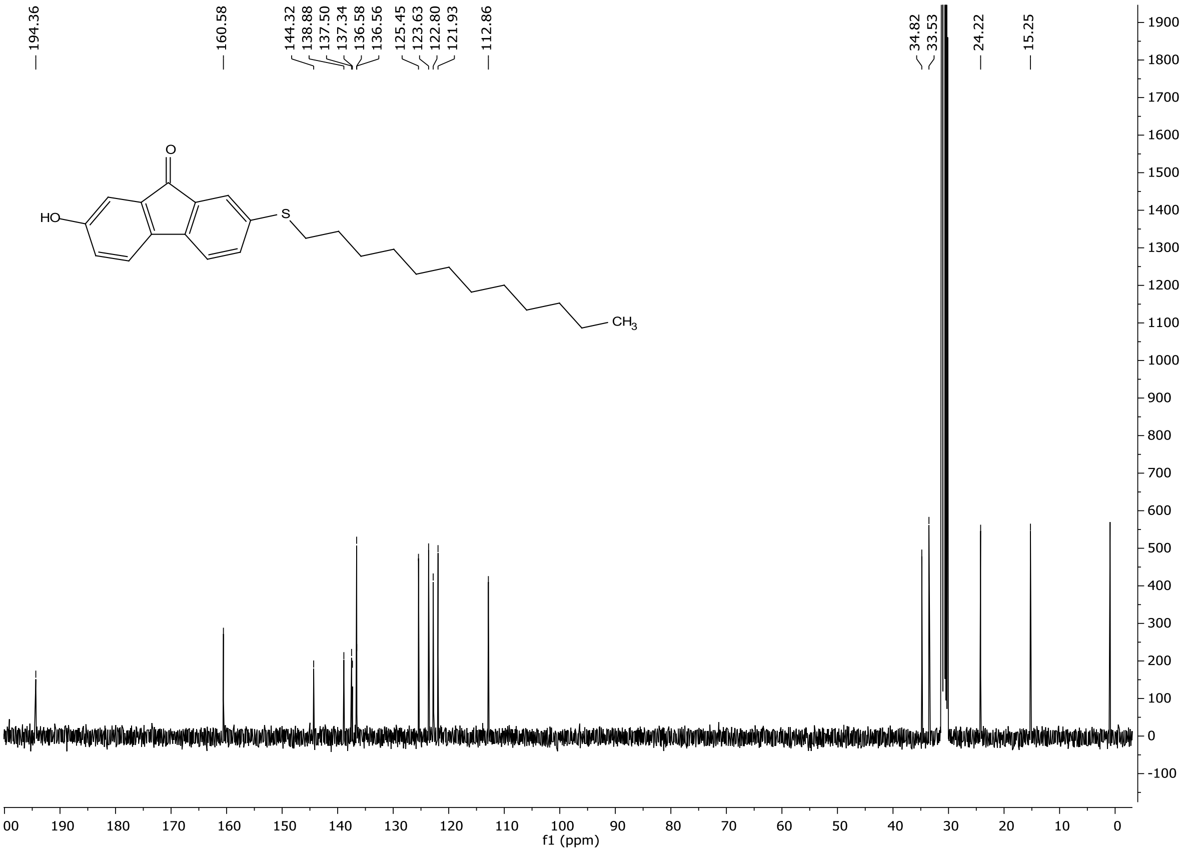

194.36
160.58
144.32
138.88
137.50
137.34
136.58
136.56
125.45
123.63
122.80
121.93
112.86
34.82
33.53
24.22
15.25
HO
S
CH3
O
f1 (ppm)

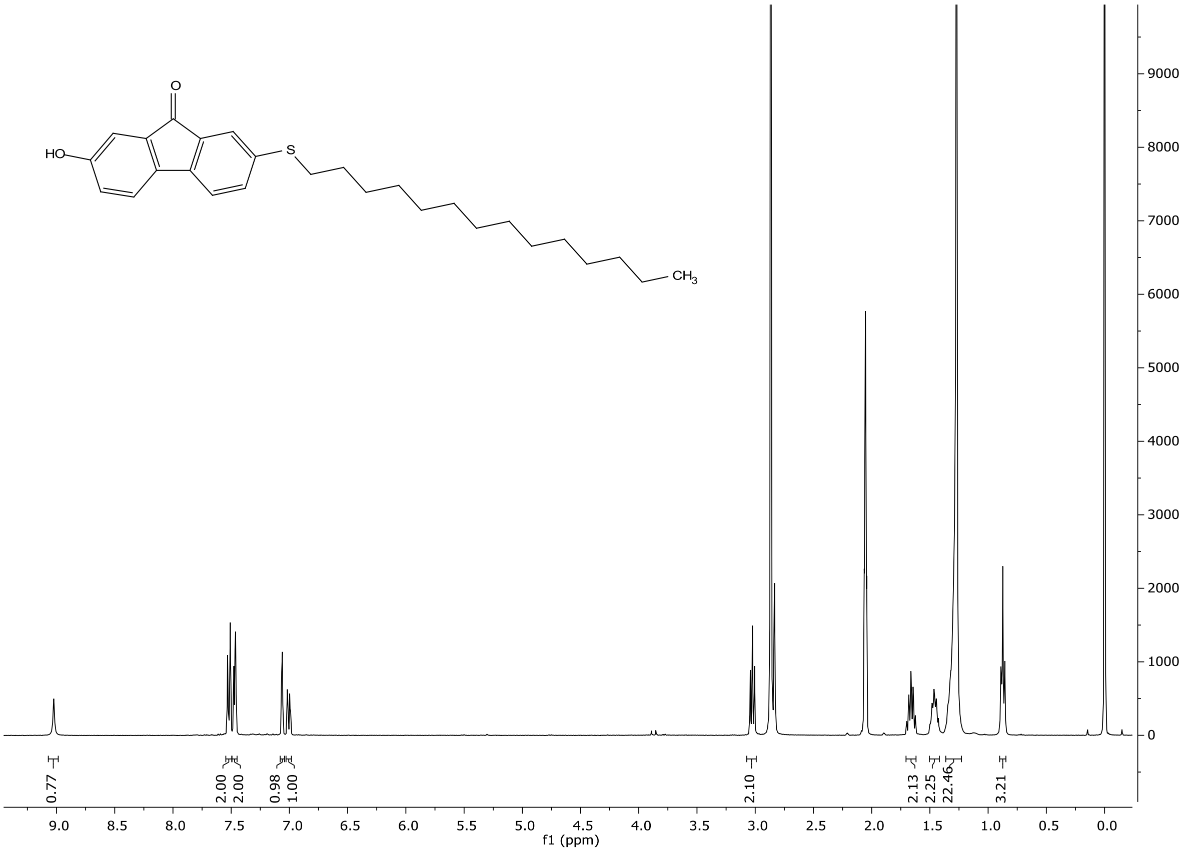
HO
S
O
CH3
0.77
2.00
2.00
0.98
1.00
2.10
2.13
2.25
22.46
3.21
f1 (ppm)

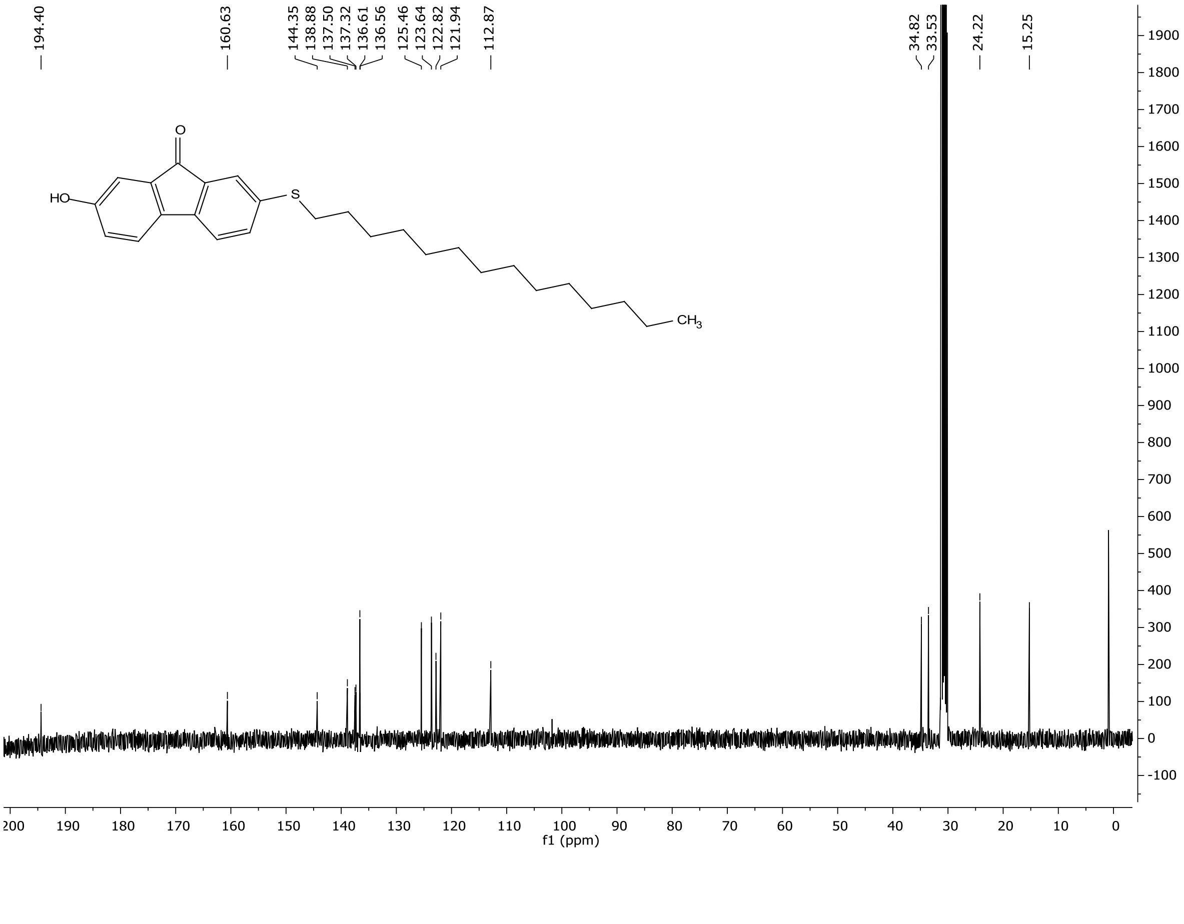
194.40
160.63
144.35
138.88
137.50
137.32
136.61
136.56
125.46
123.64
122.82
121.94
112.87
34.82
33.53
24.22
15.25
HO
S
O
CH3
f1 (ppm)

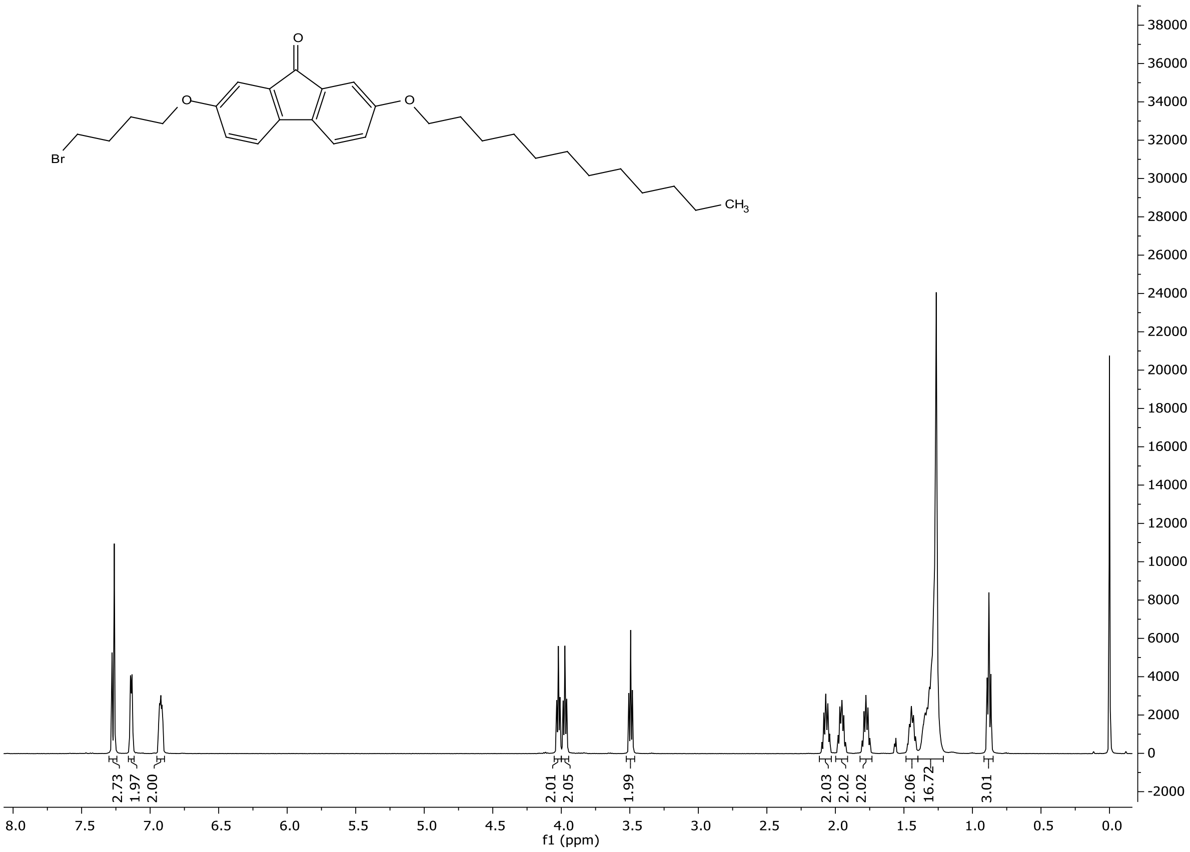

O
O
O
Br
CH3
2.73
1.97
2.00
2.01
2.05
1.99
2.03
2.02
2.02
2.06
16.72
3.01
f1 (ppm)

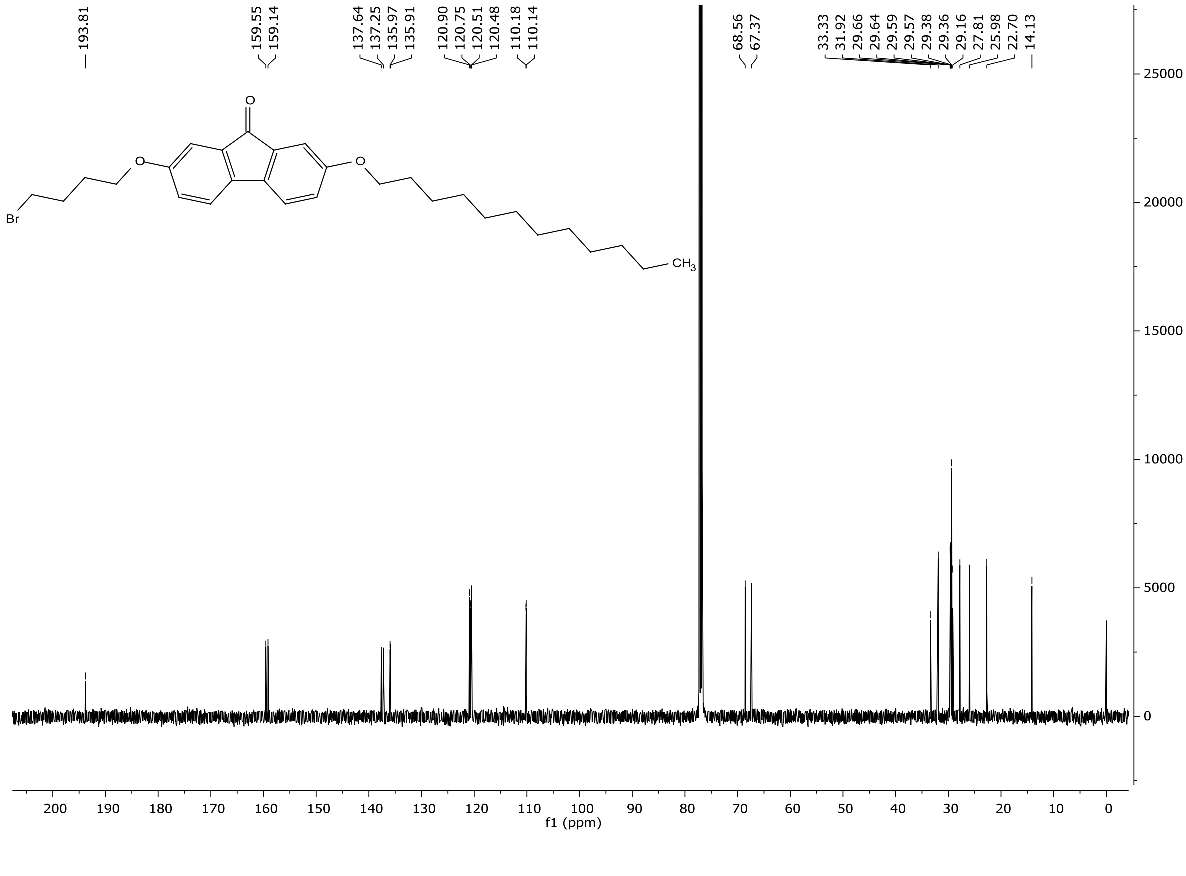

193.81
159.55
159.14
137.64
137.25
135.97
135.91
120.90
120.75
120.51
120.48
110.18
110.14
68.56
67.37
33.33
31.92
29.66
29.64
29.59
29.57
29.38
29.36
29.16
27.81
25.98
22.70
14.13
O
O
O
Br
CH3
f1 (ppm)

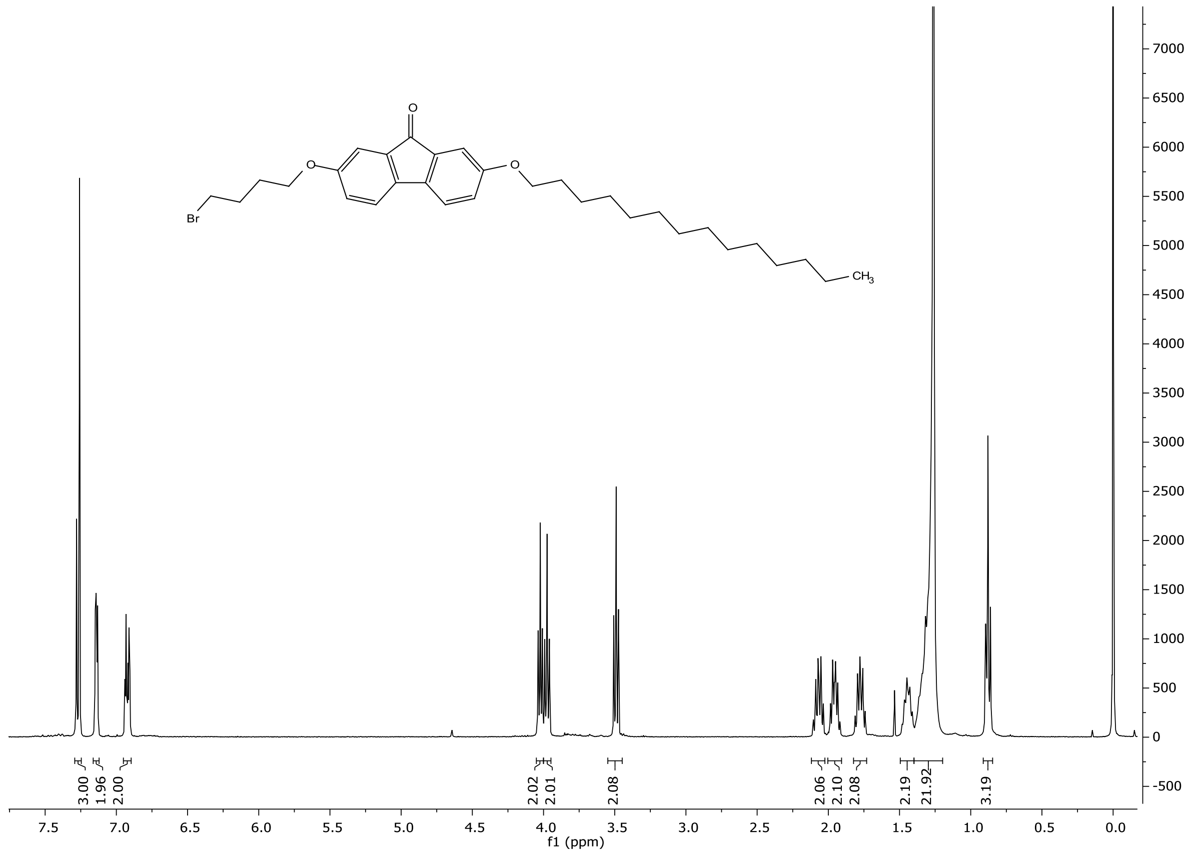

Br
O
O
O
CH3
3.00
1.96
2.00
2.02
2.01
2.08
2.06
2.10
2.08
2.19
21.92
3.19
f1 (ppm)

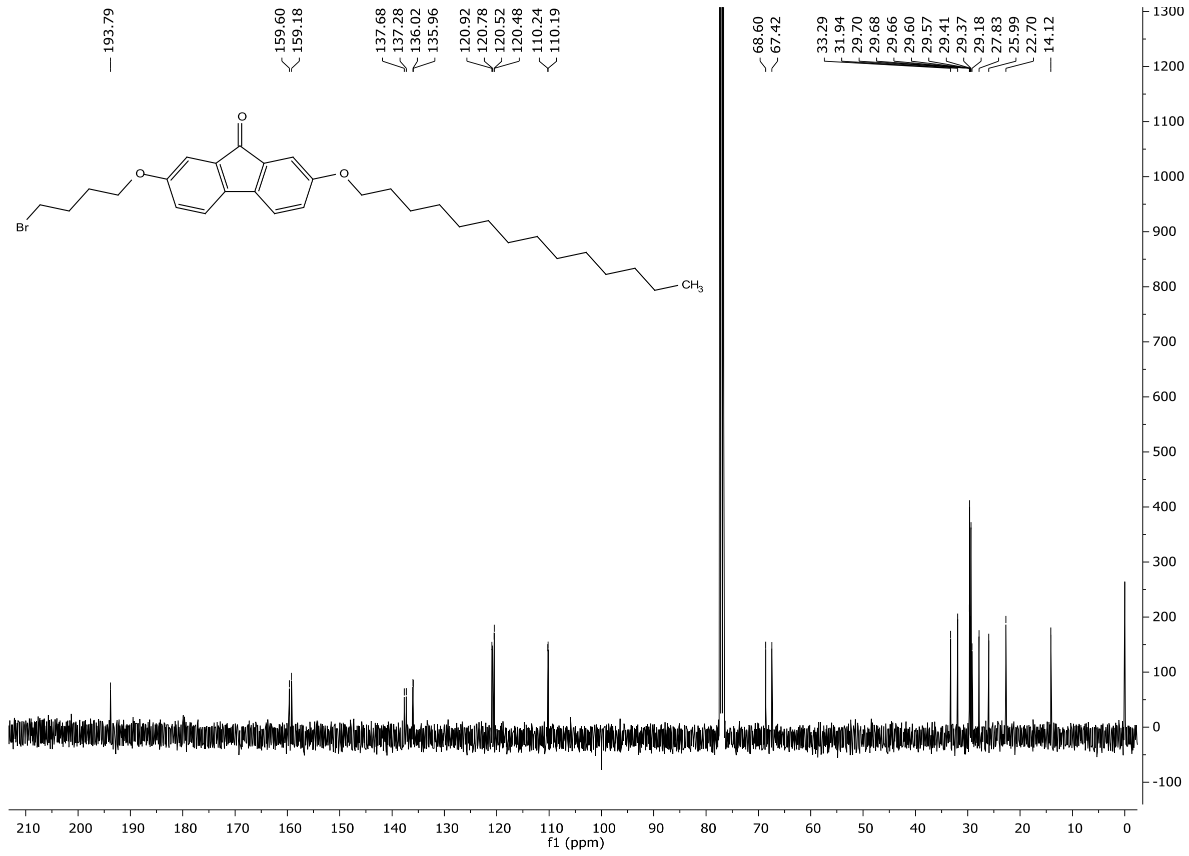

193.79
159.60
159.18
137.68
137.28
136.02
135.96
120.92
120.78
120.52
120.48
110.24
110.19
68.60
67.42
33.29
31.94
29.70
29.68
29.66
29.60
29.57
29.41
29.37
29.18
27.83
25.99
22.70
14.12
Br
O
O
O
CH3
f1 (ppm)

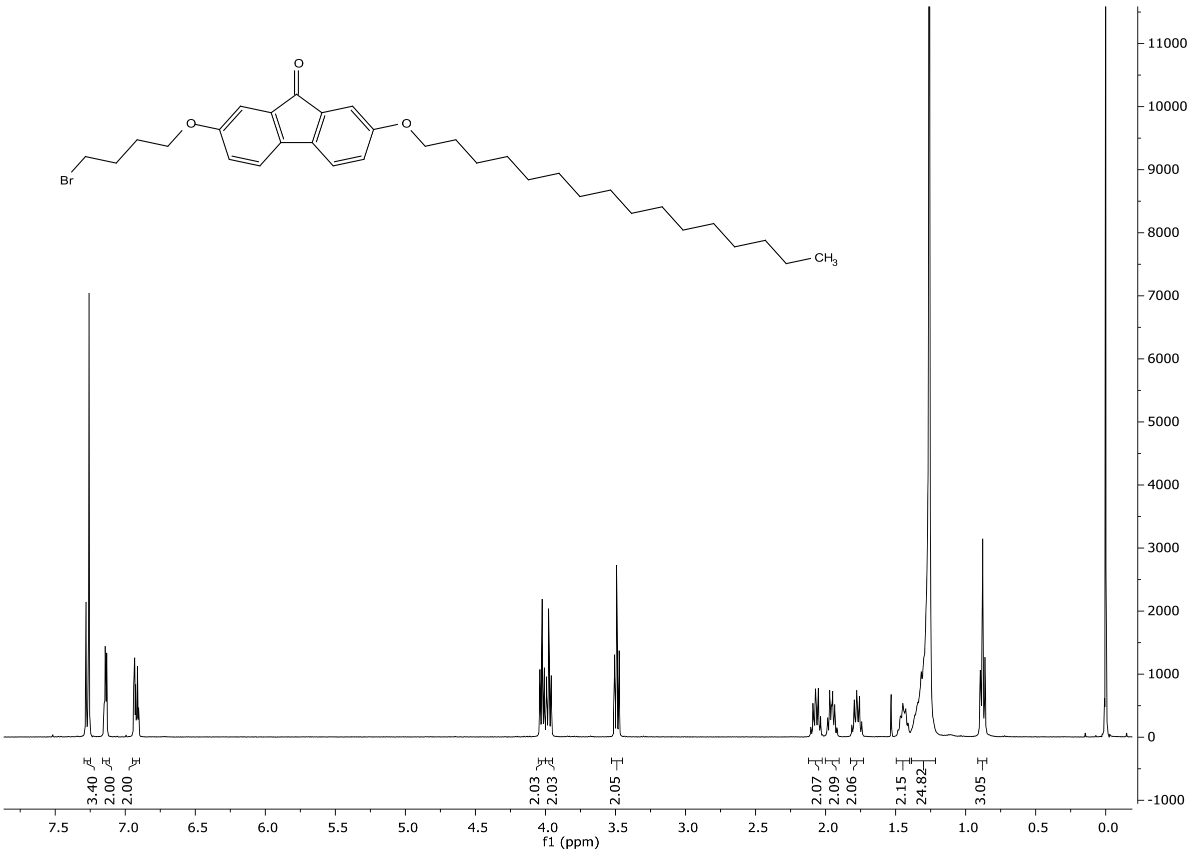
Br
O
O
O
CH3
3.40
2.00
2.00
2.03
2.03
2.05
2.07
2.09
2.06
2.15
24.82
3.05
f1 (ppm)

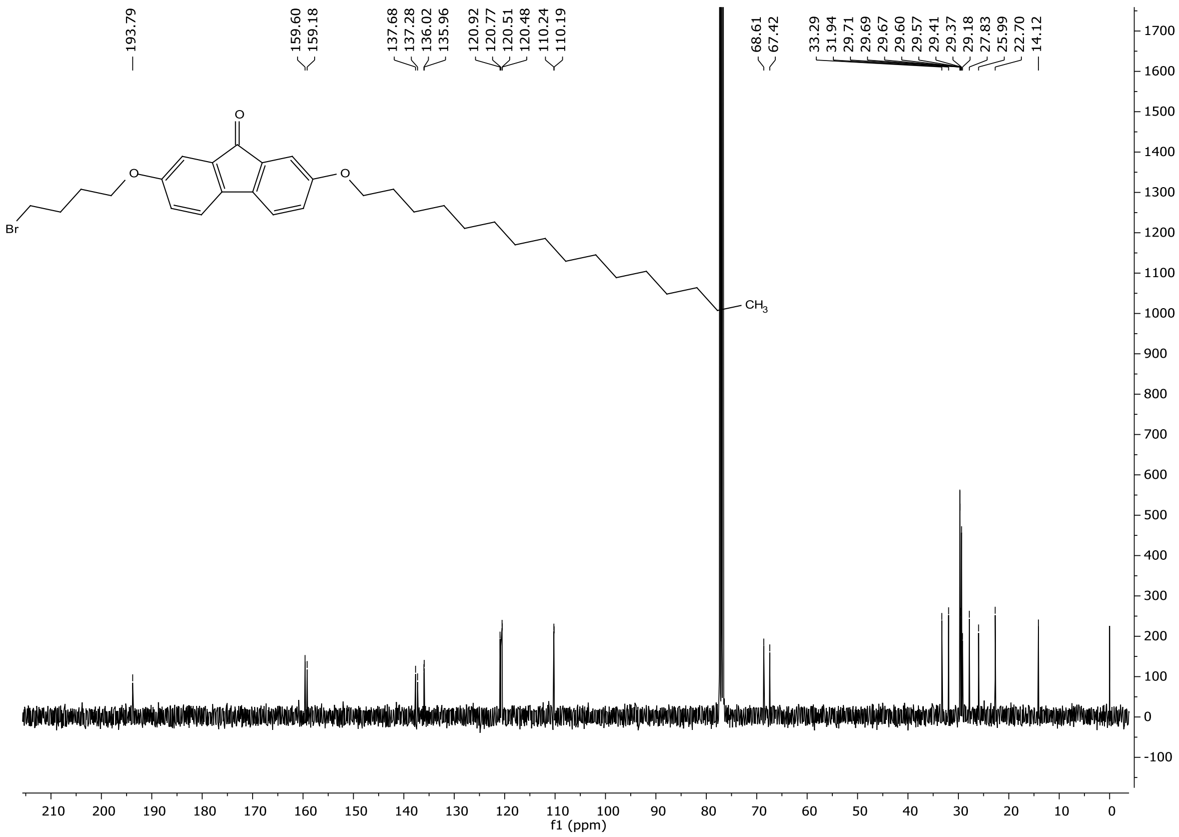
193.79
159.60
159.18
137.68
137.28
136.02
135.96
120.92
120.77
120.51
120.48
110.24
110.19
68.61
67.42
33.29
31.94
29.71
29.69
29.67
29.60
29.57
29.41
29.37
29.18
27.83
25.99
22.70
14.12
Br
O
O
O
CH3
f1 (ppm)

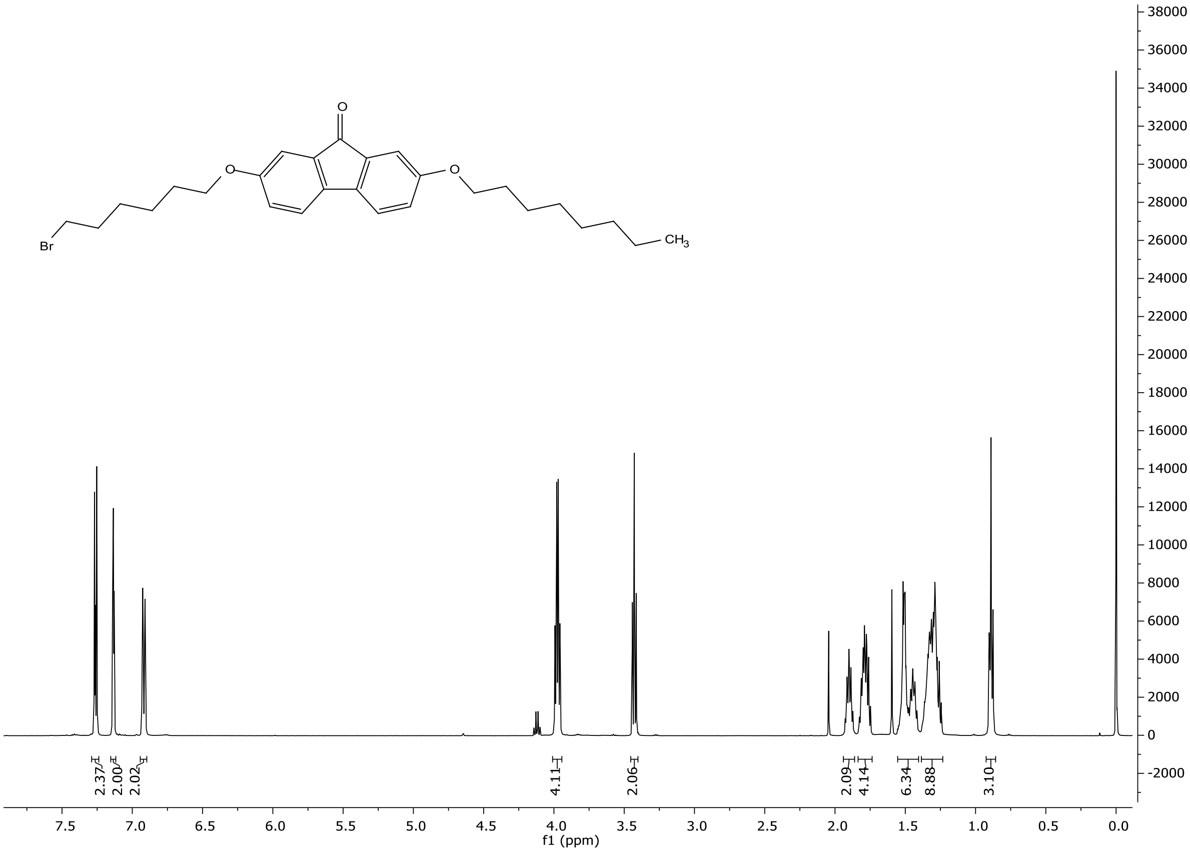

Br
O
O
O
CH3
2.37
2.00
2.02
4.11
2.06
2.09
4.14
6.34
8.88
3.10
f1 (ppm)

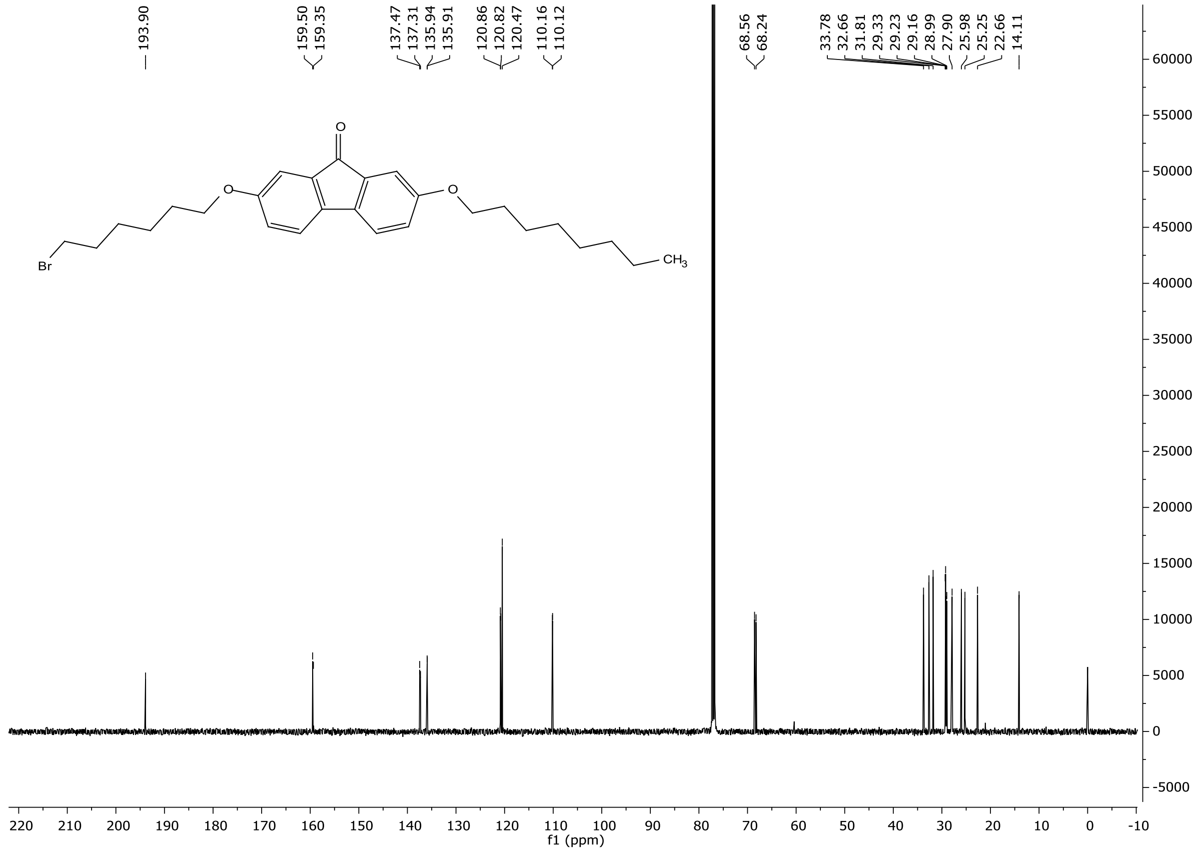

193.90
159.50
159.35
137.47
137.31
135.94
135.91
120.86
120.82
120.47
110.16
110.12
68.56
68.24
33.78
32.66
31.81
29.33
29.23
29.16
28.99
27.90
25.98
25.25
22.66
14.11
Br
O
O
O
CH3
f1 (ppm)

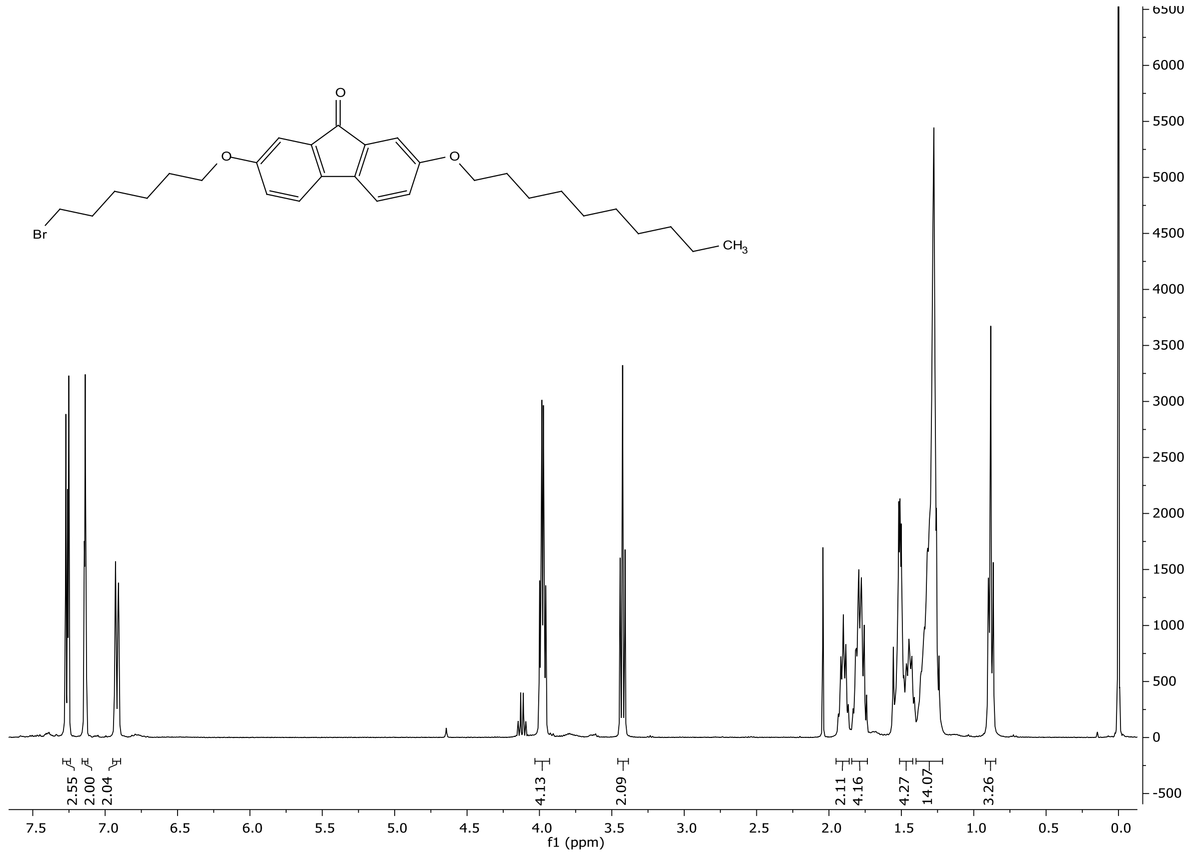

Br
O
O
O
CH3
2.55
2.00
2.04
4.13
2.09
2.11
4.16
4.27
14.07
3.26
f1 (ppm)

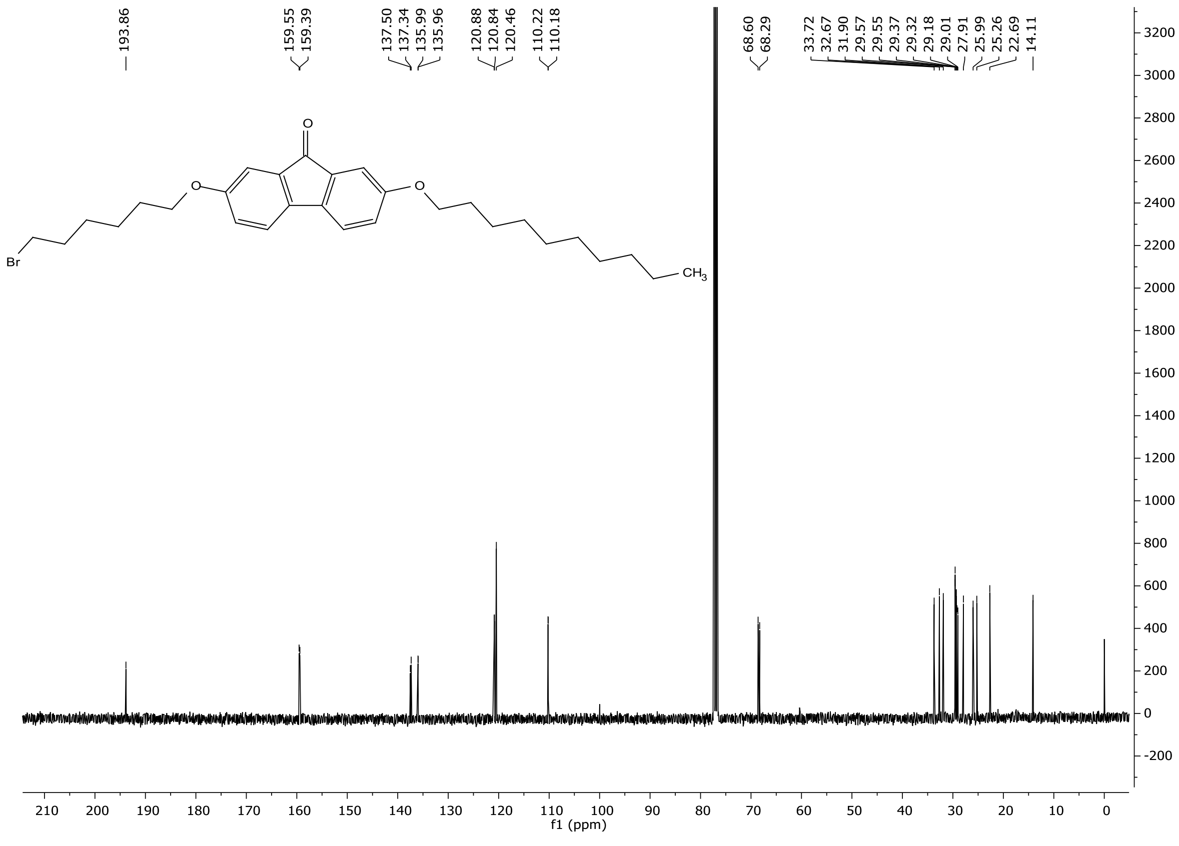

193.86
159.55
159.39
137.50
137.34
135.99
135.96
120.88
120.84
120.46
110.22
110.18
68.60
68.29
33.72
32.67
31.90
29.57
29.55
29.37
29.32
29.18
29.01
27.91
25.99
25.26
22.69
14.11
Br
O
O
O
CH3
f1 (ppm)

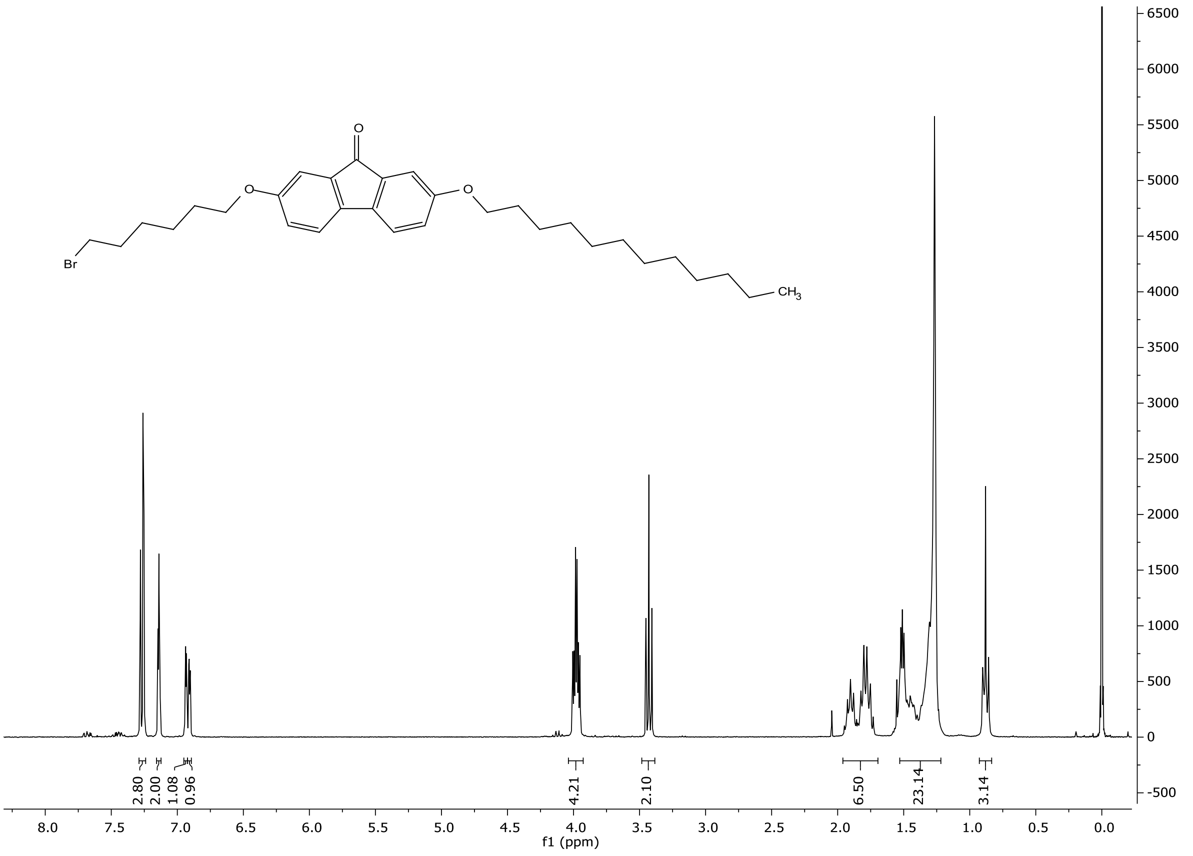

O
O
O
Br
CH_3
2.80
2.00
1.08
0.96
4.21
2.10
6.50
23.14
3.14
f1 (ppm)

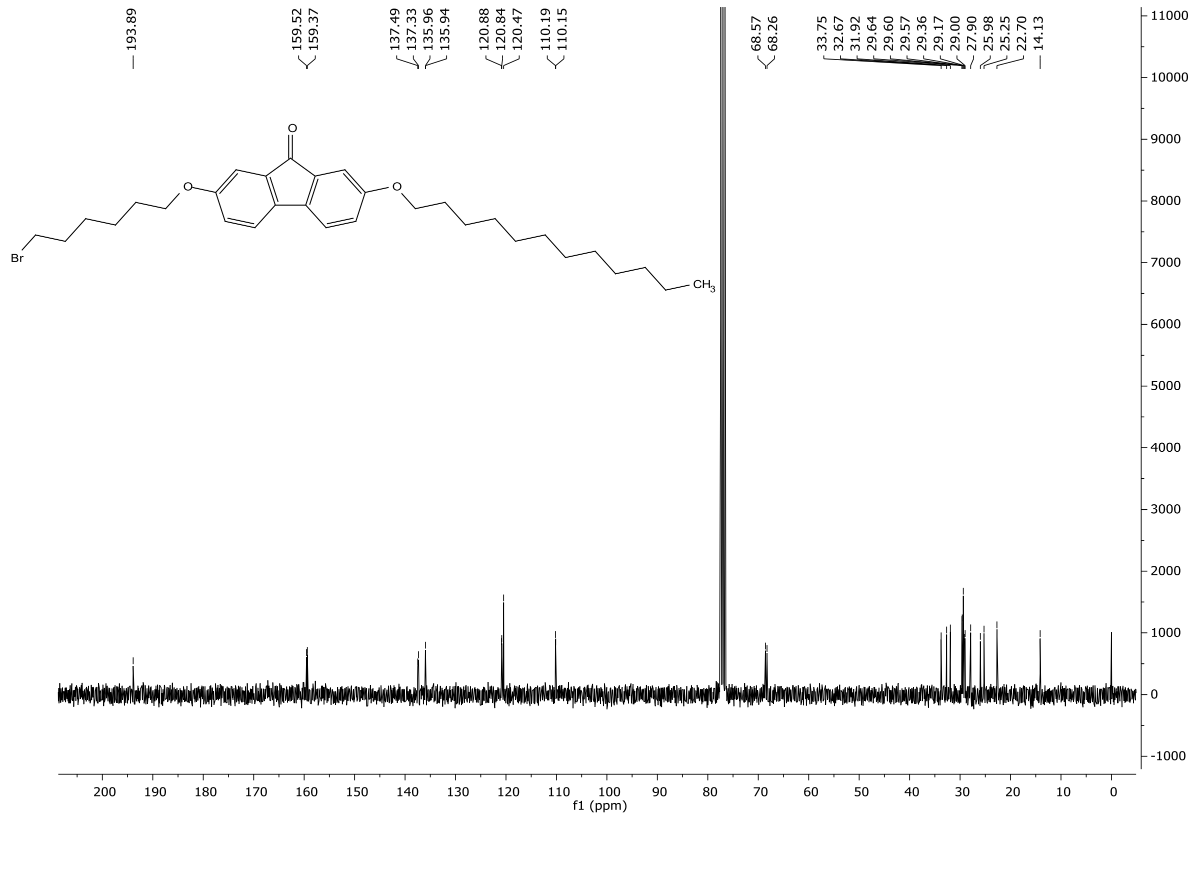

193.89
159.52
159.37
137.49
137.33
135.96
135.94
120.88
120.84
120.47
110.19
110.15
68.57
68.26
33.75
32.67
31.92
29.64
29.60
29.57
29.36
29.17
29.00
27.90
25.98
25.25
22.70
14.13
O
O
O
Br
CH_3
f1 (ppm)

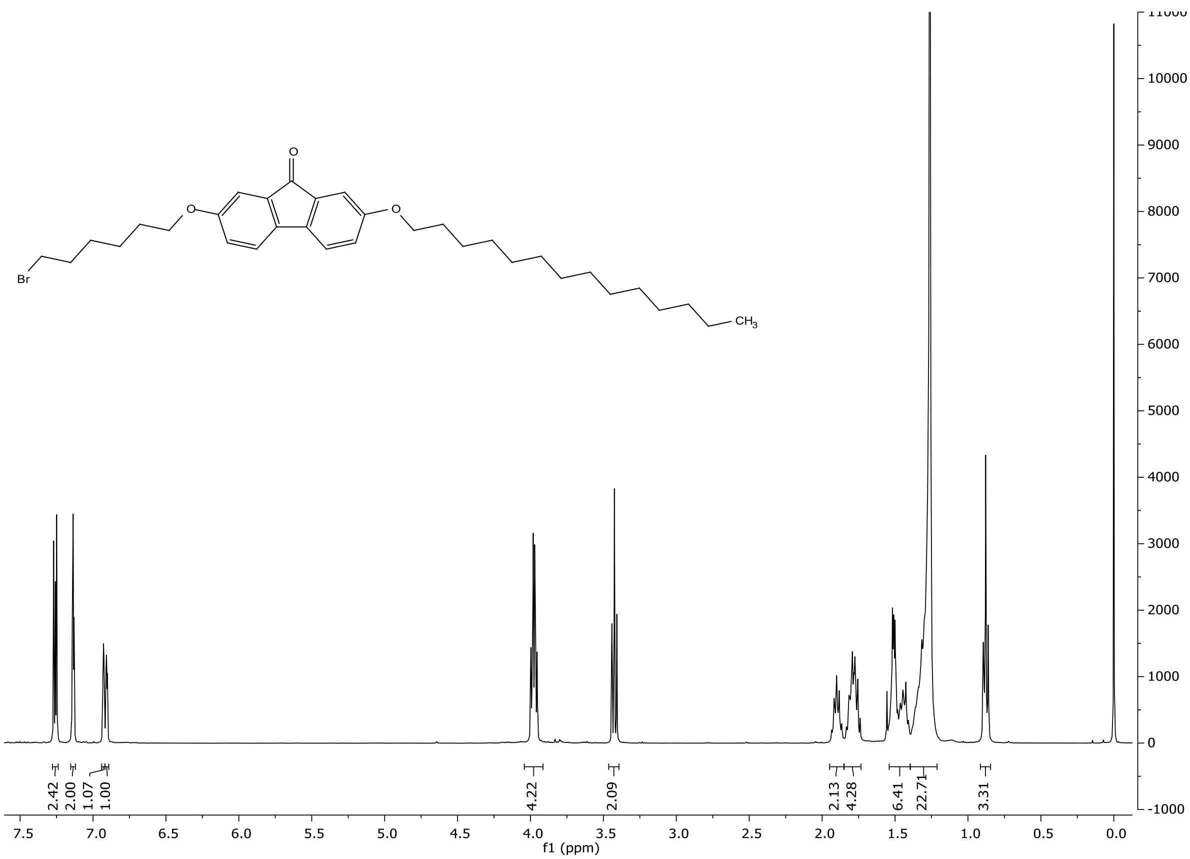
O
O
O
Br
CH3
2.42
2.00
1.07
1.00
4.22
2.09
2.13
4.28
6.41
22.71
3.31
f1 (ppm)

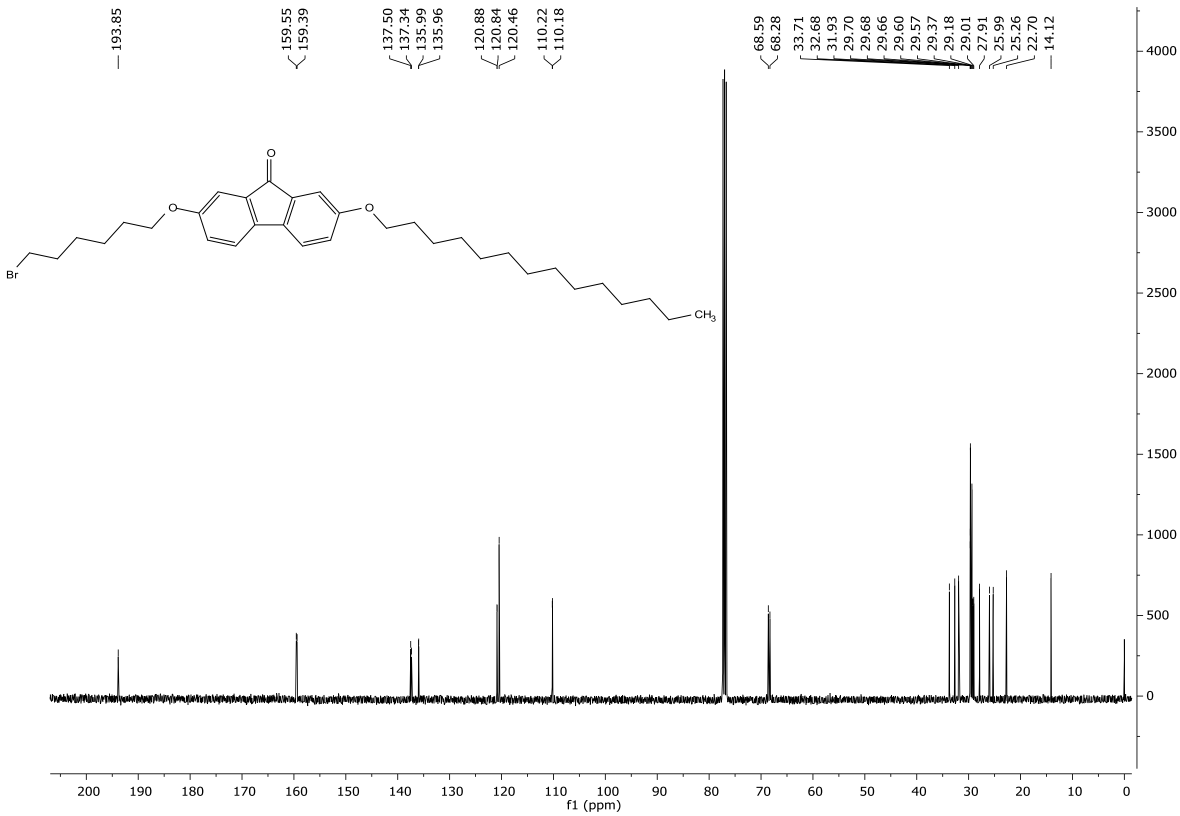
193.85
159.55
159.39
137.50
137.34
135.99
135.96
120.88
120.84
120.46
110.22
110.18
68.59
68.28
33.71
32.68
31.93
29.70
29.68
29.66
29.60
29.57
29.37
29.18
29.01
27.91
25.99
25.26
22.70
14.12
O
O
O
Br
CH3
f1 (ppm)

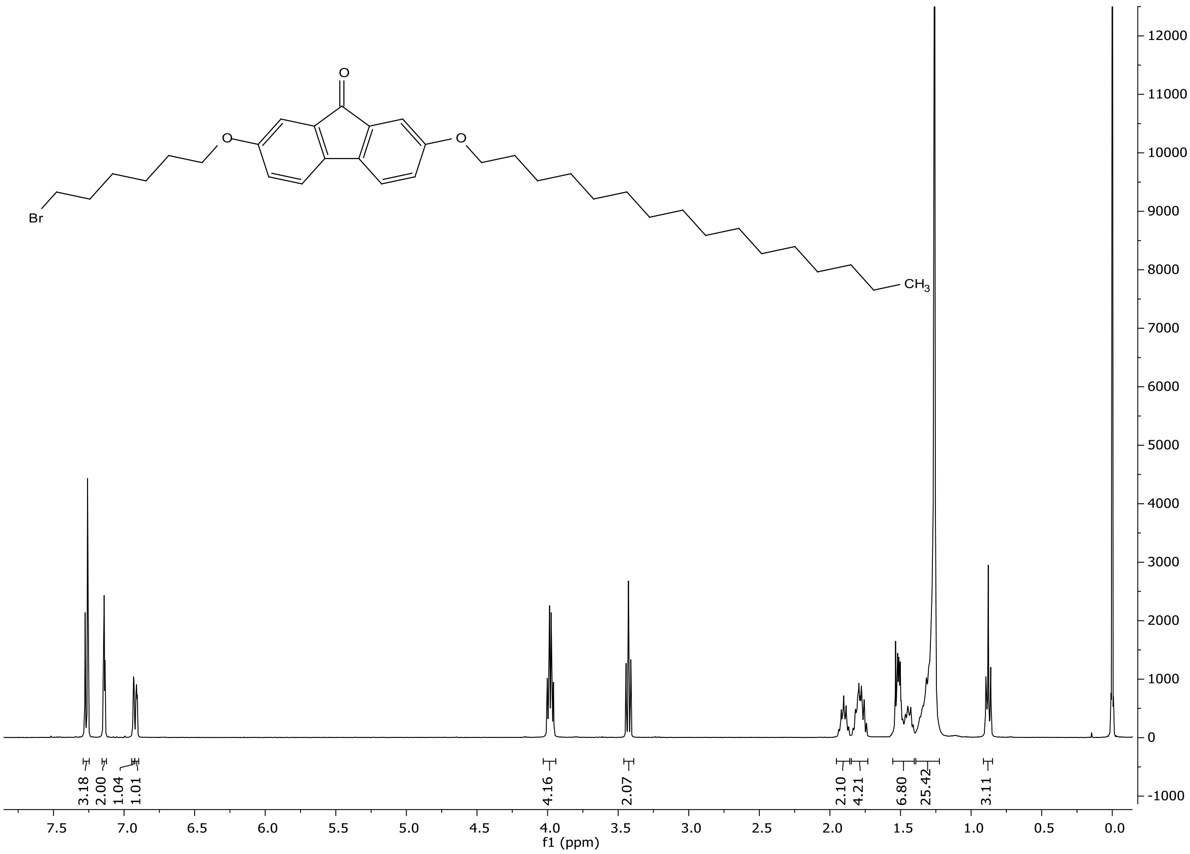

O
O
O
Br
CH3
3.18
2.00
1.04
1.01
4.16
2.07
2.10
4.21
6.80
25.42
3.11
f1 (ppm)

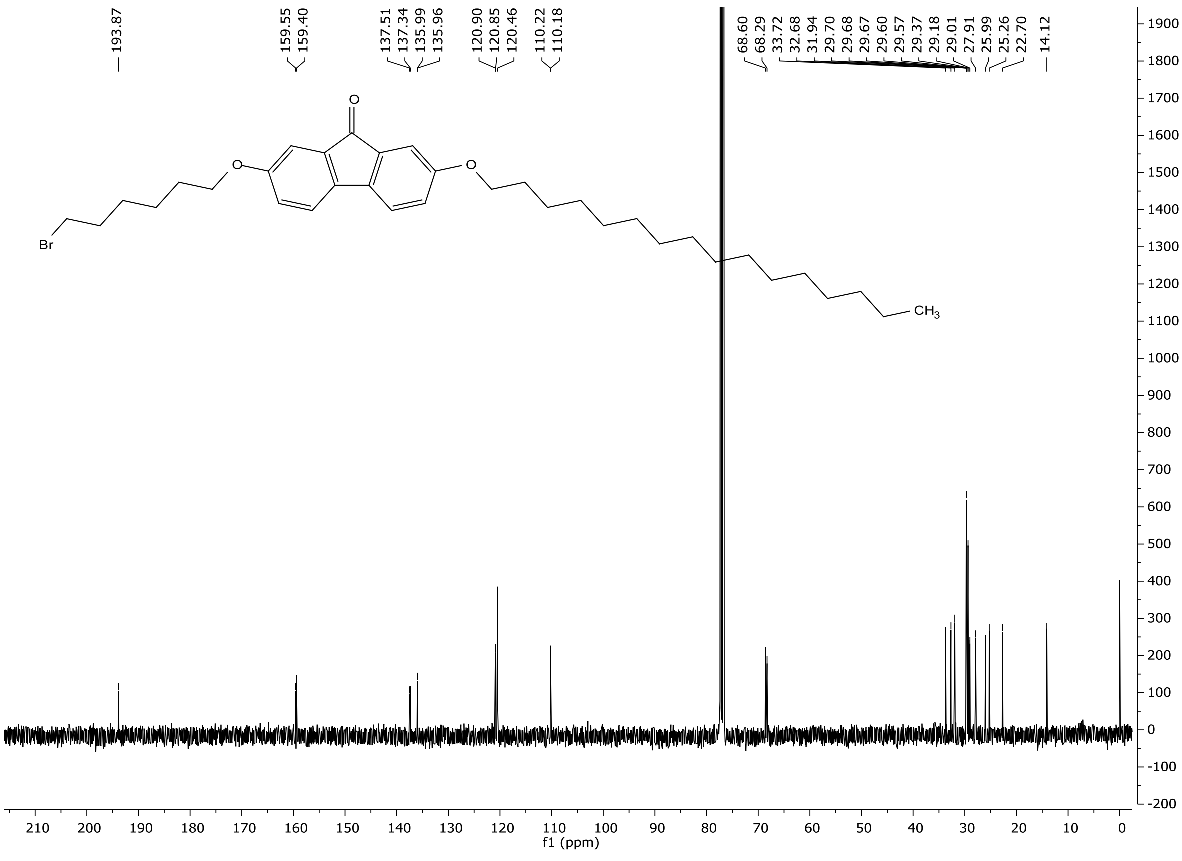

193.87
159.55
159.40
137.51
137.34
135.99
135.96
120.90
120.85
120.46
110.22
110.18
68.60
68.29
33.72
32.68
31.94
29.70
29.68
29.67
29.60
29.57
29.37
29.18
29.01
27.91
25.99
25.26
22.70
14.12
O
O
O
Br
CH3
f1 (ppm)

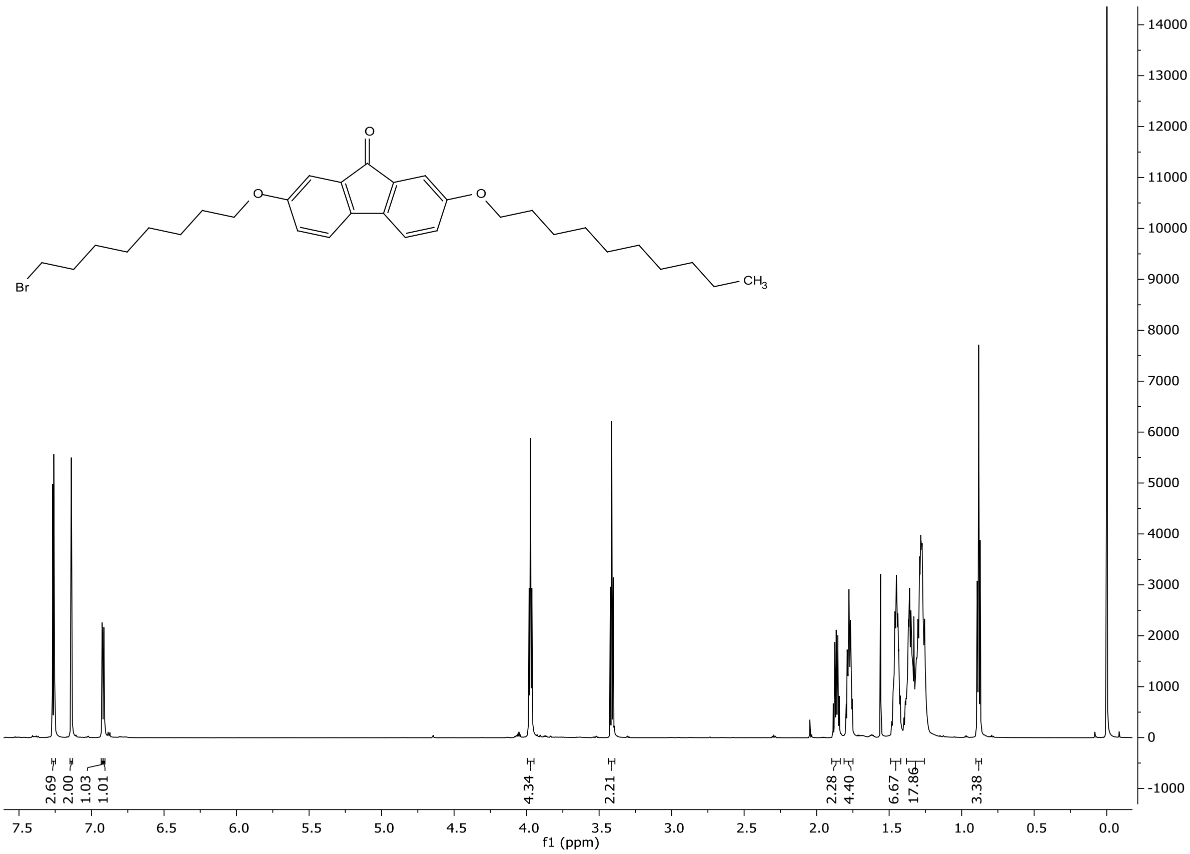

O
O
O
Br
CH3
2.69
2.00
1.03
1.01
4.34
2.21
2.28
4.40
6.67
17.86
3.38
f1 (ppm)

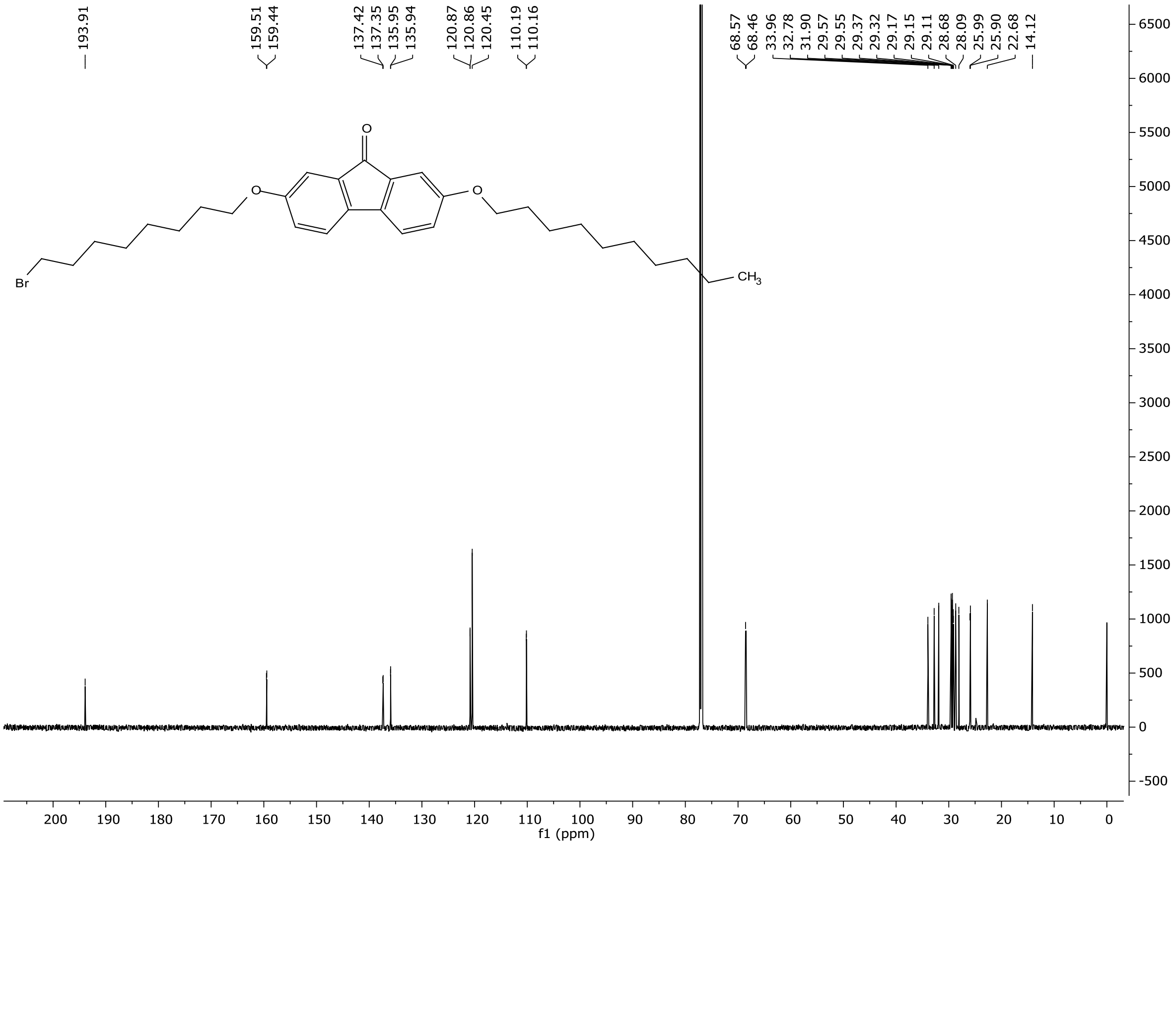

193.91
159.51
159.44
137.42
137.35
135.95
135.94
120.87
120.86
120.45
110.19
110.16
68.57
68.46
33.96
32.78
31.90
29.57
29.55
29.37
29.32
29.17
29.15
29.11
28.68
28.09
25.99
25.90
22.68
14.12
O
O
O
Br
CH3
f1 (ppm)

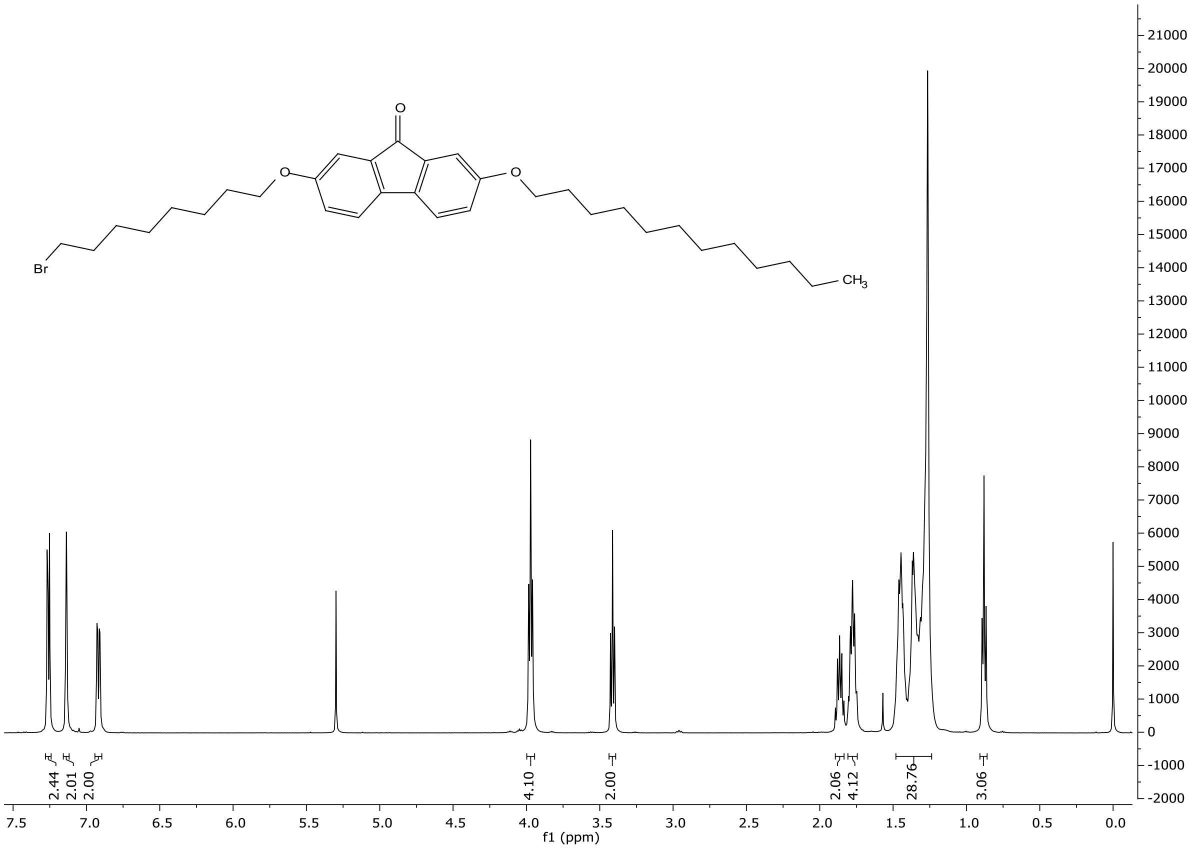
O
O
O
Br
CH3
2.44
2.01
2.00
4.10
2.00
2.06
4.12
28.76
3.06
f1 (ppm)

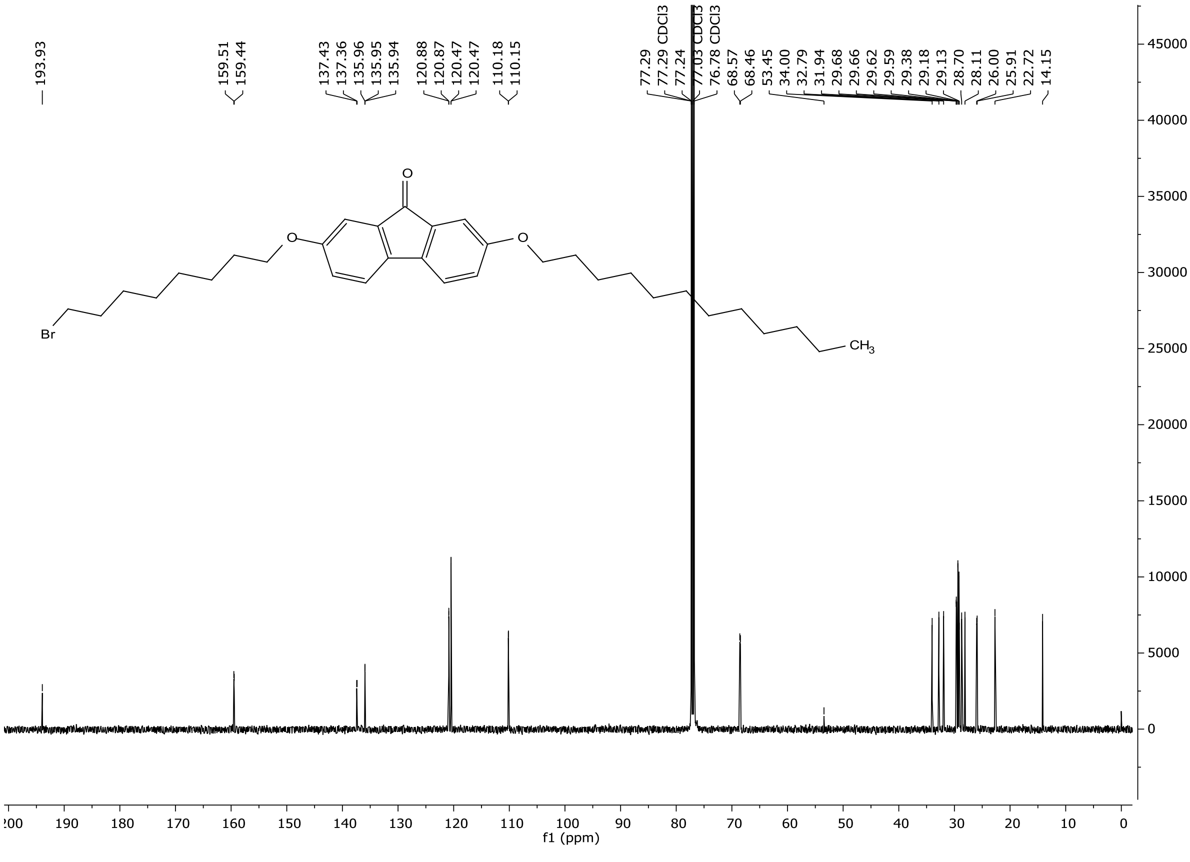
193.93
159.51
159.44
137.43
137.36
135.96
135.95
135.94
120.88
120.87
120.47
120.47
110.18
110.15
77.29
77.29 CDCl3
77.24
77.03 CDCl3
76.78 CDCl3
68.57
68.46
53.45
34.00
32.79
31.94
29.68
29.66
29.62
29.59
29.38
29.18
29.13
28.70
28.11
26.00
25.91
22.72
14.15
O
O
O
Br
CH3
f1 (ppm)

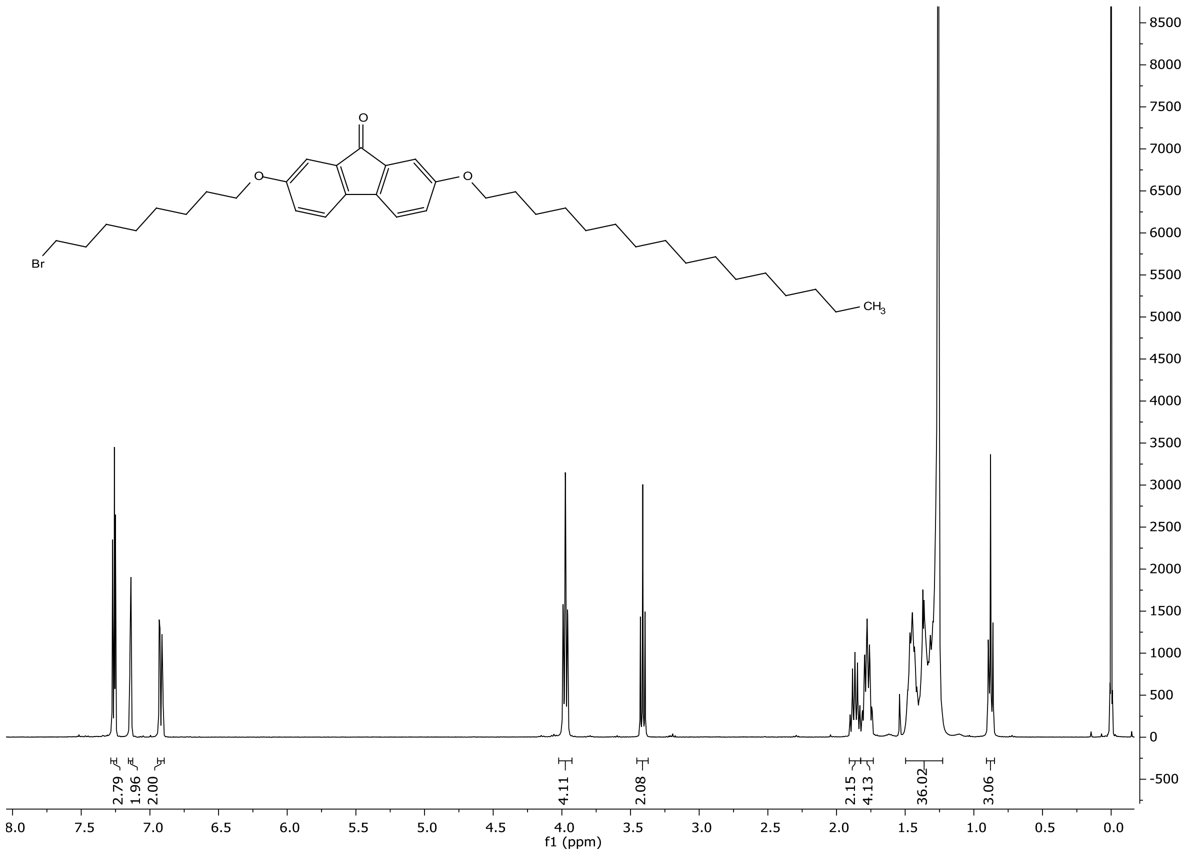
O
Br
CH3
2.79
1.96
2.00
4.11
2.08
2.15
4.13
36.02
3.06
f1 (ppm)

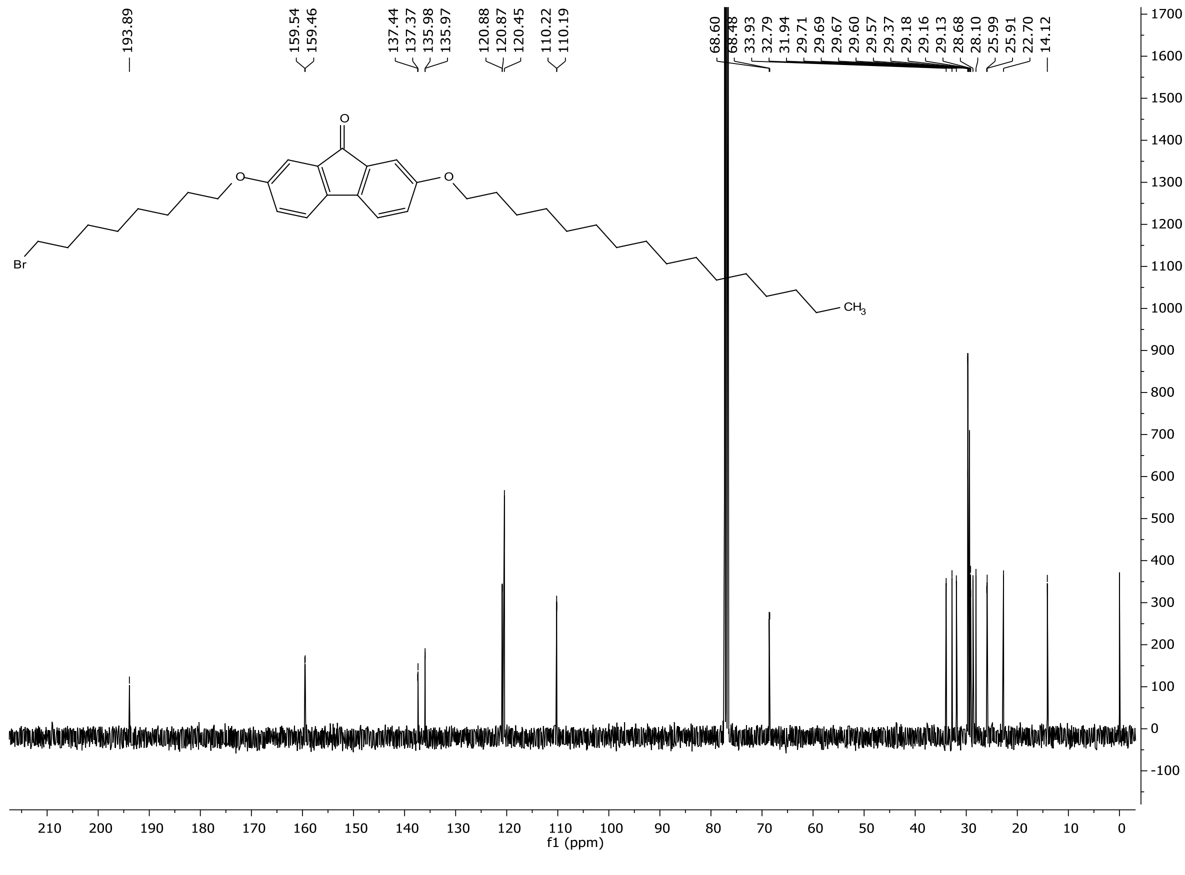
193.89
159.54
159.46
137.44
137.37
135.98
135.97
120.88
120.87
120.45
110.22
110.19
68.60
68.48
33.93
32.79
31.94
29.71
29.69
29.67
29.60
29.57
29.37
29.18
29.16
29.13
28.68
28.10
25.99
25.91
22.70
14.12
O
Br
CH3
f1 (ppm)

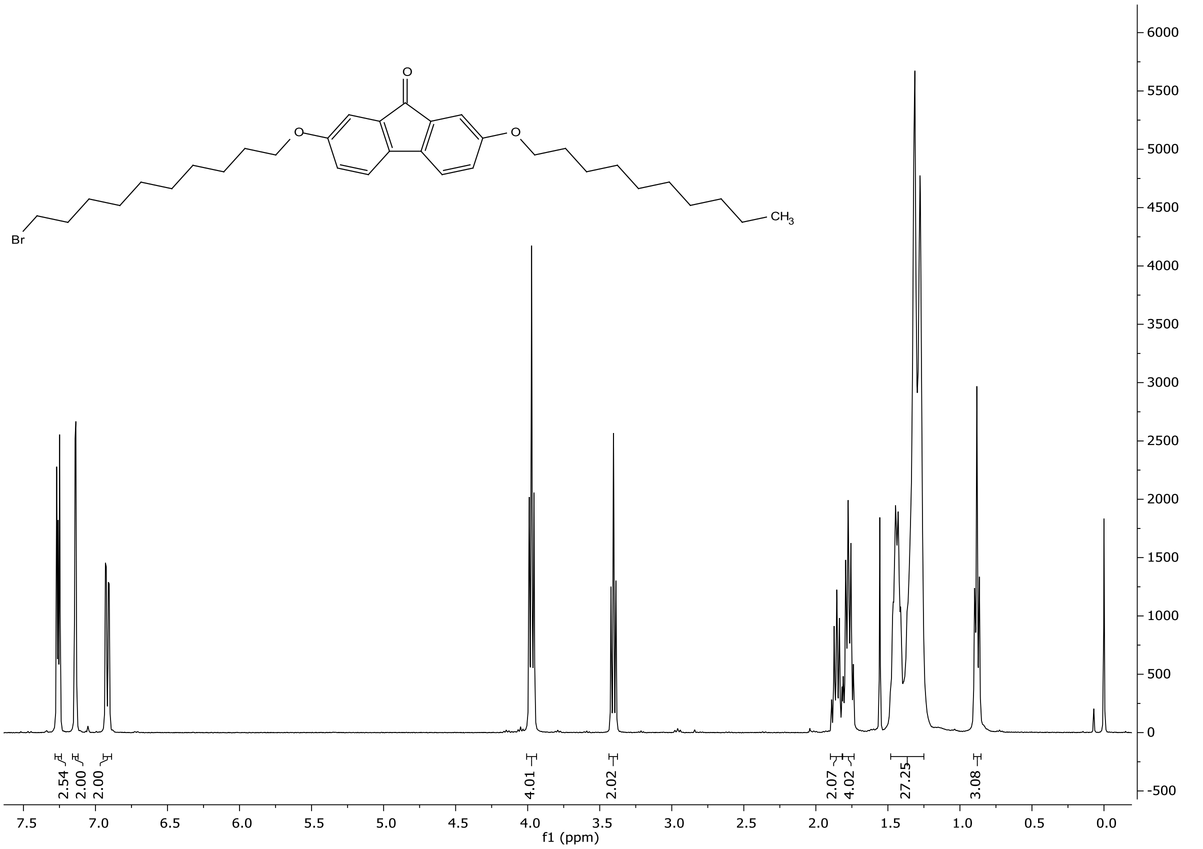

O
O
O
Br
CH3
2.54
2.00
2.00
4.01
2.02
2.07
4.02
27.25
3.08
7.5 7.0 6.5 6.0 5.5 5.0 4.5 4.0 3.5 3.0 2.5 2.0 1.5 1.0 0.5 0.0
f1 (ppm)
6000 5500 5000 4500 4000 3500 3000 2500 2000 1500 1000 500 0 -500

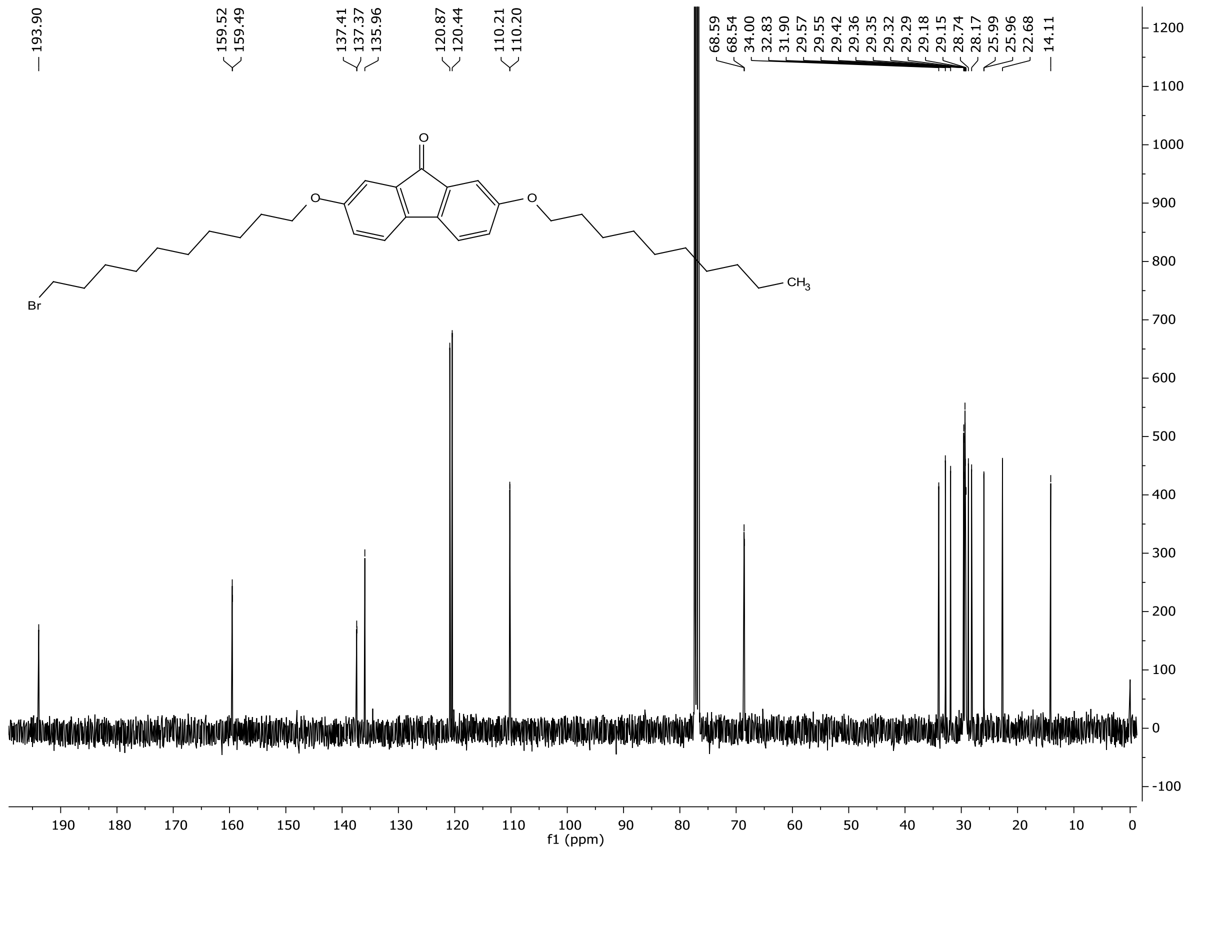

193.90
159.52
159.49
137.41
137.37
135.96
120.87
120.44
110.21
110.20
68.59
68.54
34.00
32.83
31.90
29.57
29.55
29.42
29.36
29.35
29.32
29.29
29.18
29.15
28.74
28.17
25.99
25.96
22.68
14.11
O
O
O
Br
CH3
190 180 170 160 150 140 130 120 110 100 90 80 70 60 50 40 30 20 10 0
f1 (ppm)
1200 1100 1000 900 800 700 600 500 400 300 200 100 0 -100

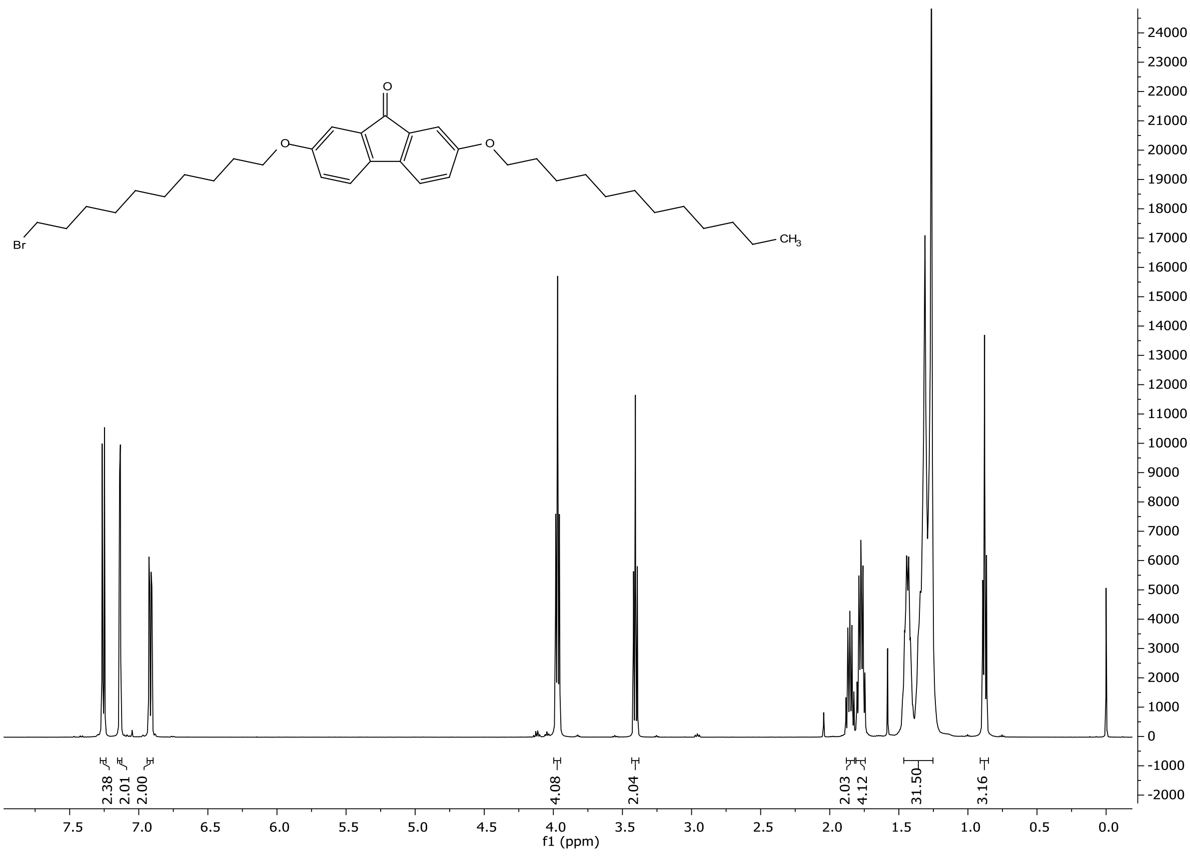
O
O
O
Br
CH3
2.38
2.01
2.00
4.08
2.04
2.03
4.12
31.50
3.16
f1 (ppm)

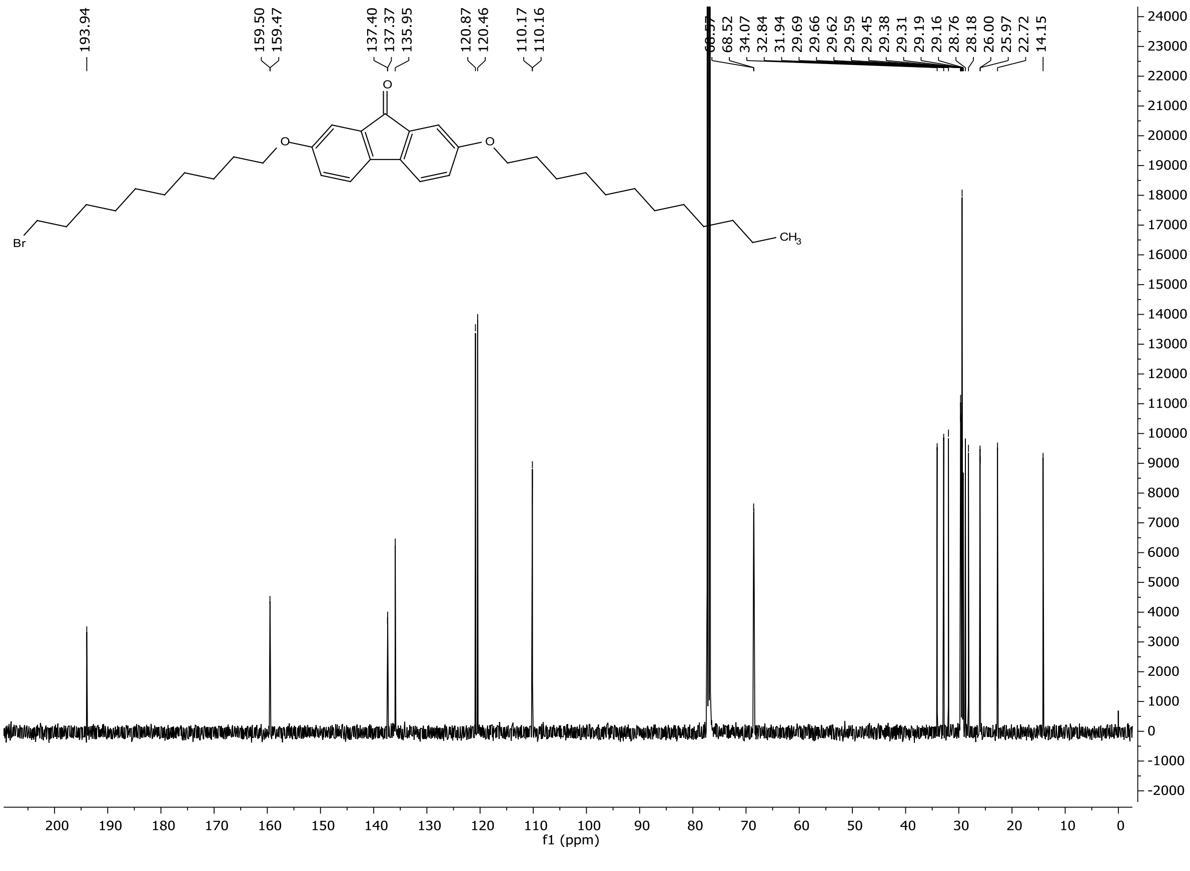
193.94
159.50
159.47
137.40
137.37
135.95
120.87
120.46
110.17
110.16
68.52
34.07
32.84
31.94
29.69
29.66
29.62
29.59
29.45
29.38
29.31
29.19
29.16
28.76
28.18
26.00
25.97
22.72
14.15
O
O
O
Br
CH3
f1 (ppm)

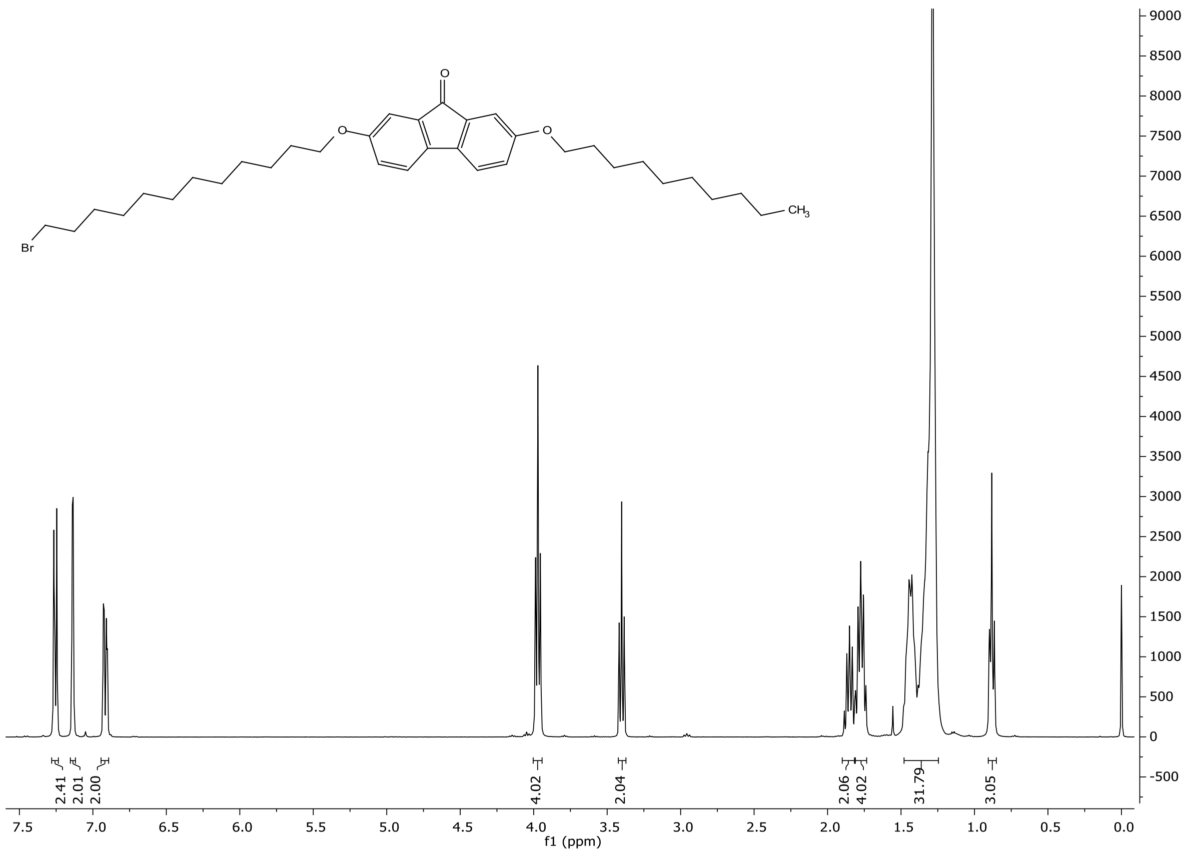

O
O
O
Br
CH3
2.41
2.01
2.00
4.02
2.04
2.06
4.02
31.79
3.05
7.5 7.0 6.5 6.0 5.5 5.0 4.5 4.0 3.5 3.0 2.5 2.0 1.5 1.0 0.5 0.0
f1 (ppm)
9000 8500 8000 7500 7000 6500 6000 5500 5000 4500 4000 3500 3000 2500 2000 1500 1000 500 0 -500

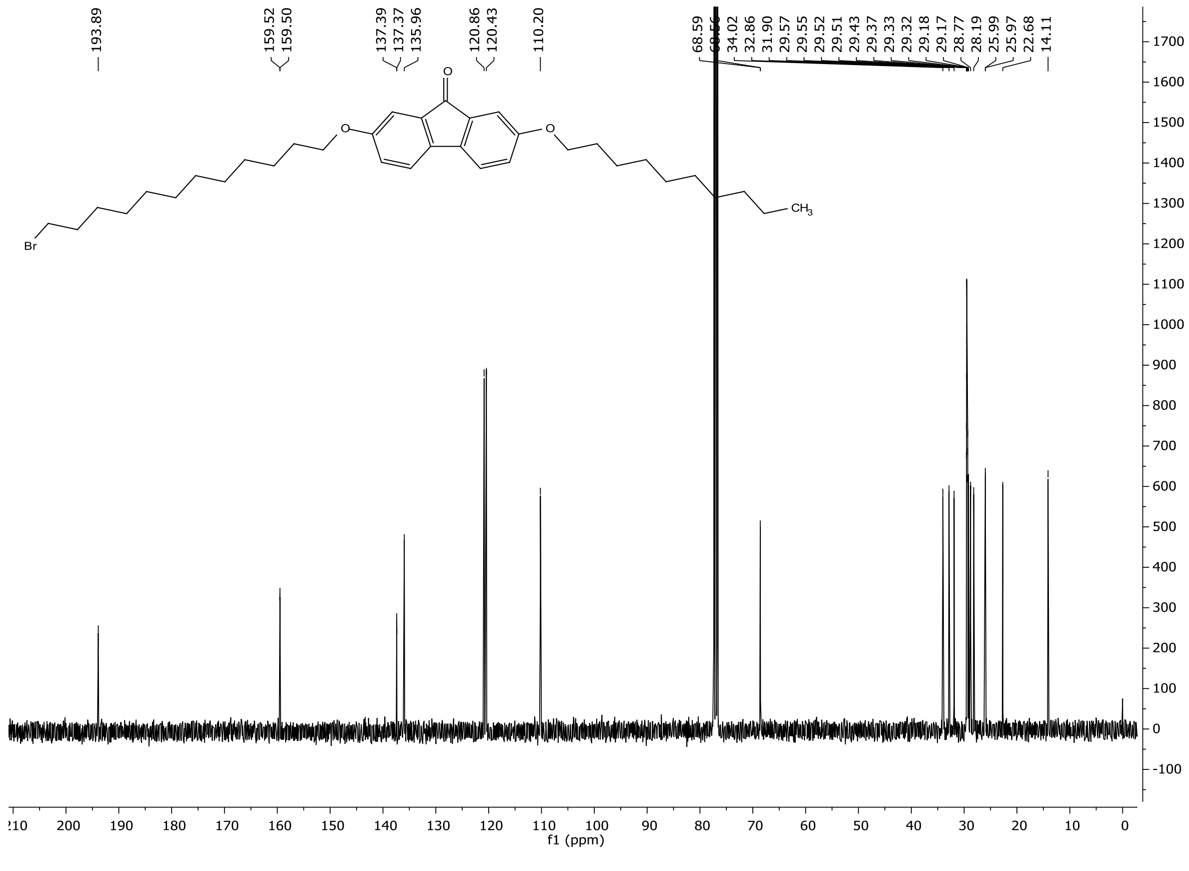

193.89
159.52
159.50
137.39
137.37
135.96
120.86
120.43
110.20
68.59
34.02
32.86
31.90
29.57
29.55
29.52
29.51
29.43
29.37
29.33
29.32
29.18
29.17
28.77
28.19
25.99
25.97
22.68
14.11
O
O
O
Br
CH3
210 200 190 180 170 160 150 140 130 120 110 100 90 80 70 60 50 40 30 20 10 0
f1 (ppm)
1700 1600 1500 1400 1300 1200 1100 1000 900 800 700 600 500 400 300 200 100 0 -100

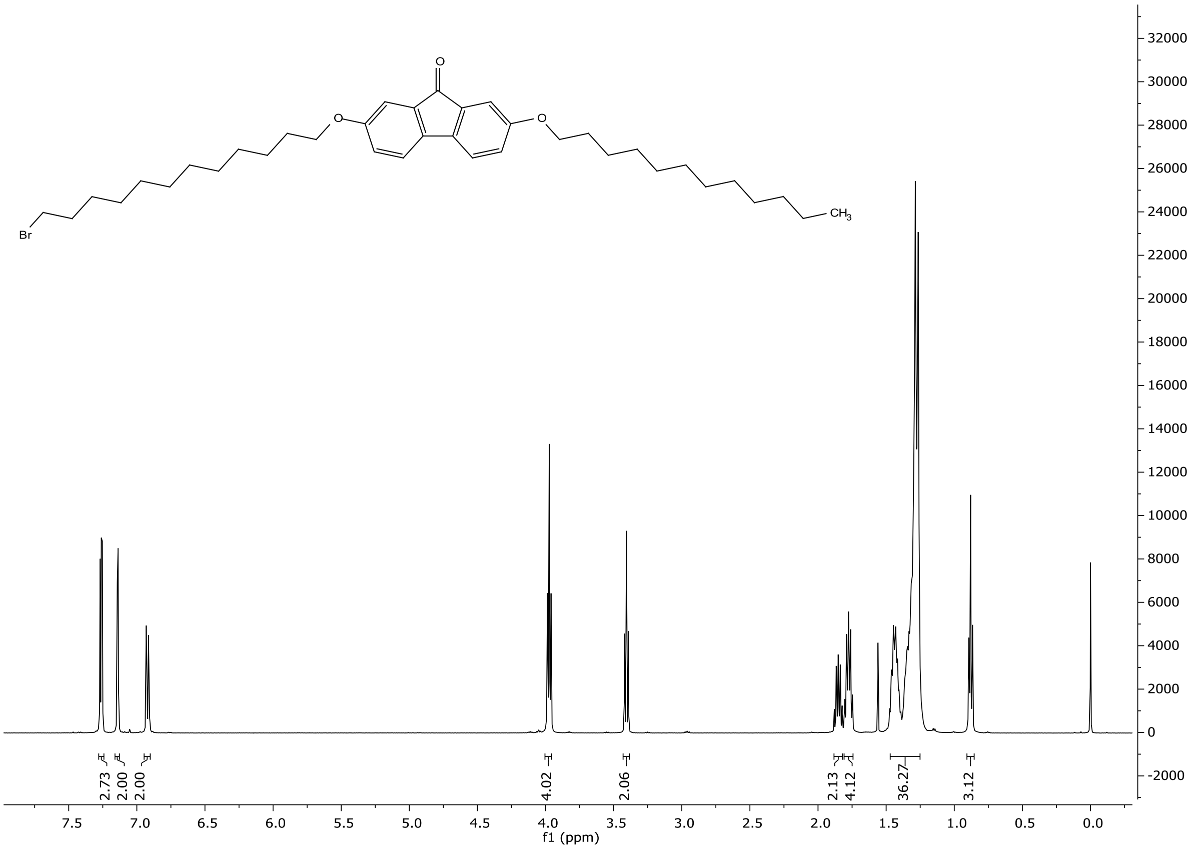

O
O
O
Br
CH3
2.73
2.00
2.00
4.02
2.06
2.13
4.12
36.27
3.12
f1 (ppm)

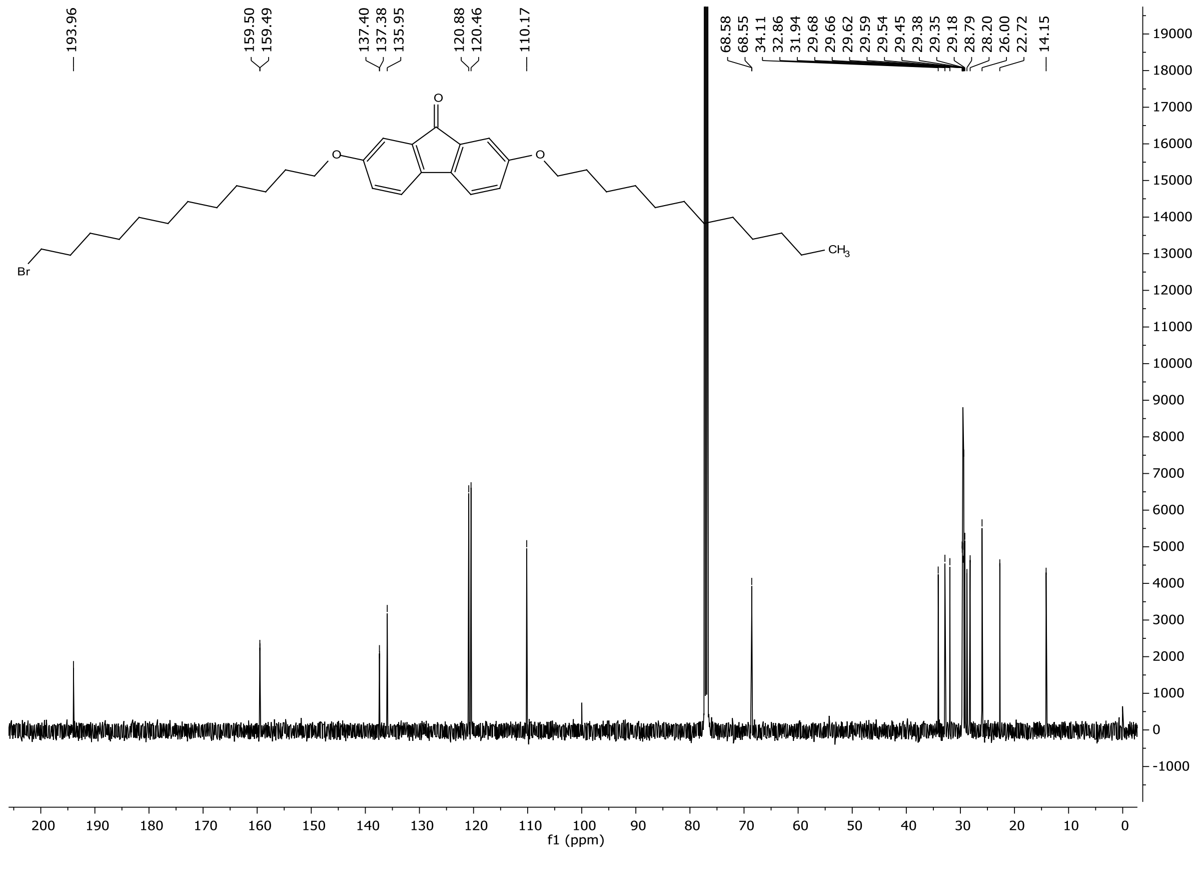

193.96
159.50
159.49
137.40
137.38
135.95
120.88
120.46
110.17
68.58
68.55
34.11
32.86
31.94
29.68
29.66
29.62
29.59
29.54
29.45
29.38
29.35
29.18
28.79
28.20
26.00
22.72
14.15
O
O
O
Br
CH3
f1 (ppm)

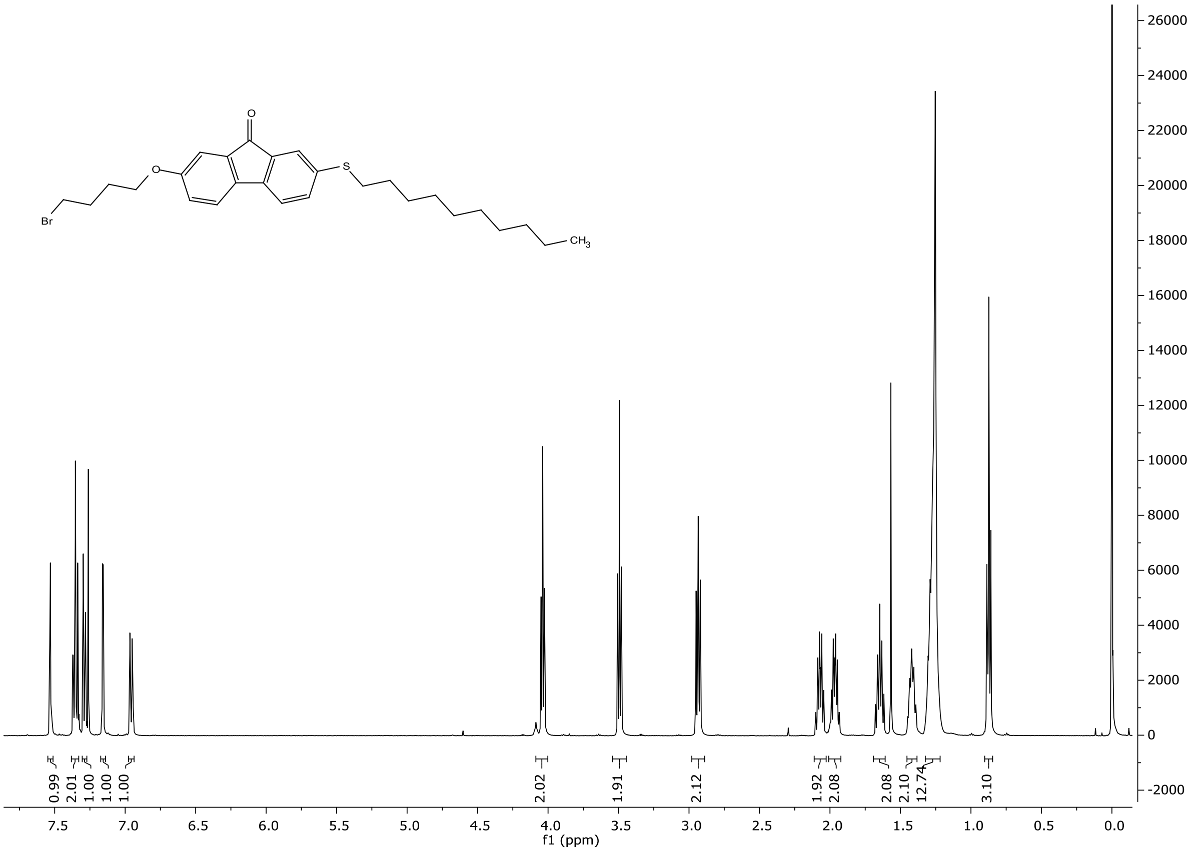
O
O
S
Br
CH3
0.99
2.01
1.00
1.00
1.00
2.02
1.91
2.12
1.92
2.08
2.08
2.10
12.74
3.10
f1 (ppm)

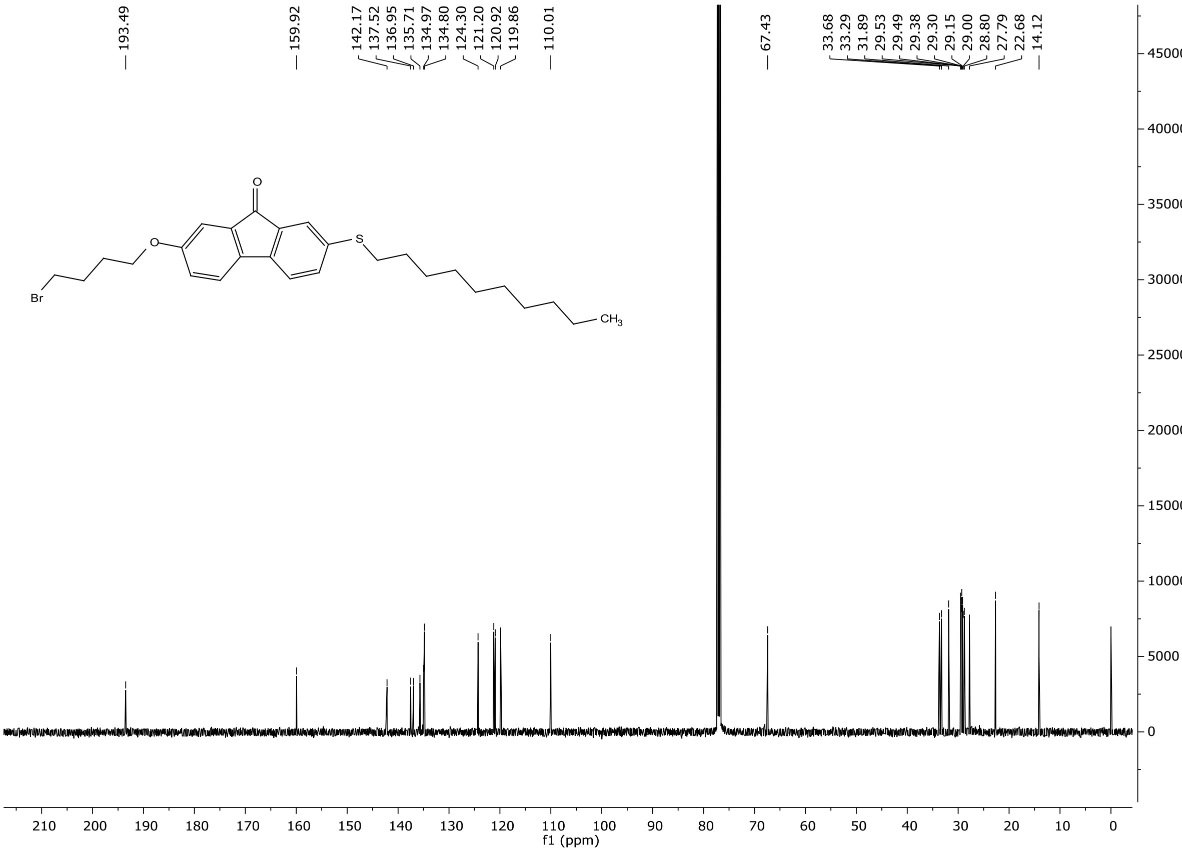
193.49
159.92
142.17
137.52
136.95
135.71
134.97
134.80
124.30
121.20
120.92
119.86
110.01
67.43
33.68
33.29
31.89
29.53
29.49
29.38
29.30
29.15
29.00
28.80
27.79
22.68
14.12
O
O
S
Br
CH3
f1 (ppm)

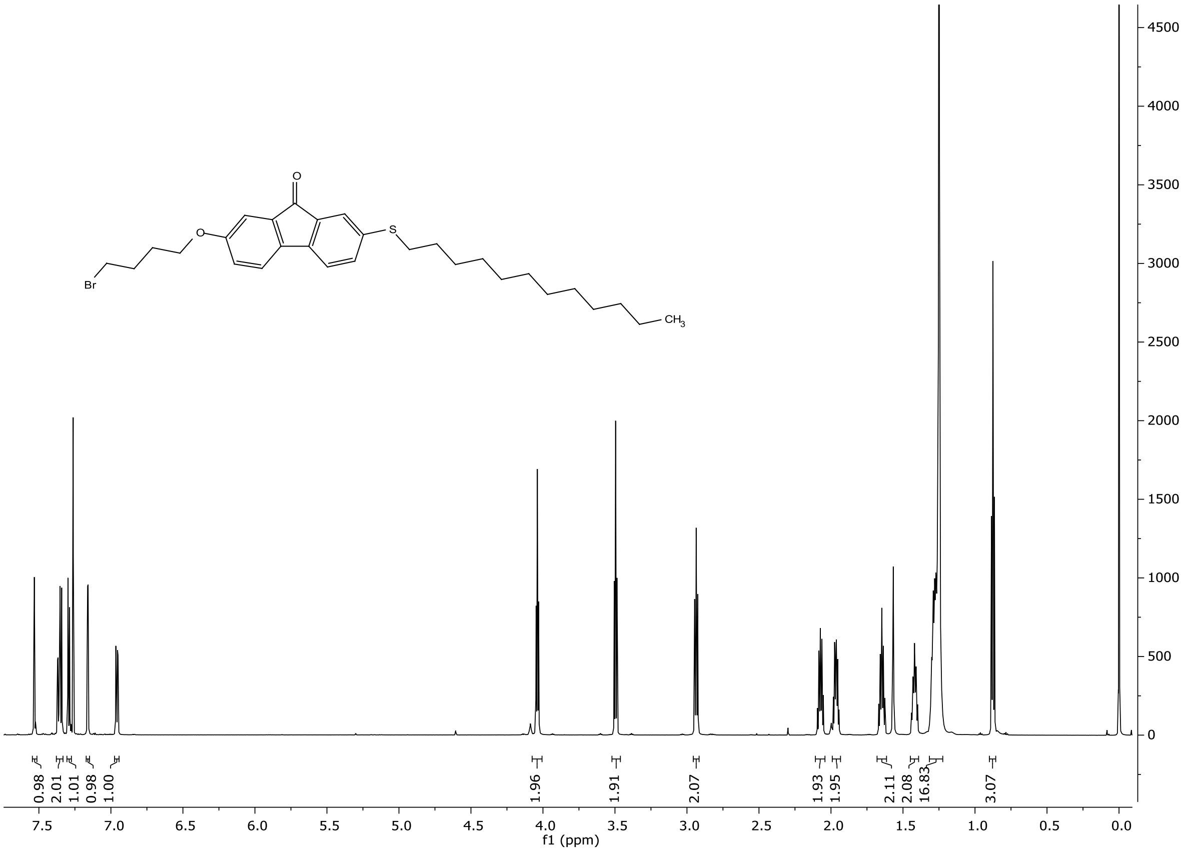

O
S
O
Br
CH3
0.98
2.01
1.01
0.98
1.00
1.96
1.91
2.07
1.93
1.95
2.11
2.08
16.83
3.07
f1 (ppm)

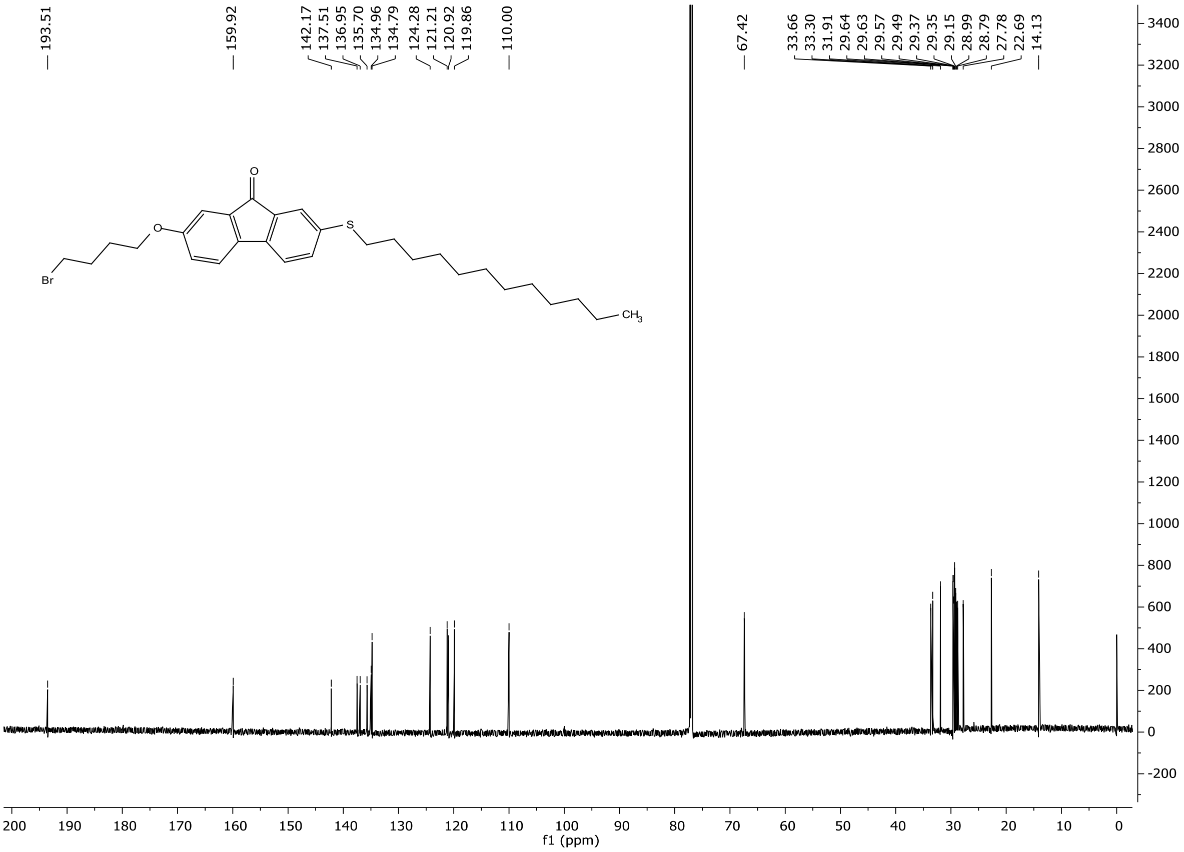

193.51
159.92
142.17
137.51
136.95
135.70
134.96
134.79
124.28
121.21
120.92
119.86
110.00
67.42
33.66
33.30
31.91
29.64
29.63
29.57
29.49
29.37
29.35
29.15
28.99
28.79
27.78
22.69
14.13
O
S
O
Br
CH3
f1 (ppm)

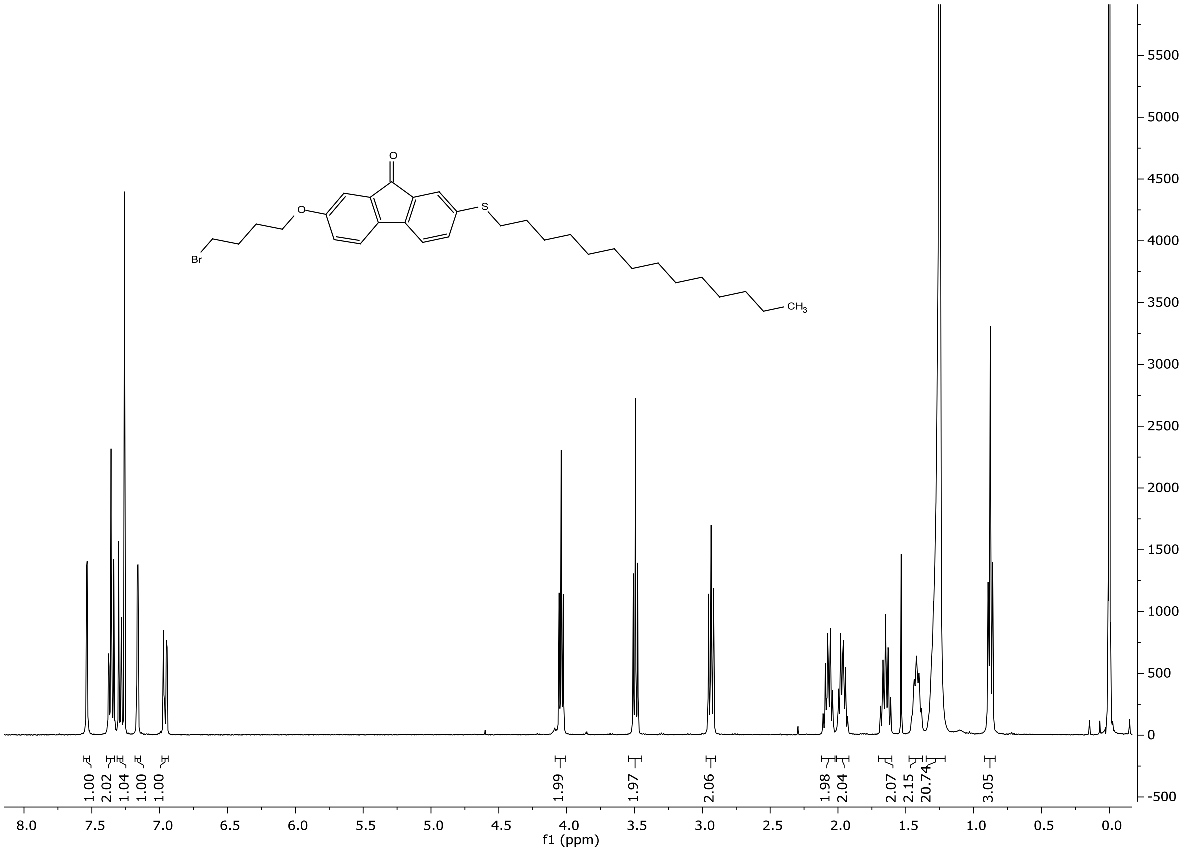

O
S
Br
CH3
1.00
2.02
1.04
1.00
1.00
1.99
1.97
2.06
1.98
2.04
2.07
2.15
20.74
3.05
f1 (ppm)

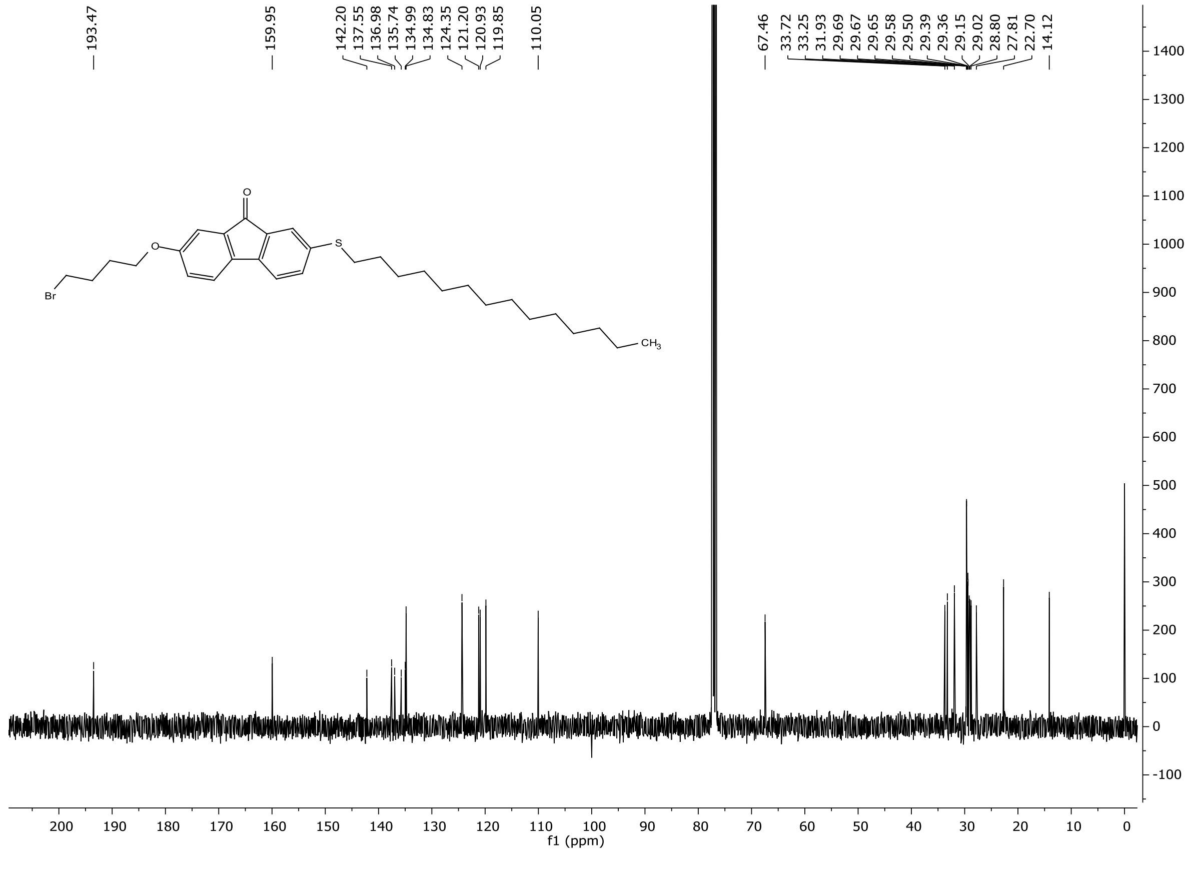

193.47
159.95
142.20
137.55
136.98
135.74
134.99
134.83
124.35
121.20
120.93
119.85
110.05
67.46
33.72
33.25
31.93
29.69
29.67
29.65
29.58
29.50
29.39
29.36
29.15
29.02
28.80
27.81
22.70
14.12
O
S
Br
CH3
f1 (ppm)

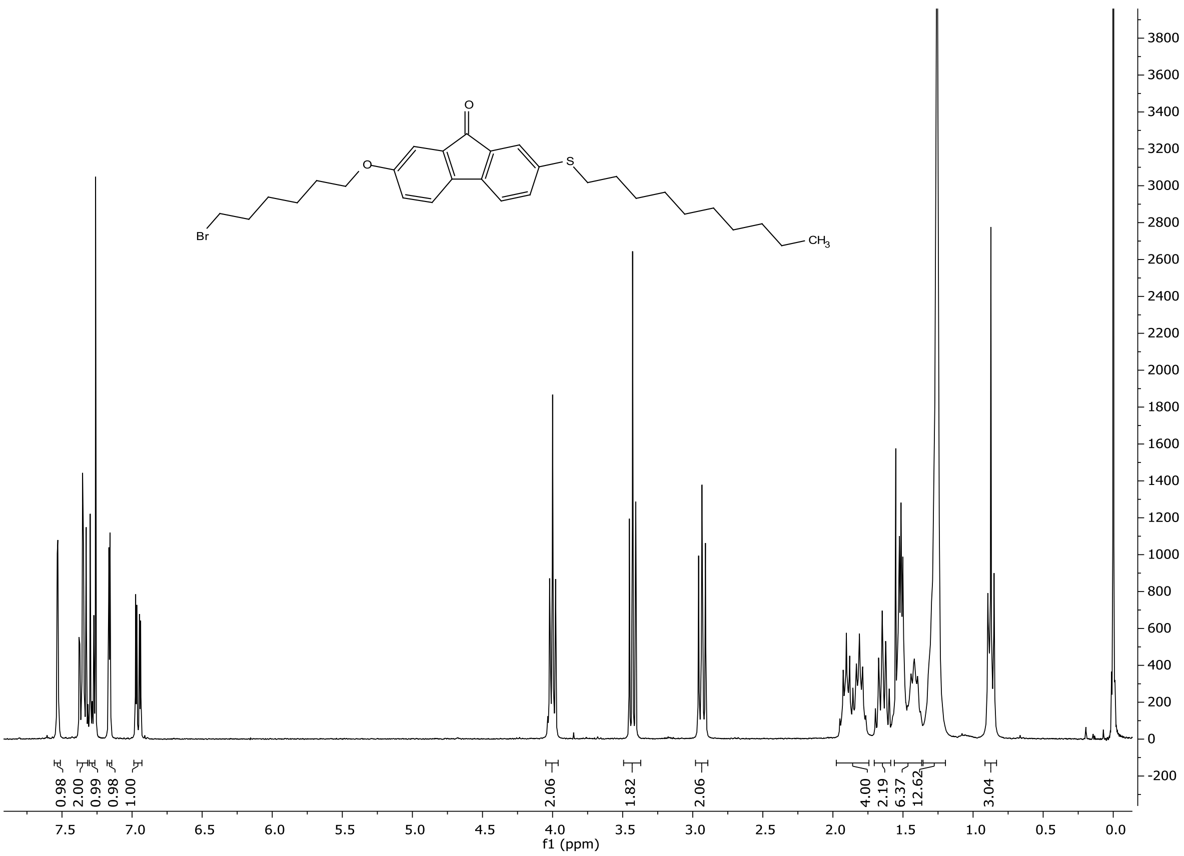
O
O
S
Br
CH3
0.98
2.00
0.99
0.98
1.00
2.06
1.82
2.06
4.00
2.19
6.37
12.62
3.04
f1 (ppm)

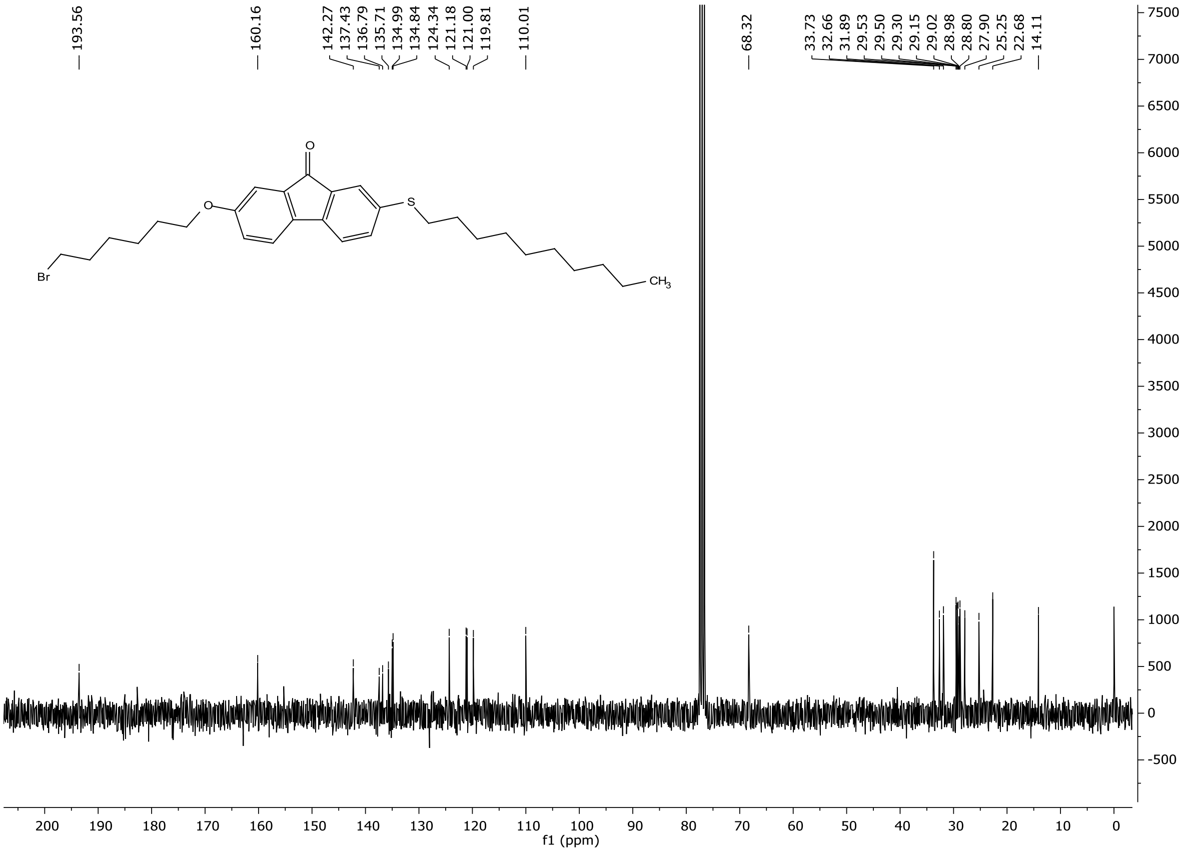
193.56
160.16
142.27
137.43
136.79
135.71
134.99
134.84
124.34
121.18
121.00
119.81
110.01
68.32
33.73
32.66
31.89
29.53
29.50
29.30
29.15
29.02
28.98
28.80
27.90
25.25
22.68
14.11
O
O
S
Br
CH3
f1 (ppm)

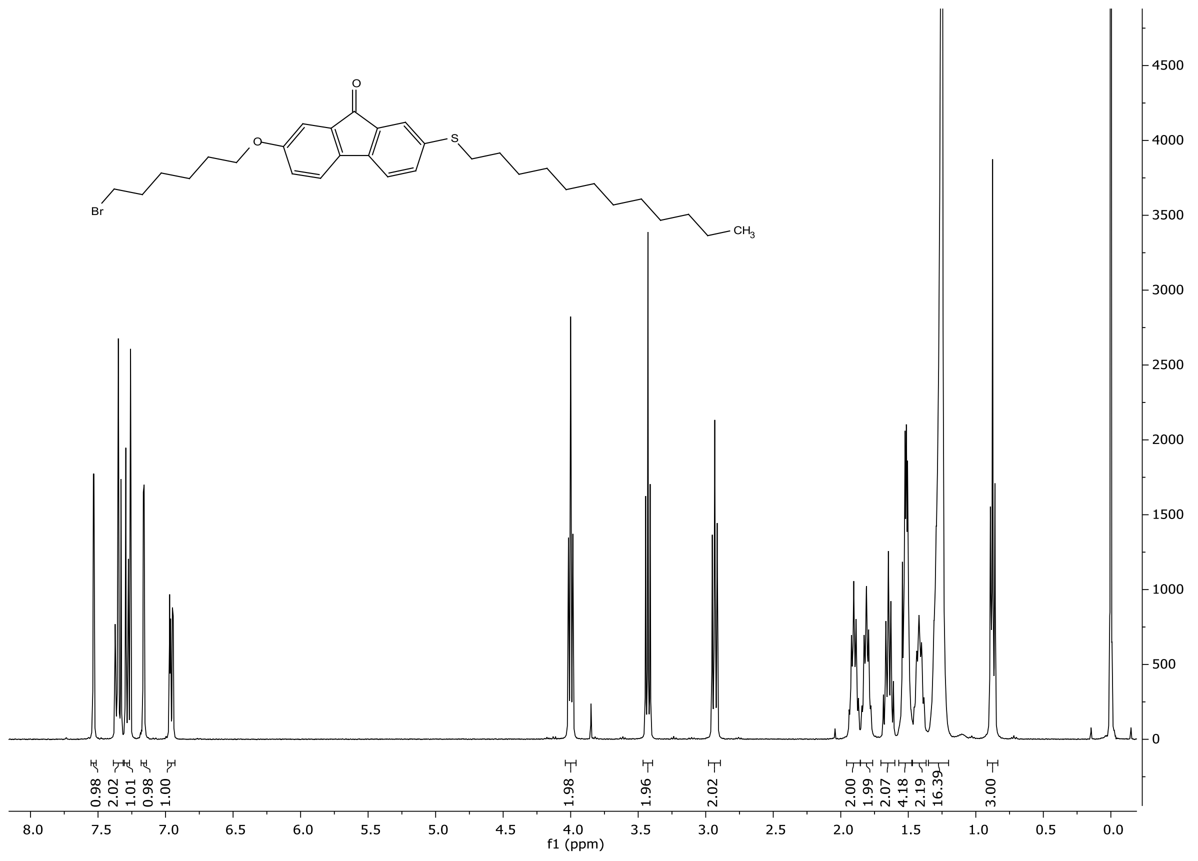

O
S
Br
CH3
0.98
2.02
1.01
0.98
1.00
1.98
1.96
2.02
2.00
1.99
2.07
4.18
2.19
16.39
3.00
f1 (ppm)

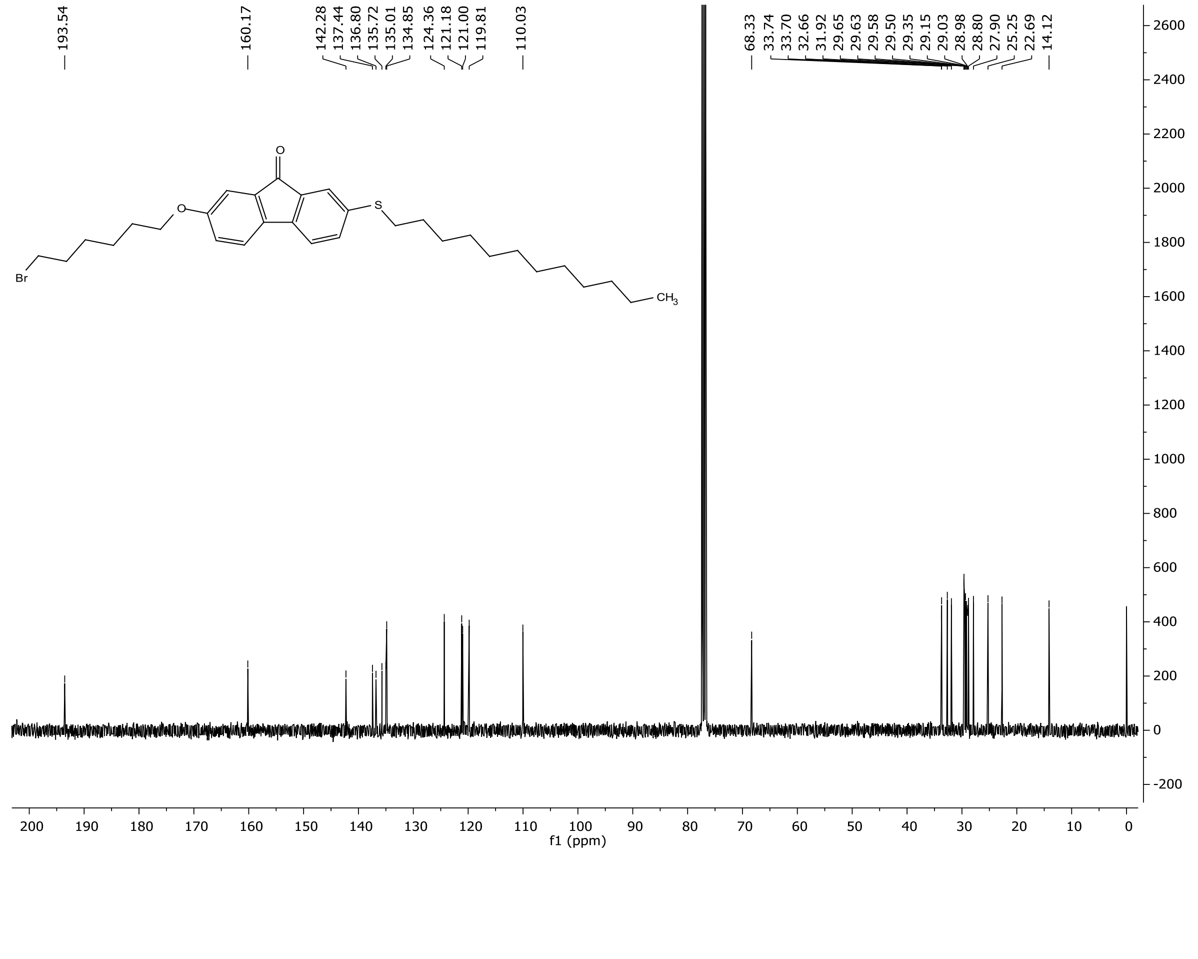

193.54
160.17
142.28
137.44
136.80
135.72
135.01
134.85
124.36
121.18
121.00
119.81
110.03
68.33
33.74
33.70
32.66
31.92
29.65
29.63
29.58
29.50
29.35
29.15
29.03
28.98
28.80
27.90
25.25
22.69
14.12
O
S
Br
CH3
f1 (ppm)

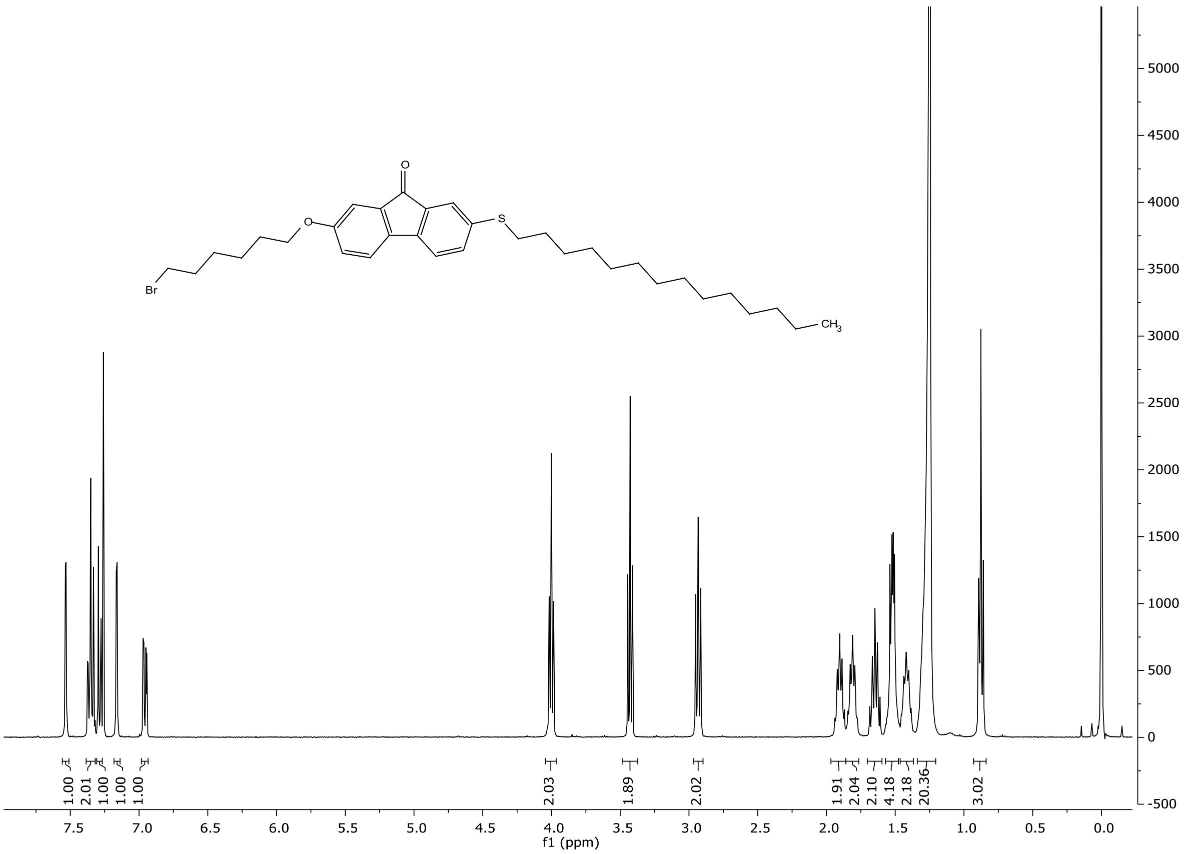

O
S
Br
CH3
1.00
2.01
1.00
1.00
1.00
2.03
1.89
2.02
1.91
2.04
2.10
4.18
2.18
20.36
3.02
7.5 7.0 6.5 6.0 5.5 5.0 4.5 4.0 3.5 3.0 2.5 2.0 1.5 1.0 0.5 0.0
f1 (ppm)
5000 4500 4000 3500 3000 2500 2000 1500 1000 500 0 -500

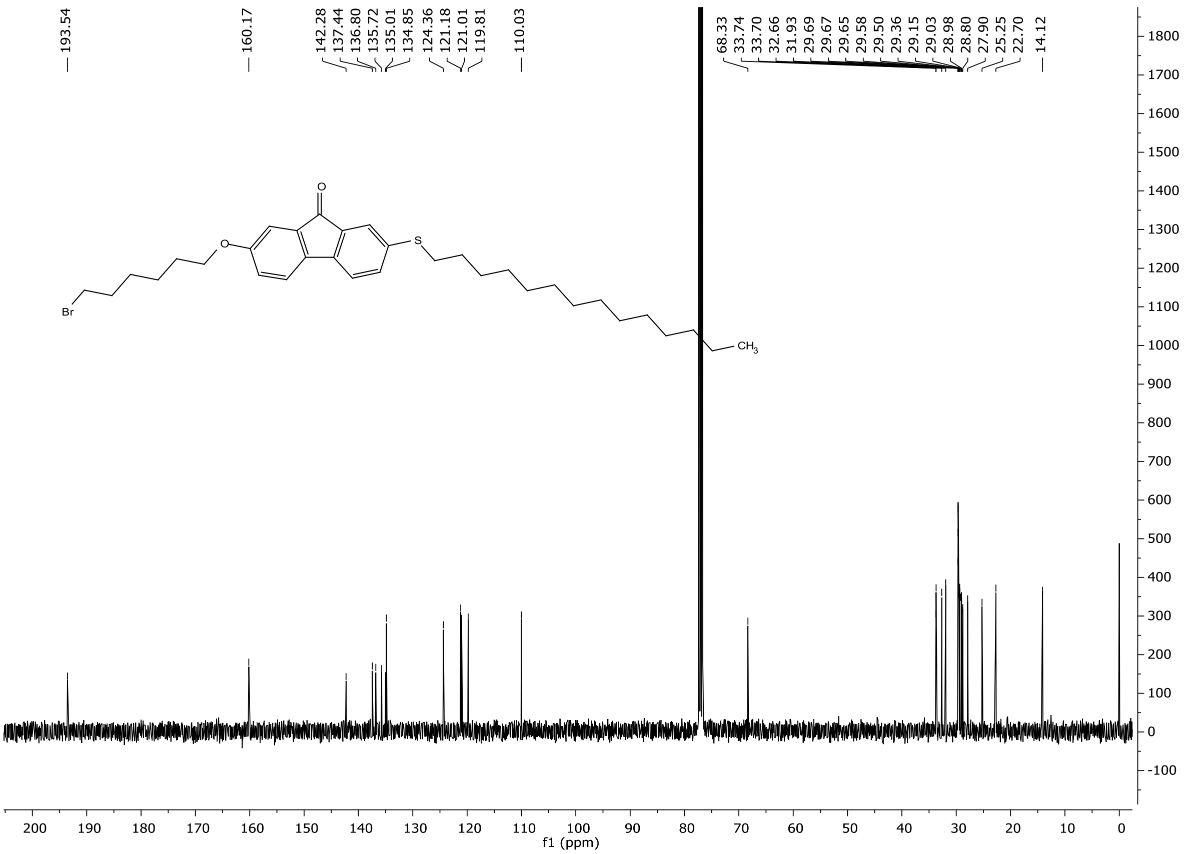

193.54
160.17
142.28
137.44
136.80
135.72
135.01
134.85
124.36
121.18
121.01
119.81
110.03
68.33
33.74
33.70
32.66
31.93
29.69
29.67
29.65
29.58
29.50
29.36
29.15
29.03
28.98
28.80
27.90
25.25
22.70
14.12
O
S
Br
CH3
200 190 180 170 160 150 140 130 120 110 100 90 80 70 60 50 40 30 20 10 0
f1 (ppm)
1800 1700 1600 1500 1400 1300 1200 1100 1000 900 800 700 600 500 400 300 200 100 0 -100

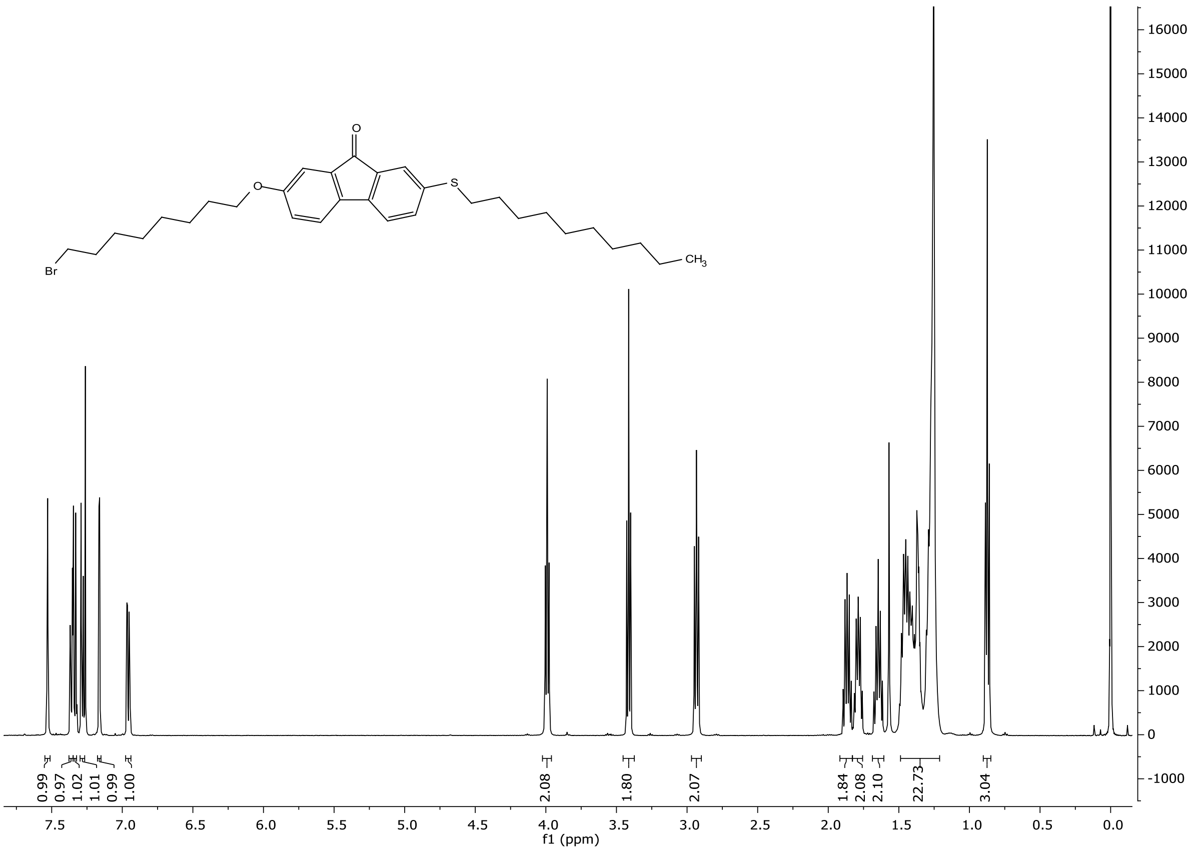
O
S
Br
CH3
0.99
0.97
1.02
1.01
0.99
1.00
2.08
1.80
2.07
1.84
2.08
2.10
22.73
3.04
f1 (ppm)

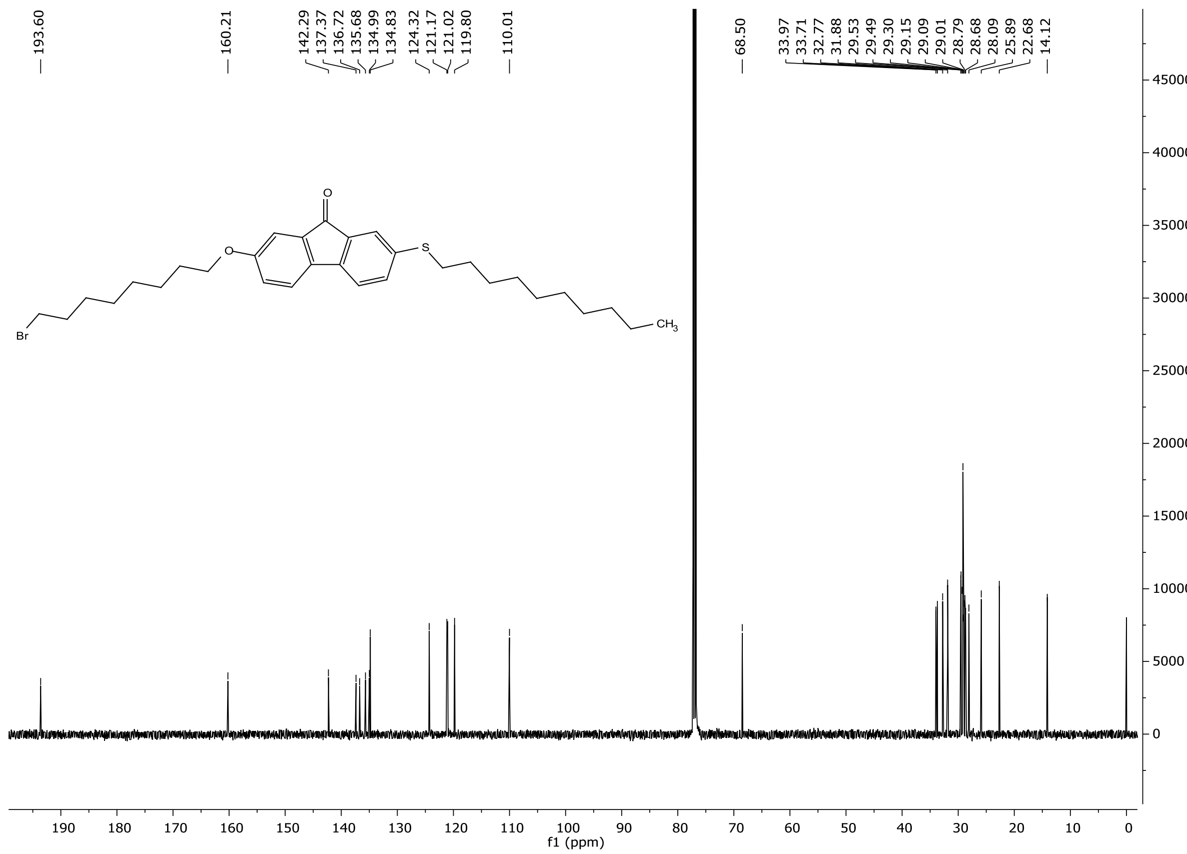
193.60
160.21
142.29
137.37
136.72
135.68
134.99
134.83
124.32
121.17
121.02
119.80
110.01
68.50
33.97
33.71
32.77
31.88
29.53
29.49
29.30
29.15
29.09
29.01
28.79
28.68
28.09
25.89
22.68
14.12
O
S
Br
CH3
f1 (ppm)

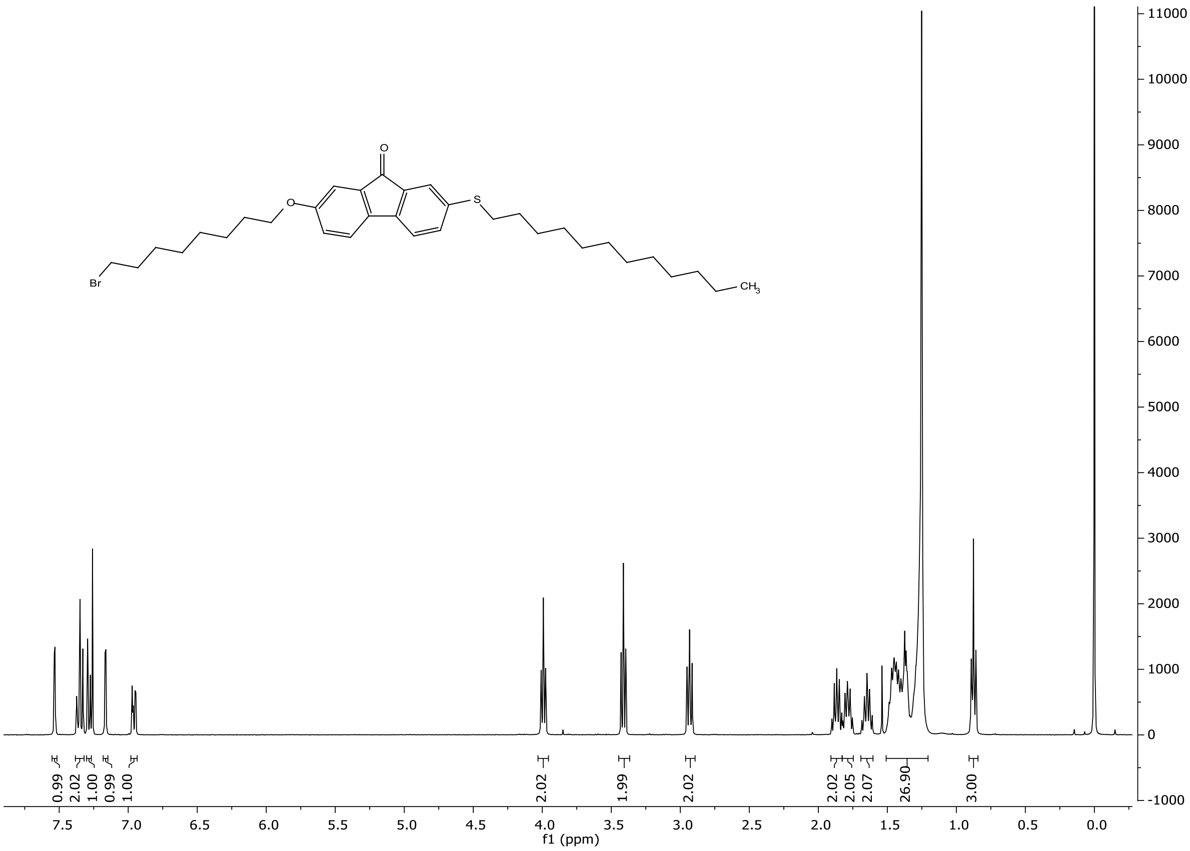

Br
CH3
7.5
7.0
6.5
6.0
5.5
5.0
4.5
4.0
3.5
3.0
2.5
2.0
1.5
1.0
0.5
0.0
f1 (ppm)
0.99
2.02
1.00
0.99
1.00
2.02
1.99
2.02
2.02
2.05
2.07
26.90
3.00

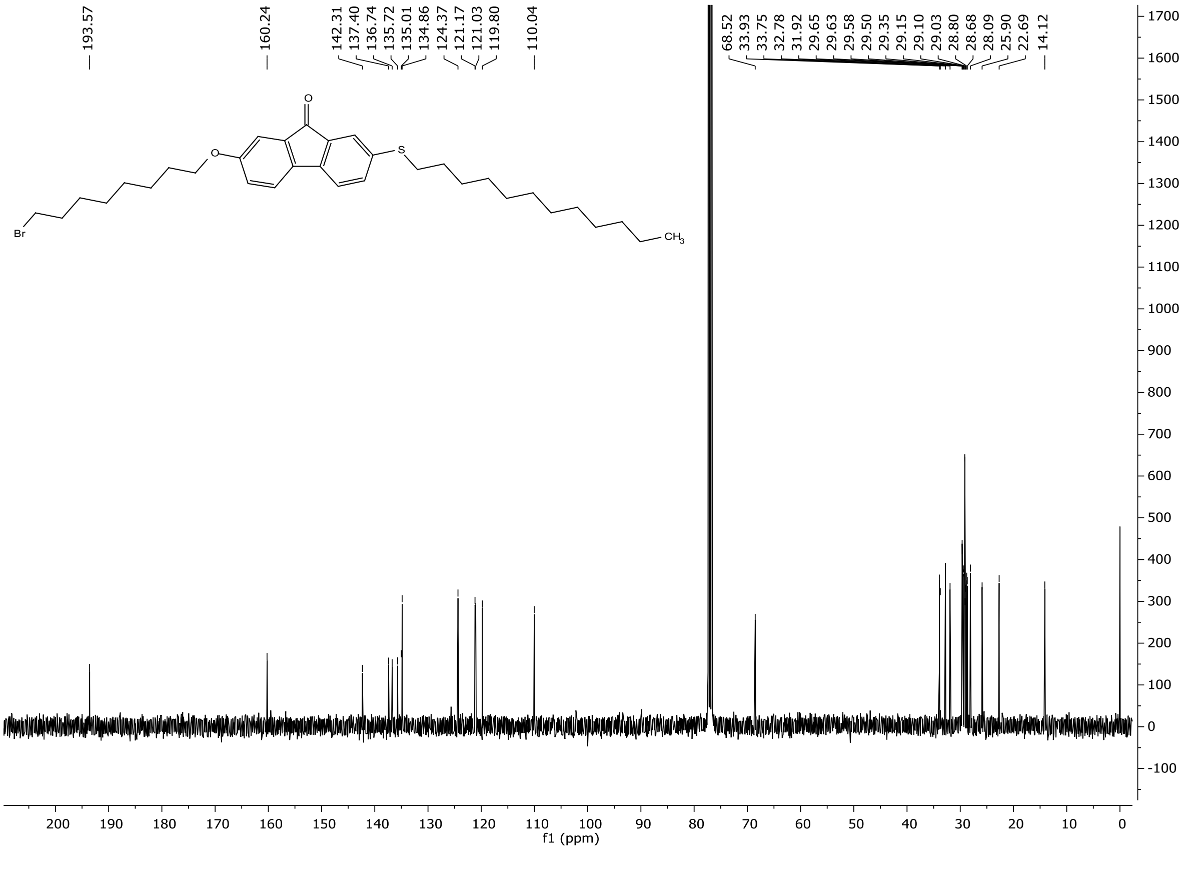

193.57
160.24
142.31
137.40
136.74
135.72
135.01
134.86
124.37
121.17
121.03
119.80
110.04
68.52
33.93
33.75
32.78
31.92
29.65
29.63
29.58
29.50
29.35
29.15
29.10
29.03
28.80
28.68
28.09
25.90
22.69
14.12
Br
CH3
f1 (ppm)

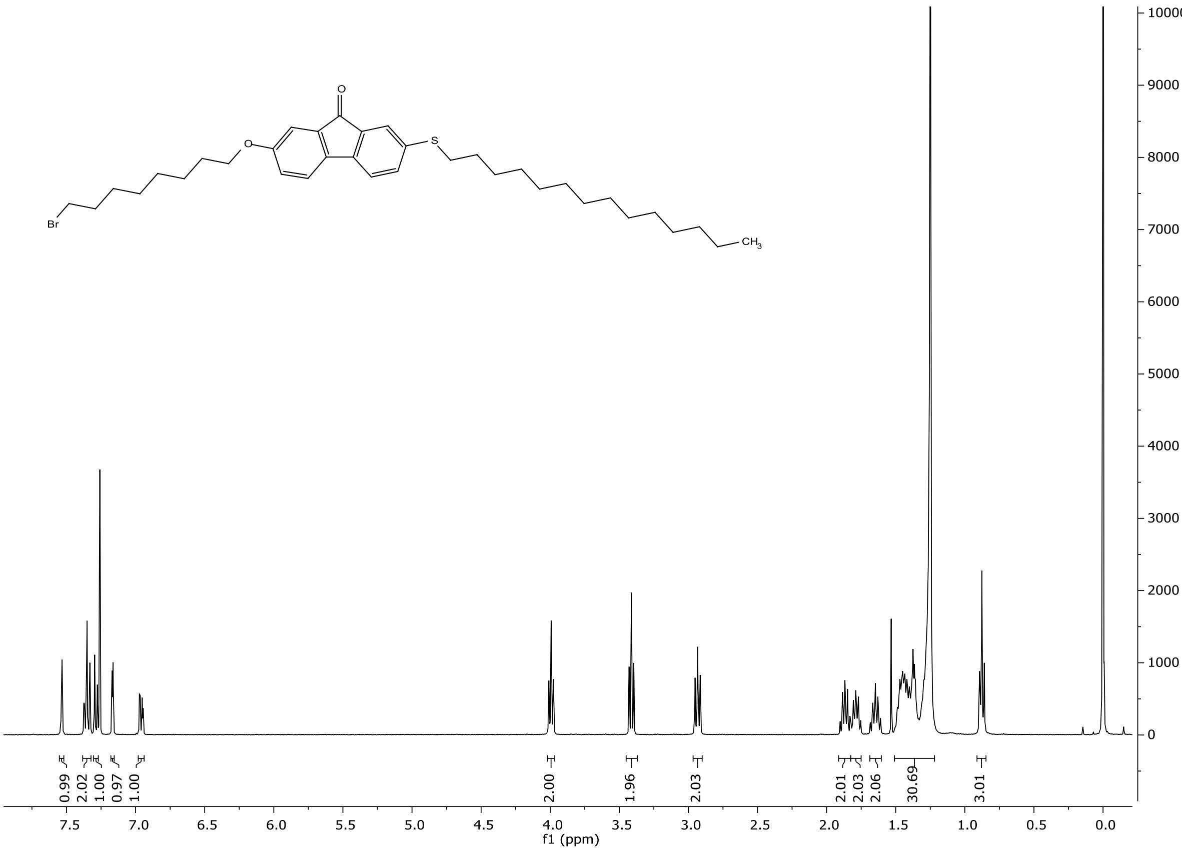

O
S
Br
CH3
0.99
2.02
1.00
0.97
1.00
2.00
1.96
2.03
2.01
2.03
2.06
30.69
3.01
f1 (ppm)

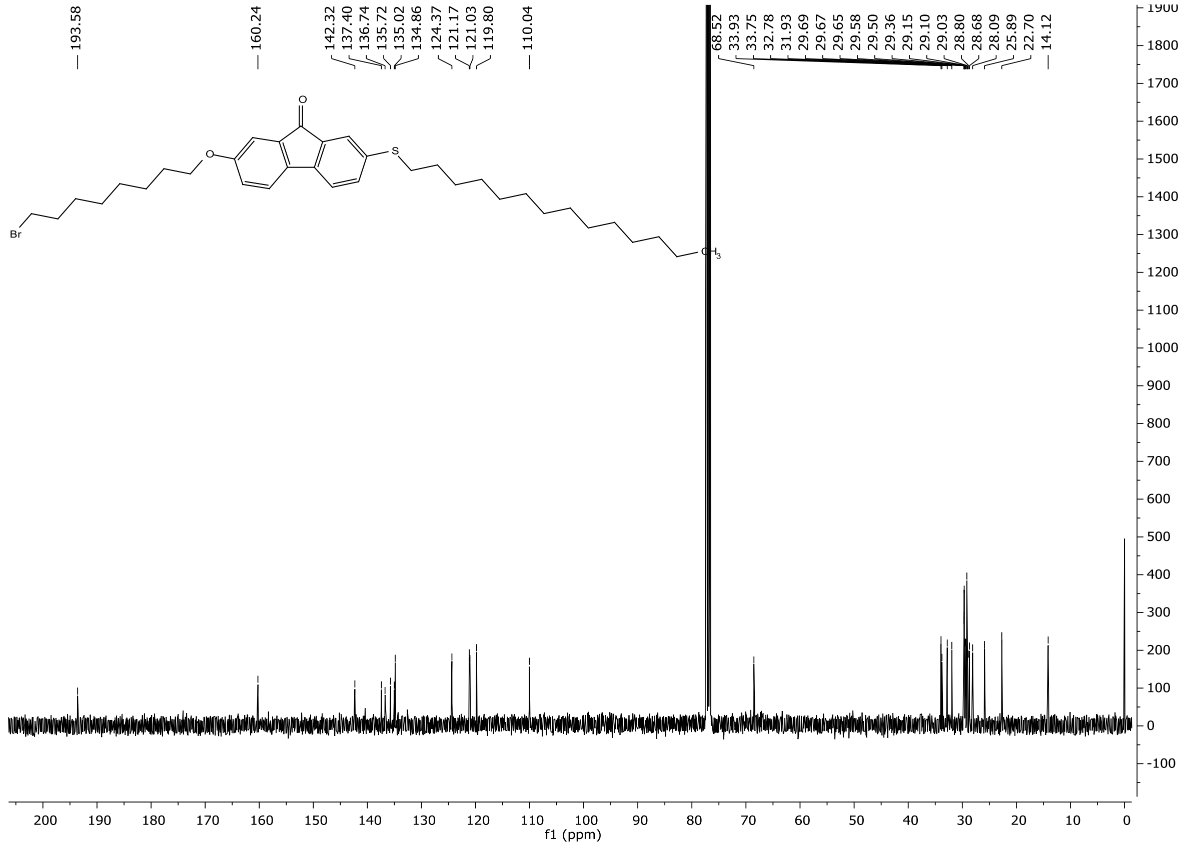

193.58
160.24
142.32
137.40
136.74
135.72
135.02
134.86
124.37
121.17
121.03
119.80
110.04
68.52
33.93
33.75
32.78
31.93
29.69
29.67
29.65
29.58
29.50
29.36
29.15
29.10
29.03
28.80
28.68
28.09
25.89
22.70
14.12
O
S
Br
CH3
f1 (ppm)

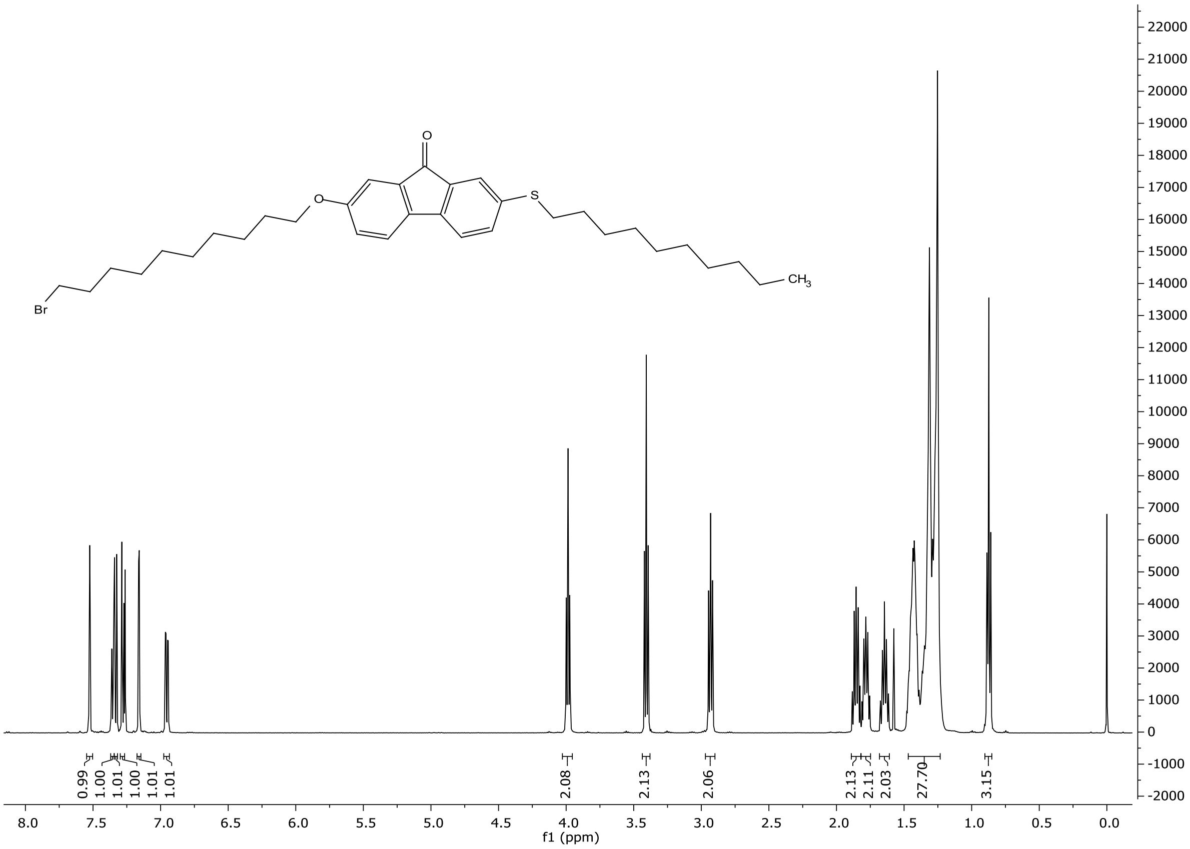

O
O
S
Br
CH3
0.99
1.00
1.01
1.00
1.01
1.01
2.08
2.13
2.06
2.13
2.11
2.03
27.70
3.15
f1 (ppm)

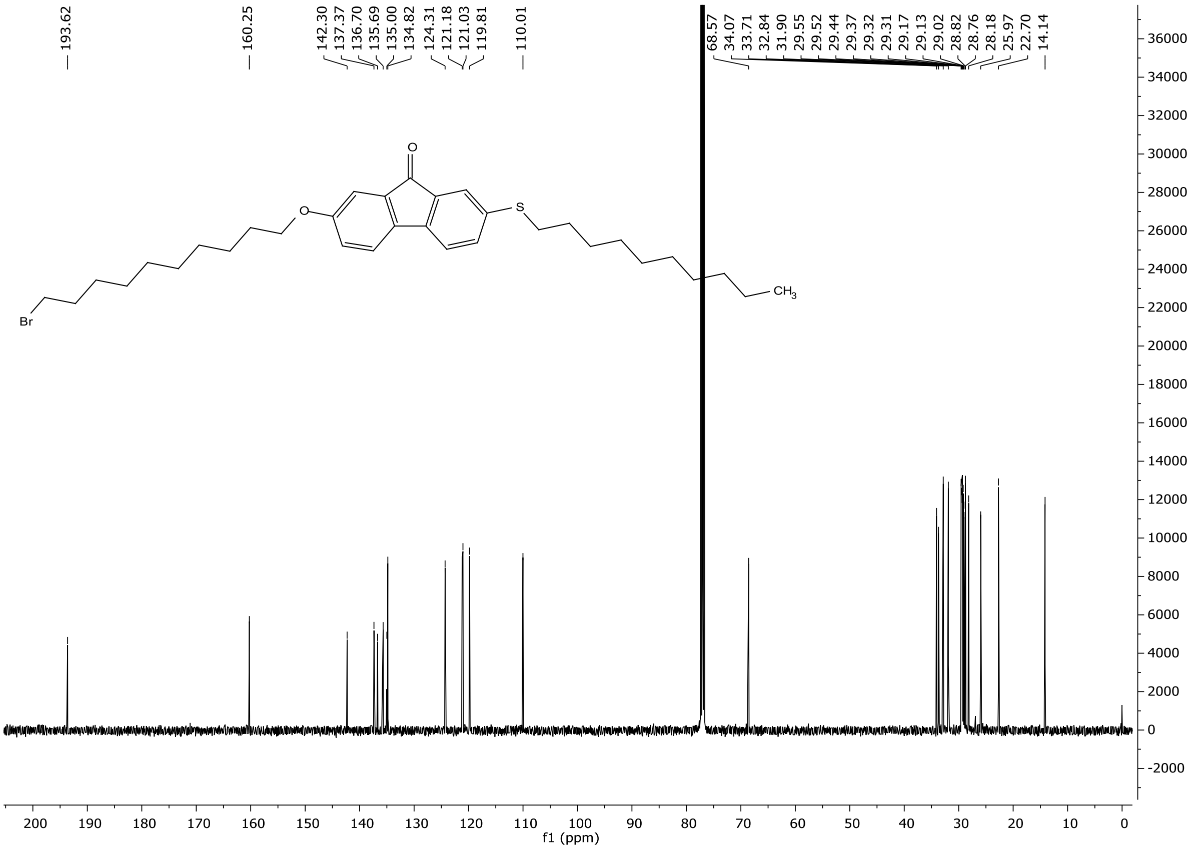

193.62
160.25
142.30
137.37
136.70
135.69
135.00
134.82
124.31
121.18
121.03
119.81
110.01
68.57
34.07
33.71
32.84
31.90
29.55
29.52
29.44
29.37
29.32
29.31
29.17
29.13
29.02
28.82
28.76
28.18
25.97
22.70
14.14
O
O
S
Br
CH3
f1 (ppm)

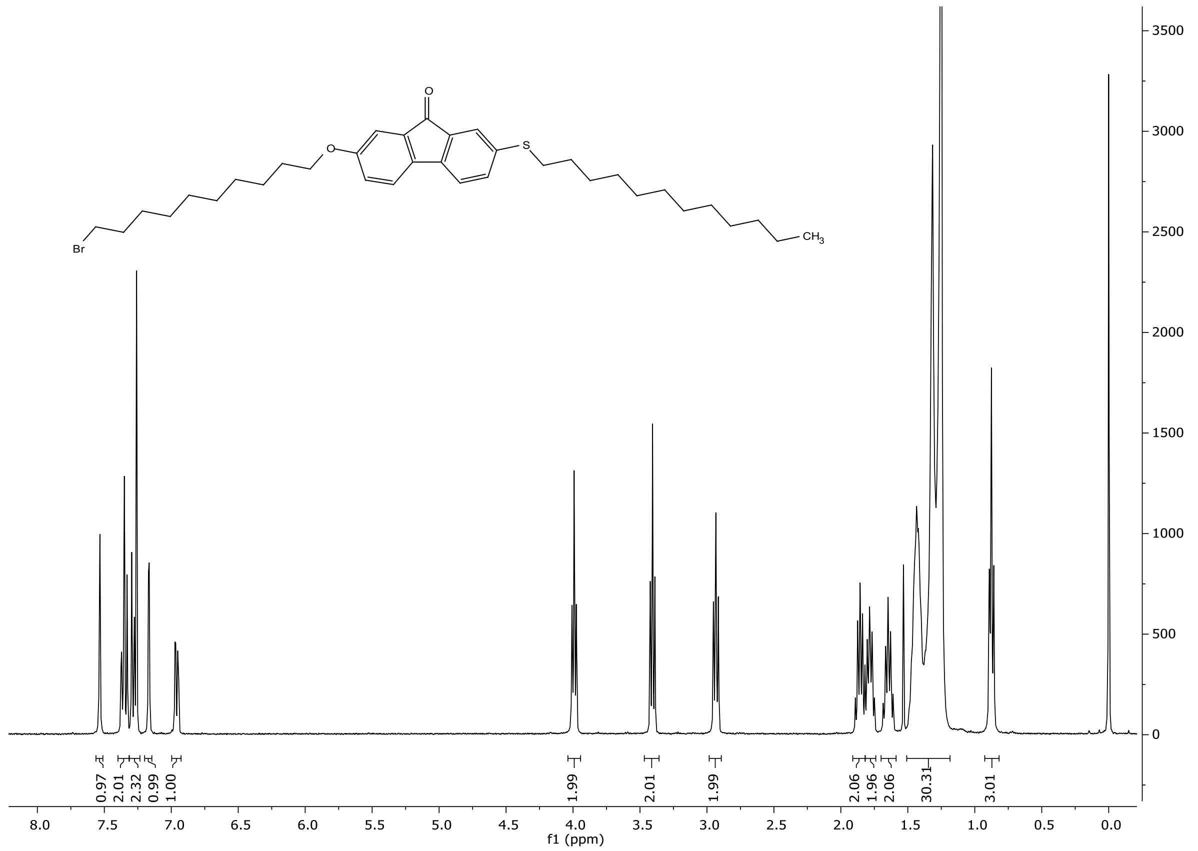

O
S
Br
CH3
0.97
2.01
2.32
0.99
1.00
1.99
2.01
1.99
2.06
1.96
2.06
30.31
3.01
8.0 7.5 7.0 6.5 6.0 5.5 5.0 4.5 4.0 3.5 3.0 2.5 2.0 1.5 1.0 0.5 0.0
f1 (ppm)
3500 3000 2500 2000 1500 1000 500 0

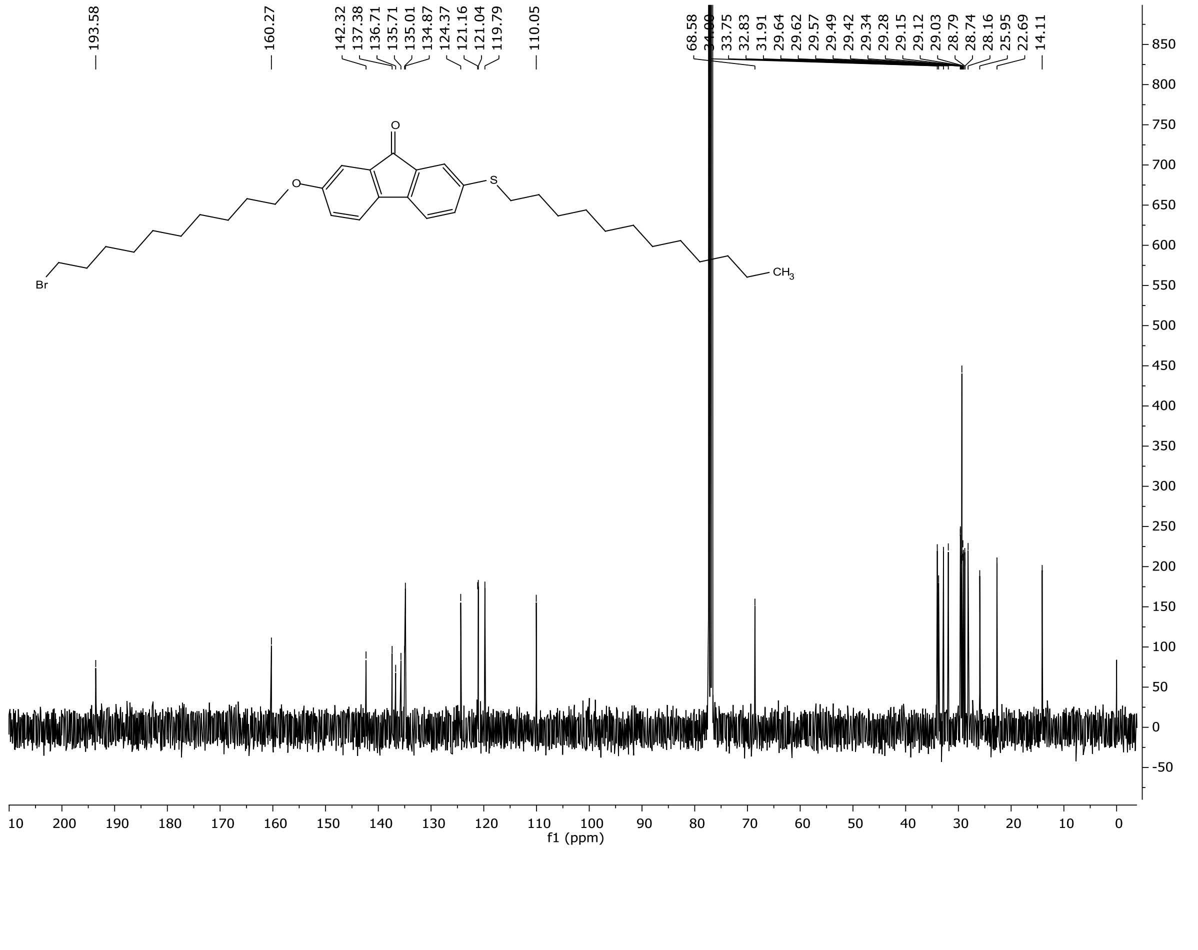

193.58
160.27
142.32
137.38
136.71
135.71
135.01
134.87
124.37
121.16
121.04
119.79
110.05
68.58
33.75
32.83
31.91
29.64
29.62
29.57
29.49
29.42
29.34
29.28
29.15
29.12
29.03
28.79
28.74
28.16
25.95
22.69
14.11
O
S
Br
CH3
10 200 190 180 170 160 150 140 130 120 110 100 90 80 70 60 50 40 30 20 10 0
f1 (ppm)
850 800 750 700 650 600 550 500 450 400 350 300 250 200 150 100 50 0 -50

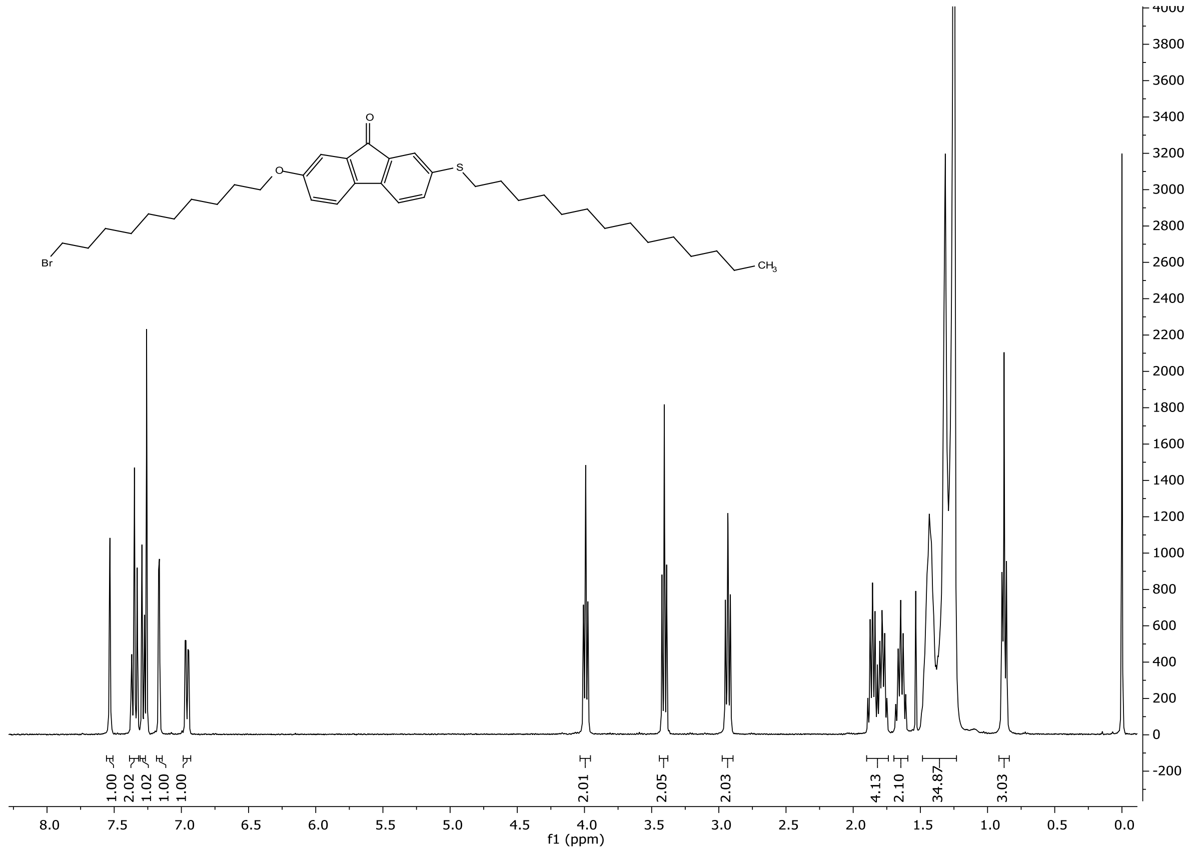
Br
O
O
S
CH3
1.00
2.02
1.02
1.00
1.00
2.01
2.05
2.03
4.13
2.10
34.87
3.03
f1 (ppm)

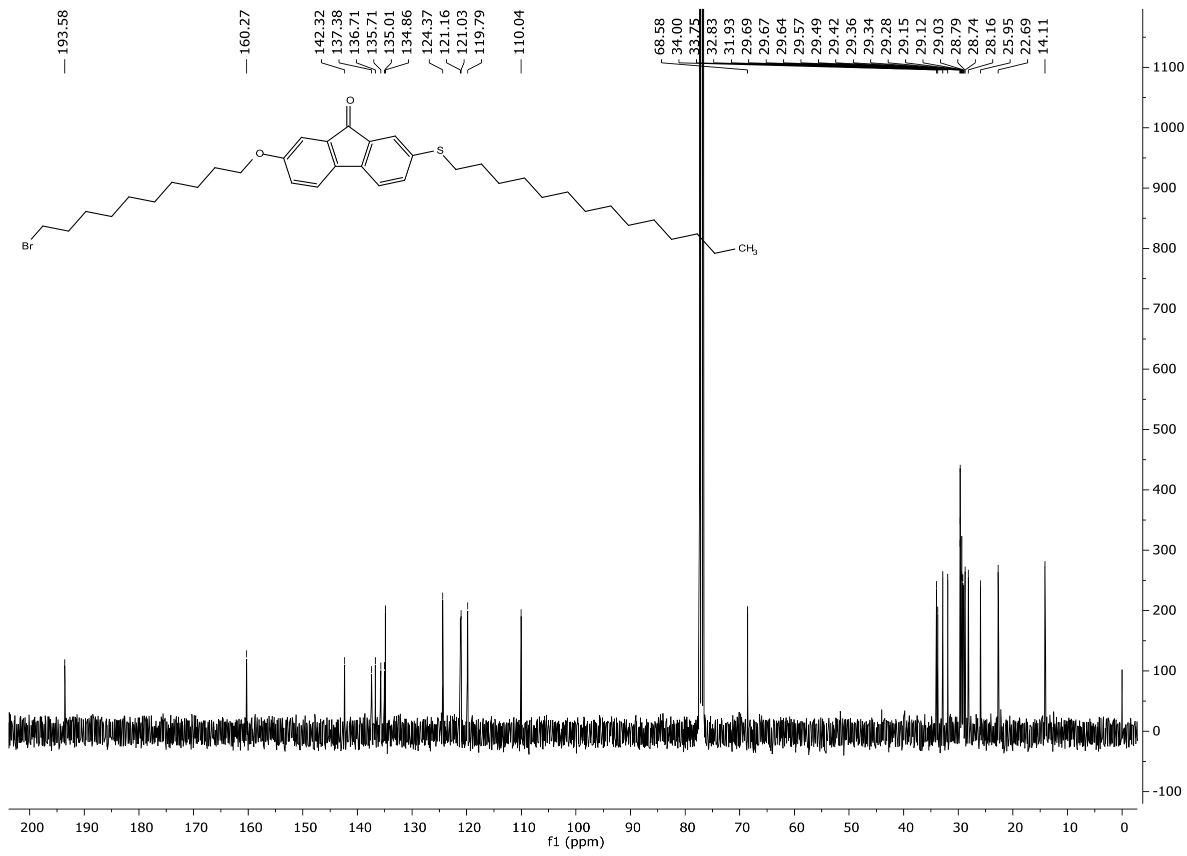
193.58
160.27
142.32
137.38
136.71
135.71
135.01
134.86
124.37
121.16
121.03
119.79
110.04
68.58
34.00
33.75
32.83
31.93
29.69
29.67
29.64
29.57
29.49
29.42
29.36
29.34
29.28
29.15
29.12
29.03
28.79
28.74
28.16
25.95
22.69
14.11
Br
O
O
S
CH3
f1 (ppm)

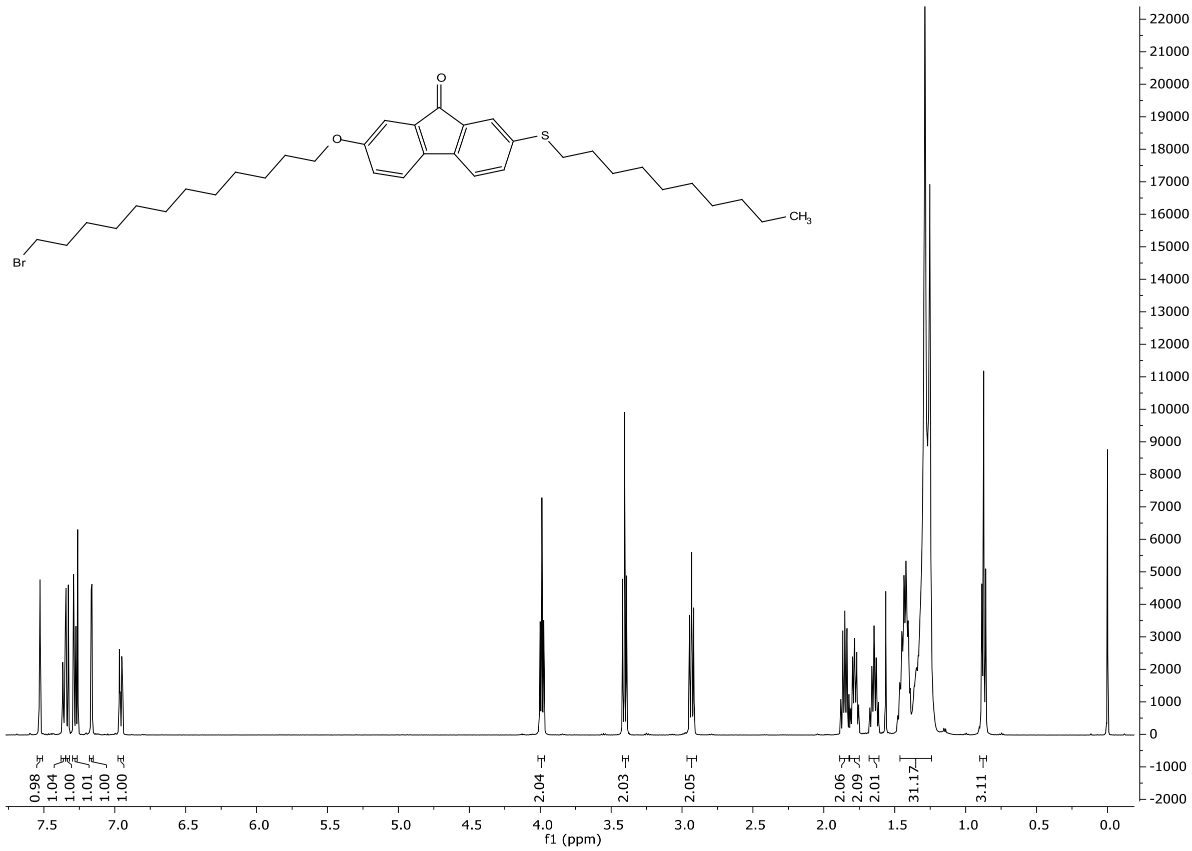

O
S
O
Br
CH3
0.98
1.04
1.00
1.01
1.00
1.00
2.04
2.03
2.05
2.06
2.09
2.01
31.17
3.11
f1 (ppm)

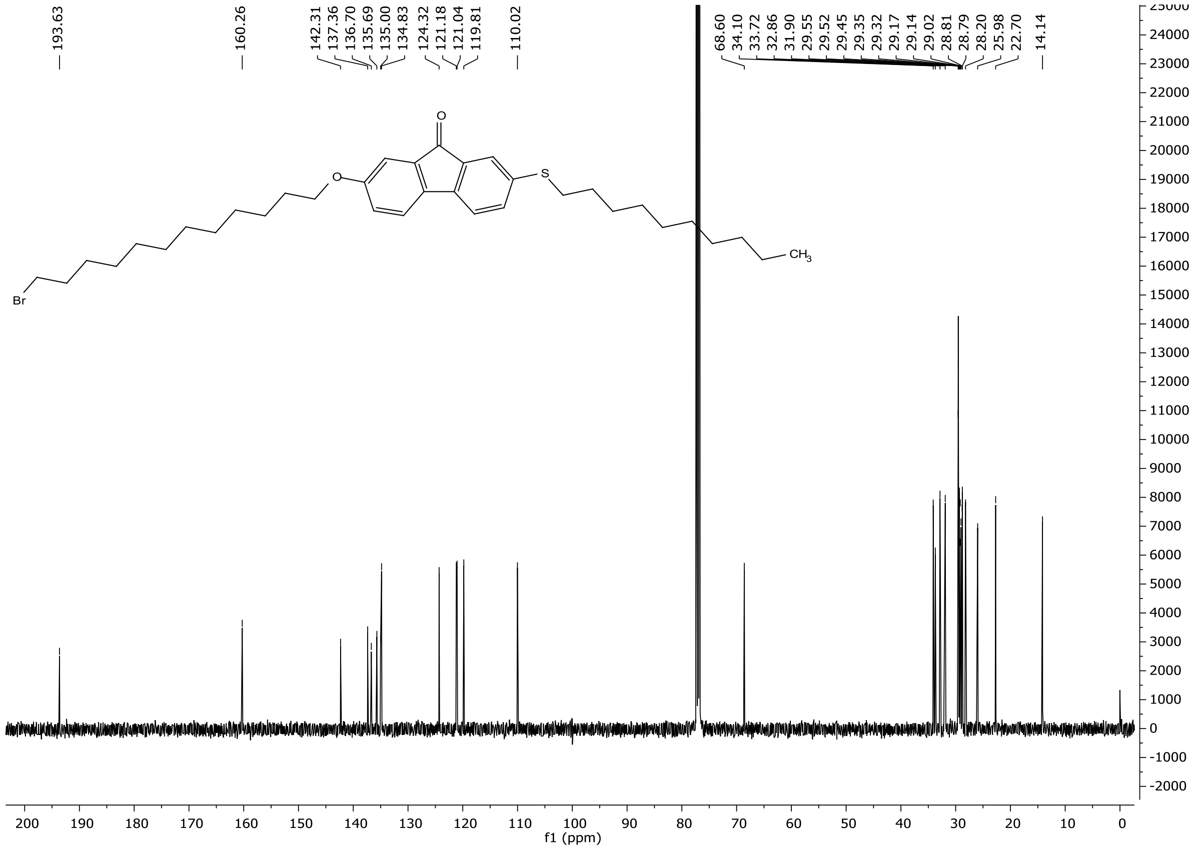

193.63
160.26
142.31
137.36
136.70
135.69
135.00
134.83
124.32
121.18
121.04
119.81
110.02
68.60
34.10
33.72
32.86
31.90
29.55
29.52
29.45
29.35
29.32
29.17
29.14
29.02
28.81
28.79
28.20
25.98
22.70
14.14
O
S
O
Br
CH3
f1 (ppm)

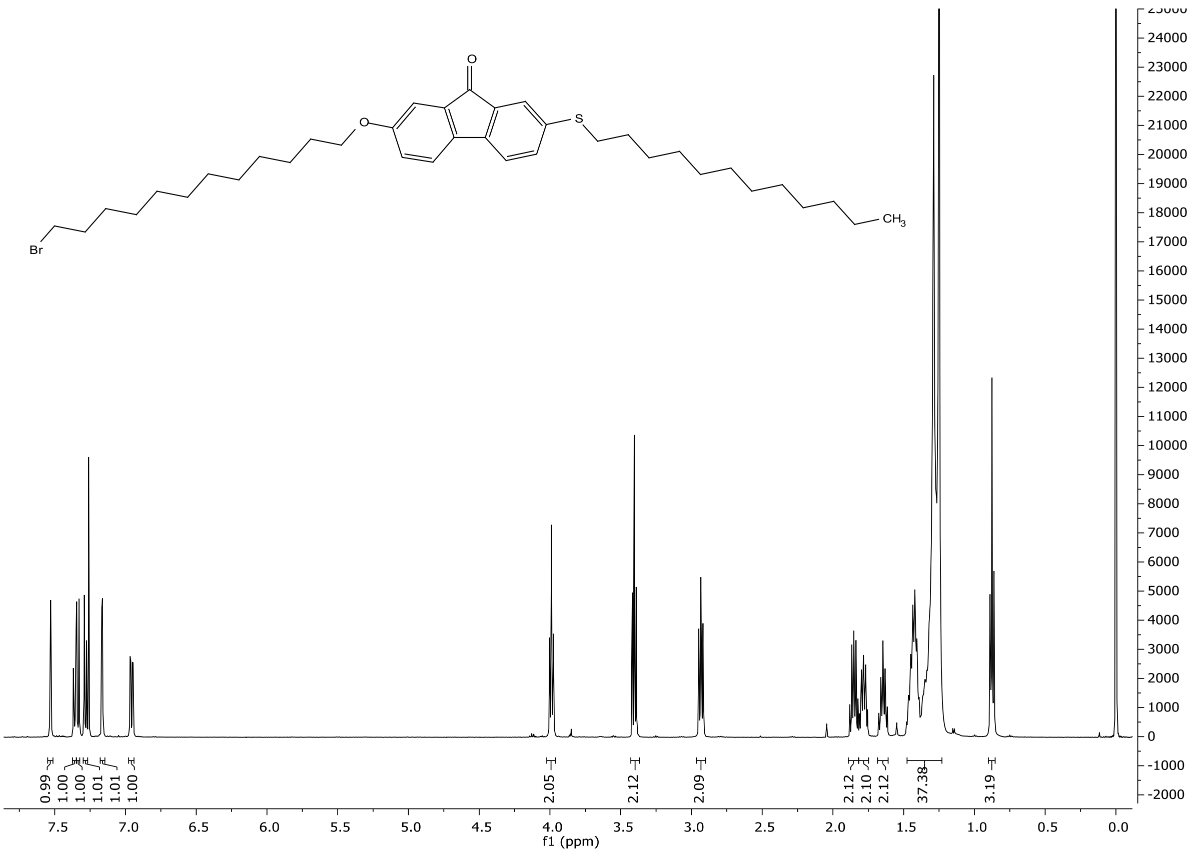
O
O
S
Br
CH3
0.99
1.00
1.00
1.01
1.01
1.00
2.05
2.12
2.09
2.12
2.10
2.12
37.38
3.19
f1 (ppm)

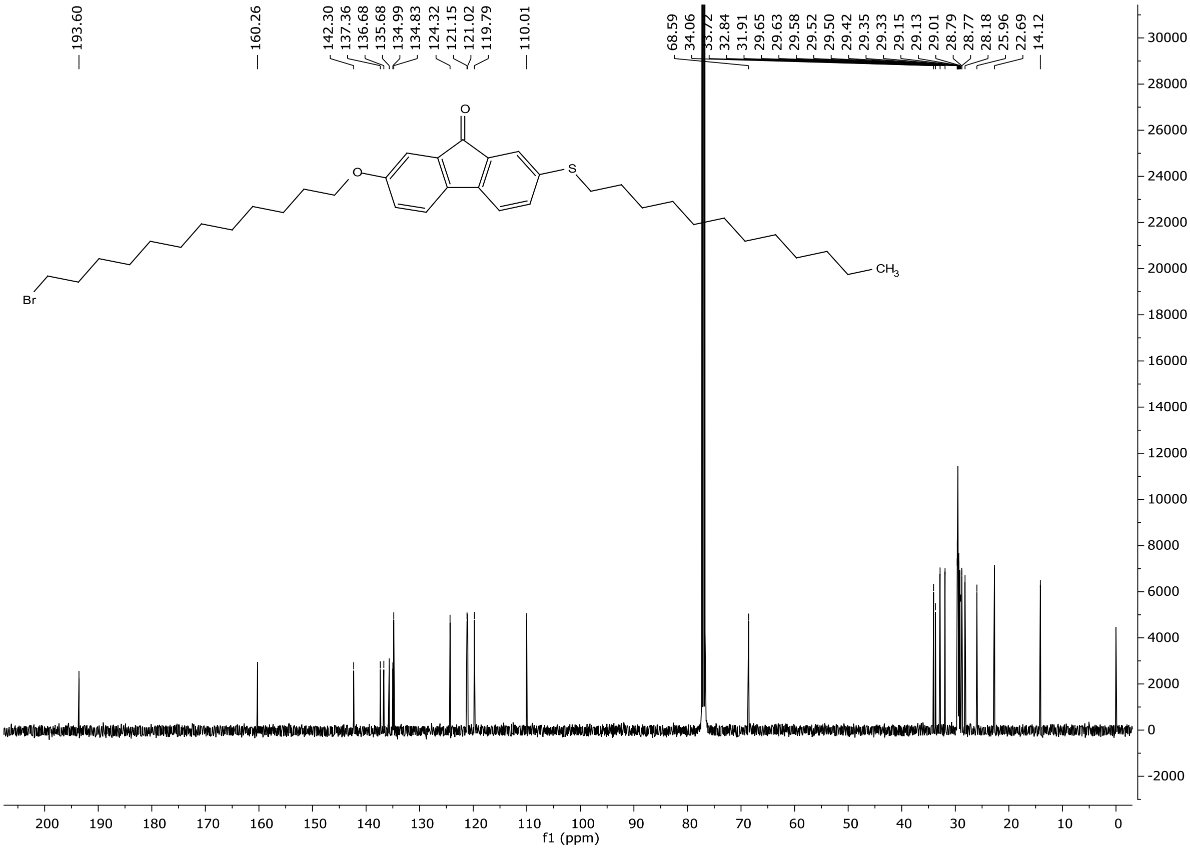
193.60
160.26
142.30
137.36
136.68
135.68
134.99
134.83
124.32
121.15
121.02
119.79
110.01
68.59
34.06
33.72
32.84
31.91
29.65
29.63
29.58
29.52
29.50
29.42
29.35
29.33
29.15
29.13
29.01
28.79
28.77
28.18
25.96
22.69
14.12
O
O
S
Br
CH3
f1 (ppm)

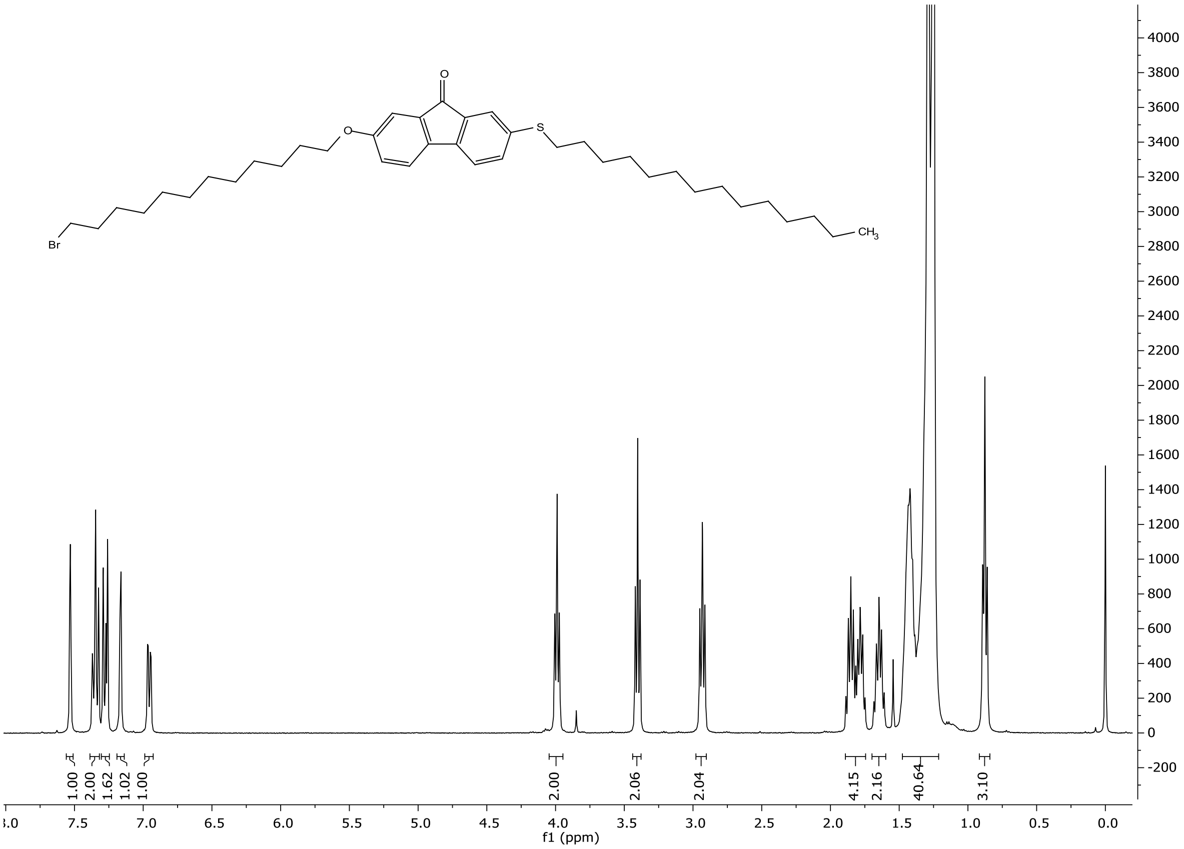

O
O
S
Br
CH3
1.00
2.00
1.62
1.02
1.00
2.00
2.06
2.04
4.15
2.16
40.64
3.10
f1 (ppm)

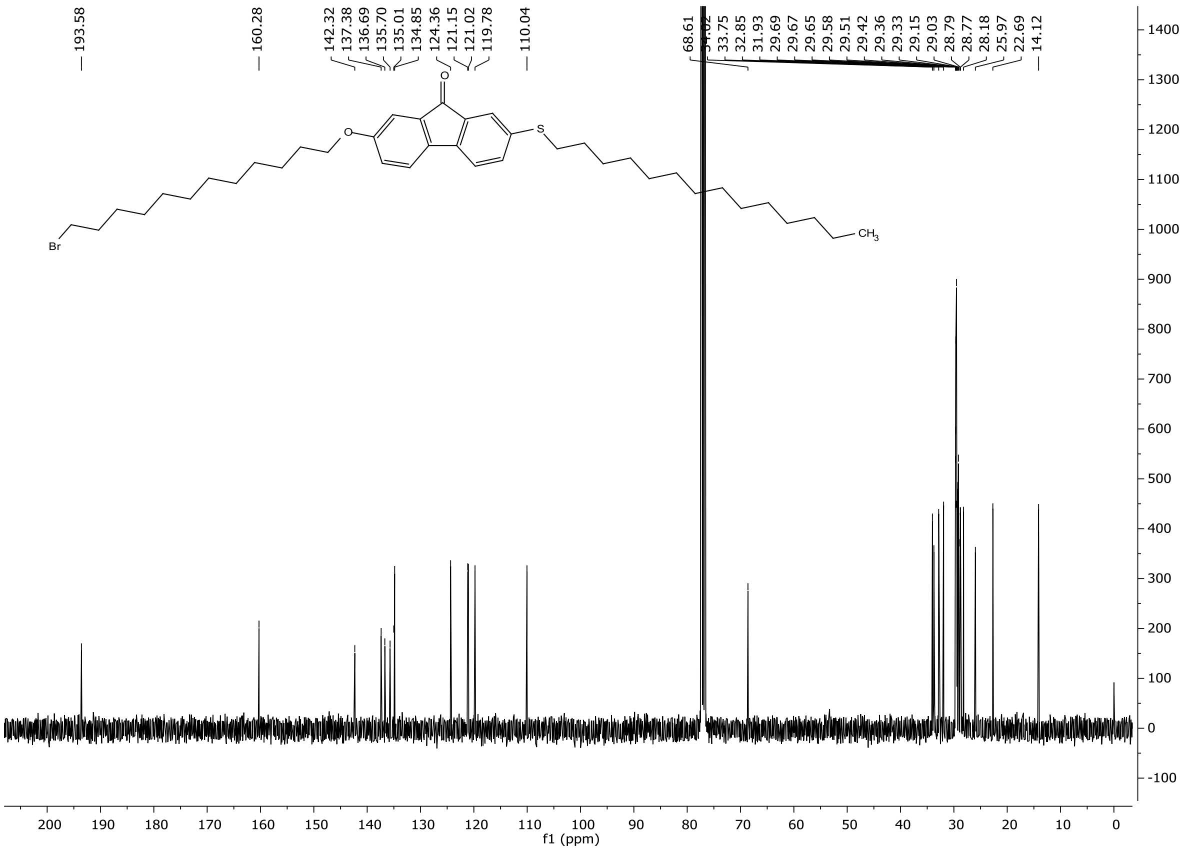

193.58
160.28
142.32
137.38
136.69
135.70
135.01
134.85
124.36
121.15
121.02
119.78
110.04
68.61
33.75
32.85
31.93
29.69
29.67
29.65
29.58
29.51
29.42
29.36
29.33
29.15
29.03
28.79
28.77
28.18
25.97
22.69
14.12
O
O
S
Br
CH3
f1 (ppm)

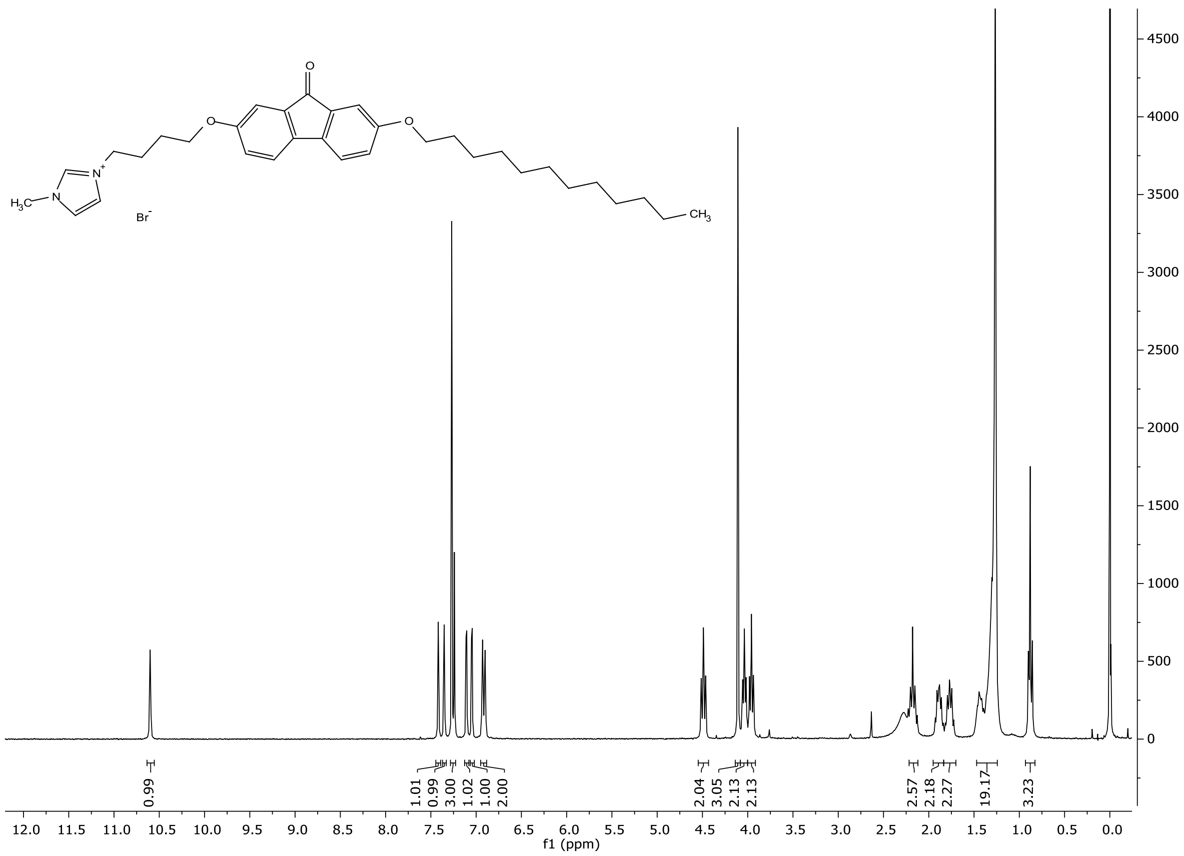

O
O
O
N
N+
H3C
Br-
CH3
0.99
1.01
0.99
3.00
1.02
1.00
2.00
2.04
3.05
2.13
2.13
2.57
2.18
2.27
19.17
3.23
f1 (ppm)

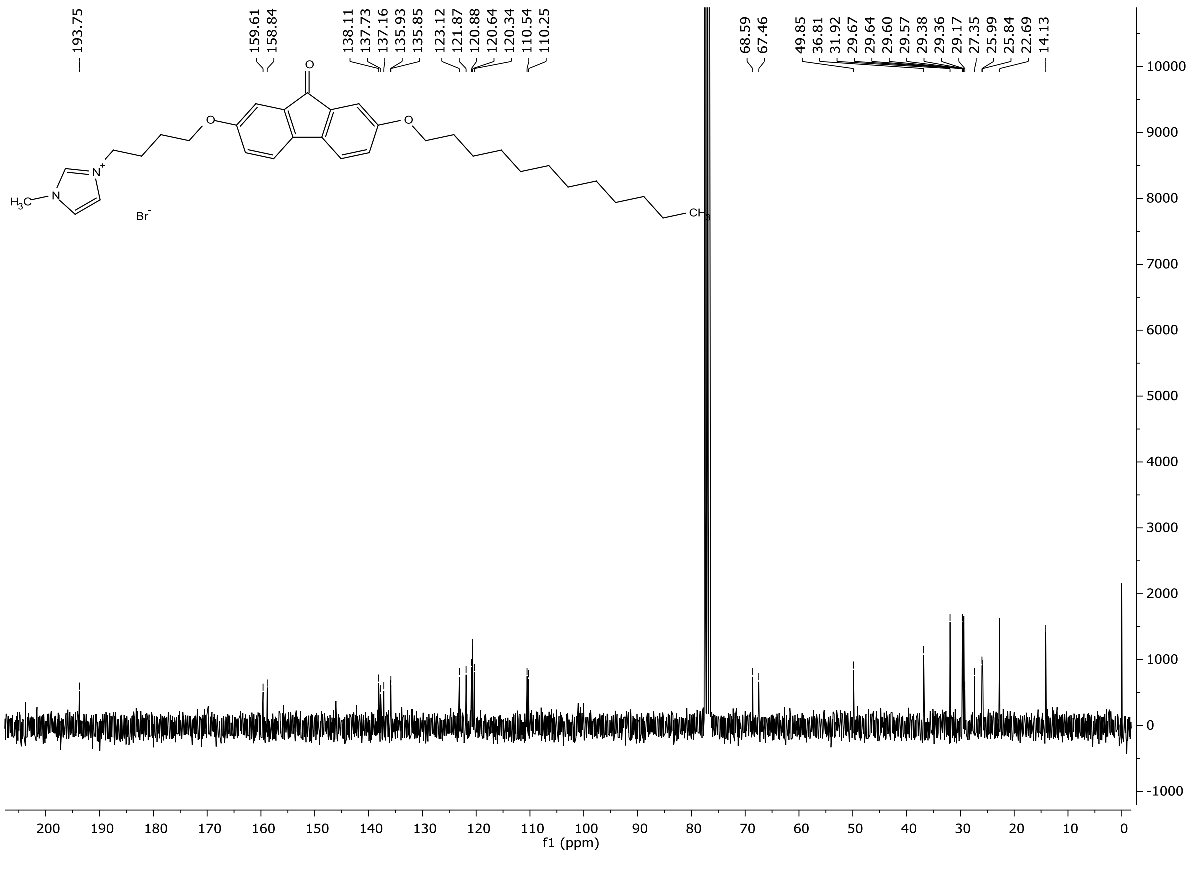

193.75
159.61
158.84
138.11
137.73
137.16
135.93
135.85
123.12
121.87
120.88
120.64
120.34
110.54
110.25
68.59
67.46
49.85
36.81
31.92
29.67
29.64
29.60
29.57
29.38
29.36
29.17
27.35
25.99
25.84
22.69
14.13
Br-
f1 (ppm)

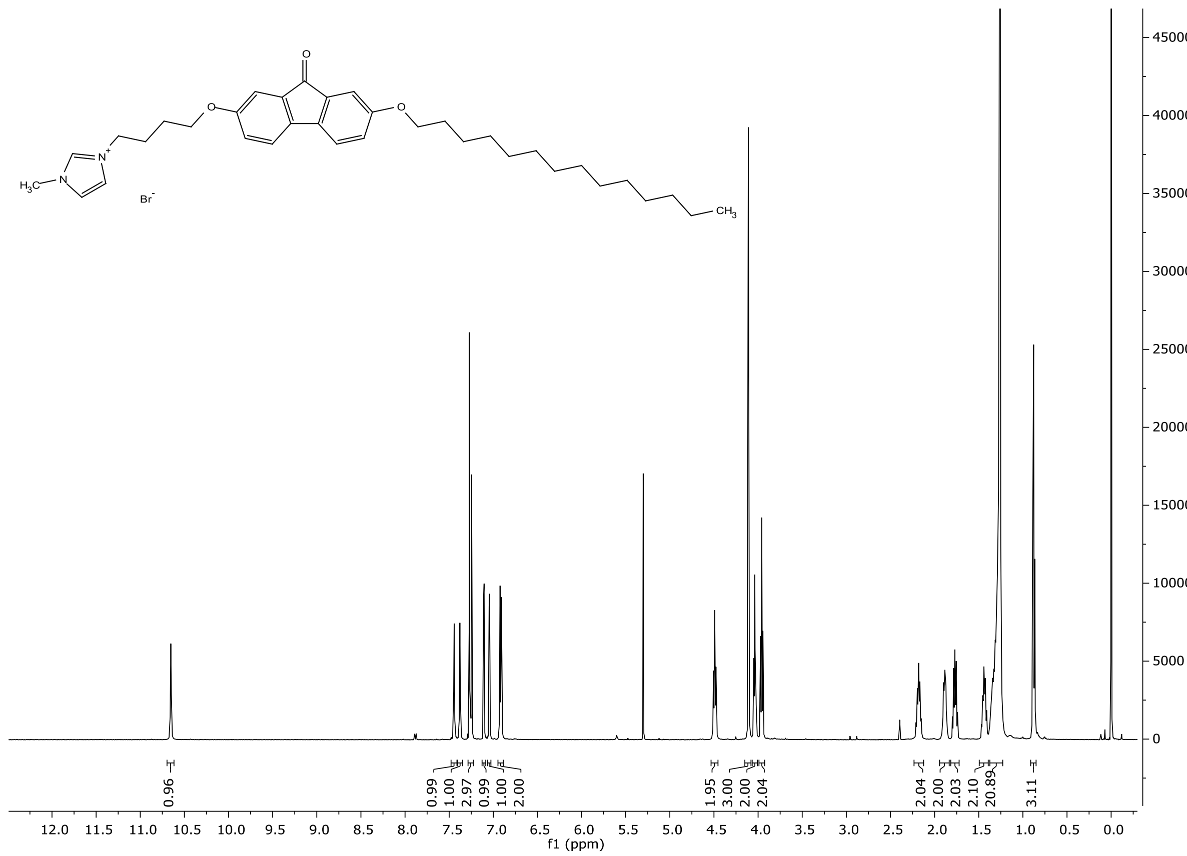
O
O
O
N
N+
H3C
Br-
CH3
0.96
0.99
1.00
2.97
0.99
1.00
2.00
1.95
3.00
2.00
2.04
2.04
2.00
2.03
2.10
20.89
3.11
f1 (ppm)

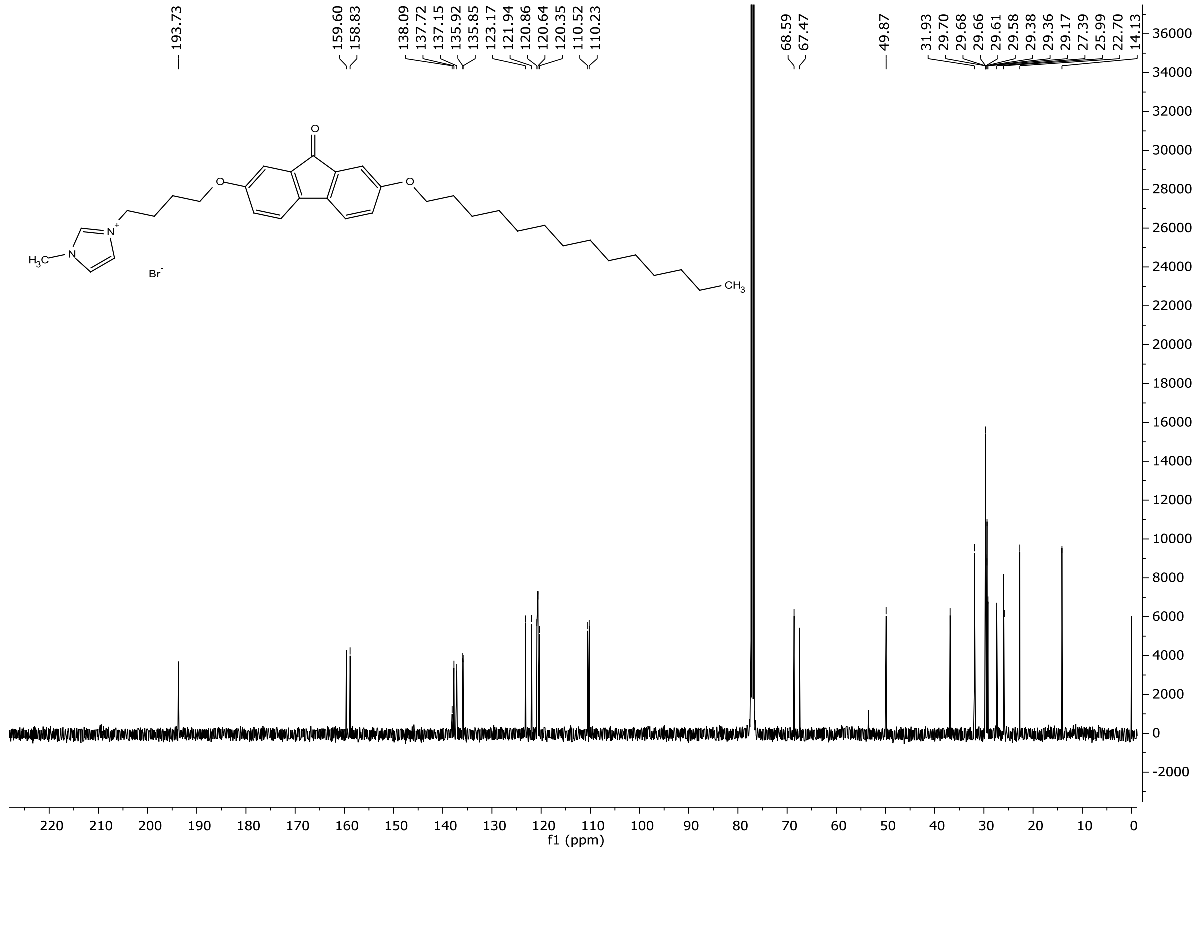
193.73
159.60
158.83
138.09
137.72
137.15
135.92
135.85
123.17
121.94
120.86
120.64
120.35
110.52
110.23
68.59
67.47
49.87
31.93
29.70
29.68
29.66
29.61
29.58
29.38
29.36
29.17
27.39
25.99
22.70
14.13
f1 (ppm)

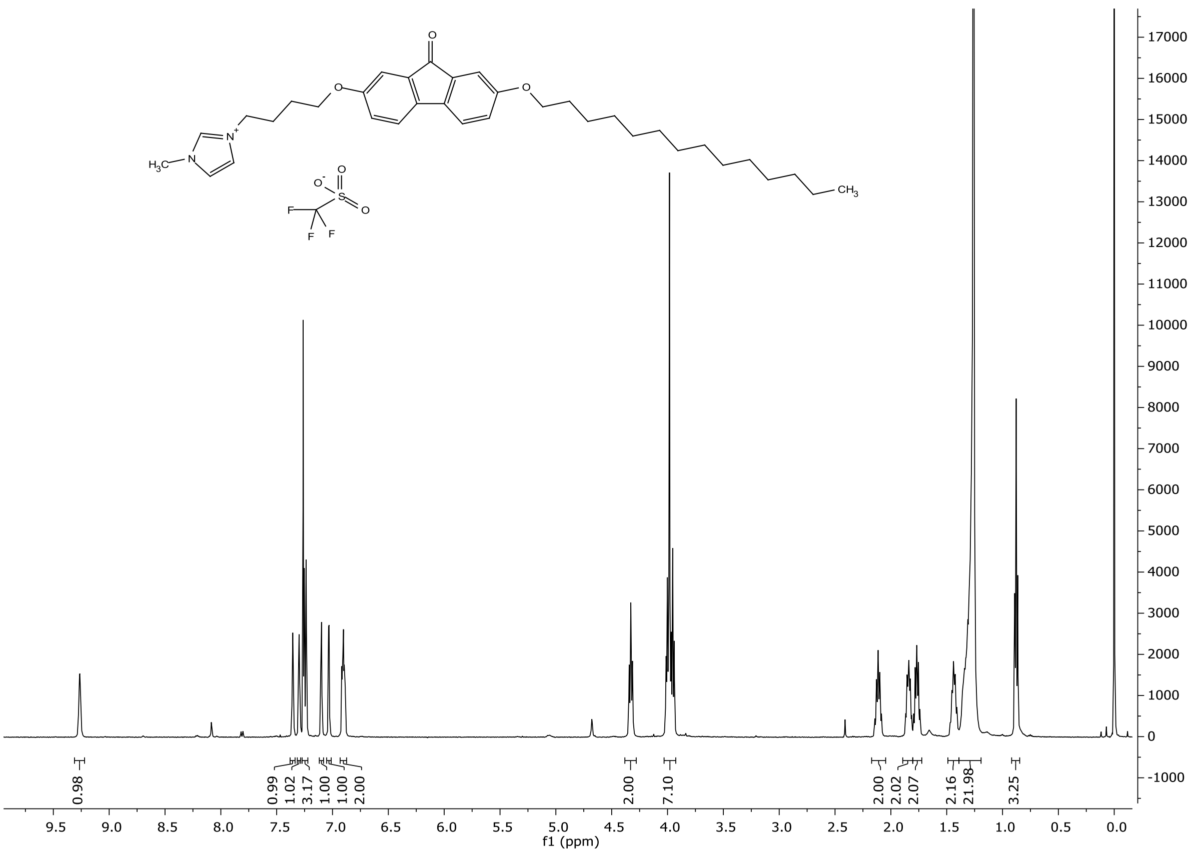
O
O
O
N+
N
H3C
O-
S
O
O
F
F
F
CH3
0.98
0.99
1.02
3.17
1.00
1.00
2.00
2.00
7.10
2.00
2.02
2.07
2.16
21.98
3.25
f1 (ppm)

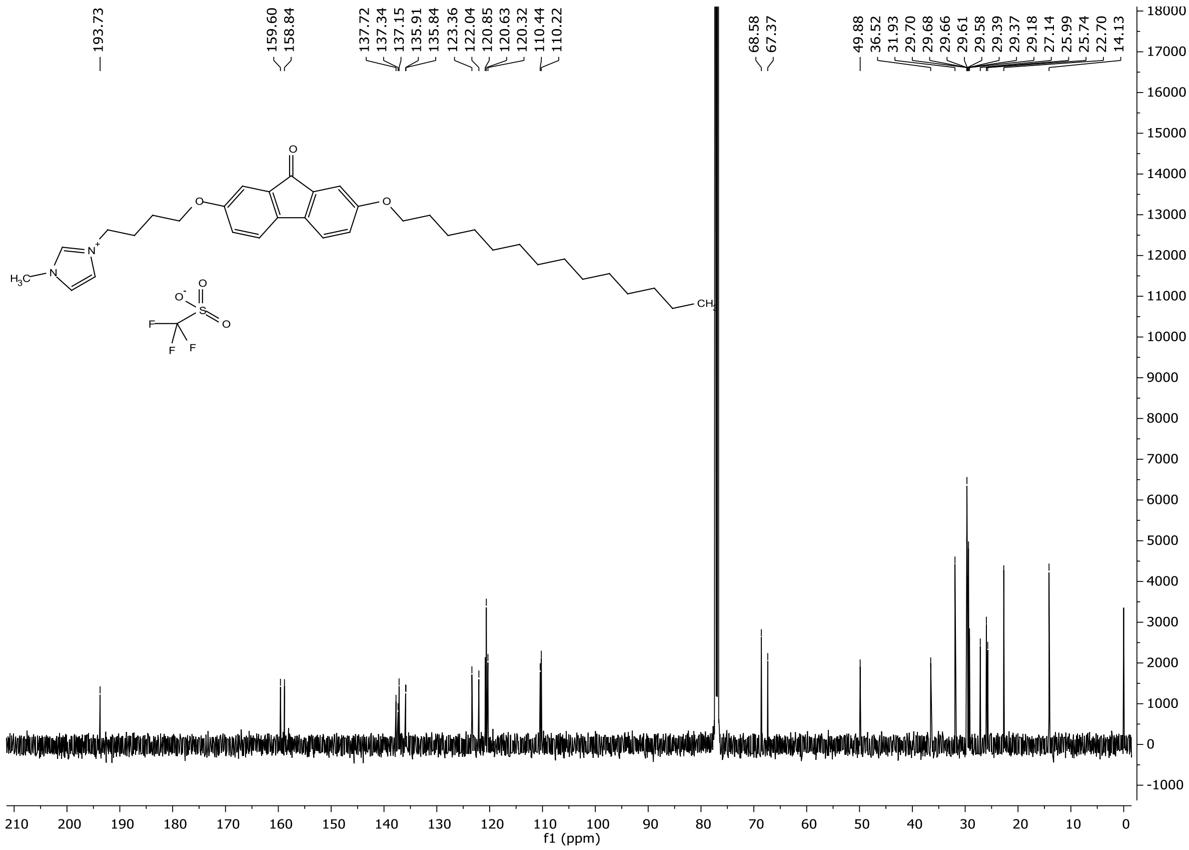
193.73
159.60
158.84
137.72
137.34
137.15
135.91
135.84
123.36
122.04
120.85
120.63
120.32
110.44
110.22
68.58
67.37
49.88
36.52
31.93
29.70
29.68
29.66
29.61
29.58
29.39
29.37
29.18
27.14
25.99
25.74
22.70
14.13
f1 (ppm)

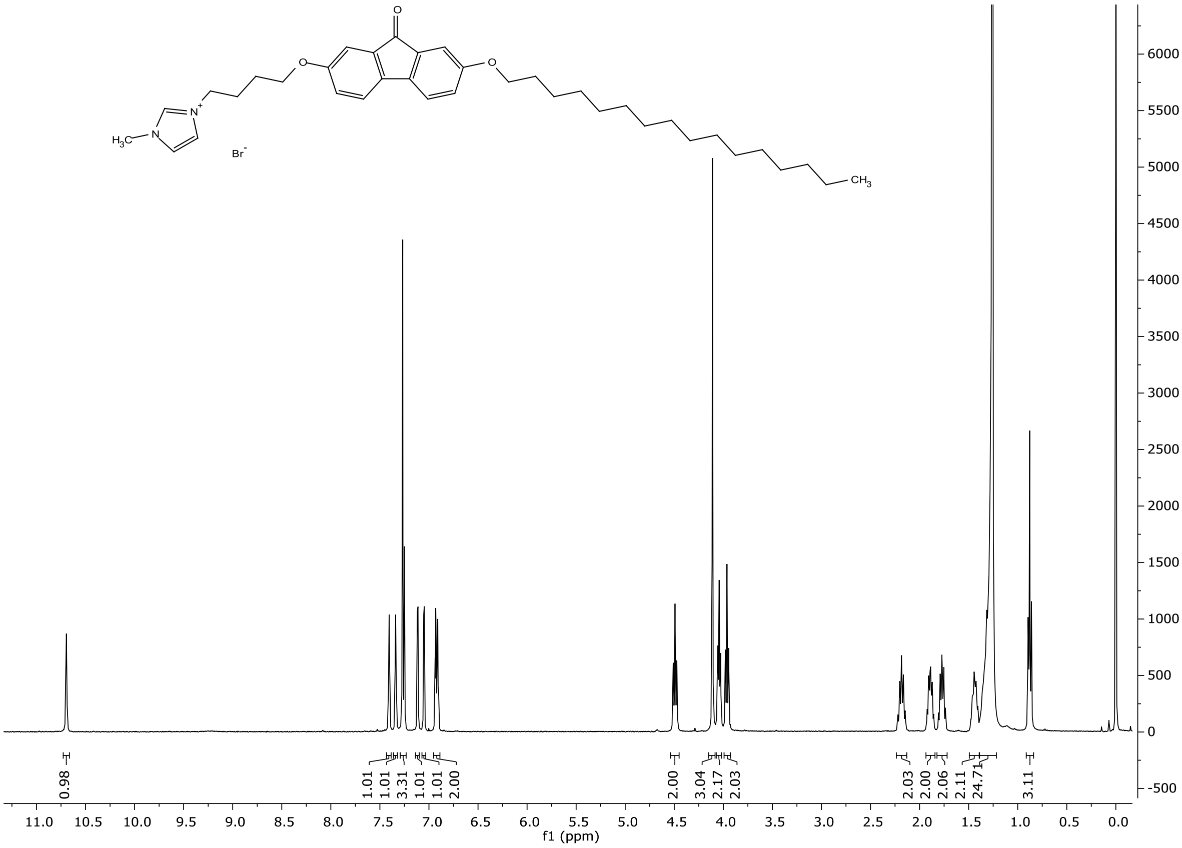

O
O
O
N+
N
H3C
Br-
CH3
0.98
1.01
1.01
3.31
1.01
1.01
2.00
2.00
3.04
2.17
2.03
2.03
2.00
2.06
2.11
24.71
3.11
f1 (ppm)

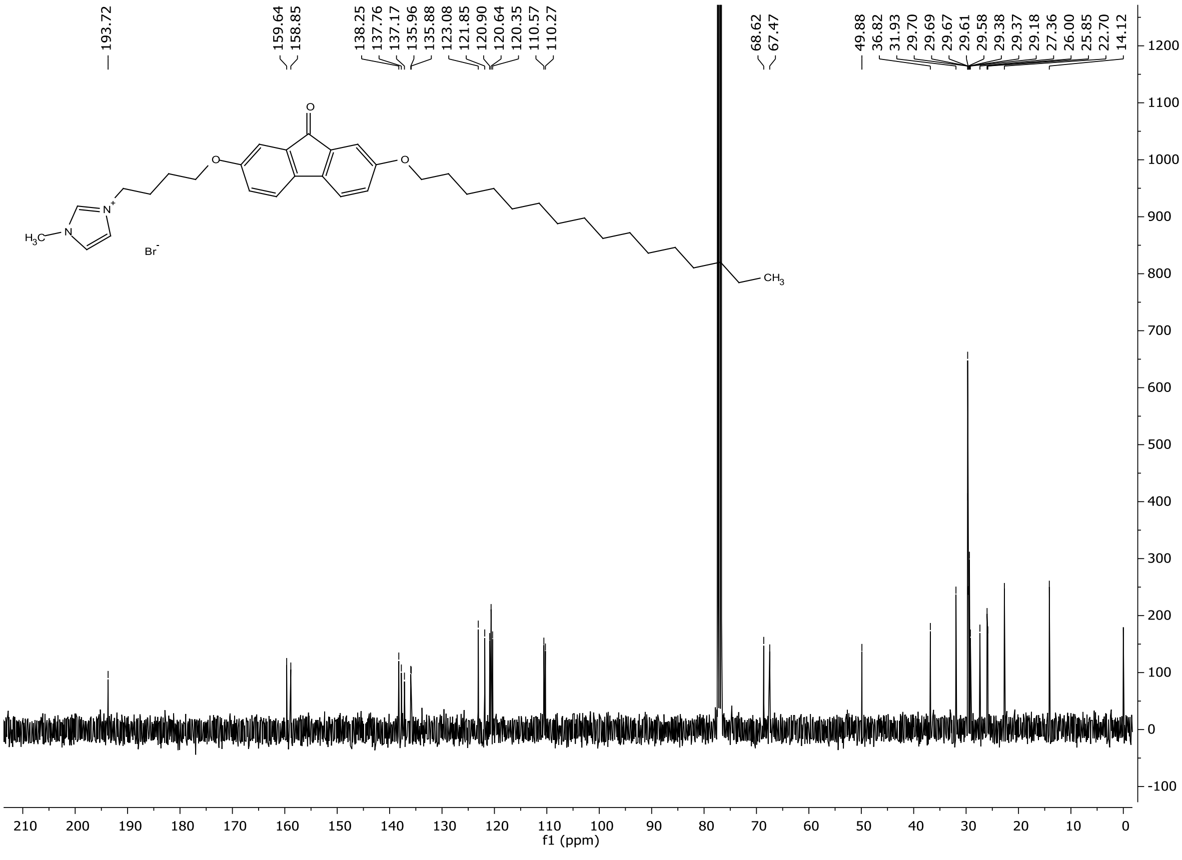

193.72
159.64
158.85
138.25
137.76
137.17
135.96
135.88
123.08
121.85
120.90
120.64
120.35
110.57
110.27
68.62
67.47
49.88
36.82
31.93
29.70
29.69
29.67
29.61
29.58
29.38
29.37
29.18
27.36
26.00
25.85
22.70
14.12
O
O
O
N+
N
H3C
Br-
CH3
f1 (ppm)

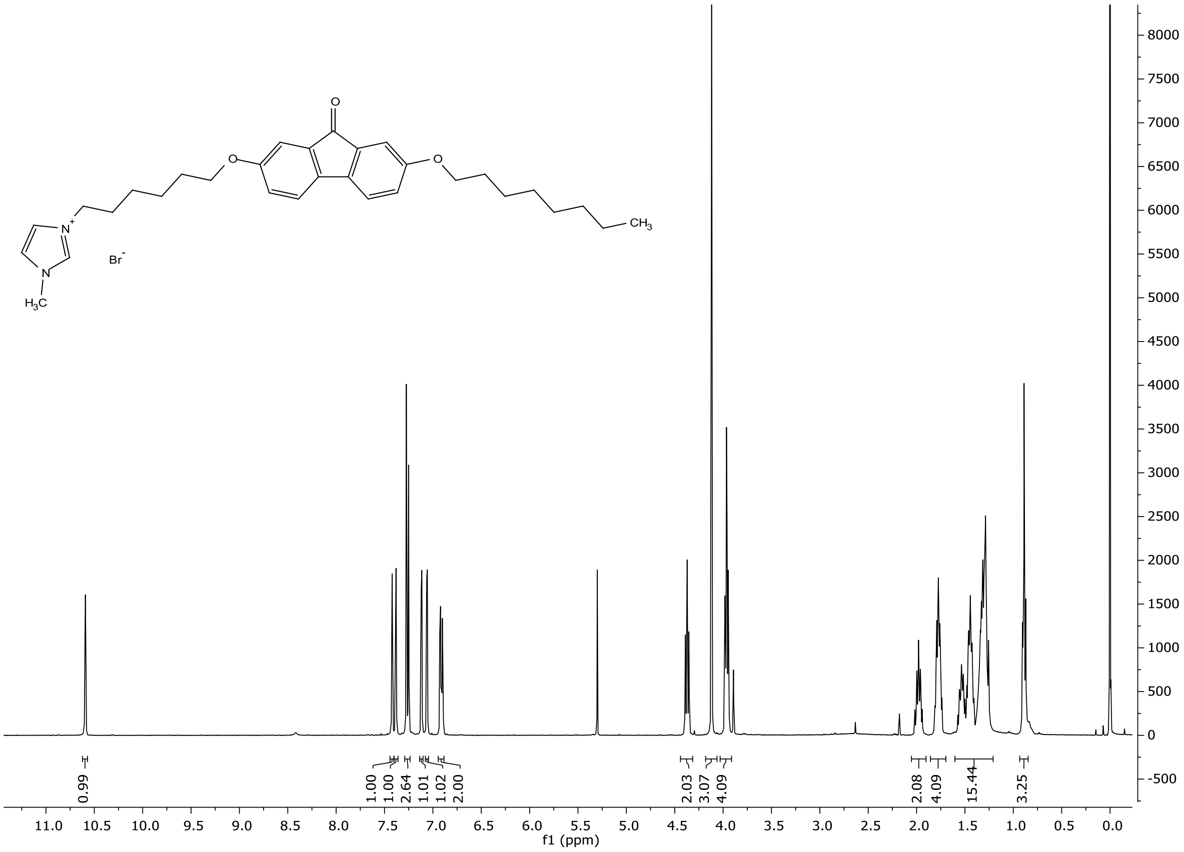

O
O
O
N+
N
H3C
Br-
CH3
0.99
1.00
1.00
2.64
1.01
1.02
2.00
2.03
3.07
4.09
2.08
4.09
15.44
3.25
f1 (ppm)

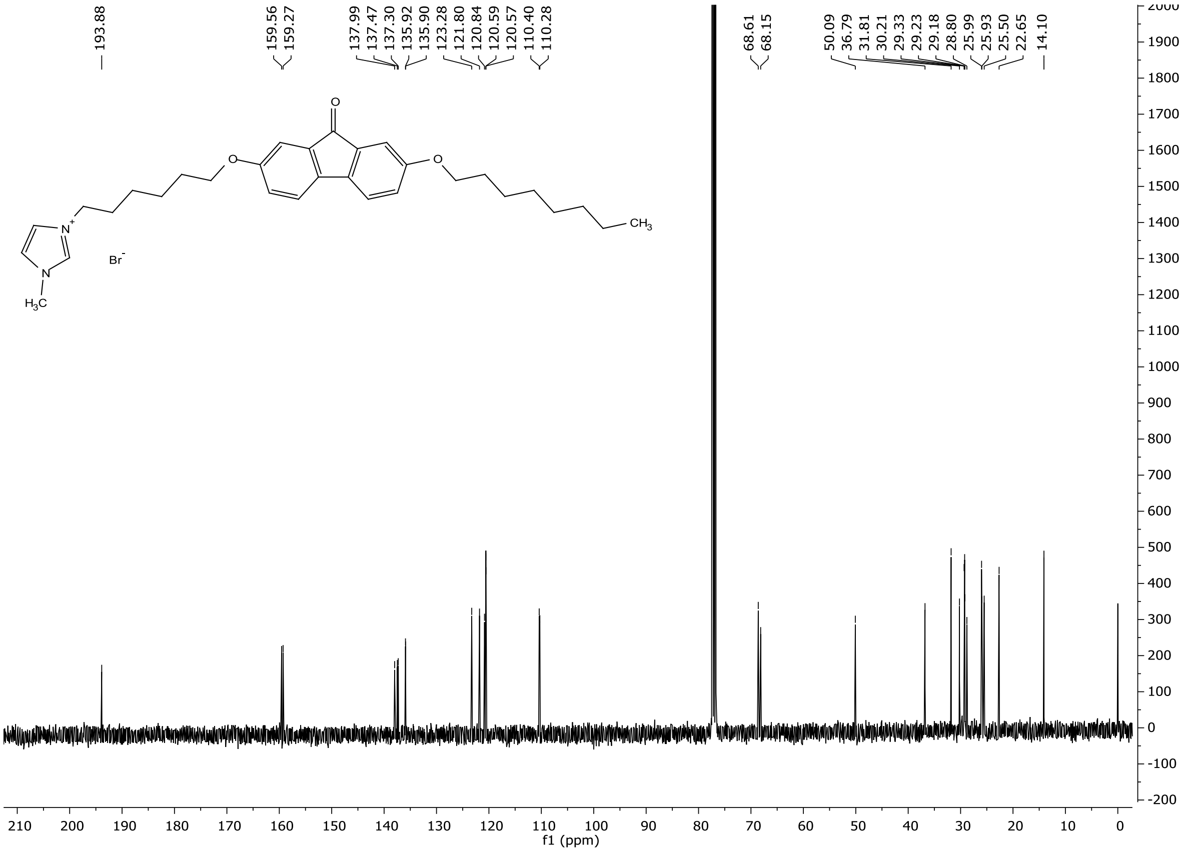

193.88
159.56
159.27
137.99
137.47
137.30
135.92
135.90
123.28
121.80
120.84
120.59
120.57
110.40
110.28
68.61
68.15
50.09
36.79
31.81
30.21
29.33
29.23
29.18
28.80
25.99
25.93
25.50
22.65
14.10
O
O
O
N+
N
H3C
Br-
CH3
f1 (ppm)

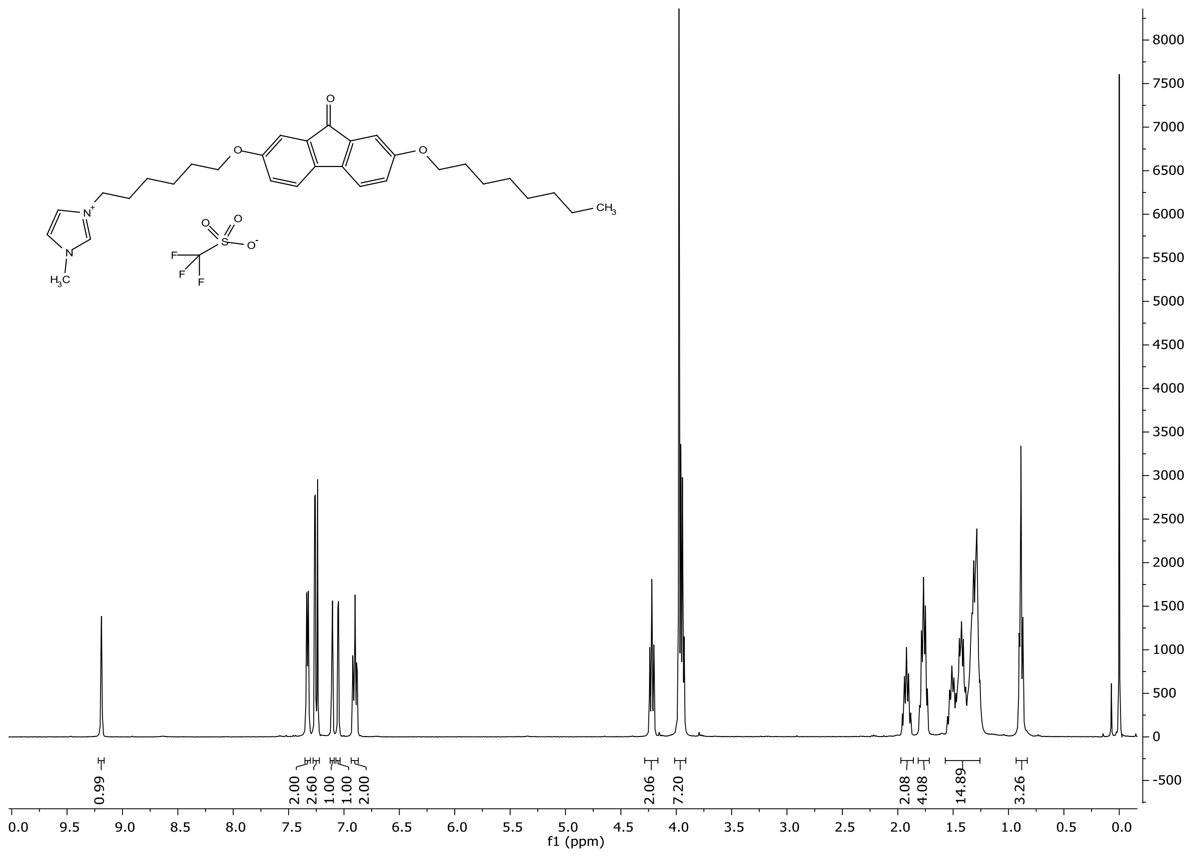

O
O
O
N+
N
H3C
CH3
O
O
S
O-
F
F
F
0.99
2.00
2.60
1.00
1.00
2.00
2.06
7.20
2.08
4.08
14.89
3.26
f1 (ppm)

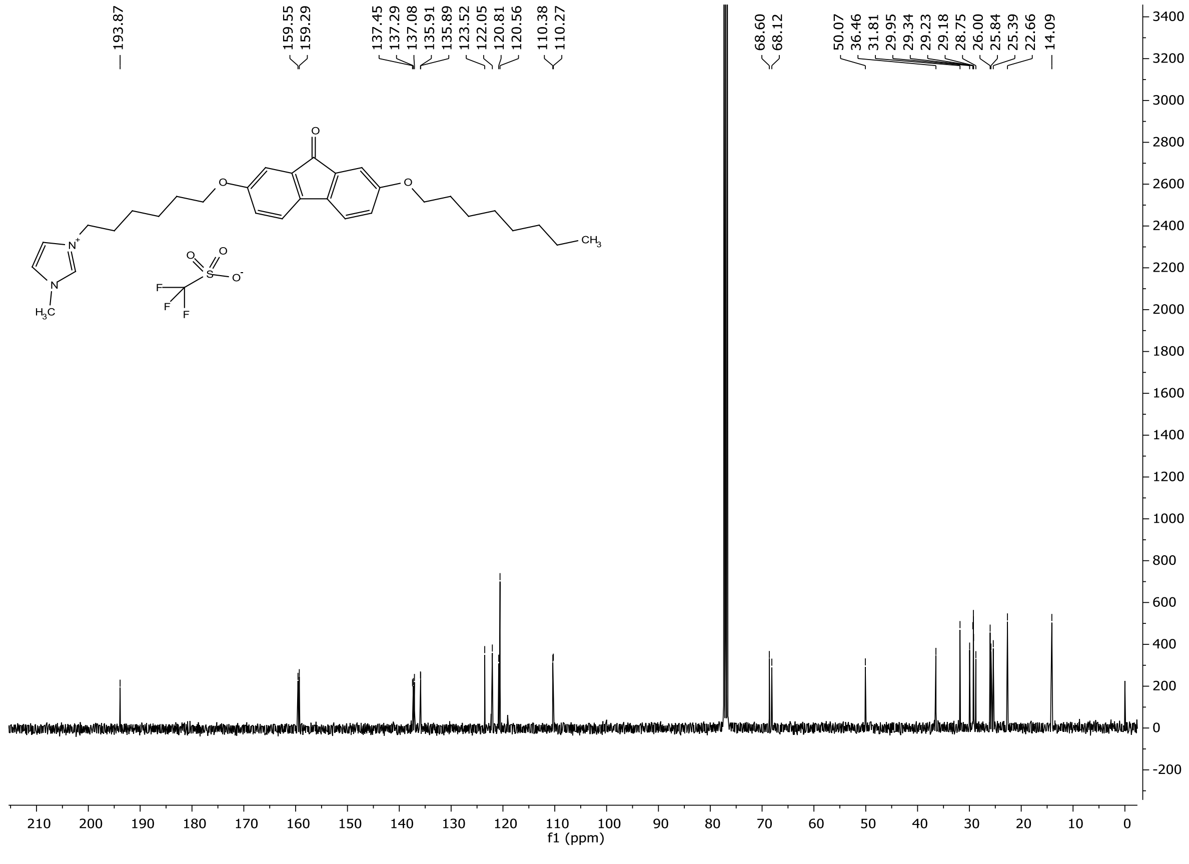

193.87
159.55
159.29
137.45
137.29
137.08
135.91
135.89
123.52
122.05
120.81
120.56
110.38
110.27
68.60
68.12
50.07
36.46
31.81
29.95
29.34
29.23
29.18
28.75
26.00
25.84
25.39
22.66
14.09
f1 (ppm)

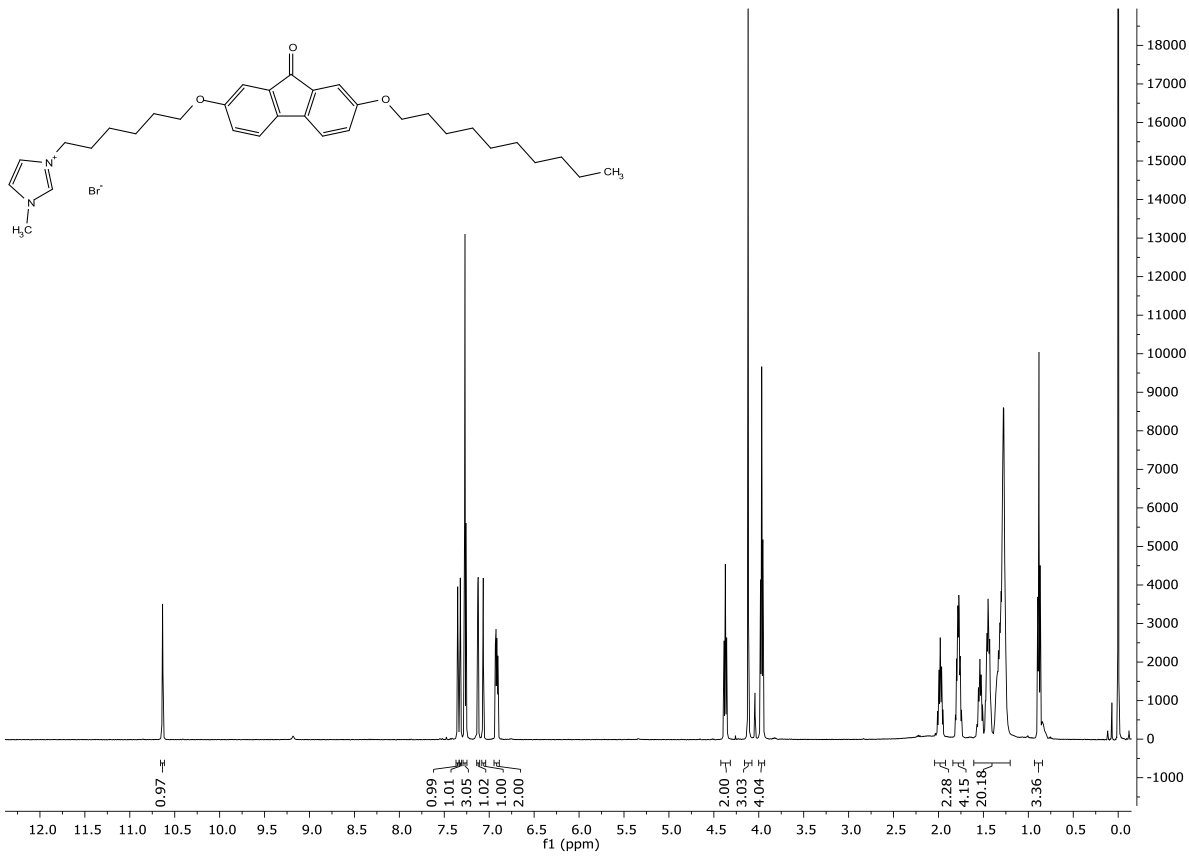

Br⁻
CH3
H3C
0.97
0.99
1.01
3.05
1.02
1.00
2.00
2.00
3.03
4.04
2.28
4.15
20.18
3.36
f1 (ppm)

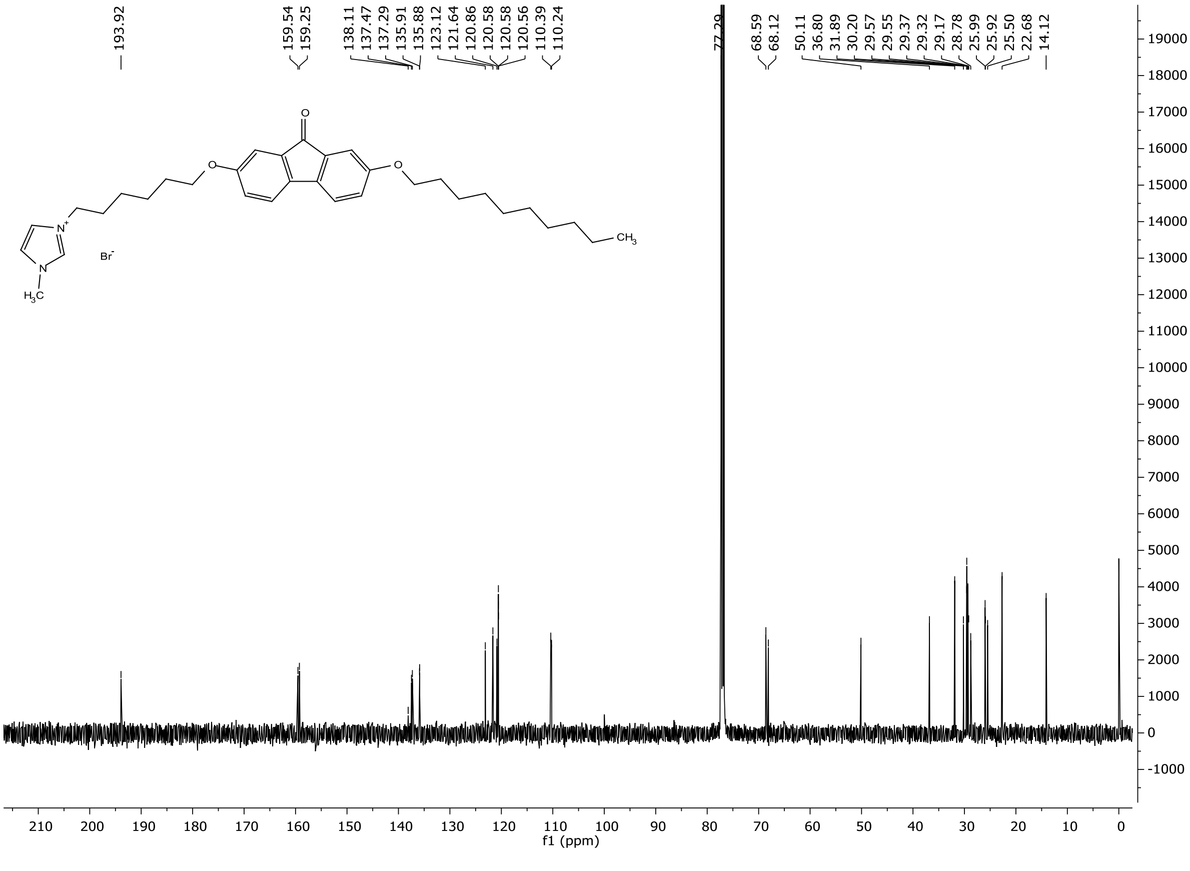

193.92
159.54
159.25
138.11
137.47
137.29
135.91
135.88
123.12
121.64
120.86
120.58
120.58
120.56
110.39
110.24
77.29
68.59
68.12
50.11
36.80
31.89
30.20
29.57
29.55
29.37
29.32
29.17
28.78
25.99
25.92
25.50
22.68
14.12
f1 (ppm)

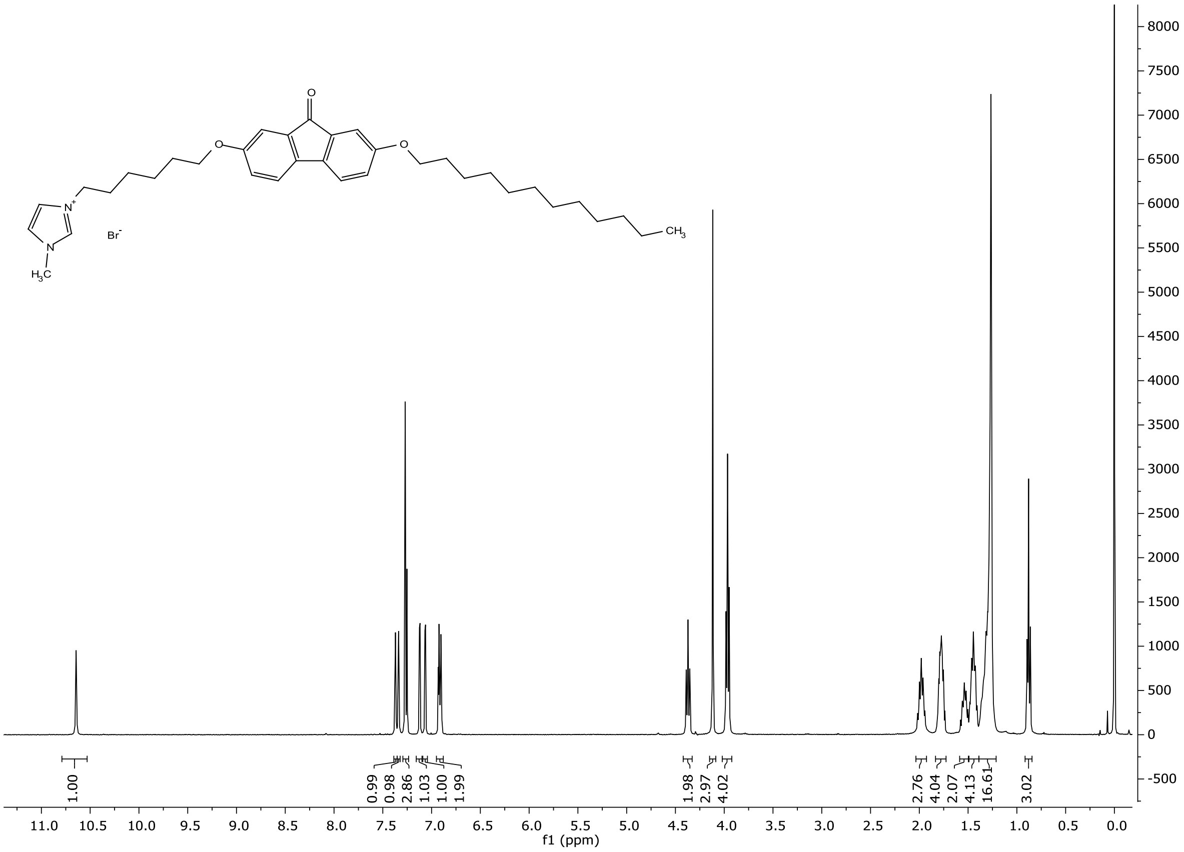

O
O
O
N+
N
Br-
H3C
CH3
1.00
0.99
0.98
2.86
1.03
1.00
1.99
1.98
2.97
4.02
2.76
4.04
2.07
4.13
16.61
3.02
11.0 10.5 10.0 9.5 9.0 8.5 8.0 7.5 7.0 6.5 6.0 5.5 5.0 4.5 4.0 3.5 3.0 2.5 2.0 1.5 1.0 0.5 0.0
f1 (ppm)
8000 7500 7000 6500 6000 5500 5000 4500 4000 3500 3000 2500 2000 1500 1000 500 0 -500

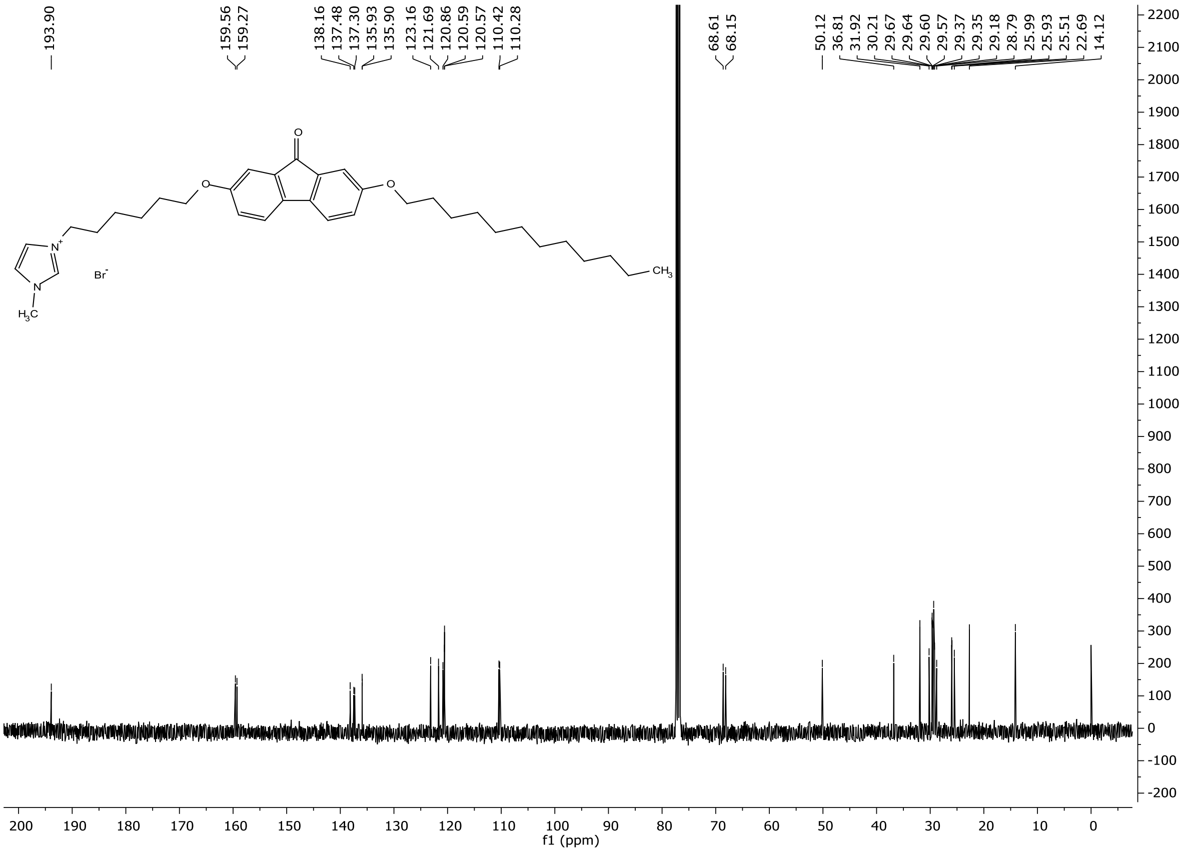

193.90
159.56
159.27
138.16
137.48
137.30
135.93
135.90
123.16
121.69
120.86
120.59
120.57
110.42
110.28
68.61
68.15
50.12
36.81
31.92
30.21
29.67
29.64
29.60
29.57
29.37
29.35
29.18
28.79
25.99
25.93
25.51
22.69
14.12
O
O
O
N+
N
Br-
H3C
CH3
200 190 180 170 160 150 140 130 120 110 100 90 80 70 60 50 40 30 20 10 0
f1 (ppm)
2200 2100 2000 1900 1800 1700 1600 1500 1400 1300 1200 1100 1000 900 800 700 600 500 400 300 200 100 0 -100 -200

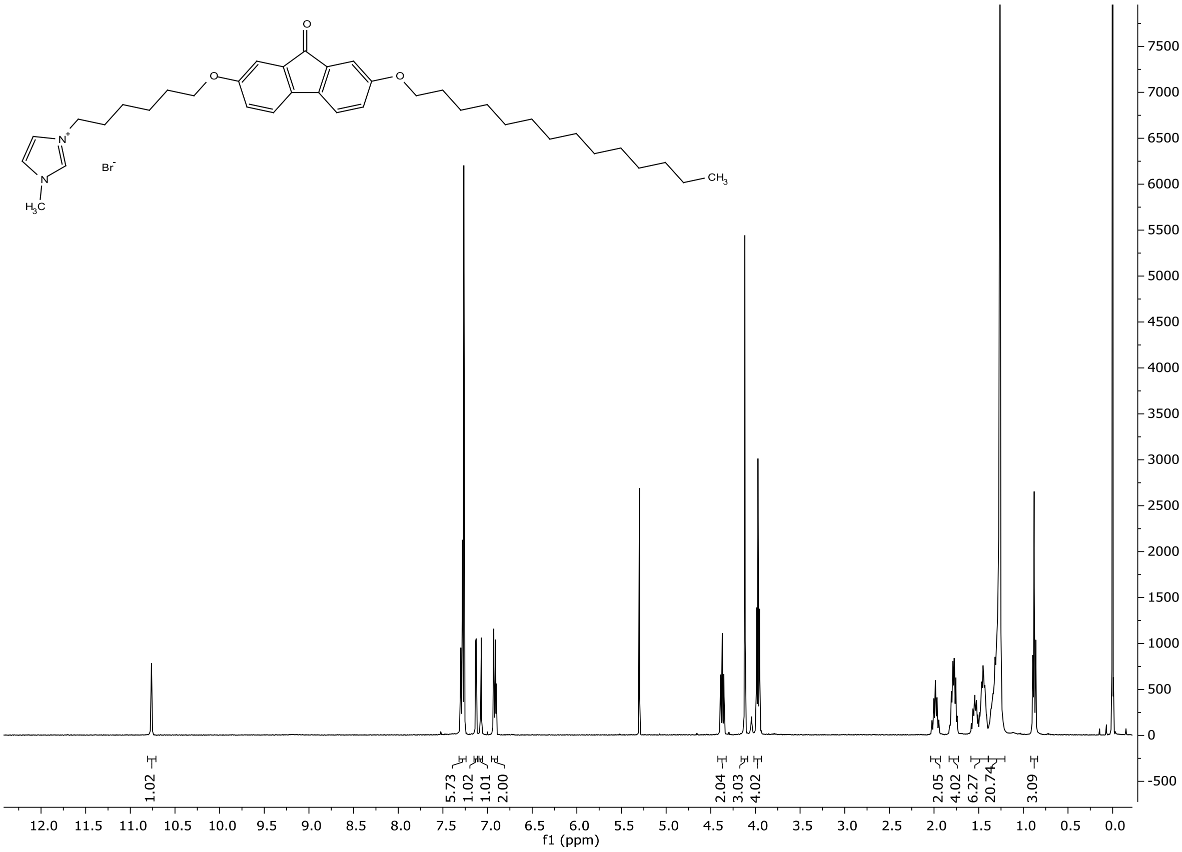

O
Br-
CH_3
H_3C
1.02
5.73
1.02
1.01
2.00
2.04
3.03
4.02
2.05
4.02
6.27
20.74
3.09
f1 (ppm)

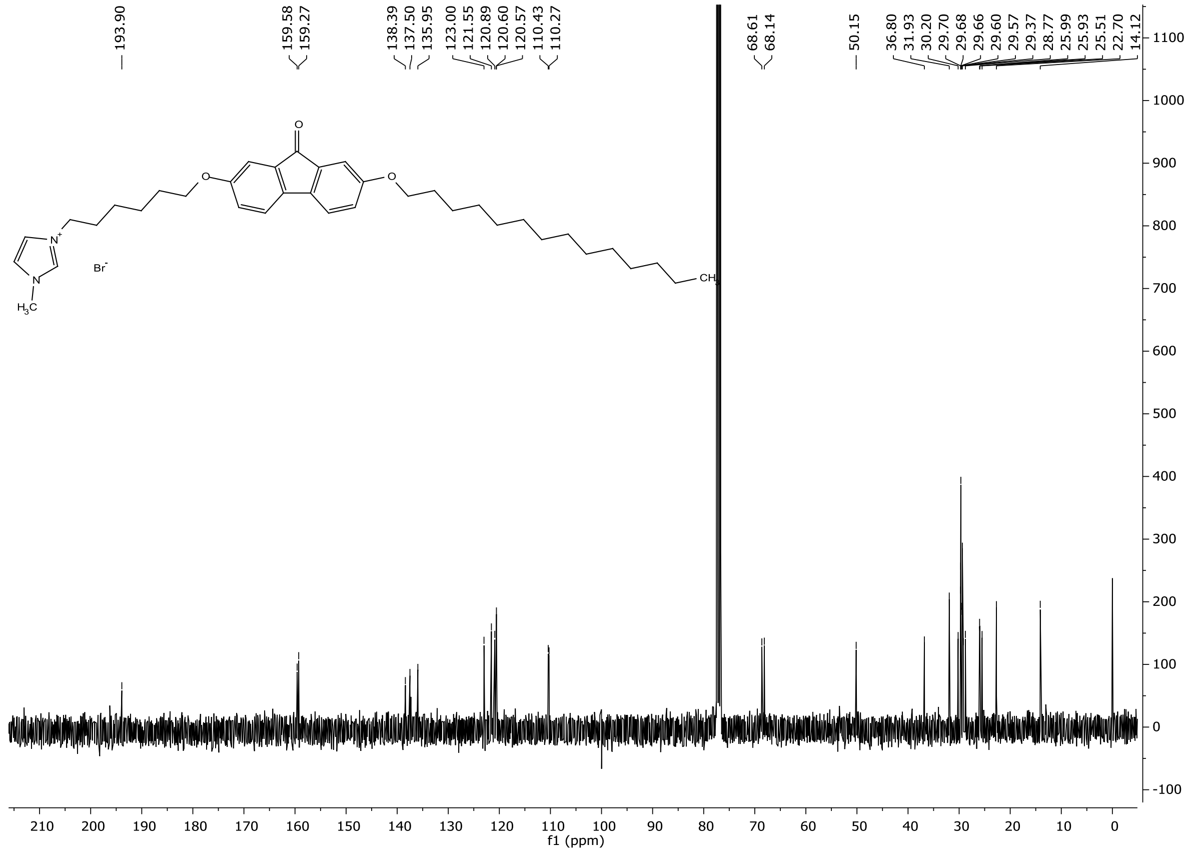

193.90
159.58
159.27
138.39
137.50
135.95
123.00
121.55
120.89
120.60
120.57
110.43
110.27
68.61
68.14
50.15
36.80
31.93
30.20
29.70
29.68
29.66
29.60
29.57
29.37
28.77
25.99
25.93
25.51
22.70
14.12
Br-
H_3C
f1 (ppm)

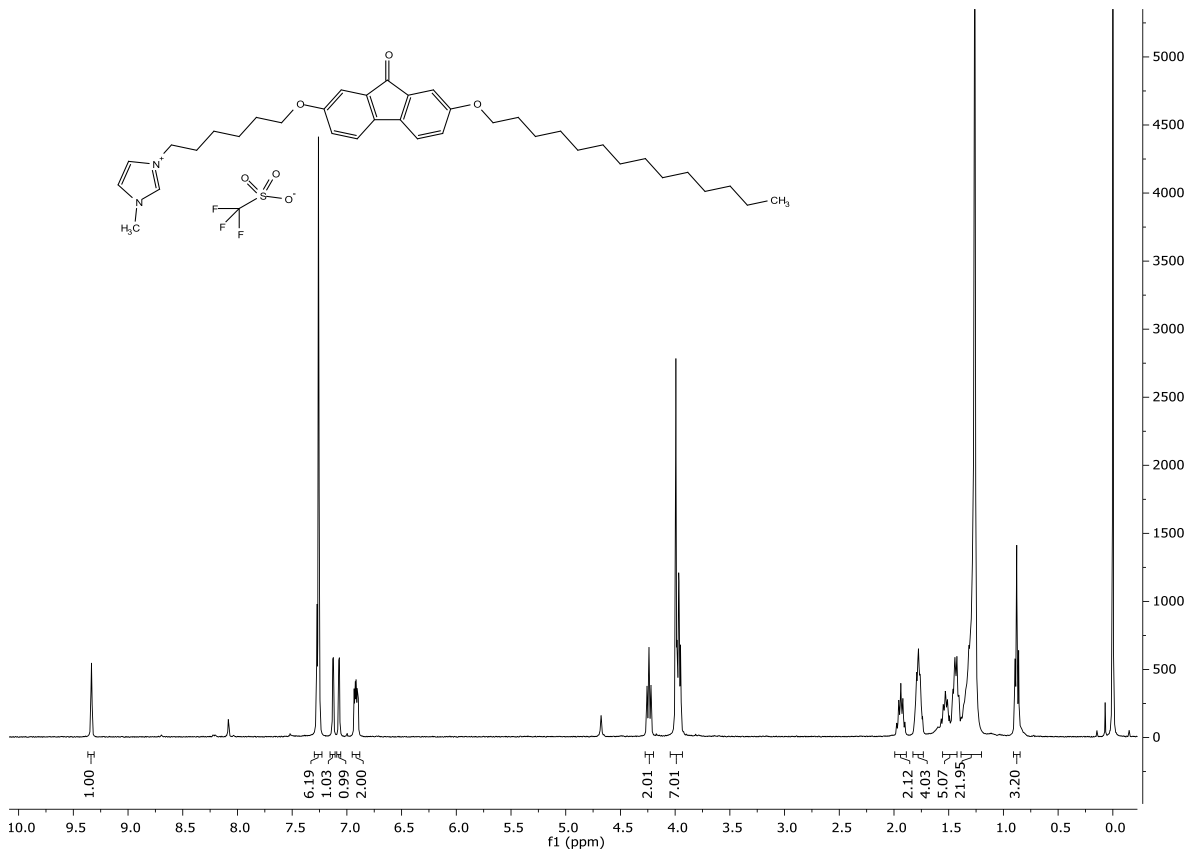

1.00
6.19
1.03
0.99
2.00
2.01
7.01
2.12
4.03
5.07
21.95
3.20
f1 (ppm)

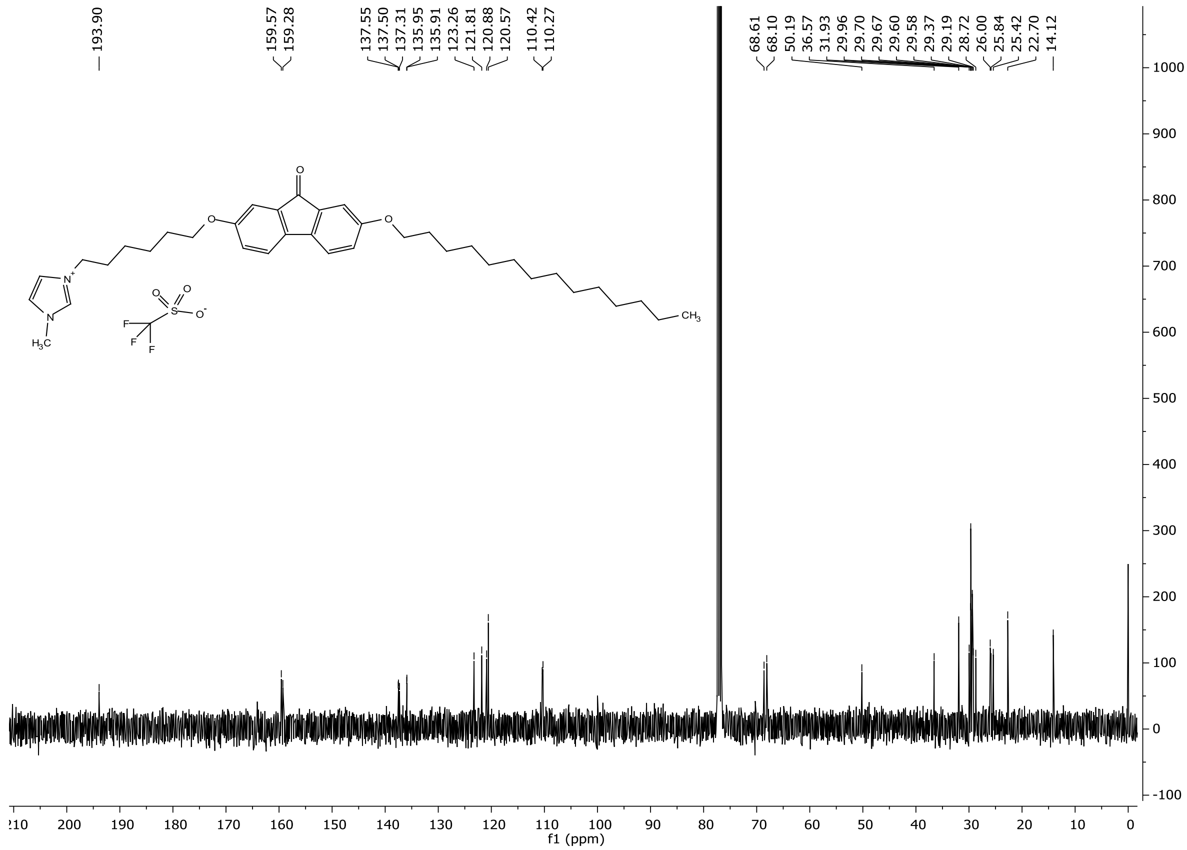

193.90
159.57
159.28
137.55
137.50
137.31
135.95
135.91
123.26
121.81
120.88
120.57
110.42
110.27
68.61
68.10
50.19
36.57
31.93
29.96
29.70
29.67
29.60
29.58
29.37
29.19
28.72
26.00
25.84
25.42
22.70
14.12
f1 (ppm)

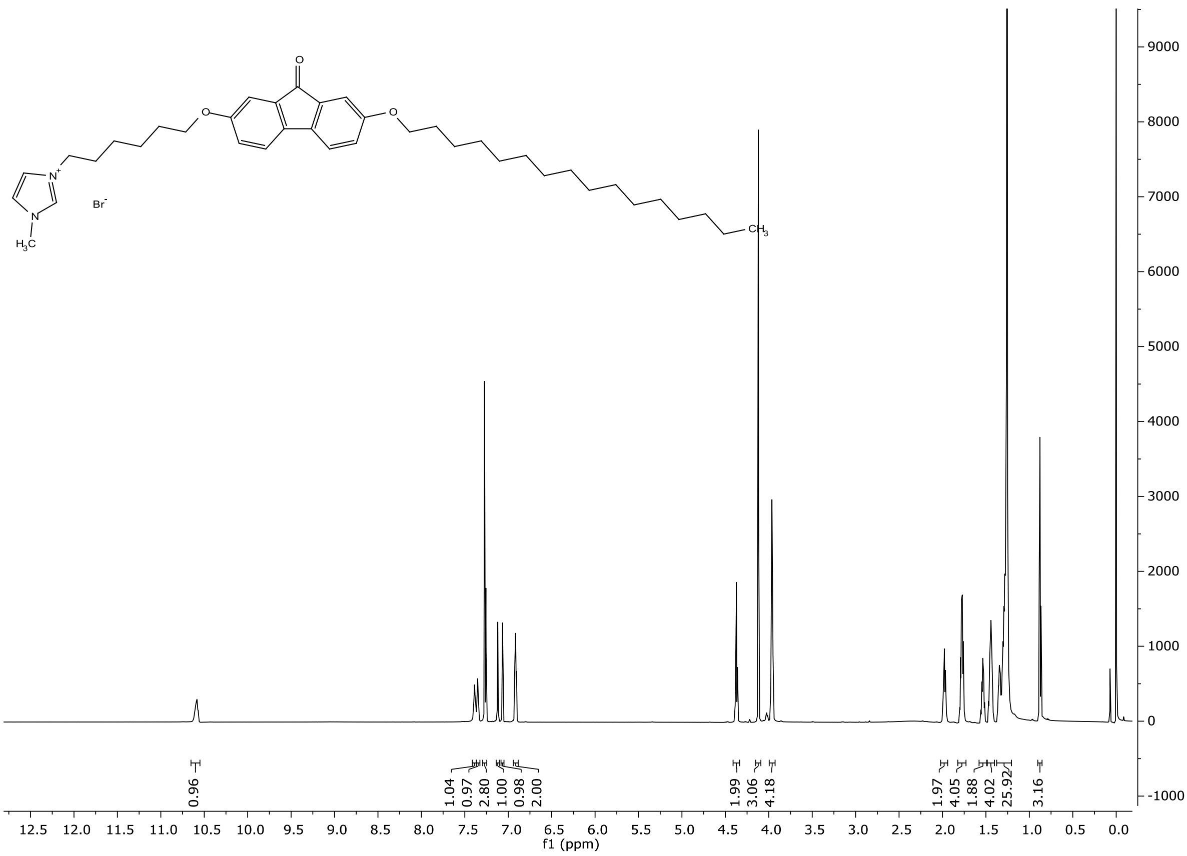
O
O
O
N+
N
H3C
Br-
CH3
0.96
1.04
0.97
2.80
1.00
0.98
2.00
1.99
3.06
4.18
1.97
4.05
1.88
4.02
25.92
3.16
f1 (ppm)

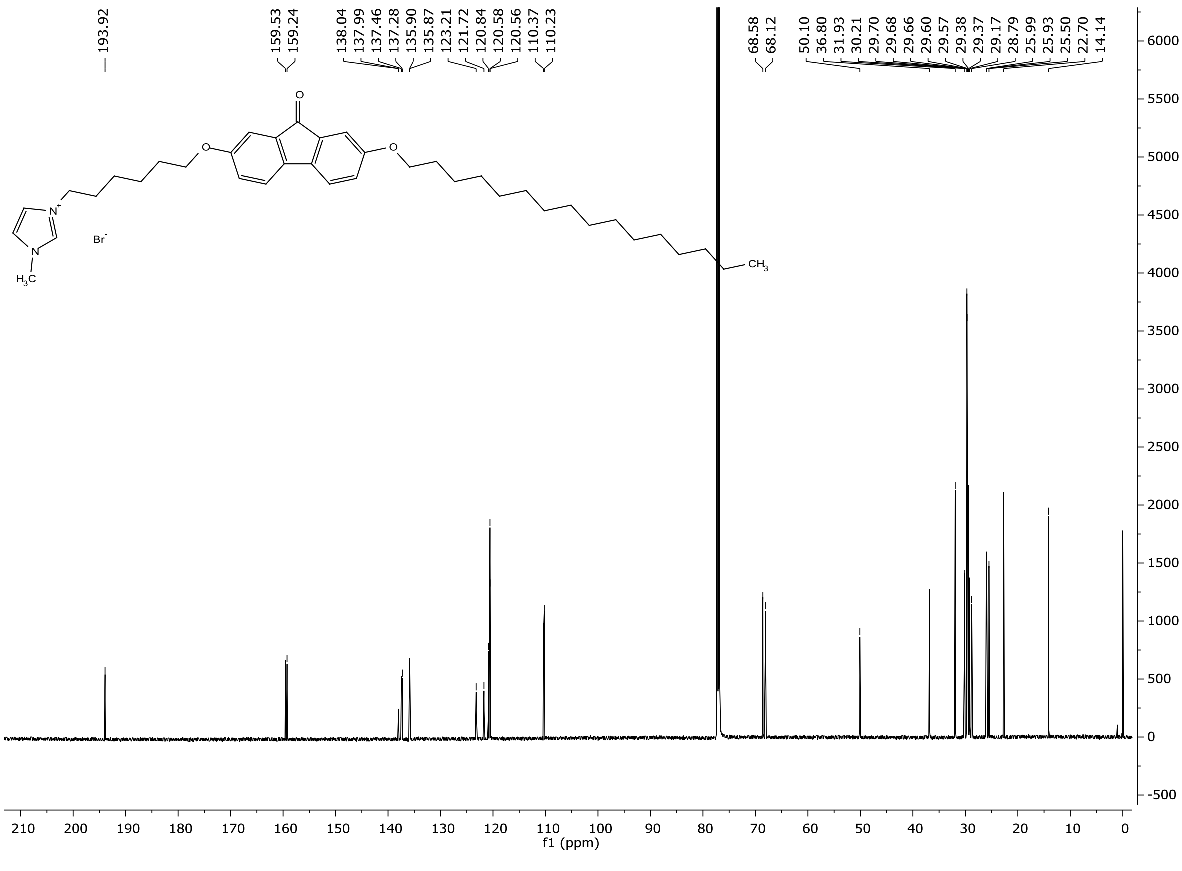
193.92
159.53
159.24
138.04
137.99
137.46
137.28
135.90
135.87
123.21
121.72
120.84
120.58
120.56
110.37
110.23
68.58
68.12
50.10
36.80
31.93
30.21
29.70
29.68
29.66
29.60
29.57
29.38
29.37
29.17
28.79
25.99
25.93
25.50
22.70
14.14
Br-
CH3
H3C
f1 (ppm)

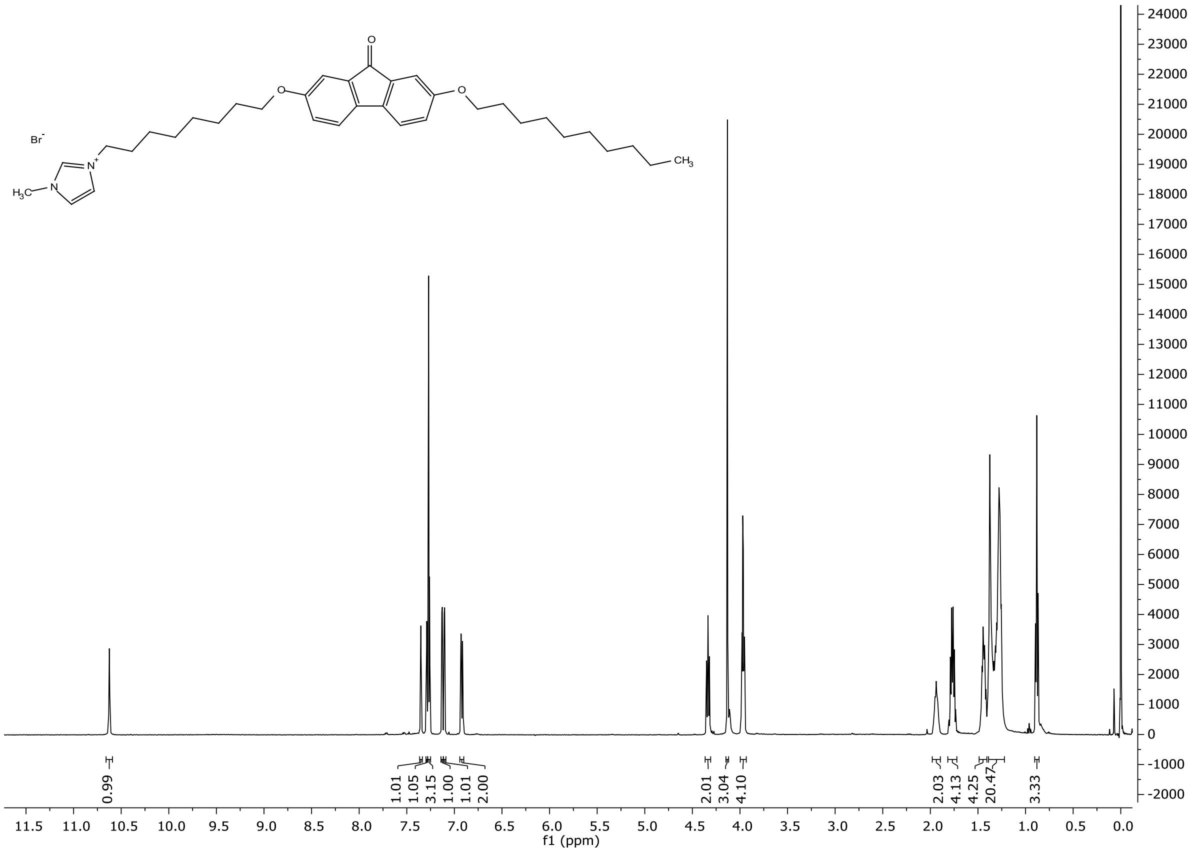
Br⁻
H3C
N
N+
O
CH3
0.99
1.01
1.05
3.15
1.00
1.01
2.00
2.01
3.04
4.10
2.03
4.13
4.25
20.47
3.33
f1 (ppm)

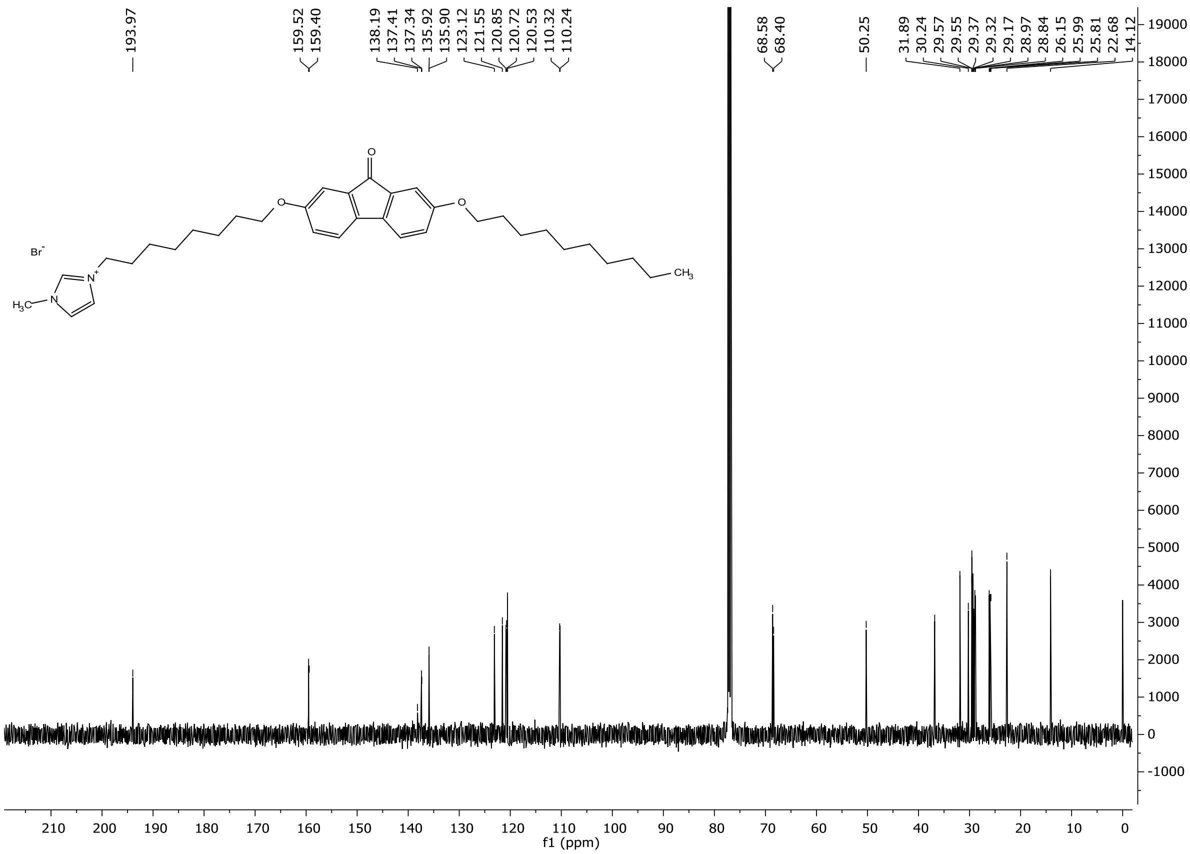
193.97
159.52
159.40
138.19
137.41
137.34
135.92
135.90
123.12
121.55
120.85
120.72
120.53
110.32
110.24
68.58
68.40
50.25
31.89
30.24
29.57
29.55
29.37
29.32
29.17
28.97
28.84
26.15
25.99
25.81
22.68
14.12
Br⁻
H3C
N
N+
O
CH3
f1 (ppm)

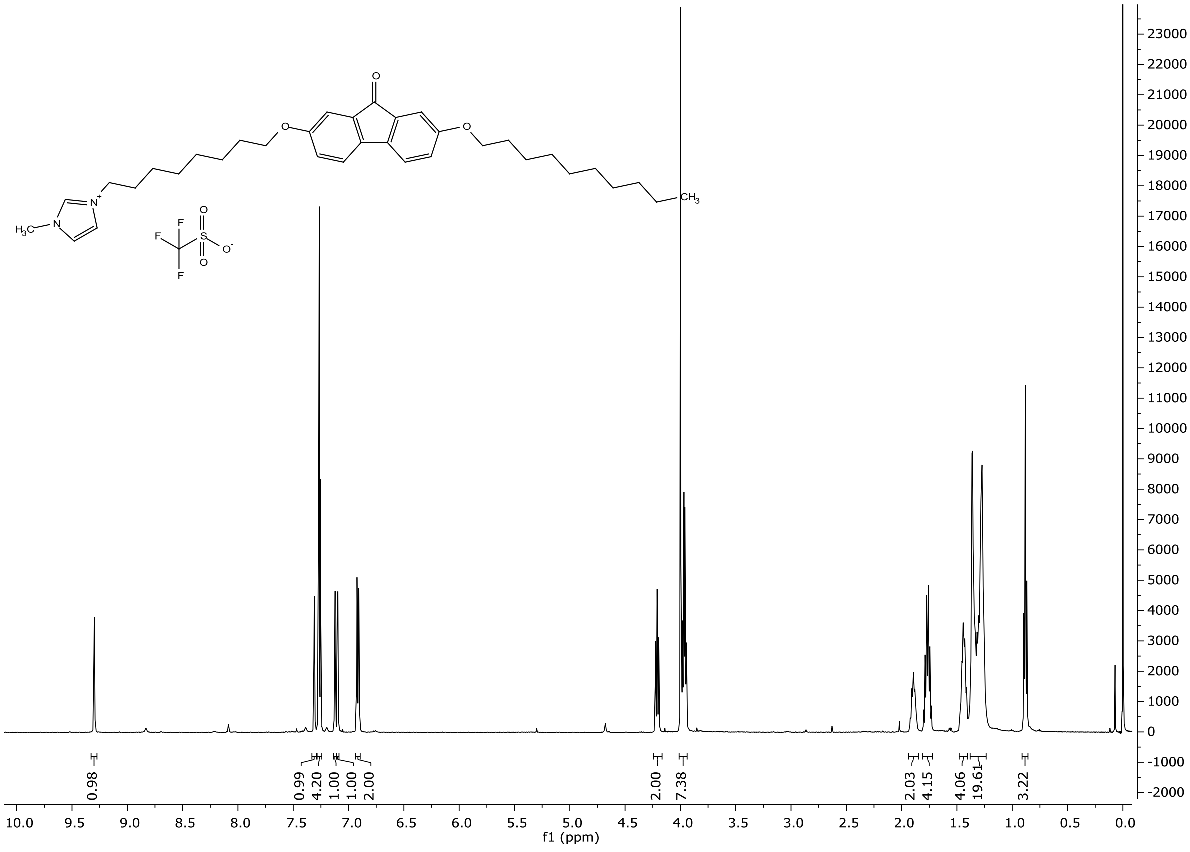

O
O
O
N
N+
H3C
CH3
F
F
F
S
O
O
O-
0.98
0.99
4.20
1.00
1.00
2.00
2.00
7.38
2.03
4.15
4.06
19.61
3.22
f1 (ppm)

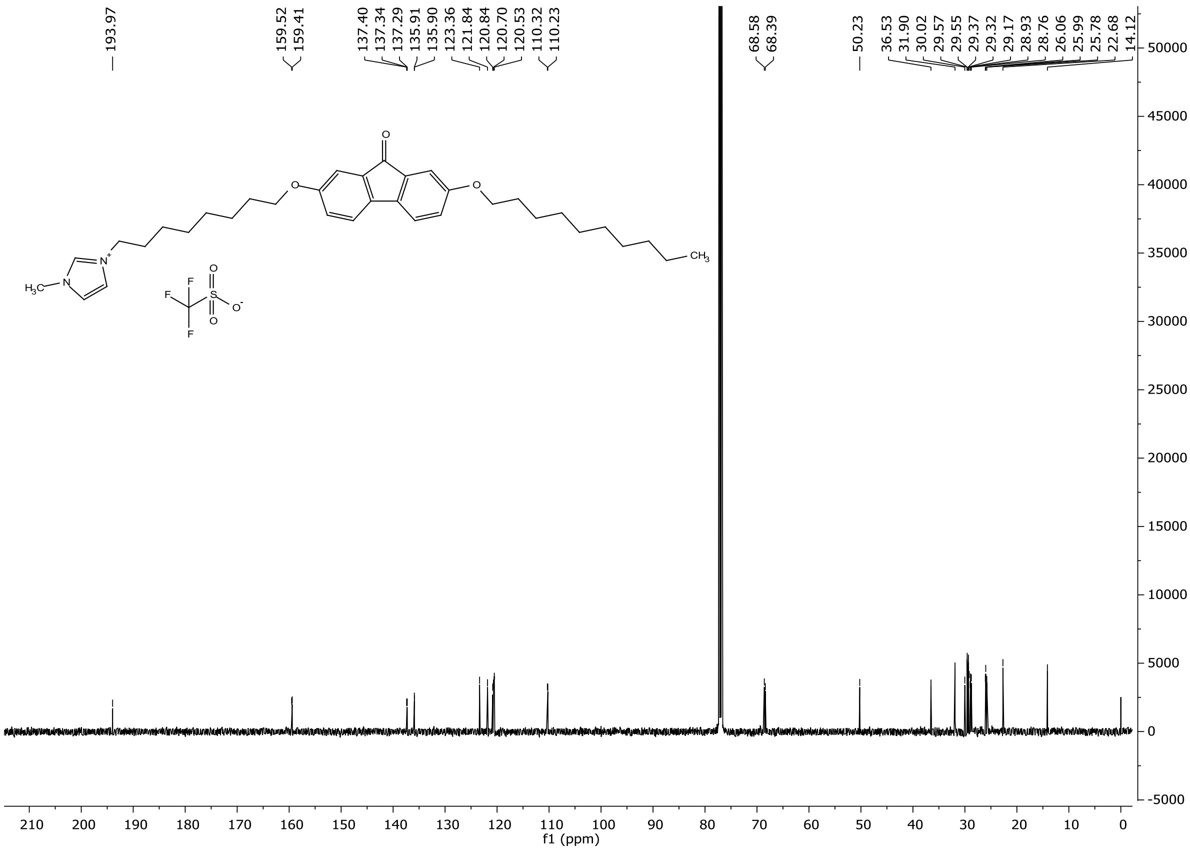

193.97
159.52
159.41
137.40
137.34
137.29
135.91
135.90
123.36
121.84
120.84
120.70
120.53
110.32
110.23
68.58
68.39
50.23
36.53
31.90
30.02
29.57
29.55
29.37
29.32
29.17
28.93
28.76
26.06
25.99
25.78
22.68
14.12
O
O
O
N
N+
H3C
CH3
F
F
F
S
O
O
O-
f1 (ppm)

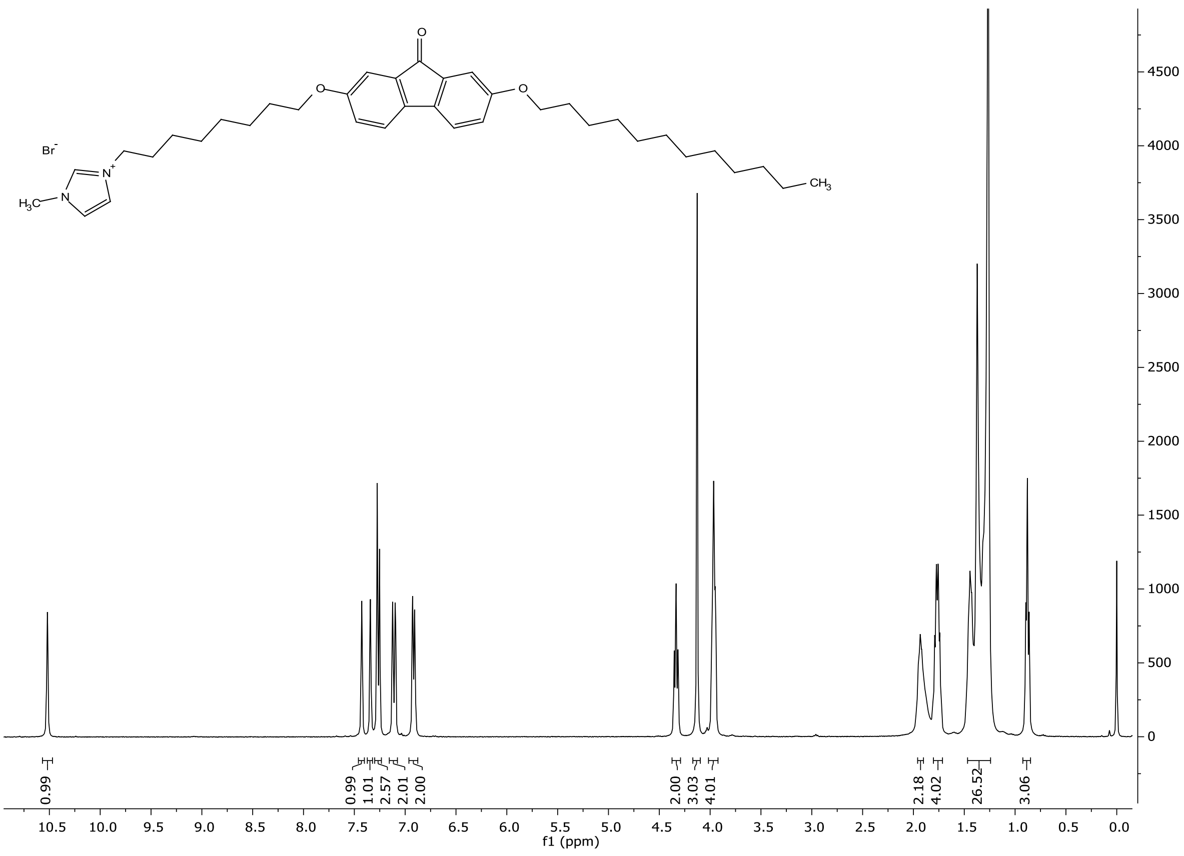

Br⁻
H_3C
N
N⁺
O
O
O
CH_3
0.99
0.99
1.01
2.57
2.01
2.00
2.00
3.03
4.01
2.18
4.02
26.52
3.06
f1 (ppm)

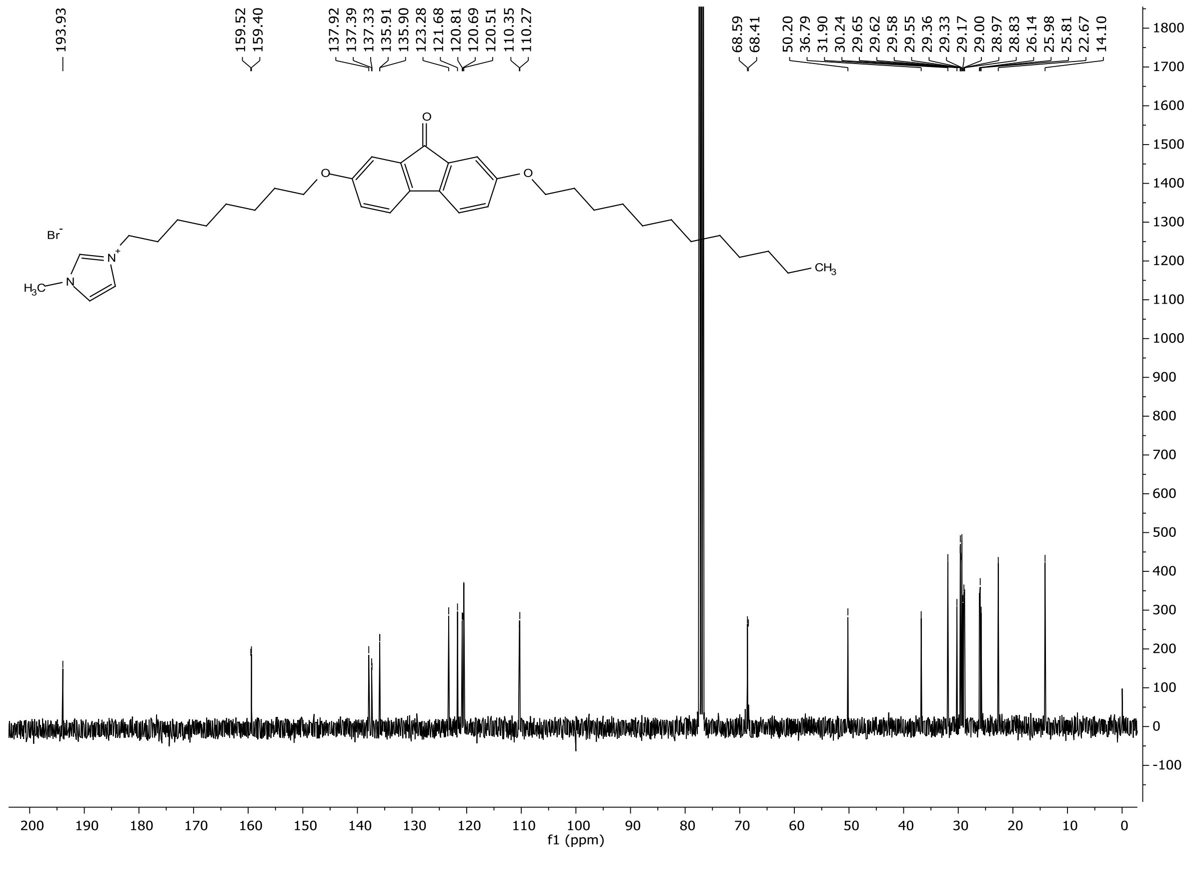

193.93
159.52
159.40
137.92
137.39
137.33
135.91
135.90
123.28
121.68
120.81
120.69
120.51
110.35
110.27
68.59
68.41
50.20
36.79
31.90
30.24
29.65
29.62
29.58
29.55
29.36
29.33
29.17
29.00
28.97
28.83
26.14
25.98
25.81
22.67
14.10
Br⁻
H_3C
N
N⁺
O
O
O
CH_3
f1 (ppm)

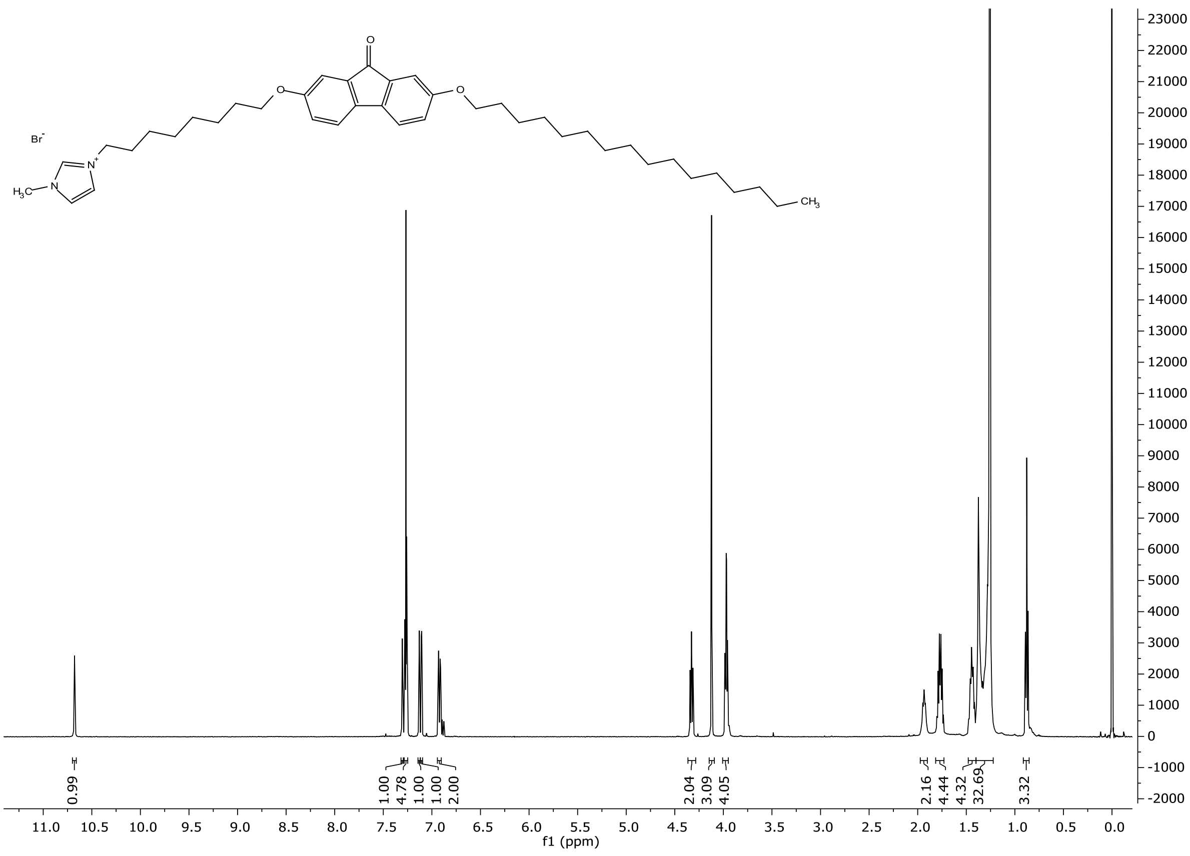

Br⁻
H3C
N
N+
O
O
O
CH3
0.99
1.00
4.78
1.00
1.00
2.00
2.04
3.09
4.05
2.16
4.44
4.32
32.69
3.32
f1 (ppm)

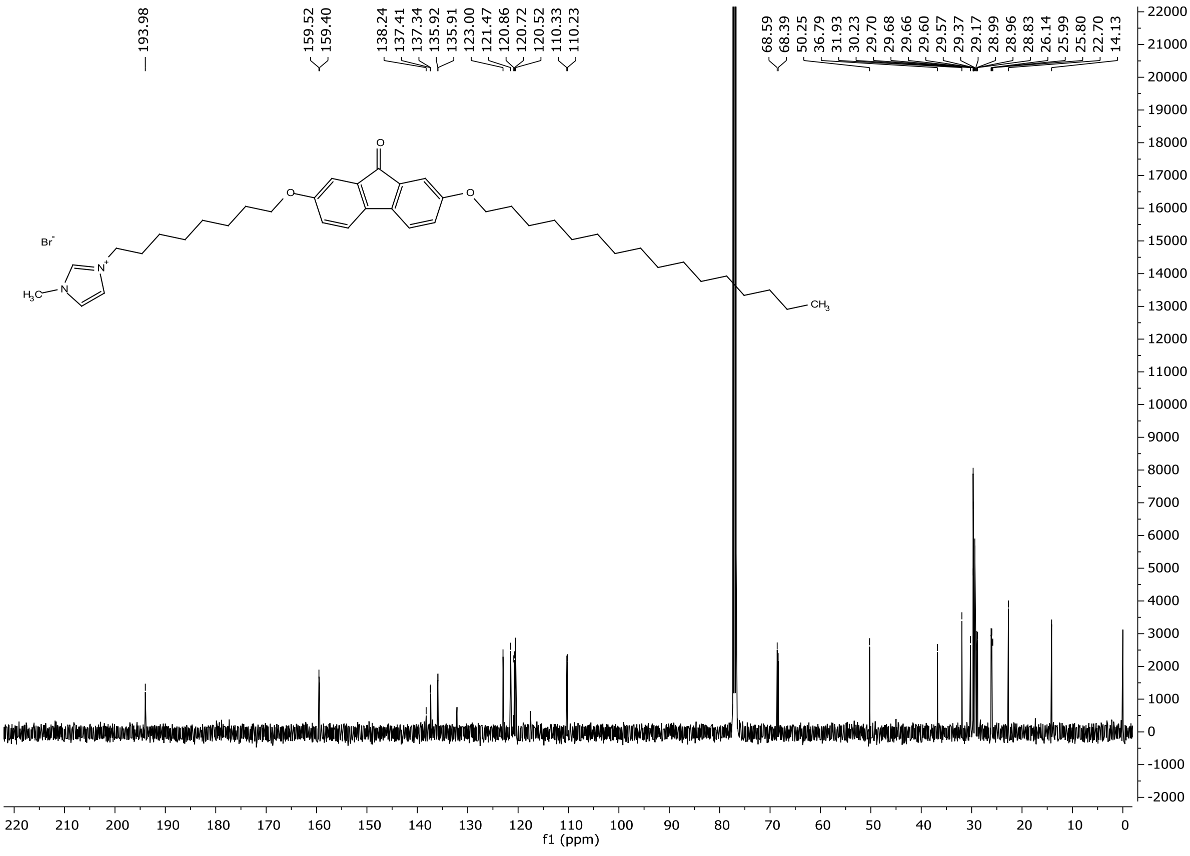

193.98
159.52
159.40
138.24
137.41
137.34
135.92
135.91
123.00
121.47
120.86
120.72
120.52
110.33
110.23
68.59
68.39
50.25
36.79
31.93
30.23
29.70
29.68
29.66
29.60
29.57
29.37
29.17
28.99
28.96
28.83
26.14
25.99
25.80
22.70
14.13
Br⁻
H3C
N
N+
O
O
O
CH3
f1 (ppm)

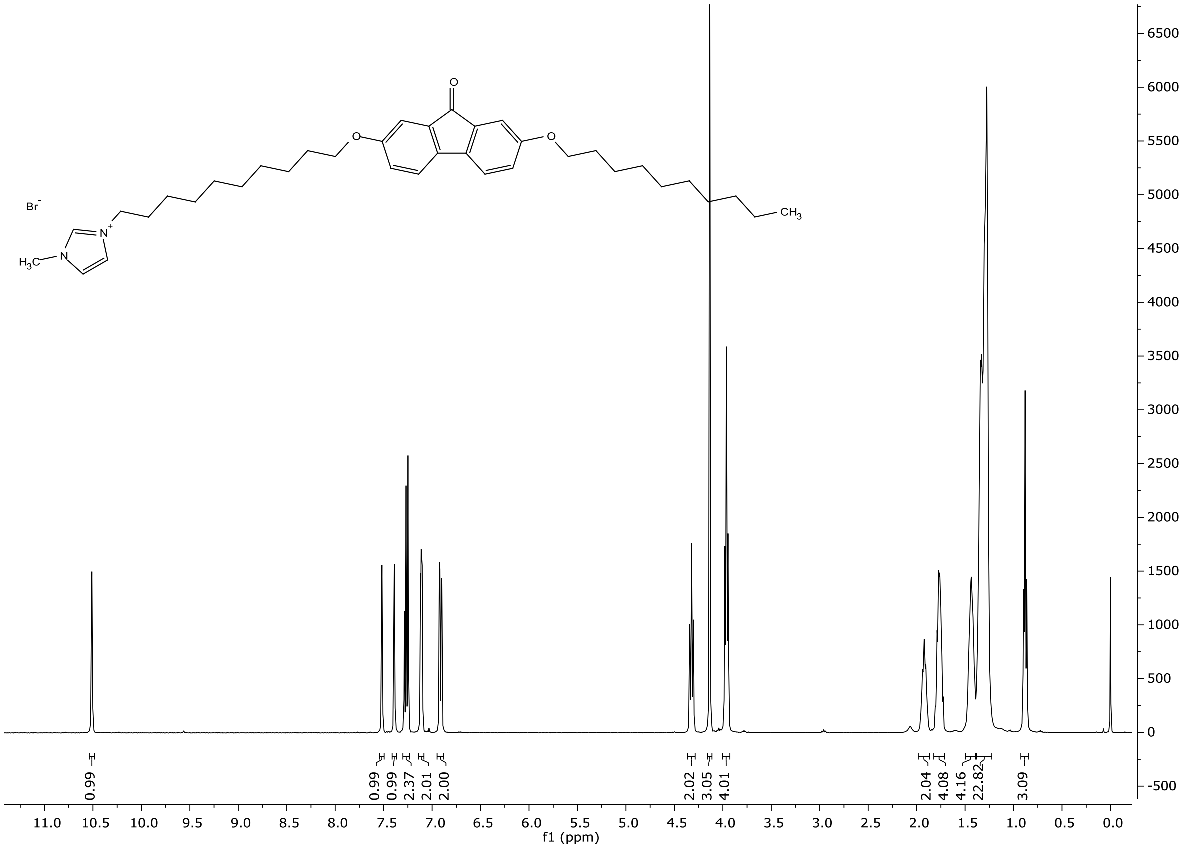

Br-
H3C
CH3
O
f1 (ppm)
0.99
0.99
0.99
2.37
2.01
2.00
2.02
3.05
4.01
2.04
4.08
4.16
22.82
3.09

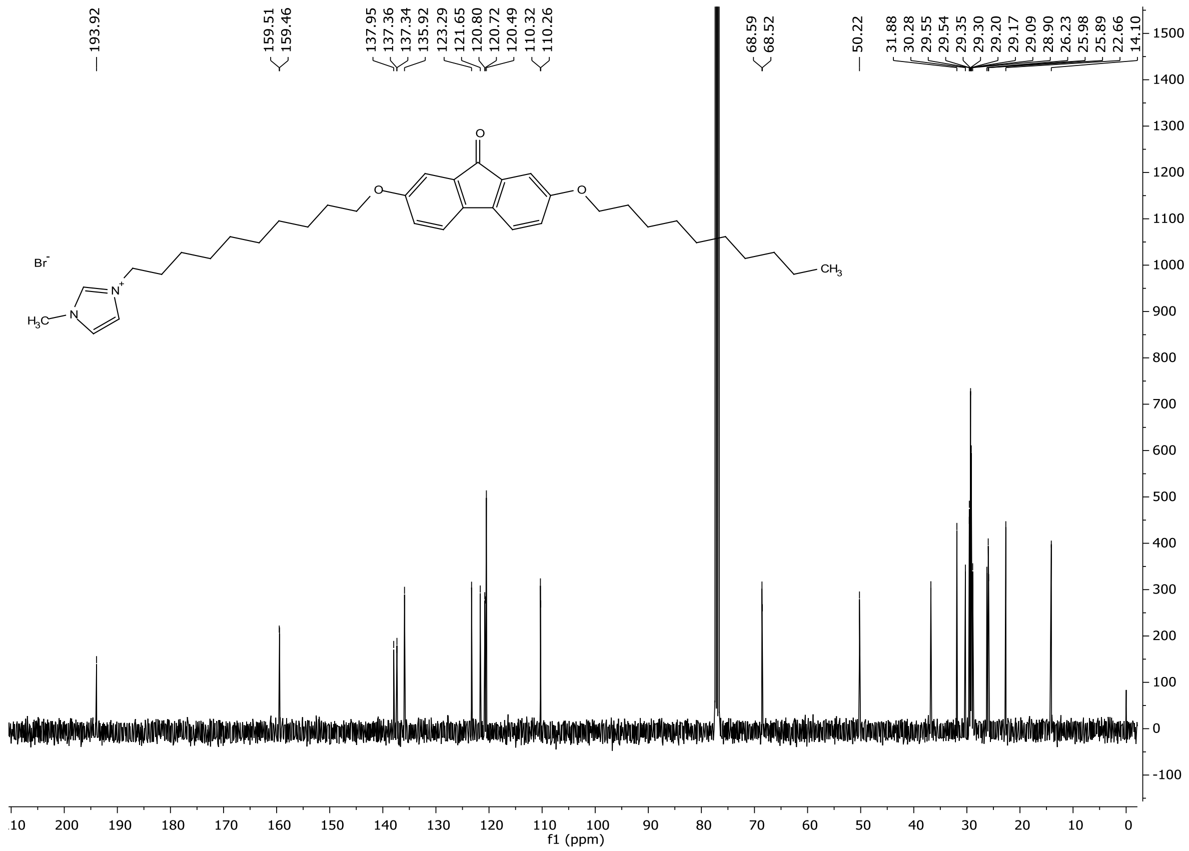

193.92
159.51
159.46
137.95
137.36
137.34
135.92
123.29
121.65
120.80
120.72
120.49
110.32
110.26
68.59
68.52
50.22
31.88
30.28
29.55
29.54
29.35
29.30
29.20
29.17
29.09
28.90
26.23
25.98
25.89
22.66
14.10
Br-
H3C
CH3
O
f1 (ppm)

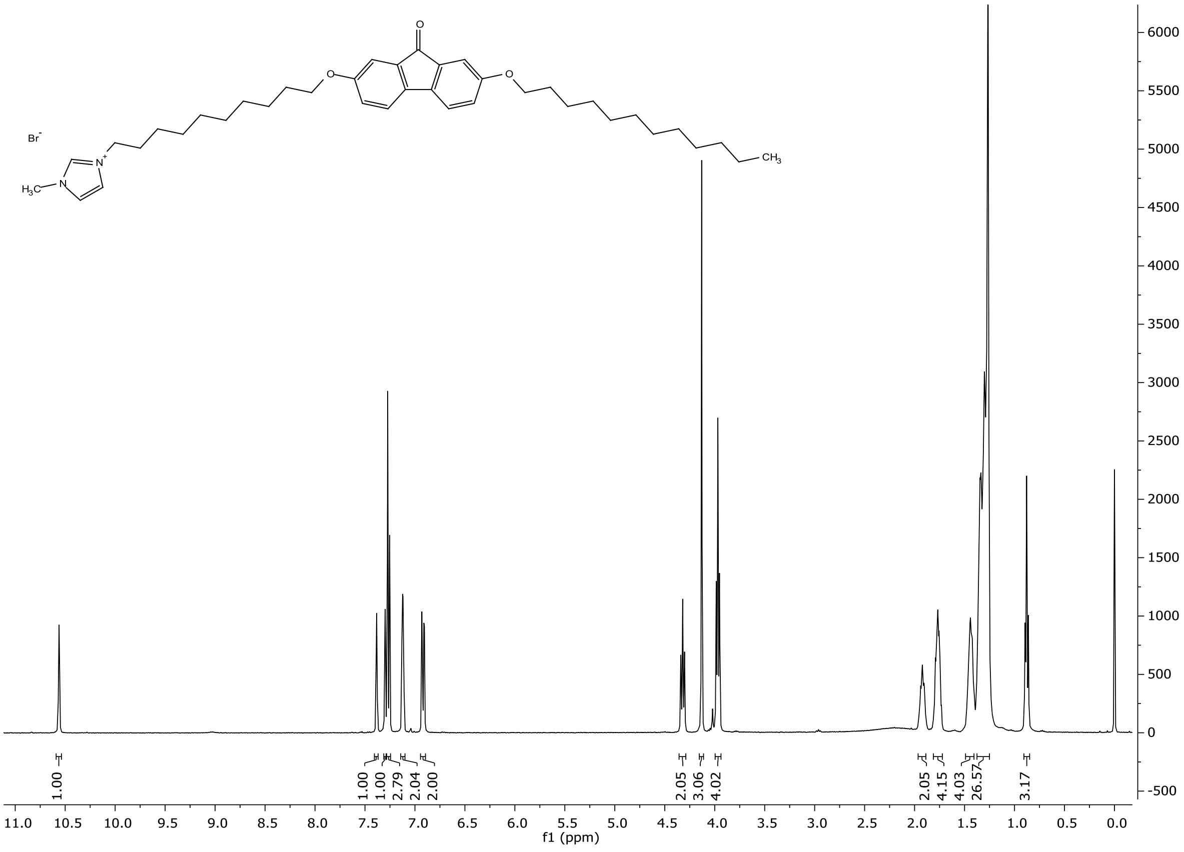

Br⁻
H3C
N
N+
O
O
O
CH3
1.00
1.00
1.00
2.79
2.04
2.00
2.05
3.06
4.02
2.05
4.15
4.03
26.57
3.17
f1 (ppm)

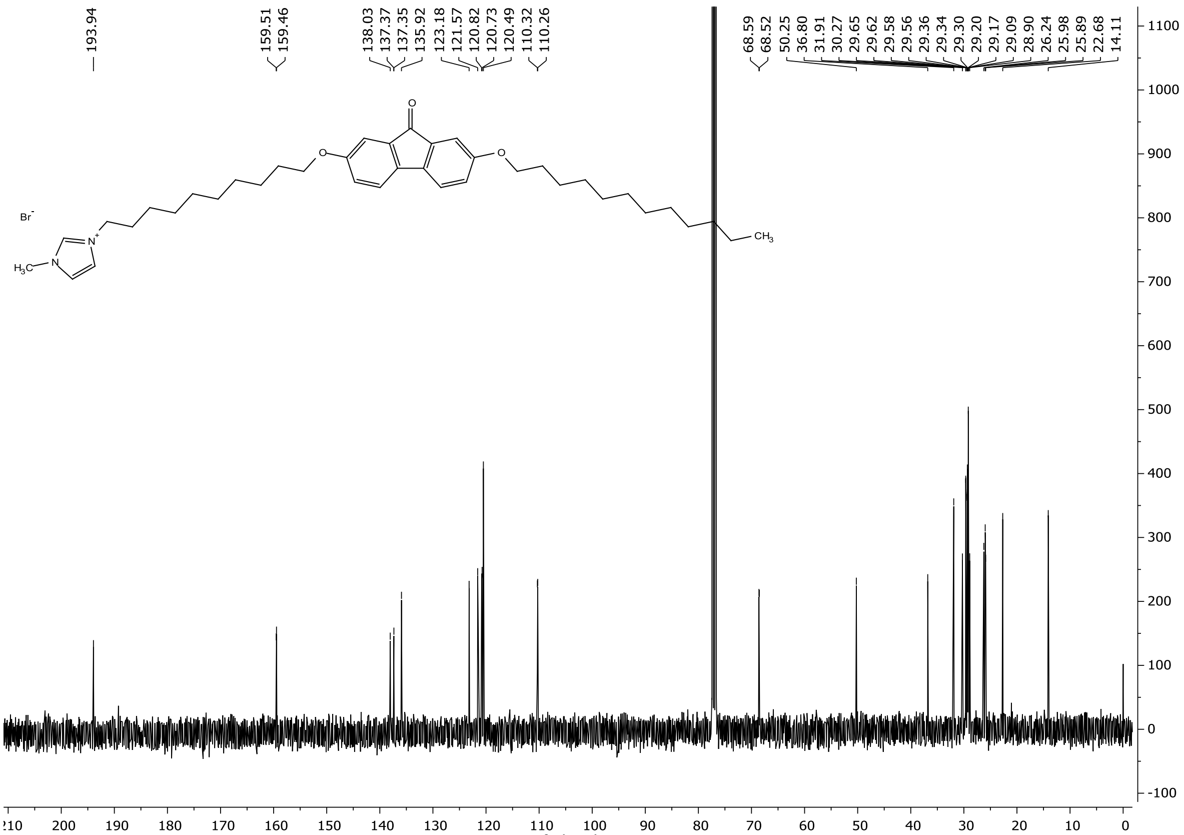

193.94
159.51
159.46
138.03
137.37
137.35
135.92
123.18
121.57
120.82
120.73
120.49
110.32
110.26
68.59
68.52
50.25
36.80
31.91
30.27
29.65
29.62
29.58
29.56
29.36
29.34
29.30
29.20
29.17
29.09
28.90
26.24
25.98
25.89
22.68
14.11
Br⁻
H3C
N
N+
O
O
O
CH3
f1 (ppm)

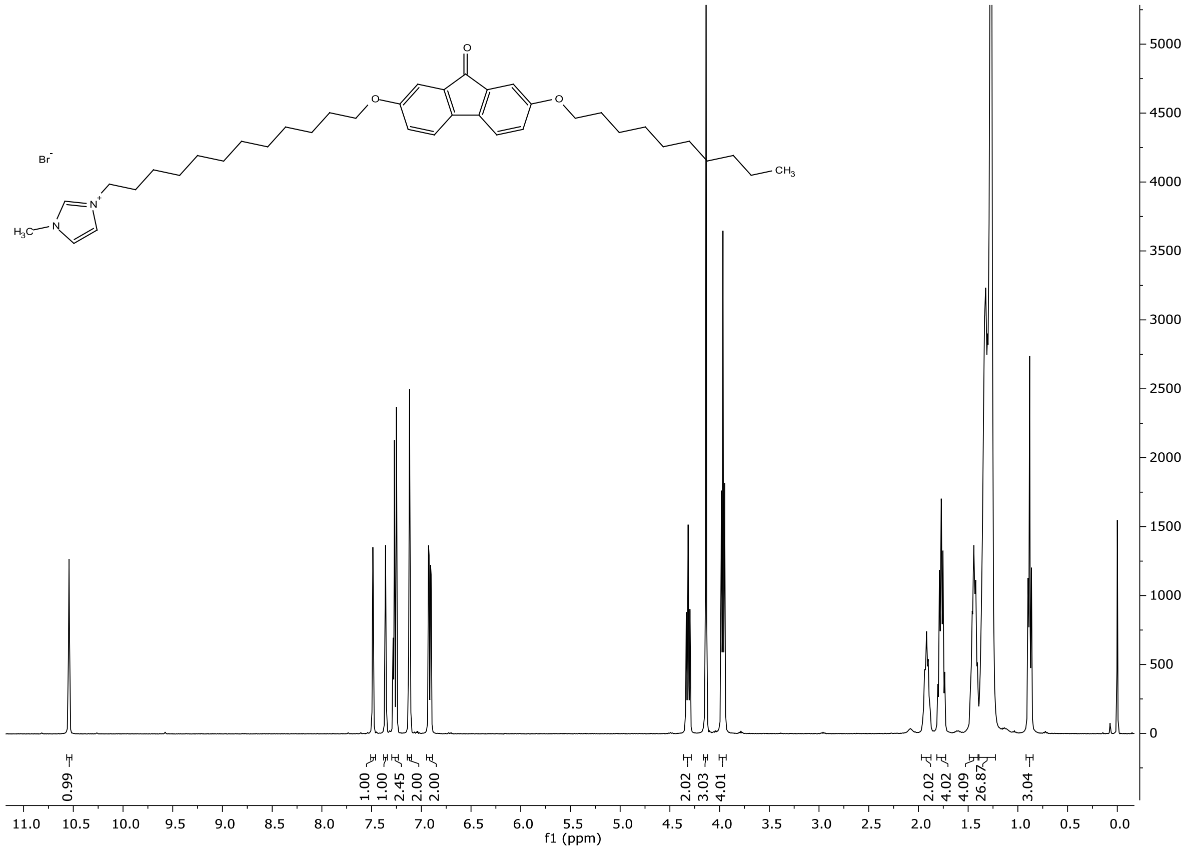
Br-
H3C
CH3
0.99
1.00
1.00
2.45
2.00
2.00
2.02
3.03
4.01
2.02
4.02
4.09
26.87
3.04
f1 (ppm)

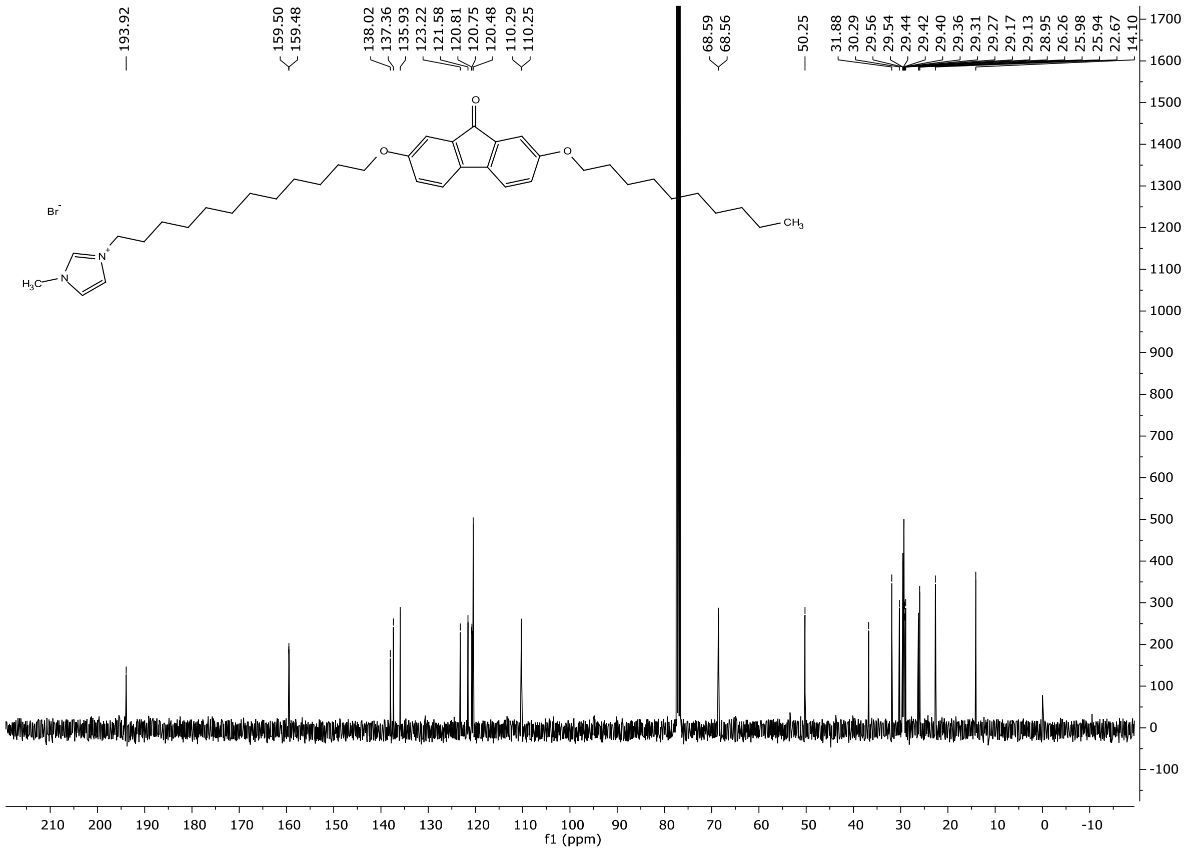
193.92
159.50
159.48
138.02
137.36
135.93
123.22
121.58
120.81
120.75
120.48
110.29
110.25
68.59
68.56
50.25
31.88
30.29
29.56
29.54
29.44
29.42
29.40
29.36
29.31
29.27
29.17
29.13
28.95
26.26
25.98
25.94
22.67
14.10
Br-
H3C
CH3
f1 (ppm)

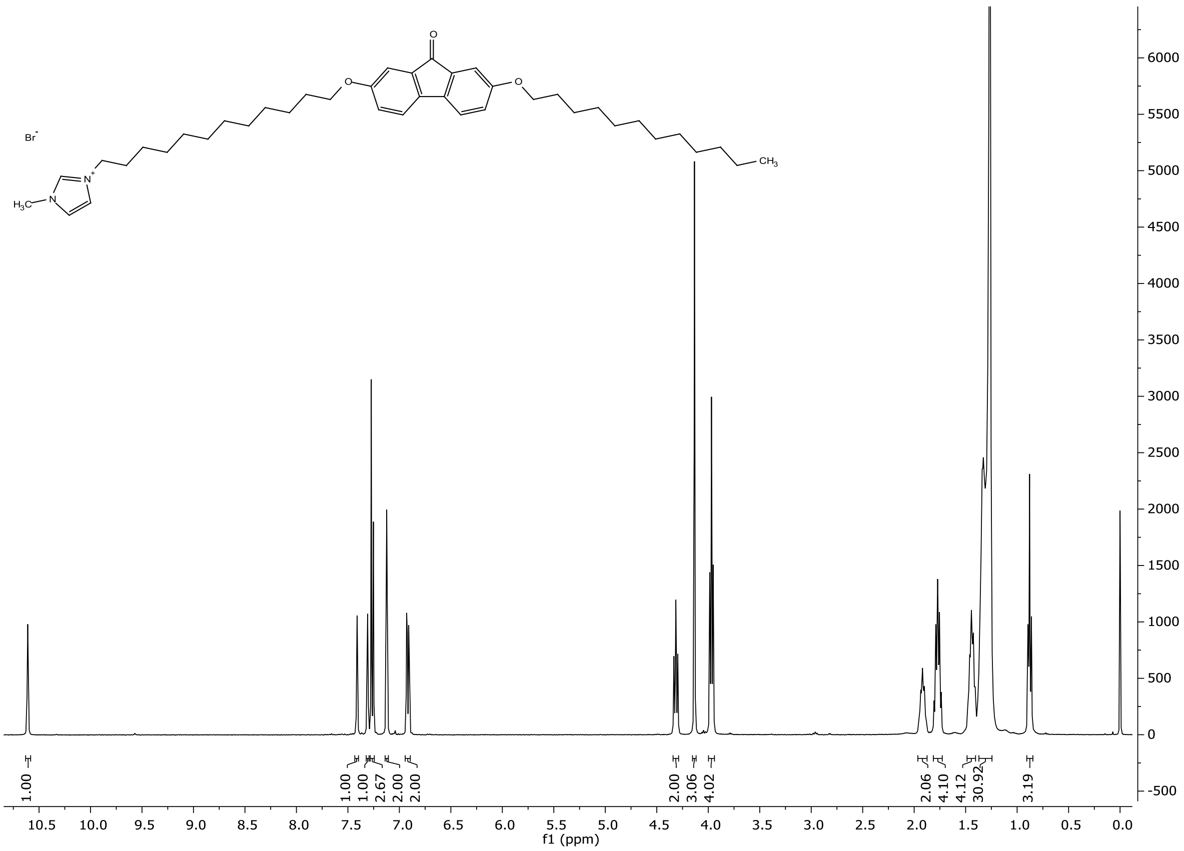

Br⁻
H3C
CH3
O
1.00
1.00
1.00
2.67
2.00
2.00
2.00
3.06
4.02
2.06
4.10
4.12
30.92
3.19
f1 (ppm)

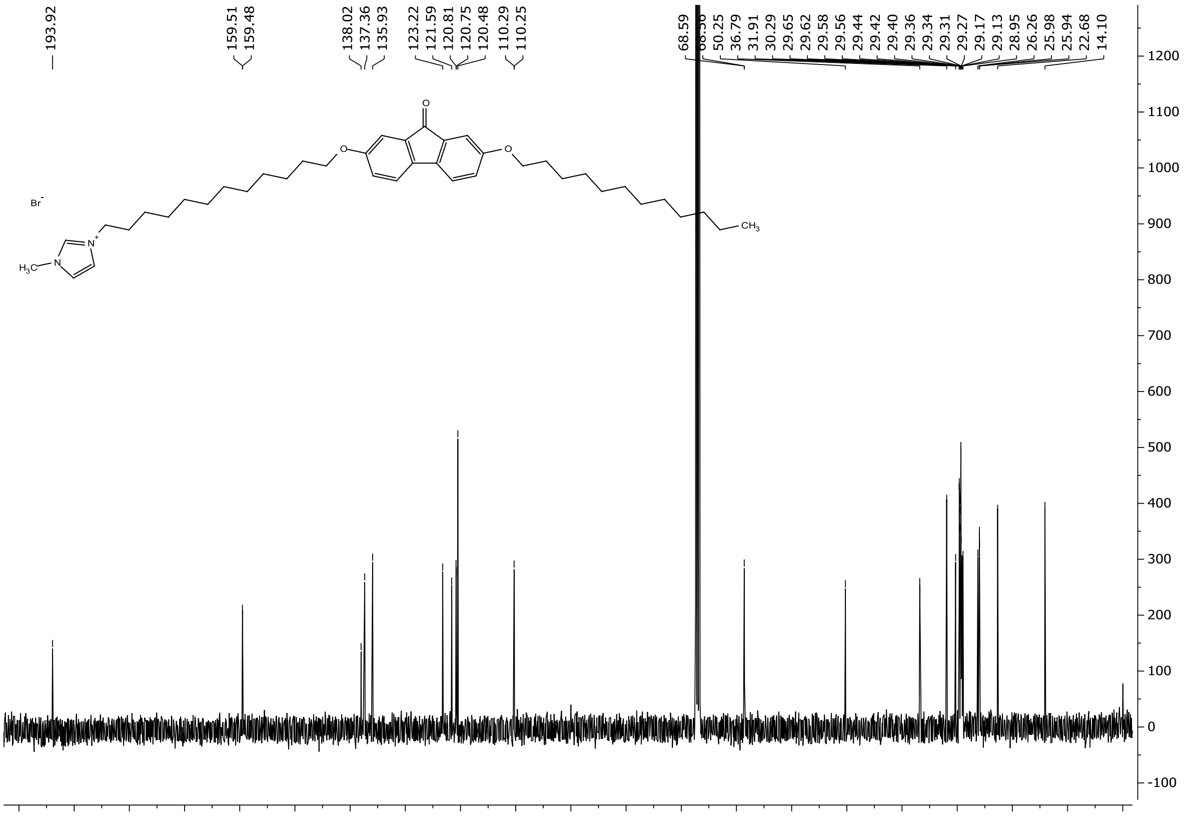

193.92
159.51
159.48
138.02
137.36
135.93
123.22
121.59
120.81
120.75
120.48
110.29
110.25
68.59
50.25
36.79
31.91
30.29
29.65
29.62
29.58
29.56
29.44
29.42
29.40
29.36
29.34
29.31
29.27
29.17
29.13
28.95
26.26
25.98
25.94
22.68
14.10
Br⁻
H3C
CH3
O
f1 (ppm)

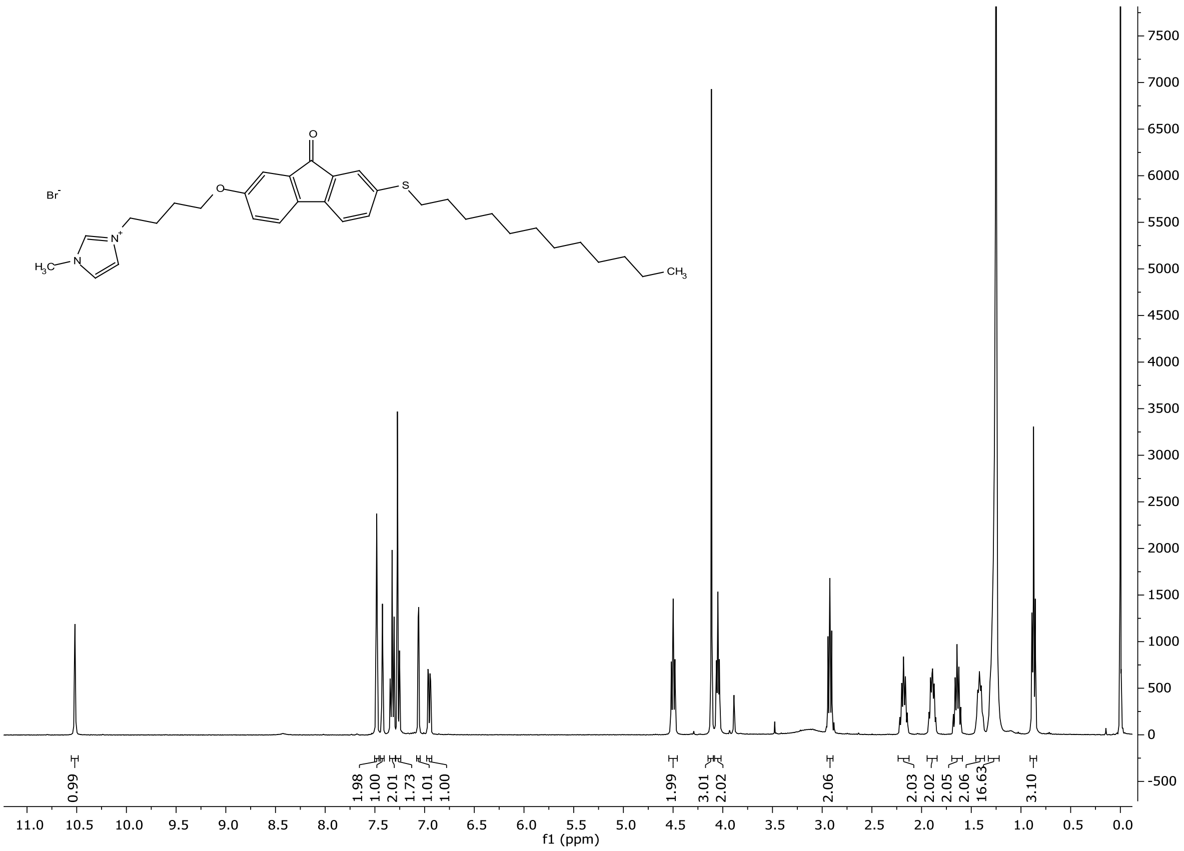

Br⁻
O
S
CH3
H3C
N
N+
0.99
1.98
1.00
2.01
1.73
1.01
1.00
1.99
3.01
2.02
2.06
2.03
2.02
2.05
2.06
16.63
3.10
f1 (ppm)

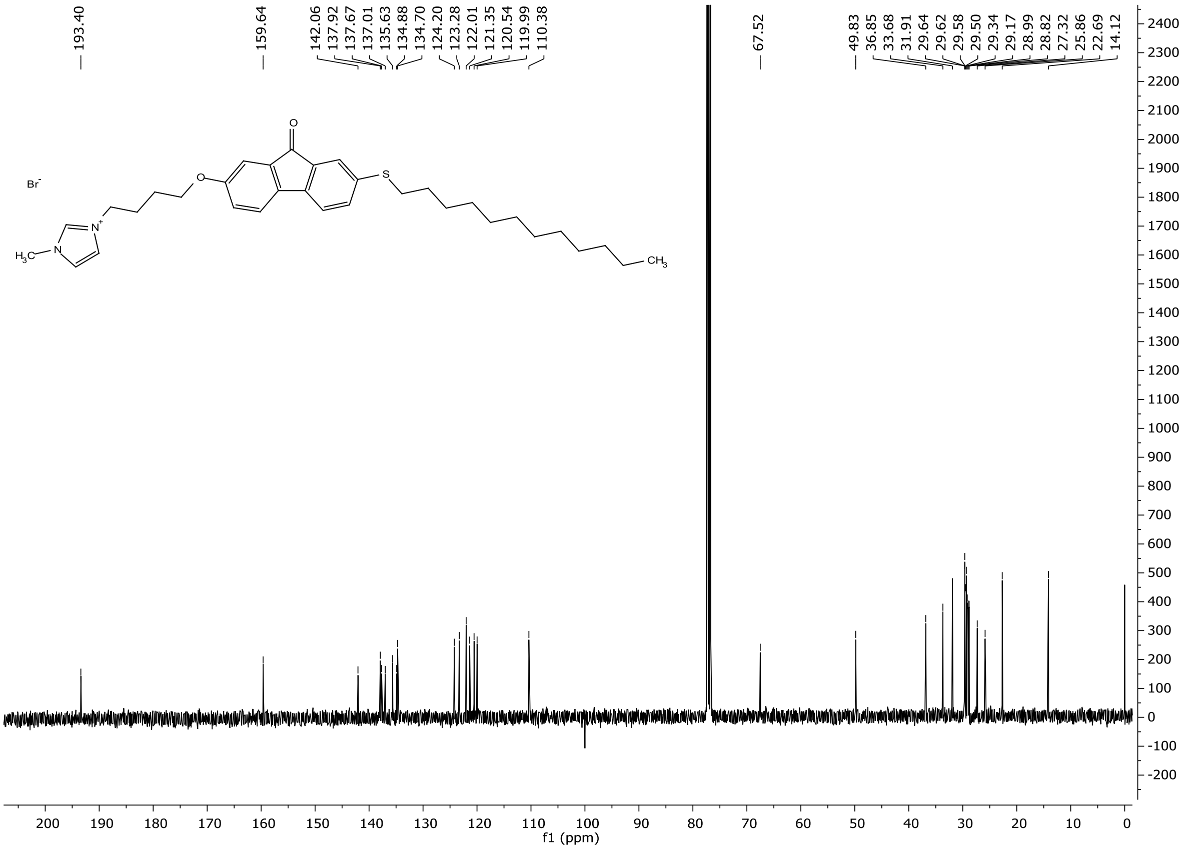

193.40
159.64
142.06
137.92
137.67
137.01
135.63
134.88
134.70
124.20
123.28
122.01
121.35
120.54
119.99
110.38
67.52
49.83
36.85
33.68
31.91
29.64
29.62
29.58
29.50
29.34
29.17
28.99
28.82
27.32
25.86
22.69
14.12
f1 (ppm)

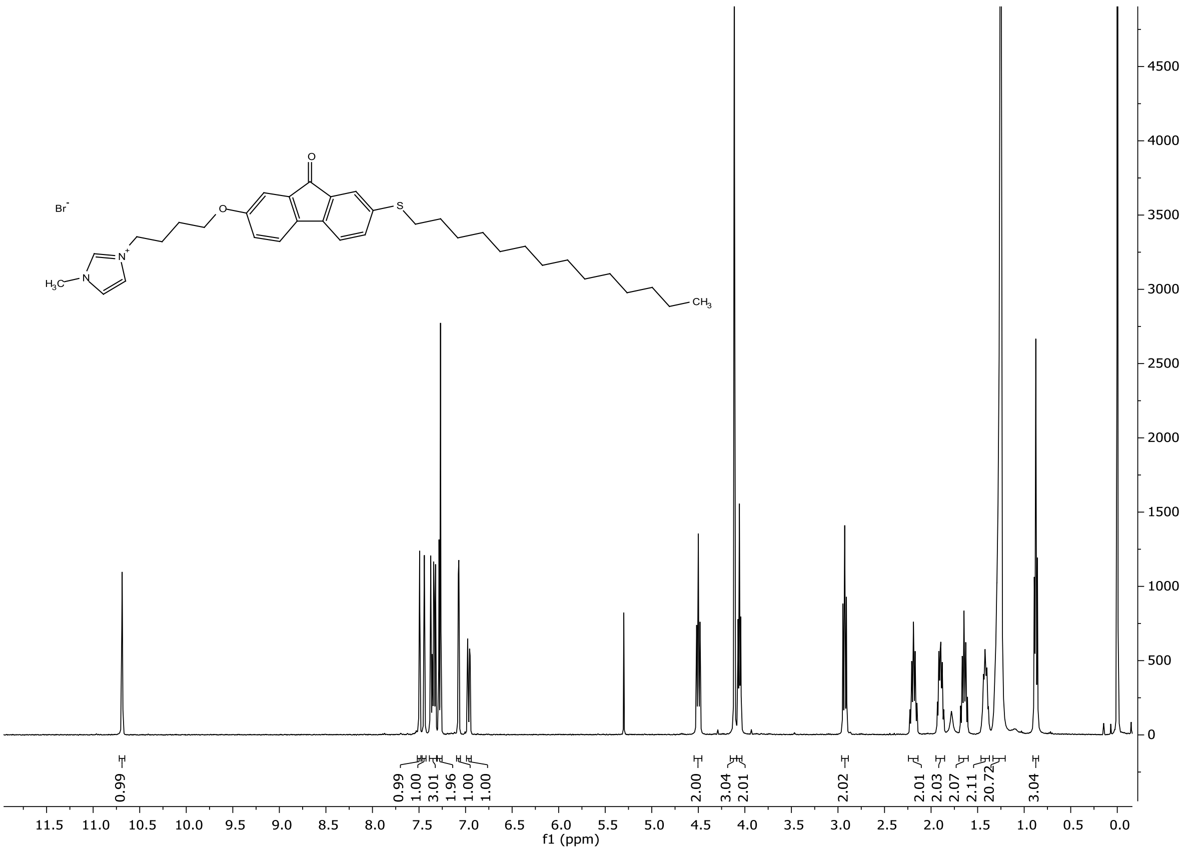
Br⁻
H3C
N
N+
O
O
S
CH3
0.99
0.99
1.00
3.01
1.96
1.00
1.00
2.00
3.04
2.01
2.02
2.01
2.03
2.07
2.11
20.72
3.04
f1 (ppm)

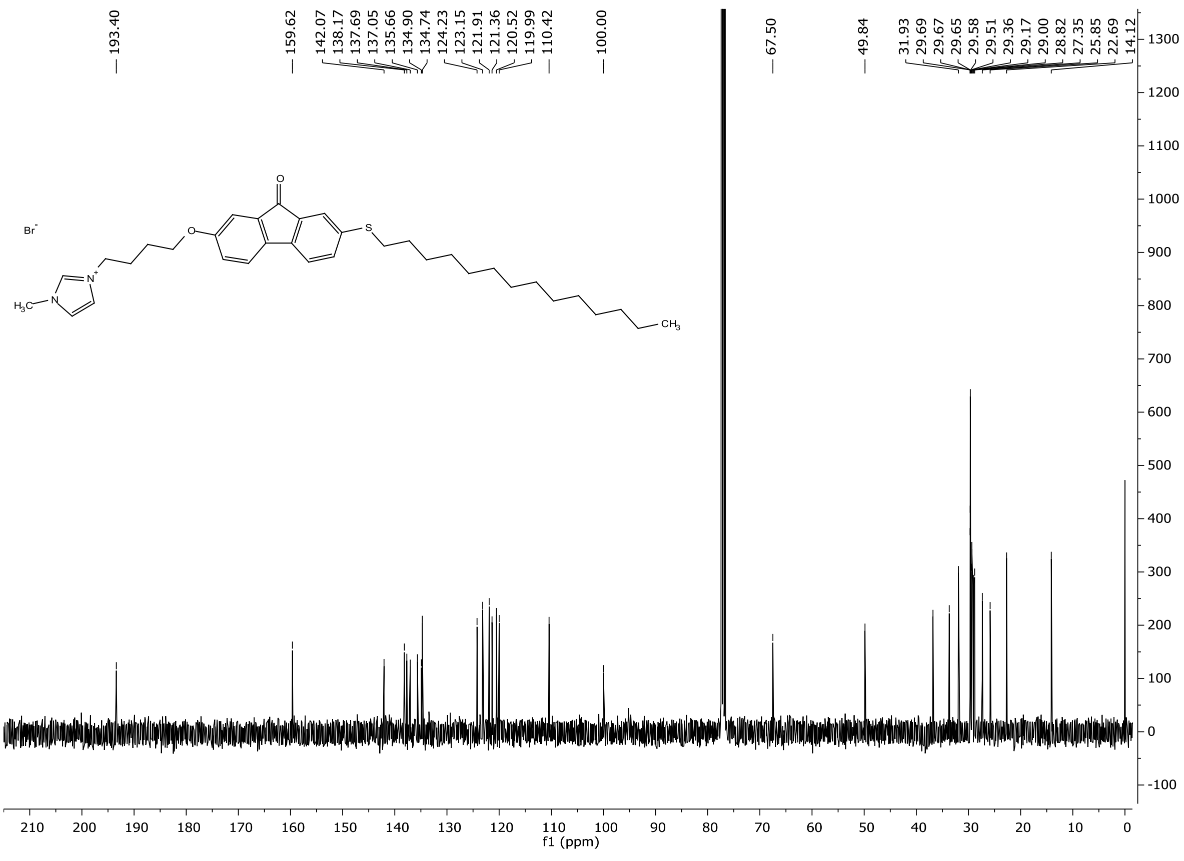
193.40
159.62
142.07
138.17
137.69
137.05
135.66
134.90
134.74
124.23
123.15
121.91
121.36
120.52
119.99
110.42
100.00
67.50
49.84
31.93
29.69
29.67
29.65
29.58
29.51
29.36
29.17
29.00
28.82
27.35
25.85
22.69
14.12
Br⁻
H3C
N
N+
O
O
S
CH3
f1 (ppm)

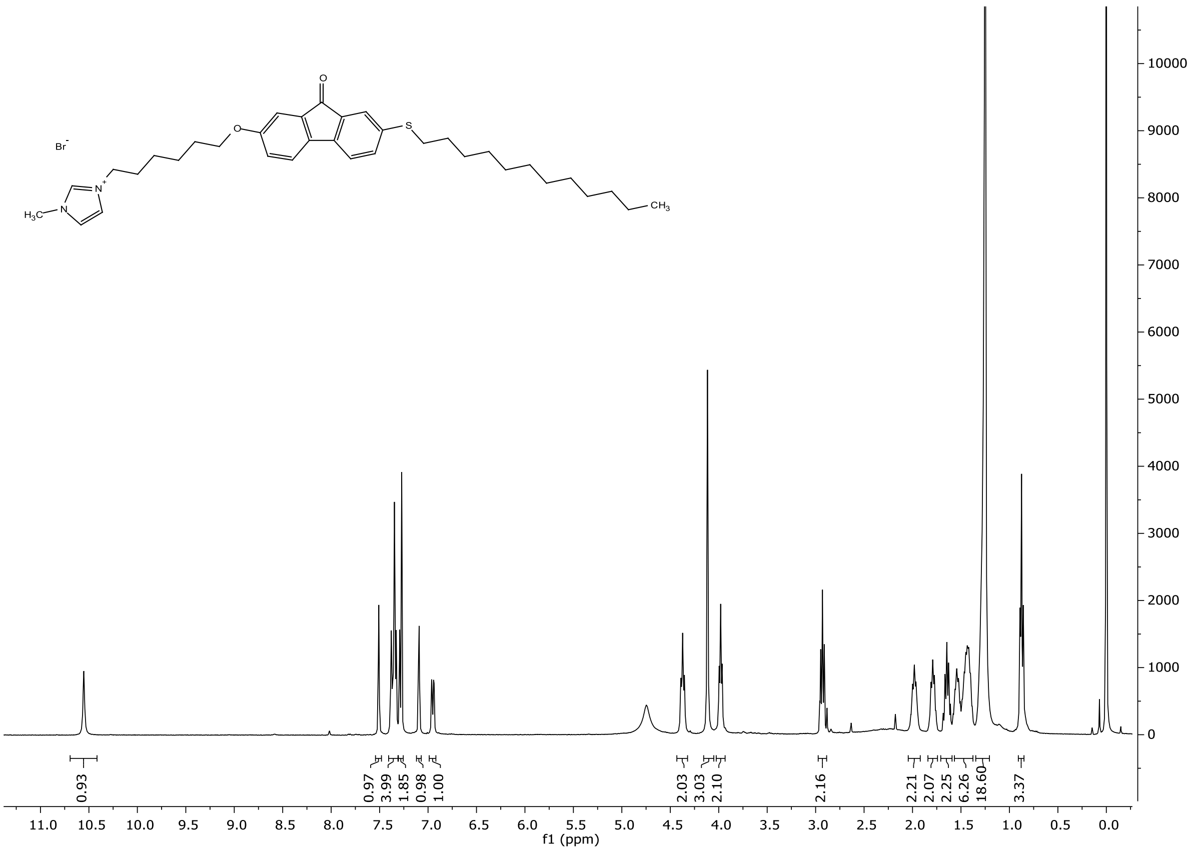
Br-
H3C
O
S
CH3
0.93
0.97
3.99
1.85
0.98
1.00
2.03
3.03
2.10
2.16
2.21
2.07
2.25
6.26
18.60
3.37
f1 (ppm)

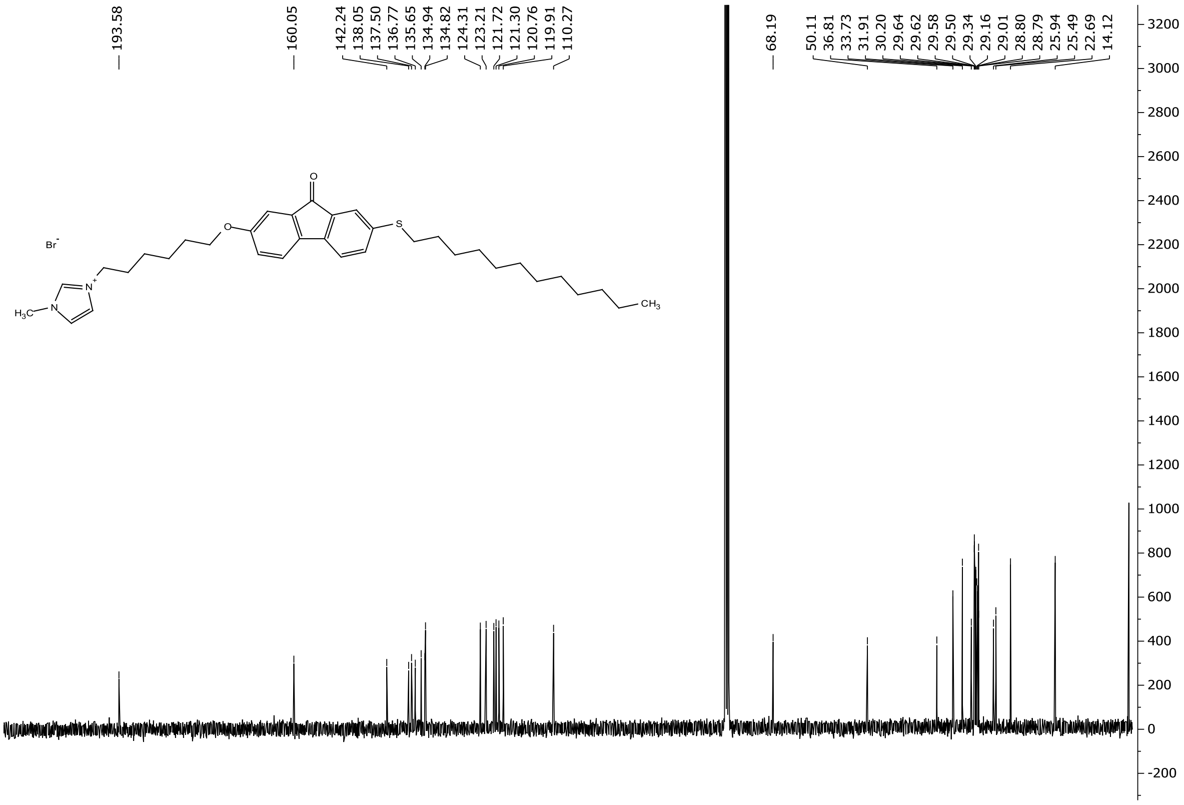
193.58
160.05
142.24
138.05
137.50
136.77
135.65
134.94
134.82
124.31
123.21
121.72
121.30
120.76
119.91
110.27
68.19
50.11
36.81
33.73
31.91
30.20
29.64
29.62
29.58
29.50
29.34
29.16
29.01
28.80
28.79
25.94
25.49
22.69
14.12
Br-
H3C
O
S
CH3
f1 (ppm)

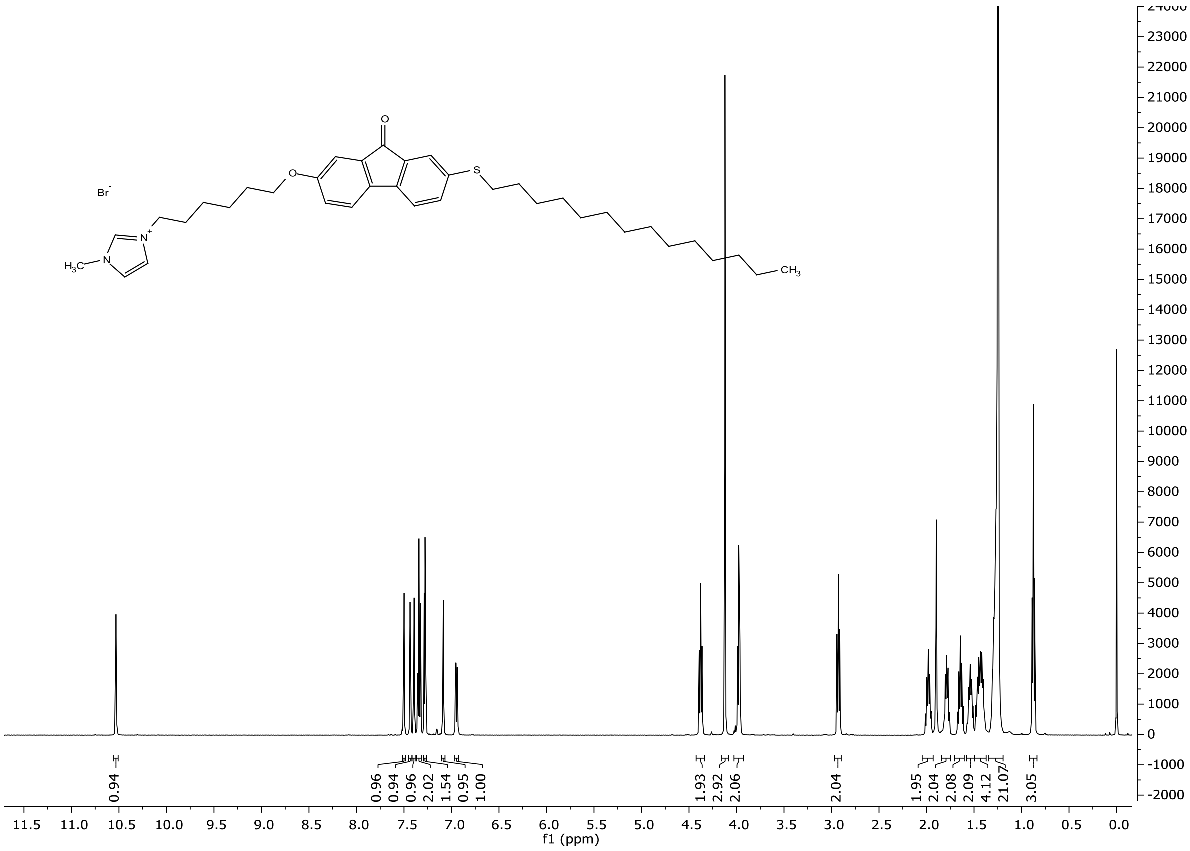

Br-
H3C
O
S
CH3
0.94
0.96
0.94
0.96
2.02
1.54
0.95
1.00
1.93
2.92
2.06
2.04
1.95
2.04
2.08
2.09
4.12
21.07
3.05
f1 (ppm)

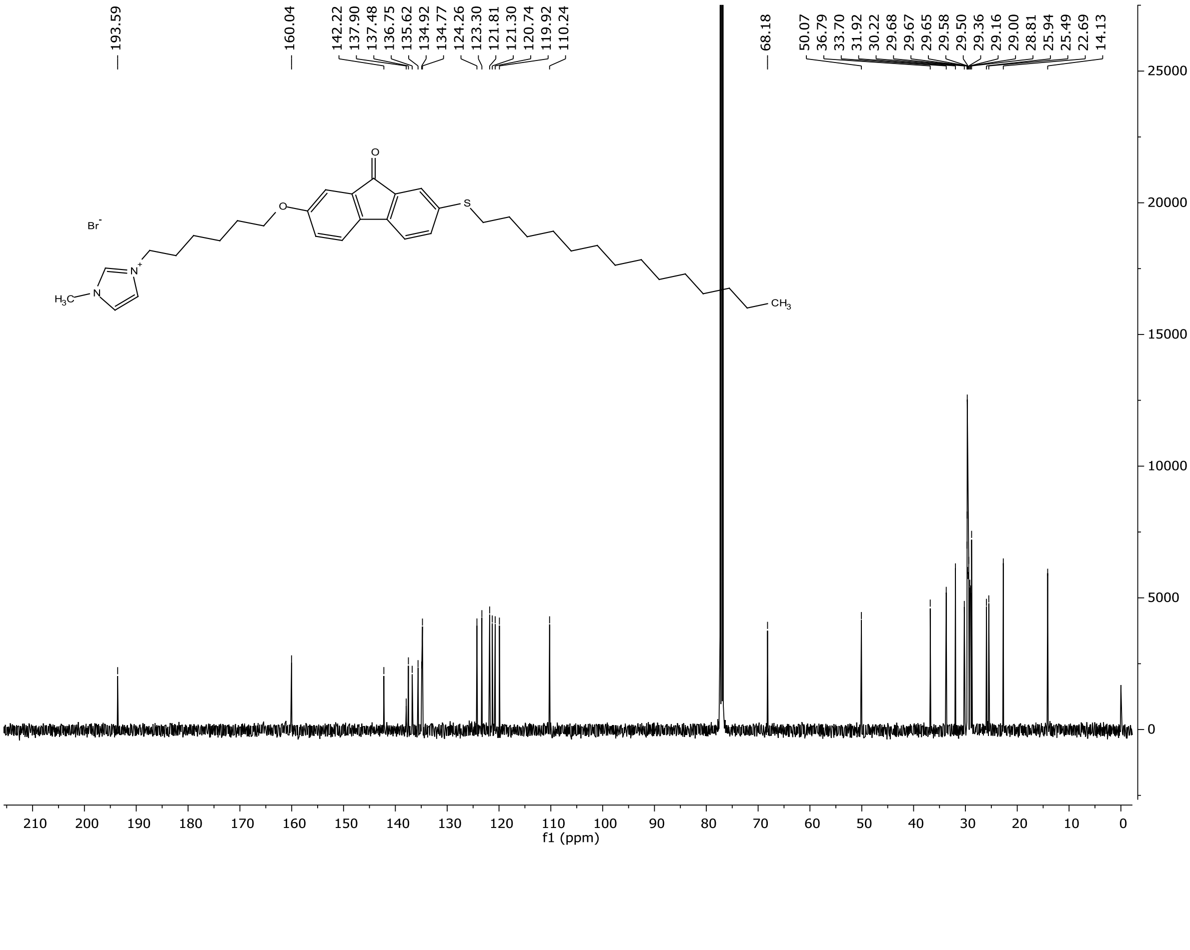

193.59
160.04
142.22
137.90
137.48
136.75
135.62
134.92
134.77
124.26
123.30
121.81
121.30
120.74
119.92
110.24
68.18
50.07
36.79
33.70
31.92
30.22
29.68
29.67
29.65
29.58
29.50
29.36
29.16
29.00
28.81
25.94
25.49
22.69
14.13
Br-
H3C
O
S
CH3
f1 (ppm)

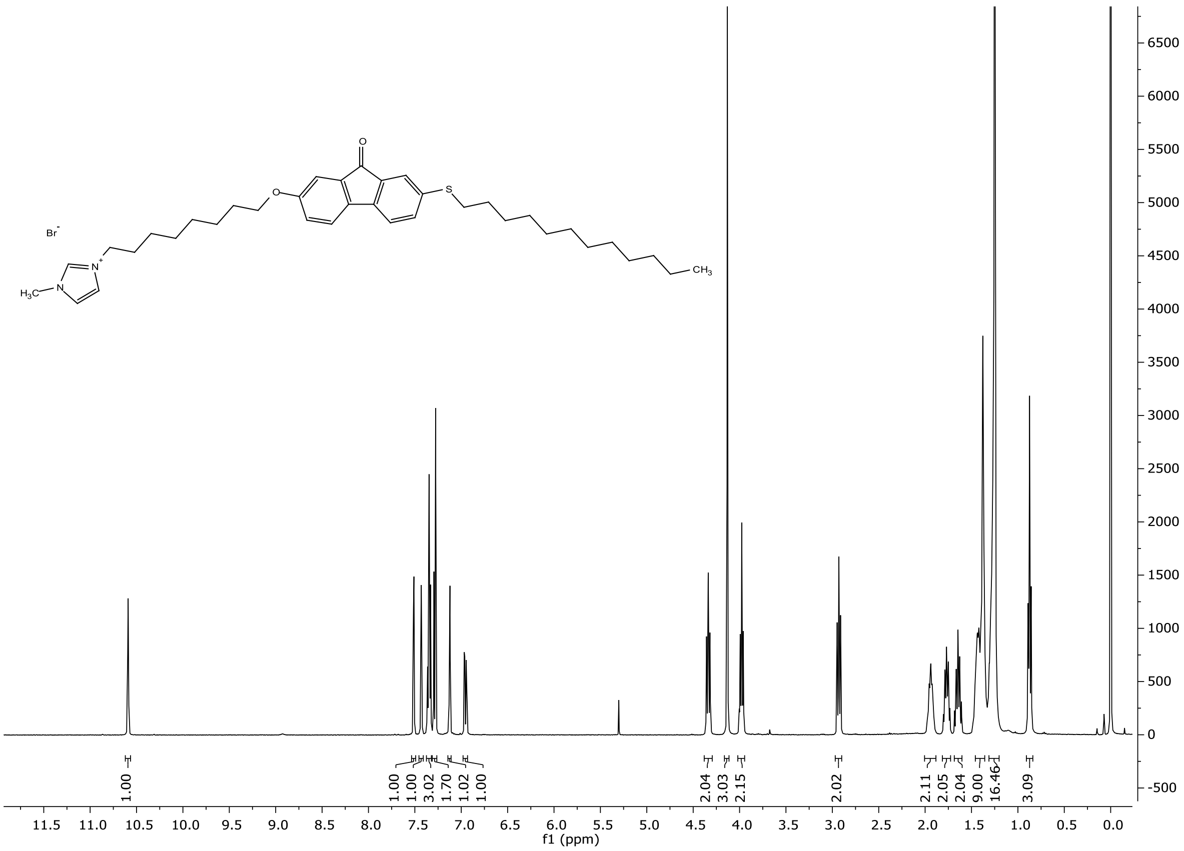

Br⁻
H3C
CH3
S
O
1.00
1.00
1.00
3.02
1.70
1.02
1.00
2.04
3.03
2.15
2.02
2.11
2.05
2.04
9.00
16.46
3.09
f1 (ppm)

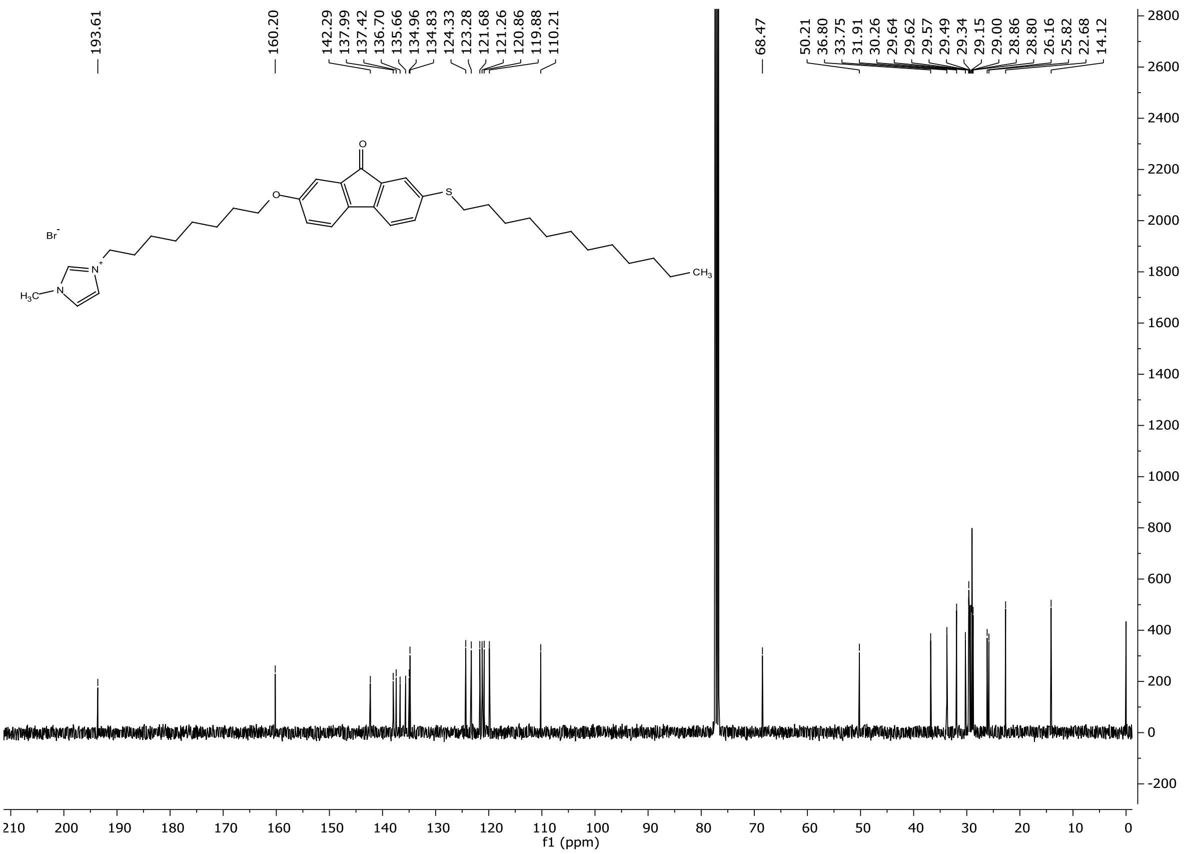

193.61
160.20
142.29
137.99
137.42
136.70
135.66
134.96
134.83
124.33
123.28
121.68
121.26
120.86
119.88
110.21
68.47
50.21
36.80
33.75
31.91
30.26
29.64
29.62
29.57
29.49
29.34
29.15
29.00
28.86
28.80
26.16
25.82
22.68
14.12
f1 (ppm)

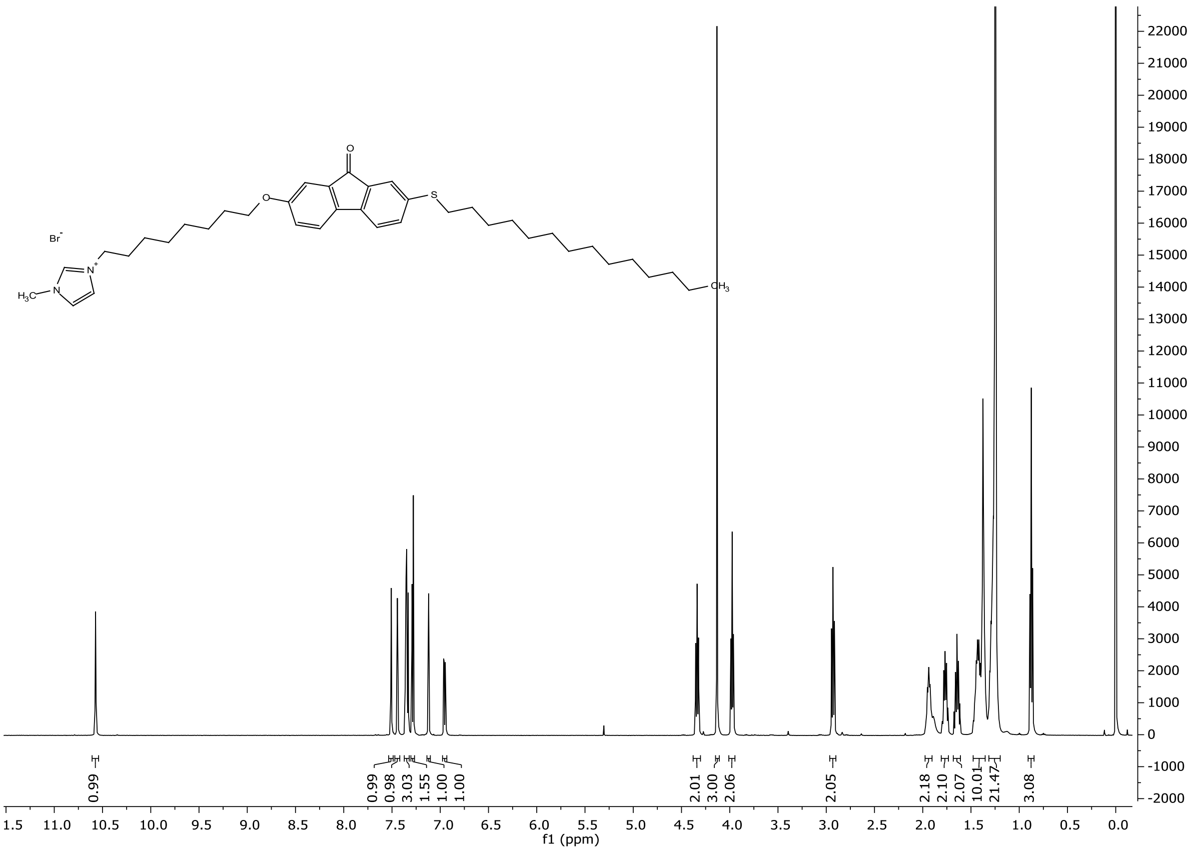

Br-
H3C
O
S
CH3
0.99
0.99
0.98
3.03
1.55
1.00
1.00
2.01
3.00
2.06
2.05
2.18
2.10
2.07
10.01
21.47
3.08
f1 (ppm)

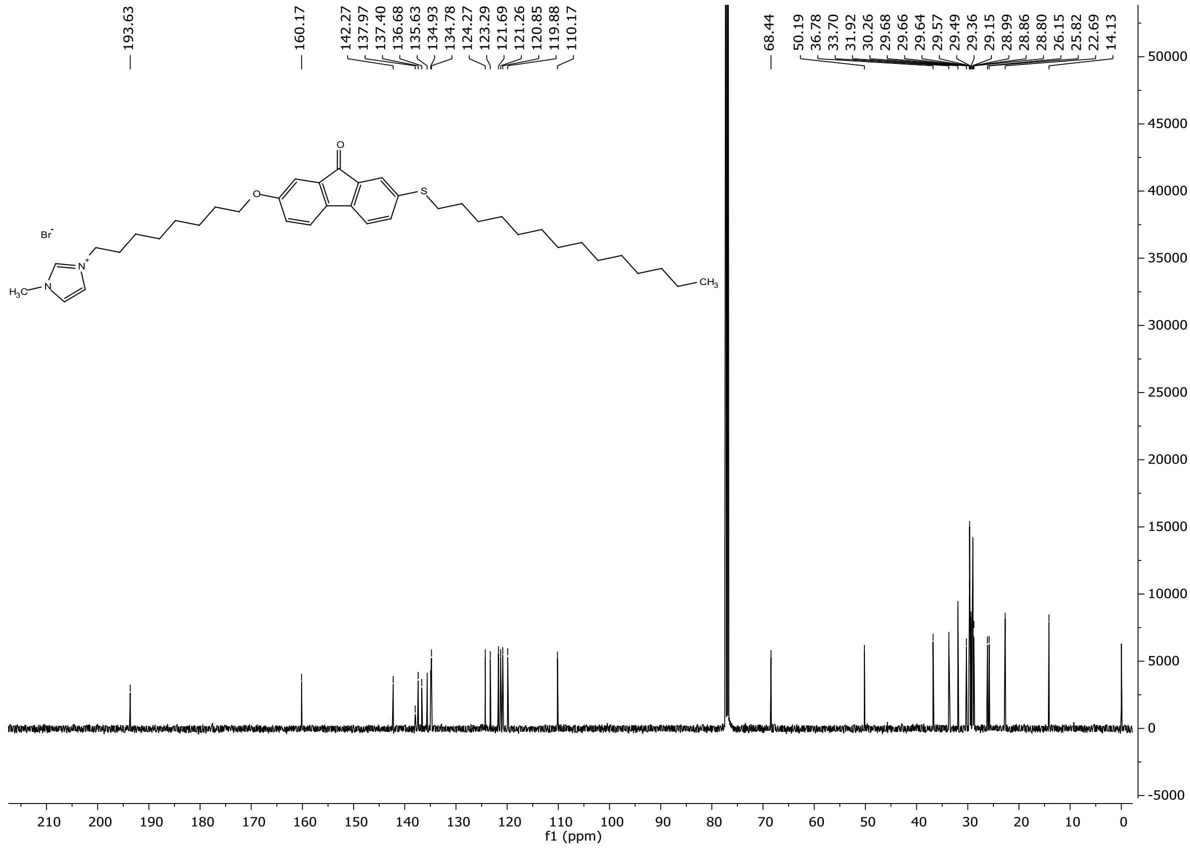

193.63
160.17
142.27
137.97
137.40
136.68
135.63
134.93
134.78
124.27
123.29
121.69
121.26
120.85
119.88
110.17
68.44
50.19
36.78
33.70
31.92
30.26
29.68
29.66
29.64
29.57
29.49
29.36
29.15
28.99
28.86
28.80
26.15
25.82
22.69
14.13
Br-
H3C
O
S
CH3
f1 (ppm)

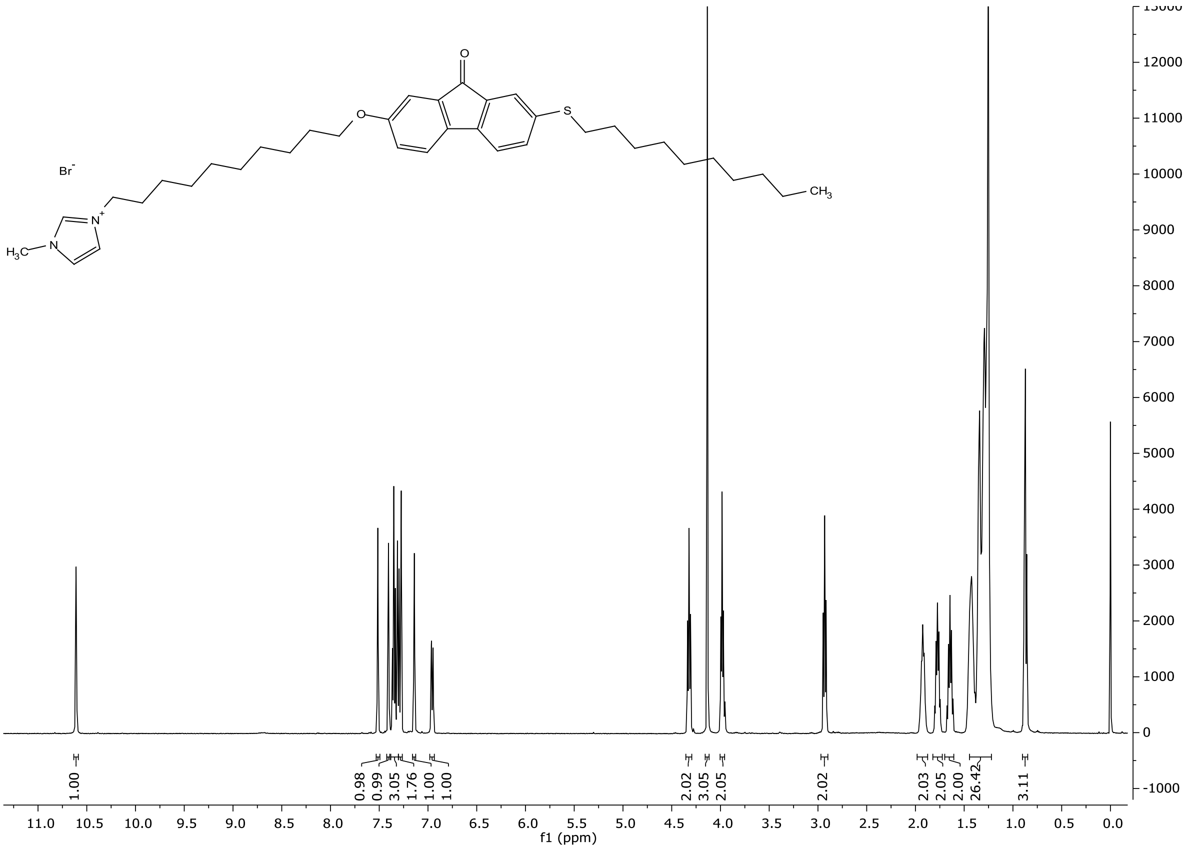

O
O
S
Br-
N+
N
H3C
CH3
1.00
0.98
0.99
3.05
1.76
1.00
1.00
2.02
3.05
2.05
2.02
2.03
2.05
2.00
26.42
3.11
f1 (ppm)

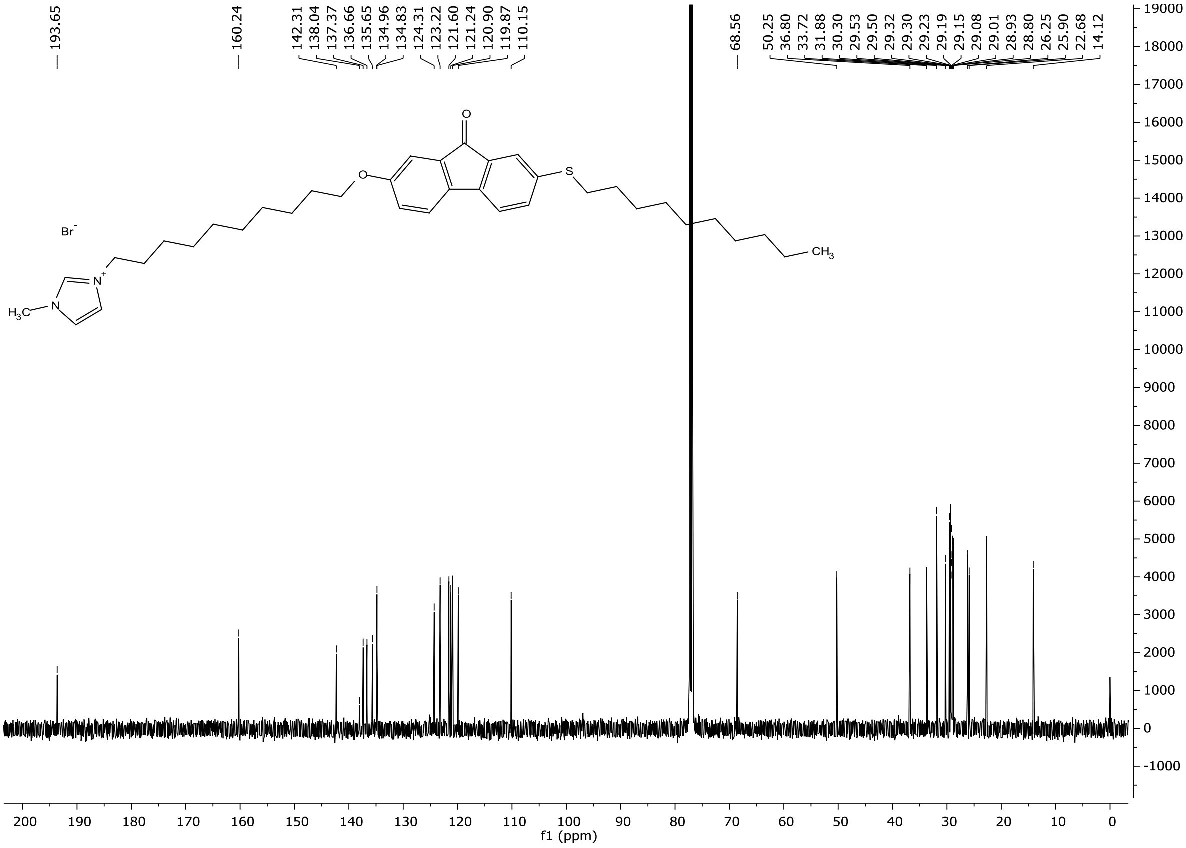

193.65
160.24
142.31
138.04
137.37
136.66
135.65
134.96
134.83
124.31
123.22
121.60
121.24
120.90
119.87
110.15
68.56
50.25
36.80
33.72
31.88
30.30
29.53
29.50
29.32
29.30
29.23
29.19
29.15
29.08
29.01
28.93
28.80
26.25
25.90
22.68
14.12
O
O
S
Br-
N+
N
H3C
CH3
f1 (ppm)

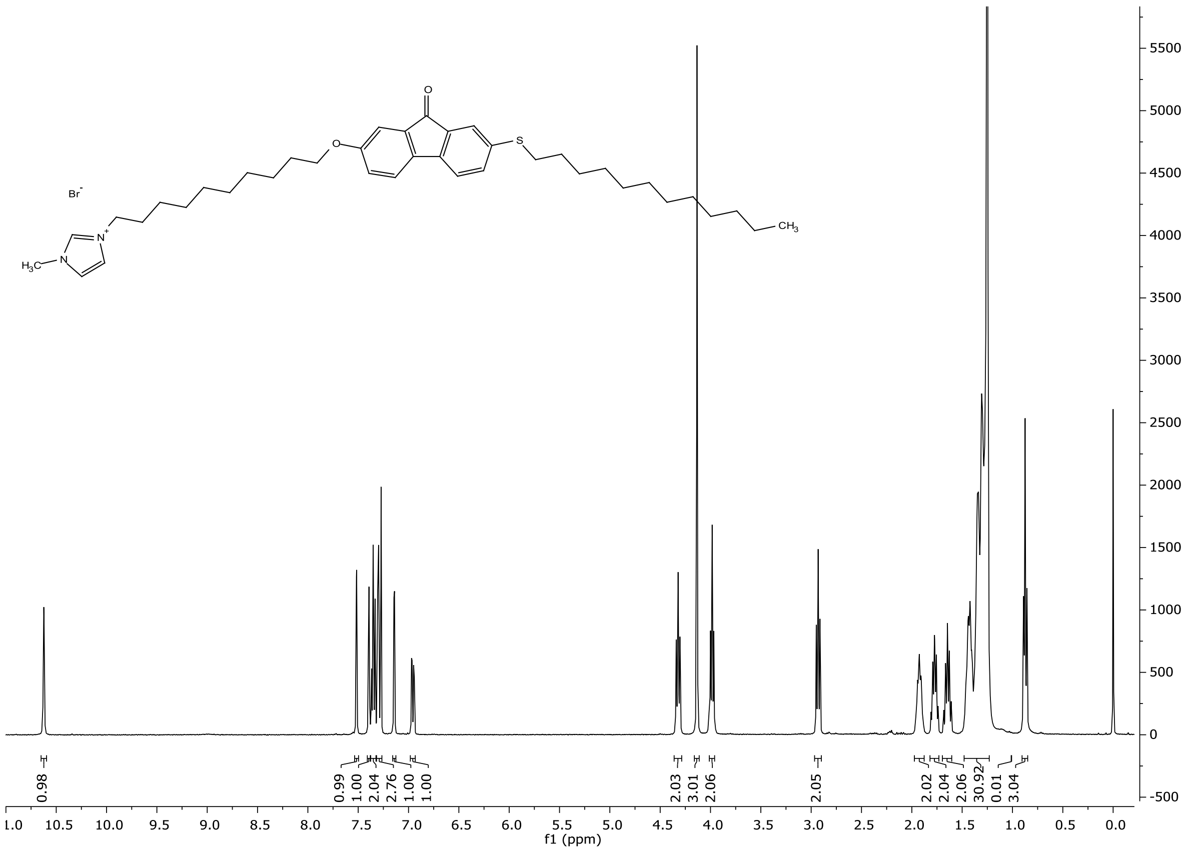

O
O
S
Br-
N+
N
H3C
CH3
0.98
0.99
1.00
2.04
2.76
1.00
1.00
2.03
3.01
2.06
2.05
2.02
2.04
2.06
30.92
0.01
3.04
f1 (ppm)

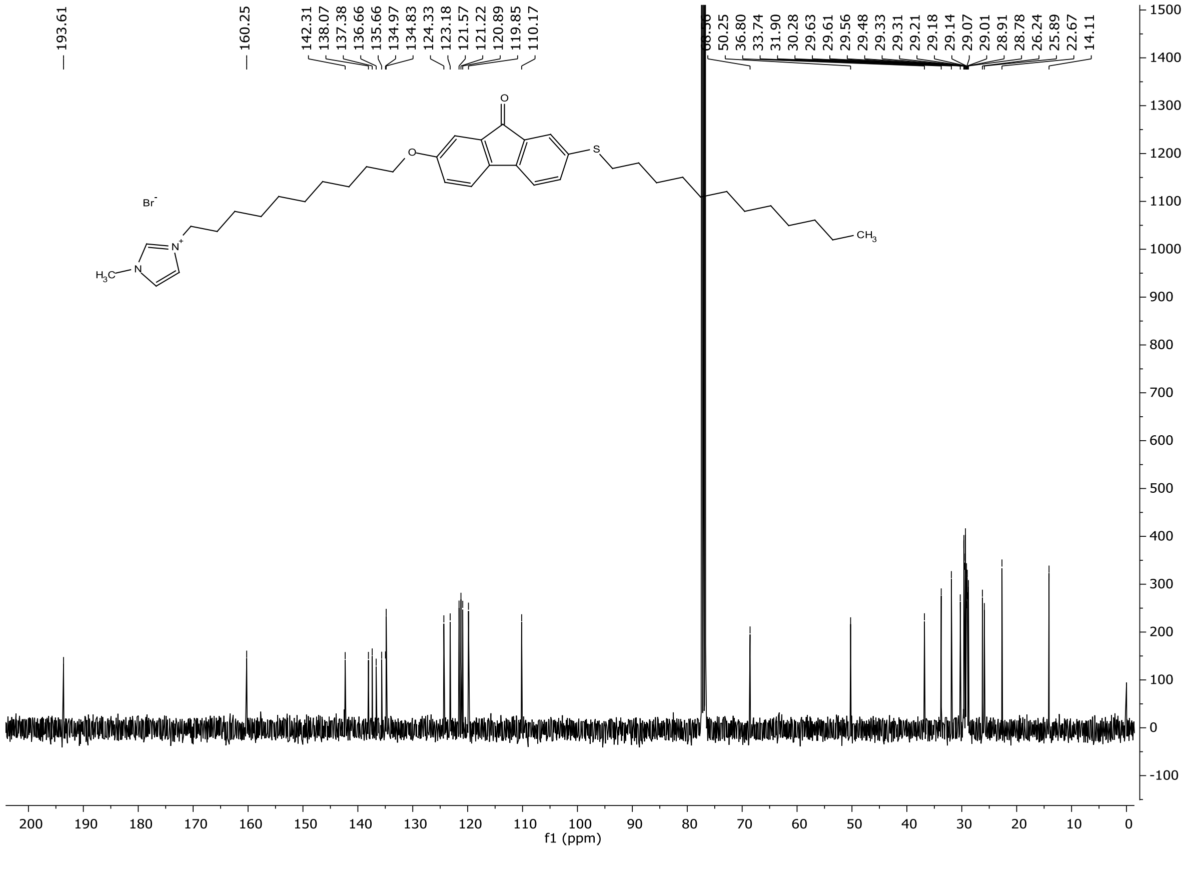

193.61
160.25
142.31
138.07
137.38
136.66
135.66
134.97
134.83
124.33
123.18
121.57
121.22
120.89
119.85
110.17
50.25
36.80
33.74
31.90
30.28
29.63
29.61
29.56
29.48
29.33
29.31
29.21
29.18
29.14
29.07
29.01
28.91
28.78
26.24
25.89
22.67
14.11
O
O
S
Br-
N+
N
H3C
CH3
f1 (ppm)

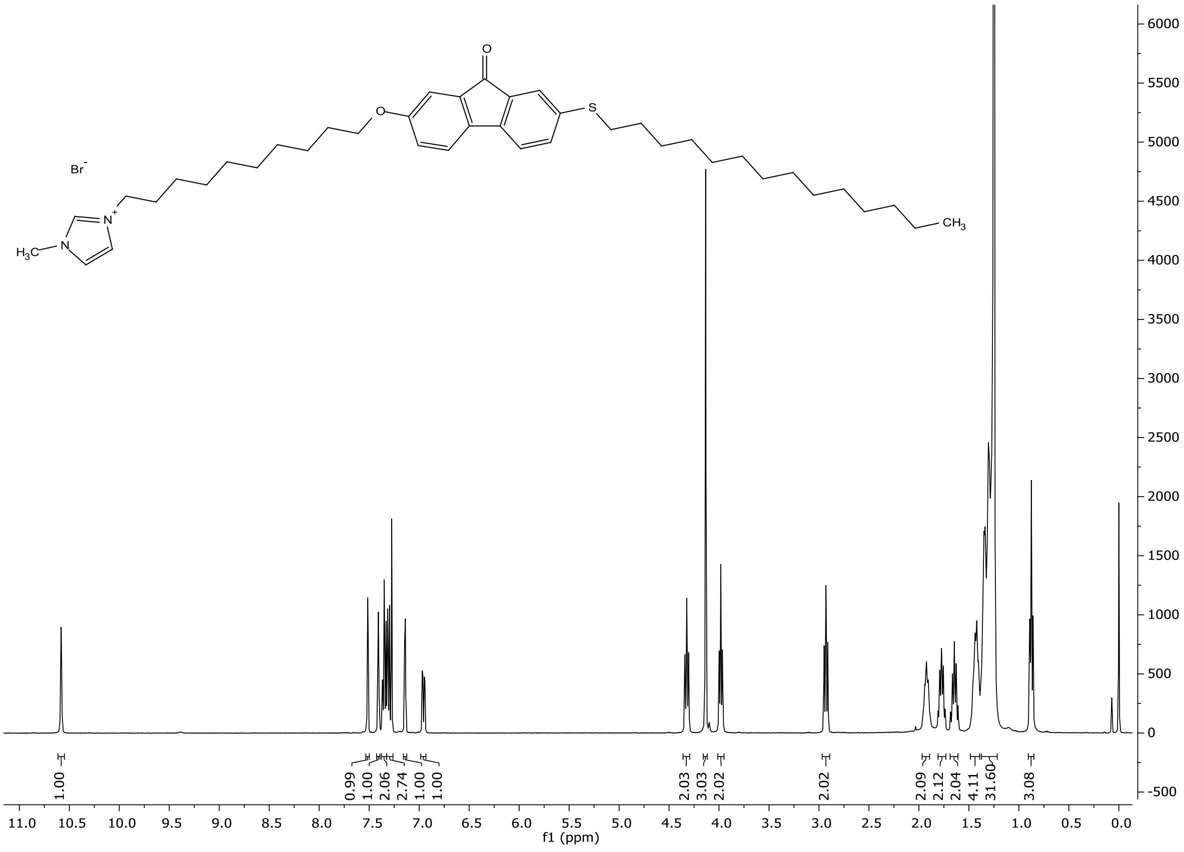

Br⁻
O
S
CH3
H3C
N
N⁺
1.00
0.99
1.00
2.06
2.74
1.00
1.00
2.03
3.03
2.02
2.02
2.09
2.12
2.04
4.11
31.60
3.08
f1 (ppm)

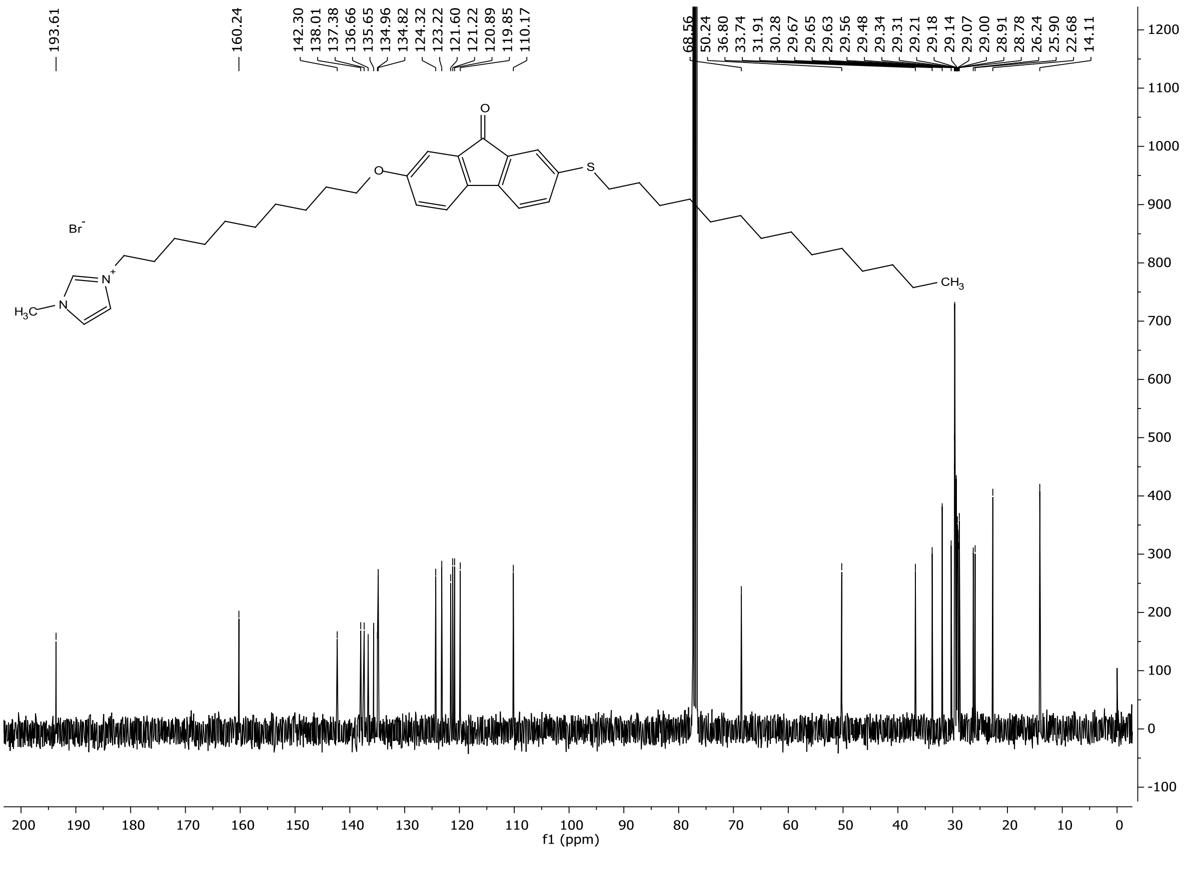

193.61
160.24
142.30
138.01
137.38
136.66
135.65
134.96
134.82
124.32
123.22
121.60
121.22
120.89
119.85
110.17
68.56
50.24
36.80
33.74
31.91
30.28
29.67
29.65
29.63
29.56
29.48
29.34
29.31
29.21
29.18
29.14
29.07
29.00
28.91
28.78
26.24
25.90
22.68
14.11
Br⁻
O
S
CH3
H3C
N
N⁺
f1 (ppm)

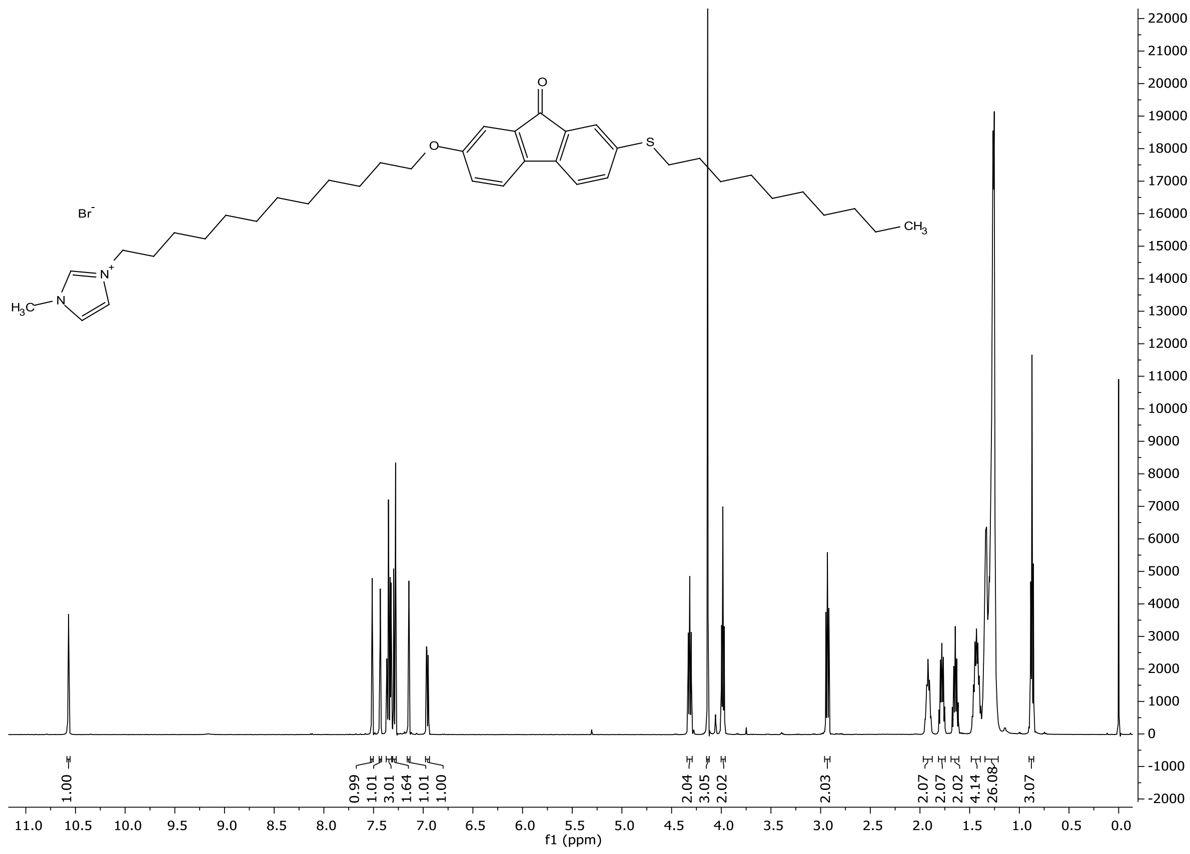
O
S
Br-
N+
N
H3C
CH3
1.00
0.99
1.01
3.01
1.64
1.01
1.00
2.04
3.05
2.02
2.03
2.07
2.07
2.02
4.14
26.08
3.07
f1 (ppm)

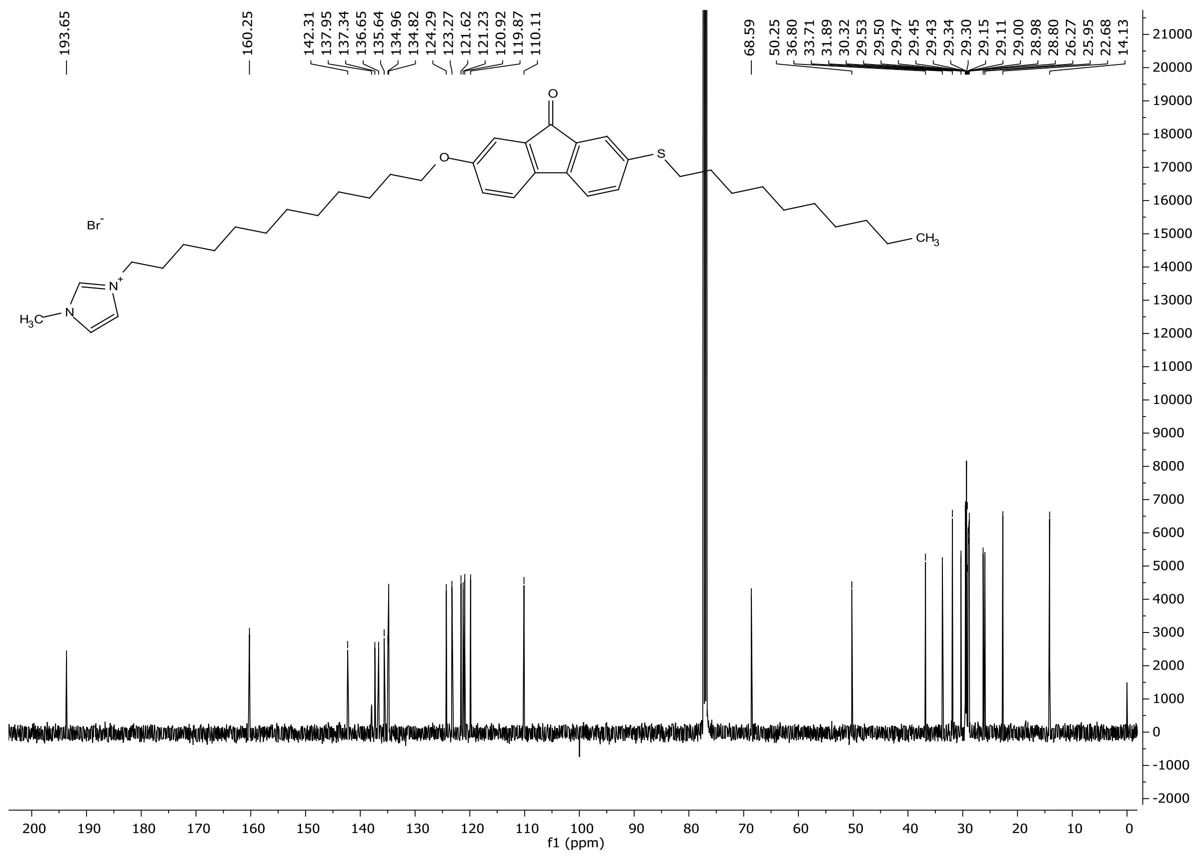
193.65
160.25
142.31
137.95
137.34
136.65
135.64
134.96
134.82
124.29
123.27
121.62
121.23
120.92
119.87
110.11
68.59
50.25
36.80
33.71
31.89
30.32
29.53
29.50
29.47
29.45
29.43
29.34
29.30
29.15
29.11
29.00
28.98
28.80
26.27
25.95
22.68
14.13
O
S
Br-
N+
N
H3C
CH3
f1 (ppm)

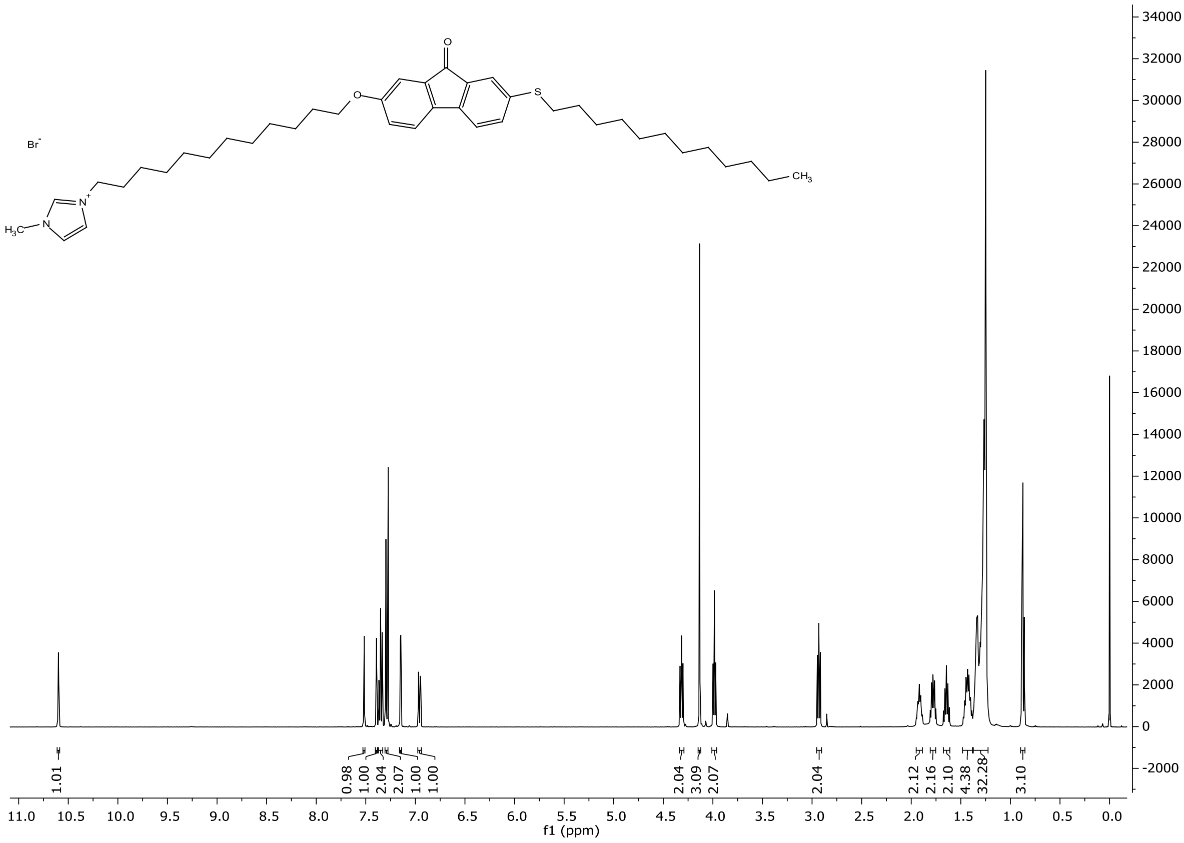
Br-
H3C
N
N+
O
S
CH3
1.01
0.98
1.00
2.04
2.07
1.00
1.00
2.04
3.09
2.07
2.04
2.12
2.16
2.10
4.38
32.28
3.10
f1 (ppm)

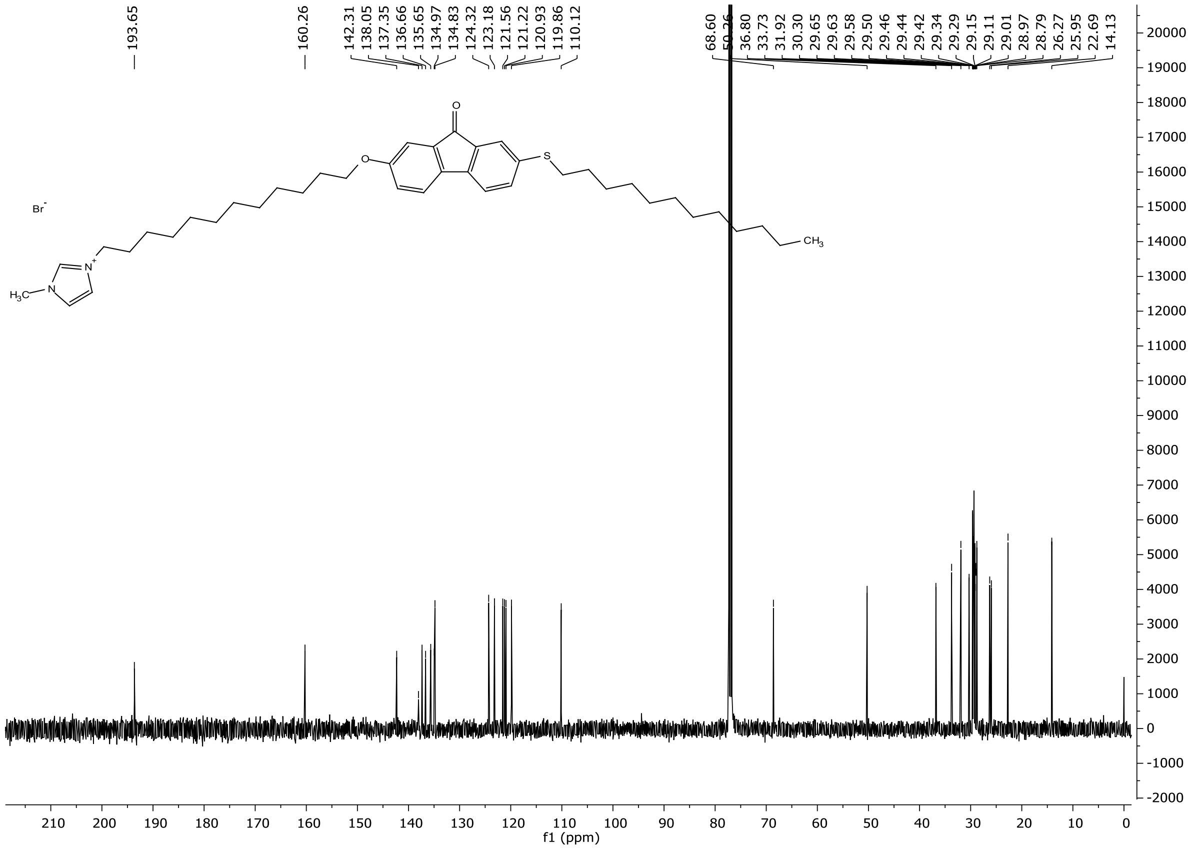
193.65
160.26
142.31
138.05
137.35
136.66
135.65
134.97
134.83
124.32
123.18
121.56
121.22
120.93
119.86
110.12
68.60
36.80
33.73
31.92
30.30
29.65
29.63
29.58
29.50
29.46
29.44
29.42
29.34
29.29
29.15
29.11
29.01
28.97
28.79
26.27
25.95
22.69
14.13
Br-
H3C
N
N+
O
S
CH3
f1 (ppm)

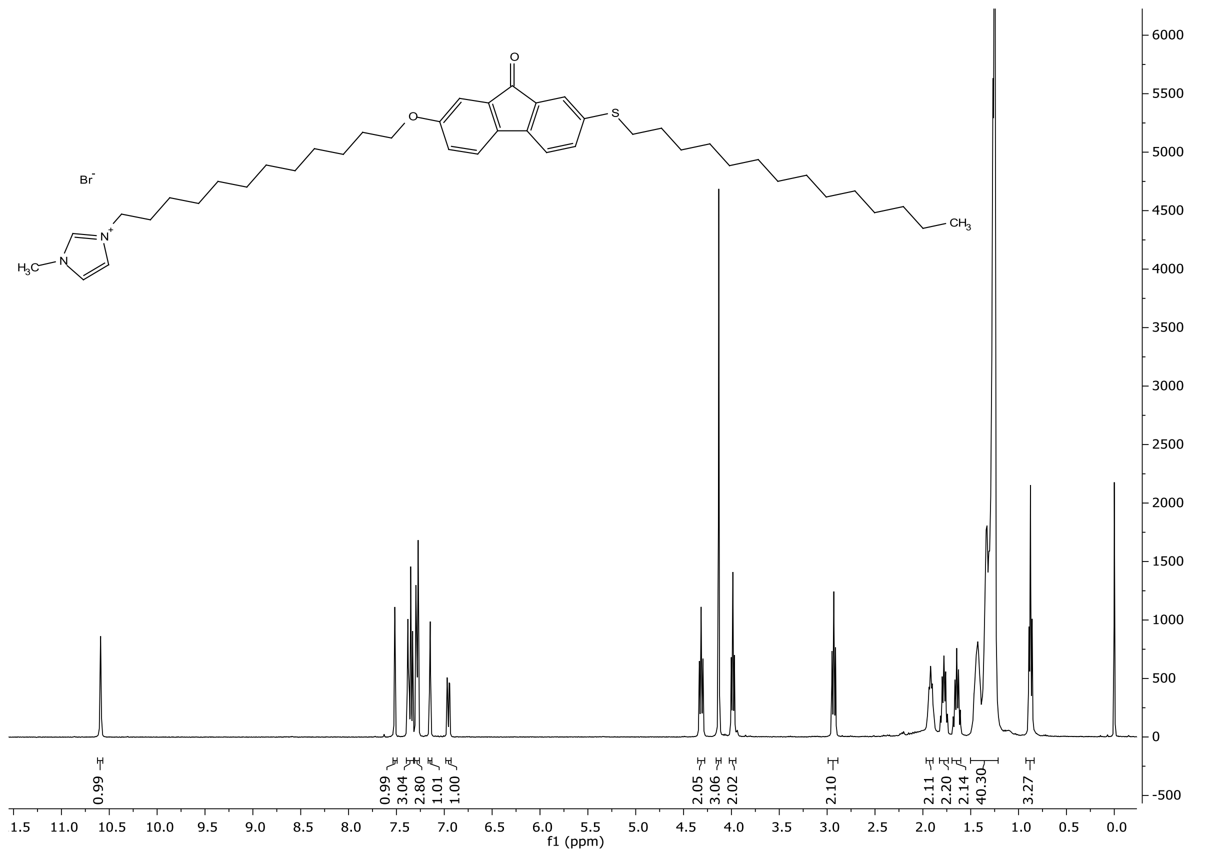

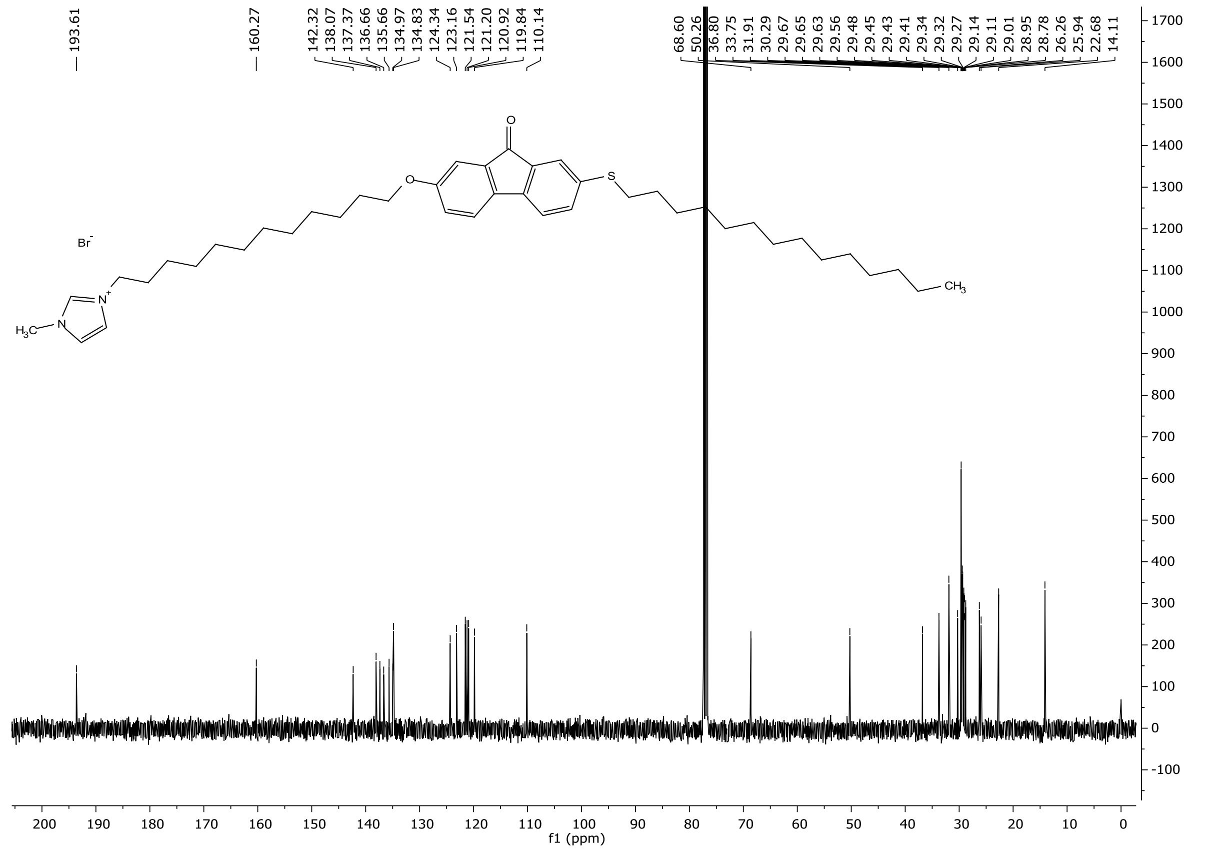

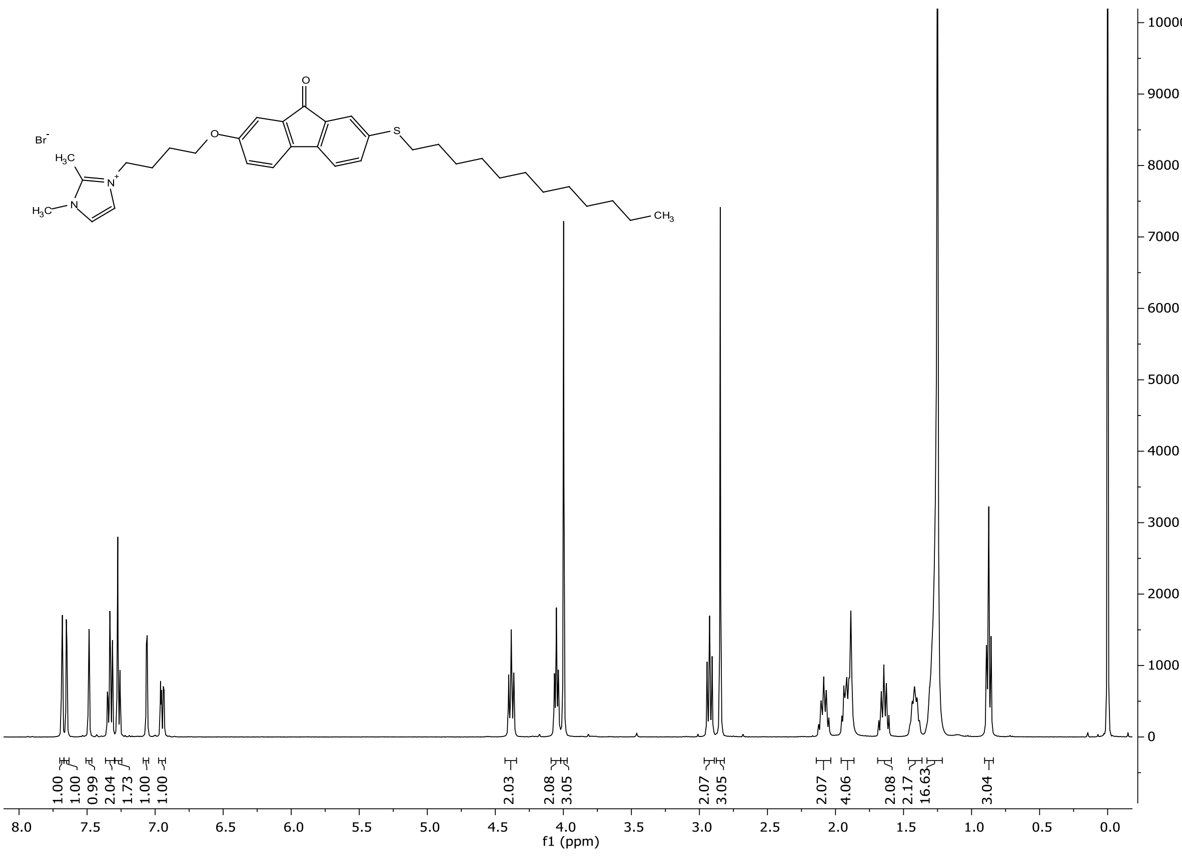
Br⁻
1.00
1.00
0.99
2.04
1.73
1.00
1.00
2.03
2.08
3.05
2.07
3.05
2.07
4.06
2.08
2.17
16.63
3.04
f1 (ppm)

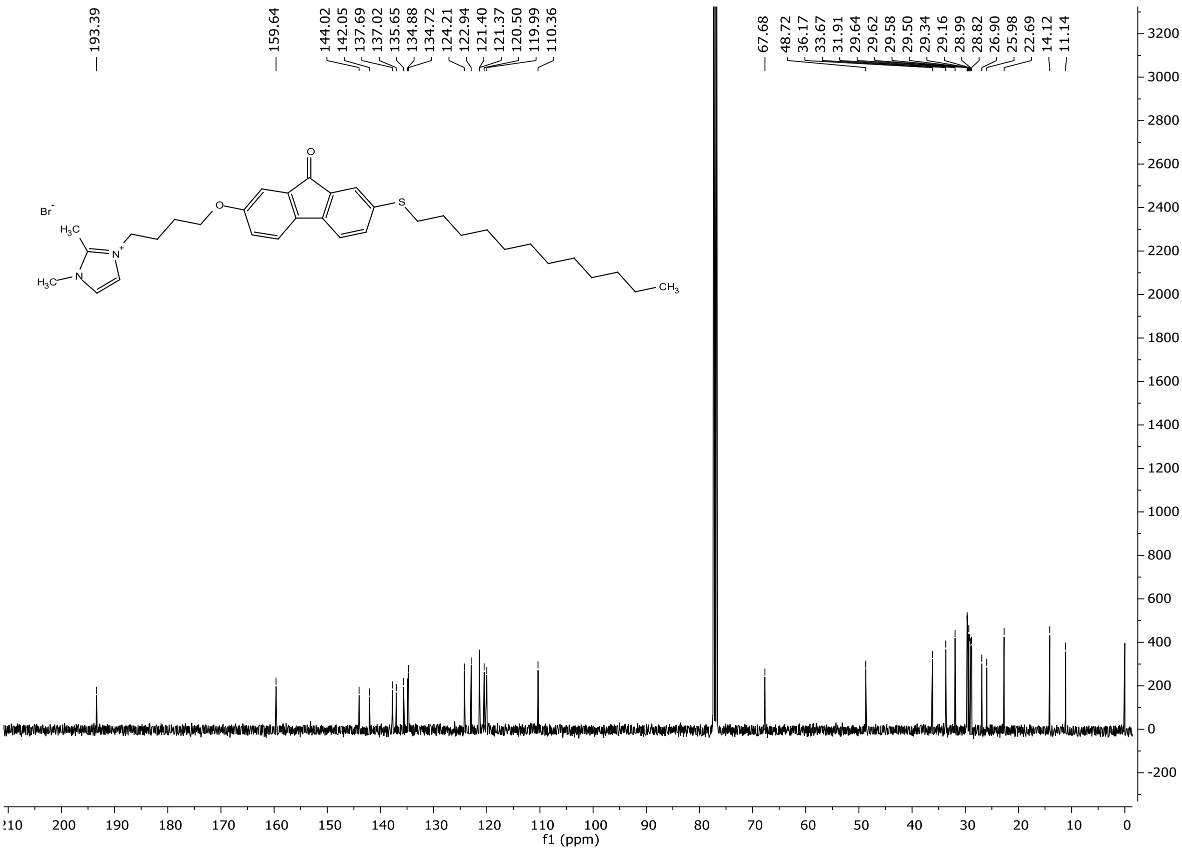
193.39
159.64
144.02
142.05
137.69
137.02
135.65
134.88
134.72
124.21
122.94
121.40
121.37
120.50
119.99
110.36
67.68
48.72
36.17
33.67
31.91
29.64
29.62
29.58
29.50
29.34
29.16
28.99
28.82
26.90
25.98
22.69
14.12
11.14
f1 (ppm)

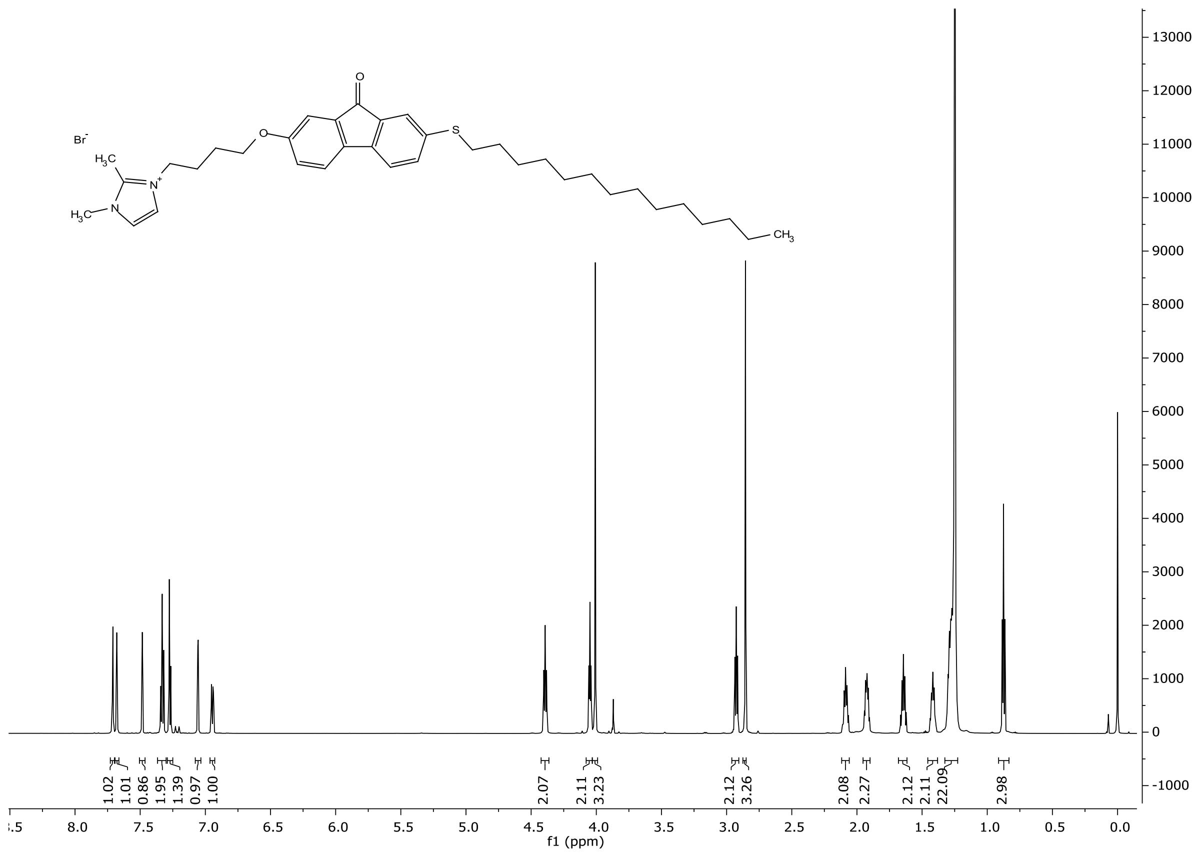

Br-
H3C
N+
N
H3C
O
O
S
CH3
1.02
1.01
0.86
1.95
1.39
0.97
1.00
2.07
2.11
3.23
2.12
3.26
2.08
2.27
2.12
2.11
22.09
2.98
f1 (ppm)

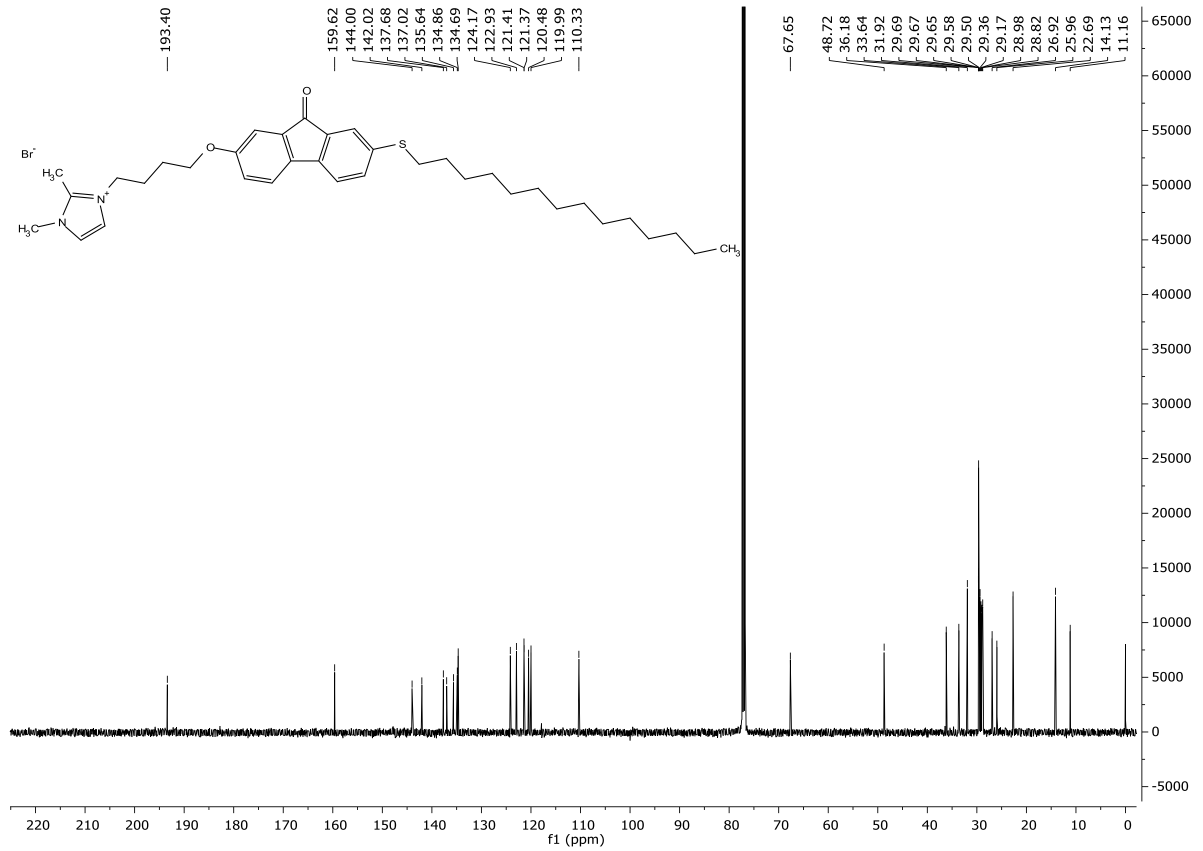

193.40
159.62
144.00
142.02
137.68
137.02
135.64
134.86
134.69
124.17
122.93
121.41
121.37
120.48
119.99
110.33
67.65
48.72
36.18
33.64
31.92
29.69
29.67
29.65
29.58
29.50
29.36
29.17
28.98
28.82
26.92
25.96
22.69
14.13
11.16
Br-
H3C
N+
N
H3C
O
O
S
CH3
f1 (ppm)

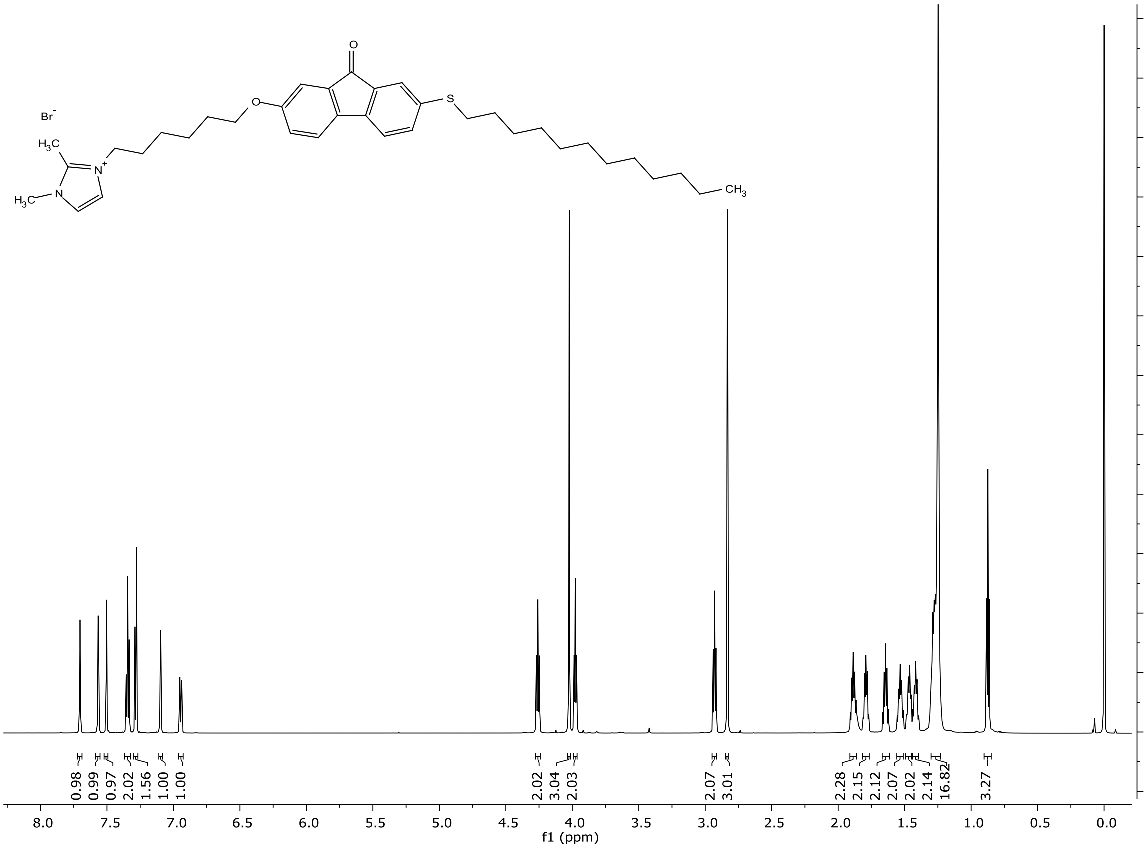
Br⁻
H3C
N+
N
H3C
O
O
S
CH3
0.98
0.99
0.97
2.02
1.56
1.00
1.00
2.02
3.04
2.03
2.07
3.01
2.28
2.15
2.12
2.07
2.02
2.14
16.82
3.27
f1 (ppm)

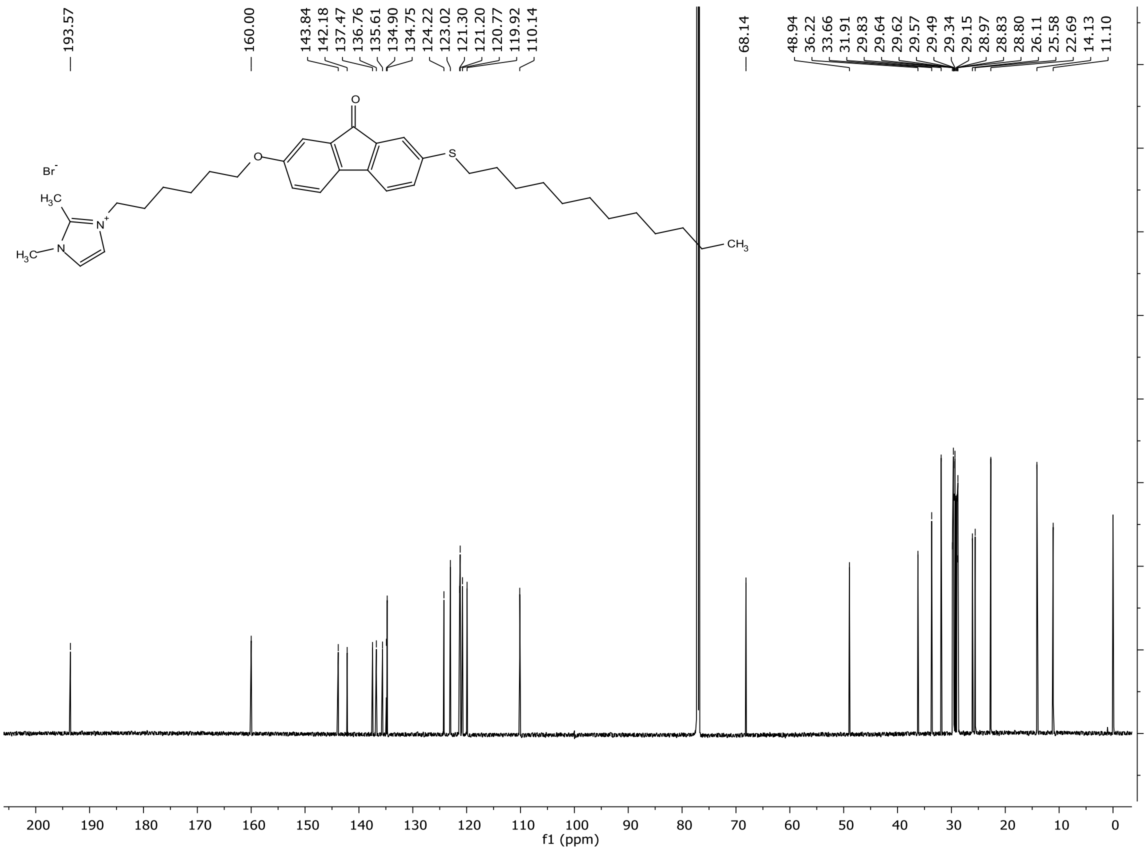
193.57
160.00
143.84
142.18
137.47
136.76
135.61
134.90
134.75
124.22
123.02
121.30
121.20
120.77
119.92
110.14
68.14
48.94
36.22
33.66
31.91
29.83
29.64
29.62
29.57
29.49
29.34
29.15
28.97
28.83
28.80
26.11
25.58
22.69
14.13
11.10
Br⁻
H3C
N+
N
H3C
O
O
S
CH3
f1 (ppm)

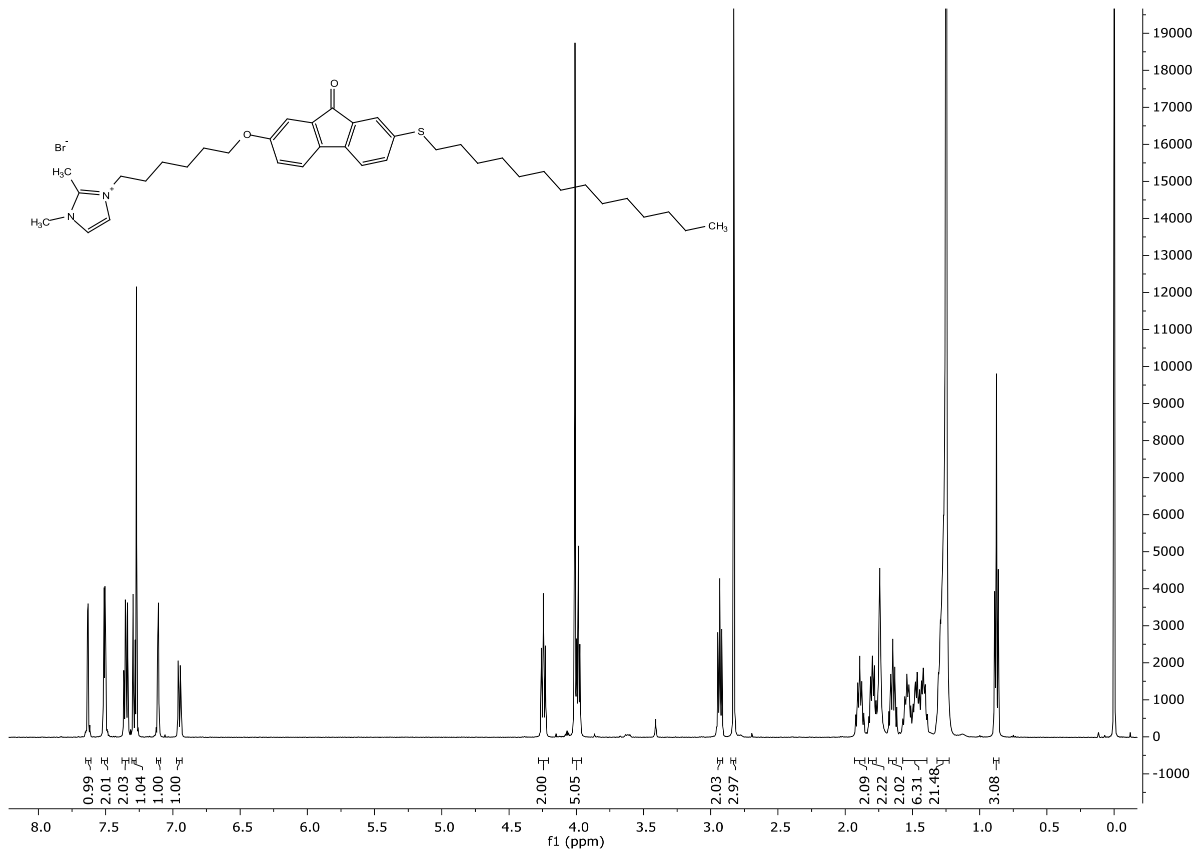

Br-
H3C
N+
H3C
N
O
O
S
CH3
0.99
2.01
2.03
1.04
1.00
1.00
2.00
5.05
2.03
2.97
2.09
2.22
2.02
6.31
21.48
3.08
f1 (ppm)

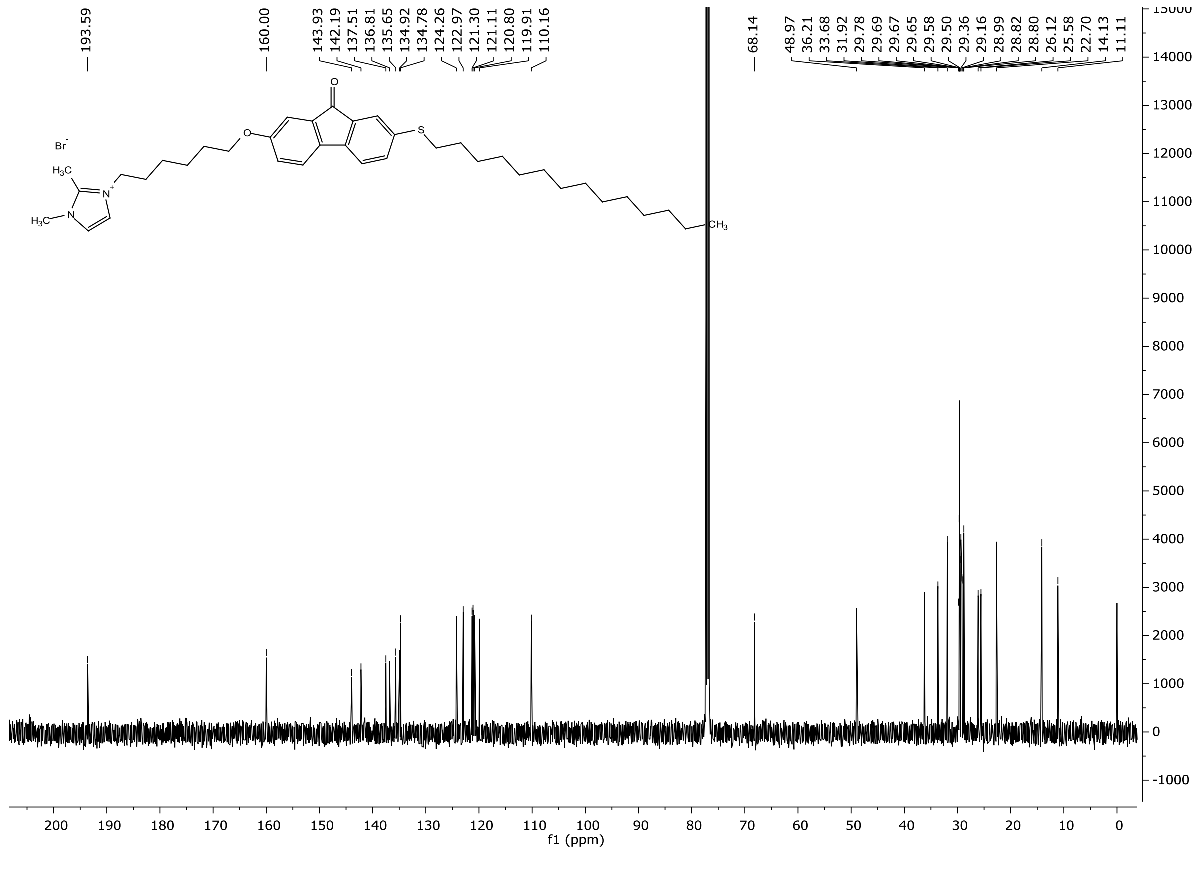

193.59
160.00
143.93
142.19
137.51
136.81
135.65
134.92
134.78
124.26
122.97
121.30
121.11
120.80
119.91
110.16
68.14
48.97
36.21
33.68
31.92
29.78
29.69
29.67
29.65
29.58
29.50
29.36
29.16
28.99
28.82
28.80
26.12
25.58
22.70
14.13
11.11
f1 (ppm)

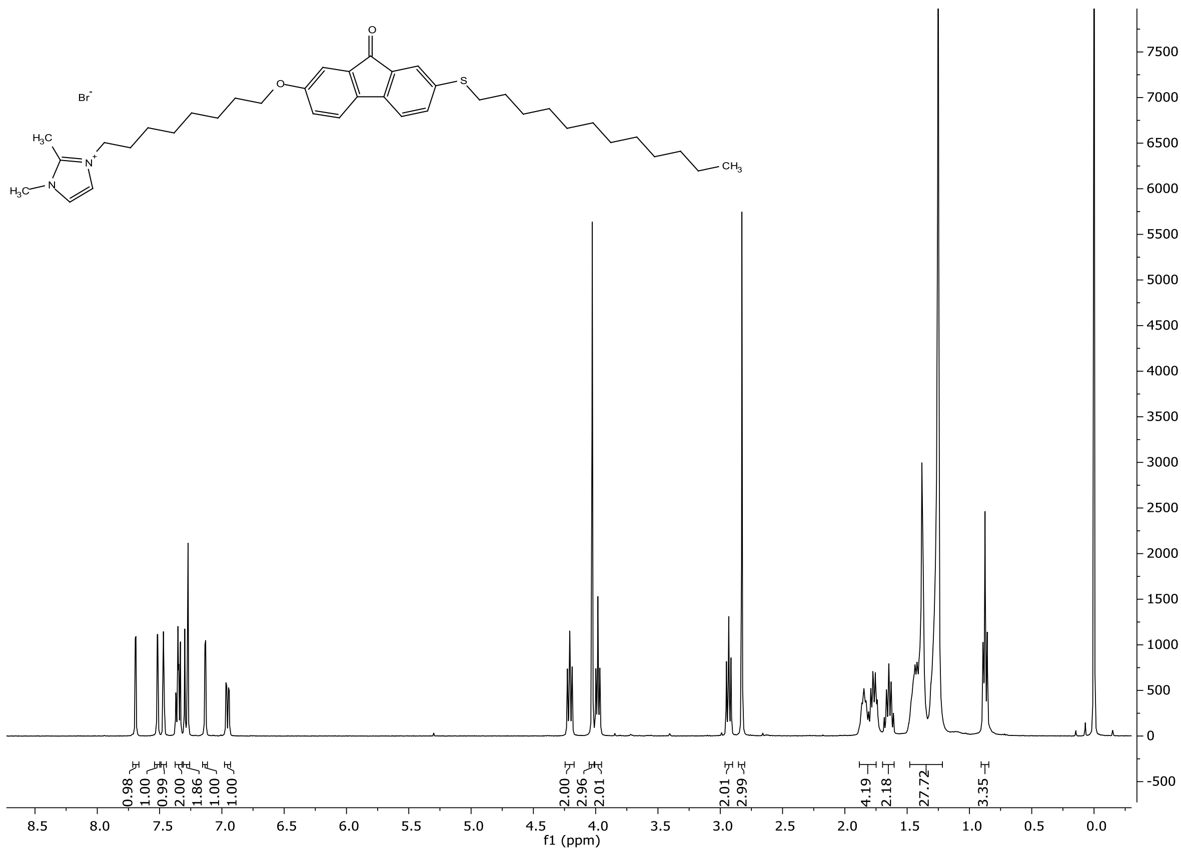

Br-
H_3C
N^+
N
H_3C
O
O
S
CH_3
0.98
1.00
0.99
2.00
1.86
1.00
1.00
2.00
2.96
2.01
2.01
2.99
4.19
2.18
27.72
3.35
f1 (ppm)

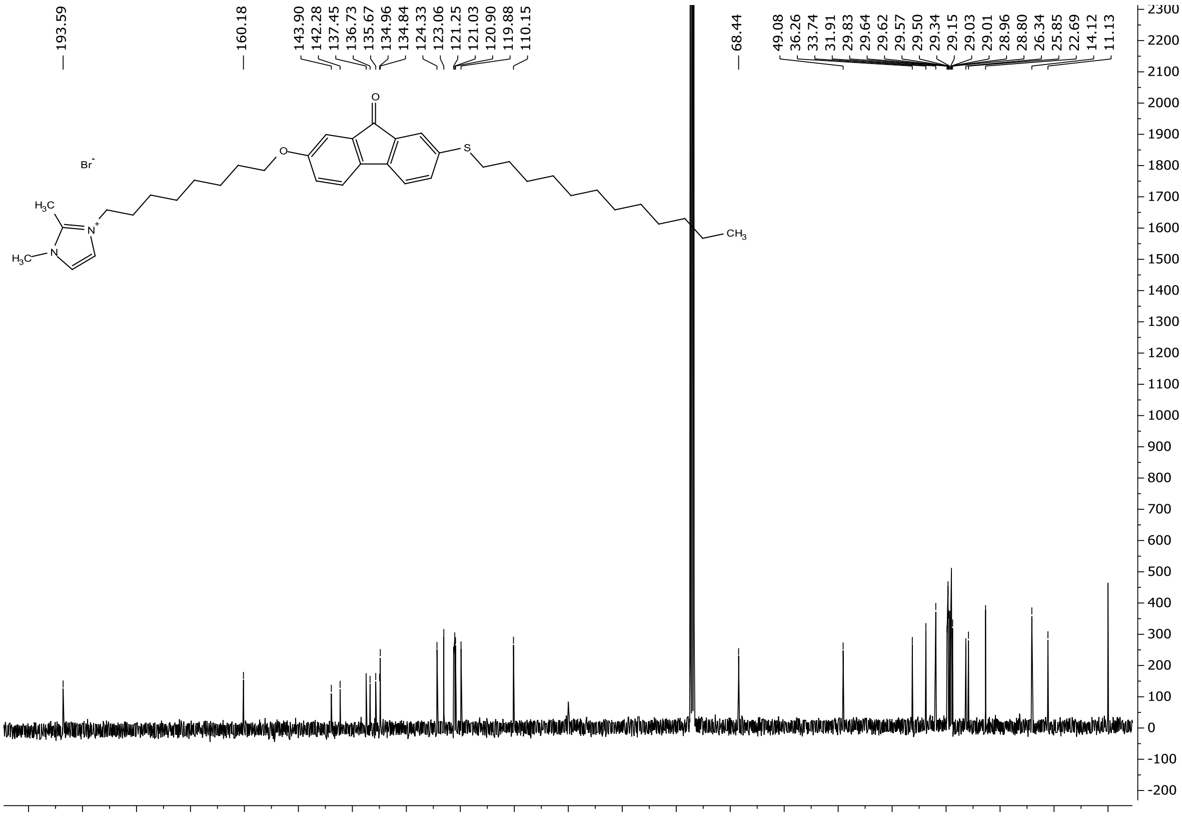

193.59
160.18
143.90
142.28
137.45
136.73
135.67
134.96
134.84
124.33
123.06
121.25
121.03
120.90
119.88
110.15
68.44
49.08
36.26
33.74
31.91
29.83
29.64
29.62
29.57
29.50
29.34
29.15
29.03
29.01
28.96
28.80
26.34
25.85
22.69
14.12
11.13
Br-
H_3C
N^+
N
H_3C
O
O
S
CH_3
f1 (ppm)

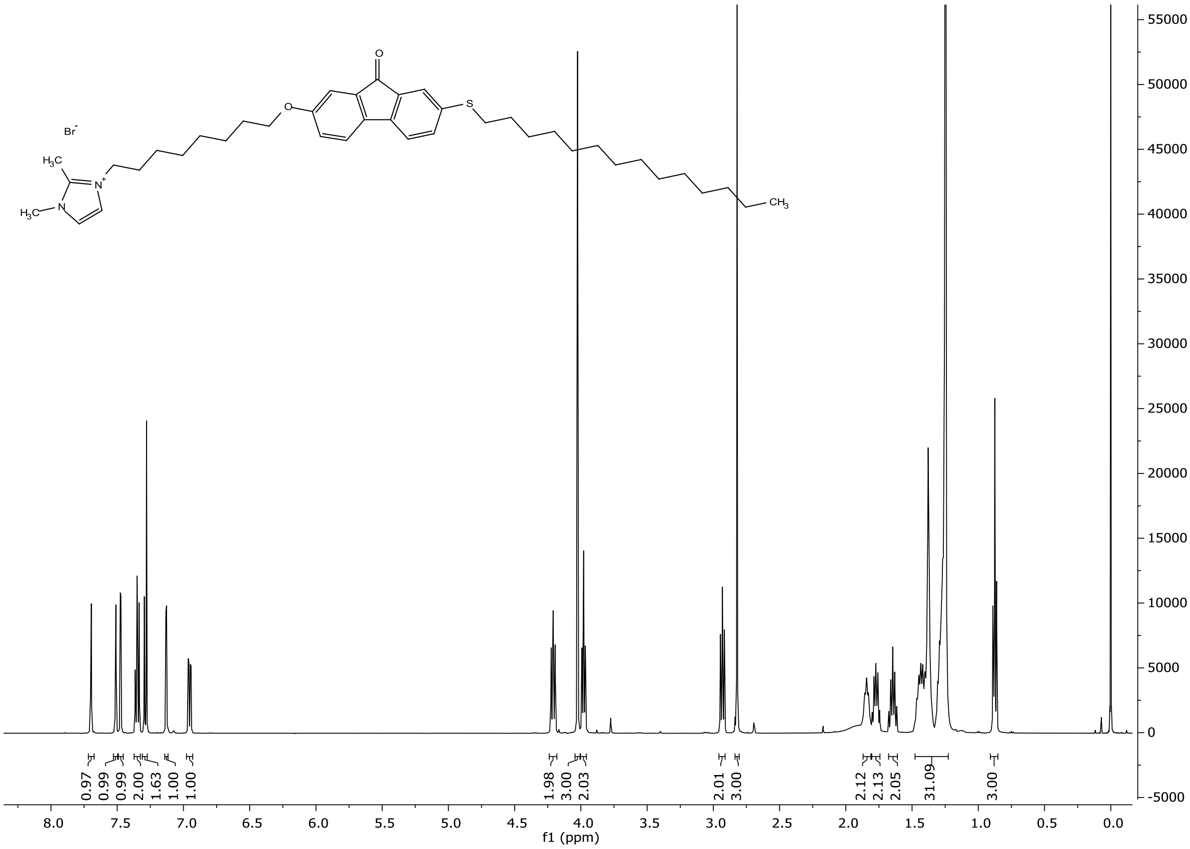
Br-
H3C
N+
N
H3C
O
O
S
CH3
0.97
0.99
0.99
2.00
1.63
1.00
1.00
1.98
3.00
2.03
2.01
3.00
2.12
2.13
2.05
31.09
3.00
f1 (ppm)

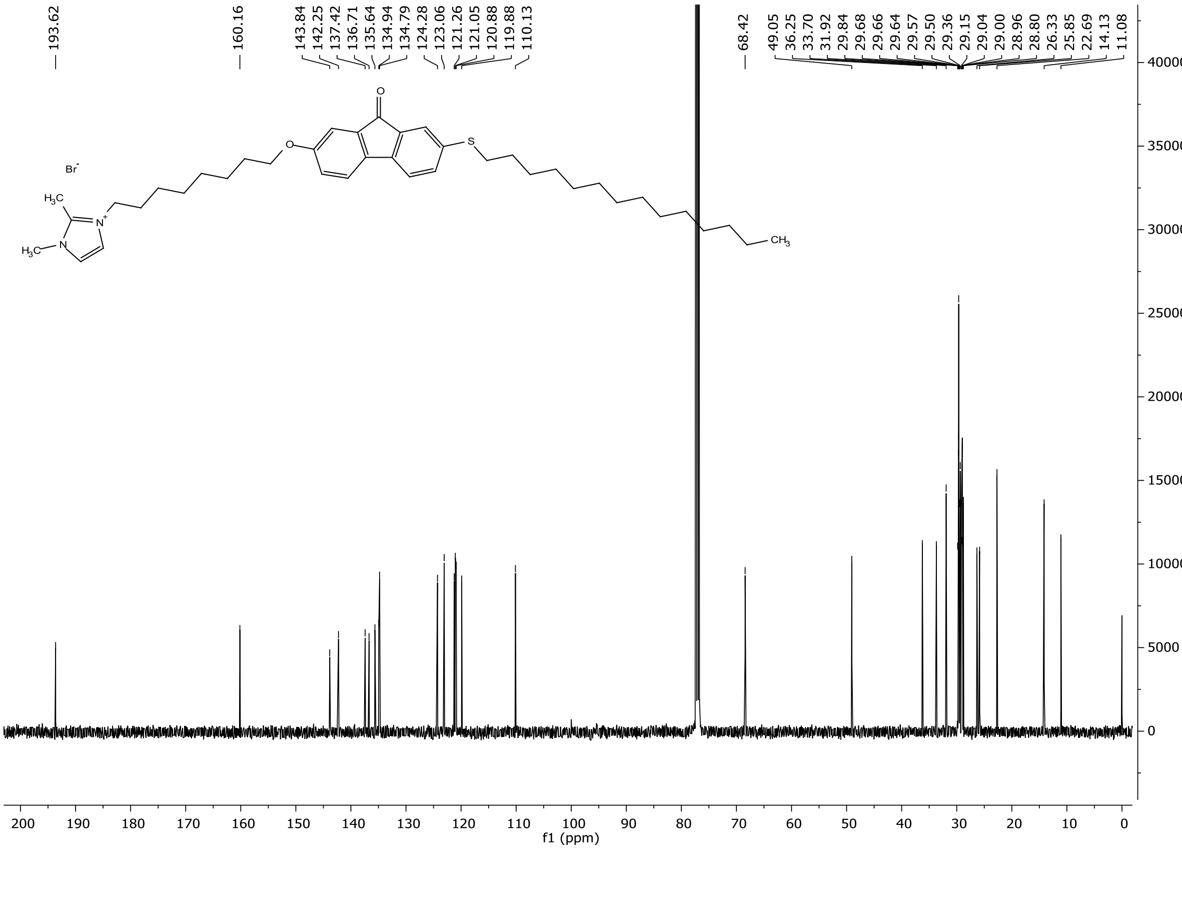
193.62
160.16
143.84
142.25
137.42
136.71
135.64
134.94
134.79
124.28
123.06
121.26
121.05
120.88
119.88
110.13
68.42
49.05
36.25
33.70
31.92
29.84
29.68
29.66
29.64
29.57
29.50
29.36
29.15
29.04
29.00
28.96
28.80
26.33
25.85
22.69
14.13
11.08
Br-
H3C
N+
N
H3C
O
O
S
CH3
f1 (ppm)

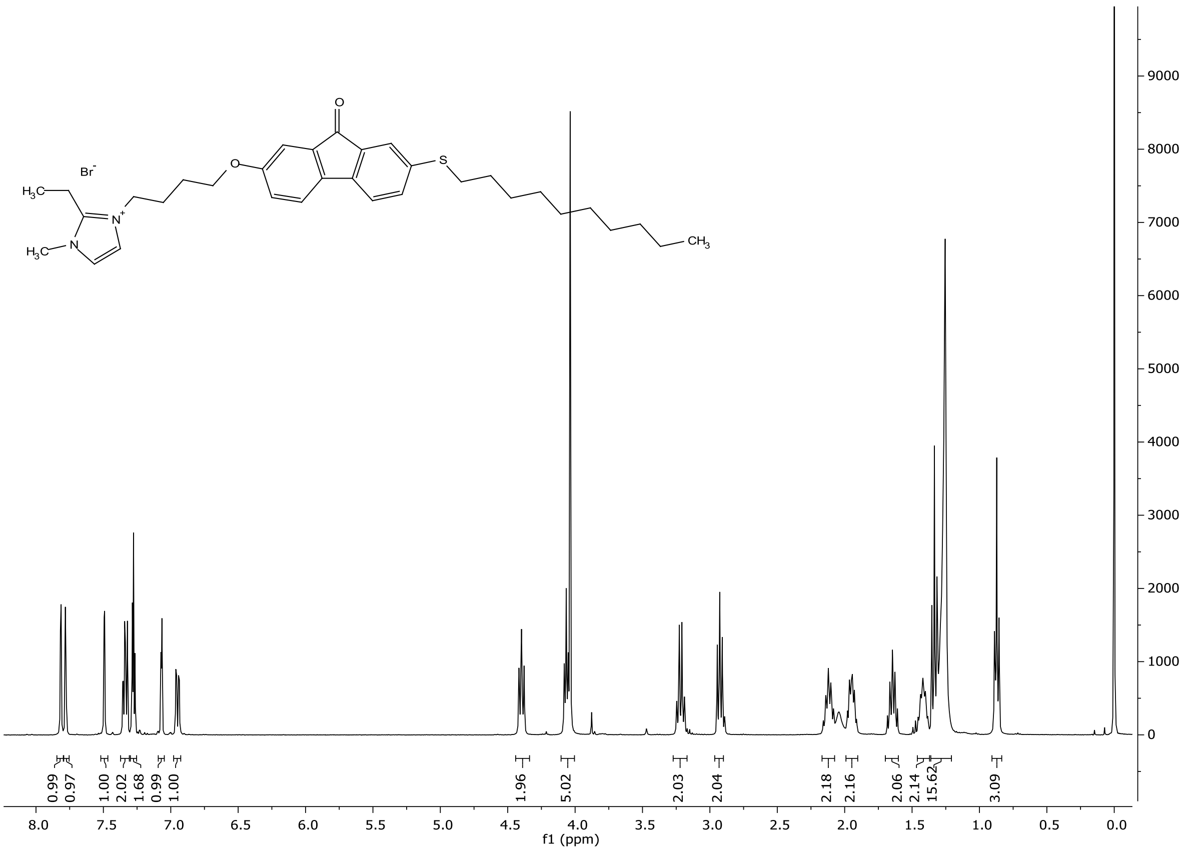

Br-
H_3C
N^+
N
H_3C
O
O
S
CH_3
0.99
0.97
1.00
2.02
1.68
0.99
1.00
1.96
5.02
2.03
2.04
2.18
2.16
2.06
2.14
15.62
3.09
f1 (ppm)

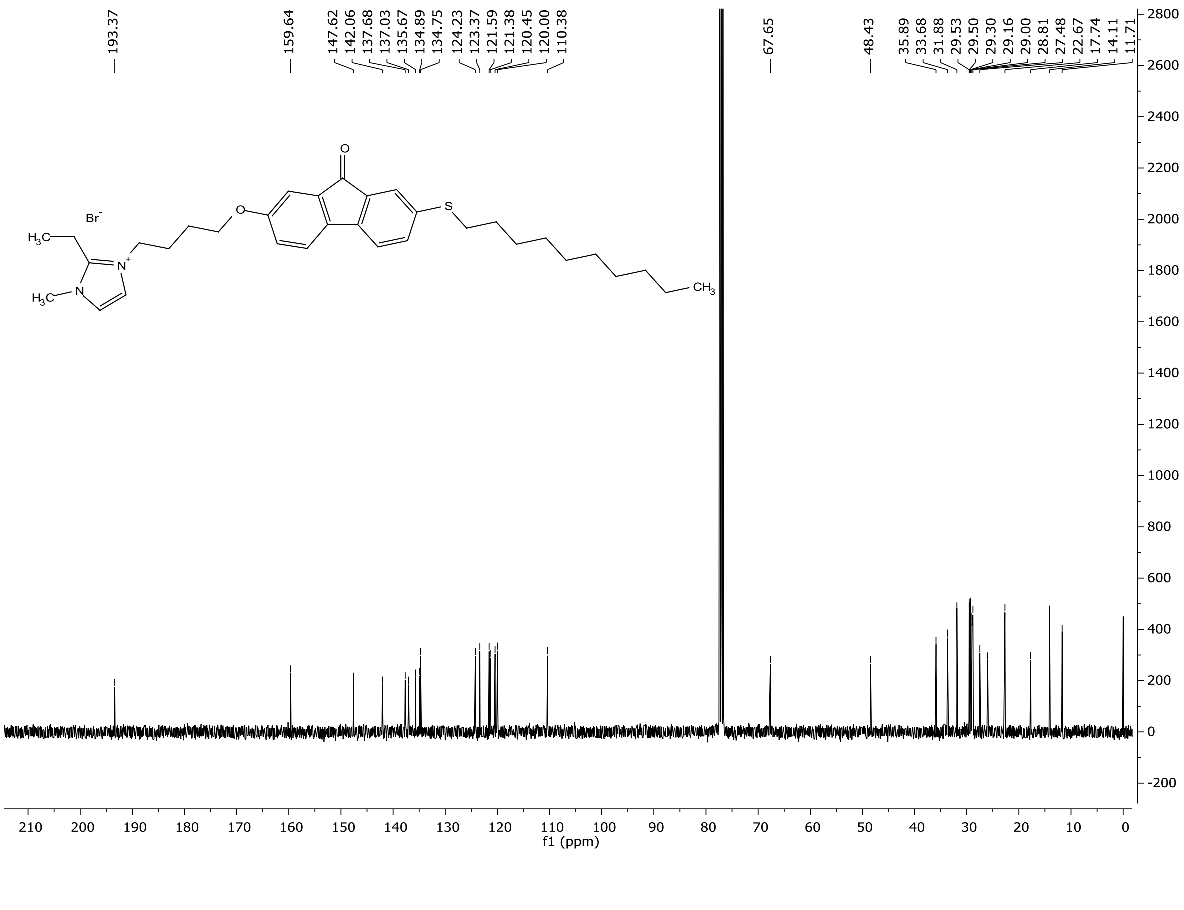

193.37
159.64
147.62
142.06
137.68
137.03
135.67
134.89
134.75
124.23
123.37
121.59
121.38
120.45
120.00
110.38
67.65
48.43
35.89
33.68
31.88
29.53
29.50
29.30
29.16
29.00
28.81
27.48
22.67
17.74
14.11
11.71
Br-
H_3C
N^+
N
H_3C
O
O
S
CH_3
f1 (ppm)

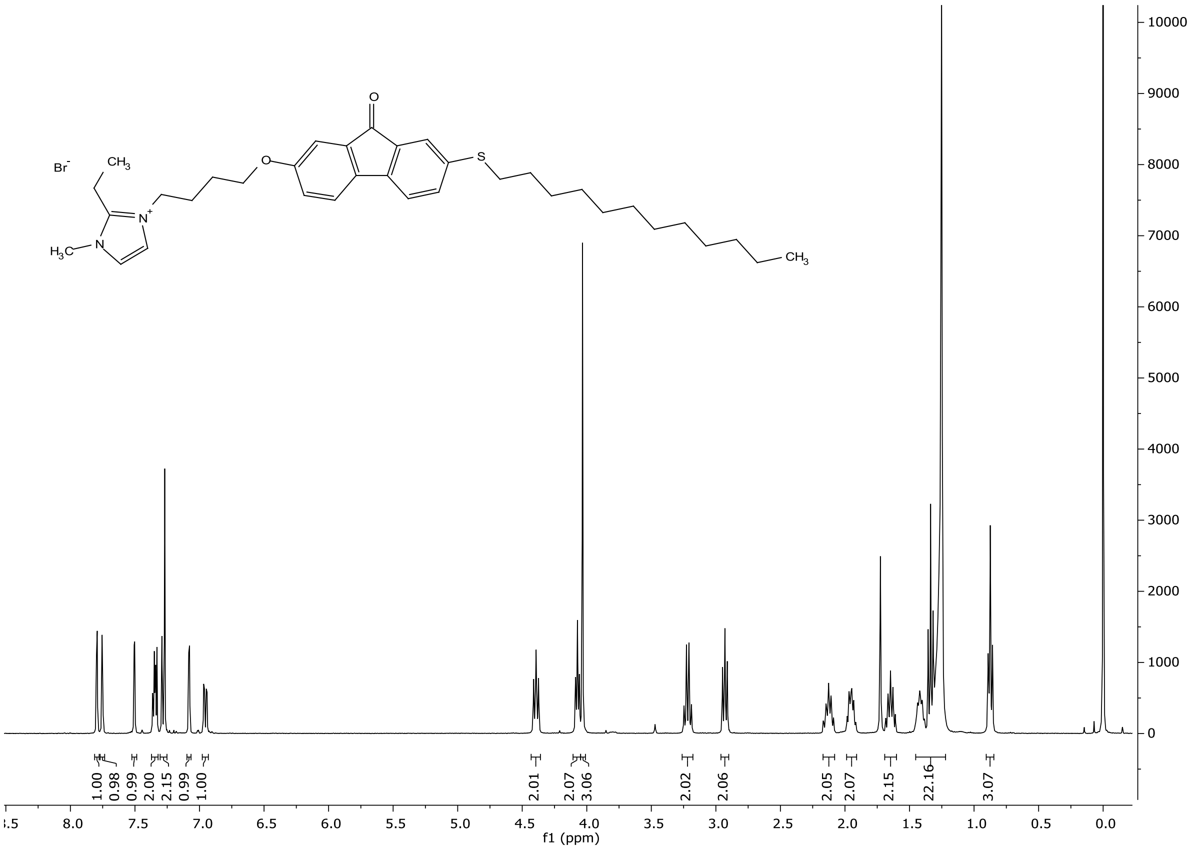
Br⁻
CH3
N+
N
H3C
O
O
S
CH3
1.00
0.98
0.99
2.00
2.15
0.99
1.00
2.01
2.07
3.06
2.02
2.06
2.05
2.07
2.15
22.16
3.07
f1 (ppm)

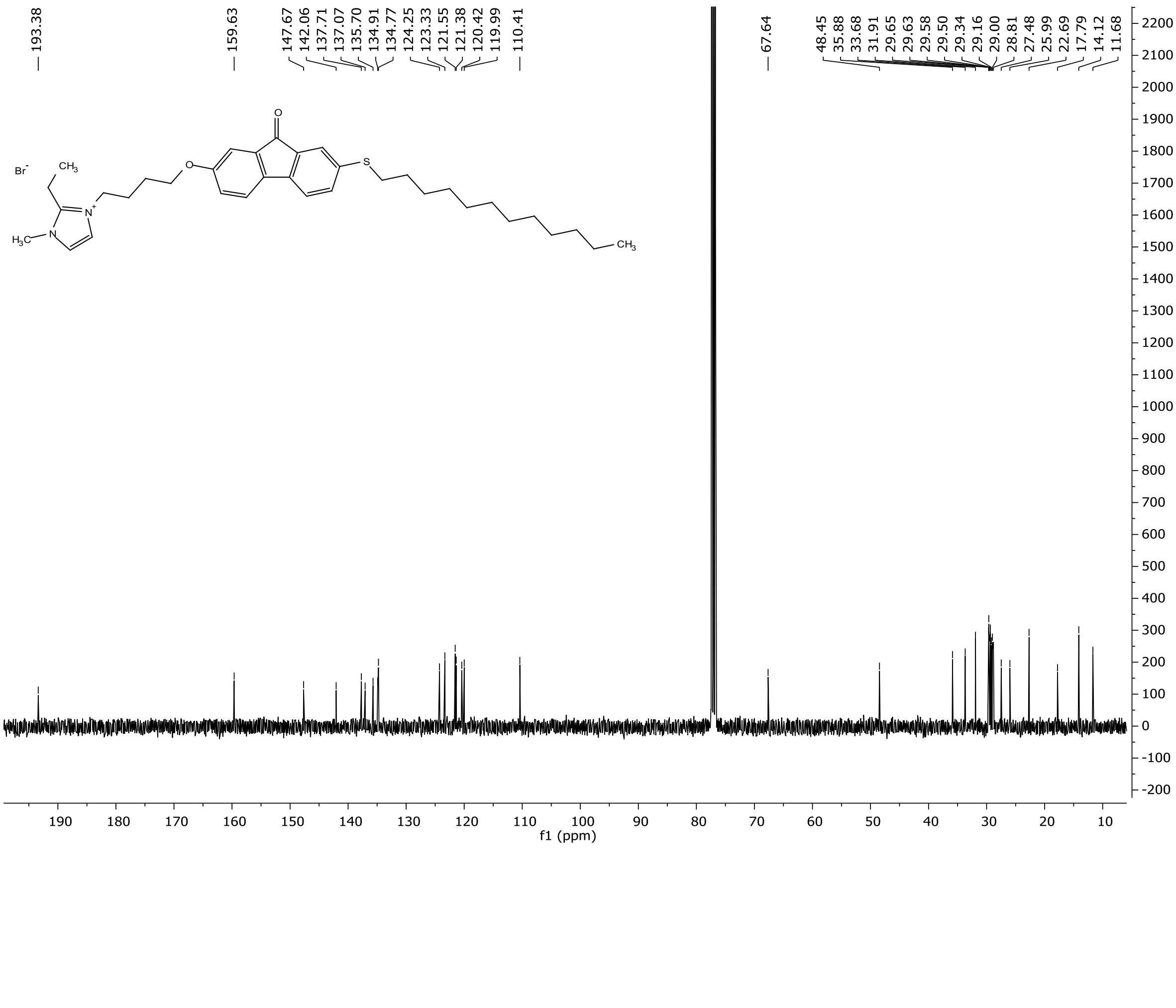
193.38
159.63
147.67
142.06
137.71
137.07
135.70
134.91
134.77
124.25
123.33
121.55
121.38
120.42
119.99
110.41
67.64
48.45
35.88
33.68
31.91
29.65
29.63
29.58
29.50
29.34
29.16
29.00
28.81
27.48
25.99
22.69
17.79
14.12
11.68
f1 (ppm)

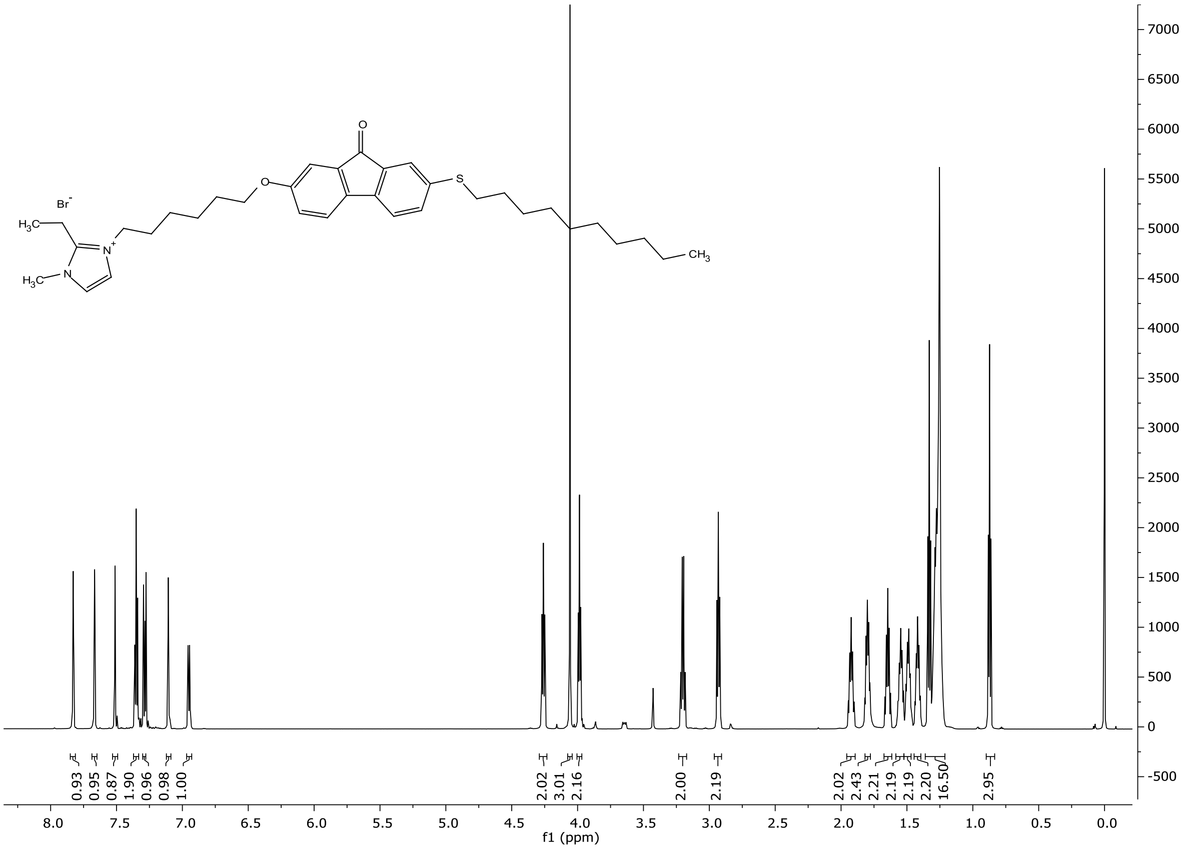

Br⁻
O
S
0.93
0.95
0.87
1.90
0.96
0.98
1.00
2.02
3.01
2.16
2.00
2.19
2.02
2.43
2.21
2.19
2.19
2.20
16.50
2.95
f1 (ppm)

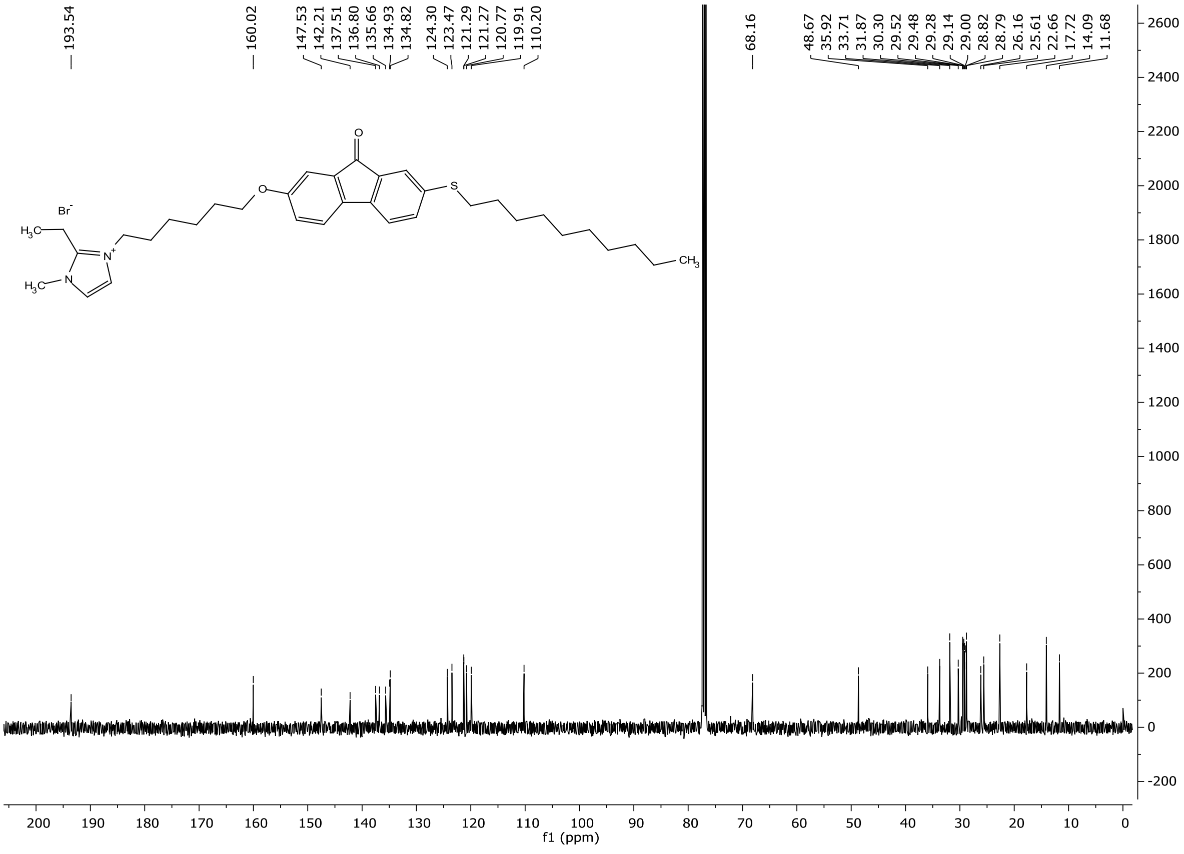

193.54
160.02
147.53
142.21
137.51
136.80
135.66
134.93
134.82
124.30
123.47
121.29
121.27
120.77
119.91
110.20
68.16
48.67
35.92
33.71
31.87
30.30
29.52
29.48
29.28
29.14
29.00
28.82
28.79
26.16
25.61
22.66
17.72
14.09
11.68
f1 (ppm)

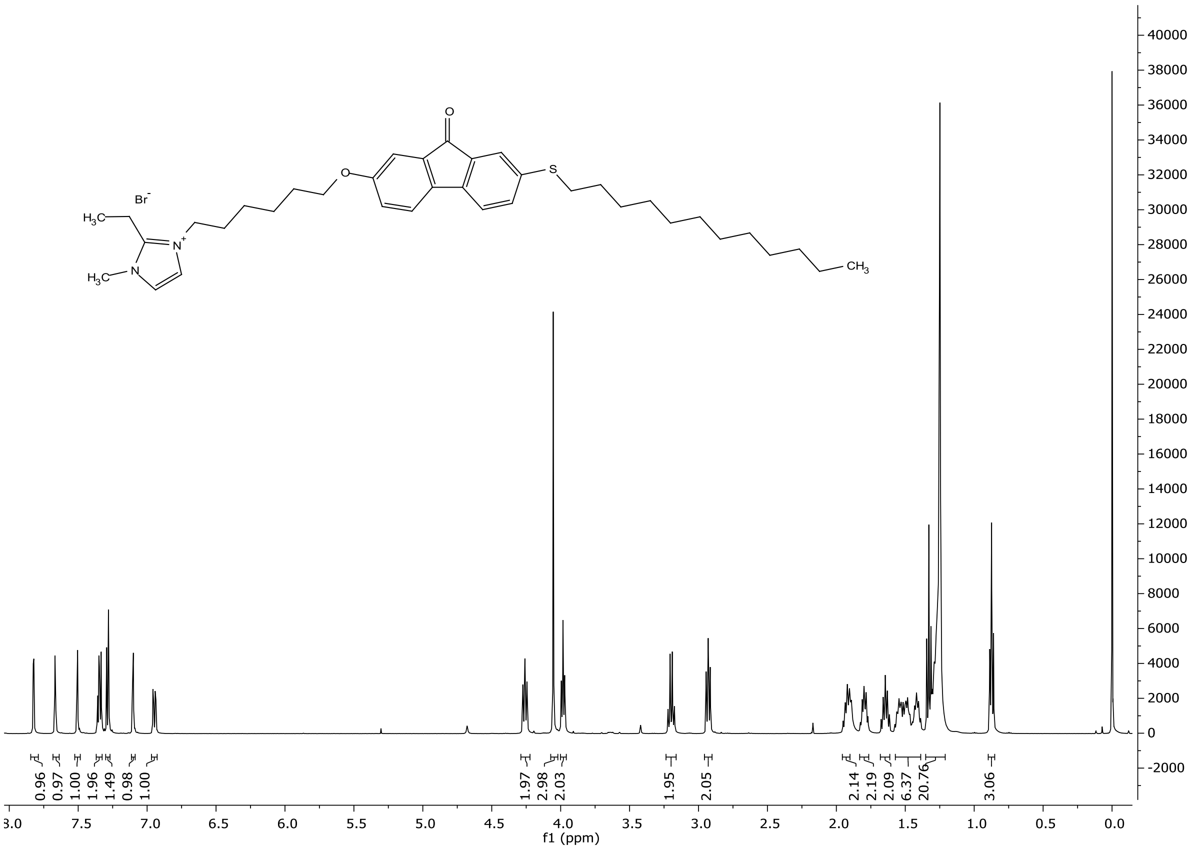

Br-
0.96
0.97
1.00
1.96
1.49
0.98
1.00
1.97
2.98
2.03
1.95
2.05
2.14
2.19
2.09
6.37
20.76
3.06
f1 (ppm)

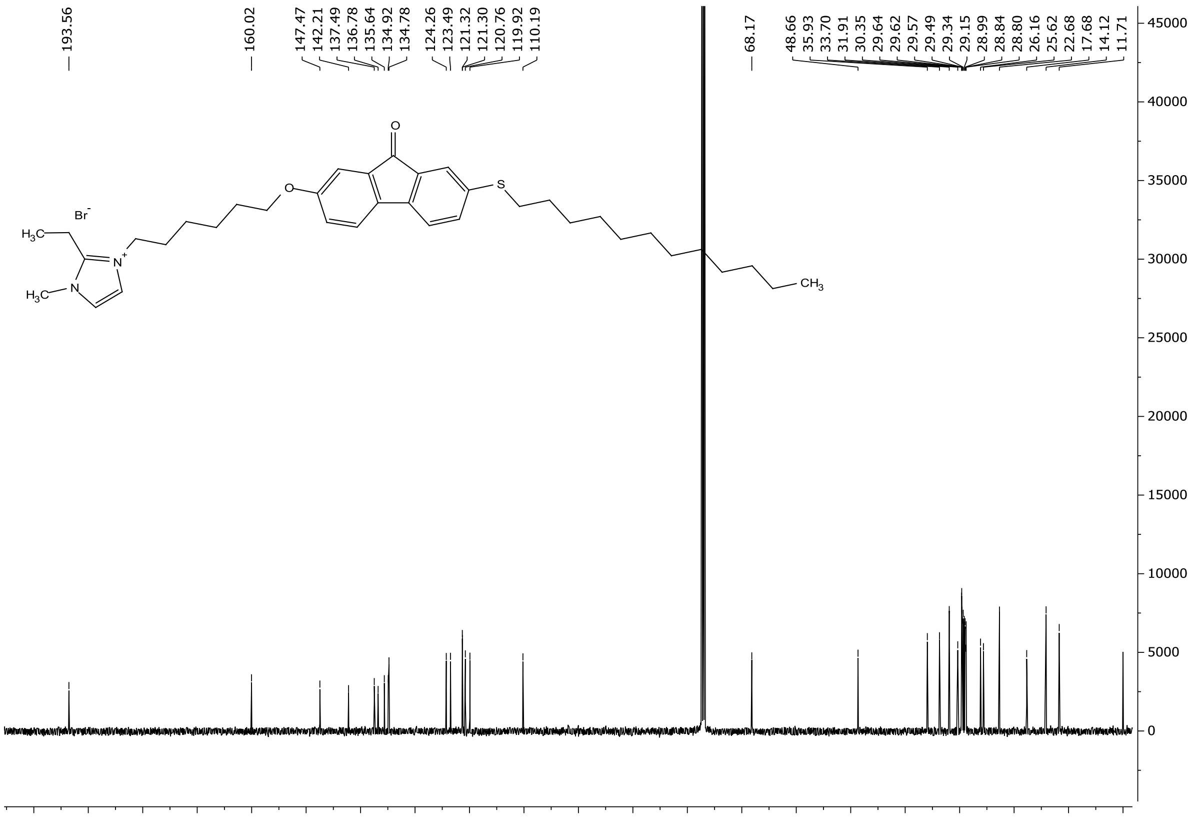

193.56
160.02
147.47
142.21
137.49
136.78
135.64
134.92
134.78
124.26
123.49
121.32
121.30
120.76
119.92
110.19
68.17
48.66
35.93
33.70
31.91
30.35
29.64
29.62
29.57
29.49
29.34
29.15
28.99
28.84
28.80
26.16
25.62
22.68
17.68
14.12
11.71
Br-
f1 (ppm)

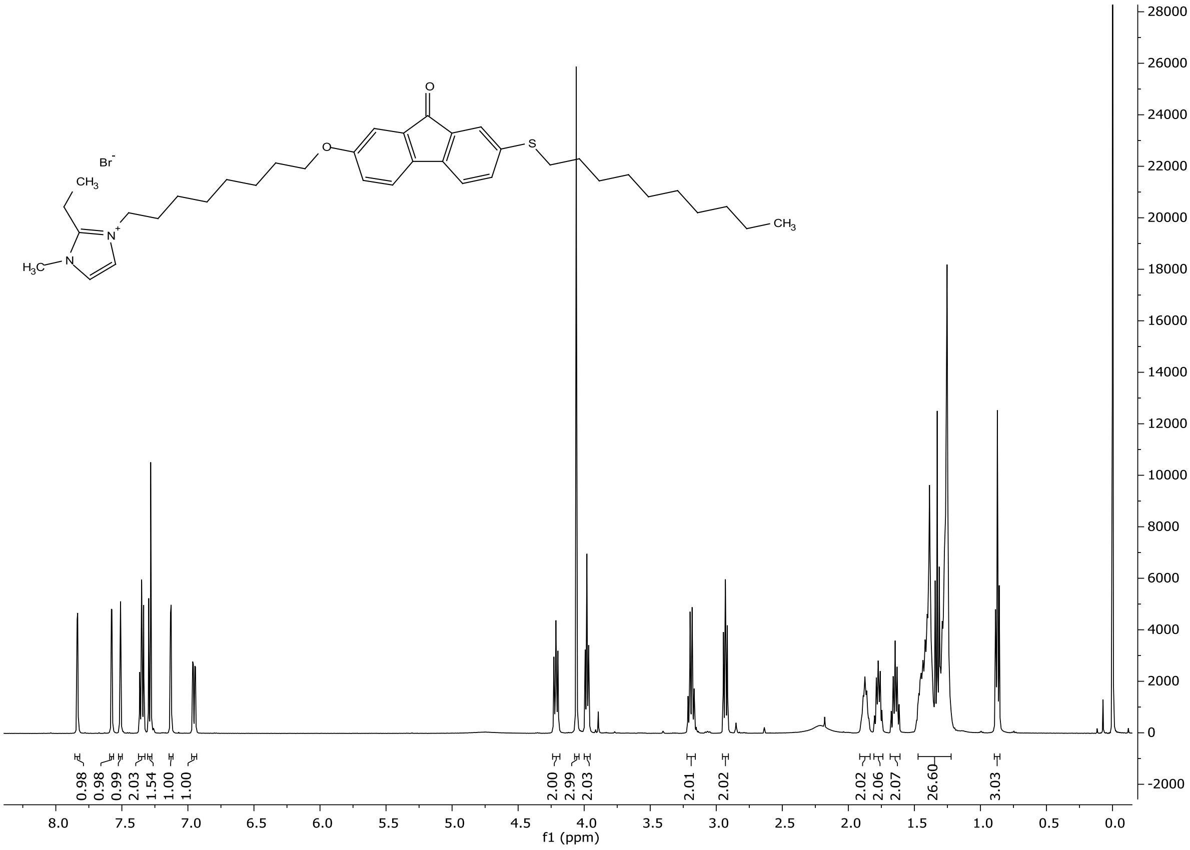

Br-
CH3
N+
N
H3C
O
O
S
CH3
0.98
0.98
0.99
2.03
1.54
1.00
1.00
2.00
2.99
2.03
2.01
2.02
2.02
2.06
2.07
26.60
3.03
f1 (ppm)

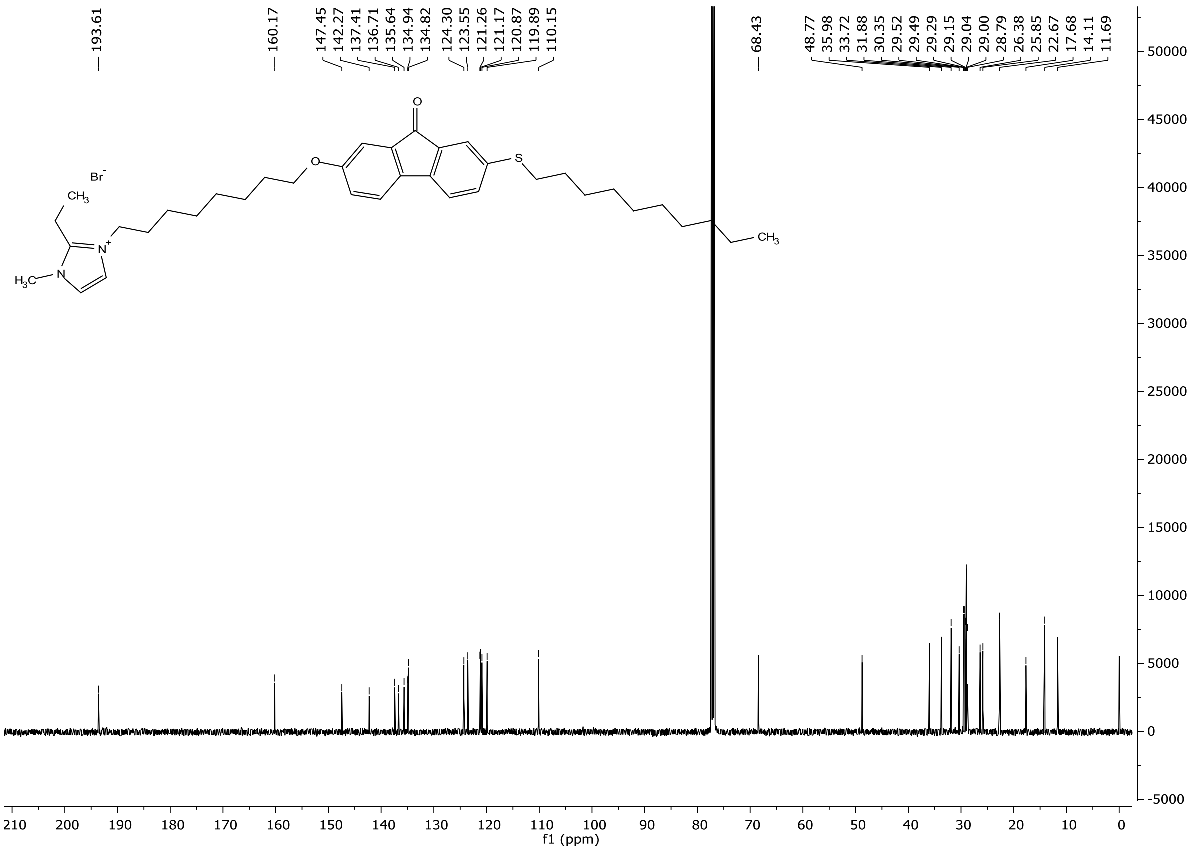

193.61
160.17
147.45
142.27
137.41
136.71
135.64
134.94
134.82
124.30
123.55
121.26
121.17
120.87
119.89
110.15
68.43
48.77
35.98
33.72
31.88
30.35
29.52
29.49
29.29
29.15
29.04
29.00
28.79
26.38
25.85
22.67
17.68
14.11
11.69
Br-
CH3
N+
N
H3C
O
O
S
CH3
f1 (ppm)

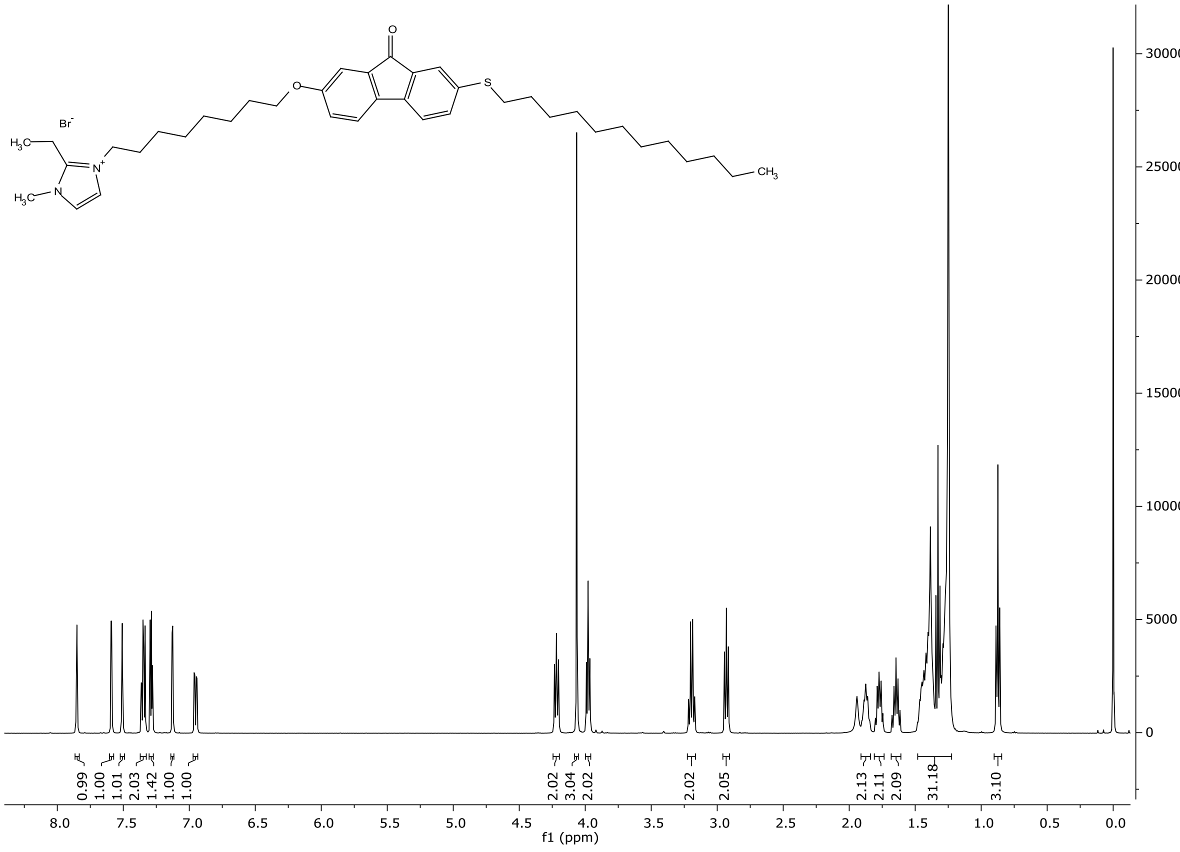

0.99
1.00
1.01
2.03
1.42
1.00
1.00
2.02
3.04
2.02
2.02
2.05
2.13
2.11
2.09
31.18
3.10
f1 (ppm)

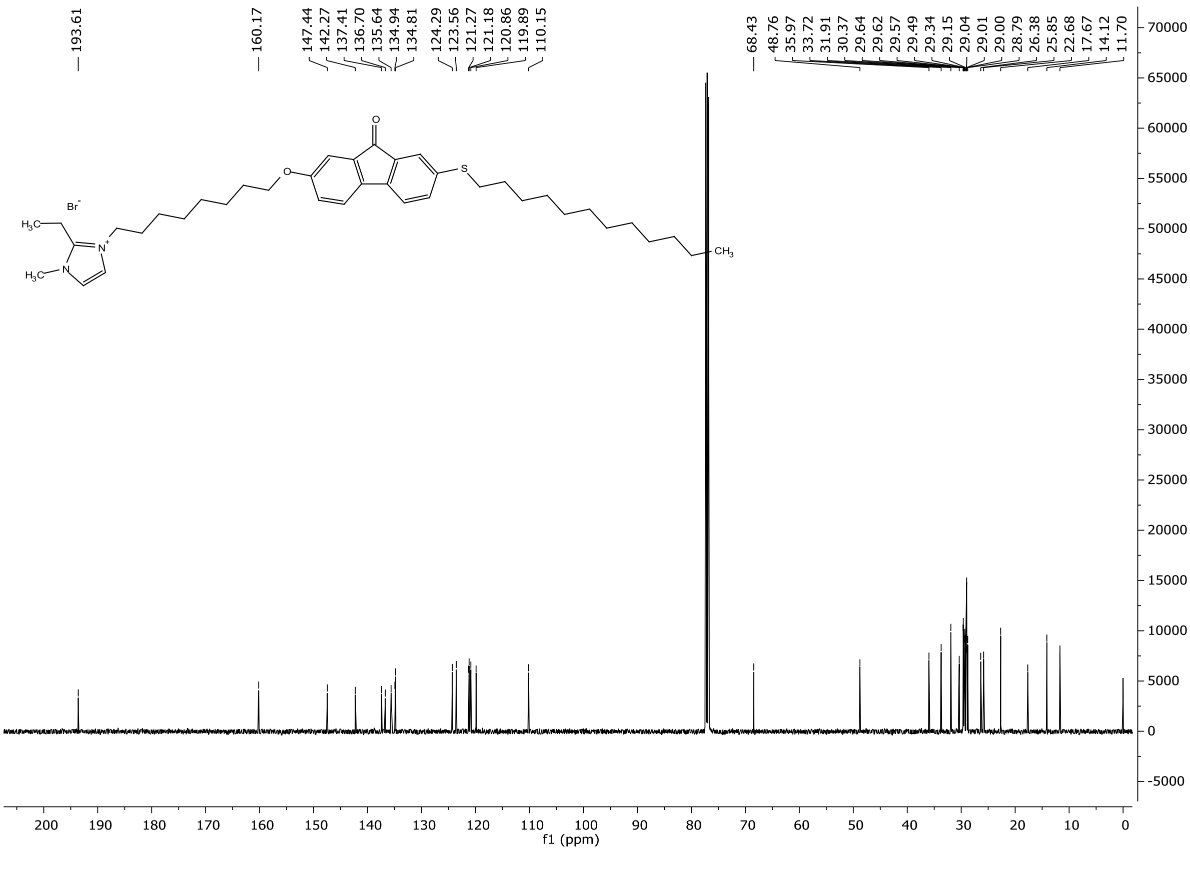

193.61
160.17
147.44
142.27
137.41
136.70
135.64
134.94
134.81
124.29
123.56
121.27
121.18
120.86
119.89
110.15
68.43
48.76
35.97
33.72
31.91
30.37
29.64
29.62
29.57
29.49
29.34
29.15
29.04
29.01
29.00
28.79
26.38
25.85
22.68
17.67
14.12
11.70
f1 (ppm)
